# Automatic brain lesion segmentation on standard magnetic resonance images: a scoping review

**DOI:** 10.1136/bmjopen-2020-042660

**Published:** 2021-01-28

**Authors:** Emilia Gryska, Justin Schneiderman, Isabella Björkman-Burtscher, Rolf A Heckemann

**Affiliations:** 1 Medical Radiation Sciences, Goteborgs universitet Institutionen for kliniska vetenskaper, Goteborg, Sweden; 2 Sektionen för klinisk neurovetenskap, Goteborgs Universitet Institutionen for Neurovetenskap och fysiologi, Goteborg, Sweden; 3 Radiology, Göteborgs universitet Institutionen för kliniska vetenskaper, Goteborg, Västra Götaland, Sweden

**Keywords:** radiology & imaging, magnetic resonance imaging, diagnostic radiology, neuroradiology

## Abstract

**Objectives:**

Medical image analysis practices face challenges that can potentially be addressed with algorithm-based segmentation tools. In this study, we map the field of automatic MR brain lesion segmentation to understand the clinical applicability of prevalent methods and study designs, as well as challenges and limitations in the field.

**Design:**

Scoping review.

**Setting:**

Three databases (PubMed, IEEE Xplore and Scopus) were searched with tailored queries. Studies were included based on predefined criteria. Emerging themes during consecutive title, abstract, methods and whole-text screening were identified. The full-text analysis focused on materials, preprocessing, performance evaluation and comparison.

**Results:**

Out of 2990 unique articles identified through the search, 441 articles met the eligibility criteria, with an estimated growth rate of 10% per year. We present a general overview and trends in the field with regard to publication sources, segmentation principles used and types of lesions. Algorithms are predominantly evaluated by measuring the agreement of segmentation results with a trusted reference. Few articles describe measures of clinical validity.

**Conclusions:**

The observed reporting practices leave room for improvement with a view to studying replication, method comparison and clinical applicability. To promote this improvement, we propose a list of recommendations for future studies in the field.

**Strengths and limitations of this study**

- This is the first overarching review of MR-based automatic brain lesion segmentation methods without restriction to a particular lesion type.
- The study was conducted following a previously published protocol.
- We consulted practitioners to ensure relevance of our work for clinical practice.
- The rigorous study design restricted our ability to expand on emerging themes, but did not entirely prevent epistemic drift.
- The restriction to a one-pass full-text analysis entailed a superficial level of interpretation—in hindsight a deeper reading would have been desirable.

## Introduction

MRI has become an integral part of diagnostics to detect, differentiate and characterise brain lesions. Properties that determine this success are safety, high tissue contrast and sensitivity to abnormality. Conditions that can lead to brain lesions include traumatic brain injury, vascular disease (including ischaemic and haemorrhagic stroke), neoplasms, autoimmune disorders, infection, degenerative diseases, congenital conditions and systemic diseases with secondary effects on the central nervous system. In some of these conditions, accurate estimation of lesion size and its progression may be essential to treatment planning, disease monitoring and/or treatment evaluation.

Visual image interpretation is still the most common and accepted way to analyse clinical images. In some cases, the visual examination must be extended to include lesion delineation. For example, the boundaries of a tumour and structures at risk need to be localised accurately for radiation therapy planning. Such delineation depends on a skilled rater, is time consuming, and is characterised by high variability between raters and high variability between repeated delineations on the same images by the same rater (ie, low objectivity and reliability, respectively). In other clinical examinations, surrogate metrics, which can be based on the largest perpendicular diameters^1 2^ or visual scoring,^3^ are used for estimating the size of a lesion rapidly. Such visual or area-based lesion volume estimates may not be accurate enough.^4^

Developments in magnetic resonance (MR) image acquisition procedures resulted in high-quality and complex images that capture a variety of structural and functional phenomena. On one hand, the images carry more information about the lesions, thereby enabling more accurate diagnosis. On the other hand, each image requires more analysis time. This reduces the rate at which radiologists can analyse images. The increasing accessibility to MR scanners furthermore results in more images being acquired, further straining image processing capacity and potentially diminishing the quality of interpretation.^5 6^

To summarise, three major issues with current clinical practices for image analysis are: subjective and time-consuming manual segmentation, limited accuracy of the surrogate volume estimates, and increasing information content and number of images to process.

Automatic lesion segmentation algorithms promise to alleviate these issues through fast and consistent lesion delineation that scales with demand. Quantitative image analysis using automatic lesion segmentation further has the potential to increase diagnostic and prognostic accuracy of lesion examination by providing radiologists with prompt and explicit information about a lesion.

Several reviews on automated brain lesion segmentation methods have previously been published. Most of these outline and analyse commonly used segmentation algorithms or general segmentation principles.^7–16^ The perspective of the clinical applicability of automated segmentation algorithms has been explored by Garcia-Lorenzo *et al*^10^ and Bauer *et al*.^7^ In both reviews, the authors recommend improvements concerning study designs and formulation of research questions (RQs). The authors stress the importance of validating the robustness of the segmentation algorithms under variation stemming from three causes: differences between scanners and acquisition protocols, natural variability in normal anatomy and lesion appearance, and artefacts. Moreover, Bauer *et al*^7^ expressed the need for better communication between researchers developing segmentation methods and clinical radiologists who are the intended users of these methods.

A comprehensive review of automatic segmentation methods without restriction to lesion type is still lacking today, perhaps because of the challenge associated with the large and growing literature. We therefore present a scoping review of such methods and, given the rapidly growing extent of the literature, provide a timely account of the field that may not be feasible in the future. To ensure rigour, reproducibility and comprehensiveness, we adopted scoping review methods proposed earlier.^17–21^

Furthermore, there is a disconnection between developers and users of automatic brain lesion segmentation methods.^7^ We therefore aim to examine clinical relevance of the research conducted in the field and seek to understand how the most prevalent methods (segmentation algorithms) and study designs (how they are validated) reflect the clinical applicability of methods described in the reviewed articles. Moreover, we aim to identify issues, limitations, and grand challenges of the field, and suggest actions to bridge the gap between research and clinical practice.

## Methods and design

This study has been conducted according to a previously published protocol^22^ and based on scoping review methods proposed by Arksey and O’Malley^17^ and further developed by Levac *et al* and Colquhoun *et al*.^18 19^ We also incorporate relevant parts of the Preferred Reporting Items for Systematic Reviews andMeta-Analyses (PRISMA)^20^ and PRISMA extension for Scoping Reviews^21^ protocol for this type of review. Here, we provide a summary of the protocol as well as an account of changes made, along with their respective rationale.

### Stage 1: identifying RQs

The following RQs were posed in the protocol^22^:

1. Which common image processing steps are necessary for automatic brain lesion segmentation on MR images?
2. Which mathematical and computational theories are most commonly applied in which types of brain lesions?
3. What is the efficacy of existing implementations?
4. What are the limitations of those methods and issues that should be addressed in future studies to develop a tool that is suitable for clinical use?
5. What are the most commonly used MR data sets that provide reference lesion segmentation and/or diagnostic classification?

While getting familiar with the abstracts of the sample, we questioned the relevance of RQs 1 and 2. We recognised the need for consultation at this early stage of the project as opposed to consultation on the findings as originally proposed by Arksey and O’Malley.^17^ The RQs do not reflect fully the potential of our study to address issues critical to advancing the field and bringing a benefit to the community. The consultation was conducted as semistructured interviews with five clinicians who have experience in brain image analysis. We interviewed two neurosurgeons, an oncologist, a radiation oncologist and a neuroradiologist. The interviews were structured to elicit understanding of their daily workflow, of how they use and analyse images, and of their opinions on automating the process of brain lesion segmentation. The initial screening of the sample abstracts and, in parallel, the consultations directed our analysis towards examining the clinical relevance of the prevalent methods and study designs.

### Stage 2: identifying relevant articles

For the purpose of this study, we define an article as an individual published item that was found according to the presented search strategy and that meets the inclusion criteria of this review.

Eligibility criteria were unchanged from those defined in the protocol: articles evaluating an automatic brain lesion segmentation method applied to MR images acquired for human brain lesion inspection, and articles evaluating the performance of the method by comparison with a reference segmentation were suitable for the study.

The initial search strategy remained mostly unchanged. Three databases (PubMed, IEEE Xplore and Scopus) were queried. Initially, a broad search phrase on automatic brain lesion segmentation methods on MRI was used to generate a first sample for each database. Controlled vocabulary tags were extracted from the articles and arranged in descending order of frequency. From this list, tags were selected to refine and customise the search phrase for each database. These refined and customised search phrases were used to retrieve the sample for our study. The search was conducted on 4 November 2018.

The relevant articles were identified through hierarchical screening at four levels with given aims and objectives:

1. Title level: exclusion of clearly ineligible articles.
2. Abstract level: refinement of inclusion and exclusion criteria based on literature content; identification of 100 eligible articles by reading of randomly selected abstracts out of a sample of 1359; identification of themes to guide the study selection process (stage 3).
3. Methods level: identification of two core concepts: algorithm validation and applicability of an automatic segmentation method for clinical parameter prediction; exclusion of studies using manual or semiautomatic segmentation, not stating the source of the tested images/patients, only using synthetic images, conducing the algorithm validation on fewer than 10 unique scans/images, or not providing results of the algorithm’s performance.
4. Full paper screening level (see stage 3).

The same hierarchical screening procedure was applied to references of the eligible sample also screening for duplicates and excluding conference proceedings that were later published in journal articles if the patient cohort was similar in both articles. Screening was discontinued on finding an exclusion criterion.

### Stage 3: study selection

In the full-text screening, we applied the above criteria again and excluded papers that:

- Did not perform segmentation or did not provide segmentation evaluation outcome measures (wrong outcome).
- Proposed methods that require user interaction.
- Did not provide information about the gold standard or a reference measurement.
- Did not provide information about the origin of the images or used only synthetic images.
- Did not provide the number of images used for evaluation.
- Evaluated the method on fewer than 10 unique scans.

### Stage 4: data charting

The data extracted were charted in a spreadsheet (online supplemental material) with five categories of variables:

10.1136/bmjopen-2020-042660.supp1 Supplementary data

1. Bibliographic category (first authors’ name, article title, journal or conference name, and year of publication).
2. Segmentation category (preprocessing procedures, algorithms and computational theories, if lesion classification used, required or allowed input modalities, processing time of the method and availability of the software).
3. Validation category (process of obtaining reference segmentations, number of raters or observers, and evaluation metrics).
4. Study cohort category comprised patients’ diagnoses, lesion type, sources and number of images).
5. General comments category (information that did not fit in other categories, but was considered relevant) stage 5: collating, summarising, and reporting the data.

### Stage 5: collating, summarising and reporting the data

The collected data are disseminated through numerical analysis of bibliographic information overview, trends in the field and study design characteristics. We provide the account of both prevalent study design choices and less common ones that are relevant to the clinical applicability assessment. Study design features are grouped into materials, preprocessing, performance evaluation and performance comparison.

### Patient and public involvement

No patients nor members of the public were involved in this research project.

## Results

The results of the scoping review include answers to RQs 2, 4 and 5. The answers to questions 1 and 5 are directly presented in the results. The answer to question 4 is formulated in the discussion based on the presented findings. In the discussion, we also justify why questions 1 and 3 could not be explicitly answered.

### Overview

**Figure 1:**
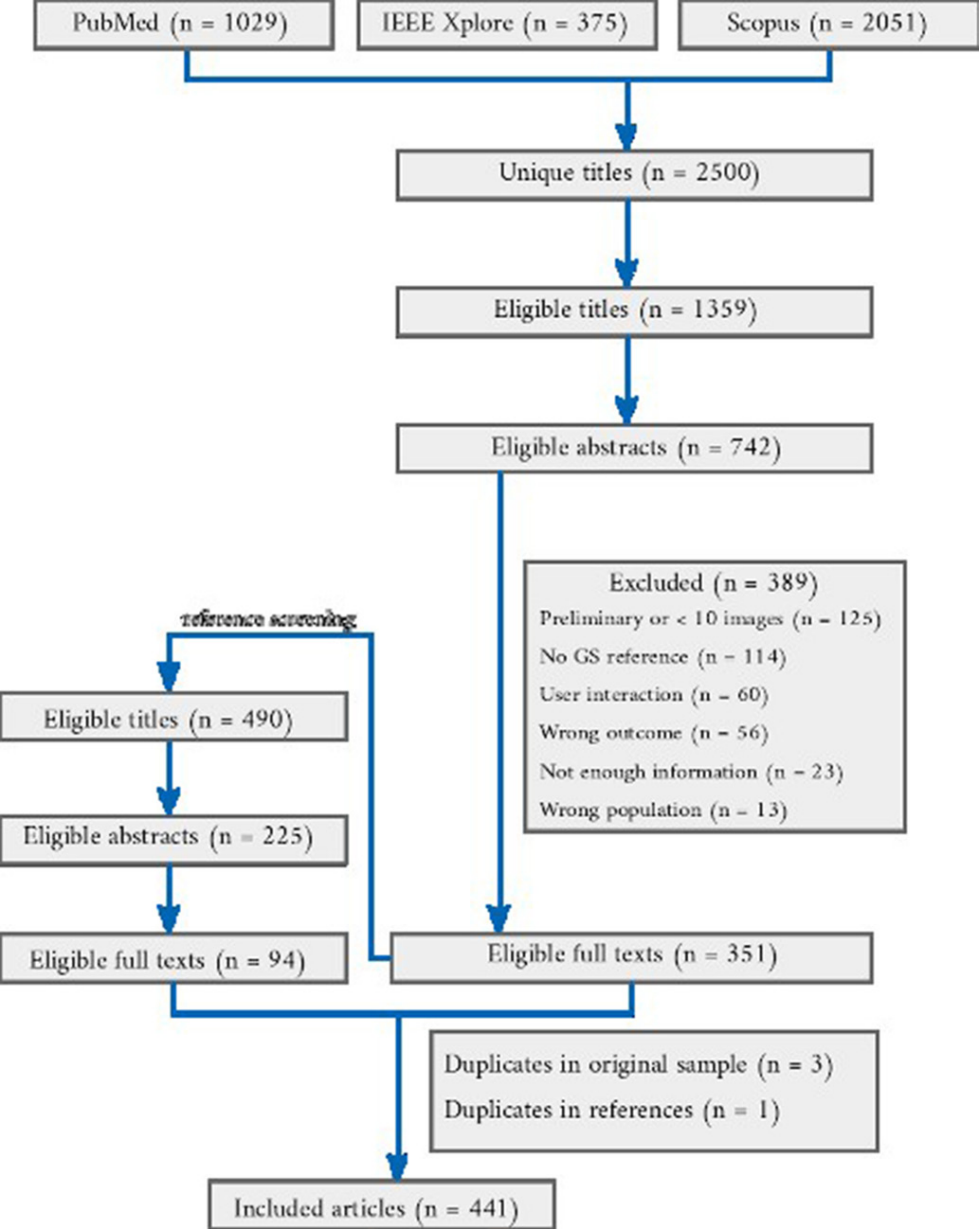
Flow chart of the article selection process from the result of querying three databases. For each stage, the number of articles selected is shown and numbers of excluded articles and reasons are given.

The number of papers included at each stage of the review is shown in figure 1. Among 2500 articles returned by the searches and 490 titles identified through reference screening, we included 441 articles in the review. Among the eligible papers, 255/441 were published in journals^23–277^ and 184/441 in conference proceedings or conference workshops,^278–460^ and 2/441 articles were uploaded as preprints.^461 462^ The following journals or conferences occurred most frequently as the publication source: International Conference on Medical Image Computing and Computer-Assisted Intervention (66 articles), *NeuroImage* (20 articles), *IEEE Transactions on Medical Imaging* (20 articles), *SPIE Medical Imaging Conference* (18 articles), *NeuroImage: Clinical* (15 articles), *IEEE International Symposium on Biomedical Imaging* (13 articles) and *PloS One* (12 articles).

**Figure 2:**
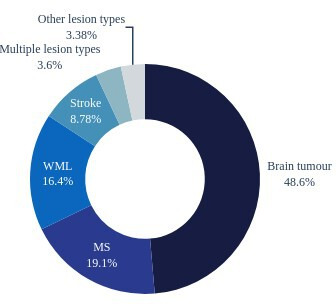
The number of algorithms described in the included articles developed for and validated on particular lesion type. The distinction is made between multiple sclerosis (MS) lesions and other white matter lesions (WML) following the similar distinction present in the reviewed articles and lesion segmentation challenges. ‘Multiple lesion types’ refers to algorithms that were evaluated on more than one type of lesion. ‘Other lesion types’ types of lesions included focal cortical dysplasia, metastasis, traumatic brain injury, cerebral palsy, abscess and necrosis.

Figure 2 shows the distribution of articles that propose a method to segment a particular brain lesion type. The dominance of methods segmenting brain tumours is evident from the data.

### Trends

**Figure 3:**
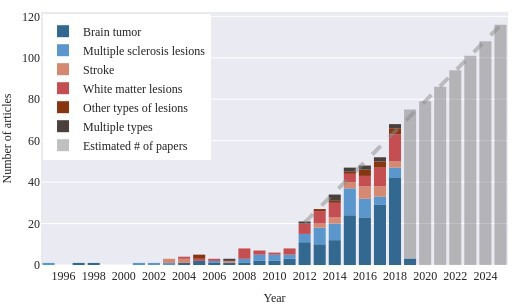
The number of articles published per year in the eligible sample with an indication of the number of articles that presented a segmentation method for a particular lesion type. We fit a linear function to the number of papers published each year from 2012 to estimate the development of the field in the upcoming years.

Substantial growth of the field was evident from the number of articles published annually (figure 3). Three distinct periods with an increase in the number of published articles compared with preceding years were noticeable. During these years, an increase is also evident in the number of articles addressing segmentation of lesion types corresponding to the challenges taking place. The first wave corresponds to the *3D Segmentation in the Clinic: A Grand Challenge II: MS Lesion Segmentation*.^463^ The second wave started in 2012 coinciding with the advent of *The Multimodal Brain Tumor Image Segmentation Benchmark (BraTS*).^464^ The third wave started in 2015 when three brain lesion segmentation challenges took place: BraTS, the *Longitudinal MS Lesion Segmentation Challenge*,^465^ and the *Ischemic Stroke Lesion Segmentation challenge (ISLES*).^466^

### Image sources and databases

The distribution of the image sources is presented in table 1. Most commonly, the image data were collected from non-public sources (254 articles). Publicly available data with reference segmentations were used in 217 articles. The most popular database was *BraTS*^464^ (157 articles) followed by the *3D Segmentation in the Clinic: A Grand Challenge II: MS Lesion Segmentation*^463^ and *ISLES* (17 articles).^466^ 10 articles rely on publicly available sources without reference segmentations. Forty-one articles validate the method on both publicly available data sets with reference segmentations and non-public data.

**Table 1:** Distribution of image sources used for algorithm validation

Non-public data sources	Publicly available data with reference segmentations				Publicly available data without reference segmentations
	BraTS^464^	Grand challenge II: MS lesion segmentation^463^	ISLES^466^	Other	
254	157	31	17	12	10

BraTS, Multimodal Brain Tumor Image Segmentation Benchmark; ISLES, Ischemic Stroke Lesion Segmentation challenge.

**Figure 4:**
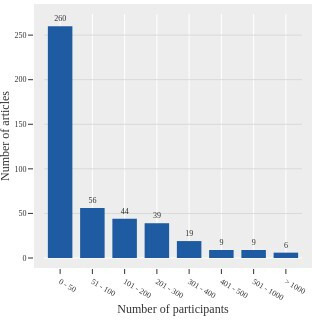
The number of patients (n) whose images were used to validate the methods in the eligible articles.

The distribution of the cohort sizes used to validate the proposed methods (figure 4) shows that more than half of the studies described in included articles test methods on 50 or fewer individuals’ brain images (260/441). Among the eligible articles, 280/441 describe a method validated on images obtained from more than one scanner.

### Sequences

The majority of the proposed methods operated on multisequence scans as inputs (307/441) while 6 accepted both single-sequence and multisequence input. Seventy-nine methods were built to evaluate single-sequence input and 22 did not specify the input modality. Articles that compared or evaluated a previously proposed method were not included in the analysis (27/441).

### Image processing steps, algorithms and computational theories

#### Preprocessing procedures

The most commonly applied preprocessing procedures and tools (disregarding publicly available databases for this purpose) are shown in table 2. Although authors commonly document individual steps and corresponding algorithms, information on whether these algorithms are integrated in the segmentation tool is rarely available.^90 195^ Fifteen articles mention visual supervision,^93 114 133^ semiautomatic preprocessing,^186 226 267 328 405^ manual correction,^155 171 209 273^ error or failure of preprocessing (usually at the brain extraction step).^135 136 160^

**Table 2:** Prevalent image preprocessing steps in 374/414 articles (not specified in 67 articles). FSL - FMRIB Software Library; BET - Brain Extraction Tool; SPM - Statistical Parametric Mapping.

Procedure	Common tool/approach	N (of 374)
Intensity normalisation	Histogram matching, intensity scaling	224
Bias field (field inhomogeneity) correction	N3, N4^476 477^	192
Brain extraction	FSL BET^478^	190
Image (co-)registration	rigid/affine; SPM, FSL^479 480^	179
Denoising	anisotropic diffusion filtering	60
None or minimal		5

Five methods proposed in the reviewed articles use minimal^268 400^ or no preprocessing,^163 164 220^ and in each case this is presented as an advantage of the proposed algorithm.

#### Algorithms and computational theories

**Figure 5:**
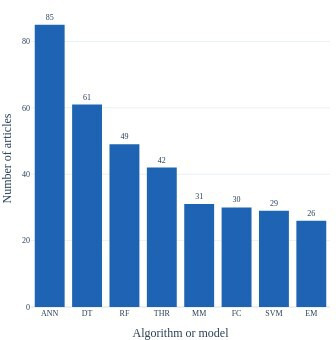
Distribution of the most common algorithms and models implemented in the lesion segmentation methods proposed by the articles eligible for the review. ANN - artificial neural networks; DT - decision trees; EM - expectation maximisation; FC - fuzzy clustering; MM - mixture models; RF - random fields; SVM - support vector machines; THR - thresholding.

Among the most commonly used algorithms or models in the segmentation methods were machine learning (decision trees, mixture models, fuzzy clustering, support vector machines, expectation maximisation) and in particular deep-learning algorithms (artificial neural networks) (figure 5). The analysis accounted for the same methods published in separate articles that deal with either different lesion types or similar lesion type with different image sources.

### Performance evaluation

Comparison with the reference by overlap measures was by far the most commonly used criterion to evaluate the accuracy of the automatic segmentation. Among articles that specify how the reference segmentations had been generated^265^ in publicly non-available data sets, most specified manual segmentation by one rater^85^ or two raters.^61^ More than two raters contributed in 38 articles, and 33 articles used semiautomatic procedures for constructing the reference. When multiple raters delineated the region of interest, the reference segmentation was obtained through consensus of the raters or majority voting. Forty-five articles have also included inter-rater and intrarater variability evaluation on data sets used in their studies. The most frequently applied measures were the Dice coefficient,^330^ sensitivity, also expressed as true positive ratio TPR, or overlap fraction,^227^ positive predictive value,^102^ Jaccard coefficient,^58^ and specificity.^82^ Volumetric measures, such as volume difference, error or correlation between the automatic and reference segmentation were used in 133 articles, and distance-based measures (such as Hausdorff distance, mean absolute distance, surface distance) in 41.

In addition to segmentation accuracy, reliability of a method was estimated in some of the articles. Reliability was directly evaluated through a test–retest procedure, where participants were scanned twice at a short interval, with repositioning between the scans in five articles.^127 177 196 321 347^ In other articles, indirect approaches were described where consistency of longitudinal data with clinical findings of stable or progressive disease was used as a proxy for reliability.^38 270 454^

### Performance comparison

Maldjian *et al*,^133^ Lesjak *et al*^150^ and de Sitter *et al*^224^ examined previously proposed methods and compared the results they obtained in their studies to the performance reported in the original papers. All three articles reported poorer independent testing results. The authors pointed out a lower lesion load of the studied population than in the original papers that accounted for reduced scores in the replication attempts. In their paper, de Sitter *et al*^224^ strongly called for improvements to automatic lesion segmentation before their introduction to routine clinical use.

Of 441 articles, 233 included information regarding both the processing time (from less than 1 s to 7 hours) and computational system requirements used for segmentation. In 56 articles, we found information about only one of the two parameters; however, such information is incomplete and cannot be used to estimate how well a method will perform on different hardware.

Only 24 of the included articles reported on methods that were made publicly available to download.^43 50 53 67 76 84 90–92 126 134 138 144 149 154 170 173 178 190 195 196 199 228 230 426^ One was available on request.^111^ Two articles promised future availability of the methods proposed^41 193^ but they were not available as of 4 May 2020.

### Reporting recommendations

Based on our findings, we developed a checklist with reporting recommendations (table 3).

**Table 3:** Proposed reporting items for automatic brain lesion segmentation studies

Technical validation	Method	List of processing steps necessary to apply to a raw image
		Computational system parameters
		Computation time
		Open documentation of the algorithm
	Reference segmentation	Number of raters
		Raters’ training/experience
		Method of segmentation
		Method of consolidation (if multiple raters)
	Validation	List of validation metrics used
		Number of images (split into training/validation/testing if applicable)
		Input sequences
		Number of scanners used to acquire images
		Acquisition parameters
		Number of time points and intervals for longitudinal data
	Results	Mean, median, SD for each validation metric
		Number of failed cases (if applicable)
Preclinical validation	Patient information	Diagnosis and level of verification for example, clinical follow-up, tissue sampling, autopsy, etc.
		Clinical presentation
		Administered treatments (if applicable)
	Clinical task	Explicit definition of the clinical task for which the algorithm is applied (eg, lesion growth estimation, treatment evaluation, radiotherapy planning).
	User validation	List of optimisation metrics used for the clinical task (if applicable).
		User’s method of evaluating the outcome.
Clinical validation	Information and storage system compatibility	Compatibility with picture archiving, communication, and storage systems
	Regulatory approval	Compliance with the regulatory approvals for software as a medical device.

## Discussion

In this paper, we present a scoping study of automatic brain lesion segmentation on MR images based on rigorous literature review methodology.^17^ This is the first review that takes all methods into account, independent of specialisation towards lesions of a particular aetiology. The key findings from our analysis of 441 articles are (1) a rapid increase of interest in the field, (2) a plethora of proposed methods contrasted with a dearth of open documentation and available software, and (3) high prevalence of problematic reporting practices that restrict the ability of independent researchers to replicate reported results and conduct method comparisons.

### Variable design of automatic segmentation methods

#### RQ1: Which common image processing steps are necessary for automatic brain lesion segmentation on MR images?

Image preprocessing emerged as a pervasive step in the processing chain for lesion segmentation. From the collected data we identified procedures that are commonly agreed to constitute preprocessing: skull stripping, image coregistration, bias field correction and intensity normalisation. Some algorithms rely on additional preprocessing, such as tissue classification. The procedures vary considerably between methods, and a lack of a universal distinction between segmentation steps and preprocessing steps became apparent during the analysis.

For a clinically suitable method, a distinction between preprocessing and segmentation steps may not be necessary since the final segmentation will rely on a whole processing chain (including the preprocessing) applied to a raw image. Validation of the whole chain, and assessing the impact of each step on the outcome, however, are desirable in method evaluation studies. Unfortunately, authors rarely state whether preprocessing is integrated with the segmentation algorithm and to what extent it relies on user input. Without such information, namely a list of all the steps that are performed on a raw image, the study cannot be replicated. This implies that the findings are of no help in assessing the potential clinical validity of the proposed method implementation.

Another implementation issue arises from the necessity of visual supervision or manual corrections of the preprocessing that was indicated in 15 articles. Any requirement of user interaction at any point in the processing chain entails disadvantages: it impedes implementation by increasing the complexity of the technical integration, and, once the tool is implemented, permanently burdens staff with an additional task. Any potential benefit of user interaction (eg, increased robustness) has to be weighed carefully against these costs.

#### RQ2: Which mathematical and computational theories are most commonly applied in which types of brain lesions?

The most prevalent methods used were artificial neural networks. The finding is not surprising given the popularity and remarkable performance of deep learning algorithms particularly in image processing applications. In the absence of widely used and agreed-upon criteria for performance evaluation, we have abstained from attempting to rank methods or make specific recommendations.

### Validation process and efficacy of automatic segmentation methods

#### RQ2: What is the efficacy of existing implementations?

We found that in view of current practices, the question cannot be answered: authors present assessments that are predicated on their own needs and biases, and there is no established standard that enables fair method comparison. Public challenges have been set up in an effort to address this problem, but they have only been partially successful, as illustrated in the following section. Three obstacles to fair comparative method assessments are paramount: the principal lack of ground truth in in vivo imaging, the enormous parameter space of acquisition settings that leads to variable feature presentations, and the lack of objectivity (as shown by inter-rater and intrarater variability^467 468^) of reference segmentations. More effort should thus be devoted to increasing the informativeness of the validation step. This can be achieved by painstakingly reporting the number of annotators, their experience, how the delineations were fused, and acquisition details that could conceivably have an impact. According to Gibson *et al,*^469^ such information can be used to calculate the statistical power of segmentation studies with respect to the number of reference images.

To answer the question we posed regarding the efficacy, we recorded the values of the comparison measures from each article. The information, however, cannot be meaningfully synthesised or compared and was not presented in the results section for this reason. Our investigation did not result in a direct answer to the question, but revealed a paucity of standard comparison procedures.

Inability to answer the posed question points to further issues on which we reflect from the perspective of clinical suitability of the proposed methods. The medical image analysis procedures commonly used in clinical diagnostics today are strongly operator dependent. They also scale poorly to the growing workload that results from the increasing number of modalities,^470^ the increasing accessibility of imaging scanners,^471^ and the increasing amount of image information due to technical advances that achieve enhanced spatial and contrast resolution.^472^ Automatic image analysis methods in general, and among them automatic lesion segmentation methods promise to alleviate some of this pressure. Other requisite characteristics that need to be assessed to evaluate the clinical applicability of a tool are reliability, robustness, and generalisability of its findings.

Reliability of an automatic segmentation algorithm seems to be assumed since the decision whether a given voxel is a lesion or not is made based on well-defined rules. However, the characteristic is rarely tested in method validation studies. Only five articles in our sample included a test–retest evaluation with patient repositioning between the scans. Such testing yields crucial reliability data against which longitudinal variations of lesion measures need to be compared with distinguish actual lesion change from other sources of variability.

Generalisability of a method is tested by processing images acquired from different scanners on a sufficient number of images from populations that are at least as heterogeneous as the population expected to be examined clinically. While more than half of the articles (280/441) used images coming from more than one scanner, validation on fewer than 50 images were equally prevalent (260/441). Such small sample sizes are insufficient for clinical validation, even if the images originate from multiple scanners. Using images acquired from multiple sources and validating a method on a large number of images reduces a method’s bias towards the data set or sets on which a given method was originally developed.

To us, one of the most surprising findings was how few methods are publicly available. Authors who share their software enable independent testing in unanticipated conditions and with various cohorts. Finally, to evaluate clinical relevance of the results of automatic segmentation, a method should be tested in genuine clinical scenarios for a well-defined task, such as monitoring disease progression, treatment response evaluation or radiotherapy planning. Each of these tasks may require a different level of error margin and validation standard and the relevance of the results must be evaluated by clinicians. Various metrics may be used to either optimise the method or evaluate its accuracy for a particular clinical question, lesion type and size.

Many papers claim usefulness of their algorithm for particular clinical tasks, but do not test the acceptability of the results for the intended purpose.

### Limitations and issues of the proposed methods, and grand challenges of the field

#### RQ4 (a) What are the limitations of those methods (…)?

Due to the inherent reference, image, and lesion variability, a meaningful comparison of algorithms evaluated independently on separate data sets to establish which method performs better is challenging. One solution to the problem has been proposed in the form of segmentation challenges. The challenges have become a quasi-standard for comparing brain lesion segmentation algorithms. The setup, however, comes with certain shortcomings with respect to evaluating clinical applicability of the evaluated algorithms. As mentioned, evaluating the whole chain of processing steps is especially important for validation of a clinical tool. In a challenge set-up, the algorithms are tested on partially preprocessed images. Even when the individual steps are described in detail, the impact of the preprocessing on the segmentation algorithms outcome is unknown. Validation of the processing chain becomes questionable if a step is changed, either based on the users’ subjective judgement or due to implementation of a different procedure for a particular step. According to *ISLES*^466^ and several articles in our sample, the skull-stripping step may need to be supervised and manually corrected. The choice of interpolation method is yet another issue that can influence the outcome of an automatic segmentation algorithm, especially in tasks requiring high accuracy.

Another aspect of the challenges is the prestige and publicity for the authors and their method after proposing a winning segmentation algorithm. The final rank is calculated by the organisers who evaluate the methods on hidden data sets. Organisers effectively take the role of independent arbiters. Participation in challenges and postcontest use of the challenge data as reference material indicate that organisers are generally trusted in this role. Maier-Hein *et al* suggest, however, that the results of such competitions should be considered carefully.^473^ The authors point to several issues that have a significant impact on the final ranking of evaluated methods. The first problem they report is lack of thorough reporting of relevant information that is essential for result interpretation. Moreover, it turns out that changes in metrics and aggregation methods for the scores of individual test cases alter the ranking of evaluated methods. A similar effect on the ranking was observed when the reference segmentations were exchanged against those of another rater. The final rank in such a competition depends also on the test data and on how missing data is handled.

These concerns cast doubt on the validity of testing clinical applicability of automatic brain lesion segmentation methods in a challenge setup. Even if the challenge databases contain images from multiple scanners, it cannot be regarded as generalisable if it has not been tested on raw images (reconstructed, but not otherwise processed) as produced by the scanning equipment. In our sample, only 10% of the articles explicitly report on such evaluation. While obtaining independent data sets is expensive, it is a crucial step on the way of creating a clinically applicable automatic lesion segmentation tool.

Ultimately, it is the target users who need to decide whether a given validation standard and resultant performance confidence are sufficient to apply a tool to answer a given clinical question. With guidance from support system developers, as well as transparent and thorough reporting of the processing steps and validation procedures, the users may consider a given tool trustworthy. Trustworthiness has been reported as a crucial factor in developing a clinically usable support tool.^474^ The present scoping review does not show what properties and features clinicians need to develop trust in a tool. Still, this is an important question that we will seek to address in future work.

We conducted an exploratory study to begin to address the issue.^475^ The aim was to learn how considering radiologists’ competent input can improve the design and validation procedures of automatic brain lesion segmentation methods with a view to increasing trust and trustworthiness. Our findings corroborate previous findings.^474^ We found that two crucial characteristics that influence clinicians’ trust in a tool are the provision of an error margin with any quantitative measure, and consideration of the varying need for accuracy, depending on the diagnostic task.

### Relevance for research and clinical practice

Noteworthy trends also emerged from the data regarding the prevalence of methods developed for segmentation of a particular lesion type, as well as the advent of segmentation challenges focussing on corresponding lesion types. We observed that the majority of the methods described in our sample have been developed for the purpose of brain tumour segmentation. We also note consistent growth of the number of images in the database underlying the *BraTS* challenge, which has been organised annually since 2012. Stroke lesion segmentation methods have not proliferated to the same extent, despite similar competitions having been organised (yearly *ISLES* challenges from 2015 to 2018). A distinct increase in the number of publications proposing white-matter lesion segmentation algorithms was observed when segmentation challenges of this type of lesion were organised.

Some compelling questions follow this observation, for example, whether some tasks are more difficult to solve algorithmically, or whether these trends reflect on clinical usefulness of developing segmentation algorithms for a particular lesion type. Even though the questions cannot be directly answered by our findings, they point to important issues that need to be explored to better understand the relevance of different lesion segmentation algorithms and implications of the trends for clinical practice.

Another finding that potentially contributes to the gap between research and clinical practice is the widespread use of deep-learning algorithms for the lesion segmentation task in the research setting. The practical efficiency of the algorithms depends on the availability of graphical processing units. Implementing a deep-learning based algorithm in a clinical setting requires dedicating resources for purchasing suitable hardware and integrating it with radiological workflow, information and storage systems.

A key factor in developing a clinical tool is obtaining regulatory approval for diagnostic use. None of the articles in our sample mentioned this requirement, possibly because authors do not consider it relevant at the stage of development when publication occurs.

It appears that despite claims of clinical relevance made by many authors, development of brain lesion segmentation methods happens predominantly in an academic space, where technological challenges matter most and implementation hurdles are not explicitly considered. To increase the relevance and societal benefit of method development in the field, it will be necessary for developers to widen their perspective to include the critical path towards clinical implementation, on which users’ demands and regulatory requirements have to be met.

### Challenges and limitations of the study

The biggest challenges we had to address in this study were designing data charting categories, extracting the relevant information regarding both inclusion and exclusion criteria, and the actual charting of the data. Arksey and O’Malley^17^ recommend deciding the inclusion criteria after becoming familiar with the identified relevant studies. For us, this was impossible due to the large size of the raw sample of original search results, along with the variability of both the type and the level of detail of reported information.

Our inclusion and exclusion criteria were defined based on a pilot analysis of articles selected randomly from the raw sample. These criteria were then applied to the full raw sample in hierarchical fashion. A major impediment at this stage was the inconsistent way the information is organised and presented in the articles. In the case of conference papers, the authors typically have to adhere to page limits, so certain compromises are inevitable. When it comes to journal publications, the methodology of a study should be thoroughly reported for the sake of reproducibility, but also to enable data extraction for systematic reviews.

Another challenge we faced and a limitation of the study comes from the mentioned incoherent way of reporting studies and a challenge to derive strict definitions of the inclusion and exclusion criteria. Often, the criteria we sought in the articles are not presented clearly, or the information is scarce. Keeping in mind the broad nature of a scoping review and the aim of mapping the field, the author conducting the scoping study (EG) chose to err on the side of inclusion if the information in an article did not allow a decisive application of the criteria. While this strategy, combined with a single-rater approach, introduces a certain amount of selection bias, this bias was at least consistent between articles. We believe the approach provides a reasonable tradeoff between transparency and reproducibility of the study, and fulfilling the objectives of conducting a scoping review.

Other sources of bias were excluded by design. In particular, we conducted the consultation interviews after the literature sampling step, eliminating potential selection bias arising from the interview results. While the strong dominance of articles treating tumour segmentation (cf. figure 2) may seem surprising, we believe that it is an accurate reflection of the research community’s interest.

This review does not consider modalities such as projection radiography, CT, nuclear imaging or ultrasonography. It focuses instead on MR, which is used to address a larger variety of brain lesions, provides more detailed information and stands out among radiological techniques regarding the number of related publications.

We acknowledge self-critically that, due to the extensive nature of the task, the time taken, and the lessons learnt during the work, some epistemic drift occurred, taking our focus away from the first two RQs and towards the question of clinical applicability that appears to be underserved by the current literature. Thanks to our early decision to hold ourselves to account by writing a detailed protocol,^22^ this drift stayed within reasonable boundaries and is well-documented, as we accounted for protocol modifications in the present work.

### Recommendations for future work

#### RQ4 (b) What issues should be addressed in future studies to develop a tool that is suitable for clinical use?

To address the shortcomings revealed by our analysis, we propose a set of recommendations for future studies as well as avenues researchers might follow that promise, in our estimation, to advance the field in the direction of enabling clinical decision support.

The most important recommendation arises from the variability encountered in many aspects of the field. We propose a checklist (table 3) of items that should be reported in investigations of automatic brain lesion segmentation methods. We split the items into three levels of validation that we see as a potential path towards developing a clinical tool. Technical validation studies focus on developing the algorithm and evaluating its performance according to common criteria in the field. The preclinical validation focuses on the performance of an algorithm in a setting resembling clinical environment and a realistic use case. The final level, clinical validation, requires the tool to be compatible with hospital and radiology information systems and to be ready for the process of obtaining regulatory approval for clinical use if such approval has not been yet obtained. This checklist, especially the technical validation level, will facilitate replication as well as comparisons between methods and studies, both informal and in meta-analyses. If authors were to follow the checklist in future studies, this would be a step towards standardisation of reporting in the interest of advancing knowledge and promoting implementation as clinical tools.

With similar priority, we ask researchers who have developed or are working on automatic brain lesion segmentation algorithms to publish software implementations. The benefits and challenges with fully automated versus interactive preprocessing should be assessed in terms of segmentation accuracy and reproducibility for a given, clinically relevant task.

Few articles in our sample evaluated their algorithm on an independent data set on top of the challenge one. We encourage authors to endeavour to test their method on images from other sites and sources. We also encourage collaboration between the authors and independent researchers who may have access to annotated test images. A preprocessing and segmentation method that has an acceptable and consistent performance on images acquired from various sources should finally be tested in clinical conditions. Therefore we strongly advocate close collaboration between researchers and authors of well-performing methods with clinicians. In such a scenario, the acceptability of the method’s performance to clinicians can be assessed in conjunction with its relevance for a given task. Moreover, an open dialogue between researchers and clinicians will help build an ABS system that meets the requirements for a clinically useful and usable tool.

Finally, efforts to define steps on the path towards designing and validating a clinically applicable ABS system should be made. We recommend consultation with stakeholders as a key element to verify the actual clinical needs and how to assess to what extent these needs are met by available research.

## Conclusions

This scoping study of automatic brain lesion segmentation on MR images shows a field growing at a rapid pace, an imbalance between proposed methods of which there are many and methods implemented for clinical application of which there are few, and a room for improvement of reporting practice with a view to enabling replication, method comparison and implementation. To promote this improvement, we propose a list of recommendations for future studies in the field. We identify knowledge gaps and potentially fruitful avenues for future research.

## Supplementary Material

Reviewer comments

Author's manuscript

## Data Availability

Data are available in a public, open access repository. DOI: 10.6084/m9.figshare.13651235

## References

[1] Therasse P, Arbuck SG, Eisenhauer EA, et al. New guidelines to evaluate the response to treatment in solid tumors. J Natl Cancer Inst 2000;92:205–16. 10.1093/jnci/92.3.205 10655437

[2] Miller AB, Hoogstraten B, Staquet M, et al. Reporting results of cancer treatment. Cancer 1981;47:207–14. 10.1002/1097-0142(19810101)47:1&lt;207::AID-CNCR2820470134&gt;3.0.CO;2-6 7459811

[3] Fazekas F, Chawluk JB, Alavi A, et al. MR signal abnormalities at 1.5 T in Alzheimer’s dementia and normal aging. AJR Am J Roentgenol 1987;149:351–6. 10.2214/ajr.149.2.351 3496763

[4] Ellingson BM, Wen PY, Cloughesy TF. Modified criteria for radiographic response assessment in glioblastoma clinical trials. Neurotherapeutics 2017;14:307–20. 10.1007/s13311-016-0507-6 28108885 PMC5398984

[5] Rosenkrantz AB, Hanna TN, Babb JS, et al. Changes in emergency department imaging: perspectives from national patient surveys over two decades. J Am Coll Radiol 2017;14:1282–90. 10.1016/j.jacr.2017.03.004 28483547

[6] Smith-Bindman R, Miglioretti DL, Larson EB. Rising use of diagnostic medical imaging in a large integrated health system. Health Aff 2008;27:1491–502. 10.1377/hlthaff.27.6.1491 PMC2765780 18997204

[7] Bauer S, Wiest R, Nolte L-P, et al. A survey of MRI-based medical image analysis for brain tumor studies. Phys Med Biol 2013;58:R97–129. 10.1088/0031-9155/58/13/R97 23743802

[8] Debette S, Markus HS. The clinical importance of white matter hyperintensities on brain magnetic resonance imaging: systematic review and meta-analysis. BMJ 2010;341:c3666. 10.1136/bmj.c3666 20660506 PMC2910261

[9] Deepa SA. Review of brain tumor detection from MRI images. In: 2016 3rd International Conference on Computing for Sustainable Global Development (INDIACom) 2016.

[10] García-Lorenzo D, Francis S, Narayanan S, et al. Review of automatic segmentation methods of multiple sclerosis white matter lesions on conventional magnetic resonance imaging. Med Image Anal 2013;17:1–18. 10.1016/j.media.2012.09.004 23084503

[11] Işın A, Direkoğlu C, Şah M. Review of MRI-based brain tumor image segmentation using deep learning methods. Procedia Computer Science 2016;102:317–24.

[12] Ito KL, Kim H, Liew S-L. A comparison of automated lesion segmentation approaches for chronic stroke T1-weighted MRI data. Hum Brain Mapp 2019;40:4669–85. 10.1002/hbm.24729 31350795 PMC6851560

[13] Kumari N, Saxena S. Review of brain tumor segmentation and classification. In: 2018 International Conference on Current Trends towards Converging Technologies (ICCTCT) 2018:1–6.

[14] Mortazavi D, Kouzani AZ, Soltanian-Zadeh H. Segmentation of multiple sclerosis lesions in MR images: a review. Neuroradiology 2012;54:299–320. 10.1007/s00234-011-0886-7 21584674

[15] Roy S, Nag S, Maitra IK. A review on automated brain tumor detection and segmentation from MRI of brain. Available: http://arxiv.org/abs/1312.6150 [Accessed 23 Nov 2020].

[16] Saritha S, Amutha Prabha N, Prabha NA. A comprehensive review: segmentation of MRI images-brain tumor. Int J Imaging Syst Technol 2016;26:295–304. 10.1002/ima.22201

[17] Arksey H, O’Malley L. Scoping studies: towards a methodological framework. Int J Soc Res Methodol 2005;8:19–32. 10.1080/1364557032000119616

[18] Levac D, Colquhoun H, O’Brien KK. Scoping studies: advancing the methodology. Implement Sci 2010;5:69. 10.1186/1748-5908-5-69 20854677 PMC2954944

[19] Colquhoun HL, Levac D, O’Brien KK, et al. Scoping reviews: time for clarity in definition, methods, and reporting. J Clin Epidemiol 2014;67:1291–4. 10.1016/j.jclinepi.2014.03.013 25034198

[20] Moher D, Liberati A, Tetzlaff J, et al. Preferred reporting items for systematic reviews and meta-analyses: the PRISMA statement. PLoS Med 2009;6:e1000097. 10.1371/journal.pmed.1000097 19621072 PMC2707599

[21] Tricco AC, Lillie E, Zarin W, et al. PRISMA extension for scoping reviews (PRISMA-ScR): checklist and explanation. Ann Intern Med 2018;169:467–73. 10.7326/M18-0850 30178033

[22] Gryska EA, Schneiderman J, Heckemann RA. Automatic brain lesion segmentation on standard MRIs of the human head: a scoping review protocol. BMJ Open 2019;9:e024824. 10.1136/bmjopen-2018-024824 PMC6398796 30765406

[23] Zijdenbos AP, Forghani R, Evans AC. Automatic “pipeline” analysis of 3-D MRI data for clinical trials: application to multiple sclerosis. IEEE Trans Med Imaging 2002;21:1280–91. 10.1109/TMI.2002.806283 12585710

[24] Zhuge Y, Krauze AV, Ning H, et al. Brain tumor segmentation using holistically nested neural networks in MRI images. Med Phys 2017;44:5234–43. 10.1002/mp.12481 28736864 PMC5646222

[25] Zhong Y, Utriainen D, Wang Y, et al. Automated white matter hyperintensity detection in multiple sclerosis using 3D T2 FLAIR. Int J Biomed Imaging 2014;2014:e239123 10.1155/2014/239123 25136355 PMC4130152

[26] Zhao Z, Yang G, Lin Y, et al. Automated glioma detection and segmentation using graphical models. PLoS One 2018;13:e0200745. 10.1371/journal.pone.0200745 30130371 PMC6103499

[27] Zhao X, Wu Y, Song G, et al. A deep learning model integrating FCNNs and CRFs for brain tumor segmentation. Med Image Anal 2018;43:98–111. 10.1016/j.media.2017.10.002 29040911 PMC6029627

[28] Zhao L, Jia K. Multiscale CNNs for brain tumor segmentation and diagnosis. Comput Math Methods Med 2016;2016:e8356294 10.1155/2016/8356294 PMC4812495 27069501

[29] Zhang R, Zhao L, Lou W, et al. Automatic segmentation of acute ischemic stroke from DWI using 3-D fully Convolutional DenseNets. IEEE Trans Med Imaging 2018;37:2149–60. 10.1109/TMI.2018.2821244 29994088

[30] Zhang N, Ruan S, Lebonvallet S, et al. Kernel feature selection to fuse multi-spectral MRI images for brain tumor segmentation. Comput Vision Image Understand 2011;115:256–69. 10.1016/j.cviu.2010.09.007

[31] Zhang J, Barboriak DP, Hobbs H, et al. A fully automatic extraction of magnetic resonance image features in glioblastoma patients. Med Phys 2014;41:042301. 10.1118/1.4866218 24694151

[32] Zhan T, Chen Y, Hong X, et al. Brain tumor segmentation using deep belief networks and pathological knowledge. CNS Neurol Disord Drug Targets 2017;16:129–36. 10.2174/1871527316666170113101559 28088902

[33] Zhan T, Shen F, Hong X, et al. A glioma segmentation method using CoTraining and superpixel-based spatial and clinical constraints. IEEE Access 2018;6:57113–22. 10.1109/ACCESS.2018.2873674

[34] Zacharaki EI, Bezerianos A. Abnormality segmentation in brain images via distributed estimation. IEEE Trans Inf Technol Biomed 2012;16:330–8. 10.1109/TITB.2011.2178422 22157062

[35] Yu R, Xiao L, Wei Z. Coarse classification to Region-Scalable refining for white matter lesions segmentation in multi-channel MRI. CNS Neurol Disord Drug Targets 2017;16:150–9. 10.2174/1871527315666161220145247 28000558

[36] Yoo BI, Lee JJ, Han JW, et al. Application of variable threshold intensity to segmentation for white matter hyperintensities in fluid attenuated inversion recovery magnetic resonance images. Neuroradiology 2014;56:265–81. 10.1007/s00234-014-1322-6 24493377

[37] Wu W, Chen AYC, Zhao L, et al. Brain tumor detection and segmentation in a CRF (conditional random fields) framework with pixel-pairwise affinity and superpixel-level features. Int J Comput Assist Radiol Surg 2014;9:241–53. 10.1007/s11548-013-0922-7 23860630

[38] Weizman L, Ben Sira L, Joskowicz L, et al. Automatic segmentation, internal classification, and follow-up of optic pathway gliomas in MRI. Med Image Anal 2012;16:177–88. 10.1016/j.media.2011.07.001 21852179

[39] Wang Y, Zhou Y, Wang H, et al. Voxel-based automated detection of focal cortical dysplasia lesions using diffusion tensor imaging and T2-weighted MRI data. Epilepsy & Behavior 2018;84:127–34. 10.1016/j.yebeh.2018.04.005 29793134

[40] Wang Y, Catindig JA, Hilal S, et al. Multi-stage segmentation of white matter hyperintensity, cortical and lacunar infarcts. Neuroimage 2012;60:2379–88. 10.1016/j.neuroimage.2012.02.034 22387175

[41] Wang R, Li C, Wang J, et al. Automatic segmentation and volumetric quantification of white matter hyperintensities on fluid-attenuated inversion recovery images using the extreme value distribution. Neuroradiology 2015;57:307–20. 10.1007/s00234-014-1466-4 25407717

[42] Wang R, Li C, Wang J, et al. Automatic segmentation of white matter lesions on magnetic resonance images of the brain by using an outlier detection strategy. Magn Reson Imaging 2014;32:1321–9. 10.1016/j.mri.2014.08.010 25131627

[43] Wang R, Li C, Wang J, et al. Automatic segmentation and quantitative analysis of white matter hyperintensities on FLAIR images using Trimmed-Likelihood estimator. Acad Radiol 2014;21:1512–23. 10.1016/j.acra.2014.07.001 25176451

[44] Wang H, Yushkevich PA. Multi-atlas segmentation without registration: a supervoxel-based approach. Med Image Comput Comput Assist Interv 2013;16:535–42. 24505803 10.1007/978-3-642-40760-4_67 PMC3918684

[45] Vishnuvarthanan A, Rajasekaran MP, Govindaraj V, et al. Development of a combinational framework to concurrently perform tissue segmentation and tumor identification in T1 - W, T2 - W, FLAIR and MPR type magnetic resonance brain images. Expert Syst Appl 2018;95:280–311. 10.1016/j.eswa.2017.11.040

[46] Vijayakumar C, Damayanti G, Pant R, et al. Segmentation and grading of brain tumors on apparent diffusion coefficient images using self-organizing maps. Comput Med Imaging Graph 2007;31:473–84. 10.1016/j.compmedimag.2007.04.004 17572068

[47] Vidyaratne L, Alam M, Shboul Z. Deep learning and Texture-Based semantic label fusion for brain tumor segmentation. Proc SPIE Int Soc Opt Eng 2018;2018. 10.1117/12.2292930 PMC5851487 29551853

[48] Verma R, Zacharaki EI, Ou Y, et al. Multiparametric tissue characterization of brain neoplasms and their recurrence using pattern classification of MR images. Acad Radiol 2008;15:966–77. 10.1016/j.acra.2008.01.029 18620117 PMC2596598

[49] Van Leemput K, Maes F, Vandermeulen D, et al. Automated segmentation of multiple sclerosis lesions by model outlier detection. IEEE Trans Med Imaging 2001;20:677–88. 10.1109/42.938237 11513020

[50] Valverde S, Cabezas M, Roura E. Improving automated multiple sclerosis lesion segmentation with a cascaded 3D convolutional neural network approach. NeuroImage Published Online First 2017. 10.1016/j.neuroimage.2017.04.034 28435096

[51] Valcarcel AM, Linn KA, Vandekar SN, et al. Mimosa: an automated method for Intermodal segmentation analysis of multiple sclerosis brain lesions. J Neuroimaging 2018;28:389–98. 10.1111/jon.12506 29516669 PMC6030441

[52] Usman K, Rajpoot K. Brain tumor classification from multi-modality MRI using wavelets and machine learning. Pattern Anal Applic 2017;20:871–81. 10.1007/s10044-017-0597-8

[53] Tustison NJ, Shrinidhi KL, Wintermark M, et al. Optimal symmetric multimodal templates and concatenated random forests for supervised brain tumor segmentation (simplified) with ANTsR. Neuroinformatics 2015;13:209–25. 10.1007/s12021-014-9245-2 25433513

[54] Tsai J-Z, Peng S-J, Chen Y-W, et al. Automated segmentation and quantification of white matter hyperintensities in acute ischemic stroke patients with cerebral infarction. PLoS One 2014;9:e104011. 10.1371/journal.pone.0104011 25127120 PMC4134193

[55] Tsai J-Z, Peng S-J, Chen Y-W, et al. Automatic detection and quantification of acute cerebral infarct by fuzzy clustering and histographic characterization on diffusion weighted MR imaging and apparent diffusion coefficient MAP. Biomed Res Int 2014;2014:e963032 10.1155/2014/963032 PMC3971548 24738080

[56] Tong J, Zhao Y, Zhang P, et al. MRI brain tumor segmentation based on texture features and kernel sparse coding. Biomed Signal Process Control 2019;47:387–92. 10.1016/j.bspc.2018.06.001

[57] Tong J-jun, Zhang P, Weng Y-xiang, et al. Kernel sparse representation for MRI image analysis in automatic brain tumor segmentation. Frontiers Inf Technol Electronic Eng 2018;19:471–80. 10.1631/FITEE.1620342

[58] Tomas-Fernandez X, Warfield SK. A model of population and subject (mops) intensities with application to multiple sclerosis lesion segmentation. IEEE Trans Med Imaging 2015;34:1349–61. 10.1109/TMI.2015.2393853 25616008 PMC4506921

[59] Thomas T, AR S. A novel automatic method for extraction of glioma tumor, white matter and grey matter from brain magnetic resonant images. Biomed Imag Intervent J 2013;9.

[60] Thiruvenkadam K, Perumal N. Fully automatic method for segmentation of brain tumor from multimodal magnetic resonance images using wavelet transformation and clustering technique. Int J Imaging Syst Technol 2016;26:305–14. 10.1002/ima.22202

[61] Szwarc P, Kawa J, Rudzki M, et al. Automatic brain tumour detection and neovasculature assessment with multiseries MRI analysis. Comput Med Imaging Graph 2015;46:178–90. 10.1016/j.compmedimag.2015.06.002 26183648

[62] Sweeney EM, Vogelstein JT, Cuzzocreo JL, et al. A comparison of supervised machine learning algorithms and feature vectors for MS lesion segmentation using multimodal structural MRI. PLoS One 2014;9:e95753. 10.1371/journal.pone.0095753 24781953 PMC4004572

[63] Sweeney EM, Shinohara RT, Shiee N, et al. OASIS is automated statistical inference for segmentation, with applications to multiple sclerosis lesion segmentation in MRI. Neuroimage 2013;2:402–13. 10.1016/j.nicl.2013.03.002 24179794 PMC3777691

[64] Sweeney EM, Shinohara RT, Shea CD, et al. Automatic lesion incidence estimation and detection in multiple sclerosis using Multisequence longitudinal MRI. AJNR Am J Neuroradiol 2013;34:68–73. 10.3174/ajnr.A3172 22766673 PMC3554794

[65] Sudre CH, Cardoso MJ, Bouvy WH, et al. Bayesian model selection for pathological neuroimaging data applied to white matter lesion segmentation. IEEE Trans Med Imaging 2015;34:2079–102. 10.1109/TMI.2015.2419072 25850086

[66] Strumia M, Schmidt FR, Anastasopoulos C, et al. White matter MS-Lesion segmentation using a geometric brain model. IEEE Trans Med Imaging 2016;35:1636–46. 10.1109/TMI.2016.2522178 26829786

[67] Stone JR, Wilde EA, Taylor BA, et al. Supervised learning technique for the automated identification of white matter hyperintensities in traumatic brain injury. Brain Injury 2016;30:1458–68. 10.1080/02699052.2016.1222080 27834541

[68] Steenwijk MD, Pouwels PJW, Daams M, et al. Accurate white matter lesion segmentation by K nearest neighbor classification with tissue type priors (kNN-TTPs). Neuroimage 2013;3:462–9. 10.1016/j.nicl.2013.10.003 24273728 PMC3830067

[69] Steed TC, Treiber JM, Patel KS, et al. Iterative probabilistic voxel labeling: automated segmentation for analysis of the cancer imaging Archive glioblastoma images. AJNR Am J Neuroradiol 2015;36:678–85. 10.3174/ajnr.A4171 25414001 PMC7964326

[70] Soltaninejad M, Yang G, Lambrou T, et al. Supervised learning based multimodal MRI brain tumour segmentation using texture features from supervoxels. Comp Method Prog Biomed 2018;157:69–84. 10.1016/j.cmpb.2018.01.003 29477436

[71] Soltaninejad M, Yang G, Lambrou T, et al. Automated brain tumour detection and segmentation using superpixel-based extremely randomized trees in FLAIR MRI. Int J CARS 2017;12:183–203. 10.1007/s11548-016-1483-3 PMC5263212 27651330

[72] Smart SD, Firbank MJ, O’Brien JT. Validation of automated white matter hyperintensity segmentation. J Aging Res 2011;2011:e391783 10.4061/2011/391783 PMC3167190 21904678

[73] Sivakumar P, Ganeshkumar P. An efficient automated methodology for detecting and segmenting the ischemic stroke in brain MRI images. Int J Imaging Syst Technol 2017;27:265–72. 10.1002/ima.22231

[74] Simões R, Mönninghoff C, Dlugaj M, et al. Automatic segmentation of cerebral white matter hyperintensities using only 3D FLAIR images. Magn Reson Imaging 2013;31:1182–9. 10.1016/j.mri.2012.12.004 23684961

[75] Shimol EB, Joskowicz L, Eliahou R, et al. Computer-based radiological longitudinal evaluation of meningiomas following stereotactic radiosurgery. Int J CARS 2018;13:215–28. 10.1007/s11548-017-1673-7 29032421

[76] Shiee N, Bazin P-L, Ozturk A, et al. A topology-preserving approach to the segmentation of brain images with multiple sclerosis lesions. Neuroimage 2010;49:1524–35. 10.1016/j.neuroimage.2009.09.005 19766196 PMC2806481

[77] Shi L, Wang D, Liu S, et al. Automated quantification of white matter lesion in magnetic resonance imaging of patients with acute infarction. J Neurosci Methods 2013;213:138–46. 10.1016/j.jneumeth.2012.12.014 23261771

[78] Selvathi D, Selvaraj H, Selvi ST. Hybrid approach for brain tumor segmentation in magnetic resonance images using cellular neural networks and optimization techniques. Int J Comput Intell Appl 2010;09:17–31. 10.1142/S1469026810002781

[79] Scully M, Anderson B, Lane T, et al. An automated method for segmenting white matter lesions through multi-level morphometric feature classification with application to lupus. Front Hum Neurosci 2010;4:27. 10.3389/fnhum.2010.00027 20428508 PMC2859868

[80] Schmidt P, Gaser C, Arsic M, et al. An automated tool for detection of FLAIR-hyperintense white-matter lesions in multiple sclerosis. Neuroimage 2012;59:3774–83. 10.1016/j.neuroimage.2011.11.032 22119648

[81] Sasikanth S, Suresh Kumar S, Kumar SS. Glioma tumor detection in brain MRI image using ANFIS-based normalized graph cut approach. Int J Imaging Syst Technol 2018;28:64–71. 10.1002/ima.22257

[82] Sanjuán A, Price CJ, Mancini L, et al. Automated identification of brain tumors from single MR images based on segmentation with refined patient-specific priors. Front Neurosci 2013;7. 10.3389/fnins.2013.00241 PMC3865426 24381535

[83] Samaille T, Fillon L, Cuingnet R, et al. Contrast-based fully automatic segmentation of white matter hyperintensities: method and validation. PLoS One 2012;7:e48953. 10.1371/journal.pone.0048953 23152828 PMC3495958

[84] Salem M, Cabezas M, Valverde S, et al. A supervised framework with intensity subtraction and deformation field features for the detection of new T2-w lesions in multiple sclerosis. Neuroimage 2018;17:607–15. 10.1016/j.nicl.2017.11.015 29234597 PMC5716954

[85] Norhashimah SM. Fully automated region growing segmentation of brain lesion in diffusion-weighted MRI. IAENG Int J Comp Sci 2012;39:10.

[86] Rundo L, Militello C, Tangherloni A, et al. Next for neuro-radiosurgery: a fully automatic approach for necrosis extraction in brain tumor MRI using an unsupervised machine learning technique. Int J Imaging Syst Technol 2018;28:21–37. 10.1002/ima.22253

[87] Roy S, He Q, Sweeney E, et al. Subject-specific sparse dictionary learning for atlas-based brain MRI segmentation. IEEE J Biomed Health Inform 2015;19:1598–609. 10.1109/JBHI.2015.2439242 26340685 PMC4562064

[88] Roy S, He Q, Carass A. Example based lesion segmentation. Proc SPIE Int Soc Opt Eng 2014;9034. 10.1117/12.2043917 PMC5082981 27795605

[89] Roy PK, Bhuiyan A, Janke A, et al. Automatic white matter lesion segmentation using contrast enhanced FLAIR intensity and Markov random field. Comput Med Imaging Graph 2015;45:102–11. 10.1016/j.compmedimag.2015.08.005 26398564

[90] Roura E, Sarbu N, Oliver A, et al. Automated detection of lupus white matter lesions in MRI. Front Neuroinform 2016;10. 10.3389/fninf.2016.00033 PMC4981618 27570507

[91] Roura E, Oliver A, Cabezas M, et al. A toolbox for multiple sclerosis lesion segmentation. Neuroradiology 2015;57:1031–43. 10.1007/s00234-015-1552-2 26227167

[92] Rios Velazquez E, Meier R, Dunn Jr WD, et al. Fully automatic GBM segmentation in the TCGA-GBM dataset: prognosis and correlation with VASARI features. Sci Rep 2015;5. 10.1038/srep16822 PMC4649540 26576732

[93] Rincón M, Díaz-López E, Selnes P, et al. Improved automatic segmentation of white matter hyperintensities in MRI based on multilevel lesion features. Neuroinformatics 2017;15:231–45. 10.1007/s12021-017-9328-y 28378263

[94] Reza S, Iftekharuddin K. Multi-class abnormal brain tissue segmentation using texture features. Proc NCI MICCAI-BRATS 2013;2013:38–42.

[95] Razzak MI, Imran M, Xu G. Efficient brain tumor segmentation with multiscale Two-Pathway-Group conventional neural networks. IEEE J Biomed Health Inform 2019;23:1911–9. 10.1109/JBHI.2018.2874033 30295634

[96] Raju AR, Suresh P, Rao RR. Bayesian HCS-based multi-SVNN: a classification approach for brain tumor segmentation and classification using Bayesian fuzzy clustering. Biocybern Biomed Eng 2018;38:646–60. 10.1016/j.bbe.2018.05.001

[97] Rajinikanth V, Satapathy SC, Fernandes SL, et al. Entropy based segmentation of tumor from brain Mr images – a study with teaching learning based optimization. Pattern Recognit Lett 2017;94:87–95. 10.1016/j.patrec.2017.05.028

[98] Rachmadi MF, Valdés-Hernández MDC, Agan MLF, et al. Segmentation of white matter hyperintensities using convolutional neural networks with global spatial information in routine clinical brain MRI with none or mild vascular pathology. Comput Med Imaging Graph 2018;66:28–43. 10.1016/j.compmedimag.2018.02.002 29523002

[99] Qu X, Yang J, Ma S, et al. Positive Unanimous voting algorithm for focal cortical dysplasia detection on magnetic resonance image. Front Comput Neurosci 2016;10. 10.3389/fncom.2016.00025 PMC4825107 27092069

[100] Qin C, Guerrero R, Bowles C, et al. A large margin algorithm for automated segmentation of white matter hyperintensity. Pattern Recognit 2018;77:150–9. 10.1016/j.patcog.2017.12.016

[101] Pustina D, Coslett HB, Turkeltaub PE, et al. Automated segmentation of chronic stroke lesions using LINDA: lesion identification with neighborhood data analysis. Hum Brain Mapp 2016;37:1405–21. 10.1002/hbm.23110 26756101 PMC4783237

[102] Praveen GB, Agrawal A, Sundaram P, et al. Ischemic stroke lesion segmentation using stacked sparse autoencoder. Comput Biol Med 2018;99:38–52. 10.1016/j.compbiomed.2018.05.027 29883752

[103] Bhanu Prakash KN, Gupta V, Bilello M, et al. Identification, segmentation, and image property study of acute infarcts in diffusion-weighted images by using a probabilistic neural network and adaptive Gaussian mixture model. Acad Radiol 2006;13:1474–84. 10.1016/j.acra.2006.09.045 17138115

[104] Porz N, Habegger S, Meier R, et al. Fully automated enhanced tumor compartmentalization: man vs. machine reloaded. PLoS One 2016;11:e0165302. 10.1371/journal.pone.0165302 27806121 PMC5091868

[105] Porz N, Bauer S, Pica A, et al. Multi-Modal glioblastoma segmentation: man versus machine. PLoS One 2014;9:e96873. 10.1371/journal.pone.0096873 24804720 PMC4013039

[106] Popuri K, Cobzas D, Murtha A, et al. 3D variational brain tumor segmentation using Dirichlet priors on a clustered feature set. Int J Comput Assist Radiol Surg 2012;7:493–506. 10.1007/s11548-011-0649-2 21833491

[107] Pinto A, Pereira S, Rasteiro D, et al. Hierarchical brain tumour segmentation using extremely randomized trees. Pattern Recognit 2018;82:105–17. 10.1016/j.patcog.2018.05.006

[108] Pereira S, Pinto A, Alves V, et al. Brain tumor segmentation using Convolutional neural networks in MRI images. IEEE Trans Med Imaging 2016;35:1240–51. 10.1109/TMI.2016.2538465 26960222

[109] Parisot S, Wells W, Chemouny S, et al. Concurrent tumor segmentation and registration with uncertainty-based sparse non-uniform graphs. Med Image Anal 2014;18:647–59. 10.1016/j.media.2014.02.006 24717540 PMC4068266

[110] Pagnozzi AM, Dowson N, Doecke J, et al. Automated, quantitative measures of grey and white matter lesion burden correlates with motor and cognitive function in children with unilateral cerebral palsy. Neuroimage 2016;11:751–9. 10.1016/j.nicl.2016.05.018 27330975 PMC4908311

[111] Ozenne B, Subtil F, Østergaard L, et al. Spatially regularized mixture model for lesion segmentation with application to stroke patients. Biostatistics 2015;16:580–95. 10.1093/biostatistics/kxv004 25745872

[112] Ong KH, Ramachandram D, Mandava R, et al. Automatic white matter lesion segmentation using an adaptive outlier detection method. Magn Reson Imaging 2012;30:807–23. 10.1016/j.mri.2012.01.007 22578927

[113] Njeh I, Sallemi L, Ayed IB, et al. 3D multimodal MRI brain glioma tumor and edema segmentation: a graph cut distribution matching approach. Comput Med Imaging Graph 2015;40:108–19. 10.1016/j.compmedimag.2014.10.009 25467804

[114] Nie J, Xue Z, Liu T, et al. Automated brain tumor segmentation using spatial accuracy-weighted hidden Markov random field. Comput Med Imaging Graph 2009;33:431–41. 10.1016/j.compmedimag.2009.04.006 19446435 PMC2739047

[115] Nagenthiraja K, Walcott BP, Hansen MB, et al. Automated Decision-Support system for prediction of treatment responders in acute ischemic stroke. Front Neurol 2013;4. 10.3389/fneur.2013.00140 PMC3783931 24133479

[116] Naceur MB, Saouli R, Akil M, et al. Fully automatic brain tumor segmentation using end-to-end incremental deep neural networks in MRI images. Comput Methods Programs Biomed 2018;166:39–49. 10.1016/j.cmpb.2018.09.007 30415717

[117] Nabizadeh N, Kubat M. Brain tumors detection and segmentation in Mr images: Gabor wavelet vs. statistical features. Comput Elect Eng 2015;45:286–301. 10.1016/j.compeleceng.2015.02.007

[118] Murphy K, van der Aa NE, Negro S, et al. Automatic quantification of ischemic injury on diffusion-weighted MRI of neonatal hypoxic ischemic encephalopathy. Neuroimage 2017;14:222–32. 10.1016/j.nicl.2017.01.005 28180081 PMC5288491

[119] Muda AF, Mohd Saad N, Abu Bakar SAR. Brain lesion segmentation using fuzzy C-means on diffusion-weighted imaging. ARPN J Eng Appl Sci 2015;10:1138–44.

[120] Mouridsen K, Nagenthiraja K, Jónsdóttir Kristjana Ýr, et al. Acute stroke: automatic perfusion lesion outlining using level sets. Radiology 2013;269:404–12. 10.1148/radiol.13121622 23687176

[121] Moeskops P, de Bresser J, Kuijf HJ, et al. Evaluation of a deep learning approach for the segmentation of brain tissues and white matter hyperintensities of presumed vascular origin in MRI. Neuroimage 2018;17:251–62. 10.1016/j.nicl.2017.10.007 29159042 PMC5683197

[122] Mitra S, Banerjee S, Hayashi Y. Volumetric brain tumour detection from MRI using visual saliency. PLoS One 2017;12:e0187209. 10.1371/journal.pone.0187209 29095877 PMC5667735

[123] Mitra J, Bourgeat P, Fripp J, et al. Lesion segmentation from multimodal MRI using random forest following ischemic stroke. Neuroimage 2014;98:324–35. 10.1016/j.neuroimage.2014.04.056 24793830

[124] Menze BH, Van Leemput K, Lashkari D, et al. A generative probabilistic model and discriminative extensions for brain lesion segmentation – with application to tumor and stroke. IEEE Trans Med Imaging 2016;35:933–46. 10.1109/TMI.2015.2502596 26599702 PMC4854961

[125] Meier R, Porz N, Knecht U, et al. Automatic estimation of extent of resection and residual tumor volume of patients with glioblastoma. J Neurosurg 2017;127:798–806. 10.3171/2016.9.JNS16146 28059651

[126] Meier R, Knecht U, Loosli T, et al. Clinical evaluation of a Fully-automatic segmentation method for longitudinal brain tumor volumetry. Sci Rep 2016;6:23376. 10.1038/srep23376 27001047 PMC4802217

[127] Meier DS, Guttmann CRG, Tummala S, et al. Dual-sensitivity multiple sclerosis lesion and CSF segmentation for multichannel 3T brain MRI. J Neuroimaging 2018;28:36–47. 10.1111/jon.12491 29235194 PMC5814929

[128] Mehta S, Grabowski TJ, Trivedi Y, et al. Evaluation of voxel-based morphometry for focal lesion detection in individuals. Neuroimage 2003;20:1438–54. 10.1016/S1053-8119(03)00377-X 14642458

[129] Mechrez R, Goldberger J, Greenspan H. Patch-based segmentation with spatial consistency: application to MS lesions in brain MRI. Int J Biomed Imaging 2016;2016 10.1155/2016/7952541 PMC4745344 26904103

[130] McKinley R, Häni L, Gralla J, et al. Fully automated stroke tissue estimation using random forest classifiers (faster). J Cereb Blood Flow Metab 2017;37:2728–41. 10.1177/0271678X16674221 27798267 PMC5536784

[131] Mazzara GP, Velthuizen RP, Pearlman JL, et al. Brain tumor target volume determination for radiation treatment planning through automated MRI segmentation. Int J Radiat Oncol Biol Phys 2004;59:300–12. 10.1016/j.ijrobp.2004.01.026 15093927

[132] Manjón JV, Coupé P, Raniga P, et al. MRI white matter lesion segmentation using an ensemble of neural networks and overcomplete patch-based voting. Comput Med Imaging Graph 2018;69:43–51. 10.1016/j.compmedimag.2018.05.001 30172092

[133] Maldjian JA, Whitlow CT, Saha BN, et al. Automated white matter total lesion volume segmentation in diabetes. AJNR Am J Neuroradiol 2013;34:2265–70. 10.3174/ajnr.A3590 23868156 PMC4038900

[134] Maji P, Roy S. SoBT-RFW: Rough-Fuzzy computing and wavelet analysis based automatic brain tumor detection method from MR images. Fundam Inform 2015;142:237–67. 10.3233/FI-2015-1293

[135] Maier O, Wilms M, von der Gablentz J, et al. Extra tree forests for sub-acute ischemic stroke lesion segmentation in Mr sequences. J Neurosci Methods 2015;240:89–100. 10.1016/j.jneumeth.2014.11.011 25448384

[136] Maier O, Schröder C, Forkert ND, et al. Classifiers for ischemic stroke lesion segmentation: a comparison study. PLoS One 2015;10:e0145118. 10.1371/journal.pone.0145118 26672989 PMC4687679

[137] Maier O. MS lesion segmentation in MRI with random forests. Proc 2015 longitudinal multiple sclerosis lesion segmentation challenge 2015:1–2.

[138] Mah Y-H, Jager R, Kennard C, et al. A new method for automated high-dimensional lesion segmentation evaluated in vascular injury and applied to the human occipital lobe. Cortex 2014;56:51–63. 10.1016/j.cortex.2012.12.008 23347558 PMC4071441

[139] Ma C, Luo G, Wang K. Concatenated and connected random forests with multiscale patch driven active contour model for automated brain tumor segmentation of Mr images. IEEE Trans Med Imaging 2018;37:1943–54. 10.1109/TMI.2018.2805821 29994627

[140] Lu Y, Jiang J, Yang W, et al. Multimodal brain-tumor segmentation based on Dirichlet process mixture model with anisotropic diffusion and Markov random field prior. Comput Math Methods Med 2014;2014:e717206 10.1155/2014/717206 25254064 PMC4164260

[141] Liu Y, Stojadinovic S, Hrycushko B, et al. A deep convolutional neural network-based automatic delineation strategy for multiple brain metastases stereotactic radiosurgery. PLoS One 2017;12:e0185844. 10.1371/journal.pone.0185844 28985229 PMC5630188

[142] Liu Y, Stojadinovic S, Hrycushko B, et al. Automatic metastatic brain tumor segmentation for stereotactic radiosurgery applications. Phys Med Biol 2016;61:8440–61. 10.1088/0031-9155/61/24/8440 27845915

[143] Liu J, Chen F, Pan C, et al. A Cascaded deep Convolutional neural network for joint segmentation and genotype prediction of brainstem gliomas. IEEE Trans Biomed Eng 2018;65:1943–52. 10.1109/TBME.2018.2845706 29993462

[144] Ling Y, Jouvent E, Cousyn L, et al. Validation and optimization of BIANCA for the segmentation of extensive white matter hyperintensities. Neuroinformatics 2018;16:269–81. 10.1007/s12021-018-9372-2 29594711

[145] Liberman G, Louzoun Y, Aizenstein O, et al. Automatic multi-modal MR tissue classification for the assessment of response to bevacizumab in patients with glioblastoma. Eur J Radiol 2013;82:e87–94. 10.1016/j.ejrad.2012.09.001 23017192

[146] Li Z, Wang Y, Yu J, et al. Low-Grade glioma segmentation based on CNN with fully connected CRF. J Healthc Eng 2017;2017:e9283480 10.1155/2017/9283480 PMC5485483 29065666

[147] Li Y, Jia F, Qin J. Brain tumor segmentation from multimodal magnetic resonance images via sparse representation. Artif Intell Med 2016;73:1–13. 10.1016/j.artmed.2016.08.004 27926377

[148] Li W, Tian J, Li E, et al. Robust unsupervised segmentation of infarct lesion from diffusion tensor MR images using multiscale statistical classification and partial volume voxel reclassification. Neuroimage 2004;23:1507–18. 10.1016/j.neuroimage.2004.08.009 15589114

[149] Li H, Jiang G, Zhang J, et al. Fully convolutional network ensembles for white matter hyperintensities segmentation in MR images. Neuroimage 2018;183:650–65. 10.1016/j.neuroimage.2018.07.005 30125711

[150] Ž L, Pernuš F, Likar B. Validation of white-matter lesion change detection methods on a novel publicly available MRI image database. Neuroinform 2016;14:403–20. 10.1007/s12021-016-9301-1 27207310

[151] Laukamp KR, Thiele F, Shakirin G, et al. Fully automated detection and segmentation of meningiomas using deep learning on routine multiparametric MRI. Eur Radiol 2019;29:124–32. 10.1007/s00330-018-5595-8 29943184 PMC6291436

[152] Lao Z, Shen D, Liu D, et al. Computer-assisted segmentation of white matter lesions in 3D MR images using support vector machine. Acad Radiol 2008;15:300–13. 10.1016/j.acra.2007.10.012 18280928 PMC2528894

[153] Korfiatis P, Kline TL, Erickson BJ. Automated segmentation of hyperintense regions in FLAIR MRI using deep learning. Tomography 2016;2:334–40. 28066806 10.18383/j.tom.2016.00166 PMC5215737

[154] Knight J, Taylor GW, Khademi A. Voxel-Wise logistic regression and leave-one-source-out cross validation for white matter hyperintensity segmentation. Magn Reson Imaging 2018;54:119–36. 10.1016/j.mri.2018.06.009 29932970

[155] Klöppel S, Abdulkadir A, Hadjidemetriou S, et al. A comparison of different automated methods for the detection of white matter lesions in MRI data. Neuroimage 2011;57:416–22. 10.1016/j.neuroimage.2011.04.053 21569857

[156] Khotanlou H, Afrasiabi M. Segmentation of multiple sclerosis lesions in brain Mr images using spatially constrained possibilistic fuzzy C-Means classification. J Med Signals Sens 2011;1:1–55. 10.4103/2228-7477.95278 22606670 PMC3347225

[157] Khayati R, Vafadust M, Towhidkhah F, et al. Fully automatic segmentation of multiple sclerosis lesions in brain MR FLAIR images using adaptive mixtures method and Markov random field model. Comput Biol Med 2008;38:379–90. 10.1016/j.compbiomed.2007.12.005 18262511

[158] Khayati R, Vafadust M, Towhidkhah F, et al. A novel method for automatic determination of different stages of multiple sclerosis lesions in brain MR FLAIR images. Comput Med Imaging Graph 2008;32:124–33. 10.1016/j.compmedimag.2007.10.003 18055174

[159] Khademi A, Venetsanopoulos A, Moody AR. Robust white matter lesion segmentation in FLAIR MRI. IEEE Trans Biomed Eng 2012;59:860–71. 10.1109/TBME.2011.2181167 22203699

[160] Kellner-Weldon F, Stippich C, Wiest R, et al. Comparison of perioperative automated versus manual two-dimensional tumor analysis in glioblastoma patients. Eur J Radiol 2017;95:75–81. 10.1016/j.ejrad.2017.07.028 28987701

[161] Kellner E, Reisert M, Kiselev VG. Automated infarct core volumetry within the hypoperfused tissue: technical implementation and evaluation. J Comput Assist Tomogr 2017;41:515–20. 27997443 10.1097/RCT.0000000000000570

[162] Keçeli AS, Can AB, Kaya A. A GPU-Based approach for automatic segmentation of white matter lesions. IETE J Res 2017;63:461–72. 10.1080/03772063.2017.1284619

[163] Kaur T, Saini BS, Gupta S. A joint intensity and edge magnitude-based multilevel thresholding algorithm for the automatic segmentation of pathological MR brain images. Neural Comput & Applic 2018;30:1317–40. 10.1007/s00521-016-2751-4

[164] Kaur T, Saini BS, Gupta S. A novel fully automatic multilevel thresholding technique based on optimized intuitionistic fuzzy sets and tsallis entropy for MR brain tumor image segmentation. Australas Phys Eng Sci Med 2018;41:41–58. 10.1007/s13246-017-0609-4 29238919

[165] Karimian A, Jafari S. A new method to segment the multiple sclerosis lesions on brain magnetic resonance images. J Med Signals Sens 2015;5:238–44. 10.4103/2228-7477.168653 26955567 PMC4759840

[166] Karimaghaloo Z, Arnold DL, Arbel T. Adaptive multi-level conditional random fields for detection and segmentation of small enhanced pathology in medical images. Med Image Anal 2016;27:17–30. 10.1016/j.media.2015.06.004 26211811

[167] Karimaghaloo Z, Shah M, Francis SJ, et al. Automatic detection of gadolinium-enhancing multiple sclerosis lesions in brain MRI using conditional random fields. IEEE Trans Med Imaging 2012;31:1181–94. 10.1109/TMI.2012.2186639 22318484

[168] Karimaghaloo Z, Rivaz H, Arnold DL, et al. Temporal hierarchical adaptive texture CRF for automatic detection of gadolinium-enhancing multiple sclerosis lesions in brain MRI. IEEE Trans Med Imaging 2015;34:1227–41. 10.1109/TMI.2014.2382561 25532171

[169] Kanas VG, Zacharaki EI, Davatzikos C, et al. A low cost approach for brain tumor segmentation based on intensity modeling and 3D random Walker. Biomed Signal Process Control 2015;22:19–30. 10.1016/j.bspc.2015.06.004

[170] Kamnitsas K, Ledig C, Newcombe VFJ, et al. Efficient multi-scale 3D CNN with fully connected CRF for accurate brain lesion segmentation. Med Image Anal 2017;36:61–78. 10.1016/j.media.2016.10.004 27865153

[171] Kamber M, Shinghal R, Collins DL, et al. Model-based 3-D segmentation of multiple sclerosis lesions in magnetic resonance brain images. IEEE Trans Med Imaging 1995;14:442–53. 10.1109/42.414608 18215848

[172] Juan-Albarracín J, Fuster-Garcia E, Manjón JV, et al. Automated glioblastoma segmentation based on a multiparametric structured unsupervised classification. PLoS One 2015;10:e0125143. 10.1371/journal.pone.0125143 25978453 PMC4433123

[173] Jiang J, Liu T, Zhu W, et al. UBO detector – a cluster-based, fully automated pipeline for extracting white matter hyperintensities. Neuroimage 2018;174:539–49. 10.1016/j.neuroimage.2018.03.050 29578029

[174] Ji S, Ye C, Li F, et al. Automatic segmentation of white matter hyperintensities by an extended FitzHugh & Nagumo reaction diffusion model. J Magn Reson Imaging 2013;37:343–50. 10.1002/jmri.23836 23023955

[175] Jeon S, Yoon U, Park J-S. Fully automated pipeline for quantification and localization of white matter hyperintensity in brain magnetic resonance image. Int J Imaging Syst Technol 2011;21:193–200. 10.1002/ima.20277

[176] Jansen JFA, Vlooswijk MCG, Majoie HJM, et al. White matter lesions in patients with localization-related epilepsy. Invest Radiol 2008;43:552–8. 10.1097/RLI.0b013e31817e90d2 18648254

[177] Jain S, Sima DM, Ribbens A, et al. Automatic segmentation and volumetry of multiple sclerosis brain lesions from MR images. Neuroimage 2015;8:367–75. 10.1016/j.nicl.2015.05.003 26106562 PMC4474324

[178] Ithapu V, Singh V, Lindner C, et al. Extracting and summarizing white matter hyperintensities using supervised segmentation methods in Alzheimer’s disease risk and aging studies. Hum Brain Mapp 2014;35:4219–35. 10.1002/hbm.22472 24510744 PMC4107160

[179] Islam A, Reza SMS, Iftekharuddin KM. Multifractal texture estimation for detection and segmentation of brain tumors. IEEE Trans Biomed Eng 2013;60:3204–15. 10.1109/TBME.2013.2271383 23807424 PMC5126980

[180] Iqbal S, Ghani MU, Saba T, et al. Brain tumor segmentation in multi-spectral MRI using convolutional neural networks (CNN). Microsc Res Tech 2018;81:419–27. 10.1002/jemt.22994 29356229

[181] Ilunga–Mbuyamba E, Avina–Cervantes JG, Cepeda–Negrete J, et al. Automatic selection of localized region-based active contour models using image content analysis applied to brain tumor segmentation. Comput Biol Med 2017;91:69–79. 10.1016/j.compbiomed.2017.10.003 29049909

[182] Ilunga-Mbuyamba E, Cruz-Duarte JM, Avina-Cervantes JG, et al. Active contours driven by cuckoo search strategy for brain tumour images segmentation. Expert Syst Appl 2016;56:59–68. 10.1016/j.eswa.2016.02.048

[183] Hussain S, Anwar SM, Majid M. Segmentation of glioma tumors in brain using deep convolutional neural network. Neurocomputing 2018;282:248–61. 10.1016/j.neucom.2017.12.032

[184] Hulsey KM, Gupta M, King KS, et al. Automated quantification of white matter disease extent at 3 T: comparison with volumetric readings. J Magn Reson Imaging 2012;36:305–11. 10.1002/jmri.23659 22517404

[185] Huang M, Yang W, Wu Y, et al. Brain tumor segmentation based on local independent Projection-Based classification. IEEE Trans Biomed Eng 2014;61:2633–45. 10.1109/TBME.2014.2325410 24860022

[186] Herskovits E, Bryan R, Yang F. Automated Bayesian segmentation of microvascular white-matter lesions in the ACCORD-MIND study. Adv Med Sci 2008;53. 10.2478/v10039-008-0039-3 18842559

[187] Havaei M, Davy A, Warde-Farley D, et al. Brain tumor segmentation with deep neural networks. Med Image Anal 2017;35:18–31. 10.1016/j.media.2016.05.004 27310171

[188] Harmouche R, Subbanna NK, Collins DL, et al. Probabilistic multiple sclerosis lesion classification based on modeling regional intensity variability and local neighborhood information. IEEE Trans Biomed Eng 2015;62:1281–92. 10.1109/TBME.2014.2385635 25546852

[189] Harati V, Khayati R, Farzan A. Fully automated tumor segmentation based on improved fuzzy connectedness algorithm in brain Mr images. Comput Biol Med 2011;41:483–92. 10.1016/j.compbiomed.2011.04.010 21601840

[190] Hansen MB, Nagenthiraja K, Ribe LR, et al. Automated estimation of salvageable tissue: comparison with expert readers. Journal of Magnetic Resonance Imaging 2016;43:220–8. 10.1002/jmri.24963 26036930

[191] Gupta N, Bhatele P, Khanna P. Identification of gliomas from brain MRI through adaptive segmentation and run length of centralized patterns. J Comput Sci 2018;25:213–20. 10.1016/j.jocs.2017.02.009

[192] Guo D, Fridriksson J, Fillmore P, et al. Automated lesion detection on MRI scans using combined unsupervised and supervised methods. BMC Med Imaging 2015;15:50. 10.1186/s12880-015-0092-x 26518734 PMC4628334

[193] Guizard N, Coupé P, Fonov VS, et al. Rotation-invariant multi-contrast non-local means for MS lesion segmentation. Neuroimage 2015;8:376–89. 10.1016/j.nicl.2015.05.001 26106563 PMC4474283

[194] Guerrero R, Qin C, Oktay O, et al. White matter hyperintensity and stroke lesion segmentation and differentiation using convolutional neural networks. Neuroimage 2018;17:918–34. 10.1016/j.nicl.2017.12.022 29527496 PMC5842732

[195] Griffis JC, Allendorfer JB, Szaflarski JP. Voxel-based Gaussian naïve Bayes classification of ischemic stroke lesions in individual T1-weighted MRI scans. J Neurosci Methods 2016;257:97–108. 10.1016/j.jneumeth.2015.09.019 26432931 PMC4662880

[196] Griffanti L, Zamboni G, Khan A, et al. BIANCA (brain intensity abnormality classification algorithm): a new tool for automated segmentation of white matter hyperintensities. Neuroimage 2016;141:191–205. 10.1016/j.neuroimage.2016.07.018 27402600 PMC5035138

[197] Gooya A, Pohl KM, Bilello M, et al. GLISTR: glioma image segmentation and registration. IEEE Trans Med Imaging 2012;31:1941–54. 10.1109/TMI.2012.2210558 22907965 PMC4371551

[198] Gonçalves N, Nikkilä J, Vigário R. Self-supervised MRI tissue segmentation by discriminative clustering. Int J Neural Syst 2014;24:1450004. 10.1142/S012906571450004X 24344692

[199] Goetz M, Weber C, Binczyk F, et al. DALSA: domain adaptation for supervised learning from sparsely annotated Mr images. IEEE Trans Med Imaging 2016;35:184–96. 10.1109/TMI.2015.2463078 26259241

[200] Ghribi O, Sellami L, Slima MB, et al. Multiple sclerosis exploration based on automatic MRI modalities segmentation approach with advanced volumetric evaluations for essential feature extraction. Biomed Signal Process Control 2018;40:473–87. 10.1016/j.bspc.2017.07.008

[201] Ghribi O, Sellami L, Ben Slima M, Slima MB, et al. An advanced MRI Multi-Modalities segmentation methodology dedicated to multiple sclerosis lesions exploration and differentiation. IEEE Trans Nanobioscience 2017;16:656–65. 10.1109/TNB.2017.2763246 29035222

[202] Ghafoorian M, Karssemeijer N, van Uden IWM. Automated detection of white matter hyperintensities of all sizes in cerebral small vessel disease. Med Phys 2016;43:6246. 27908171 10.1118/1.4966029

[203] Ghafoorian M, Karssemeijer N, Heskes T. Location sensitive deep Convolutional neural networks for segmentation of white matter hyperintensities. Scientific Reports 2017;7:5110. 28698556 10.1038/s41598-017-05300-5 PMC5505987

[204] Ghafoorian M, Karssemeijer N, Heskes T. Deep multi-scale location-aware 3D convolutional neural networks for automated detection of lacunes of presumed vascular origin. NeuroImage: Clinical 2017;14:391–9. 28271039 10.1016/j.nicl.2017.01.033 PMC5322213

[205] Geremia E, Clatz O, Menze BH. Spatial decision forests for MS lesion segmentation in multi-channel magnetic resonance images. NeuroImage 2011;57:378–90. 21497655 10.1016/j.neuroimage.2011.03.080

[206] García-Lorenzo D, Prima S, Arnold DL. Trimmed-likelihood estimation for focal lesions and tissue segmentation in multisequence MRI for multiple sclerosis. IEEE Trans Med Imaging 2011;30:1455–67. 21324773 10.1109/TMI.2011.2114671 PMC3326634

[207] Gao J, Li C, Feng C. Non-locally regularized segmentation of multiple sclerosis lesion from multi-channel MRI data. Magn Reson Imaging 2014;32:1058–66. 24948583 10.1016/j.mri.2014.03.006 PMC4373080

[208] Ganiler O, Oliver A, Diez Y. A subtraction pipeline for automatic detection of new appearing multiple sclerosis lesions in longitudinal studies. Neuroradiology 2014;56:363–74. 24590302 10.1007/s00234-014-1343-1

[209] Galimzianova A, Pernuš F, Likar B. Stratified mixture modeling for segmentation of white-matter lesions in brain MR images. Neuroimage 2016;124:1031–43. 26427644 10.1016/j.neuroimage.2015.09.047

[210] Galimzianova A, Ž L, Rubin DL. Locally adaptive magnetic resonance intensity models for unsupervised segmentation of multiple sclerosis lesions. J Med Imaging 2018;5. 10.1117/1.JMI.5.1.011007 PMC5665678 29134190

[211] Freire PGL, Ferrari RJ. Automatic iterative segmentation of multiple sclerosis lesions using student’s T mixture models and probabilistic anatomical atlases in FLAIR images. Comput Biol Med 2016;73:10–23. 10.1016/j.compbiomed.2016.03.025 27058437

[212] Fiot J-B, Cohen LD, Raniga P. Efficient brain lesion segmentation using multi-modality tissue-based feature selection and support vector machines. Int J Numer Method Biomed Eng 2013;29:905–15. 23303595 10.1002/cnm.2537

[213] Fartaria MJ, Todea A, Kober T. Partial volume-aware assessment of multiple sclerosis lesions. Neuroimage Clin 2018;18:245–53. 29868448 10.1016/j.nicl.2018.01.011 PMC5984601

[214] Fartaria MJ, Bonnier G, Roche A, et al. Automated detection of white matter and cortical lesions in early stages of multiple sclerosis. J Magn Reson Imaging 2016;43:1445–54. 10.1002/jmri.25095 26606758

[215] Essadike A, Ouabida E, Bouzid A. Brain tumor segmentation with Vander Lugt correlator based active contour. Comput Methods Programs Biomed 2018;160:103–17. 10.1016/j.cmpb.2018.04.004 29728237

[216] Erus G, Zacharaki EI, Davatzikos C. Individualized statistical learning from medical image databases: application to identification of brain lesions. Med Image Anal 2014;18:542–54. 24607564 10.1016/j.media.2014.02.003 PMC4001866

[217] Emblem KE, Nedregaard B, Hald JK. Automatic glioma characterization from dynamic susceptibility contrast imaging: brain tumor segmentation using knowledge-based fuzzy clustering. J Magn Reson Imaging 2009;30:1–10. 19557840 10.1002/jmri.21815

[218] Elliott C, Arnold DL, Collins DL. Temporally consistent probabilistic detection of new multiple sclerosis lesions in brain MRI. IEEE Trans Med Imaging 2013;32:1490–503. 23613032 10.1109/TMI.2013.2258403

[219] Dyrby TB, Rostrup E, Baaré WFC. Segmentation of age-related white matter changes in a clinical multi-center study. NeuroImage 2008;41:335–45. 18394928 10.1016/j.neuroimage.2008.02.024

[220] Dvořák P, Bartusek K, Smékal Z. Unsupervised pathological area extraction using 3D T2 and FLAIR Mr images 2014.

[221] Ding Y, Dong R, Lan T, et al. Multi-modal brain tumor image segmentation based on SDAE. Int J Imaging Syst Technol 2018;28:38–47. 10.1002/ima.22254

[222] Dickson S, Thomas BT, Goddard P. Using neural networks to automatically detect brain tumours in MR images. Int J Neural Syst 1997;8:91–9. 9228581 10.1142/s0129065797000124

[223] Demirhan A, Törü M, Guler I. Segmentation of tumor and edema along with healthy tissues of brain using wavelets and neural networks. IEEE J Biomed Health Inform 2015;19:1451–8. 10.1109/JBHI.2014.2360515 25265636

[224] de Sitter A, Steenwijk MD, Ruet A. Performance of five research-domain automated WM lesion segmentation methods in a multi-center MS study. Neuroimage 2017;163:106–14. 28899746 10.1016/j.neuroimage.2017.09.011

[225] de Boer R, Vrooman HA, van der Lijn F. White matter lesion extension to automatic brain tissue segmentation on MRI. NeuroImage 2009;45:1151–61. 19344687 10.1016/j.neuroimage.2009.01.011

[226] Datta S, Sajja BR, He R. Segmentation of gadolinium-enhanced lesions on MRI in multiple sclerosis. J Magn Reson Imaging 2007;25:932–7. 17457804 10.1002/jmri.20896

[227] Datta S, Narayana PA. A comprehensive approach to the segmentation of multichannel three-dimensional MR brain images in multiple sclerosis. Neuroimage Clin 2013;2:184–96. 24179773 10.1016/j.nicl.2012.12.007 PMC3777770

[228] Damangir S, Westman E, Simmons A. Reproducible segmentation of white matter hyperintensities using a new statistical definition. Magn Reson Mater Phy 2017;30:227–37. 10.1007/s10334-016-0599-3 PMC5440501 27943055

[229] Damangir S, Manzouri A, Oppedal K. Multispectral MRI segmentation of age related white matter changes using a cascade of support vector machines. J Neurol Sci 2012;322:211–6. 22921728 10.1016/j.jns.2012.07.064

[230] Dadar M, Maranzano J, Misquitta K. Performance comparison of 10 different classification techniques in segmenting white matter hyperintensities in aging. Neuroimage 2017;157:233–49. 28602597 10.1016/j.neuroimage.2017.06.009 PMC6469398

[231] Dadar M, Maranzano J, Ducharme S. Validation of T1w‐based segmentations of white matter hyperintensity volumes in large‐scale datasets of aging. Hum Brain Mapp 2017;39:1093–107. 29181872 10.1002/hbm.23894 PMC6866430

[232] Dadar M, Pascoal TA, Manitsirikul S, et al. Validation of a regression technique for segmentation of white matter hyperintensities in Alzheimer’s disease. IEEE Trans Med Imaging 2017;36:1758–68. 10.1109/TMI.2017.2693978 28422655

[233] Cui S, Mao L, Xiong S. Brain tumor automatic segmentation using fully Convolutional networks. J Med Imaging Health Inform 2017;7:1641–7. 10.1166/jmihi.2017.2179

[234] Cui S, Mao L, Jiang J. Automatic semantic segmentation of brain gliomas from MRI images using a deep cascaded neural network. J Healthc Eng 2018;2018. 10.1155/2018/4940593 PMC5884212 29755716

[235] Corso JJ, Sharon E, Dube S. Efficient multilevel brain tumor segmentation with integrated bayesian model classification. IEEE Trans Med Imaging 2008;27:629–40. 18450536 10.1109/TMI.2007.912817

[236] Cordier N, Delingette H, Ayache N. A patch-based approach for the segmentation of pathologies: application to glioma labelling. IEEE Trans Med Imaging 2016;35:1066–76. 10.1109/TMI.2015.2508150 26685225

[237] Commowick O, Maarouf A, Ferré J-C, et al. Diffusion MRI abnormalities detection with orientation distribution functions: a multiple sclerosis longitudinal study. Med Image Anal 2015;22:114–23. 10.1016/j.media.2015.02.005 25867549

[238] Colliot O, Mansi T, Bernasconi N. Segmentation of focal cortical dysplasia lesions on MRI using level set evolution. Neuroimage 2006;32:1621–30. 16887367 10.1016/j.neuroimage.2006.04.225

[239] Clarke LP, Velthuizen RP, Clark M, et al. Mri measurement of brain tumor response: comparison of visual metric and automatic segmentation. Magn Reson Imaging 1998;16:271–9. 10.1016/S0730-725X(97)00302-0 9621968

[240] Cheriyan MM, Michael PA, Kumar A. Blind source separation with mixture models - A hybrid approach to MR brain classification. Magn Reson Imaging 2018;54:137–47. 10.1016/j.mri.2018.08.023 30172941

[241] Chen W, Liu B, Peng S, et al. Computer-Aided grading of gliomas combining automatic segmentation and Radiomics. Int J Biomed Imaging 2018;2018:e2512037 10.1155/2018/2512037 29853828 PMC5964423

[242] Chen L, Bentley P, Rueckert D. Fully automatic acute ischemic lesion segmentation in DWI using convolutional neural networks. Neuroimage Clin 2017;15:633–43. 28664034 10.1016/j.nicl.2017.06.016 PMC5480013

[243] Charron O, Lallement A, Jarnet D, et al. Automatic detection and segmentation of brain metastases on multimodal MR images with a deep convolutional neural network. Comput Biol Med 2018;95:43–54. 10.1016/j.compbiomed.2018.02.004 29455079

[244] Cabria I, Gondra I. MRI segmentation fusion for brain tumor detection. Information Fusion 2017;36:1–9. 10.1016/j.inffus.2016.10.003

[245] Cabezas M, Oliver A, Valverde S, et al. Boost: a supervised approach for multiple sclerosis lesion segmentation. J Neurosci Methods 2014;237:108–17. 10.1016/j.jneumeth.2014.08.024 25194638

[246] Cabezas M, Oliver A, Roura E, et al. Automatic multiple sclerosis lesion detection in brain MRI by FLAIR thresholding. Comput Methods Programs Biomed 2014;115:147–61. 10.1016/j.cmpb.2014.04.006 24813718

[247] Cabezas M, Corral JF, Oliver A, et al. Improved automatic detection of new T2 lesions in multiple sclerosis using deformation fields. AJNR Am J Neuroradiol 2016;37:1816–23. 10.3174/ajnr.A4829 27282863 PMC7960461

[248] Brosch T, Tang LYW et al. Deep 3D convolutional encoder networks with shortcuts for multiscale feature integration applied to multiple sclerosis lesion segmentation. IEEE Trans Med Imaging 2016;35:1229–39. 10.1109/TMI.2016.2528821 26886978

[249] Bowles C, Qin C, Guerrero R, et al. Brain lesion segmentation through image synthesis and outlier detection. Neuroimage Clin 2017;16:643–58. 10.1016/j.nicl.2017.09.003 29868438 PMC5984574

[250] Boudraa AO, Dehak SM, Zhu YM. Automated segmentation of multiple sclerosis lesions in multispectral MR imaging using fuzzy clustering. Comput Biol Med 2000;30:23–40. 10695813 10.1016/s0010-4825(99)00019-0

[251] Bonte S, Goethals I, Van Holen R. Machine learning based brain tumour segmentation on limited data using local texture and abnormality. Comput Biol Med 2018;98:39–47. 10.1016/j.compbiomed.2018.05.005 29763764

[252] Boldsen JK, Engedal TS, Pedraza S. Better diffusion segmentation in acute ischemic stroke through automatic tree learning anomaly segmentation. Front Neuroinform 2018;12. 10.3389/fninf.2018.00021 PMC5996895 29910721

[253] Binczyk F, Stjelties B, Weber C, et al. MiMSeg - an algorithm for automated detection of tumor tissue on NMR apparent diffusion coefficient maps. Inf Sci 2017;384:235–48. 10.1016/j.ins.2016.07.052

[254] Binaghi E, Pedoia V, Balbi S. Meningioma and peritumoral edema segmentation of preoperative MRI brain scans. Comput Methods Biomech Biomed Engin 2018;6:362–70. 10.1080/21681163.2016.1250108

[255] Bijar A, Khayati R, Benavent AP. Increasing the contrast of the brain MR FLAIR images using fuzzy membership functions and structural similarity indices in order to segment MS lesions. Plos One 2013;8:e65469. 23799015 10.1371/journal.pone.0065469 PMC3684600

[256] Bijar A, Khanloo MM, Benavent AP, et al. Segmentation of MS lesions using entropy-based em algorithm and Markov random fields. J Biomed Sci Eng 2011;04:552–61. 10.4236/jbise.2011.48071

[257] Bhanu Prakash KN, Gupta V, Jianbo H. Automatic processing of diffusion-weighted ischemic stroke images based on divergence measures: slice and hemisphere identification, and stroke region segmentation. Int J CARS 2008;3:559–70.

[258] Beare R, Srikanth V, Chen J. Development and validation of morphological segmentation of age-related cerebral white matter hyperintensities. Neuroimage 2009;47:199–203. 19344777 10.1016/j.neuroimage.2009.03.055

[259] Banerjee S, Mitra S, Uma Shankar B. Automated 3D segmentation of brain tumor using visual saliency. Inf Sci 2018;424:337–53. 10.1016/j.ins.2017.10.011

[260] Banerjee S, Mitra S, Shankar BU. A novel GBM Saliency detection model using multi-channel MRI. Plos One 2016;11:e0146388. 26752735 10.1371/journal.pone.0146388 PMC4709039

[261] Asman AJ, Chambless LB, Thompson RC. Out-of-atlas likelihood estimation using multi-atlas segmentation. Med Phys 2013;40. 10.1118/1.4794478 PMC3625241 23556928

[262] MeghaP A, Reddy GRM. Computer-aided diagnosis system for tissue characterization of brain tumor on magnetic resonance images. SIViP 2015;9:409–25.

[263] Anbeek P, Vincken KL, Viergever MA. Automated MS-Lesion segmentation by k-nearest neighbor classification. MIDAS J 2008;610.

[264] Anbeek P, Vincken KL, van Osch MJP, et al. Automatic segmentation of different-sized white matter lesions by voxel probability estimation. Med Image Anal 2004;8:205–15. 10.1016/j.media.2004.06.019 15450216

[265] Amiri S, Ali Mahjoub M, Rekik I. Tree-Based ensemble classifier learning for automatic brain glioma segmentation. Neurocomputing 2018;313:135–42.

[266] Alex V, Vaidhya K, Thirunavukkarasu S. Semisupervised learning using denoising autoencoders for brain lesion detection and segmentation. J Med Imaging 2017;4:041311. 10.1117/1.JMI.4.4.041311 PMC5730366 29285516

[267] Alegro MdeC, Amaro Junior E, Lopes RdeD. Computerized brain tumor segmentation in magnetic resonance imaging. Einstein 2012;10:158–63. 10.1590/S1679-45082012000200008 23052450

[268] AlBadawy EA, Saha A, Mazurowski MA. Deep learning for segmentation of brain tumors: impact of cross-institutional training and testing. Med Phys 2018;45:1150–8. 10.1002/mp.12752 29356028

[269] Salman Al-Shaikhli SD, Yang MY, Rosenhahn B. Brain tumor classification and segmentation using sparse coding and dictionary learning. Biomed Tech 2016;61:413–29. 10.1515/bmt-2015-0071 26351901

[270] Akselrod-Ballin A, Galun M, Gomori JM. Automatic segmentation and classification of multiple sclerosis in multichannel MRI. IEEE Trans Biomed Eng 2009;56:2461–9. 19758850 10.1109/TBME.2008.926671

[271] Ahmed B, Brodley CE, Blackmon KE. Cortical feature analysis and machine learning improves detection of “MRI-negative” focal cortical dysplasia. Epilepsy Behav 2015;48:21–8. 26037845 10.1016/j.yebeh.2015.04.055 PMC4500682

[272] Admiraal-Behloul F, van den Heuvel DMJ, Olofsen H. Fully automatic segmentation of white matter hyperintensities in MR images of the elderly. Neuroimage 2005;28:607–17. 16129626 10.1016/j.neuroimage.2005.06.061

[273] Adler S, Wagstyl K, Gunny R. Novel surface features for automated detection of focal cortical dysplasias in paediatric epilepsy. Neuroimage Clin 2016;14:18–27. 28123950 10.1016/j.nicl.2016.12.030 PMC5222951

[274] Abdulraqeb AR, Al-Haidri WA, Sushkova LT. An automated method for Segmenting brain tumors on MRI images. Biomed Eng 2017;51:97–101.

[275] Abdullah BA, Younis AA, John NM. Multi-Sectional views textural based SVM for MS lesion segmentation in Multi-Channels MRIs. Open Biomed Eng J 2012;6:56–72. 22741026 10.2174/1874230001206010056 PMC3382289

[276] Abd-Ellah MK, Awad AI, Khalaf AAM, et al. Two-phase multi-model automatic brain tumour diagnosis system from magnetic resonance images using convolutional neural networks. EURASIP J Image Video Process 2018;2018:97. 10.1186/s13640-018-0332-4

[277] Abbasi S, Tajeripour F. Detection of brain tumor in 3D MRI images using local binary patterns and histogram orientation gradient. Neurocomputing 2017;219:526–35.

[278] Agam G, Weiss D, Soman M. Probabilistic brain lesion segmentation in DT-MRI. in: 2006 International Conference on image processing 2006:89–92.

[279] Agn M, Law I, Rosenschöld P, et al. A generative model for segmentation of tumor and organs-at-risk for radiation therapy planning of glioblastoma patients. In: Progress in Biomedical Optics and Imaging - Proceedings of SPIE. SPIE 2016.

[280] Agn M, Puonti O. Brain tumor segmentation using a generative model with an RBM prior on tumor shape. In: Crimi A, Menze B, Maier O, eds. Brainlesion: glioma, multiple sclerosis, stroke and traumatic brain injuries. Cham: Springer International Publishing, 2016: 168–80.

[281] Akter MK, Khan SM, Azad S. Automated brain tumor segmentation from MRI data based on exploration of histogram characteristics of the cancerous hemisphere. in: 2017 IEEE region 10 humanitarian technology conference (R10-HTC) 2017:815–8.

[282] Al-Shaikhli SDS, Yang MY, Rosenhahn B. Coupled dictionary learning for automatic multi-label brain tumor segmentation in flair MRI images. In: Bebis G, Boyle R, Parvin B, eds. Advances in visual computing. Cham: Springer International Publishing, 2014: 489–500.

[283] Alex V, Safwan KPM, Chennamsetty SS. Generative adversarial networks for brain lesion detection. In: Medical Imaging 2017: Image Processing 2017.

[284] Amiri S, Rekik I, Mahjoub MA. Deep random forest-based learning transfer to SVM for brain tumor segmentation. in: 2016 2nd International Conference on advanced technologies for signal and image processing (ATSIP) 2016:297–302.

[285] Amiri S, Mahjoub M, Rekik I. Bayesian network and structured random forest cooperative deep learning for automatic Multi-label brain tumor segmentation. ICAART, 2018: 183–90.

[286] Ananda RS, Thomas T. Automatic segmentation framework for primary tumors from brain MRIs using morphological filtering techniques. In: 2012 5th International Conference on biomedical engineering and informatics 2012:238–42.

[287] Anwar SM, Yousaf S, Majid M. Brain tumor segmentation on Multimodal MRI scans using EMAP Algorithm. In: 2018 40th Annual International Conference of the IEEE Engineering in Medicine and Biology Society (EMBC) 2018:550–3. 10.1109/EMBC.2018.8512304 30440456

[288] Hoogi A, Lee A, Bharadwa V. Multimodal brain tumor segmentation (BRATS) using sparse coding and 2-layer neural network. in proceedings of the multimodal brain tumor image segmentation challenge 2015;34.

[289] Baid U, Talbar S, Talbar SN. Novel approach for brain tumor segmentation with non negative matrix factorization. In: 2017 International Conference on Innovations in Electronics, Signal Processing and Communication (IESC) 2017:101–5.

[290] Bauer S, Gratz PP, Gralla J. Towards automatic MRI volumetry for treatment selection in acute ischemic stroke patients. In: annual International Conference of the IEEE engineering in medicine and biology Society 2014:1521–4. 10.1109/EMBC.2014.6943891 25570259

[291] Bauer S, Nolte L-P, Reyes M. Fully automatic segmentation of brain tumor images using support vector machine classification in combination with hierarchical conditional random field regularization. In: Fichtinger G, Martel A, Peters T, eds. Medical image computing and computer-assisted intervention. Berlin, Heidelberg: Springer, 2011: 354–61. 10.1007/978-3-642-23626-6_44 22003719

[292] Behzadfar N, Soltanian-Zadeh H. Automatic segmentation of brain tumors in magnetic resonance images. In: proceedings of 2012 IEEE-EMBS International Conference on biomedical and health informatics 2012:329–32.

[293] Ben Salah M, Diaz I, Greiner R. Fully automated brain tumor segmentation using two MRI modalities. In: Bebis G, Boyle R, Parvin B, eds. Advances in visual computing. Berlin, Heidelberg: Springer, 2013: 30–9.

[294] Benson CC, Deepa V, Lajish VL. Brain tumor segmentation from MR brain images using improved fuzzy C-Means clustering and watershed algorithm. in: 2016 International Conference on advances in computing, communications and informatics (ICACCI) 2016:187–92.

[295] Bento M, de Souza R, Lotufo R. WMH segmentation challenge: a texture-based classification approach. In: Crimi A, Bakas S, Kuijf H, eds. Brainlesion: glioma, multiple sclerosis, stroke and traumatic brain injuries. Cham: Springer International Publishing, 2018: 489–500.

[296] Bento M, Sym Y, Frayne R. Probabilistic segmentation of brain white matter lesions using texture-based classification. In: Karray F, Campilho A, Cheriet F, eds. Image analysis and recognition. Cham: Springer International Publishing, 2017: 71–8.

[297] Bharath HN, Colleman S, Sima DM. Tumor segmentation from multimodal MRI using random forest with superpixel and tensor based feature extraction. In: Crimi A, Bakas S, Kuijf H, eds. Brainlesion: glioma, multiple sclerosis, stroke and traumatic brain injuries. Cham: Springer International Publishing, 2018: 463–73.

[298] Bhattacharya D, Sinha N. An improved approach of high graded glioma segmentation using sparse autoencoder and fuzzy C-Means clustering from multi-modal Mr images. in: medical imaging 2018: biomedical applications in molecular, structural, and functional imaging. International Society for optics and Photonics 2018.

[299] Bianchi A, Miller JV, Tan ET. Brain tumor segmentation with symmetric texture and symmetric intensity-based decision forests. In: Proceedings / IEEE International Symposium on biomedical imaging: from nano to macro 2013:748–51. 10.1109/ISBI.2013.6556583 PMC4232942 25404996

[300] Bijar A, Khayati R. Segmentation of MS lesions using active contour model, adaptive mixtures method and MRF model. In: 2011 7th International Symposium on image and signal processing and analysis (IspA) 2011:159–64.

[301] Bilotta E, Cerasa A, Pantano P. A CNN based algorithm for the automated segmentation of multiple sclerosis lesions. In: Di Chio C, Cagnoni S, Cotta C, eds. Applications of evolutionary computation. Berlin, Heidelberg: Springer, 2010: 211–20.

[302] Birenbaum A, Greenspan H. Longitudinal multiple sclerosis lesion segmentation using multi-view convolutional neural networks. In: Carneiro G, Mateus D, Peter L, eds. Deep learning and data labeling for medical applications. Cham: Springer International Publishing, 2016: 58–67.

[303] Bougacha A, Boughariou J, Slima MB. Comparative study of supervised and unsupervised classification methods: application to automatic MRI glioma brain tumors segmentation. In: 2018 4th International Conference on advanced technologies for signal and image processing (ATSIP) 2018:1–5.

[304] Boussaid H, Kokkinos I, Paragios N. Rapid mode estimation for 3D brain MRI tumor segmentation. In: Heyden A, Kahl F, Olsson C, eds. Energy minimization methods in computer vision and pattern recognition. Berlin, Heidelberg: Springer, 2013: 1–11.

[305] Cabezas M, Oliver A, Freixenet J. A supervised approach for multiple sclerosis lesion segmentation using context features and an outlier map. In: Sanches JM, Micó L, Cardoso JS, eds. Pattern recognition and image analysis. Berlin, Heidelberg: Springer, 2013: 782–9.

[306] Cai H, Verma R, Ou Y. Probabilistic segmentation of brain tumours based on multimodality magnetic resonance images. In: 2007 4th IEEE International Symposium on biomedical imaging: from nano to macro 2007:600–3.

[307] Chang P. Fully convolutional neural networks with hyperlocal features for brain tumor segmentation 2016;118.

[308] Chen L, Wu Y, DSouza AM. MRI tumor segmentation with densely connected 3D CNN. In: medical imaging 2018: image processing International Society for optics and Photonics 2018;105741F.

[309] Chen W, Qiao X, Liu B. Automatic brain tumor segmentation based on features of separated local square. In: 2017 Chinese automation Congress (CAC). 2017:6489–93.

[310] Corso JJ, Sharon E, Yuille A. Multilevel segmentation and integrated bayesian model classification with an application to brain tumor segmentation. In: Larsen R, Nielsen M, Sporring J, eds. Medical image computing and computer-assisted intervention – MICCAI. Berlin, Heidelberg: Springer, 2006: 790–8. 10.1007/11866763_97 17354845

[311] Corso JJ, Yuille A, Sicotte NL. Detection and segmentation of pathological structures by the extended graph-shifts algorithm. In: International Conference on medical image computing and computer-assisted intervention 2007:985–93. 10.1007/978-3-540-75757-3_119 18051154

[312] Dalca AV, Sridharan R, Cloonan L. Segmentation of cerebrovascular pathologies in stroke patients with spatial and shape Priors. In: International Conference on medical image computing and computer-assisted intervention 2014:773–80. 10.1007/978-3-319-10470-6_96 PMC4260817 25485450

[313] Derntl A, Plant C, Gruber P. Stroke lesion segmentation using a probabilistic atlas of cerebral vascular territories. In: Crimi A, Menze B, Maier O, eds. Brainlesion: glioma, multiple sclerosis, stroke and traumatic brain injuries. Cham: Springer International Publishing, 2016: 21–32.

[314] Deshpande H, Maurel P, Barillot C. Adaptive dictionary learning for competitive classification of multiple sclerosis lesions. In: 2015 IEEE 12th International Symposium on biomedical imaging 2015:136–9. 10.1016/j.compmedimag.2015.05.003 26055435

[315] Diaz I, Boulanger P, Greiner R, et al. An automatic brain tumor segmentation tool. Annu Int Conf IEEE Eng Med Biol Soc 2013;2013:3339–42. 10.1109/EMBC.2013.6610256 24110443

[316] Dong H, Yang G, Liu F. Automatic brain tumor detection and segmentation using U-Net based fully convolutional networks. In: Valdés Hernández M, González-Castro V, eds. Medical image understanding and analysis. Cham: Springer International Publishing, 2017: 506–17.

[317] Doyle S, Forbes F, Jaillard A. Sub-acute and chronic ischemic stroke lesion MRI segmentation. In: Crimi A, Bakas S, Kuijf H, eds. Brainlesion: glioma, multiple sclerosis, stroke and traumatic brain injuries. Cham: Springer International Publishing, 2018: 111–22.

[318] Dvorak P, Bartusek K, Gescheidtova E. Automatic extraction of pathological area in 2D Mr brain scan. in: PIERS proceedings 2014:1885–9.

[319] Dvořák P, Menze B. Local structure prediction with convolutional neural networks for multimodal brain tumor segmentation. In: Menze B, Langs G, Montillo A, eds. Medical computer vision: algorithms for big data. Cham: Springer International Publishing, 2016: 59–71.

[320] El-Khamy SE, Sadek RA, El-Khoreby MA. An efficient brain mass detection with adaptive clustered based fuzzy C-mean and thresholding. In: 2015 IEEE International Conference on Signal and Image Processing Applications (ICSIPA) 2015:429–33.

[321] Elliott C, Francis SJ, Arnold DL. Bayesian classification of multiple sclerosis lesions in longitudinal MRI using subtraction images. In: Jiang T, Navab N, Pluim JPW, eds. Medical image computing and computer-assisted intervention – MICCAI. Berlin, Heidelberg: Springer, 2010: 290–7. 10.1007/978-3-642-15745-5_36 20879327

[322] Ellwaa A, Hussein A, AlNaggar E. Brain tumor Segmantation using random forest trained on Iteratively selected patients, 2016: 129–37. https://www.springerprofessional.de/en/brain-tumor-segmantation-using-random-forest-trained-on-iterativ/12216552

[323] Feng C, Zhao D, Huang M. Segmentation of ischemic stroke lesions in multi-spectral MR images using weighting suppressed FCM and three phase level set. In: Crimi A, Menze B, Maier O, eds. Brainlesion: glioma, multiple sclerosis, stroke and traumatic brain injuries. Cham: Springer International Publishing, 2016: 233–45.

[324] Ferrari RJ, XW Md, YZ Md. Segmentation of multiple sclerosis lesions using support vector machines. In: medical imaging 2003: image processing. International Society for optics and Photonics 2003:16–26.

[325] Festa J, Pereira S, Mariz J. Automatic brain tumor segmentation of multi-sequence mr images using random decision forests. In: Proceedings of the MICCAI grand challenge on MR brain image segmentation (MRBrainS’13) 2013.

[326] CÖ F, Eroğul O, Telatar Z. Fully automated brain tumor segmentation and volume estimation based on symmetry analysis in MR images. Singapore: Springer, 2017: 53–60.

[327] Folgoc LL, Nori AV, Alvarez-Valle J. Segmentation of brain tumors via cascades of lifted decision forests. In: proceedings MICCAI-BRATS workshop 2016.

[328] Folgoc L, Nori A, Ancha S. Lifted auto-context forests for brain tumour segmentation. 2016:171–83.

[329] García-Lorenzo D, Lecoeur J, Arnold DL. Multiple sclerosis lesion segmentation using an automatic multimodal graph cuts. In: Yang G-Z, Hawkes D, Rueckert D, eds. Medical image computing and computer-assisted intervention – MICCAI. Berlin, Heidelberg: Springer, 2009: 584–91. 10.1007/978-3-642-04271-3_71 20426159

[330] García-Lorenzo D, Prima S, Morrissey S. A robust Expectation-Maximization algorithm for multiple sclerosis lesion segmentation. In: proceeding of MICCAI workshop 2008.

[331] Geremia E, Menze BH, Ayache N. Spatial decision forests for glioma segmentation in multi-channel MR images, 2012. Available: https://hal.inria.fr/hal-00813827 [Accessed 15 Nov 2020]. 10.1007/978-3-642-15705-9_14 20879221

[332] Goel S, Sehgal A, Mangipudi P. Brain tumor segmentation in multispectral MR images. In: 2017 4th International Conference on signal processing and integrated networks (SPIN) 2017.

[333] Gondra I, Cabria I. Automated segmentation of brain tumors in MRI using potential field clustering. In: IEEE EUROCON 2015 - International Conference on Computer as a Tool (EUROCON) 2015:1–6.

[334] Götz M, Weber C, Blöcher J. Extremely randomized trees based brain tumor segmentation. In: Proceeding of BRATS challenge-MICCAI 2014:6–11.

[335] Hadjiprocopis A, Tofts P. An automatic lesion segmentation method for fast spin echo magnetic resonance images using an ensemble of neural networks. In: 2003 IEEE XIII workshop on neural networks for signal processing (IEEE cat. No.03TH8718) 2003:709–18.

[336] Haeck T, Maes F, Suetens P. Automated model-based segmentation of brain tumors in MR images. In: proceedings BraTS challenge 2015:25–8.

[337] Haeck T, Maes F, Suetens P. ISLES challenge 2015: automated model-based segmentation of ischemic stroke in MR images. In: Crimi A, Menze B, Maier O, eds. Brainlesion: glioma, multiple sclerosis, stroke and traumatic brain injuries. Cham: Springer International Publishing, 2016: 246–53.

[338] Harmouche R, Collins L, Arnold D, et al. Lesion classification modeling regional and local spatial information. In: 18th International Conference on Pattern Recognition (ICPR’06) 2006:984–7.

[339] Havaei M, Dutil F, Pal C. A convolutional neural network approach to brain tumor segmentation. In: Crimi A, Menze B, Maier O, eds. Brainlesion: glioma, multiple sclerosis, stroke and traumatic brain injuries. Cham: Springer International Publishing, 2016: 195–208.

[340] Hevia-Montiel N, Jiménez-Alaniz JR, Medina-Bañuelos V, et al. Robust nonparametric segmentation of infarct lesion from diffusion-weighted MR images. Annu Int Conf IEEE Eng Med Biol Soc 2007;2007:2102–5. 10.1109/IEMBS.2007.4352736 18002402

[341] Hooda H, Verma OP, Singhal T. Brain tumor segmentation: a performance analysis using k-means, fuzzy C-Means and region growing algorithm. in: 2014 IEEE International Conference on advanced communications, control and computing technologies 2014:1621–6.

[342] Hu Y, Xia Y. 3D deep neural network-based brain tumor segmentation using multimodality magnetic resonance sequences. In: Crimi A, Bakas S, Kuijf H, eds. Brainlesion: glioma, multiple sclerosis, stroke and traumatic brain injuries. Cham: Springer International Publishing, 2018: 423–34.

[343] Huang J, Yang F, Chen W. Brain tumor segmentation based on texture, intensity, and edge. In: Long M, ed. World congress on medical physics and biomedical engineering may 26-31, 2012, Beijing, China. Berlin, Heidelberg: Springer, 2013: 991–4.

[344] Hussain S, Anwar SM, Majid M. Brain tumor segmentation using cascaded deep convolutional neural network. In: 2017 39th annual International Conference of the IEEE engineering in medicine and biology Society (EMBC) 2017:1998–2001. 10.1109/EMBC.2017.8037243 29060287

[345] Iftekharuddin KM, Islam MA, Shaik J. Automatic brain tumor detection in MRI: methodology and statistical validation. in: medical imaging 2005: image processing. International Society for optics and Photonics 2005:2012–22.

[346] Ilunga-Mbuyamba E, Avina-Cervantes JG, Lindner D. Automatic brain tumor tissue detection based on hierarchical centroid shape descriptor in Tl-weighted MR images. in: 2016 International Conference on electronics, communications and computers (CONIELECOMP) 2016:62–7.

[347] Jain S, Ribbens A, Sima DM. Unsupervised framework for consistent longitudinal MS lesion segmentation. In: Müller H, Kelm BM, Arbel T, eds. Medical computer vision and Bayesian and graphical models for biomedical imaging. Cham: Springer International Publishing, 2017: 208–19.

[348] Jerman T, Galimzianova A, Pernuš F. Combining unsupervised and supervised methods for lesion segmentation. In: Crimi A, Menze B, Maier O, eds. Brainlesion: glioma, multiple sclerosis, stroke and traumatic brain injuries. Cham: Springer International Publishing, 2016: 45–56.

[349] Jesson A, Arbel T. Hierarchical MRF and random forest segmentation of MS lesions and healthy tissue in brain MRI. in: proceedings of the 2015 longitudinal multiple sclerosis lesion segmentation challenge 2015;3.

[350] Jiang Y, Hou J, Xiao X. A brain tumor segmentation new method based on statistical thresholding and multiscale CNN. In: Huang D-S, Gromiha MM, Han K, eds. Intelligent computing methodologies. Cham: Springer International Publishing, 2018: 235–45.

[351] Jin D, Xu Z, Harrison AP. White matter hyperintensity segmentation from T1 and FLAIR images using fully convolutional neural networks enhanced with residual connections. In: 2018 IEEE 15th International Symposium on biomedical imaging (ISBI 2018) 2018:1060–4.

[352] Jog A, Carass A, Pham DL. Multi-Output decision trees for lesion segmentation in multiple sclerosis. In: proceedings of SPIE the International Society for optical engineering 2015. 10.1117/12.2082157 PMC5041594 27695155

[353] Kanas VG, Zacharaki EI, Dermatas E. Combining outlier detection with random walker for automatic brain tumor segmentation. In: Iliadis L, Maglogiannis I, Papadopoulos H, eds. Artificial intelligence applications and innovations. Berlin, Heidelberg: Springer, 2012: 26–35.

[354] Kapás Z, Lefkovits L, Iclănzan D. Automatic brain tumor segmentation in multispectral mri volumes using a random forest approach. In: Paul M, Hitoshi C, Huang Q, eds. Image and video technology. Cham: Springer International Publishing, 2018: 137–49.

[355] Karimaghaloo Z, Arnold DL, Collins DL. Hierarchical conditional random fields for detection of gad-enhancing lesions in multiple sclerosis. In: Ayache N, Delingette H, Golland P, eds. Medical image computing and computer-assisted intervention – MICCAI 2012. Berlin, Heidelberg: Springer, 2012: 379–86. 10.1007/978-3-642-33418-4_47 23286071

[356] Karimaghaloo Z, Rivaz H, Arnold DL. Adaptive voxel, texture and temporal conditional random fields for detection of gad-enhancing multiple sclerosis lesions in brain MRI. In: Mori K, Sakuma I, Sato Y, eds. Medical image computing and computer-assisted intervention – MICCAI 2013. Berlin, Heidelberg: Springer, 2013: 543–50. 10.1007/978-3-642-40760-4_68 24505804

[357] Karpate Y, Commowick O, Barillot C. Probabilistic one class learning for automatic detection of multiple sclerosis lesions. in: 2015 IEEE 12th International Symposium on biomedical imaging (ISBI) 2015:486–9.

[358] Khademi A, Moody AR. Multiscale partial volume estimation for segmentation of white matter lesions using FLAIR MRI. In: 2015 IEEE 12th International Symposium on biomedical imaging (ISBI). 2015:568–71.

[359] Khotanlou H, Colliot O, Bloch I. Automatic brain tumor segmentation using symmetry analysis and deformable models. In: Advances in pattern recognition. world scientific 2006:198–202.

[360] Knight J, Khademi A. MS lesion segmentation using FLAIR MRI only. In: Proceedings of the 1st MICCAI challenge on multiple sclerosis lesions segmentation challenge using a data management and processing infrastructure-MICCAI-MSSEG 2016:21–8.

[361] Kroon D-J, van Oort E, Slump K. Multiple sclerosis detection in multispectral magnetic resonance images with principal components analysis. In: Grand challenge work.: Mult. Scler. lesion Segm. challenge, 2008: 1–14. http://hdl.handle.net/10380/1441

[362] Kuijf HJ, CMW T, Zaanen LK. The added value of diffusion tensor imaging for automated white matter hyperintensity segmentation. In: O’Donnell L, Nedjati-Gilani G, Rathi Y, eds. Computational diffusion MRI. Cham: Springer International Publishing, 2014: 45–53.

[363] Kumar SVA, Harish BS, Aradhya VNM. A picture fuzzy clustering approach for brain tumor segmentation. In: 2016 second International Conference on cognitive computing and information processing (CCIP) 2016:1–6.

[364] Kwon D, Akbari H, Da X. Multimodal brain tumor image segmentation using GLISTR. In: MICCAI brain tumor segmentation (BraTS) challenge manuscripts 2014.

[365] Latif G, Butt MM, Khan AH. Automatic multimodal brain image classification using MLP and 3D glioma tumor reconstruction. In: 2017 9th IEEE-GCC conference and exhibition (GCCCE) 2017:1–9.

[366] Lefkovits L, Lefkovits S, Szilágyi L. Brain tumor segmentation with optimized random forest. In: Crimi A, Menze B, Maier O, eds. Brainlesion: glioma, multiple sclerosis, stroke and traumatic brain injuries. Cham: Springer International Publishing, 2016: 88–99.

[367] Li W, Tian J. Automatic segmentation of brain infarction in diffusion-weighted MR images. In: medical imaging 2003: image processing. International Society for optics and Photonics 2003:1531–42.

[368] Li W, Tian J, Dai J. Automatic segmentation of cerebral ischemic lesions from diffusion tensor MR images. In: medical imaging 2004: image processing. International Society for optics and Photonics 2004:1640–9.

[369] Li Y, Dou Q, Yu J. Automatic brain tumor segmentation from MR images via a multimodal sparse coding based probabilistic model. In: 2015 International workshop on pattern recognition in neuroimaging 2015:41–4.

[370] Liu J, Smith CD, Chebrolu H. Automatic multiple sclerosis detection based on integrated square estimation. in: 2009 IEEE computer Society conference on computer vision and pattern recognition workshops 2009:31–8.

[371] Liu R, Cheng J, Zhu X. Multi-modal brain tumor segmentation based on self-organizing active contour model. In: Tan T, Li X, Chen X, eds. Pattern recognition. Singapore: Springer, 2016: 486–98.

[372] López-Zorrilla A, de Velasco-Vázquez M, Serradilla-Casado O. Brain white matter lesion segmentation with 2D/3D CNN. In: Ferrández Vicente JM, Álvarez-Sánchez JR, de la Paz López F, eds. Natural and artificial computation for biomedicine and neuroscience. Cham: Springer International Publishing, 2017: 394–403.

[373] Lyksborg M, Puonti O, Agn M. An ensemble of 2D convolutional neural networks for tumor segmentation. In: Paulsen RR, Pedersen KS, eds. Image analysis. Cham: Springer International Publishing, 2015: 201–11.

[374] Mahmood Q, Basit A. Automatic ischemic stroke lesion segmentation in multi-spectral MRI images using random forests classifier. In: Crimi A, Menze B, Maier O, eds. Brainlesion: glioma, multiple sclerosis, stroke and traumatic brain injuries. Cham: Springer International Publishing, 2016: 266–74.

[375] Mahmood Q, Basit A. Prediction of ischemic stroke lesion and clinical outcome in multi-modal MRI images using random forests. In: Crimi A, Menze B, Maier O, eds. Brainlesion: glioma, multiple sclerosis, stroke and traumatic brain injuries. Cham: Springer International Publishing, 2016: 244–55.

[376] Maier O, Wilms M, Handels H. Image features for brain lesion segmentation using random forests. In: Crimi A, Menze B, Maier O, eds. 2015 longitudinal multiple sclerosis lesion segmentation challenge. Cham: Springer International Publishing, 2015: 119–30.

[377] McKinley R, Häni L, Wiest R. Segmenting the ischemic penumbra: a decision forest approach with automatic threshold finding. In: Crimi A, Menze B, Maier O, eds. Brainlesion: glioma, multiple sclerosis, stroke and traumatic brain injuries. Cham: Springer International Publishing, 2015: 275–83.

[378] Mechrez R, Goldberger J, Greenspan H. MS lesion segmentation using a multi-channel patch-based approach with spatial consistency. in: medical imaging 2015: image processing. International Society for optics and Photonics 2015.

[379] Mehmood I, Baik R, Baik SW. Automatic segmentation of region of interests in MR images using saliency information and active contours. In: Kim KJ, Chung K-Y, eds. IT convergence and security 2012. Dordrecht: Springer Netherlands, 2012: 537–44.

[380] Meier R, Karamitsou V, Habegger S. Parameter learning for CRF-based tissue segmentation of brain tumors. In: Crimi A, Menze B, Maier O, eds. Brainlesion: glioma, multiple sclerosis, stroke and traumatic brain injuries. Cham: Springer International Publishing, 2016: 156–67.

[381] Meier R, Knecht U, Wiest R. CRF-based brain tumor segmentation: alleviating the shrinking bias. In: Crimi A, Menze B, Maier O, eds. Brainlesion: glioma, multiple sclerosis, stroke and traumatic brain injuries. Cham: Springer International Publishing, 2016: 100–7.

[382] Mengqiao W, Jie Y, Yilei C. The multimodal brain tumor image segmentation based on convolutional neural networks. In: 2017 2nd IEEE International Conference on computational intelligence and applications (ICCIA) 2017:336–9.

[383] Mitra J, Bourgeat P, Fripp J. Classification forests and markov random field to segment chronic ischemic infarcts from multimodal MRI. In: Shen L, Liu T, Yap P-T, eds. Multimodal brain image analysis. Cham: Springer International Publishing, 2013: 107–18.

[384] Morra J, Tu Z, Toga A. Automatic segmentation of MS lesions using a contextual model for the MICCAI grand challenge. in: grand challenge work Mult. Scler. lesion Segm. challenge 2008:1–7.

[385] Mote SR, Baid UR, Talbar SN. Non-Negative matrix factorization and self-organizing map for brain tumor segmentation. in: 2017 International Conference on wireless communications, signal processing and networking (WiSPNET) 2017:1133–7.

[386] Muda AF, Saad NM, Waeleh N. Integration of fuzzy C-Means with correlation template and active contour for brain lesion segmentation in diffusion-weighted MRI. in: 2015 3rd International Conference on artificial intelligence, modelling and simulation (aims) 2015:268–73.

[387] Müller S, Weickert J, Graf N. Automatic brain tumor segmentation with a fast Mumford-Shah algorithm. in: medical imaging 2016: image processing. International Society for optics and Photonics 2016.

[388] Nabizadeh N, Dorodchi M, Kubat M. Automatic tumor lesion detection and segmentation using modified winnow algorithm. In: 2015 IEEE 12th International Symposium on biomedical imaging (ISBI) 2015:71–4.

[389] Oliveira GC, Varoto R JAC. Brain tumor segmentation in magnetic resonance images using genetic algorithm clustering and adaboost classifier, 2020: 77–82. https://www.scitepress.org/PublicationsDetail.aspx?ID=y+ZZXSvPyrc=&t=1

[390] Osman AFI. Automated brain tumor segmentation on magnetic resonance images and patient’s overall survival prediction using support vector machines. In: Crimi A, Bakas S, Kuijf H, eds. Brainlesion: glioma, multiple sclerosis, stroke and traumatic brain injuries. Cham: Springer International Publishing, 2018: 435–49.

[391] Pandian B, Boyle J, Orringer DA. Multimodal tumor segmentation with 3D convolutional neural networks. in: proceedings of the MICCAI challenge on multimodal brain tumor image segmentation (BRATS) 2016.

[392] Parisot S, Duffau H, Chemouny S. Graph-based detection, segmentation characterization of brain tumors In: 2012 IEEE conference on computer vision and pattern recognition 2012:988–95.

[393] Parisot S, Duffau H, Chemouny S. Joint tumor segmentation and dense deformable registration of brain MR images. In: Ayache N, Delingette H, Golland P, eds. Medical image computing and computer-assisted intervention – MICCAI 2012. Berlin, Heidelberg: Springer, 2012: 651–8. 10.1007/978-3-642-33418-4_80 23286104

[394] Buendia P, Taylor T, Ryan M. A grouping artificial immune network for segmentation of tumor images. in: proceedings of the MICCAI challenge on multimodal brain tumor image segmentation (BRATS) 2013;2013.

[395] Pedoia V, Balbi S, Binaghi E. Fully automatic brain tumor segmentation by using competitive EM and graph cut. In: Murino V, Puppo E, eds. Image analysis and processing — ICIAP 2015. Cham: Springer International Publishing, 2015: 568–78.

[396] Pereira S, Oliveira A, Alves V. On hierarchical brain tumor segmentation in MRI using fully convolutional neural networks: a preliminary study. In: 2017 IEEE 5th Portuguese meeting on bioengineering (ENBENG) 2017:1–4.

[397] Pinto A, Pereira S, Correia H. Brain tumour segmentation based on extremely randomized forest with high-level features. in: 2015 37th annual International Conference of the IEEE engineering in medicine and biology Society (EmbC) 2015:3037–40. 10.1109/EMBC.2015.7319032 26736932

[398] Pinto A, Pereira S, Dinis H. Random decision forests for automatic brain tumor segmentation on multi-modal MRI images. In: 2015 IEEE 4th Portuguese meeting on bioengineering (ENBENG) 2015:1–5.

[399] Pourreza R, Zhuge Y, Ning H. Brain tumor segmentation in MRI scans using deeply-supervised neural networks. In: Crimi A, Bakas S, Kuijf H, eds. Brainlesion: glioma, multiple sclerosis, stroke and traumatic brain injuries. Cham: Springer International Publishing, 2018: 320–31.

[400] Puonti O, Van Leemput K. Simultaneous whole-brain segmentation and white matter lesion detection using contrast-adaptive probabilistic models. In: Crimi A, Menze B, Maier O, eds. Brainlesion: glioma, multiple sclerosis, stroke and traumatic brain injuries. Cham: Springer International Publishing, 2016: 9–20.

[401] Rachmadi MF, Valdés-Hernández M del C, Komura T. Automatic irregular texture detection in brain MRI without human supervision. In: Medical image computing and computer assisted intervention – MICCAI 2018. Springer, 2018.

[402] Randhawa RS, Modi A, Jain P. Improving boundary classification for brain tumor segmentation and longitudinal disease progression. In: Crimi A, Menze B, Maier O, eds. Brainlesion: glioma, multiple sclerosis, stroke and traumatic brain injuries. Cham: Springer International Publishing, 2016: 65–74.

[403] Raniga P, Schmitt P, Bourgeat P. Local intensity model: an outlier detection framework with applications to white matter hyperintensity segmentation. In: 2011 IEEE International Symposium on biomedical imaging: from nano to macro 2011:2057–60.

[404] Rao A, Ledig C, Newcombe V. Contusion segmentation from subjects with traumatic brain injury: a random forest framework. In: 2014 IEEE 11th International Symposium on biomedical imaging (ISBI) 2014:333–6.

[405] Rexilius J, Hahn HK, Klein J. Multispectral brain tumor segmentation based on histogram model adaptation. In: medical imaging 2007: computer-aided diagnosis. International Society for optics and Photonics 2007.

[406] Reza S, Linmin P, Iftekharuddin KM. Ischemic stroke lesion segmentation using local gradient and texture features. In: ischemic stroke lesion segmentation 2015.

[407] Rezaei M, Yang H, Meinel C. Deep neural network with l2-norm unit for brain lesions detection. In: Liu D, Xie S, Li Y, eds. Neural information processing. Cham: Springer International Publishing, 2017: 798–807.

[408] Riad MM, Platel B, de Leeuw F-E. Detection of white matter lesions in cerebral small vessel disease 2013;867014.

[409] Rios Piedra EA, Ellingson BM, Taira RK. Brain tumor segmentation by variability characterization of tumor boundaries. In: Crimi A, Menze B, Maier O, eds. Brainlesion: glioma, multiple sclerosis, stroke and traumatic brain injuries. Cham: Springer International Publishing, 2016: 206–16.

[410] Rodrigo F, Graffigna JP, Isoardi R. Segmentation of hyperintense regions applied to multiple sclerosis lesions. In: Braidot A, Hadad A, eds. VI Latin American Congress on Biomedical Engineering CLAIB 2014, Paraná, Argentina 29, 30 & 31 October 2014. Cham: Springer International Publishing, 2015: 425–8.

[411] Roy PK, Bhuiyan A, Janke A. Automated segmentation of white matter lesions using global neighbourhood given contrast feature-based random forest and Markov random field. In: 2014 IEEE International Conference on healthcare informatics 2014:1–6.

[412] Roy S, Maji P. A new post-processing method to detect brain tumor using rough-fuzzy clustering. In: Kryszkiewicz M, Bandyopadhyay S, Rybinski H, eds. Pattern recognition and machine intelligence. Cham: Springer International Publishing, 2015: 407–17.

[413] Doyle S, Reyes M, Dojat M. Fully automatic brain tumor segmentation from multiple MR sequences using hidden Markov fields and variational. in: NCI-MICCAI BraTS, 2013. Available: /paper/Fully-Automatic-Brain-Tumor-Segmentation-from-MR-Menze-Reyes/c3912d865a263e42d51ae28850cd8332cd1612ee [Accessed 16 Nov 2020].

[414] Reza S, Iftekharuddin KM. Improved brain tumor tissue segmentation using texture features. In: Proceedings MICCAI BraTS (brain tumor segmentation challenge) 2014:27–30.

[415] Saha R, Phophalia A, Mitra SK. Brain tumor segmentation from multimodal MR images using rough sets. In: Mukherjee S, Mukherjee S, Mukherjee DP, eds. Computer vision, graphics, and image processing. Cham: Springer International Publishing, 2017: 133–44.

[416] Sankari A, Vigneshwari S. Automatic tumor segmentation using convolutional neural networks. In: 2017 third International Conference on science technology engineering management (ICONSTEM) 2017:268–72.

[417] Schmidt M, Levner I, Greiner R. Segmenting brain tumors using alignment-based features. In: Fourth International Conference on Machine Learning and Applications (ICMLA’05) 2005.

[418] Sehgal A, Goel S, Mangipudi P. Automatic brain tumor segmentation and extraction in MR images. In: 2016 conference on advances in signal processing (CASP) 2016:104–7.

[419] Shah N, Ziauddin S, Shahid AR. Brain tumor segmentation and classification using cascaded random decision forests. In: 2017 14th International Conference on electrical Engineering/Electronics, computer, telecommunications and information technology (ECTI-CON) 2017:718–20.

[420] Shaikh M, Anand G, Acharya G. Brain tumor segmentation using dense fully convolutional neural network. In: Crimi A, Bakas S, Kuijf H, eds. Brainlesion: glioma, multiple sclerosis, stroke and traumatic brain injuries. Cham: Springer International Publishing, 2018: 309–19.

[421] Shen H, Wang R, Zhang J. Multi-task fully convolutional network for brain tumour segmentation. In: Valdés Hernández M, González-Castro V, eds. Medical image understanding and analysis. Cham: Springer International Publishing, 2017: 239–48.

[422] Shivhare SN, Sharma S, Singh N. An efficient brain tumor detection and segmentation in MRI using parameter-free clustering. In: Tanveer M, Pachori RB, eds. Machine intelligence and signal analysis. Singapore: Springer, 2019: 485–95.

[423] Shreyas V, Pankajakshan V. A deep learning architecture for brain tumor segmentation in MRI images. In: 2017 IEEE 19th International workshop on multimedia signal processing (MMSP) 2017:1–6.

[424] Soltaninejad M, Zhang L, Lambrou T. MRI brain tumor segmentation and patient survival prediction using random forests and fully convolutional networks. In: Crimi A, Bakas S, Kuijf H, eds. Brainlesion: glioma, multiple sclerosis, stroke and traumatic brain injuries. Cham: Springer International Publishing, 2018: 204–15.

[425] Song B, Chou C-R, Chen X. Anatomy-guided brain tumor segmentation and classification. In: Crimi A, Menze B, Maier O, eds. Brainlesion: glioma, multiple sclerosis, stroke and traumatic brain injuries. Cham: Springer International Publishing, 2016: 162–70.

[426] Souplet J-C, Lebrun-Frenay C, Ayache N. An automatic segmentation of T2-FLAIR multiple sclerosis lesions. in: MICCAI-Multiple sclerosis lesion segmentation challenge workshop 2008.

[427] Srivastava S, Maes F, Vandermeulen D. Feature-based statistical analysis of structural MR data for automatic detection of focal cortical dysplastic (FCD) lesions. In: 2004 2nd IEEE International Symposium on biomedical imaging: nano to macro (IEEE cat No. 04EX821) 2004:1127–30.

[428] Bauer S, Fejes T, Slotboom J. Segmentation of brain tumor images based on integrated hierarchical classification and regularization 2012.

[429] Subbanna N, Precup D, Arbel T. Iterative multilevel MRF Leveraging context and Voxel information for brain tumour segmentation in MRI. In: 2014 IEEE conference on computer vision and pattern recognition 2014:400–5.

[430] Subbanna NK, Precup D, Collins DL. Hierarchical probabilistic gabor and MRF segmentation of brain tumours in MRI volumes. In: Mori K, Sakuma I, Sato Y, eds. Medical image computing and computer-assisted intervention – MICCAI 2013. Berlin, Heidelberg: Springer, 2013: 751–8. 10.1007/978-3-642-40811-3_94 24505735

[431] Subbanna N, Precup D, Arnold D, et al. Image: iterative multilevel probabilistic graphical model for detection and segmentation of multiple sclerosis lesions in brain MRI. Inf Process Med Imaging 2015;24:514–26. 10.1007/978-3-319-19992-4_40 26221699

[432] Subbanna N, Shah M, Francis S. MS lesion segmentation using Markov random fields. in: proceedings of international Conference on medical image computing and computer assisted intervention, London, UK 2009.

[433] Szilágyi L, Lefkovits L, Iantovics B. Automatic brain tumor segmentation in multispectral MRI volumetric records. In: Arik S, Huang T, Lai WK, eds. Neural information processing. Cham: Springer International Publishing, 2015: 174–81.

[434] Hsu W. Brain tumor segmentation using deep convolutional neural network. In: Proceedings of BRATS-MICCAI 2016.

[435] Tang H, Lu H, Liu W. Tumor segmentation from single contrast MR images of human brain. in: 2015 IEEE 12th International Symposium on biomedical imaging (ISBI) 2015:46–9.

[436] Taylor T, John N, Buendia P. Map-reduce enabled hidden Markov models for high throughput multimodal brain tumor segmentation. In: Proceedings of the MICCAI Challenge on Multimodal Brain Tumor Image Segmentation (BRATS) 2013;2013.

[437] Uchiyama Y, Kunieda T, Hara T. Automatic segmentation of different-sized leukoaraiosis regions in brain Mr images. in: medical imaging 2008: computer-aided diagnosis. International Society for optics and Photonics 2008;69151S.

[438] Urban G, Bendszus M, Hamprecht F. Multi-modal brain tumor segmentation using deep Convolutional neural networks. in: MICCAI BraTS (brain tumor segmentation) challenge 2014.

[439] Vaidhya K, Thirunavukkarasu S, Alex V. Multi-modal brain tumor segmentation using stacked denoising autoencoders. In: Crimi A, Menze B, Maier O, eds. Brainlesion: glioma, multiple sclerosis, stroke and traumatic brain injuries. Cham: Springer International Publishing, 2016: 181–94.

[440] Vaidya S, Chunduru A, Muthuganapathy R. Longitudinal multiple sclerosis lesion segmentation using 3D convolutional neural networks. In: Proceedings of the 2015 longitudinal multiple sclerosis lesion segmentation challenge 2015:1–2.

[441] Vaishnavee KB, Amshakala K. An automated MRI brain image segmentation and tumor detection using SOM-clustering and Proximal Support Vector Machine classifier. In: ICETECH, ed. 2015 IEEE International Conference on Engineering and Technology, 2015: 1–6.

[442] Wang G, Li W, Ourselin S. Automatic brain tumor segmentation using cascaded anisotropic convolutional neural networks. In: Crimi A, Bakas S, Kuijf H, eds. Brainlesion: glioma, multiple sclerosis, stroke and traumatic brain injuries. Cham: Springer International Publishing, 2018: 178–90.

[443] Wang T, Cheng I, Basu A. Fully automatic brain tumor segmentation using a normalized Gaussian Bayesian classifier and 3D fluid vector flow. in: 2010 IEEE International Conference on image processing 2010:2553–6.

[444] Wang Y, Katsaggelos AK, Wang X. A deep symmetry convnet for stroke lesion segmentation. In: ICIP, ed. 2016 IEEE International Conference on Image Processing, 2016: 111–5.

[445] Weiss N, Rueckert D, Rao A. Multiple sclerosis lesion segmentation using dictionary learning and sparse coding. In: Mori K, Sakuma I, Sato Y, eds. Medical image computing and computer-assisted intervention – MICCAI 2013. Berlin, Heidelberg: Springer, 2013: 735–42. 10.1007/978-3-642-40811-3_92 24505733

[446] Xu Y, Géraud T, É P. White matter hyperintensities segmentation in a few seconds using fully convolutional network and transfer learning. In: Crimi A, Bakas S, Kuijf H, eds. Brainlesion: glioma, multiple sclerosis, stroke and traumatic brain injuries. Cham: Springer International Publishing, 2018: 501–14.

[447] Chen X, Nguyen BP, Chui C-K. Automated brain tumor segmentation using kernel dictionary learning and superpixel-level features. In: SMC, ed. 2016 IEEE International Conference on Systems, Man, and Cybernetics, 2016: 002547–52.

[448] Xuan X, Liao Q. Statistical structure analysis in MRI brain tumor segmentation. In: ICIG, ed. Fourth International Conference on Image and Graphics. 2007, 2007: 421–6.

[449] Yoo Y, Tang LW, Brosch T. Deep learning of brain lesion patterns for predicting future disease activity in patients with early symptoms of multiple sclerosis. In: Carneiro G, Mateus D, Peter L, eds. Deep learning and data labeling for medical applications. Cham: Springer International Publishing, 2016: 86–94.

[450] C-P Y, Ruppert G, Nguyen D. Statistical asymmetry-based brain tumor segmentation from 3D MR images, 2012.

[451] Yu R, Xiao L, Wei Z. Automatic segmentation of white matter lesions using SVM and RSF model in multi-channel MRI. In: Zhang Y-J, ed. Image and graphics. Cham: Springer International Publishing, 2015: 654–63.

[452] Zabir I, Paul S, Rayhan MA. Automatic brain tumor detection and segmentation from multi-modal MRI images based on region growing and level set evolution. In: WIECON-ECE, ed. 2015 IEEE International WIE Conference on Electrical and Computer Engineering, 2015: 503–6.

[453] Zacharaki EI, Erus G, Bezerianos A. Fuzzy multi-channel clustering with individualized spatial priors for segmenting brain lesions and infarcts. In: Iliadis L, Maglogiannis I, Papadopoulos H, eds. Artificial intelligence applications and innovations. Berlin, Heidelberg: Springer, 2012: 76–85.

[454] Zacharaki EI, Kanterakis S, Bryan RN. Measuring brain lesion progression with a supervised tissue classification system. In: Metaxas D, Axel L, Fichtinger G, et al, eds. Medical image computing and computer assisted intervention – MICCAI 2008. Berlin, Heidelberg: Springer, 2008: 620–7. 10.1007/978-3-540-85988-8_74 18979798

[455] Zhan T, Gu S, Jiang L. A novelnovel brainbrain tumortumor segmentationsegmentation methodmethod for multimulti-modalitymodality humanhuman brainbrain MRIs 2015.

[456] Zhao L, Sarikaya D, Corso JJ. Automatic brain tumor segmentation with MRF on supervoxels. in. multimodal brain tumor segmentation 2013;51.

[457] Xiao Z, Huang R, Ding Y. A deep learning-based segmentation method for brain tumor in MR images. In: 2016 IEEE 6th International Conference on computational advances in BIO and medical sciences (ICCABS) 2016:1–6.

[458] Lao Z, Shen D, Jawad A. Automated segmentation of white matter lesions in 3D brain MR images, using multivariate pattern classification. In: 3rd IEEE International Symposium on biomedical imaging: nano to macro, 2006 2006:307–10.

[459] Zikic D, Glocker B, Konukoglu E. Context-Sensitive classification forests for segmentation of brain tumor tissues, 2012.

[460] Zikic D, Glocker B, Konukoglu E. Decision forests for tissue-specific segmentation of high-grade gliomas in multi-channel MR. In: Ayache N, Delingette H, Golland P, eds. Medical image computing and computer-assisted intervention – MICCAI 2012. Berlin, Heidelberg: Springer, 2012: 369–76. 10.1007/978-3-642-33454-2_46 23286152

[461] Yi D, Zhou M, Chen Z. 3-D convolutional neural networks for glioblastoma segmentation. Available: http://arxiv.org/abs/1611.04534

[462] Beers A, Chang K, Brown J. Sequential 3D U-Nets for biologically-informed brain tumor segmentation, 2017. Available: http://arxiv.org/abs/1709.02967

[463] Styner M, Lee J, Chin B. 3D segmentation in the clinic: a grand challenge II: MS lesion segmentation. The MIDAS Journal 2008;638.

[464] Menze BH, Jakab A, Bauer S, et al. The multimodal brain tumor image segmentation benchmark (BRATS). IEEE Trans Med Imaging 2015;34:1993–2024. 10.1109/TMI.2014.2377694 25494501 PMC4833122

[465] Carass A, Roy S, Jog A, et al. Longitudinal multiple sclerosis lesion segmentation: resource and challenge. Neuroimage 2017;148:77–102. 10.1016/j.neuroimage.2016.12.064 28087490 PMC5344762

[466] Maier O, Menze BH, von der Gablentz J, et al. ISLES 2015 - A public evaluation benchmark for ischemic stroke lesion segmentation from multispectral MRI. Med Image Anal 2017;35:250–69. 10.1016/j.media.2016.07.009 27475911 PMC5099118

[467] Bø HK, Solheim O, Jakola AS, et al. Intra-rater variability in low-grade glioma segmentation. J Neurooncol 2017;131:393–402. 10.1007/s11060-016-2312-9 27837437

[468] Zijdenbos AP, Dawant BM, Margolin RA, et al. Morphometric analysis of white matter lesions in MR images: method and validation. IEEE Trans Med Imaging 1994;13:716–24. 10.1109/42.363096 18218550

[469] Gibson E, Hu Y, Huisman HJ, et al. Designing image segmentation studies: statistical power, sample size and reference standard quality. Med Image Anal 2017;42:44–59. 10.1016/j.media.2017.07.004 28772163 PMC5666910

[470] Recent advances in MRI technology. imaging technology news, 2016. Available: https://www.itnonline.com/article/recent-advances-mri-technology [Accessed 23 Nov 2020].

[471] OECD STAT. Available: https://stats.oecd.org/Index.aspx?ThemeTreeId=9 [Accessed 23 Nov 2020].

[472] FDA clears first 7T MRI system, Magnetom TERRA. imaging technology news, 2017. Available: https://www.itnonline.com/content/fda-clears-first-7t-mri-system-magnetom-terra [Accessed 23 Nov 2020].

[473] Maier-Hein L, Eisenmann M, Reinke A, et al. Why rankings of biomedical image analysis competitions should be interpreted with care. Nat Commun 2018;9:5217. 10.1038/s41467-018-07619-7 30523263 PMC6284017

[474] Jorritsma W, Cnossen F, van Ooijen PMA. Improving the radiologist-CAD interaction: designing for appropriate trust. Clin Radiol 2015;70:115–22. 10.1016/j.crad.2014.09.017 25459198

[475] Gryska E, Cerna K, Heckemann RA. Increasing trust through the design of algorithm-based lesion segmentation support systems. In: iConference 2020 proceedings. iSchools, 2020. Available: https://www.ideals.illinois.edu/handle/2142/106560 [Accessed 23 Nov 2020].

[476] Sled JG, Zijdenbos AP, Evans AC. A nonparametric method for automatic correction of intensity nonuniformity in MRI data. IEEE Trans Med Imaging 1998;17:87–97. 10.1109/42.668698 9617910

[477] Tustison NJ, Avants BB, Cook PA, et al. N4ITK: improved N3 bias correction. IEEE Trans Med Imaging 2010;29:1310–20. 10.1109/TMI.2010.2046908 20378467 PMC3071855

[478] Smith SM. Fast robust automated brain extraction. Hum Brain Mapp 2002;17:143–55. 10.1002/hbm.10062 12391568 PMC6871816

[479] Penny W, Friston K, Ashburner J. Statistical parametric mapping: the analysis of functional brain. 1st edn. Elsevier, 2006. https://www.elsevier.com/books/statistical-parametric-mapping-the-analysis-of-functional-brain-images/penny/978-0-12-372560-8

[480] Smith SM, Jenkinson M, Woolrich MW, et al. Advances in functional and structural MR image analysis and implementation as fsl. Neuroimage 2004;23:S208–19. 10.1016/j.neuroimage.2004.07.051 15501092

