# Supplementary material for: Automatic brain lesion segmentation on standard magnetic resonance images: a scoping review
Source: BMJ Open. 2021 Jan 28;11(1):e042660. doi: 10.1136/bmjopen-2020-042660 (PMC7849889; doi:10.1136/bmjopen-2020-042660)
Supplement: Supplementary data [file bmjopen-2020-042660supp001.pdf]

Bibliographic data					General comments	Segmentation								Study cohort					Validation		
**Item type**	**Publication year**	**Author(s)**	**Title**	**Publication Title**		**Multi-sequence input**	**Input sequence(s):**	**Algorithms and computational theories**	**Preprocessing procedures**	**Hardware**	**Segmentation time**	**Lesion classification used**	**Software made available by authors**	**Lesion type**	**Diagnosed conditions**	**Sample size**	**Sample source corrected**	**Multiscanner images**	**Reference segmentation**	**Decision (if multpiple raters and specified)**	**Evaluation measures**
C – conference paper<br>J – journal article						**y - yes<br>n - no**	CBF – Cerebral blood flow<br>DIR – Double inversion recovery<br>DSC – Dynamic susceptibility contrast<br>DTI – Diffusion tensor imaging<br>DWI – Diffusion weighted imaging<br>FA – Fractional anisotropy<br>FLAIR – Fluid-attenuated inversion recovery<br>FSE – Fast spin echo<br>MPR/MPRAGE – Magnetization Prepared Rapid Acquisition Gradient Echo<br>PD – Proton density<br>PWI – Perfusion weighted imaging<br>(R)CBV – (Relative) Cerebral blood volume<br>SD – Spin density weighted<br>T1c – T1 weighted with contrast<br>T1 – T1 weighted<br>T2 – T2 weighted<br>Tmax – Time to maximum<br>TTP – Time to peak		0u1v – 0 mean unit variance<br>ADF – Anidotropic diffusion filtering<br>BE – Brain extraction<br>BFC – Bias field(field inhomogeneity) correction<br>EM – Expectation maximization<br>IN – Intensity normalization<br>M(N)I – Mutual (normalized) information<br>N3 – Sled et al. 1998<br>N4 – Tustison et al. 2010<br>n/s – Not specified<br>REG – Image (co-) registration<br>WM – White matter			**y - yes<br>n - no**	**y - yes<br>n - no**	AD – Alzheimer's disease BT – Brain tumour CADASIL - Cerebral autosomal dominant arteriopathy with subcortical infarcts and leukoencephalopathy CI – Cognitive impairment CIS – Clinically isolatd syndrome CP – Cerebral palsy D - Dementia DM(2) – Diabetes mellitus (type II) EP – Epilepsy FCD – Focal cortical displasia GB(M) – Glioblastoma (multiforme) G – glioma HEM – Hemorrhage or bleeding HGG - high grade glioma HT – hypertension IFL – ischemic focal lesions LBD – Lewy body dementia LGG - low grade glioma MCI - minimal cognitive impairement MET – Metastasis MS – Multiple sclerosis lesions NEC – Necrosis (RR)MS - (Relapsing-remitting) multiple sclerosis SLE - systemic lupus erythematosus S – Stroke SVD – Small vessel disease TBI – Traumatic brain Injury T - Tumours VaD – Vascular disease WML – White matter lesions (other than MS lesions)			**1 – Publicably available data with reference segmentation (source)**<br><br>**2 – Publicably available data without reference segmentation (source)**<br><br>**3 – Non-public data sources (source)**	**y - yes<br>n - no**	**Procedure, number of raters**		Acc - Accuracy<br>AD - Average distance<br>avg - Average<br>DE - Detection error<br>DER - Detection error rate<br>DSC - Dice similarity coefficient<br>F1 - F1 score<br>FN - False negative<br>FNR - False negative ratio<br>FP - False positive<br>FPR - False positive rate<br>(F)ROC - (Free-response Receiver) operator curve<br>Hdff - Hausdorff distance<br>ICC - Inter-class correlation<br>Jcc - Jaccard coefficient<br>Kappa - Kappa coefficient<br>NPV - Negative predictive value<br>OER - Outline error rate<br>PPV - Positive predictive value<br>sd - Standard deviation<br>SD - Surface distance ??<br>Sen, TPR, Overlap fraction - Sensitivity<br>SI - Similarity index<br>Spe - Specificity<br>SSD - Symmetric SD<br>TI - Tanimoto index<br>TP - True positive<br>VC - Volume correlation<br>VD - Volume difference<br>VE - Volume error<br>VO - Volume overlap<br>VR - Regression analysis of volume
J	2014	M. Cabezas et al.	BOOST: A supervised approach for multiple sclerosis lesion segmentation	Journal of Neuroscience Methods		y	T1, T2, PD, FLAIR	Boosting	BE:BET; BFC:N4; denoising:ADF; IN:histogram matching	n/a	n/a	n	n	MS	-	45	3	y	Semiautomatic		DSC; avgSD
J	2014	S. Parisot et al.	Concurrent tumor segmentation and registration with uncertainty-based sparse non-uniform graphs	Medical Image Analysis	Inter-rater agreement evaluated	y	T2, FLAIR	Markov Random Field	BE; REG:rigid; IN:matching median and interquartile range to reference	n/a	~5 min	n	n	BT	LGG	110 (27 segmented); 10	1 (BraTS'12); 3	y	Manual, 2		DSC; FPR; TPR; MAD
J	2013	M. D. Steenwijk et al.	Accurate white matter lesion segmentation by k nearest neighbor classification with tissue type priors (kNN-TTPs)	NeuroImage: Clinical	Inter-rater agreement evaluated	y	T1, FLAIR	K-Nearest Neighbours	BE:BET; BFC:N3	n/a	n/a	n	n	WML	MS; HT	20; 20	3	y	Manual, 3	consensus	DSC; ICC; Sen; OER; DER
J	2018	D. S. Meier et al.	Dual-Sensitivity Multiple Sclerosis Lesion and CSF Segmentation for Multichannel 3T Brain MRI	Journal of Neuroimaging	Scan-rescan reproducibility	y	T1, T2, FLAIR	Other	BFC:N4; REG:rigid, affine + Bspline(BRAINS/ITK); BE:BET; tissue maps:Freesurfer; IN:histogram matching	n/a	n/a	n	n	MS	MS	29; 13x2	3	n	Manual, 2	supervision	DSC; Jcc; Hdff; Spe; PPV; Sen; ICC; Bland-Altman
J	2013	A. Sanjuán et al.	Automated identification of brain tumors from single MR images based on segmentation with refined patient-specific priors	Front. Neurosci.	Inter-rater agreement evaluated	n	T1	Previously proposed	n/a	n/a	n/a	n	n	BT	T	18 + 64 healthy	3	n	Manual, 2		DSC; ROC
C	2014	S. Roy et al.	Example based lesion segmentation	Proceedings of SPIE - The International Society for Optical Engineering		y	T1, FLAIR	Previously proposed	BE; IN:mean WM intensity=1	n/a	n/a	n	n	WML	MS	47	3 (described in Shee et al. 2009)	n	Manual		DSC;VC; Sen; Spe
J	2016	A. Galimzianova et al.	Stratified mixture modeling for segmentation of white-matter lesions in brain MR images	NeuroImage	Manual corrections of preprocessing (brain extraction)	y	T1, T2, FLAIR	Comparison	BE:Iglesias; REG:rigid; BFC:N4; downsampling	n/a	n/a	n	n	MS	MS	30	3	n	Manual, 2	consensus	DSC
C	2015	S. Roy and P. Maji	A New Post-processing Method to Detect Brain Tumor Using Rough-Fuzzy Clustering	Pattern Recognition and Machine Intelligence		n/a		Fuzzy C-Means, Region Growing	n/a	n/a	n/a	n	n	BT	-	10	1 (BraTS'12)	y	-		DSC; other
J	2011	S. Klöppel et al.	A comparison of different automated methods for the detection of white matter lesions in MRI data	NeuroImage	Manual corrections of preprocessing	y	T1, FLAIR	Comparison	REG:MI; tissue maps, spatial normalization, BFC:SPM8; IN:0 median + 1 interquartile interval; resampling	n/a	few sec to 5 hrs	n	n	WML	MCI, D	20	3	n	Manual, 1&2		PPV; Sen; ROC; DSC
C	2018	A. Bougacha et al.	Comparative study of supervised and unsupervised classification methods: Application to automatic MRI glioma brain tumors segmentation	[illegible] International Conference on Advanced Technologies for Signal and Image Processing (ATSIP)		y	T1, T1c, T2, FLAIR	Comparison	n/a	n/a	n/a	n	n	BT	LGG; HGG	273	1 (BraTS'15)	y	-		DSC; Jcc
J	2005	S. Srivastava et al.	Feature-based statistical analysis of structural MR data for automatic detection of focal cortical dysplastic lesions	NeuroImage		n	T1	Previously proposed	REG:affine; tissue maps w/ non-rigid registration; IN	n/a	n/a	n	n	FCD	FCD	17 + 64 controls	3	y	Manual		VO
J	2013	S. Datta and P. A. Narayana	A comprehensive approach to the segmentation of multichannel three-dimensional MR brain images in multiple sclerosis	NeuroImage: Clinical		y	T1, T2, FLAIR	Other	REG:rigid (SPM2); BE:thresholding; BFC:SPM2 + ADF; IN:histogram matching	n/a	n/a	n	n	MS	-	60; 50	1 (MS GC MICCAI'08); 3	y	Segmentation correction		VD; avgSD; TPR; FPR; Bland-Altman; VC
J	2016	E. Roura et al.	Automated Detection of Lupus White Matter Lesions in MRI	Front. Neuroinform	Integrated preprocessing	y	T1, FLAIR	Thresholding	REG:SPM; BE:SPM; tissue masks:SPM; denoising:ADF; BFC:EM-based	n/a	n/a	n	y	WML	SLE	20	3	n	Manual, 1		DSC; PPV; TPR; VC; Bland-Altman; ROC
J	2018	W. Chen et al.	Computer-Aided Grading of Gliomas Combining Automatic Segmentation and Radiomics	International Journal of Biomedical Imaging		y	T1, T1c, T2, FLAIR	Previously proposed	BFC:N4; IN:histogram matching + 0u1v	GeForce GTX 1080 Ti GPU	n/a	LGG, HGG	n	BT	LGG; HGG	274	1 (BraTS'15)	y	-		DSC; Sen; Spe

J	2018	P. Moeskops et al.	Evaluation of a deep learning approach for the segmentation of brain tissues and white matter hyperintensities of presumed vascular origin in MRI	NeuroImage: Clinical	Inter-rater variability evaluated (visual scores)	y	T1, T1 inversion recovery, FLAIR	Artificial neural networks	n/a	n/a	n/a	n	n	WML	DM2	20; 96 relatively healthy; 110	3 (UDES2, Reijmer et al., 2013); 3 (the Dutch Parelsnoer Study, Aalten et al., 2014)	n	Manual		DSC; avgSD; VC; Sen; DER; OER
J	2018	Y. Ling et al.	Validation and Optimization of BIANCA for the Segmentation of Extensive White Matter Hyperintensities	Neuroinformatics	Inter-rater agreement evaluated	works with single/multi	T1, FLAIR	Previously proposed	BE:BET; REG:FLIRT; IN:variance scaling	I5 3.2GHz	4-22 min	n	y	WML	CADASIL	90; 66	3 (Chabriat et al. 2016)	y	Semiautomatic		DSC; FPR; FNR; ICC; Bland-Altman
J	2018	M. Dadar et al.	Validation of T1w-based segmentations of white matter hyperintensity volumes in large-scale datasets of aging	Human Brain Mapping		n	T1	Previously proposed	denoising:ref; BFC:ref; IN:ref; REG:non-linear	n/a	n/a	n	n	WML	MCI; AD	70; 669; 481	2 (ADNI); 2 (ADNI2/GO); 3 (Davis Alzheimer's Disease Center, Hinton et al. 2010)	y	Manual, 1		VC; VR
J	2018	O. Charron et al.	Automatic detection and segmentation of brain metastases on multimodal MR images with a deep convolutional neural network	Computers in Biology and Medicine		n/a	T1, T1c, FLAIR	Artificial neural networks	REF:rigid (FLIRT) + resampling; BE:BET; BFC:N4; IN:0u1v + thresholding	n/a	n/a	n	n	MET	-	182 + 3 of other lesion causes	3 (Paul Strauss Center, Strasbourg, France)	n	Manual, multiple raters		DSC; Sen; PPV
J	2017	R. Meier et al.	Automatic estimation of extent of resection and residual tumor volume of patients with glioblastoma	Journal of Neurosurgery	Intended clinical application	y	T1, T1c, T2, FLAIR	Previously proposed	BE:ITK; REG	n/a	n/a	n	n	BT	GB	19	3	y	Manual, 5	supervision	other
J	2017	F. Kellner-Weldon et al.	Comparison of perioperative automated versus manual two-dimensional tumor analysis in glioblastoma patients	European Journal of Radiology	Intended clinical application, Preprocessing failure, Segmentation failure	y	T1, T1c, T2, FLAIR	Previously proposed	n/a	n/a	n/a	n	n	BT	GB	92x2	3	y	n/a		other
J	2017	A. de Sitter et al.	Performance of five research-domain automated WM lesion segmentation methods in a multi-center MS study	NeuroImage	Inter-rater agreement evaluated	y*	T1, FLAIR	Comparison	n/a	n/a	n/a	n	n	MS	MS	70	3 (MAGNIMS, Ropele et al., 2014)	y	Manual, 2	supervision	VD; ICC; DSC; DER; OER
J	2017	R. McKinley et al.	Fully automated stroke tissue estimation using random forest classifiers (FASTER)	J Cereb Blood Flow Metab	Inter-rater agreement evaluated	y	T1c, T2, DWI, DSC, PWI	Decision Tree	denoising:Gaussian iltering; REG:rigid; BE:ref; resampling:linear interpolation; IN:0-225 range	n/a	n/a	n	n	S	S	80x2 + 20	3 (Bernese stroke registry)	y	Semiautomatic		ROC; Sen; Spe; PPV
C	2015	R. McKinley et al.	Segmenting the ischemic penumbra: a decision forest approach with automatic threshold finding	Brainlesion: Glioma, Multiple Sclerosis, Stroke and Traumatic Brain Injuries		y	T1c, T2, DWI, CBF, CBV, TTP, Tmax	Decision Tree	filtering:ITK; IN: 0225r; REG	n/a	n/a	n	n	S	-	50	1 (ISLES'15)	n	-		DSC
J	2017	Y. Renping et al.	Coarse Classification to Region-Scalable Refining for White Matter Lesions Segmentation in Multi-Channel MRI	CNS & Neurological Disorders-Drug Targets		y	T1, T2, PD, FLAIR	Support Vector Machine, Level Set	REG:rigid; BE:ref; BFC: N3; IN: paper	n/a	n/a	n	n	WML	-	45	3 (ACCORD-MIND)	y	Manual, 1		TPR; TNR; PPV; DSC
J	2016	Ž. Lesjak, F. Pernuš, B. Likar, Ž. Špiclin	Validation of White-Matter Lesion Change Detection Methods on a Novel Publicly Available MRI Image Database	Neuroinformatics		y	T1, T2, FLAIR	Comparison	REG:affine; tissue maps – WM; BFC:N4; BE:BET 2; resampling:ANTs; IN:M u & v	n/a	n/a	n	n	WML	MS	20x2	3 (University Medical Centre Ljubljana)	y	Manual, 2	consensus	ROC; DSC; other
J	2016	M. B. Hansen et al.	Automated estimation of salvageable tissue: Comparison with expert readers.	Journal of Magnetic Resonance Imaging		y	PWI, DWI	Previously proposed	n/a	I7 2.6GHz	28 sec	n	y	S	-	167	3 (I-KNOW)	y	Manual, 4	consensus	DSC; VC; Bland-Altman
J	2016	R. Meier et al.	Clinical Evaluation of a Fully-automatic Segmentation Method for Longitudinal Brain Tumor Volumetry	Scientific Reports	Inter-rater agreement evaluated, Treatment response evaluation, Survival prediction	y	T1, T1c, T2, FLAIR	Previously proposed	BE; REG:rigid	n/a	n/a	n	y	BT	GBM	64 (14xN)	3	n	Manual, 2		VD; DSC
J	2016	N. Porz et al.	Fully Automated Enhanced Tumor Compartmentalization: Man vs. Machine Reloaded	PloS one		y	T1, T1c, T2, FLAIR	Previously proposed	n/a	n/a	n/a	n	n	BT	-	19	3	y	Manual, 2	supervision	DSC; PPV; Sen; VD; other
J	2015	E. Rios Velazquez et al.	Fully automatic GBM segmentation in the TCGA-GBM dataset: Prognosis and correlation with VASARI features	Scientific Reports		y	T1, T1c, T2, FLAIR	Previously proposed	BE; REG:rigid	n/a	5 min	Overall survival; 1 year survival	y	BT	GB	109	2 (TCIA, Clark et al. 2013)	y	Manual		VC; VR
J	2015	O. Maier et al.	Classifiers for Ischemic Stroke Lesion Segmentation: A Comparison Study	PloS one	Inter-rater agreement evaluated, Errors in preprocessing, Segmentation failure	both	T1 and/or DWI, FLAIR	Comparison	downsampling:ref; BE:ref; BFC:ref; IN:ref	n/a	n/a	n	n	S	S	37	3 (Meier et al. 2015)	n	Manual, 2		DSC; avgSSD; Hdff; Sen; PPV; ROC
J	2015	S. Roy et al.	Subject-Specific Sparse Dictionary Learning for Atlas-Based Brain MRI Segmentation	IEEE Journal of Biomedical and Health Informatics		y	T1, T2, PD, FLAIR	Sparse representation, Dictionary Learning	REG:rigid (FLIRT); BFC:N4; BE:Spectre	2.7GHz 12-core AMD	15 min	n	n	MS	MS	18; 122	3	n	Manual, 1		DSC; VD; TPR; FPR; avgSSD

J	2014	N. Porz et al.	Multi-Modal Glioblastoma Segmentation: Man versus Machine	PloS one			y	T1, T1c, T2, FLAIR	Previously proposed	n/a	n/a	n/a	n	n	BT	GBM	25	1 (BraTS'12); 3	y	Manual, 3	supervision	DSC; PPV; Sen; VE
J	2014	J. Gao et al.	Non-locally regularized segmentation of multiple sclerosis lesion from multi-channel MRI data	Magnetic Resonance Imaging			y	T1, T2, FLAIR	Other	REG:rigid	n/a	n/a	n	n	MS	MS	20	1 (MS GC MICCAI'08)	y	-		DSC; Spe; FNR; VD
J	2013	J. A. Maldjian et al.	Automated White Matter Total Lesion Volume Segmentation in Diabetes	American Journal of Neuroradiology	Visual validation of preprocessing		y	T1, FLAIR	Other	REG:toMNI; tissue maps:DARTEL + SPM8	n/a	n/a	n	n	WML	DM2	50 (+50 healthy)	3 (Diabetes Heart Study-Mind, Bowden et al. 2010)	n	Manual, 4	supervision, consensus	VC; Bland-Altman
J	2013	K. Nagenthiraja et al.	Automated Decision-Support System for Prediction of Treatment Responders in Acute Ischemic Stroke	Front. Neurol.	Intended clinical application		y	PWI, DWI	Previously proposed	REG:linear; reslicing:SPM8	I7 2.6GHz	31 sec	n	n	S	-	288	3 (I-KNOW); 3 (Remote Ischemic Perconditioning Study, Hougaard et al. 2013)	y	Manual, 4	majority	VC; VD
J	2011	S. D. Smart et al.	Validation of Automated White Matter Hyperintensity Segmentation	Journal of Aging Research	Inter-rater agreement evaluated		y	T1, FLAIR	Previously proposed	REG:SPM	n/a	n/a	n	n	WML	-	30	3 (Firbank et al. 2010)	n	Manual, 2		Jcc
J	2004	G. P. Mazzara et al.	Brain tumor target volume determination for radiation treatment planning through automated MRI segmentation	International Journal of Radiation Oncology*Biology*Physics	Inter-rater agreement evaluated; Segmentation failure		y	T1c, T2, PD	Previously proposed	REG	n/a	n/a	n	n	BT	G	11x2	3	y	Manual, 3		Acc
J	2003	S. Mehta et al.	Evaluation of voxel-based morphometry for focal lesion detection in individuals	NeuroImage			n	T1	Previously proposed	n/a	n/a	n/a	n	n	S	IFL	10	3 (Cognitive Neuroscience Patient Registry)	n	Manual, 1		VC; avgSSD; sdSSD; other
C	2017	S. Jain et al.	Unsupervised Framework for Consistent Longitudinal MS Lesion Segmentation	Medical Computer Vision and Bayesian and Graphical Models for Biomedical Imaging	Scan-rescan reproducibility		y	T1, FLAIR	Previously proposed	REG; BFC; IN:histogram matching	n/a	n/a	n	n	WML	MS	10; 12	3	y	Expert segmentation		TPR; FPR; DSC
C	2016	C. C. Benson et al.	Brain tumor segmentation from MR brain images using improved fuzzy c-means clustering and watershed algorithm	2016 International Conference on Advances in Computing, Communications and Informatics (ICACCI)			n	T1	Fuzzy C-Means, Watershed	denoising: median filter; BE: ref; contrast enhancement:ref	n/a	n/a	n	n	BT	GBM	90	3	n	Manual		DSC; TI
C	2013	N. Weiss et al.	Multiple Sclerosis Lesion Segmentation Using Dictionary Learning and Sparse Coding	Medical Image Computing and Computer-Assisted Intervention – MICCAI 2013			n	FLAIR	Sparse representation, Dictionary Learning	BE:BET; BFC:N4; IN: sqrt of largest L1 norm of all patches	2.4GHz 4GB RAM	5 min	n	n	MS	MS	20	1 (MS GC MICCAI'08)	y	-		DSC; TPR; PPV
C	2013	H. Wang and P.A. Yushkevich	Multi-atlas Segmentation without Registration: A Supervoxel-Based Approach	MICCAI 2013			y	T1, T1c, T2, FLAIR	Other	IN:histogram equalization + 0-1 range	2 GHz	few sec	n	n	BT	LGG; HGG	20 (25 synthetic)	1 (BraTS'12)	y	-		DSC
C	2009	D. García-Lorenzo et al.	Multiple Sclerosis Lesion Segmentation Using an Automatic Multimodal Graph Cuts	MICCAI 2009	Inter-rater agreement evaluated		y	T1, T2, PD	Expectation-Maximization Gausian mixture model, Graph Cut	BFC:ref; REG:ref; BE:ref	n/a	n/a	n	n	MS	MS	10	3	n/a	Manual, 5	majority	DSC
C	2006	J. Corso et al.	Multilevel Segmentation and Integrated Bayesian Model Classification with an Application to Brain Tumor Segmentation	MICCAI 2006			y	T1, T1c, FLAIR	Bayesian classification	REG:FSL; denoising:FSL; BE:FSL; IN:FSL	n/a	n/a	n	n	BT	GBM	30	3	n/a	Manual		Jcc
J	2019	J. Tong et al.	MRI brain tumor segmentation based on texture features and kernel sparse coding	Biomedical Signal Processing and Control			n	FLAIR	Sparse representation, Dictionary Learning	IN:0-225 range; denoising:Gaussian filtering; brain mask	4GHz 32GB RAM	38 sec	n	n	BT	LGG; HGG	50	1 (BraTS'12)	y	-		Jcc; DSC; Spe; Sen
J	2018	J. Tong et al.	Kernel sparse representation for MRI image analysis in automatic brain tumor segmentation	Frontiers of Information Technology & Electronic Engineering			y	T1, T1c, T2, FLAIR	K-Means, Sparse representation	IN:0-225 range; denoising:Gaussian filtering; brain mask	4GHz 32 GB RAM	40s	n	n	BT	LGG; HGG	384	1 (BraTS'15)	y	-		DSC; PPV; Sen; Kappa

J	2018	O. Ghribi et al.	Multiple sclerosis exploration based on automatic MRI modalities segmentation approach with advanced volumetric evaluations for essential feature extraction	Biomedical Signal Processing and Control		y	T1, T1c; T1, T2, FLAIR; T1, T2, PD, FLAIR	Support Vector Machine, Genetic Algorithm	BFC; REG:BET; BE: FSL; denoising: ADF; IN	n/a	n/a	n	n	MS	MS	70; 45; 10x4, 3x5, 1x6	1 (MS GC MICCAI'08); 1 (MSC'15); 3 (Habib-Bourguiba University Hospital)	y	Manual, 1&2		DSC; TPR; PPV; Jcc; TPR; FPR; FP; VD
J	2018	A. Vishnuvarthanan et al.	Development of a combinational framework to concurrently perform tissue segmentation and tumor identification in T1 - W, T2 - W, FLAIR and MPR type magnetic resonance brain images	Expert Systems with Applications		y	T1, T2, FLAIR, MPR	Fuzzy C-Means, Bacteria Foraging Optimization	IN: histogram equalization	I7 2.6GHz 8GB RAM	unclear	n	n	BT	T	4; 25	1 (BraTS'13); 2 (The Whole Brain Atlas);	y	Manual		DSC; Sen; Spe
J	2018	J. Jiang et al.	UBO Detector – A cluster-based, fully automated pipeline for extracting white matter hyperintensities	NeuroImage		y	T1, FLAIR	K-Nearest Neighbours	REG:SPM12; tissue maps:SPM12; REG:DARTEL; BE	2.2GHz 256GB RAM	15 min	n	y	WML	-	397; 1037 (subsample longitudinal)	3 (OATS, Sachdev et al. 2013); 3 (Sydney Memory and Ageing Study, Sachdev et al. 2010)	y	Manual, 2		DSC; TPR; FPR; Spe; Acc; ICC
J	2018	H. Li et al.	Fully convolutional network ensembles for white matter hyperintensities segmentation in MR images	NeuroImage		y	T1, FLAIR	Artificial neural networks	cropping; IN:Gaussian normalization	32 GB RAM	CPU: 60 sec GPU: 8 sec	n	y	WML	MS	170	1 (WMH Segmentation Challenge '17)	y	-		DSC; Hdff; avgVD; Sen; F1
J	2018	T. Kaur et al.	A joint intensity and edge magnitude-based multilevel thresholding algorithm for the automatic segmentation of pathological MR brain images	Neural Comput & Applic	No preprocessing	n	FLAIR	Thresholding, Particle Swarm Optimization	IN:0-255 range; BE:Otsu's thresholding	3.1GHz i5 4GB RAM	5 sec	n	n	BT	LGG	10; 20; 10	1 (BraTS'12); 1 (BraTS'15); 3 (Advance Diagnostic Centre, Ludhiana, Punjab, India)	y	Manual,1		DSC; Jcc; other
J	2018	L. Rundo et al.	NeXt for neuro-radiosurgery: A fully automatic approach for necrosis extraction in brain tumor MRI using an unsupervised machine learning technique	International Journal of Imaging Systems and Technology		n	T1c	Fuzzy C-Means	morphological operations on GTV	2.8GHz i7 16BG RAM	< 1 sec	n	n	NEC	-	32	3	n	Manual,1		DSC; Jcc; Sen; Spe; MAD; Hdff; other
J	2018	M. Soltaninejad et al.	Supervised learning based multimodal MRI brain tumour segmentation using texture features from supervoxels	Computer Methods and Programs in Biomedicine		y	T1, T1c, T2, FLAIR, DTI	Random Forest	Eddy curent correction:FSL; BE:BET; REG:SPM12; IN:histogram matching + dynamic range normalization	n/a	n/a	n	n	BT	LGG; HGG	11; 30	1 (BraTS'13); 3	y	Manual,1		PPV; Sen; DSC; other
J	2018	E. A. AlBadawy et al.	Deep learning for segmentation of brain tumors: Impact of cross-institutional training and testing	Medical Physics	Minimal preprocessing	y	T1, T1c, FLAIR	Artificial neural networks	REG:FLIRT	n/a	n/a	n	n	BT	GB	44	2 (TCIA, Clark et al. 2013)	y	Manual, 3	supervision	DSC; avgHdff; avgVD; Sen; PPV
J	2018	T. Kaur et al.	A novel fully automatic multilevel thresholding technique based on optimized intuitionistic fuzzy sets and tsallis entropy for MR brain tumor image segmentation	Australas Phys Eng Sci Med	No preprocessing	n	FLAIR	Thresholding, Particle Swarm Optimization	none	3.10GHz i5 4GB RAM	< 5 sec	n	n	BT	LGG; HGG	8; 8	1 (BraTS'12); 3	y	Manual		DSC; Jcc; other
J	2018	M. Salem et al.	A supervised framework with intensity subtraction and deformation field features for the detection of new T2-w lesions in multiple sclerosis	NeuroImage: Clinical		y	T1, PD, T2, FLAIR	Logistic Regression	BE:ROBEX; BFC:N4; IN:histogram matching; REG:ITKv4Demons	n/a	n/a	n	y	MS	MS	60x2	3 (Vall d'Hebron Hospital)	n	Semiautomatic		DSC; VC
J	2018	E. B. Shimol et al.	Computer-based radiological longitudinal evaluation of meningiomas following stereotactic radiosurgery	Int J CARS		n	T1c	Active Contour	REG:SPM; IN:histogram normalization	n/a	n/a	n	n	BT	MEN	28	3	n	Manual, 1		DSC; Sen; maxSD; avgSD
J	2018	Z. Zhao et al.	Automated glioma detection and segmentation using graphical models	PloS one		y	T1, T1c, T2, FLAIR	Conditional Random Field	n/a	n/a	n/a	n	n	BT	LGG; HGG	30; 274; 161	1 (BraTS'13); 1 (BraTS'15); 3 (Henan Provincial People's Hospital)	y	Manual, 1		DSC; Hdff; VC; Spe; Sen
J	2018	M. M. Cheriyan et al.	Blind source separation with mixture models – A hybrid approach to MR brain classification	Magnetic Resonance Imaging		y	T1, T2, FLAIR	Gaussian Mixture Model, Particle Swarm Optimization	n/a	n/a	n/a	n	n	BT, MS, S	BT, MS, HEM	38 + 22 healthy; 152	3 (Medall Diagnostics, Kochi)	n	Manual, 3		Jcc; Sen; Spe; Acc
J	2018	M. B. Naceur et al.	Fully Automatic Brain Tumor Segmentation using End-To-End Incremental Deep Neural Networks in MRI images	Computer Methods and Programs in Biomedicine		y	T1, T1c, T2, FLAIR	Artificial neural networks	IN:1-99% clipping + 0u1v	GPU	22 sec	n	n	BT	-	285	1 (BraTS'17)	y	-		DSC; Sen; Spe; Hdff
J	2018	M. K. Abd-Ellah et al.	Two-phase multi-model automatic brain tumour diagnosis system from magnetic resonance images using convolutional neural networks	EURASIP Journal on Image and Video Processing	Detection followed by segmentation	n	T2	Artificial neural networks	resizing and RGB conversion	n/a	n/a	n	n	BT	LGG; HGG	19; 30	1 (BraTS'13)	y	-		Sen; Spe; Acc; PPV; NPV; DSC
J	2018	J. K. Boldsen et al.	Better Diffusion Segmentation in Acute Ischemic Stroke Through Automatic Tree Learning Anomaly Segmentation	Front. Neuroinform.		n	DWI	Decision Tree	IN: division by mean of contralateral hemisphere; smoothing:Gaussian kernel; morphological operations	n/a	n/a	n	n	S	S	108	3 (I-KNOW)	y	Manual, 1		DSC

J	2018	R. Guerrero et al.	White matter hyperintensity and stroke lesion segmentation and differentiation using convolutional neural networks	NeuroImage: Clinical		y	T1, FLAIR	Artificial neural networks	REG:FLIRT; reslicing; tissue maps; IN:0u1v + clipping (3stdev)	n/a	n/a	n	n	WML, S	S	167	3 (Brain Research Imaging Centre of Edinburgh)	n* various protocols	Semiautomatic		DSC; VD; other
J	2018	T. Zhan et al.	A Glioma Segmentation Method Using CoTraining and Superpixel-Based Spatial and Clinical Constraints	IEEE Access		y	T1, T1c, T2, FLAIR	Support Vector Machine, Sparse representation	BFC:N4; IN: 0-255 range	n/a	n/a	n	n	BT	LGG; HGG	30	1 (BraTS'12); 1 (BraTS'13)	y	-		DSC; PPV; Sen
J	2018	S. Cui et al.	Automatic Semantic Segmentation of Brain Gliomas from MRI Images Using a Deep Cascaded Neural Network	Journal of Healthcare Engineering		y	T1, T1c, T2, FLAIR	Fuzzy C-Means, Artificial neural networks	BFC:N4; IN:1-99% clipping + 0u1v	Intel E5 GTX 1080	2 sec	n	n	BT	LGG; HGG	274	1 (BraTS'15)	y	-		DSC; PPV; Sen
J	2018	S. Bonte et al.	Machine learning based brain tumour segmentation on limited data using local texture and abnormality	Computers in Biology and Medicine		y	T1c, FLAIR	Random Forest	REG:SPM12; tissue maps: SPM; BFC:SPM12	n/a	n/a	n	n	BT	LGG; HGG	30; 285; 257	1 (BraTS'17); 1 (BraTS'17); 3 (Ghent University Hospital)	y	n/a		DSC
J	2018	M. J. Fartaria et al.	Partial volume-aware assessment of multiple sclerosis lesions	NeuroImage: Clinical		y	T1, FLAIR	Other	REG:rigid (ELASTIX); BFC:N4; BE:in house; tissue maps:in house	n/a	n/a	n	n	MS	early MS	39	3	n	Manual, 2	consensus	DSC; FPR; Detection Rate
J	2018	C. Ma et al.	Concatenated and Connected Random Forests With Multiscale Patch Driven Active Contour Model for Automated Brain Tumor Segmentation of MR Images	IEEE Transactions on Medical Imaging		y	T1, T1c, T2, FLAIR	Random Forest, Active Contour	n/a	n/a	5 min	n	n	BT	-	n/a	1 (BraTS'15); 1 (TCIA, Bakas et al. 2017)	y	-		DSC; Sen; Spe
J	2018	I. Razzak et al.	Efficient Brain Tumor Segmentation with Multiscale Two-Pathway-Group Conventional Neural Networks	IEEE Journal of Biomedical and Health Informatics		y	T1, T1c, T2, FLAIR	Artificial neural networks	IN:1-99% clipping + 0u1v + 0-1 range; BFC:N4	n/a	3 min	n	n	BT	-	285 + 110;	1 (BraTS'13); 1 (BraTS'15)	y	-		DSC; Sen; Spe
J	2018	S. Hussain et al.	Segmentation of glioma tumors in brain using deep convolutional neural network	Neurocomputing		y	T1, T1c, T2, FLAIR	Artificial neural networks	BFC:N4; IN: 1-99% clipping + 0u1v	i7 16GB RAM	5 min	n	n	BT	LGG; HGG	30; 274	1 (BraTS'13); 1 (BraTS'15)		-		DSC; Sen; Spe
J	2018	S. Banerjee et al.	Automated 3D segmentation of brain tumor using visual saliency	Information Sciences	Inter-rater agreement evaluated	y	T1c, T2, FLAIR	Other, Grow Cut	BRATS only	I7 3.4GHz 16GB RAM	2 min	n	n	BT	LGG; HGG	30	1 (BraTS'13)	y	-		DSC; Hdff; other
J	2018	E. Binaghi et al.	Meningioma and peritumoral edema segmentation of preoperative MRI brain scans	Computer Methods in Biomechanics and Biomedical Engineering: Imaging & Visualization		y	T1c, FLAIR	Sparse representation, Graph Cut	REG:Iterative Closest Point; contrast enhancement:log transform	2.26GHz	145 sec	n	n	BT	T	15	3	n	Manual, 3	majority	DSC; Jcc; VE
J	2018	C. Qin et al.	A large margin algorithm for automated segmentation of white matter hyperintensity	Pattern Recognition		y	T1, FLAIR	Other	REG:FLIRT; tissue maps:ref; IN:ref	3.6GHz 16GB RAM	16 sec	n	n	WML	-	88	3 (Brain Research Imaging Centre of Edinburgh)	n	Semiautomatic		DSC; Acc; PPV; Sen; Bland-Altman; VC; other
J	2018	Y. Ding et al.	Multi-modal brain tumor image segmentation based on SDAE	International Journal of Imaging Systems and Technology		y	T1, T1c, T2, FLAIR	Artificial neural networks	contrast enhancement:top-hat transform	12 GB RAM	n/a	n	n	BT	-	95 ?	1 (BraTS'15)	y	-		DSC; Jcc
J	2018	A. M. Valcarcel et al.	MIMoSA: An Automated Method for Intermodal Segmentation Analysis of Multiple Sclerosis Brain Lesions	Journal of Neuroimaging		y	T1, T2, FLAIR, PD	Logistic Regression	REG:rigid; BFC:N3; brain tissue extraction:BE:SPECTRE + thresholding; IN:0u1v or WhiteStripe	n/a	22 min	n	n	MS	MS	94; 19xn	1 (MSC'15); 3 (Johns Hopkins Hospital)	y	Manual, 1		DSC; ROC; VC
J	2018	A. R. Raju et al.	Bayesian HCS-based multi-SVNN: A classification approach for brain tumor segmentation and classification using Bayesian fuzzy clustering	Biocybernetics and Biomedical Engineering		y	T1, T1c, T2, FLAIR	Fuzzy Clustering	n/a	2GB RAM	n/a	benign, malignant	n	BT	LGG; HGG	30	1 (BraTS'12)	y	-		Sen
J	2018	A. Essadike et al.	Brain tumor segmentation with Vander Lugt correlator based active contour	Computer Methods and Programs in Biomedicine		n/a	n/a	Active Contour, Other	n/a	n/a	<1 sec	n	n	BT	LGG; HGG	45 + 65 synthetic; 35	1 (BraTS'12); 1 (BraTS'13)	y	-		DSC; Hdff; Spe; Sen
J	2018	Y. Wang et al.	Voxel-based automated detection of focal cortical dysplasia lesions using diffusion tensor imaging and T2-weighted MRI data	Epilepsy & Behavior		y	T2, DTI	Other	BE:FSL; IN; REG; tissue maps; BFC; resampling	n/a	n/a	n	n	FCD		12	3 (Sanbo Brain Hospital Capital Medical University)	n	Manual, 1		ROC; FPR
J	2018	A. Pinto et al.	Hierarchical brain tumour segmentation using extremely randomized trees	Pattern Recognition		y	T1, T1c, T2, FLAIR	Decision Tree	BFC:N4; IN: histogram matching	n/a	n/a	n	n	BT	LGG; HGG	65	1 (BraTS'13)	y	-		DSC; PPV; Sen
J	2018	G. B. Praveen et al.	Ischemic stroke lesion segmentation using stacked sparse autoencoder	Computers in Biology and Medicine		y	T1, T2, DWI, FLAIR	Artificial neural networks	BFC:N4; IN:0u1v + whitening; resizing	3.5GHz 32 GB RAM	10 min	n	n	S	S	28	1 (ISLES'15)	y	-		DSC; PPV; Sen; Spe; Acc; ROC

J	2018	J. Knight et al.	Voxel-Wise Logistic Regression and Leave-One-Source-Out Cross Validation for white matter hyperintensity segmentation	Magnetic Resonance Imaging		n	FLAIR	Logistic Regression	BFC:SPM12; REG:affine + non-linear; resmpling:trilinear; BE:brain tissue probability maps; IN:histogram matching + 0-1 range	n/a	n/a	n	y	WML	-	96; 110	1 (WMH Segmentation Challenge '17); 1 (MSC'15); 1 (MS Lesion Segmentation Challenge '16)	y	-		DSC; PPV; Sen
J	2018	S. Amiri et al.	Tree-based Ensemble Classifier Learning for Automatic Brain Glioma Segmentation	Neurocomputing		y	T1c, T2, FLAIR	Decision Tree, Bayesian classification	BFC:N4; IN:histogram linear transformation	n/a	n/a	n	n	BT	HGG	50	1 (BraTS'15)	y	-		DSC
J	2018	J. Liu et al.	A Cascaded Deep Convolutional Neural Network for Joint Segmentation and Genotype Prediction of Brainstem Gliomas	IEEE Transactions on Biomedical Engineering		n	T1	Artificial neural networks	resizing; cropping	n/a	n/a	prediction of H3 K27M mutation	n	BT	T; LGG, HGG	55; 280	1 (BraTS'17); 3	y	Manual, 2		DSC; SD
J	2018	S. Sasikanth and S. Suresh Kumar	Glioma tumor detection in brain MRI image using ANFIS-based normalized graph cut approach	International Journal of Imaging Systems and Technology		n/a	n/a	Graph Cut	n/a	2.4GHz 2GB RAM	n/a	normal, abnormal	n	BT	LGG; HGG	100	1 (BraTS'15)	y	-		DSC; Acc; Sen; Spe
J	2018	J. V. Manjón et al.	MRI white matter lesion segmentation using an ensemble of neural networks and overcomplete patch-based voting	Computerized Medical Imaging and Graphics		n	FLAIR	Thresholding, Artificial neural networks	denoising:non-local means; REG:affine; BFC:SPM12; BE:SPM12; IN:division by median and squared	i7 16GB RAM	3 min	n	n	WML	-	128;20	1 (MS GC MICCAI'08); 3 (AIBL, Ellis et al., 2009)	y	Manual, 1		DSC; Spe; Sen; VD; VC
J	2018	M. F. Rachmadi et al.	Segmentation of white matter hyperintensities using convolutional neural networks with global spatial information in routine clinical brain MRI with none or mild vascular pathology	Computerized Medical Imaging and Graphics	Inter-rater agreement evaluated	y	T1, FLAIR	Artificial neural networks	REG:rigid(FLIRT); BE:opti-BET + morphological operations; tissue maps:FAST; IN:histogram matching + 0u1v	3.4GHz GPU 8GB RAM	9 sec	n	n	WML	-	20x3; 268 (with only Fazekas scores)	2 (ADNI)	y	Semiautomatic		DSC; PPV; Sen; VC; VD
J	2017	A. R. Abdulraqeb et al.	An Automated Method for Segmenting Brain Tumors on MRI Images	Biomed Eng		n	T1c	Thresholding	n/a	n/a	n/a	n	n	BT	-	12; 44	3	y	Expert segmentation		DSC; Jcc; Sen; Spe
J	2017	S. Cui et al.	Brain tumor Segmentation using fully convolutional networks	Journal of Medical Imaging and Health Informatics		y	T1, T1c, T2, FLAIR	Artificial neural networks	BFC:N4; IN:mode & deviation scaling + 0-255 range	GeForce GTX 1080; 2 Intel e5 2603 CPUs	<30 sec	n	n	BT	HGG	220	1 (BraTS'15)	y	-		DSC; PPV; Sen
J	2017	Y. Zhuge et al.	Brain tumor segmentation using holistically nested neural networks in MRI images	Medical Physics		y	T1, T1c, T2, FLAIR	Artificial neural networks	BFC:N4; IN:histogram matching	2.66GHz 32 GB RAM	30 sec	n	n	BT	HGG	20; 10	1 (BraTS'13); 3	y	Manual, 1		DSC; Sen
J	2017	L. Chen et al.	Fully automatic acute ischemic lesion segmentation in DWI using convolutional neural networks	NeuroImage: Clinical		n	DWI	Artificial neural networks	resampling:homogeneous linear; IN:0u1v	i7 16GB RAM	<1 sec	n	n	S	S	741	3	y	Expert segmentation		DSC; Detection Rate; other
J	2017	M. Dadar et al.	Performance comparison of 10 different classification techniques in segmenting white matter hyperintensities in aging	NeuroImage	Inter-rater agreement evaluated	y	T1, T2, PD, FLAIR	Comparison	denoising:MINCnlm; BFC:MINCnu_estimate; IN:histogram matching & 0-100 range; REG; BE	i7 2.6GHz 20GB RAM	0.11 – 3021 sec	n	y	WML	CI	70; 32; 53; 46	2 (ADNI); 2 (ADNI2/GO); 2 (NACC) 3 (Davis Alzheimer's Disease Center, Hinton et al. 2010)	y	Manual, 2		DSC; ICC; Sen; PPV; OER; DER
J	2017	K. Murphy et al.	Automatic quantification of ischemic injury on diffusion-weighted MRI of neonatal hypoxic ischemic encephalopathy	NeuroImage: Clinical	Inter-rater agreement evaluated	y	ADC, DWI	Random Forest	BE:BET2; BFC:N4; IN:histogram matching	n/a	n/a	n	n	S	S	74	3 (Wilhelmina Children's Hospital, University Medical Center Utrecht)	y	Manual, 2		ROC: Sen; Spe; F1; VC
J	2017	S. Damangir et al.	Reproducible segmentation of white matter hyperintensities using a new statistical definition	Magnetic Resonance Materials in Physics, Biology and Medicine		y	T1, T2, PD, FLAIR	Other	REG:FLIRT; BE:BET; BFC:N3; tissue maps:FSL-FAST	n/a	n/a	n	y	WML	CI	119	3 (Kings Health Partners-Dementia Case Register)	n	Manual, 1		FNR; VC; DSC
J	2017	M. Soltaninejad et al.	Automated brain tumour detection and segmentation using superpixel-based extremely randomized trees in FLAIR MRI	International Journal of Computer Assisted Radiology and Surgery		n	FLAIR	Decision Tree	n/a	n/a	n/a	n	n	BT	-	19; 30	1 (BraTS'12); 3	y	Manual, 1		DSC; Sen; PPV; other
J	2017	M. Dadar et al.	Validation of a Regression Technique for Segmentation of White Matter Hyperintensities in Alzheimer's Disease	IEEE Transactions on Medical Imaging		y	T1, T2, PD, FLAIR	Linear regression, Thresholding	denoising:non-local means; BFC:N3; IN:histogram matching	i3 3.3GHz	1.6 sec	n	n	WML	-	80; 40; 10	2 (ADNI2/GO); 3 (Davis Alzheimer's Disease Center, Hinton et al. 2010) 3 (PREVENT-AD, Tremblay-Mercier et al. 2014)	y	Semiautomatic; Manual, 2	union; supervision	ICC; DSC; FPR; Sen
J	2017	Z. Li et al.	Low-Grade Glioma Segmentation Based on CNN with Fully Connected CRF	Journal of Healthcare Engineering		n	FLAIR	Artificial neural networks, Conditional Random Field	BE:BrainSuite; BFC:BrainSuite	Intel Xeon 2.4GHz Nvidia Quadro	2-10 min	n	n	BT	LGG	160	3 (Shanghai Huashan Hospital)	n	Manual, 2		DSC; PPV; Sen
J	2017	A. Galimzianova et al.	Locally adaptive magnetic resonance intensity models for unsupervised segmentation of multiple sclerosis lesions	Journal of Medical Imaging	Inter-rater agreement evaluated	y	T1, T2, FLAIR	Gaussian Mixture Model, Markov Random Field	REG:ref; BE:ref; BFC:ref; resampling	n/a	n/a	n	n	MS	MS	30	3	n	Semiautomatic		DSC; VC
J	2017	C. Bowles et al.	Brain lesion segmentation through image synthesis and outlier detection	NeuroImage: Clinical		y	T1, FLAIR	Gaussian Mixture Model, Support Vector Machine	REG:rigid+free form (MIRTK); BFC:N4; BE:pincram; tissue maps:MALPEM; IN:ref	n/a	n/a	n	n	WML	SVD	127	3 (Brain Research Imaging Centre of Edinburgh)	n – different protocols	Semiautomatic		DSC; avgSD; Hdff; PPV; Sen; ICC; Bland-Altman

Gryska E, *et al. BMJ Open* 2022; 11:e042660. doi: 10.1136/bmjopen-2020-042660

J	2017	O. Ghribi et al.	An Advanced MRI Multi-Modalities Segmentation Methodology Dedicated to Multiple Sclerosis Lesions Exploration and Differentiation	IEEE Transactions on NanoBioscience	Inter-rater agreement evaluated	y	T1c, FLAIR	Gaussian Mixture Model, Thresholding	REG:rigid; resampling:spline interpolation; BFC; BE:FSL; denoising: ADF; IN	i5 1.8GHz	2 min	n	n	MS	-	70 + 50 healthy; 45; 21x4 (or more)	1 (MS GC MICCAI'08); 1 (MSC'15); 3 (Habib-Bourguiba University Hospital)	y	Manual, 1		DSC; Jcc; PPV; TPR; FPR; SD; VD; DE; OE
J	2017	K. Kamnitsas et al.	Efficient multi-scale 3D CNN with fully connected CRF for accurate brain lesion segmentation	Medical Image Analysis		y	n/a	Artificial neural networks	BE:ROBEX; resampling; REG:affine; IN:0u1v	NVIDIA GTX Titan X GPU 3GB RAM	3 min	n	y	TBI; BT; S	-	61; 384; 64	1 (ISLES'15); 1 (BraTS'15); 3 (Neurosciences Critical Care Unit at Addenbrooke's Hospital, Cambridge, UK)	y	n/a		DSC; Sen; PPV; avgSD; Hdff
J	2017	V. Rajinikanth et al.	Entropy based segmentation of tumor from brain MR images – a study with teaching learning based optimization	Pattern Recognition Letters		y	T1c, T2, FLAIR	Level Set	BE:thresholding; Multilevel thresholding + teaching-learning based optimization – Shannon entropy optimization	n/a	n/a	n	n	BT	-	15?	1 (BraTS'12)	y	-		DSC; Jcc; FPR; FNR; PPV; F-score; Sen; Spe; Acc; other
J	2017	S. Abbasi and F. Tajeripour	Detection of brain tumor in 3D MRI images using local binary patterns and histogram orientation gradient	Neurocomputing		y	T1, T1c, T2, FLAIR	Thresholding, Random Forest	BFC:N4; IN:histogram matching	4GHz 32GB RAM	1900 sec	n	n	BT	LGG; HGG	30 (+50 simulated)	1 (BraTS'13)	y	-		DSC; Jcc
J	2017	F. Binczyk et al.	MiMSeg - an algorithm for automated detection of tumor tissue on NMR apparent diffusion coefficient maps	Information Sciences		y	DWI (ADC), FLAIR	Expectation-Maximization Gausian mixture model, K-Means	BE:BET; tissue maps:Otsu's thresholding	n/a	n/a	n	n	BT	GBM	17x7	3 (Maria Sklodowska-Curie Memorial Cancer Center and Institute of Oncology Gliwice Branch, Gliwice, Poland)	n	Manual, 1		DSC; Sen; Spe; PPV; NPV
J	2017	M. Rincón et al.	Improved Automatic Segmentation of White Matter Hyperintensities in MRI Based on Multilevel Lesion Features	Neuroinformatics	Inter-rater agreement evaluated; Visual inspection of preprocessing	y	T1, FLAIR	Thresholding, Support Vector Machine	REG:rigid; BFC: SPM8; tissue maps:Freesurfer; IN:Gaussian fit	n/a	n/a	n	n	WML	S, MCI	28	3 (Selnes et al. 2015)	n	Manual, 3	majority	DSC; other
J	2017	M. Havaei et al.	Brain tumor segmentation with Deep Neural Networks	Medical Image Analysis		y	T1, T1c, T2, FLAIR	Artificial neural networks	BFC:N4; IN:1-99% clipping + 0u1v	GPU	~3 min	n	n	BT	LGG; HGG	65	1 (BraTS'13)	y	-		DSC; Sen; Spe
J	2017	T. Zhan et al.	Brain Tumor Segmentation Using Deep Belief Networks and Pathological Knowledge	CNS & Neurological Disorders-Drug Targets		y	T1, T1c, T2, FLAIR	Logistic Regression, Graph Cut, Artificial neural networks, Markov Random Field	n/a	n/a	n/a	n	n	BT	LGG; HGG	10 hgg + N LGG (+20 synthetic)	1 (BraTS'12); 1 (BraTS'13)	y	-		DSC; Sen; PPV; F-score
J	2017	E. Kellner et al.	Automated Infarct Core Volumetry Within the Hypoperfused Tissue: Technical Implementation and Evaluation	Journal of Computer Assisted Tomography		y	PWI, DWI	Other	BE; hemisphere extraction	n/a	1 min	n	n	S	S	30	1 (ISLES'15)	y	-		TPR; FPR; FDR; DSC
J	2017	S. Adler et al.	Novel surface features for automated detection of focal cortical dysplasias in paediatric epilepsy	Neuroimage: Clinical	Manual corrections of preprocessing	y	T1, FLAIR	Artificial neural networks	REG:Freesurfer: resampling; IN; BFC; BE:ref	n/a	n/a	n	n	FCD	EP	22 + 28 healthy	3 (Great Ormond Street Hospital)	n	Expert segmentation		ROC; Sen
J	2017	P. Sivakumar and P. Ganeshkumar	An efficient automated methodology for detecting and segmenting the ischemic stroke in brain MRI images	International Journal of Imaging Systems and Technology		n/a	n/a	Graph Cut	enhancement: histogram equalization; Gabor transform	2.4GHz Core-2-Duo 4GB RAM	n/a	normal, abnormal	n	S	S	20 +25 healthy	1 (ISLES)	y	-		Sen; Spe; Acc; PPV; NPV; VC
J	2017	E. Ilunga–Mbuyamba et al.	Automatic selection of localized region-based active contour models using image content analysis applied to brain tumor segmentation	Computers in Biology and Medicine		n/a	n/a	K-Nearest Neighbours, Random Forest, Active Contour,	n/a	n/a	n/a	n	n	BT	LGG, HGG	21	1 (BraTS'12); 3 (University Hospital of Leipzig, Germany)	y	Manual, 1		DSC; Hdff
J	2017	S. Valverde et al.	Improving automated multiple sclerosis lesion segmentation with a cascaded 3D convolutional neural network approach	NeuroImage		y	T1, T2, PD, FLAIR	Artificial neural networks	BE:BET; BFC:N3; reg:SPM	32 GB RAM	n/a	n	y	MS	MS	45; 60	1 (MS GC MICCAI'08); 3 (Hospital Vall d'Hebron, Barcelona, Spain)	y	Semiautomatic		VD; TPR; FPR; DSC; PPV
J	2017	A. S. Keçeli et al.	A GPU-Based Approach for Automatic Segmentation of White Matter Lesions	IETE Journal of Research		n	FLAIR	Fuzzy C-Means, Region Growing	BE:morphological/watershed/ACM; enhancement: histogram equalization	GPU	<1 sec; ~5-10 sec	n	n	WML	-	10	3 (Hacettepe University Medical School Hospital)	n	Expert segmentation		SI; TPR; FPR
J	2016	B. H. Menze et al	A generative probabilistic model and discriminative extensions for brain lesion segmentation – with application to tumor and stroke	IEEE Trans Med Imaging		y	T1, T1c, T2, FLAIR	Markov Random Field, Watershed , Expectation-Maximization Gausian mixture model,	BFC:polynomial spline model;IN:linear template matching	n/a	n/a	n	n	BT; S	LGG; HGG	45; 18	1 (BraTS'12); 1 (BraTS'13)	y	-		DSC
J	2016	M. Strumia et al.	White Matter MS-Lesion Segmentation Using a Geometric Brain Model	IEEE Transactions on Medical Imaging		y	T1, FLAIR	Expectation-Maximization Gausian mixture model	REG:ITK; BE:BET; BFC:ref	n/a	3-7 hrs	n	n	MS	MS	42; 20	1 (MS GC MICCAI'08); 3	y	Manual, 1&2		VD; avgSD; TPR; FPR; DSC
J	2016	L. Griffanti et al.	BIANCA (Brain Intensity AbNormality Classification Algorithm): A new tool for automated segmentation of white matter hyperintensities	NeuroImage	Scan-rescan reproducibility	y: flexible to the number of input sequences	T1, FLAIR	K-Nearest Neighbours	BE: BET; REG:FLIRTIN: variance scaling	3GHz Intel Xeon	12-13 min	n	y	WML	neurodegenerative and vascular disorders	85; 474 + 20 longitudinal	3 (OPTIMA, Zamboni et al., 2013); 3 (OXVASC, Rothwell et al., 2004)	y	Manual, 3		DSC; FPR; FNR; DER; OER; ICC; Bland-Altman
J	2016	M. Cabezas et al.	Improved Automatic Detection of New T2 Lesions in Multiple Sclerosis Using Deformation Fields	American Journal of Neuroradiology		n	T1, PD; FLAIR	Thresholding	BE:BET2; BFC:N4; IN:histogram matching	n/a	n/a	n	n	MS	CIS; early MS	36x2	3	n	Semiautomatic		avgSD; DSC; VO; other
J	2016	Z. Karimaghaloo et al.	Adaptive multi-level conditional random fields for detection and segmentation of small enhanced pathology in medical images	Mecidal image Analysis		y	T1, T1c, T2, PD, FLAIR	Conditional Random Field	BE:BET; BFC:N3; IN: histogram matching	n/a	n/a	n	n	MS	-	1760; 2770	3	y	Manual, 2	consensus	Sen; FDR
J	2016	M. Ghafoorian et al.	Automated detection of white matter hyperintensities of all sizes in cerebral small vessel disease: Automated detection of white matter hyperintensities of all sizes	Medical Physics		y	T1, FLAIR	Random Forest, Boosting	REG: FLIRT; BE:BET; BFC:FSL-FAST; IN:histogram matching	n/a	n/a	n	n	WML	SVD	503	3 (RUNDMC, van Norden et al. 2011)	n	Manual, 1-3	supervision	FROC
J	2016	Y. Liu et al.	Automatic metastatic brain tumor segmentation for stereotactic radiosurgery applications	Physics in Medicine and Biology		n	T1c	Thresholding, Active Contour	BE:ROBEX	E3-1505M CPU 32GB RAM	<7 min	n	n	MET	-	15?	3 (University of Texas Southwestern Medical Center)	y	Manual, 1		DSC; SI; Hdff; avgSSD; sdSSD; other

J	2016	T. Brosch et al.	Deep 3D Convolutional Encoder Networks With Shortcuts for Multiscale Feature Integration Applied to Multiple Sclerosis Lesion Segmentation	IEEE Transactions on Medical Imaging		y	T1, T2, PD, FLAIR	Artificial neural networks	BE:BET; IN:0-1 range; REG:rigid; cropping	GeForce GTX 780	<1 sec	n	n	MS	-	43; 21; 195x2+n (377)	1 (MS GC MICCAI'08); 1 (ISBI'15); 3	y	Semiautomatic		DSC; VD; TPR; FPR
J	2016	D. Pustina et al.	Automated segmentation of chronic stroke lesions using LINDA: Lesion identification with neighborhood data analysis	Human Brain Mapping	Segmentation failure (low DSC)	n	T1	Random Forest	denoising:edge-preserving anisotropic algorithm; BFC:N4; BE:antsBrainExtraction	Xeon E4-2450, 2.1GHz 25GB RAM	3 hrs	n	n	S	S	60; 45	3	y	Manual, 1		DSC; Hdff; Sen; PPV; VC; other
J	2016	M. Goetz et al.	DALSA: Domain Adaptation for Supervised Learning From Sparsely Annotated MR Images	IEEE Transactions on Medical Imaging		y	T1, T1c, FLAIR, DTI	Random Forest	REG:rigid; BE; IN	GeCONiI	70 sec	n	y	BT	HGG	19; 30	1 (BraTS'13); 3	y	Manual, 1		DSC; Sen; Spe
J	2016	K. Thiruvenkadam and N. Perumal	Fully automatic method for segmentation of brain tumor from multimodal magnetic resonance images using wavelet transformation and clustering technique	International Journal of Imaging Systems and Technology		y	T1c, T2, FLAIR	Fuzzy C-Means, Discrete Wavelet Transform	sharpening:Haar WT	Intel Pentium Core Duo 1.6 GHz 512 MB RAM	2-3 min	n	n	BT	LGG; HGG	30	1 (BraTS'12)	y	-		DSC; Sen; PPV; Kappa
J	2016	R. Mechrez et al.	Patch-based Segmentation with Spatial Consistency: Application to MS Lesions in Brain MRI	Journal of Biomedical Imaging		y	T1, T2, FLAIR	K-Nearest Neighbours, Thresholding	subsampling; IN: histogram matching; BFC:N4; BE:BrainSuite13; tissue maps	i7 3.4GHz 8GB RAM	4 min	n	n	MS	-	65	1 (MS GC MICCAI'08)	y	-		TPR; PPV; DSC
J	2016	P. Korfiatis et al.	Automated Segmentation of Hyperintense Regions in FLAIR MRI Using Deep Learning	Tomography	Inter-rater agreement evaluated	n	FLAIR	Artificial neural networks	BE:atlas-based; BFC:N4; IN:histogram matching	NVIDIA K2	1 hr	n	n	BT	LGG; HGG	186; 135	1 (BraTS'15); 3		Manual, 3		DSC; Jcc; FPR; TPR
J	2016	A. M. Pagnozzi et al.	Automated, quantitative measures of grey and white matter lesion burden correlates with motor and cognitive function in children with unilateral cerebral palsy	NeuroImage: Clinical		y	T1, T2	Expectation-Maximization, Markov Random Field, Thresholding	BFC:N4; REG:affine; denoising:ADF; BE:in-house; tissue probability maps reg:free-form	n/a	n/a	n	n	CP	-	125	3 (University of Queensland Cerebral Palsy and Research Rehabilitation Centre)	y	Manual		Sen; Spe; Acc; FPR; FNR
J	2016	X. Qu et al.	Positive Unanimous Voting Algorithm for Focal Cortical Dysplasia Detection on Magnetic Resonance Image	Front. Comput. Neurosci		n	T1	Bayesian classification, Other	IN:histogram matching + 0-225 range; BE:BET; BFC:EM; resampling; tri-linear interpolation; REG:rigid; masking	n/a	n/a	n	n	FCD	-	10 + 31 controls	3 (Ghent University Hospital)	n	Manual, 1		TPR; FPR; F-score
J	2016	S. Banerjee et al.	A Novel GBM Saliency Detection Model Using Multi-Channel MRI	PloS one		y	T1c T2, FLAIR	Other	n/a	I7 3.4GHz 16GB RAM	n/a	n	n	BT	LGG; HGG	30 +50 simulated	1 (BraTS'12)	y	-		ROC
J	2016	L. Zhao and K. Jia	Multiscale CNNs for Brain Tumor Segmentation and Diagnosis	Computational and Mathematical Methods in Medicine		y	T1, T1c, T2, FLAIR	Artificial neural networks	n/a	n/a	n/a	n	n	BT	LGG; HGG	30 +50 simulated	1 (BraTS'13)	y	-		DSC
J	2016	P. G. L. Freire and R. J. Ferrari	Automatic iterative segmentation of multiple sclerosis lesions using Student's t mixture models and probabilistic anatomical atlases in FLAIR images	Computers in Biology and Medicine	Inter-rater agreement evaluated	n	FLAIR	Other	denoising:non-local means; BFC:N4; REG:non-rigid	i7 16GB RAM	~20 min	n	n	MS	-	5x4/5	1 (MSC'15)	y	-		DSC; Sen; FPR; VD; VC
J	2016	S. Pereira et al.	Brain Tumor Segmentation Using Convolutional Neural Networks in MRI Images	IEEE Transactions on Medical Imaging		y	T1, T1c, T2, FLAIR	Artificial neural networks	BFC:N4; IN:histogram matching	n/a	n/a	n	n	BT	LGG; HGG	65; 327	1 (BraTS'13); 1 (BraTS'15)	y	-		DSC; PPV; Sen
J	2016	N. Cordier et al.	A Patch-Based Approach for the Segmentation of Pathologies: Application to Glioma Labelling	IEEE Transactions on Medical Imaging		y	T1, T1c, T2, FLAIR	Other	REG:affine; resampling; IN:1-99% clipping + u & v scaling; tissue maps:FSL FAST	2.66GHz 6GB RAM	up to 16 min	n	n	BT	LGG; HGG	304?	1 (BraTS'13); 1 (BraTS'14)	y	-		DSC; Hdff
J	2016	M. J. Fartaria et al.	Automated detection of white matter and cortical lesions in early stages of multiple sclerosis	Journal of Magnetic Resonance Imaging		y	T1, FLAIR, MP2RAGE, DIR	K-Nearest Neighbours, Region Growing	REG:rigid; BE:MorphoBox; BFC:N4; IN:histogram mtaching; tissue maps	n/a	n/a	n	n	MS	MS	39	3	n	Manual, 2	consensus	DSC; Sen; Spe; Acc; Bland-Altman; other
J	2016	J. C. Griffis et al.	Voxel-based Gaussian naïve Bayes classification of ischemic stroke lesions in individual T1-weighted MRI scans	Journal of Neuroscience Methods		n	T1	Bayesian classification	tissue map:SPM12; BFC:SPM12; REG:SPM12	n/a	n/a	n	y	S	S	30	3 (Cincinnati Children's Hospital Medical Center)	y	Semiautomatic		DSC; VD; VC
J	2016	Y. Li et al.	Brain tumor segmentation from multimodal magnetic resonance images via sparse representation	Artificial Intelligence in Medicine		y	T1, T1c, T2, FLAIR	Sparse representation, Markov Random Field, Graph Cut,	BFC:N3; IN:histogram matching + 0-1 range	i7 1.8GHz 64 GB RAM	2-5 min	n	n	BT	LGG; HGG	64	1 (BraTS'13)	y	-		DSC; PPV; Sen; Kappa
J	2016	J. R. Stone et al.	Supervised learning technique for the automated identification of white matter hyperintensities in traumatic brain injury	Brain Injury		y	T1, T2, FLAIR	Random Forest, Markov Random Field	convert to nii; REG:rigid; denoising:ref; BFC:N4; IN:0-1 range; BE:ANTs	n/a	n/a		y	WML	TBI	24	3 (Chronic Effects of Neurotrauma Consortium, Walker et al. 2016)	n	Manual, 1		Sen; PPV; F1; VD
J	2015	V. G. Kanas et al.	A low cost approach for brain tumor segmentation based on intensity modeling and 3D Random Walker	Biomedical Signal Processing and Control	Segmentation failure	y	T1, T1c, T2, FLAIR	Random Walker	denoising; BFC; REG:FSL; BE:BET	3GHz 8GB RAM	<1 min	n	n	BT	LGG; HGG	34; 23	1 (BraTS'13); 3 (Prastawa et al. 2009); 3	y	Manual, 1		FPR; TRP; DSC; Hdff
J	2015	O. Commowick et al.	Diffusion MRI abnormalities detection with orientation distribution functions: A multiple sclerosis longitudinal study	Medical Image Analysis		n	DTI	Other	Movement correction; distortion correction; tensor and ODF estimation	n/a	n/a	n	n	MS	CIS	15x2	3	n	Manual, 1		other
J	2015	D. Guo et al.	Automated lesion detection on MRI scans using combined unsupervised and supervised methods	BMC Medical Imaging		n	T1	Support Vector Machine, Other	enantiomorphic normalization	I7 2.2GHz 16GB RAM	388 min	n	n	S; BT	S; G	60; 30	1 (BraTS'12); 3	y	Manual, 3	consensus	Acc; PPV; Sen; DSC
J	2015	P. K. Roy et al.	Automatic white matter lesion segmentation using contrast enhanced FLAIR intensity and Markov Random Field	Computerized Medical Imaging and Graphics		y	T1, FLAIR	Random Forest, Markov Random Field	REG:rigid(SPM8); BE:BET; BFC:N3	n/a	n/a	n	n	WML	HT	24; 20	1 (MS GC MICCAI'08); 3 (ENVISion, Reid et al. 2012)	y	Manual, 2		FROC; DSC; TPR; PPV
J	2015	E. Roura et al.	A toolbox for multiple sclerosis lesion segmentation.	Neuroradiology		y	T1, FLAIR	Thresholding	BE:BET; denoising:ADF;BFC:N3;REG:affine(SPM8/12)	n/a	n/a	n	y	MS	CIS	70; 20 (+23 test online); 14	1 (MS GC MICCAI'08); 3 (Hospital Vall d'Hebron, Spain); 3 (Clínica Girona, Spain)	y	Manual, multiple		DSC; TPR; PPV

J	2015	N. Guizard et al.	Rotation-invariant multi-contrast non-local means for MS lesion segmentation	NeuroImage: Clinical		y	T2, FLAIR	Non-local Means	denoising:NLM; BFC:N3; IN: histogram matching; REG:rigid; BE:ref	i7 3.06 GHz	40 min	n	planned	MS	-	108; 20 (+23 test online)	1 (MS GC MICCAI'08); 3	y	Semiautomatic		DSC; Sen; PPV; VDf; FPR; SSD; TPR; PPV; VR
J	2015	S. Jain et al.	Automatic segmentation and volumetry of multiple sclerosis brain lesions from MR images	NeuroImage: Clinical	Scan-rescan reproducibility	y	T1, FLAIR	Other	REG:rigid; BE:MNI brain mask reg(affine+non-rigid); tissue maps	n/a	n/a	n	n	MS	-	20; 10x2	3 (VU University Medical Center, Amsterdam, the Netherlands); 3 (UZ Brussels, Brussels, Belgium)	y	Manual, 3	consensus	DSC; ICC; absVD; Sen; PPV
J	2015	R. Harmouche et al.	Probabilistic Multiple Sclerosis Lesion Classification Based on Modeling Regional Intensity Variability and Local Neighborhood Information	IEEE Transactions on Biomedical Engineering		y	T1, T2, PD, FLAIR	Other	BFC:N3; REG:ref; BE:BET; IN:histogram matching	Optiplex 980 quad-core	2 min	n	n	MS	-	100	3	y	Manual, 1&5	majority	DSC; Sen; PPV
J	2015	Z. Karimaghaloo et al.	Temporal Hierarchical Adaptive Texture CRF for Automatic Detection of Gadolinium-Enhancing Multiple Sclerosis Lesions in Brain MRI	IEEE Transactions on Medical Imaging		y	T1, T1c, T2, PD, FLAIR	Conditional Random Field	REG:ref; BE:BET; BFC:N3; IN:histogram matching	2.66GHz	56 sec	treatment; placebo	n	MS	-	1190x2; 120x2; 69x4	3	y	Manual, 2	consensus	Sen; FPR
J	2015	T. Zhan et al.	A novel brain tumor segmentation method for multi-modality human brain MRIs	International Journal of Multimedia and Ubiquitous Engineering		y	T1, T1c, T2, FLAIR	Bayesian classification, Logistic Regression	REG:rigid; BFC:N3; BE	n/a	n/a	n	n	BT	-	10 +10 simulated	1 (BraTS'12)	y	-		Jcc
J	2015	M. P. Arakeri and G. R. M. Reddy	Computer-aided diagnosis system for tissue characterization of brain tumor on magnetic resonance images	SIViP	Inter-rater agreement evaluated	y	T1c, T2	Fuzzy C-Means	denoising:median filtering; contrast enhnacement:histogram equalization; REG:FLIRT; BE:Otsu's thresholding	3GHz 3GB RAM	1 sec	benign, malignant	n	BT	-	550	3 (Shirdi Sai Cancer Hospital, Manipal, India)	n	Manual, 2		Hdff; VO; avgSD
J	2015	A. Demirhan et al.	Segmentation of Tumor and Edema Along With Healthy Tissues of Brain Using Wavelets and Neural Networks	EEE journal of biomedical and health informatics		y	T1, T2, FLAIR	Self Organising Maps	BE: thresholding + morphological operations; IN:0-1 range; denoising:ADF; REG:affine	n/a	n/a	n	n	BT	G	20	1 (BraTS'12); 3 (Mayıs Hospital in Ankara, Turkey)	y	Manual		DSC; Sen; Spe
J	2015	C. H. Sudre et al.	Bayesian Model Selection for Pathological Neuroimaging Data Applied to White Matter Lesion Segmentation	IEEE Transactions on Medical Imaging		y	T1, FLAIR; T2, PD, FLAIR	Expectation-Maximization Gausian mixture model, Markov Random Field	BFC:in house; REG; IN:0-1 range; BE:ref	n/a	n/a	n	n	WML	MS; DM	20	1 (MS GC MICCAI'08)	y	-		DSC; TPR; FNR; VD; AD; DE; OER
J	2015	B. Ahmed et al.	Cortical feature analysis and machine learning improves detection of 'MRI-negative' focal cortical dysplasia	Epilepsy & Behavior		n	T1	Linear regression, Bagging	BFC; BE:ref; FreeSurfer	n/a	n/a	n	n	FCD	FCD	31 (24 MRI negative) + 62 controls	3 (New York University School of Medicine Comprehensive Epilepsy Center)	n	Manual, 2	supervision	TPR; FPR; DSC; avgSD
J	2015	S. D. S. Al-Shaikhli et al.	Brain tumor classification and segmentation using sparse coding and dictionary learning	Biomedical Engineering / Biomedizinische Technik		n	FLAIR	Graph Cut, Dictionary Learning	BE:BET; BFC:MIPAV	n/a	n/a	normal, abnormal	n	BT	GBM; G; MET	30; 30; 30; 30	1 (BraTS'13); 1 (IBSR); 1 (SPL)	y	-		DSC; PPV; Sen; Jcc; Kappa
J	2015	P. Szwarc et al.	Automatic brain tumour detection and neovasculature assessment with multiseries MRI analysis	Computerized Medical Imaging and Graphics	Inter-rater agreement evaluated	y	T1, T1c, FLAIR, RCBV	Fuzzy C-Means	REG; interpolation: nearest neighbour; BE:Otsu's thresholding; symmetry plane detection	n/a	n/a	n	n	BT	-	10; 10	3	y	Manual, 1&2		DSC; Sen; Spe
J	2015	J. Juan-Albarracín et al.	Automated Glioblastoma Segmentation Based on a Multiparametric Structured Unsupervised Classification	PloS one		y	T1, T1c, T2, FLAIR	Markov Random Field	denoising:ref; BE:Brain Suite Software; BFC:N4; resampling:ref	Intel Xeon E5-2620 64GB RAM	~39 min	n	n	BT	-	31	1 (BraTS'13)	y	-		DSC; PPV; Sen; Kappa
J	2015	B. Ozenne et al.	Spatially regularized mixture model for lesion segmentation with application to stroke patients	Biostatistics		y	T1, FLAIR	Markov Mixture Models	REG; tissue maps	n/a	15 min	n	upon request	S	S	12	3 (I-KNOW)	n	Manual, 3	majority	other
J	2015	X. Tomas-Fernandez and S. K. Warfield	A Model of Population and Subject (MOPS) Intensities With Application to Multiple Sclerosis Lesion Segmentation	IEEE Transactions on Medical Imaging		y	T1, T2, FLAIR	Expectation-Maximization Gausian mixture model, Graph Cut	BFC:ref; denoising:ref	n/a	n/a	n	n	MS	MS	3 (synthetic); 51	1 (MS GC MICCAI'08)	y	-		TPR; FPR; Extra Fraction; Miss Fraction; PPV
J	2015	I. Njeh et al.	3D multimodal MRI brain glioma tumor and edema segmentation: A graph cut distribution matching approach	Computerized Medical Imaging and Graphics		y	T1, T1c, T2, FLAIR	Other, Graph Cut	BRATS only	n/a	<0.5 sec	n	n	BT	LGG; HGG	45 +65 simulated	1 (BraTS'12)	y	-		DSC
J	2015	O. Maier et al.	Extra Tree forests for sub-acute ischemic stroke lesion segmentation in MR sequences	Journal of Neuroscience Methods	Errors in preprocessing, Segmentation failure	y – adaptable to single and multi	T1, T2, FLAIR, DWI	Random Forest	resampling; REG:rigid(elastix); BE:BET; BFC:Bies Field Correction Tool; IN:histogram matching	n/a	n/a	n	n	S	S	37	3 (Machner et al. 2012)	n	Manual, 1		DSC; avgSSD; Hdff; PPV; Sen
C	2015	O. Maier et al.	MS lesion segmentation in MRI with random forests	2015 Longitudinal Multiple Sclerosis Lesion Segmentation Challenge	Inter-rater agreement evaluated	n/a	n/a	Random Forest	IN:MedPy	n/a	n/a	n	n	MS	-	5x4	1 (MSC'15)	n	-		DSC; avgSSD; TPR. FPR
J	2015	T. C. Steed et al.	Iterative Probabilistic Voxel Labeling: Automated Segmentation for Analysis of The Cancer Imaging Archive Glioblastoma Images	American Journal of Neuroradiology	Inter-rater agreement evaluated	y	T1, T1c, T2, FLAIR	K-Nearest Neighbours, Thresholding	BFC:FAST; REG:nonlinear + affine; BE:BET+ROBEX; tissue maps	n/a	11 min	n	n	BT	-	40	2 (TCIA, Clark et al. 2013)	y	Manual, 3	supervision	DSC
J	2015	R. Wang et al.	Automatic segmentation and volumetric quantification of white matter hyperintensities on fluid-attenuated inversion recovery images using the extreme value distribution	Neuroradiology		n	FLAIR	Other	BFC:N3; IN:0-255 range; BE:BET	n/a	n/a	n	planned	WML	MS	60, 10	1 (MS GC MICCAI'08); 3	y	Manual, 2	consensus	DSC; FPR; FNR; OER; DER; Bland-altman; ICC; VC; SI
J	2015	P. Maji and S. Roy	SoBT-RFW: Rough-Fuzzy Computing and Wavelet Analysis Based Automatic Brain Tumor Detection Method from MR Images	Fundamenta Informaticae		n/a	n/a	Fuzzy C-Means, Region Growing	BE:S3	3.4GHz 16GB RAM	n/a	n	y	BT	-	25	1 (BraTS'12)	y	-		DSC; Sen; Spe
J	2015	N. Nabizadeh and M. Kubat	Brain tumors detection and segmentation in MR images: Gabor wavelet vs. statistical features	Computers & Electrical Engineering	Detection followed by segmentation	n	T1/FLAIR	Comparison	IN; histogram normalization	3GHz 64GB RAM	~5 min	n	n	BT	HGG	25 (unclear how many real and how many simulated)	1 (BraTS'13)	y	-		Sen; Spe; Acc

J	2014	N. Gonçalves et al.	Self-supervised MRI tissue segmentation by discriminative clustering	Int. J. Neur. Syst		y	T1, T2, PD; T1 T2, FLAIR	Self Organising Maps	REG:SPM5; BE:BET2	n/a	n/a	n	n	MS	MS	2; 54	1 (MS GC MICCAI'08)	y	-		DSC; other
J	2014	Y. Zhong et al.	Automated White Matter Hyperintensity Detection in Multiple Sclerosis Using 3D T2 FLAIR	Journal of Biomedical Imaging		n	FLAIR	Other	BE:in-house; BFC:modified homodyne HP filter; tissue maps:thresholding	I7 2.8GHz 8GB RAM	5 min	n	n	MS	-	26	3 (Synergy Health Concepts, CA)	n	Manual		SI; VC
J	2014	Y.-H. Mah et al.	A new method for automated high-dimensional lesion segmentation evaluated in vascular injury and applied to the human occipital lobe	Cortex		n	DWI	Other	REG:SPM5	n/a	n/a	n	y	S	-	38 + 95 controls	3	n	Manual, 1		DSC; Sen; Spe
J	2014	B. I. Yoo et al.	Application of variable threshold intensity to segmentation for white matter hyperintensities in fluid attenuated inversion recovery magnetic resonance images	Neuroradiology		n	FLAIR	Thresholding	BFC:SPM8; BE:SPM8	n/a	n/a	n	n	WML	-	48	3	y	Manual, 2		DSC; Sen; Spe; Jcc; ICC
J	2014	M. Cabezas et al.	Automatic multiple sclerosis lesion detection in brain MRI by FLAIR thresholding	Computer Methods and Programs in Biomedicine		n	FLAIR	Expectation-Maximization, Thresholding	BE:BET; denoising:ADF; BFC:N4; REG:affine	QuadCore 2.3 GHz, 16 Gb RAM	several min	n	n	MS	-	45	3 (Hospital Vall d'Hebron); 3 (Hospital Josep Trueta); 3 (Clínica Girona)	y	Semiautomatic		TPR; FPR; DSC; avgSD
J	2014	J.-Z. Tsai et al.	Automated Segmentation and Quantification of White Matter Hyperintensities in Acute Ischemic Stroke Patients with Cerebral Infarction	PloS one		y	T1, DWI, FLAIR	Thresholding, Other	REG:rigid(MNI); tisue maps:SPM8; IN:1-100 range; BE:BET	i5 2.67GHz 4GB RAM	~15 min	n	n	WML	S	30	3 (Taiwan Stroke Registry, Hsieh, et al. 2010)	n	Semiautomatic		SI; ICC; Bland-Altman; Sen; Spe
J	2014	P. Dvořák et al.	Unsupervised Pathological Area Extraction using 3D T2 and FLAIR MR Images	Measurement Science Review	No preprocessing	y	T2, FLAIR	Other	Mid-sagittal plane extraction	n/a	<1 min	n	n	BT	LGG; HGG	30 +50 simulated	1 (BraTS'12)	y	-		DSC; Acc
J	2014	Y. Lu et al.	Multimodal Brain-Tumor Segmentation Based on Dirichlet Process Mixture Model with Anisotropic Diffusion and Markov Random Field Prior	Computational and Mathematical Methods in Medicine		y	T1c, FLAIR	Markov Random Field, Other	denoising: ADF	n/a	n/a	n	n	BT	LGG; HGG	55 + 65 synthetic	1 (BraTS'12)	y	-		DSC; Jcc; Sen; Spe
J	2014	R. Wang et al.	Automatic segmentation of white matter lesions on magnetic resonance images of the brain by using an outlier detection strategy	Magnetic Resonance Imaging		y – adaptable to single and multi	T1, T2, FLAIR	Expectation-Maximization Gausian mixture model, Other	BFC:N3; BE:BET; REG:demons registration	n/a	2.2 sec per slice	n	n	WML	-	86 + synthetic	3	n	Manual, 2		DSC; Overlap Fraction; Extra Fraction; VC; Band-Altman
J	2014	M. Huang et al.	Brain Tumor Segmentation Based on Local Independent Projection-Based Classification	IEEE Transactions on Biomedical Engineering		y	T1, T1c, T2, FLAIR	Other	BFC:N3; IN:1-99% clipping + 0-100 range	I5 3.1GHz	26 min	n	n	BT	LGG; HGG	55 +65 synthetic	1 (BraTS'12); 1 (BraTS'13)	y	-		DSC; Jcc; FPR; FNR
J	2014	J. Mitra et al.	Lesion segmentation from multimodal MRI using random forest following ischemic stroke	NeuroImage		y	T1, T2, FLAIR, DWI (ADC)	Markov Random Field, Random Forest	REG:rigid; tissue masks:ref; BE: masking	3.2GHz 23.5GB RAM	27 min	n	n	S, WML	S	36	1 (MS GC MICCAI'08); 3	y	Manual, 1		DSC; PPV; TPR; avgSSD; VD
J	2014	J.-Z. Tsai et al.	Automatic Detection and Quantification of Acute Cerebral Infarct by Fuzzy Clustering and Histographic Characterization on Diffusion Weighted MR Imaging and Apparent Diffusion Coefficient Map	BioMed Research International		n	DWI (ADC)	Fuzzy C-Means, Thresholding, edge detection,	reg:rigid + trilinear interpolation NMI; IN:0-1 range; BE:BET; histogram smoothing:3rd order moving avg filter	i5 2.67GHz 4GB RAM	5 min	n	n	S	S	22	3 (Taiwan Stroke Registry, Hsieh, et al. 2010)	n	Semiautomatic		DSC; Kappa; ICC; Sen; Spe; PPV
J	2014	J. Zhang et al.	A fully automatic extraction of magnetic resonance image features in glioblastoma patients	Med Phys		y	T1, T1c, FLAIR	Random Forest	REG:rigid (FLIRT); denoising:median filtering	n/a	n/a	n	n	BT	GBM	73	2 (TCIA, Clark et al. 2013)	n	Manual, 1		DSC
J	2014	O. Ganiler et al.	A subtraction pipeline for automatic detection of new appearing multiple sclerosis lesions in longitudinal studies	Neuroradiology		y	T1, T2, PD	Thresholding, Other	BE:BET; REG:affine + rigid; BFC:N4; IN:histogram matching; tissue map	n/a	n/a	n	n	MS	MS	20x2	3	n	Manual, 2	supervision	Sen; FPR; DSC
J	2014	G. Erus et al.	Individualized statistical learning from medical image databases: Application to identification of brain lesions	Medical Image Analysis	Inter-rater agreement evaluated	n/a	n/a	Other	BE:BET; BFC: N3; IN:histogram matching; REG:non-lin(HAMMER)	n/a	n/a	n	n	WML, S	DM	80	3	n/a	Manual, 1&2		DSC; Sen; Spe
J	2014	E. M. Sweeney et al.	A Comparison of Supervised Machine Learning Algorithms and Feature Vectors for MS Lesion Segmentation Using Multimodal Structural MRI	PloS one	Raters' years of experience given	y	T1, T2, FLAIR	Comparison	REG:rigd; BFC:N3; BE:SPECTRE; CSF removal:15th percentile	1 core	<1 min	n	n	MS	MS	98	3	y	Manual, 2	supervision	ROC; DSC
J	2013	G. Liberman et al.	Automatic multi-modal MR tissue classification for the assessment of response to bevacizumab in patients with glioblastoma	European Journal of Radiology	Treatment response evaluation	y	T1, T1c, T2, T2*, T2*c, FLAIR	Expectation-Maximization Gausian mixture model, K-Nearest Neighbours	BFC:N3; REG:SPM; IN:multiplicative model; BE:BET	n/a	n/a	n	n	BT	GB	59	3	n	Manual		Sen; Spe; VC; other
J	2013	E. M. Sweeney et al.	Automatic Lesion Incidence Estimation and Detection in Multiple Sclerosis Using Multisequence Longitudinal MRI	American Journal of Neuroradiology		y	T1, T2, PD, FLAIR	Other, Logistic Regression	resampling; REG: rigid; BE:ref; tissue maps	n/a	n/a	n	n	MS	-	10x11	3	n	Manual, 1		ROC; Volume Change
J	2013	S. Ji et al.	Automatic segmentation of white matter hyperintensities by an extended FitzHugh & nagumo reaction diffusion model	Journal of Magnetic Resonance Imaging		n	FLAIR	Other	BE:BET; denoising:ADF	n/a	n/a	n	n	WML	-	127	3	y	Manual, 3	consensus	DSC
J	2013	C. Elliott et al.	Temporally Consistent Probabilistic Detection of New Multiple Sclerosis Lesions in Brain MRI	IEEE Transactions on Medical Imaging		y	T1, T2, PD, FLAIR	Bayesian classification, Markov Random Field	BE:BET; BFC:N3; REG:rigid; IN:histogram matchingl + least trimmed squares	n/a	n/a	n	n	MS	-	684 (155 participants)	3	y	Semiautomatic		Sen; FD
J	2013	E. M. Sweeney et al.	OASIS is Automated Statistical Inference for Segmentation, with applications to multiple sclerosis lesion segmentation in MRI	NeuroImage: Clinical	Inter-rater agreement evaluated	y	T1, T2, PD, FLAIR	Logistic Regression	REG:rigid; BFC:N3; BE:SPECTRE; IN:0u1v; smoothing	n/a	n/a	n	n	MS	-	98 + 33 healthy; 20x2, 128 + 1 healthy	3	y	Manual, 1		ROC; Sen; DSC
J	2013	K. Mouridsen et al.	Acute Stroke: Automatic Perfusion Lesion Outlining Using Level Sets	Radiology	Inter-rater agreement evaluated	y	PWI, DWI	Level Set	REG:linear	n/a	n/a	n	n	S	S	14	3	n	Manual, 4	consensus	Bland – Altman; DSC
J	2013	A. Resmi et al.	A novel automatic method for extraction of glioma tumour, white matter and grey matter from brain magnetic resonance images	Biomedical Imaging and Intervention Journal	Inter-rater agreement evaluated	y	T1, FLAIR, T2	Thresholding, Other	IN:0-1 range; BE:morphological operations	n/a	1 sec	n	n	BT	LGG, HGG	20 +50 synthetic	3 (Sree Chitra Institute of Medical Sciences and Technology); 3 (Regional Cancer Centre, Thiruvananthapuram, Kerala, India)	y	Manual, 1		PPV; Jcc; other
J	2013	A. Bijar et al.	Increasing the Contrast of the Brain MR FLAIR Images Using Fuzzy Membership Functions and Structural Similarity Indices in Order to Segment MS Lesions	PloS one		n	FLAIR	Thresholding, Genetic Algorithm	n/a	2.5 GHz 512 MB RAM	<30 sec	n	n	MS	MS	20	3 (Khayati et al. 2008)	n	Manual, 2	consensus	DSC; Sen; Extra Fraction; ICC
J	2013	R. Simões et al.	Automatic segmentation of cerebral white matter hyperintensities using only 3D FLAIR images	Magnetic Resonance Imaging		n	FLAIR	Expectation-Maximization Gausian mixture model	BE:BET; BFC:FAST	n/a	n/a	n	n	WML	MCI; progressive MCI	25 + 15 healthy; 23	1 (MS GC MICCAI'08); 3 (University Hospital of Essen, Germany)	y	Manual, 1		DSC; Overlap Fraction; Extra Fraction; VC
J	2013	L. Shi et al.	Automated quantification of white matter lesion in magnetic resonance imaging of patients with acute infarction	Journal of Neuroscience Methods		y	T1, FLAIR, DWI	Other	REG:affine; IN:in house; BE:BET; tissue maps:REF	2.8GHz 12GB RAM	25 sec	n	n	WML	S	91(30 segmented)	3	y	Semiautomatic		DSC; Sen; PPV; ICC; Bland-Altman
J	2012	L. Weizman et al.	Automatic segmentation, internal classification, and follow-up of optic pathway gliomas in MRI	Medical Image Analysis	Indirect reproducibility evaluation	y	T1, T2, FLAIR	Other	REG:SPM; IN:dynamic histogram warping	n/a	n/a	n	n	BT	optic pathway G	28	3	n	Manual, 2		VD; avgSSD; DSC; ROC
J	2012	Y. Wang et al.	Multi-stage segmentation of white matter hyperintensity, cortical and lacunar infarcts	NeuroImage		y	T1, T2, FLAIR	Expectation-Maximization Gausian mixture model, Region Growing	BFC:REF; BE:BET; REG: FLIRT; tissue map:FreeSurfer	n/a	n/a	WMH; CI; LI	n	WML	-	272	3 (Memory Aging & Cognition Center at the National University of Singapore)	n	Manual, 1-3	supervision	DSC; VC; Bland-Altman

J	2012	K. H. Ong et al.	Automatic white matter lesion segmentation using an adaptive outlier detection method	Magnetic Resonance Imaging		y	T1, FLAIR; T1, T2, FLAIR	Thresholding	BE:model-based level set; BFC:N3	n/a	n/a	n	n	WML	-	38; 23	1 (MS GC MICCAI'08); 3 (Universiti Sains Malaysia's hospital)	y	Manual, 1		VR; Jcc; DSC; Sen; FPR; VD
J	2012	T. Samaille et al.	Contrast-Based Fully Automatic Segmentation of White Matter Hyperintensities: Method and Validation	PloS one		y	T1, FLAIR	Thresholding	BE:SPM8; REG:rigid; BFC:SPM8	n/a	30 min	n	n	WML	MCI; CADASIL	24; 43	3 (Salpêtrière Hospital); 3 (Lariboisière Hospital)	y	Semiautomatic		ICC; Bland-Altman; VR; DSC
J	2012	B. A. Abdullah et al.	Multi-Sectional Views Textural Based SVM for MS Lesion Segmentation in Multi-Channels MRIs	Open Biomed Eng J		y	T1, T2, FLAIR	Support Vector Machine	Resampling; REG:AIR; IN:ref	n/a	n/a	n	n	MS	-	51 (+synthetic); 10	1 (MS GC MICCAI'08); 3 (University of Miami)	y	n/a		DSC; TPR; PPV; other
J	2012	P. Schmidt et al.	An automated tool for detection of FLAIR-hyperintense white-matter lesions in Multiple Sclerosis	NeuroImage		y	T1, FLAIR	Region Growing, Partial Volume Estimation	BFC:VBM8; tissue maps; REG:linear + non-linear	3.2 GHz 8GB RAM	13 min	n	planned	MS	-	52 + 10 (unclear)+ 18 control	3	n	Manual, 2	consensus	VC; VR; Sen; Spe; Acc; DSC
J	2012	A. Gooya et al.	GLISTR: Glioma Image Segmentation and Registration	IEEE Transactions on Medical Imaging		y	T1, T1c, T2, FLAIR	Expectation-Maximization, tumor growth, atlas registration,	BFC; BE; cerebelum removal; REG:affine	n/a	3-6 hs	n	n	BT	-	122 (10 segmented)	3 (University of Pennsylvania)	n	Manual, 1		DSC; SD
C	2014	D. Kwon et al.	Multimodal brain tumor image segmentation using glistr	MICCAI BRATS		y	T1, T1c, T2, FLAIR	Expectation-Maximization, tumor growth, atlas registration,	BFC; IN:0-255 range	I7 3.4 GHz	85 min	n	n	BT	LGG, HGG	35	1 (BraTS'13)	y	-		DSC; PPV; Sen
J	2012	K. Popuri et al.	3D variational brain tumor segmentation using Dirichlet priors on a clustered feature set	Int J CARS		y	T1, T1c, T2	Active Contour, Other	denoising:edge-preserving non-linear filter; IN:weighted least squares; BFC:N3; symmetrical alignment:rigid	n/a	n/a	n	n	BT	G	15	3	n	Manual, 1		Jcc; PPV; Sen; Hdff
J	2012	N. M. Saad et al.	Fully Automated Region Growing Segmentation of Brain Lesion in Diffusion-weighted MRI	IAENG International Journal of Computer Science		n	DWI	Thresholding, Region Growing, Other,	IN:0-1 range + gamma-law transformation; BE:thresholding + morphological operations	n/a	n/a	n	n	S, BT, abscess	-	23 (slices used)	3 (General Hospital of Kuala Lumpur)	n	Manual		Jcc; FPR; FNR; other
J	2012	M. de C. Alegro et al.	Computerized brain tumor segmentation in magnetic resonance imaging	Einstein (São Paulo)	Semiautomatic preprocessing	y	T1, T1c, FLAIR	Support Vector Machine	BFC:N3; denoising:ADF; REG:semiautomatic; BE:ref; IN:histogram matching	n/a	n/a	n	n	BT	T	11	3 (Universidade de São Paulo)	n	Manual 2		other
J	2012	A. Khademi et al.	Robust White Matter Lesion Segmentation in FLAIR MRI	IEEE Transactions on Biomedical Engineering		n	FLAIR	Thresholding, Partial Volume Estimation	BE:ref; bilateral filtering	n/a	n/a	n	n	WML	-	24	3	n	Manual, 1		DSC; Sen; Spe
C	2015	A. Khademi et al.	Multiscale Partial Volume Estimation for Segmentation of White Matter Lesions Using Flair MRI	2015 IEEE 12th International Symposium on Biomedical Imaging (ISBI)		n	FLAIR	edge content, Partial Volume Estimation	n/a	n/a	n/a	n	n	WML	-	30	3 (Khademi et al. 2012)	n/a	Manual, 1		DSC; Extra Fraction; Bland-Altman
J	2012	Zacharaki, E. and Bezerianos A.	Abnormality Segmentation in Brain Images Via Distributed Estimation	IEEE Transactions on Information Technology in Biomedicine		n/a		Other	BE:ref; BFC:ref; IN:histogram matching, 0-1 range + 0u1v; REG:deformable	Intel Core2 Duo CPU 2GHz	Min – hrs	n	n	WML	DM	33 +73 healthy	3 (ACCORD-MIND)	n/a	Manual, 1		ROC
J	2011	D. Garcia-Lorenzo et al.	Trimmed-Likelihood Estimation for Focal Lesions and Tissue Segmentation in Multisequence MRI for Multiple Sclerosis	IEEE Transactions on Medical Imaging	Inter-rater agreement evaluated	y	T1, T2, PD	Other	BFC:ref; reg:rigid; BE:BET	2.4GHz 4GB RAM	2 min	n	n	MS	MS	10; simulated	1 (BrainWeb); 3	n	Manual, 5	majority	DSC; ICC
C	2008	D. Garcia-Lorenzo et al.	A Robust Expectation-Maximization Algorithm for Multiple Sclerosis Lesion Segmentation	MICCAI Workshop: 3D Segmentation in the Clinic: A Grand Challenge II, MS Lesion Segmentation		y	T1, T2, FLAIR	Expectation-Maximization, outlier detection	BFC:ref; BE:BET	n/a	n/a	n	n	MS	MS	44	1 (MS GC MICCAI'08)	y	-		VD; AD; TP; FP
J	2011	E. Geremia et al.	Spatial decision forests for MS lesion segmentation in multi-channel magnetic resonance images	NeuroImage		y	T1, T2, FLAIR	Decision Tree	resampling; cropping; BFC:REF; IN:REF; REF:ref; tissue maps:INRIA	IBM e325 dual-Opterons246 2 GHz	n/a	n	n	MS	MS	45	1 (MS GC MICCAI'08)	y	-		TPR; PPV; VD; SD
C	2012	E. Geremia et al.	Spatial decision forests for glioma segmentation in multi-channel MR images	MICCAI Challenge on Multimodal Brain Tumor Segmentation		y	T1, T1c, T2, FLAIR	Decision Tree	IN:histogram matching	n/a	n/a	n	n	BT	LGG, HGG	30 (+25 synthetic)	1 (BrainWeb); 3	y	n/a		DSC; TPR; PPV
J	2011	N. Zhang et al.	Kernel feature selection to fuse multi-spectral MRI images for brain tumor segmentation	Computer Vision and Image Understanding		y	T2, PD, FLAIR	Support Vector Machine, Region Growing	REG:SPM	n/a	n/a	n	n	BT	-	8+3x5(?)	3	y	Manual, multiple		TP;FP; FN; other
J	2011	S. Jeon et al.	Fully automated pipeline for quantification and localization of white matter hyperintensity in brain magnetic resonance image	International Journal of Imaging Systems and Technology		y	T1, FLAIR	Other	BFC:N3; BE:BET; REG:affine; tissue masks:INSECT	n/a	n/a	n	n	WML	SVD	10 annotated (+35 not)	3 (Lee et al. 2011)	n	Manual, 1		DSC; Jcc; Bland-Altman; VR
J	2011	V. Harati et al.	Fully automated tumor segmentation based on improved fuzzy connectedness algorithm in brain MR images	Computers in Biology and Medicine		n	T1c	Other	denoising:ADF; head mask:thresholding; IN:0-255 range	3.2GHz, 1GB RAM	2.5 min	n	n	BT	T	10	3	n/a	Manual, 1		DSC; Spe; Extra fraction
J	2010	D. Selvathi et al.	Hybrid approach for brain tumor segmentation in magnetic resonance images using cellular neural networks and optimization techniques	International Journal of Computational Intelligence and Applications		y	T2, FLAIR	Artificial neural networks, Genetic Algorithm	IN:-1-1 range	3GHz 512MB RAM	n/a	n	n	BT	astrocytoma; MET; G; meningioma	30	3	n	Manual		Acc
J	2010	M. Scully et al.	An automated method for segmenting white matter lesions through multi-level morphometric feature classification with application to lupus	Frontiers in Human Neuroscience		y	T1, T2, FLAIR	Support Vector Machine	resampling:trilinear interpolation; REG:MI; BFC:ref; IN:histogram matching	n/a	n/a	n	n	WML	lupus	27	3 (The Mind Research Network, University of New Mexico Hospital)	n	Manual, multiple		Sen; Spe
J	2010	N. Shiee et al.	A topology-preserving approach to the segmentation of brain images with multiple sclerosis lesions	NeuroImage		y- adaptable	T1, FLAIR	Fuzzy Clustering, Other	REG:rigid	modern Linux workstation	~45 min	n	y	MS	MS	10	1 (BrainWeb); 3	n	Semiautomatic		DSC; TP; VC
J	2009	K. E. Emblem et al.	Automatic glioma characterization from dynamic susceptibility contrast imaging: Brain tumor segmentation using knowledge-based fuzzy clustering	Journal of Magnetic Resonance Imaging	Inter-rater agreement evaluated	y	T1c, T2, FLAIR	Fuzzy C-Means, Thresholding	REG:NMI (SPM5); IN:adaptive histogram equalization; BE:SPM5	Pentium 4 3.19GHz 3GB RAM	4 min	Survival: ow-risk; high-risk	n	BT	G	50	3	n	Manual, 4	majority	Sen; PPV; VC; ROC
J	2009	R. Beare et al.	Development and validation of morphological segmentation of age-related cerebral white matter hyperintensities	NeuroImage		y	T1, T2, FLAIR	boosting, Watershed	REG:SPM5; BFC:ref; IN:median and interquartile range scaling; tissue map	n/a	n/a	n	n	WML	-	247	3 (Tasmanian Study of Cognition and Gait)	n	Manual, 1; Semiautomatic		ICC; SI
J	2009	R. de Boer et al.	White matter lesion extension to automatic brain tissue segmentation on MRI	NeuroImage	Inter-rater agreement evaluated	y	T1, PD, FLAIR	K-Nearest Neighbours, Thresholding	REG:rigid; resampling; BE:ELASTIX; BFC:N3; IN:4-96% clipping + 0-1 range	n/a	n/a	n	n	WML	-	195 + 20	3 (Rotterdam Scan Study, de Leeuw et al. 2001)	n	Manual, 1&2		DSC; Sen; Extra Fraction; VD; other
J	2009	J. Nie et al.	Automated brain tumor segmentation using spatial accuracy-weighted hidden Markov Random Field	Computerized Medical Imaging and Graphics	Visual validation of preprocessing	y	T1, T2, FLAIR	Expectation-Maximization-Markov Random Field	REG; BE:BET	3GHz 2GB RAM	20-25 min	n	n	BT	G	15	3	n	Semiautomatic		Jcc; VO
J	2009	A. Akselrod-Ballin et al.	Automatic Segmentation and Classification of Multiple Sclerosis in Multichannel MRI	IEEE Transactions on Biomedical Engineering	Indirect reproducibility evaluation	y – adaptable to single and multi	T1, T2, PD and/or FLAIR	Decision Tree, Other	REG:during acquisition	Xeon PC 1.7GHz	<10 min	n	n	MS	MS	25 + 4x4	3 (cientific Institute Ospedale San Raffaele)	n	Manual, 2	consensus	DSC; Sen; Spe; Acc; VC
J	2008	R. Khayati et al.	Fully automatic segmentation of multiple sclerosis lesions in brain MR FLAIR images using adaptive mixtures method and markov random field model	Computers in Biology and Medicine		n	FLAIR	Markov Random Field, Mixture Model	IN:0-255 range; BE:in house	2.66GHz 512 MB RAM	21 sec	n	n	MS	-	20	3	n	Manual, 2		F1; Sen: Extra fraction; VC
J	2008	R. Khayati et al.	A novel method for automatic determination of different stages of multiple sclerosis lesions in brain MR FLAIR images	Computerized Medical Imaging and Graphics		n	FLAIR	Markov Random Field, Mixture Model	BE:ref	Pentium 4 2.66GHz 512 MB RAM	n/a	acute (recent/ early); chronic	n	MS	MS	20	3	n	Manual, 2		DSC; Sen; Extra Fraction
J	2008	T. B. Dyrby et al.	Segmentation of age-related white matter changes in a clinical multi-center study	NeuroImage		y – adaptable to single and multi	T1, T2, FLAIR	Artificial neural networks	IN:polynomial fitting & z-score; REG:rigid(SPM2); resampling:b-spline interpolation; BE:SPM2; BFC:N3	n/a	n/a	n	n	WML	-	362	3 (LADIS, Pantoni et al. 2005)	y	Semiautomatic		SI; Sen

J	2008	J. F. A. Jansen et al.	White Matter Lesions in Patients With Localization-Related Epilepsy	Investigative Radiology		y	T1, T2, FLAIR	Other	REG; IN; BE:ref; tissue maps	n/a	n/a	n	n	WML	EP	33 + 16 healthy	3	n	Expert segmentation		ICC; VC
J	2008	E. Herskovits et al.	Automated Bayesian Segmentation of Microvascular White-Matter Lesions in the ACCORD-MIND Study	Advances in medical sciences	Semiautomatic preprocessing (brain extraction)	y	T1, T2, FLAIR, SD	Bayesian classification, Other	REG: rigid; BE:ref; tissue maps; IN:histogram matching; smoothing:Gaussian kernel	Silicon Graphics (Mountain View, CA) Origin 300 workstation	4 min	n	n	WML	-	42	3 (ACCORD-MIND)	y	Manual, 1		TPR; FPR; DSC; ROC
J	2008	J. J. Corso et al.	Efficient Multilevel Brain Tumor Segmentation With Integrated Bayesian Model Classification	IEEE Transactions on Medical Imaging		y	T1, T1c, T2, FLAIR	Other	REG:FSL; denoising:FSL; BE:FSL; IN:FSL	1.67 Gz 1.5 GB	<2 min	n	n	BT	GBM	20	3	n/a	Expert segmentation		Jcc
J	2005	F. Admiraal-Behloul et al.	Fully automatic segmentation of white matter hyperintensities in MR images of the elderly	NeuroImage	Inter-rater agreement evaluated, Scan-rescan reproducibility	y	PD; T2, FLAIR	Fuzzy C-Means	REG:rigid(AIR); tissue maps: affine transformation from MNI; BE:FCM	Pentium 4 512MB RAM	2 min	n	n	WML	-	100; 44 (different slice thickness)	3 (PROSPER, Shepherd et al. 1999)	n	Manual, 2		ICC; Bland-Altman; DSC
J	2005	O. Colliot et al.	Segmentation of focal cortical dysplasia lesions on MRI using level set evolution	NeuroImage	Inter-rater agreement evaluated, Segmentation failure	n	T1	Level Set	IN; BFC; REG; BE: BET; tissue maps:thresholding	1.6GHz	14 min	n	n	FCD	FCD	24	3	n	Manual, 2	consensus	SI; FP; other
J	2004	W. Li et al.	Robust unsupervised segmentation of infarct lesion from diffusion tensor MR images using multiscale statistical classification and partial volume voxel reclassification	Computational Diffusion MRI	Inter-rater agreement evaluated	n	DTI	Other	BFC & denoising:ADF	n/a	n/a	n	n	S	-	20	3	y	Manual		DSC
J	2004	P. Anbeek et al.	Automatic segmentation of different-sized white matter lesions by voxel probability estimation	Medical Image Analysis		y	T1, IR, T2, PD, FLAIR	K-Nearest Neighbours, Thresholding	IN:histogram matching; REG:rigid; BE:Mbrase	n/a	n/a	n	n	WML	Arterial VaD	19	3	n	Manual, 2	supervision	TPR; FPR; DSC; overlap fraction; error fraction; other
J	2002	A. P. Zijdenbos et al.	Automatic 'pipeline' analysis of 3-D MRI data for clinical trials: application to multiple sclerosis	IEEE Transactions on Medical Imaging		y	T1, T2, PD	Artificial neural networks	IN:adaptive classification; denoising:ADF; BFC:N3; REG; resampling	n/a	n/a	n	n	MS	-	10; 29	3 (Weiner et al. 1997)	y	Manual, 7; Semiautomatic		other
J	2001	K. Van Leemput et al.	Automated segmentation of multiple sclerosis lesions by model outlier detection	IEEE Transactions on Medical Imaging	Inter-rater agreement evaluated	y	PD, T2	Other	REG:affine; BFC:EM	n/a	n/a	n	n	MS	-	10x2 + 3 (HiRes)	3 (BIOMORPH, 1996–1998)	n	Manual, 1&2		DSC; VC
J	1998	L. P. Clarke et al.	MRI Measurement of Brain Tumor Response: Comparison of Visual Metric and Automatic Segmentation	Magnetic Resonance Imaging		y	T1, T2, PD	Fuzzy C-Means	n/a	n/a	n/a	n	n	BT	-	6x(2-5)	3	n	Manual, multiple		VC
J	1997	S. Dickson et al.	Using Neural Networks to Automatically Detect Brain Tumours in MR Images	International Journal of Neural Systems		n	T1c	Artificial neural networks	n/a	n/a	n/a	n	n	BT	Acoustic neuroma	50	3	n	Manual		other
J	1995	M. Kamber et al.	Model-based 3-D segmentation of multiple sclerosis lesions in magnetic resonance brain images	IEEE Transactions on Medical Imaging	Inter-rater agreement evaluated, Segmentation failure, Manual corrections of preprocessing	y	PD, T2	Other	BE; BFC:homomorphic filtering	n/a	n/a	n	n	MS	MS	12; 44	3	n	Manual, 1		other
C	2019	S. N. Shivhare et al.	An Efficient Brain Tumor Detection and Segmentation in MRI Using Parameter-Free Clustering	Machine Intelligence and Signal Analysis		n	T1c	K-Means, Other	n/a	n/a	n/a	n	n	BT	HGG	273	1 (BraTS'15)	y	-		DSC
C	2018	S. M. Anwar et al.	Brain tumor segmentation on Multimodal MRI scans using EMAP Algorithm	IEEE EMBC		y	T2; FLAIR	K-Means, Expectation-Maximization	BFC:N4r; IN:0-1 range	i7 16BG RAM	comparable to previous techniques	n	n	BT	LGG; HGG	40	1 (BraTS'15)	y	-		DSC; Jcc; PPV; Sen; Kappa
C	2018	D. Jin et al.	White matter hyperintensity segmentation from T1 and FLAIR images using fully convolutional neural networks enhanced with residual connections	ISBI		y	T1, FLAIR	Artificial neural networks	IN:0-1 range; tissue map	n/a	n/a	n	n	WML	-	170	1 (WMH Segmentation Challenge '17)	y	-		DSC; Hdff; avgVD; Sen; F1
C	2018	A. F. I. Osman	Automated Brain Tumor Segmentation on Magnetic Resonance Images and Patient's Overall Survival Prediction Using Support Vector Machines	Brainlesion: Glioma, Multiple Sclerosis, Stroke and Traumatic Brain Injuries		y	T1, T1c, T2, FLAIR	Support Vector Machine	IN:max global intensity + thresholding; denoising:median filtering	n/a	n/a	Overall survival:short, medium, long	n	BT	LGG; HGG	477	1 (BraTS'17)	y	-		DSC
C	2018	Y. Jiang et al.	A Brain Tumor Segmentation New Method Based on Statistical Thresholding and Multiscale CNN	Intelligent Computing Methodologies		y	T1, T1c, T2, FLAIR	Artificial neural networks, Thresholding	denoising:median filtering	n/a	n/a	n	n	BT	LGG; HGG	274	1 (BraTS'15)	y	-		DSC; PPV; Sen
C	2018	L. Chen et al.	MRI tumor segmentation with densely connected 3D CNN	Medical Imaging 2018: Image Processing		y	T1, T1c, T2, FLAIR	Artificial neural networks	BFC:N4; IN:0u1v	n/a	n/a	n	n	BT	-	210	1 (BraTS'17)	y	-		DSC
C	2018	Y. Xu et al.	White Matter Hyperintensities Segmentation in a Few Seconds Using Fully Convolutional Network and Transfer Learning	Brainlesion: Glioma, Multiple Sclerosis, Stroke and Traumatic Brain Injuries		y	T1, FLAIR	Artificial neural networks	BFC; morphological operations	NVIDIA GeForce GTX 1080	10 sec	n	n	WML	-	60 + 110 (testing)	1 (WMH Segmentation Challenge '17)	y	-		DSC; Hdff; avgVD; Sen; F1

Gryska E, et al. BMJ Open 2022; 11:e042660. doi: 10.1136/bmjopen-2020-042660

C	2018	M. Soltaninejad et al.	MRI Brain Tumor Segmentation and Patient Survival Prediction Using Random Forests and Fully Convolutional Networks	Brainlesion: Glioma, Multiple Sclerosis, Stroke and Traumatic Brain Injuries		y	T1, T2, FLAIR	Artificial neural networks, Random Forest	IN:1-99% clipping + 0u1v + histogram matching	i7 16BG RAM GTX980i	n/a	overall survival; long; medium; short	n	BT	-	497	1 (BraTS'17)	y	-		DSC; Sen; Spe; Hdff
C	2017	M. Bento et al.	Probabilistic Segmentation of Brain White Matter Lesions Using Texture-Based Classification	Image Analysis and Recognition		y	T1, T2, DWI, FLAIR	Thresholding, Support Vector Machine, K-Nearest Neighbours, Random Forest	resizing; tissue maps:semi-automatic region growing; IN:0-255 range	n/a	n/a	n	n	S	S	28	1 (ISLES'15)	y	-		DSC; Sen; Spe; Acc
C	2018	M. Bento et al.	WMH Segmentation Challenge: A Texture-Based Classification Approach	Brainlesion: Glioma, Multiple Sclerosis, Stroke and Traumatic Brain Injuries		y	T1, FLAIR	Random Forest	cropping; resizing; tissue map; IN:0-255 range	Intel Zeon 12-Core 2.7 GHz 64GB DDR3 RAM	90 sec	n	n	WML	-	60 + 110 (testing)	1 (MS GC MICCAI'08)	y	-		DSC; Hdff; avgVD; Sen; F1
C	2018	M. Shaikh et al.	Brain Tumor Segmentation Using Dense Fully Convolutional Neural Network	Brainlesion: Glioma, Multiple Sclerosis, Stroke and Traumatic Brain Injuries		y	T1, T1c, T2, FLAIR	Artificial neural networks	IN:z-score	Titan X GPU	<30 sec	n	n	BT	LGG; HGG	284; 29	1 (BraTS'17)	y	-		DSC
C	2018	Y. Hu and Y. Xia	3D Deep Neural Network-Based Brain Tumor Segmentation Using Multimodality Magnetic Resonance Sequences	Brainlesion: Glioma, Multiple Sclerosis, Stroke and Traumatic Brain Injuries		y	T1, FLAIR	Artificial neural networks	IN:0-255 range	Intel Xeon 2.10 GHz CPU, NVIDIA GTX 1080 Ti GPU	3 min	n	n	BT	LGG; HGG	477	1 (BraTS'17)	y	-		DSC; Hdff
C	2018	Z. Kapás et al.	Automatic Brain Tumor Segmentation in Multispectral MRI Volumes Using a Random Forest Approach	Image and Video Technology		y	T1, T1c, T2, FLAIR	Random Forest	IN:linear transformation	I7 3.4GHz	45 sec	n	n	BT	HGG	220	1 (BraTS'16)	y	-		DSC; Sen; Spe
C	2018	L. Vidyaratne et al.	Deep learning and texture-based semantic label fusion for brain tumor segmentation	Proceedings of SPIE--the International Society for Optical Engineering		y	T1, T1c, T2, FLAIR	Artificial neural networks, Random Forest	BFC:N4; IN:ref	n/a	n/a	n	n	BT	HGG	243	1 (BraTS'17)	y	-		DSC
C	2018	S. Doyle et al.	Sub-acute and Chronic Ischemic Stroke Lesion MRI Segmentation	Brainlesion: Glioma, Multiple Sclerosis, Stroke and Traumatic Brain Injuries		y	T1, FLAIR	Other	IN: to a reference image; BFC:N4; REG:ITK; tissue maps:ITK	n/a	n/a	n	n	S	-	37; 47	1 (Maier et al. 2015); 3 (HERMES)	y	Manual, 1		DSC; Hdff; avgSD; PPV; Sen; VD
C	2018	S. Amiri et al.	Bayesian Network and Structured Random Forest Cooperative Deep Learning for Automatic Multi-label Brain Tumor Segmentation	International Conference on Agents and Artificial Intelligence		y	T1, T1c, FLAIR	Artificial neural networks, Random Forest	BFC:N4; IN:histogram linear transformation	n/a	n/a	n	n	BT	HGG	220	1 (BraTS'15)	y	-		DSC
C	2018	G. C. Oliveira et al.	Brain Tumor Segmentation in Magnetic Resonance Images using Genetic Algorithm Clustering and AdaBoost Classifier	BIOIMAGING			n/a	boosting, Genetic Algorithm	n/a	I7 2.4GHz 8GB RAM	6.22 min	n	n	BT	LGG	42	1 (BraTS'15)	y	-		DSC
C	2018	G. Wang et al.	Automatic Brain Tumor Segmentation Using Cascaded Anisotropic Convolutional Neural Networks	Brainlesion: Glioma, Multiple Sclerosis, Stroke and Traumatic Brain Injuries		y	T1, T1c, T2, FLAIR	Artificial neural networks	n/a	NVIDIA TITAN X GPU	n/a	n	n	BT	LGG; HGG	285; 46; 146	1 (BraTS'17)	y	-		DSC; Hdff
C	2018	D. Bhattacharya and N. Sinha	An improved approach of high graded glioma segmentation using sparse autoencoder and fuzzy c-means clustering from multi-modal MR images	SPIE Medical Imaging		y	T1, T1c, T2, FLAIR	Artificial neural networks	none	n/a	n/a	n	n	BT	HGG	15	1 (BraTS'15)	y	-		DSC; Jcc
C	2018	H. N. Bharath et al.	Tumor Segmentation from Multimodal MRI Using Random Forest with Superpixel and Tensor Based Feature Extraction	Brainlesion: Glioma, Multiple Sclerosis, Stroke and Traumatic Brain Injuries		y	T1, T1c, T2, FLAIR	Random Forest	IN:0-1 range + histogram equalization; backgound removal:Otsu's thresholding	n/a	n/a	n	n	BT	HGG	285; 46; 146	1 (BraTS'17)	y	-		DSC; Sen; Hdff
C	2018	R. Pourreza et al.	Brain Tumor Segmentation in MRI Scans Using Deeply-Supervised Neural Networks	Brainlesion: Glioma, Multiple Sclerosis, Stroke and Traumatic Brain Injuries		y	T1c, T2, FLAIR	Artificial neural networks	BFC:N4; IN:histogram mathcing	NVIDIA Titan Xp	30 sec	n	n	BT	LGG; HGG	285	1 (BraTS'17)	y	-		DSC
C	2018	M. F. Rachmadi et al.	Automatic Irregular Texture Detection in Brain MRI Without Human Supervision	Medical Image Computing and Computer Assisted Intervention – MICCAI 2018		n	FLAIR	Other	BE:optiBET, tissue map:in-house	GPU	13 min	n	n	WML	-	20	2 (ADNI)	y	n/a		DSC
C	2017	C. Ö. Fiçici et al.	Fully Automated Brain Tumor Segmentation and Volume Estimation Based on Symmetry Analysis in MR Images	CMBEBIH		y	T1, T1c, FLAIR	Fuzzy C-Means, Other	denoising:median filtering; cropping; BE:thresholding	n/a	n/a	n	n	BT	-	10	3	n	Expert segmentation		DSC; Jcc; Sen; Spe;
C	2017	S. R. Mote et al.	Non-negative matrix factorization and self-organizing map for brain tumor segmentation	2017 International Conference on Wireless Communications, Signal Processing and Networking (WiSPNET)		y	T2, FLAIR	Self Organising Maps	BFC & denoising:ADF	n/a	n/a	n	n	BT	LGG; HGG	30 (+25 synthetic)	1 (BraTS'12)	y	-		DSC; Sen; Spe
C	2017	A. López-Zorrilla et al.	Brain White Matter Lesion Segmentation with 2D/3D CNN	Natural and Artificial Computation for Biomedicine and Neuroscience		y	T1, FLAIR, DTI	Artificial neural networks	REG:affine; IN:0-1 range	GTX 1070 16GB RAM	n/a	n	n	WML	AD, VaD	18	3 (Price et al. 2012)	n	Manual		TPR
C	2017	H. Shen et al.	Multi-task Fully Convolutional Network for Brain Tumour Segmentation	Medical Image Understanding and Analysis		y	T1, T1c, T2, FLAIR	Artificial neural networks	n/a	n/a	n/a	n	n	BT	HGG	30; 220	1 (BraTS'13); 1 (BraTS'15)	y	-		DSC; Sen; PPV; F-score

C	2017	S. Pereira et al.	On hierarchical brain tumor segmentation in MRI using fully convolutional neural networks: A preliminary study,	ENBENG		y	T1, T1c, T2, FLAIR	Artificial neural networks	IN:histogram normalization + 0u1v	I7 3.2GHz 48GB RAM GTX970 GPU	40 sec	n	n	BT	-	40	1 (BraTS'13)	y	-		DSC; PPV; Sen
C	2017	H. Dong et al.	Automatic Brain Tumor Detection and Segmentation Using U-Net Based Fully Convolutional Networks	Medical Image Understanding and Analysis		y	T1c, FLAIR	Artificial neural networks	IN:0u1v	Titan X GPU 12Gb RAM	3 sec	n	n	BT	LGG; HGG	274	1 (BraTS'15)	y	-		DSC; Sen
C	2017	M. Rezaei et al.	Deep Neural Network with l2-Norm Unit for Brain Lesions Detection	Neural Information Processing		y	T1,T2, DWI, FLAIR	Artificial neural networks	n/a	n/a	n/a	LGG; HGG, AD; S	n	BT, S	LGG; HGG; S	328; 30	1 (BraTS'16); 1 (ISLES'16)	y	-		DSC
C	2017	S. Hussain et al.	Brain tumor segmentation using cascaded deep convolutional neural network	Annual International Conference of the IEEE		y	T1, T1c, T2, FLAIR	Artificial neural networks	BFC:N4; IN:0u1v	n/a	n/a	n	n	BT	LGG; HGG	30 +25 synthetic	1 (BraTS'13)	y	-		DSC; Sen; Spe
C	2017	W. Chen et al.	Automatic brain tumor segmentation based on features of separated local square	Chinese Automation Congress		n	FLAIR	Support Vector Machine	BFC:N4; IN:histogram matching	n/a	n/a	n	n	BT	LGG; HGG	20 (randomly selected from 30)	1 (BraTS'13)	y	-		DSC; Sen; Spe
C	2017	G. Latif et al.	Automatic Multimodal Brain Image Classification Using MLP and 3D Glioma Tumor Reconstruction	IEEE-GCC Conference and Exhibition		y	T1, T1c, T2, FLAIR	Other, Active Contour	denoising: ADF; BE:thresholding + morphologica operations	n/a	n/a	normal, abnormal	n	BT	LGG; HGG	160	1 (BraTS'15)	y	-		DSC; Spe; Sen; Acc; other
C	2017	W. Mengqiao et al.	The multimodal brain tumor image segmentation based on convolutional neural networks	IEEE International Conference on Computational Intelligence and Applications		y	T1, T1c, T2, FLAIR	Artificial neural networks	BFC:N4; IN:0u1v	I7 2.8GHz GPU GeForce 1050	n/a	n	n	BT	LGG; HGG	120	1 (BraTS'15)	y	-		DSC; PPV; Sen
C	2017	V. Shreyas et al.	A deep learning architecture for brain tumor segmentation in MRI images	International Workshop on Multimedia Signal Processing		y	T1, T1c, T2, FLAIR	Artificial neural networks	BFC:N4; IN:histogram matching + z-score	i7 128GB RAM	13 sec	n	n	BT	HGG	220	1 (BraTS'15)	y	-		DSC; PPV; Sen
C	2017	N. Shah et al.	Brain tumor segmentation and classification using cascaded random decision forests	International Conference on Electrical Engineering/Electronics, Computer, Telecommunications and Information Technology		y	T1, T1c, T2, FLAIR	Random Forest	IN:histogram matching	n/a	n/a	n	n	BT	LGG; HGG	30 +50 simulated	1 (BraTS'13)	y	-		DSC; Jcc
C	2017	U. Baid et al.	Novel approach for brain tumor segmentation with non negative matrix factorization	International Conference on Innovations in Electronics, Signal Processing and Communication		y	T2, FLAIR	Fuzzy C-Means, Matrix Factorization	denoising:ADF; BFC:N3	n/a	n/a	n	n	BT	LGG; HGG	30 +50 simulated	1 (BraTS'12)	y	-		DSC; Sen; Spe; Hdff
C	2017	M. K. Akter et al.	Automated brain tumor segmentation from mri data based on exploration of histogram characteristics of the cancerous hemisphere	IEEE Region 10 Humanitarian Technology Conference		y	T1, T1c, T2, FLAIR	Thresholding, Other	n/a	n/a	n/a	n	n	BT	HGG	15	1 (BraTS'12)	y	-		DSC; Sen; Spe
C	2017	S. Goel et al.	Brain tumor segmentation in multispectral MR images	International Conference on Signal Processing and Integrated Networks		y	T1, T1c, T2, FLAIR	Fuzzy C-Means	BFC:N3; IN:thresholding + contrast enhancement, image fusion; smoothing	n/a	n/a	n	n	BT	HGG	15	1 (BraTS'13)	y	-		DSC; PPV; Sen
C	2017	A. Sankari and S. Vigneshwari	Automatic tumor segmentation using convolutional neural networks	International Conference on Science Technology Engineering Management		n/a	n/a	Artificial neural networks	denoising; IN; BFC; contrast enhancement:histogram equalization	n/a	n/a	n	n	BT	LGG; HGG	391	1 (BraTS'15); 1 (IBSR)	y	-		Sen; Spe; acc
C	2016	R. Liu et al.	Multi-modal Brain Tumor Segmentation Based on Self-organizing Active Contour Model	Chinese Conference on Pattern Recognition		y	T1, T1c, T2, FLAIR	Self Organising Maps, Active Contour	IN:histogram normalization; contrast adjustment:ITKSnap	n/a	n/a	n	n	BT	LGG; HGG	45	1 (BraTS'13); 1 (BraTS'15)	y	-		DSC; Sen; Spe
C	2016	Y. Wang et al.	A deep symmetry convnet for stroke lesion segmentation	IEEE ICIP		n	T1	Artificial neural networks	BFC:AFNI; BE:SymROBEX; IN:0u1v; contrast enhancement:histogram equalization	n/a	n/a	n	n	S	Aphasia due to S	18	3	n	Manual		DSC
C	2016	B. Song et al.	Anatomy-Guided Brain Tumor Segmentation and Classification	Brainlesion: Glioma, Multiple Sclerosis, Stroke and Traumatic Brain Injuries		y	T1, T1c, T2, FLAIR	Random Forest, Grow Cut	BFC:N4; IN:linear normalization of tissue histograms	2.67GHz 24 GB RAM	1 min	n	n	BT	LGG; HGG	274	1 (BratTS'15); 1 (BraTS'16)	y	-		DSC; Hdff
C	2016	E. Ilunga-Mbuyamba et al.	Automatic brain tumor tissue detection based on hierarchical centroid shape descriptor in Tl-weighted MR images	2016 International Conference on Electronics, Communications and Computers (CONIELECOMP)		n	T1	K-Means, Other	BE: thresholding + morphological operations	1.5GHz 2GB RAM	n/a	n	n	BT	-	254	3 (University Hospital, Department of Neurosurgery, University of Leipzig, Germany)	n	Expert segmentation		DSC; Jcc
C	2016	M. Agn et al.	Brain Tumor Segmentation Using a Generative Model with an RBM Prior on Tumor Shape	Brainlesion: Glioma, Multiple Sclerosis, Stroke and Traumatic Brain Injuries		y	T1, T1c, T2, FLAIR	Artificial neural networks	BFC:log transform	i7 CPU, GTX Titan Black GPU	30 min	n	n	BT	LGG; HGG	65; 297	1 (BraTS'13); 1 (BraTS'15)	y	-		DSC
C	2015	M. Havaei et al.	A Convolutional Neural Network Approach to Brain Tumor Segmentation	Brainlesion: Glioma, Multiple Sclerosis, Stroke and Traumatic Brain Injuries		y	T1, T1c, T2, FLAIR; T1, DWI, FLAIR	Artificial neural networks	n/a	Nvidia Titan black GPU	25 sec – 3 min	n	n	BT, S	LGG; HGG; S	40; 327; 114	1 (BraTS'13); 1 (BraTS'15); 1 (ISLES'15)	y	-		DSC; Sen; Spe; avgSD; Hdff
C	2016	X. Chen et al.	Automated brain tumor segmentation using kernel dictionary learning and superpixel-level features	IEEE SMC		y	T1, T1c, T2, FLAIR	Sparse representation, Graph Cut	BFC:N4; IN:histogram matching	I7 3.2GHz 32GB RAM	30 sec	n	n	BT	HGG	20; 10	1 (BraTS'13)	y	-		DSC; Jcc; Sen
C	2016	Q. Mahmood and A. Basit	Prediction of Ischemic Stroke Lesion and Clinical Outcome in Multi-modal MRI Images Using Random Forests	Brainlesion: Glioma, Multiple Sclerosis, Stroke and Traumatic Brain Injuries		y	DWI, PWI	Random Forest	n/a	15 2.5GHz	5 min	clinical outcome – medical ranking score	n	S	S	49	1 (ISLES'16)	y	-		avgSSD; Hdff; DSC
C	2015	Q. Mahmood and A. Basit	Automatic ischemic stroke lesion segmentation in multi-spectral mri images using random forests classifier	MICCAI BrainLes		y	T1, T2, DWI, FLAIR	Random Forest	BFC:N3; IN:0-1 range	I5 2.5 GHz 4GB RAM	~30 min	n	n	S	S	64	1 (ISLES'15)	y	-		avgSSD; Hdff; DSC; PPV; Sen
C	2016	K. Vaidhya et al.	Multi-modal Brain Tumor Segmentation Using Stacked Denoising Autoencoders	Brainlesion: Glioma, Multiple Sclerosis, Stroke and Traumatic Brain Injuries		n/a	n/a	Artificial neural networks	IN:histogram matching + mean division and outlier removal	n/a	30 min	n	n	BT	LGG; HGG	274	1 (BraTS'15)	y	-		DSC
C	2016	S. Amiri et al.	Deep random forest-based learning transfer to SVM for brain tumor segmentation	International Conference on Advanced Technologies for Signal and Image Processing		n	FLAIR	Random Forest, Support Vector Machine	IN; contrast enhancement	n/a	n/a	n	n	BT	HGG	20	1 (BraTS'13)	y	-		DSC
C	2016	S. Müller et al.	Automatic brain tumor segmentation with a fast Mumford-Shah algorithm	SPIE Medical Imaging		y	T1c, T2, FLAIR	Thresholding, Other	BFC:N4; IN histogram equalization + 0-225 range	I7 3.4GHz 16BG RAM GTX 970 4GB RAM	<1 min	n	n	BT	LGG; HGG	213	1 (BraTS'14)	y	-		DSC; Sen; PPV
C	2016	E. A. R. Piedra et al.	Brain Tumor Segmentation by Variability Characterization of Tumor Boundaries	Brainlesion: Glioma, Multiple Sclerosis, Stroke and Traumatic Brain Injuries		y	T1, T1c, T2, FLAIR	Other	tissue maps	n/a	n/a	n	n	BT	-	220	1 (BraTS'15)	y	-		DSC; Hdff
C	2016	Z. Xiao et al.	A deep learning-based segmentation method for brain tumor in MR images	International Conference on Computational Advances in Bio and Medical Sciences		n/a	n/a	Artificial neural networks	cropping; IN	n/a	n/a	n	n	BT	-	10	3 (West China Hospital)	n	Manual		percentage match
C	2016	A. Sehgal et al.	Automatic brain tumor segmentation and extraction in MR images	Conference on Advances in Signal Processing		n	T1	Fuzzy C-Means, Other	BFC:N3; denoising:ADF; enhancement:unsharp mask	n/a	n/a	n	n	BT	-	30	1 (BraTS'13)	y	-		DSC
C	2016	A. Derntl et al.	Stroke Lesion Segmentation Using a Probabilistic Atlas of Cerebral Vascular Territories	Brainlesion: Glioma, Multiple Sclerosis, Stroke and Traumatic Brain Injuries		y	not specific to a modality	Expectation-Maximization Gausian mixture model, Conditional Random Field, Other,	REG; BFC; BE	n/a	n/a	n	n	S	S	13	3	n/a	Manual		DSC

C	2015	Y. Li et al.	Automatic Brain Tumor Segmentation from MR Images via a Multimodal Sparse Coding Based Probabilistic Model	International Workshop on Pattern Recognition in NeuroImaging		y	T1, T1c, T2, FLAIR	Sparse representation, Markov Random Field	REG:rigid; BE; IN:0-1 range	n/a	n/a	n	n	BT	-	30	1 (BraTS'12)	y	-		DSC; Sen; Spe
C	2015	M. Lyksborg et al.	An Ensemble of 2D Convolutional Neural Networks for Tumor Segmentation	Scandinavian Conference on Image Analysis		y	T1, T1c, T2, FLAIR	Artificial neural networks	BFC:N4; IN:histogram scaling	n/a	n/a	n	n	BT	-	131; 187 (longitudinal)	1 (BraTS'14)	y	-		DSC; PPV; Sen
C	2015	R. Mechrez et al.	MS lesion segmentation using a multi-channel patch-based approach with spatial consistency	Medical Imaging 2015: Image Processing		y	T1, T2, FLAIR	Non-local Means, K-Nearest Neighbours, Other,	BE; BFC; sub-sampling; IN	n/a	n/a	n	n	MS	-	10	1 (MS GC MICCAI'08)	n	-		DSC; TPR; PPV
C	2015	I. Zabir et al.	Automatic brain tumor detection and segmentation from multi-modal MRI images based on region growing and level set evolution	2015 IEEE International WIE Conference on Electrical and Computer Engineering (WIECON-ECE)		y	T2, FLAIR	K-Nearest Neighbours, Thresholding, region growing, level set	n/a	n/a	n/a	n	n	BT	-	17	1 (BraTS'12)	y	-		DSC; Jcc; Sen; Spe
C	2015	A. Jog et al.	Multi-output decision trees for lesion segmentation in multiple sclerosis	Proceedings of SPIE--the International Society for Optical Engineering		y	T1, T2, FLAIR	Decision Tree	BFC:N4; BE:ref; REG:ref; IN	n/a	2 min	n	n	MS	-	20; 49	1 (MS GC MICCAI'08); 3	y	Manual		PPV; TPR
C	2015	H. Tang et al.	Tumor segmentation from single contrast MR images of human brain	2015 IEEE 12th International Symposium on Biomedical Imaging (ISBI)	Segmentation failure	n	T2	Random Forest	REG:rigid+affine; IN:linear scaling to MNI	n/a	n/a	n	n	BT	LGG; HGG	30	1 (BraTS'12)	y	-		DSC
C	2015	L. Szilágyi et al.	Automatic Brain Tumor Segmentation in Multispectral MRI Volumetric Records	Neural Information Processing		y	T1, T1c, T2, FLAIR	Fuzzy C-Means	n/a	n/a	n/a	n	n	BT	-	13	1 (BraTS'12)	y	-		Jcc; DSC
C	2015	Y. Karpate et al.	Probabilistic one class learning for automatic detection of multiple sclerosis lesions	2015 IEEE 12th International Symposium on Biomedical Imaging (ISBI)		y	T1, T2, FLAIR	Thresholding, probabilistic classification	n/a	n/a	n/a	n	n	MS	-	16 + 20 controls	3	n	Manual, 1		PPV; Sen
C	2015	H. Deshpande et al.	Adaptive dictionary learning for competitive classification of multiple sclerosis lesions	2015 IEEE 12th International Symposium on Biomedical Imaging (ISBI)		y	T1, T2, PD, FLAIR	Sparse representation, Dictionary Learning	BFC; denoising:non-local means; REG:BE; ref	n/a	n/a	n	n	MS	MS	13	3	n	Manual, 1		Sen; PPV
C	2015	I. Gondra and I. Cabria	Automated segmentation of brain tumors in MRI using potential field clustering	International Conference on Computer as a Tool		n	FLAIR	Region Growing, Other	n/a	n/a	n/a	n	n	BT	-	22	1 (BraTS'12)	y	-		other
C	2015	A. Pinto et al.	Brain Tumour Segmentation based on Extremely Randomized Forest with high-level features	Annual International Conference of the IEEE Engineering in Medicine and Biology Society (EMBC)		y	T1, T1c, T2, FLAIR	Decision Tree	BFC:ref; IN: histogram matching	n/a	n/a	n	n	BT	LGG; HGG	40	1 (BraTS'13)	y	-		DSC; PPV; Sen
C	2015	K. B. Vaishnavee and K. Amshakala	An automated MRI brain image segmentation and tumor detection using SOM-clustering and Proximal Support Vector Machine classifier	IEEE International Conference on Engineering and Technology		n	T1	Self Organising Maps, Support Vector Machine	BE:BET; enhancement:histogram equalization	n/a	n/a	n	n	BT	-	20	1 (IBSR); 3 (Medicine Service of the "Virgen de las Nieves" Hospital, Granada, Spain)	y	Manual		TI
C	2015	A. Pinto et al.	Random decision forests for automatic brain tumor segmentation on multi-modal MRI images	IEEE Portuguese Meeting on Bioengineering		y	T1, T1c, T2, FLAIR	Random Forest	BFC:N4; IN:histogram matching	I7 3.2GHz 47GB RAM	n/a	n	n	BT	LGG; HGG	30	1 (BraTS'13)	y	-		DSC; PPV; Sen
C	2015	Y. Renping et al.	Automatic Segmentation of White Matter Lesions Using SVM and RSF Model in Multi-channel MRI	Image and Graphics		y	T1, T2, PD, FLAIR	Support Vector Machine, Other	REG:rigid (MI); BE:ref; BFC:N3; IN:in paper	n/a	n/a	n	n	WML	-	45	3 (ACCORD-MIND)	n/a	Manual, 1		DSC; TPR; TNR; PPV
C	2015	N. Nabizadeh et al.	Automatic tumor lesion detection and segmentation using modified winnow algorithm	ISBI	Detection followed by segmentation	n	FLAIR	Active Contour, Level Set	IN:histogram normalization	n/a	n/a	n	n	BT	HGG	25 +25 simulated	1 (BraTS'13)	y	-		Acc
C	2015	S. E. El-Khamy et al.	An efficient brain mass detection with adaptive clustered based fuzzy C-mean and thresholding	International Conference on Signal and Image Processing Applications		n/a	n/a	Fuzzy C-Means	enhancement:intensity scaling	I5 2.3GHz 4GB RAM	~5 sec	n	n	BT	T	15	3	n/a	Expert segmentation		Acc, other
C	2015	A. F. Muda et al.	Integration of Fuzzy C-Means with Correlation Template and Active Contour for Brain Lesion Segmentation in Diffusion-Weighted MRI	International Conference on Artificial Intelligence, Modelling and Simulation		n	DWI	Fuzzy C-Means, Active Contour, Other,	Ref	n/a	n/a	n	n	BT, S	-	40	3	n/a	Manual		Jcc; DSC; FPR; FNR
J	2015	A. F. Muda et al.	Brain Lesion Segmentation Using Fuzzy C-Means On Diffusion-Weighted Imaging	Journal of Engineering and Applied Sciences		n	DWI	Fuzzy C-Means, Edge detection, Watershed	Ref	n/a	n/a	n	n	BT, S	T, S	20	3 (General Hospital of Kuala Lumpur)	y	Manual		FPR; FNR; DSC; other
C	2014	S. D. S. Al-Shaikhli et al.	Coupled Dictionary Learning for Automatic Multi-Label Brain Tumor Segmentation in Flair MRI images	Advances in Visual Computing		n	FLAIR; T1	Graph Cut, Dictionary Learning	n/a	2GHz CPU	n/a	n	n	BT	-	10; 30	1 (BraTS'13); 1 (SPL)	y	-		DSC; PPV; Sen; Jcc; Kappa
C	2014	H. J. Kuijf et al.	The Added Value of Diffusion Tensor Imaging for Automated White Matter Hyperintensity Segmentation	Computational Diffusion MRI		y	T1, FLAIR, DTI	K-Nearest Neighbours	IN:histogram matching; REG:rigid (elastix)	n/a	n/a	n	n	WML	-	20	3	n	Manual, 2	supervision	DSC; Sen
C	2014	P. K. Roy et al.	Automated Segmentation of White Matter Lesions Using Global Neighbourhood Given Contrast Feature-Based Random Forest and Markov Random Field	2014 IEEE International Conference on Healthcare Informatics		y	T1, FLAIR	Random Forest, Markov Random Field	REG:rigid(SPM8); BE:BET; BFC:N3	n/a	n/a	n	n	WML	HT	24	3 (ENVISion, Reid et al. 2012)	n	Manual, 2	supervision	DSC
C	2014	A. Rao et al.	Contusion segmentation from subjects with Traumatic Brain Injury: A random forest framework	2014 IEEE 11th International Symposium on Biomedical Imaging (ISBI)		y	T1, DTI, FLAIR	Random Forest	BFC:N4; IN:linear scaling; REG:affine; tissue maps	n/a	n/a	n	n	TBI	-	23	3	n	Manual, 1		DSC; TPR; PPV
C	2014	N. Subbanna et al.	Iterative Multilevel MRF Leveraging Context and Voxel Information for Brain Tumour Segmentation in MRI	IEEE Conference on Computer Vision and Pattern Recognition		y	T1, T1c, T2, FLAIR	Bayesian classification, Markov Random Field	BFC; IN	Dell Optiplex 980 I7	75 min	n	n	BT	LGG; HGG	30;25 (challenge sets)	1 (BraTS'12); 1 (BraTS'13)	y	-		DSC
C	2015	S. Reza et al.	Ischemic stroke lesion segmentation using local gradient and texture features	MICCAI ISLES		y	T1, T2, DWI, FLAIR	Random Forest	IN:histogram matching + CSF normalizaiton	n/a	n/a	n	n	S	S	28	1 (ISLES'15)	y	-		DSC
C	2014	S. Reza et al.	Improved brain tumor tissue segmentation using texture features	MICCAI 2014		y	T1, T1c, T2, FLAIR	Random Forest	BFC; IN	n/a	n/a	n	n	BT	LGG; HGG	246	1 (BraTS'13); 1 (BraTS'14)	y	-		DSC; Jcc; PPV; Sen; Kappa
C	2014	H. Hooda et al.	Brain tumor segmentation: A performance analysis using K-Means, Fuzzy C-Means and Region growing algorithm	IEEE International Conference on Advanced Communications, Control and Computing Technologies		n/a	n/a	Fuzzy C-Means	BC: median filtering; BE: Otsu's thresholding + morphological operations	2GHz 2GB RAM	n/a	n	n	BT	-	10	3	n	Manual		TP; other
C	2014	P. Dvorak et al.	Automatic Extraction of Pathological Area in 2D MR Brain Scan	Progress in Electromagnetics Research Symposium		y	T2, FLAIR	Other	symmetry axis detection	n/a	n/a	n	n	BT	-	22	1 (BraTS'12)	y	-		DSC; Acc
C	2014	S. Bauer et al.	Towards automatic MRI volumetry for treatment selection in acute ischemic stroke patients	Annual International Conference of the IEEE Engineering in Medicine and Biology Society		y	T1c, T2, DWI, DSC	Decision Tree, Conditional Random Field	REG:rigid; BE:ref;	n/a	<1 min	n	n	S	S	10	3	n	Manual, 1		DSC; VE
C	2014	A. V. Dalca et al.	Segmentation of Cerebrovascular Pathologies in Stroke Patients with Spatial and Shape Priors	International Conference on Medical Image Computing and Computer-Assisted Intervention		y	FLAIR, T2	Markov Random Field	n/s	n/a	n/a	n	n	WML, S	-	148	3	n/a	Manual		VC
C	2014	F. Rodrigo et al.	Segmentation of Hyperintense Regions Applied to Multiple Sclerosis Lesions	Latin American Congress on Biomedical Engineering	Segmentation failure	n	FLAIR	Thresholding	IN: histogram matching; BE:SPM; REG; enhancement: LP filter + saturation	n/a	n/a	n	n	MS	MS	10	3	n/a	Manual, 1		other

C	2013	Z. Karimaghaloo et al.	Adaptive Voxel, Texture and Temporal Conditional Random Fields for Detection of Gad-Enhancing Multiple Sclerosis Lesions in Brain MRI	International Conference on Medical Image Computing and Computer-Assisted Intervention		y	T1, T1c, T2, PD, FLAIR	Conditional Random Field	BFC; BE; REG; IN:histogram equalization	n/a	n/a	n	n	MS	-	940x2	3	y	Manual, 2	consensus	Sen; PPV; avgFP
C	2013	M. M. Riad et al.	Detection of white matter lesions in cerebral small vessel disease	Medical Imaging 2013: Computer-Aided Diagnosis	Inter-rater agreement evaluated	y	T1, T2, FLAIR	Boosting	REG:FLIRT; BE:BET; BFC:MRF+EM; IN:mean-based	n/a	n/a	n	n	WML	-	100	3 (RUNDMC, van Norden et al. 2011)	n	Manual, 1-3	supervision	ROC; FROC
C	2013	M. Cabezas et al.	A Supervised Approach for Multiple Sclerosis Lesion Segmentation Using Context Features and an Outlier Map	Pattern Recognition and Image Analysis		y	T1, T2, PD, FLAIR	Boosting	BE:BET; denoising:ADF; BFC:N4; REG:affine+non-linear	n/a	n/a	n	n	MS	-	30	3 (Hospital Vall d'Hebron): 3 (Hospital Josep Trueta)	y	Manual, 1		DSC; TPR
C	2013	A. Bianchi et al.	Brain tumor segmentation with symmetric texture and symmetric intensity-based decision forests	Proceedings / IEEE International Symposium on Biomedical Imaging: from nano to macro. IEEE International Symposium on Biomedical Imaging		y	T1, T1c, T2, FLAIR	Decision Tree	IN:mode matching (shifting)	2.4GHz 16GB RAM	1.5 sec	n	n	BT	HGG	20 +50 synthetic	1 (BraTS'12)	y	-		DSC
C	2013	J. Mitra et al.	Classification Forests and Markov Random Field to Segment Chronic Ischemic Infarcts from Multimodal MRI	Multimodal Brain Image Analysis		y	T1, T2, FLAIR, DWI (ADC)	Random Forest, Markov Random Field	resampling; REG; BFC and tissue maps:ref; BE:masking with tissue maps	3.2GHz 23.5GB	13.5 min	n	n	S	-	17	3	n	Manual, 1		DSC; PPV; NPV; Acc
C	2013	M. B. Salah et al.	Fully Automated Brain Tumor Segmentation Using Two MRI Modalities	Advances in Visual Computing	Segmentation failure	y	T1c, FLAIR	Thresholding	BE:in paper; resampling	n/a	n/a	n	n	BT	GBM	19	3 (Cross Cancer Institute, Alberta)	n	Manual, multiple		DSC
C	2013	N. K. Subbanna et al.	Hierarchical Probabilistic Gabor and MRF Segmentation of Brain Tumours in MRI Volumes	Medical Image Computing and Computer-Assisted Intervention – MICCAI 2013		y	T1, T1c, T2, FLAIR	Bayesian classification, Markov Random Field	REG: non-linear	Dell Optiplex 980 i7	~1 hr	n	n	BT	LGG; HGG	45 +65 simulated	1 (BraTS'12)	y	-		DSC
C	2013	I. Diaz et al.	An automatic brain tumor segmentation tool	Annual International Conference of the IEEE Engineering in Medicine and Biology Society		y	T1, T1c, T2, FLAIR	Thresholding, Other	BE:thresholding; IN:histogram matching	1.73 GHz, 6GB RAM	~50 sec	n	n	BT	GBM	16 annotated (+ 60 not)	3 (Cross Cancer Institute, Alberta)	n	Manual, multiple		DSC
C	2013	H. Boussaid et al.	Rapid Mode Estimation for 3D Brain MRI Tumor Segmentation	Energy Minimization Methods in Computer Vision and Pattern Recognition		n/a	n/a	Graph Cut, Other	REG:rigid(medInria)	2.67GHz single core	19 sec	n	n	BT	LGG	113	3	n/a	Manual		DSC
C	2012	V. G. Kanas et al.	Combining Outlier Detection with Random Walker for Automatic Brain Tumor Segmentation	Artificial Intelligence Applications and Innovations		y	T1, T1c, FLAIR	Random Walker	denoising; BFC; REG; BE; IN:histogram matching	n/a	n/a	n	n	BT	meningiona, G, GBM	26	3 (Zacharaki et al. 2009)	n	Expert segmentation		DSC
C	2012	R. S. Ananda and T. Thomas	Automatic segmentation framework for primary tumors from brain MRIs using morphological filtering techniques	International Conference on BioMedical Engineering and Informatics		y	T1-FLAIR, T2	Thresholding, Other	BE:morphological operations	n/a	n/a	n	n	BT	-	50	3 (Sree Chitra Institute of Medical Sciences and Technology (SCIMST)); 3 (Regional Cancer Centre, Thiruvananthapuram, Kerala, Indi)	y	Manual, 1		TI
C	2012	S. Parisot et al.	Joint Tumor Segmentation and Dense Deformable Registration of Brain MR Images	Medical Image Computing and Computer-Assisted Intervention – MICCAI 2012		n	FLAIR	boosting, Markov Random Field	BE; IN:matching median and interquartile range to reference; REG:rigid	n/a	~6 min	n	n	BT	-	93	3	n/a	Manual, 1		DSC; FP; other
C	2012	Z. Karimaghaloo et al.	Hierarchical Conditional Random Fields for Detection of Gad-Enhancing Lesions in Multiple Sclerosis	Medical Image Computing and Computer-Assisted Intervention – MICCAI 2012		n	T1c	Conditional Random Field	BFC; BE; REG; IN	n/a	n/a	n	n	MS	MS	122	3	y	Manual, 2	consensus	Sen; PPV; FP
C	2012	D. Zikic et al.	Decision Forests for Tissue-Specific Segmentation of High-Grade Gliomas in Multi-channel MR	Medical Image Computing and Computer-Assisted Intervention – MICCAI 2012	Fully automatic pre-processing	y	T1, T1c, T2, DTI, FLAIR	Decision Tree, GMM	BE:ref; REG:affine; IN:mean intensity matching	n/a	n/a	n	n	BT	HGG	40	3	n	Manual		DSC
C	2012	D. Zikic et al.	Context-sensitive classification forests for segmentation of brain tumor tissues	Medical Image Computing and Computer-Assisted Intervention – MICCAI 2012		y	T1, T1c, T2, FLAIR	GMM, Decision Tree	BFC: N3; IN: global multiplicative factor	single desktop PC	1-2 min	n	n	BT	LGG, HGG	30 (+25 synthetic)	1 (BraTS'12)	y	-		DSC; Error; Volume Error
C	2012	N. Behzadfar and H. Soltanian-Zadeh	Automatic segmentation of brain tumors in magnetic resonance images	IEEE-EMBS International Conference on Biomedical and Health Informatics		y	T1, T1c, T2, FLAIR	Thresholding, Region Growing, Other,	IN:scaling; BE: LPF + Rodler's method + moprhological operations	n/a	n/a	n	n	BT	GBM	12	3 (Henry Ford Health System, Detroit, MI, USA)	n	Manual, 1		VC; VR
C	2012	I. Mehmood et al.	Automatic Segmentation of Region of Interests in MR Images Using Saliency Information and Active Contours	IT Convergence and Security		n/a	n/a	Other, Active Contour	BE:McStrip	n/a	n/a	n	n	BT	T	n/a (synthetic?); 10	1 (SPL); 3	y	Manual		FROC; DSC
C	2011	S. Bauer et al.	Fully Automatic Segmentation of Brain Tumor Images Using Support Vector Machine Classification in Combination with Hierarchical Conditional Random Field Regularization	Medical Image Computing and Computer-Assisted Intervention – MICCAI 2011		y	T1, T1c, T2, FLAIR	Support Vector Machine, Conditional Random Field	REG:rigid; BE:ref; denoising:edge-preserving smoothing filter; BFC	single CPU 2.33GHz	<120 sec	n	n	BT	-	10	3 (ContraCancrum brain tumor database, Marias et al. 2011)	n	Manual		DSC
C	2010	C. Elliott et al.	Bayesian Classification of Multiple Sclerosis Lesions in Longitudinal MRI Using Subtraction Images	Medical Image Computing and Computer-Assisted Intervention – MICCAI 2010	Scan-rescan reproducibility	y	T1, T2, PD	Bayesian classification	BFC; BE; REG:rigid	n/a	n/a	n	n	MS	-	3x2; 89x(2-4)	3	y	Semiautomatic; Manual		Sen
C	2010	E. Bilotta et al.	A CNN Based Algorithm for the Automated Segmentation of Multiple Sclerosis Lesions	Applications of Evolutionary Computation		n	FLAIR	Artificial neural networks, Genetic Algorithm	tissue maps:SPM8; BE:using WM map	n/a	n/a	n	n	MS	-	11	3	n	Manual, 1		DSC
C	2010	T. Wang et al.	Fully automatic brain tumor segmentation using a normalized Gaussian Bayesian Classifier and 3D Fluid Vector Flow	IEEE International Conference on Image Processing		n/a	n/a	GMM, Other	BE:MIPAV; REG:MIPAV	n/a	~430 sec	n	n	BT	-	10	1 (SPL)	n/a	-		TI
C	2009	J. Liu et al.	Automatic Multiple Sclerosis detection based on integrated square estimation	IEEE Computer Society Conference on Computer Vision and Pattern Recognition Workshops		y	T2, FLAIR	Expectation-Maximization Gausian mixture model, Other	BE:BET; IN:SPM2	1.66GHz 2GB RAM	2 min	n	n	MS	MS	16	3 (University of Kentucky Hospital)	n	Manual, 1		VD; DSC; Sen; Spe
C	2008	E. I. Zacharaki et al.	Measuring Brain Lesion Progression with a Supervised Tissue Classification System	Medical Image Computing and Computer Assisted Intervention – MICCAI 2008	Indirect reproducibility evaluation	y	T1, T2, PD	Support Vector Machine, Boosting	REG:FSL(NMI); BE:BET; BFC:N3; IN:histogram transformation; denoising:Gaussian filter	n/a	n/a	n	n	WML	DM	19 + 23x2	3	n	Manual		other
C	2008	Y. Uchiyama et al.	Automatic segmentation of different-sized leukoaraiosis regions in brain MR images	SPIE Medical Imaging		y	T1, T2	K-Means, Other	BE:region growing	n/a	n/a	n	n	WML	-	73	3 (Gero Hot Springs Hospital, Japan)	n	Manual		ROC

C	2007	X. Xuan and Q. Liao	Statistical Structure Analysis in MRI Brain Tumor Segmentation	International Conference on Image and Graphics		y	T1, T2, FLAIR	boosting	BE; IN	n/a	n/a	n	n	BT	G	10	3	n	Manual		other
C	2007	J. J. Corso et al.	Detection and Segmentation of Pathological Structures by the Extended Graph-Shifts Algorithm	Medical Image Computing and Computer Assisted Intervention – MICCAI 2007		y	T1, T1c, T2, FLAIR	Other	REG;BE;IN:FSL	n/a	~1 min	n	n	BT, MS	GBM; MS	20; 12	3	n/a	Manual		Jcc; PPV; Sen; DR
C	2007	N. Hevia-Montiel et al.	Robust Nonparametric Segmentation of Infarct Lesion from Diffusion-Weighted MR Images	Annual International Conference of the IEEE Engineering in Medicine and Biology Society		n	DWI	Other	ADC thresholding	n/a	n/a	n	n	S	S	15	3 (La Salpêtrière hospital, Paris, France)	n/a	Manual, 1		VC; TI
C	2006	Z. Lao et al.	Automated segmentation of white matter lesions in 3D brain MR images, using multivariate pattern classification	3rd IEEE International Symposium on Biomedical Imaging: Nano to Macro, 2006.	Inter-rater agreement evaluated	y	n/a	Support Vector Machine, Boosting	REG:ITK(MI); BE:BET; BFC:histogram matching; smoothing:Gaussian kernel	n/a	n/a	n	n	WML	-	45	3 (ACCORD-MIND)	n/a	Manual, 2		VC; other
C	2006	R. Harmouche et al.	Bayesian MS Lesion Classification Modeling Regional and Local Spatial Information	18th International Conference on Pattern Recognition (ICPR'06)	Inter-rater agreement evaluated	y	T1, T2, PD	Bayesian classification, Markov Random Field	BFC:N3; REG:ref; BE:ref; IN:ref	n/a	n/a	n	n	MS	MS	10	3	n/a	Manual, 5	majority	DSC
C	2005	K. M. Iftekharuddin et al.	Automatic brain tumor detection in MRI: methodology and statistical validation	SPIE Medical Imaging		n	T1	Self Organising Maps, Artificial neural networks	n/a	n/a	n/a	n	n	BT	-	50	3	n	Manual		ROC; DSC; Jcc; other
C	2005	M. Schmidt et al.	Segmenting brain tumors using alignment-based features	International Conference on Machine Learning and Applications		n/a	n/a	Support Vector Machine	denoising:non-linear filtering; BFC:N3; IN:weighted regression; REG:SPM	n/a	n/a	n	n	BT	-	10	3	y	Manual, 1		Jcc
C	2004	W. Li et al.	Automatic segmentation of cerebral ischemic lesions from diffusion tensor MR images	Medical Imaging 2004: Image Processing	Inter-rater agreement evaluated	n	DTI	Other	denoising:non-linear filtering	n/a	n/a	n	n	S	S	20	3	y	Manual		SI
C	2003	A. Hadjiprocopis and P. Tofts	An automatic lesion segmentation method for fast spin echo magnetic resonance images using an ensemble of neural networks	IEEE XIII Workshop on Neural Networks for Signal Processing		y	T1, T2, PD	Artificial neural networks	n/a	n/a	n/a	n	n	MS	-	20	3	y	Manual		Sen; Spe
C	2003	W. Li and J. Tian	Automatic segmentation of brain infarction in diffusion-weighted MR images	Medical Imaging 2003: Image Processing	Inter-rater agreement evaluated	n	DWI	Other	denoising:ADF; REG:global-rigid + local-non-rigid	n/a	n/a	n	n	S	-	20	3	y	Manual		DSC
C	2013	J. Huang et al.	Brain Tumor Segmentation Based on Texture, Intensity, and Edge	World Congress on Medical Physics and Biomedical Engineering		y	T1c, T2	Other	n/a	n/a	n/a	n	n	BT	-	19	3	n/a	Manual, multiple		TP; FP; FN; other
J	2012	Z. Karimaghaloo et al.	Automatic Detection of Gadolinium-Enhancing Multiple Sclerosis Lesions in Brain MRI Using Conditional Random Fields	IEEE Transactions on Medical Imaging		y	T1, T1c, T2, PD, FLAIR	Conditional Random Field, Graph Cut	REG:ref; BFC:N3; IN:histogram matching; BE:ref	2.67GHz quad-core P4 desktop	~5 sec	n	n	MS	MS	80	3	n/a	Manual, 2	consensus	Sen; FP; PPV
J	2017	Y. Liu et al.	A deep convolutional neural network-based automatic delineation strategy for multiple brain metastases stereotactic radiosurgery	PloS one		intended monomodality	T1c, FLAIR	Artificial neural networks	BE:ROBEX	NVIDIA Quadro M2000M Intel Xeon E3-1505	<2 min	n	n	MET	-	265; 240	1 (BraTS);<br>3 (University of Texas Southwestern Medical Center, Wardak et al. 2016)	y	Maunal, 1		DSC; avgnSSD; sdSSD; Sen; Spe; ROC
J	2017	S. Mitra et al.	Volumetric brain tumour detection from MRI using visual saliency	PloS one		y	T1c, T2, FLAIR	Other	IN; 8bit range	n/a	n/a	n	n	BT	LGG; HGG	274	1 (BraTS'15)	y	-		ROC
C	2016	M. Agn et al.	A generative model for segmentation of tumor and organs-at-risk for radiation therapy planning of glioblastoma patients	SPIE Medical ImagingMedical Imaging 2016: Image Processing	Organs at Risk segmentation	y	T1c, T2, FLAIR	Artificial neural networks	REG	Core i5-2400 3.1GHz CPU	2 hrs	n	n	BT	LGG, HGG; GBM	30; 20	1 (BraTS'13);<br>3 (Rigshospitalet, Copenhagen University Hospital)	y	Manual		DSC; TPR; PPV
J	2019	K. R. Laukamp et al.	Fully automated detection and segmentation of meningiomas using deep learning on routine multiparametric MRI	European Radiology		y	T1, T1c, T2, FLAIR	Artificial neural networks	BFC; REG; BE; resampling; IN:0u1v	n/a	n/a	n	n	BT	meningioma	136	3 (University Hospital of Cologne)	y	Semiautomatic		DSC
J	2015	A. Karimian and S. Jafari	A New Method to Segment the Multiple Sclerosis Lesions on Brain Magnetic Resonance Images	J Med Signals Sens		y	T1, T2, FLAIR	GMM	BFC:LPF; BE:morphological operations	n/a	n/a	n	n	MS	-	25	3	y	Manual		DSC; Acc; Sen; Spe
J	2014	R. Wang et al.	Automatic Segmentation and Quantitative Analysis of White Matter Hyperintensities on FLAIR Images Using Trimmed-Likelihood Estimator	Academic Radiology		n	FLAIR	Other	BFC:N3; BE:BET	n/a	n/a	n	y	WML	-	82	3	n	Manual, 1		DSC; FPR; FNR; VR; VD
J	2018	X. Zhao et al.	A deep learning model integrating FCNNs and CRFs for brain tumor segmentation	Medical Image Analysis		y	T1, T1c, T2, FLAIR	Artificial neural networks, Conditional Random Field	IN:in paper; BFC:N4	Tesla K80 GPU, Inter E5-2620 CPU	2-4 min	n	n	BT	LGG, HGG	65+31; 274+110; 191	1 (BraTS'13);<br>1 (BraTS'15);<br>1 (BraTS'16)	y	-		DSC; PPV; Sen
C	2013	L. Zhao et al.	Automatic brain tumor segmentation with MRF on supervoxels	Multimodal Brain Tumor Segmentation		y	T1, T1c, T2, FLAIR	Supervoxel, Markov Random Field, Graph Cut	Denoising: SUSAN; IN: z-score	n/a	n/a	n	n	BT	LGG; HGG	65+31	1 (BraTS'13)	y	-		DSC; Spe; Sen
J	2018	R. Zhng et al.	Automatic segmentation of acute ischemic stroke from DWI using 3D Fully Convolutional DenseNets	IEEE Trans Med Imaging		y	DTI, ADC	Artificial neural networks	IN:0u1v, zero-padding, cropping, resampling	I7-4790 3.6GHz, 16GB RAM CPU, Nvidia Titan X GPU	~0.1 sec	n	n	S	S	242; 64	1 (ISLES'15);<br>3	y	Manual, 2	supervision	DSC; Sen; F1; PPV; Sen; VD
J	2017	T. Zhan et al.	Multimodal spatial-based segmentation framework for white matter lesions in multi-sequence magnetic resonance images	Biomedical Signal Processing and Control		y	T1, T2, FLAIR	Logistic Regression, Markov Random Field, Bayesian classification	BE: BET; BFC: N4; IN:linear contrast adjustment	n/a	n/a	n	n	WML	-	50; 54	1 (MS GC MICCAI'08);<br>3 (ACCORD-MIND)	y	Manual, 1		DSC; TPR; TNR; VD; AD; ROC
C	2012	E. Zacharaki et al.	Fuzzy Multichannel clustering with individualized spatial priors for segmenting brain lesions and infarcts	Artificial Intelligence Applications and Innovations		y	T1, FLAIR	K-Means, Fuzzy C-Means	BE; BFC; IN; REG:HAMMER	n/a	n/a	n	n	S, NEC	DM	47 (+72 healthy)	3	y	Manual, 1&2		DSC
C	2016	Y. Yoo et al.	Deep learning of brain lesion patterns for predicting future disease activity in patients with early symptoms of multiple sclerosis	Deep Learning and Data Labeling for Medical Applications		y	T2, PD	Artificial neural networks, Euclidean Distance Transform	BE; IN: linear; REF: affine	n/a	n/a	n	n	MS	early MS	140	3	n/a	Semiautomatic		Acc; Sen; Spe; ROC
preprint	2016	D. Yi, et al.	3-D convolutional neural networks for glioblastoma segmentation	arXiv preprint		y	T1, T1c, T2, FLAIR	Artificial neural networks	n/a	n/a	n/a	n	n	BT	LGG; HGG	274 + n	1 (BraTS'13);<br>1 (BraTS'15)	y	-		DSC
J	2014	W. Wu et al.	Brain tumor detection and segmentation in a conditional random fields framework with pixel-pairwise affinity and superpixel-level features	International Journal of Computer Assisted Radiology and Surgery		y	T1, T1c, T2, FLAIR	Superpixel, Gabor wavelets, SVM, Conditional Random Field	n/a	single desktop PC	30 min	n	n	BT	GBM	20; 30 (+25 synthetic)	1 (BraTS'12);<br>3 (Corso et al. 2008)	y	n/a		Acc; PPV; Sen; Jcc; DSC
J	2007	C. Vijayakumar et al.	Segmentation and grading of brain tumors on apparent diffusion coefficient images using self-organizing maps	Computerized Medical Imaging and Graphics		y	ADC, T2, FLAIR	Self Organising Maps	REG; IN; BE	n/a	n/a	n	n	BT	LGG; HGG	10	3	n	Manual, 2		ROC; Sen; Spe
J	2008	R. Verma et al.	Multi-parametric tissue characterization of brain neoplasms and their recurrence using pattern classification of MR images	Academic Radiology		y	B0, DWI, FLAIR, T1, T1c, FA, ADC	Bayesian classification, SVM	BE:FSL; REG:FLIRT; IN: histogram matching	n/a	n/a	n	n	BT	HGG	14	3	y	Manual, 1		Sen; Spe; other
C	2015	S. Vaidya et al	Longitudinal multiple sclerosis lesion segmentation using 3D convolutional neural networks	2015 Longitudinal Multiple Sclerosis Lesion Segmentation Challenge		y	T1, T2, PD, FLAIR	Artificial neural networks	n/a	Nvidia Tesla K20 GPU	2 min	n	n	MS	MS	5xn; 14xn	1 (MSC'15)	n	-		DSC; Jcc; PPV; Sen; TPR; FPR; VD; SD; VC
J	2017	K. Usman et al	Brain tumor classification from multi-modality MRI using wavelets and machine learning	Pattern Analysis and Applications		y	T1, T1c, T2, FLAIR	Decision Tree	IN: histogram matching; bounding box of tumor	I5, 2.5GHz, 8 GB RAM	3 min	n	n	BT	LGG, HGG	30	1 (BraTS'13)	y	-		DSC; Jcc; Spe
C	2014	G. Urban et al.	Multi-modal brain tumor segmentation using deep convolutional neural networks	MICCAI BraTS (brain tumor segmentation) challenge		y	T1, T1c, T2, FLAIR	Artificial neural networks	IN: mean CSF normalization	n/a	n/a	n	n	BT	LGG, HGG	30	1 (BraTS'13)	y	-		DSC
J	2015	N. J. Tustison et al.	Optimal symmetric multimodal templates and concatenated random forests for supervised brain tumor segmentation (simplified) with ANTsR	Neuroinform		y	T1, T1c, T2, FLAIR	Decision Tree, MAP, Markov Random Field	IN: 0-1range	Single threaded cluster	2 hrs	n	y	BT	LGG, HGG	55	1 (BraTS'13)	Y	-		DSC; PPV; Sen
C	2013	T. Taylor et al.	Map-reduce enabled hidden Markov models for high throughput multimodal brain tumor segmentation	Multimodal Brain Tumor Segmentation		y	T1, T1c, T2, FLAIR	Hidden Markov Models	movement artefact removal; BFC; IN	n/a	~500 sec	n	n	BT	HGG	20 (+25 synthetic)	1 (BraTS'13)	y	-		DSC
C	2009	N. Subbanna et al.	MS lesion segmentation using Markov Random Fields	MICCAI 2009		y	T1, T2, PD	Markov Random Field	BFC: N3; REG:ref; BE:ref; IN	n/a	n/a	n	n	MS	MS	24	3	n	Manual, 5	consensus	DSC; FPR; FNR
C	2015	N. Subbanna et al.	IMaGe: Iterative multilevel probabilistic graphical model for detection and segmentation of multiple sclerosis lesions in brain MRI	Information Processing in Medical Imaging		y	T1, T2, PD, FLAIR	Markov Random Field	BFC: N3; REG:ref; BE:FSL; IN:histogram matching	Dell Optiplex 980 i7	~40 min	n	n	MS	MS	1195	3	y	Semiautomatic		PPV; Sen; DSC
C	2008	J. C. Souplet	An automatic segmentation of T2-FLAIR multiple sclerosis lesions	MICCAI 2008		y	T1, T2, FLAIR	Expectation-Maximization, outlier detection	cropping; BE:ref; BFC:ref	n/a	96 min	n	y	MS	MS	25	1 (MS GC MICCAI'08)	y	-		VD; AD; TP; FP
C	2016	R. Saha et al.	Brain tumor segmentation from multimodal mr images using rough sets	Computer Vision, Graphics, and Image Processing		y	T1c, T2, FLAIR	Rough set, k-means, Quadtree partitioning	n/a	n/a	n/a	n	n	BT	LGG, HGG	304	1 (BraTS'13);<br>1 (BraTS'15)	y	-		PPV; Sen; DSC

C	2007	J. Rexilius et al.	Multispectral brain tumor segmentation based on histogram model adaptation	SPIE Medical Imaging	Semiautomatic preprocessing (brain extraction)	y	T2, FLAIR	tumor model, region growing	movement artefact removal; REG: affine; BE: watershed	2GHz AMD Athlon	10 min	n	n	BT	-	12	3	y	Manual		Jcc
C	2012	K. K. Reddy	Confidence guided enhancing brain tumor segmentation in multi-parametric MRI	ISBI		y	T1, T1c, T2, FLAIR	SVM, AdaBoost, Level Set, Region Growing	n/a	n/a	n/a	n	n	BT	-	11	3	n	Manual, 1		TPR; FPR; DSC
C	2011	P. Raniga et al.	Local intensity model: An outlier detection framework with applications to white matter hyperintensity segmentation	ISBI		y	T2, FLAIR	local intensity model, outlier detection	BFC:ref; REG:rigid; IN: CSF and WM normalization	n/a	n/a	n	n	WML	MCI, AD	14 (+44 healthy elderly)	3 (AIBL, Ellis et al., 2009)	n	Manual, 1		DSC; Overlap fraction; Extra fraction; missed fraction
C	2016	R. P. Randhawa	Improving segment boundary classification for brain tumor segmentation and longitudinal disease progression	Brainlesion: Glioma, Multiple Sclerosis, Stroke and Traumatic Brain Injuries		y	T1, T1c, T2, FLAIR	Artificial neural networks	BFC:N4; IN:0u1v	n/a	6 min	n	n	BT	LGG, HGG	274	1 (BraTS'15)	y	-		DSC
C	2015	O. Puonti	Simultaneous whole-brain segmentation and white matter lesion detection using contrast-adaptive probabilistic models	Brainlesion: Glioma, Multiple Sclerosis, Stroke and Traumatic Brain Injuries	Minimal preprocessing	y	T1, T2, FLAIR	Restricted Boltzmann machine, GMM	none	Quad-core Xeon 5472 3GHz 32 RM, GTX Titan 6 GB GPU	2 hrs	n	n	WML	MS	20	1 (MS GC MICCAI'08)	y	-		PPV; TPR
J	2006	K. B. Prakash	Identification, segmentation, and image property study of acute infarcts in diffusion-weighted images by using a probabilistic neural network and adaptive gaussian mixture model	Academic Radiology		n	DWI	Artificial neural networks, GMM	n/a	n/a	1 min	n	n	S	-	13	3	n/a	Semiautomatic		DSC; Sen; Spe
C	2015	V. Pedoia	Fully automatic brain tumor segmentation by using competitive EM and Graph Cut	Image Analysis and Processing – ICIAP		n/a	FLAIR	Competitive Expectation-Maximization, Graph cut	n/a	n/a	n/a	n	n	BT	LGG, HGG	20; 30 (+50 synthetic)	1 (BraTS'12); 3	y	Manual, 6		Jcc; DSC
C	2012	S. Parisot et al.	Graph-based Detection, Segmentation and Characterisation of Brain Tumours	IEEE Conference on Computer Vision and Pattern Recognition		n	FLAIR	Sparse graph	IN: mean and IQ based	n/a	n/a	n	n	BT	LGG	113	3	n/a	Manual		DSC
C	2016	B. Pandian et al.	Multimodal tumor segmentation with 3D convolutional neural networks	MICCAI BRATS		y	T1, T1c, T2, FLAIR	Artificial neural networks	BFC:N4; IN: 0u1v	4 core i7 32GB RAM, NVIDIA GTX 1080 GPU	n/a	n	n	BT	-	40	1 (BraTS'16)	y	-		DSC
C	2008	J. Morra et al.	Automatic segmentation of MS lesions using a contextual model for the MICCAI grand challenge	MICCAI 2008		y	T1, T2, FLAIR, DTI	Probabilistic boosting tree	downsampling, BFC: ref	n/a	n/a	n	n	MS	MS	25	1 (MS GC MICCAI'08)	y	-		VD; AD; Sen; Spe; PPV
C	2015	R. Meier, et al.	Parameter Learning for CRF-based Tissue Segmentation of Brain Tumors	Brainlesion: Glioma, Multiple Sclerosis, Stroke and Traumatic Brain Injuries		y	T1, T1c, T2, FLAIR	Conditional Random Field, Decision trees	n/a	n/a	n/a	n	n	BT	LGG, HGG	24x2; 20; 53	1 (BraTS'13); 1 (BraTS'15)	y	-		DSC; VE
C	2016	R. Meier, et al.	CRF-based brain tumor segmentation: alleviating the shrinking bias	Brainlesion: Glioma, Multiple Sclerosis, Stroke and Traumatic Brain Injuries		y	T1, T1c, T2, FLAIR	Dense Conditional Random Field, Decision trees	n/a	n/a	n/a	n	n	BT	LGG; HGG	51; 357; 25	1 (BraTS'13); 1 (BraTS'15)	y	-		DSC; VE
C	2016	O. Maier at al.	Image features for brain lesion segmentation using random forests	Brainlesion: Glioma, Multiple Sclerosis, Stroke and Traumatic Brain Injuries		y	T1, T1c, T2, FLAIR, DWI, CBF, CBV, TTP, Tmax	Random Forest	BFC:CMTK; IN:intensity harmonization	n/a	n/a	n	n	BT, S	S, LGG, HGG	104; 274	1 (BraTS'15); 1 (ISLES'15)	y	-		DSC; avgSSD; Hdff
C	2016	T .K. Lun et al.	Brain tumor segmentation using deep convolutional neural network	MICCAI BRATS		y	T1, T1c, T2, FLAIR	Artificial neural networks	n/a	n/a	n/a	n	n	BT	-	274	1 (BraTS'15)	y	-		DSC; VE
C	2016	L. Lefkovits et al.	Brain Tumor Segmentation with Optimized Random Forest	Brainlesion: Glioma, Multiple Sclerosis, Stroke and Traumatic Brain Injuries		y	T1, T1c, T2, FLAIR	Random Forest	denoising: ADF; BFC:N4; IN;histogram matching	n/a	n/a	n	n	BT	LGG; HGG	274	1 (BraTS'15)	y	-		DSC
J	2008	Z. Lao et al.	Computer-assisted segmentation of white matter lesions in 3D MR images using support vector machine	Academic Radiology	Inter-rater agreement evaluated	y	T1, T2, PD, FLAIR	SVM	REG:affine; BE:BET; BFC:N3; IN:histogram matching	n/a	n/a	n	n	WML	DM	45	3 (ACCORD-MIND)	y	Manual, 1		ROC; VC
C	2016	S. Kumar et al.	A picture fuzzy clustering approach for brain tumor segmentation	International Conference on Cognitive Computing and Information Processing (CCIP)		n/a	n/a	Fuzzy set	n/a	n/a	n/a	n	n	BT	LGG; HGG	65 (+65 synthetic)	1 (BraTS)	y	-		Jcc; DSC
C	2008	D. Kroon	Multiple sclerosis detection in multispectral magnetic resonance images with principal components analysis	Grand Challenge Work.: Mult. Scler. Lesion Segm. Challenge		y	T1, T2, FLAIR, MD, FA	PCA, log.likelihood ratio	BFC: various	n/a	n/a	n	n	MS	MS	44	1 (MS GC MICCAI'08)	y	-		DSC; VD; AD; Sen; Spe; PPV
C	2016	J. Knight et al.	MS lesion segmentation using FLAIR MRI only	MICCAI-MSSEG		n	FLAIR	edge detection, Fuzzy classification	BFC:SPM12; denoising:Gaussian LPF	n/a	n/a	n	n	MS	MS	15	1 (MS Lesion Segmentation Challenge '16)	y	-		DSC; PPV; TPR; Bland-Altman; ANCOVA
C	2007	H. Khotanlou et al.	Automatic brain tumor segmentation using symmetry analysis and deformable models	Advances in Pattern Recognition		n	T1	symmetry plane, histogram subtraction, deformable model	n/a	n/a	n/a	n	n	BT	-	10	3	n	Manual		Hdff; SD; other
C	2015	A. Jesson et al.	Hierarchical MRF and random forest segmentation of MS lesions and healthy tissue in brain MRI	Proceedings of the 2015 Longitudinal Multiple Sclerosis Lesion Segmentation Challenge		y	T1, T2, FLAIR	Random Forest, pathological atlases	IN:sigmoid; denoising:non-local means	n/a	n/a	n	n	MS	-	5x21	1 (MSC'15)	y	-		DSC; Jcc; TPR; FPR
C	2015	T. Jerman et al.	Combining unsupervised and supervised methods for lesion segmentation	Brainlesion: Glioma, Multiple Sclerosis, Stroke and Traumatic Brain Injuries		y	T1, T2, FLAIR	GMM, Decision trees	BE:reg; REG:affine; BFC:N4; IN:histogram matching; resampling:iso 1mm3 vox	n/a	n/a	n	n	MS	-	18	3 (University Medical Centre Ljubljana)	n	Manual, 2	consensus	DSC; TPR; PPV
J	2014	V. Ithapu et al.	Extracting and summarizing white matter hyperintensities using supervised segmentation methods in Alzheimer's disease risk and aging studies	Human Brain Mapping		n	FLAIR	SVM, Random Forest	REG; tissue seg:SPM12	n/a	n/a	n	y	WML	MCI, AD	38	3 (Wisconsin Alzheimer's Disease Research Center)	n	Semiautomatic		ROC; F score; other
J	2013	A. Islam et al.	Multifractal Texture Estimation for Detection and Segmentation of Brain Tumors	IEEE Transactions on Biomedical Engineering		y	T1, T2, FLAIR	boosting	REG:SPM8; BFC:SPM8; IN:histogram matching; BE:BET	n/a	n/a	n	n	BT	Astrocytoma, medulloblastoma	14 ;14	1 (BraTS'12); 3	y	Manual		ROC; DSC; Jcc; TI; other
J	2018	S. Iqbal et al.	Brain tumor segmentation in multi-spectral MRI using convolutional neural networks	Microscopy Research & Technique		y	T1, T1c, T2, FLAIR	Artificial neural networks	IN:0-255 range & mean subtraction; BFC:N4; cropping	Titan X 12 GB RAM GPU	<1 sec	n	n	BT	LGG, HGG	274	1 (BraTS'15)	y	-		DSC; Sen; Spe
J	2016	E. Ilunga-Mbuyamba et al.	Active contours driven by Cuckoo Search strategy for brain tumour images segmentation	Expert Systems with Applications		n	T1	Active Contour, Cuckoo Search Strategy	n/a	1.9GHz 4GB RAM	~180 sec	n	n	BT	GBM, MET	10	3 (University Hospital, Department of Neurosurgery, University of Leipzig, Germany)	n	Manual, 1		DSC; Jcc; Hdff
J	2012	K. M. Hulsey et al.	Automated quantification of white matter disease extent at 3T: Comparison with volumetric readings	Journal of Magnetic Resonance Imaging		y	MP-RAGE, FLAIR	thresholding	BE:FSL; tissue seg:Sienax; REG:affine	n/a	n/a	n	n	WML	-	28	3 (Dallas Heart Study, Ronald et al 2004)	n	Manual, 2		VC; VR; Bland-Altman
C	2015	A. Hoogi et al.	Multimodal Brain Tumor Segmentation (BRATS) using Sparse Coding and 2-layer Neural Network	MICCAI BRATS		y	T1, T1c, T2, FLAIR	Artificial neural networks, Sparse coding	IN: mean subtraction	n/a	n/a	n	n	BT	LGG, HGG	100	1 (BraTS'15)	y	-		DSC
J	2011	H. Khotanlou et al.	Segmentation of multiple sclerosis lesions in brain MR images using spatially constrained possibilistic fuzzy c-means classification	J Med Signals Sens		y	T1, T2	probabilistic fuzzy c-means	n/a	n/a	n/a	n	n	MS	-	10	3 (Imam Khomeini hospital)	n	Manual		DSC; Overlap fraction; Extra fraction
C	2015	T. Haeck et al.	Automated model-based segmentation of brain tumors in MR images	MICCAI BRATS		y	T1, T1c, T2, FLAIR	Expectation-Maximization, level set	BFC; Brain mask erosion	2 x 2.66 GHz Quad Core CPU	15 min	n	n	BT	LGG, HGG	274	1 (BraTS'15)	y	-		DSC
C	2015	T. Haeck et al.	Automated model-based segmentation of ischemic stroke in MR images	Brainlesion: Glioma, Multiple Sclerosis, Stroke and Traumatic Brain Injuries		y	T1, TTP, FLAIR	Expectation-Maximization, level set	none	2 x 2.66 GHz Quad Core CPU	15 min	n	n	S	-	64; 50	1 (ISLES'15)	y	-		DSC; avgSSD; Hdff; PPV; Sen
J	2018	N. Gupta et al.	Identification of gliomas from brain mri through adaptive segmentation and run length of centralized patterns	Journal of Computational Science		y	T1, T1c, T2, FLAIR	Image fusion, Thresholding	denoising: ADF	n/a	n/a	n	n	BT	LGG; HGG	80, 134	1 (BraTS'13); 3 (NSCB Medical College Jabalpur, India)	y	n/a		Jcc; DSC; Hdff; other
C	2014	M. Goetz et al.	Extremely randomized trees based brain tumor segmentation	MICCAI BRATS		y	T1, T1c, T2, FLAIR	Decision Tree	BFC:N4; IN:histogram matching + mode subtraction and 1v	n/a	n/a	n	n	BT	LGG; HGG	30	1 (BraTS'13)	y	-		DSC
J	2016	M. Ghafoorian et al.	Location sensitive deep convolutional neural networks for segmentation of white matter hyperintensities	Scientific Reports	Inter-rater agreement evaluated	y	T1, FLAIR	Artificial neural networks	REG:FLIRT; BE:BET; BFC:FAST; IN:0-1 range	n/a	n/a	n	n	WML	SVD	420	3 (RUNDMC, van Norden et al. 2011)	n	Manual		DSC; ROC
J	2017	M. Ghafoorian et al.	Deep multiscale location-aware 3d convolutional neural networks for automated detection of lacunes of presumed vascular origin	NeuroImage: Clinical	Inter-rater agreement evaluated	y	T1, FLAIR	Artificial neural networks	REG:FLIRT; BE:BET; BFC:FAST; IN:0-1 range	n/a	n/a	n	n	S	SVD; S	654; 421	3 (RUNDMC, van Norden et al. 2011); 3 (FUTURE, Rutten-Jacobs et al. 2011)	y	Manual, 1		FROC

C	2016	L. Le Folgoc et al.	Segmentation of brain tumors via cascades of lifted decision forests	MICCAI BRATS			y	T1, T1c, T2, FLAIR	Decision Tree, mixture model, Markov Random Field	n/a	Intel Xeon 3.6GHz 16GB RAM	~30 sec	n	n	BT	LGG; HGG	20; 200	1 (BraTS'13); 1 (BraTS'15)	y	-		DSC
C	2016	L. Le Folgoc et al.	Lifted auto-context forests for brain tumour segmentation	Brainlesion: Glioma, Multiple Sclerosis, Stroke and Traumatic Brain Injuries	semiautomatic preprocessing *brats 2016 contains some unpreprocessed or partially preprocessed images		y	T1, T1c, T2, FLAIR	Decision Tree, mixture model, Markov Random Field	IN:median and deviation; reg:rigid; BFC:ref; BE	Intel Xeon 3.6GHz 16GB RAM	~20 sec	n	n	BT	LGG; HGG	30;70; 274(?)	1 (BraTS'13); 1 (BraTS'15); 1 (BraTS'16)	y	-		DSC
J	2013	J. B. Fiot et l.	Efficient brain lesion segmentation using multi-modality tissue-based feature selection and support vector machines	International journal for numerical methods in biomedical engineering			y	T1, T2, PD, FLAIR	Support Vector Machine	REG:rigid; BE:ref; BFC:ref; denoising:ADF; tissue seg:EM	n/a	n/a	n	n	WML	-	125	3 (AIBL, Ellis et al., 2009)	n/a	Manual, 2	supervision	DSC; Sen; Spe
C	2013	J. Festa et al.	Automatic brain tumor segmentation of multi-sequence MR images using random decision forests	MICCAI BRATS			y	T1, T1c, T2, FLAIR	Random Forest	BFC:N4; IN:histogram matching	I7 3.2GHz 24 GB RAM	20-25 min	n	n	BT	LGG; HGG	30 +50 simulated	1 (BraTS'13)	y	-		DSC; Jcc; Sen; Spe; Kappa
C	2003	R. J. Ferrari et al.	Segmentation of multiple sclerosis lesions using support vector machines	SPIE Medical Imaging			y	T1, T2, FLAIR	Support Vector Machine	denoising: ADF; BFC:N3; IN:CSF linear normalization	2GHz 1GB RAM	~40 min	n	n	MS	-	18	3	n	Semiautomatic		Acc; FNR
C	2015	C. Feng et al.	Segmentation of ischemic stroke lesions in multi-spectral MR images using weighting suppressed FCM and three phase level set	Brainlesion: Glioma, Multiple Sclerosis, Stroke and Traumatic Brain Injuries			y	T1, T1c, T2, FLAIR, DWI, CBF, CBV, TTP, Tmax	Fuzzy C-Means, Image fusion, Level set	n/a	n/a	n/a	n	n	S	-	64; 50	1 (ISLES'15)	y	-		DSC; avgSSD; Hdff
C	2016	A. Ellawaa et al.	Brain tumor segmentation using random forest trained on iteratively selected patients	Brainlesion: Glioma, Multiple Sclerosis, Stroke and Traumatic Brain Injuries			y	T1, T1c, T2, FLAIR	Random Forest	BFC:N4; IN:histogram matching	n/a	n/a	n	n	BT	LGG; HGG	70?	1 (BraTS'16)	y	-		DSC; Sen; Spe
C	2015	P. Dvorak et al.	Local Structure Prediction with Convolutional Neural Networks for Multimodal BrainTumor Segmentation	Medical Computer Vision: Algorithms for Big Data			y	T1, T1c, T2, FLAIR	Artificial neural networks	n/a	4 core Intel Xeon E3 3.3GHz	13 sec	n	n	BT	LGG; HGG	309	1 (BraTS'14)	y	-		DSC
C	2013	S. Doyle et al.	Fully automatic brain tumor segmentation from multiple MR sequences using hidden Markov fields and variational EM	MICCAI BRATS			y	n/a	Markov Random Field, Expectation-Maximization	n/a	n/a	n/a	n	n	BT	LGG; HGG	30?	1 (BraTS'13)	y	-		DSC
J	2007	S. Datta et al.	Segmentation of gadolinium-enhanced lesions on MRI in multiple sclerosis	Journal of Magnetic Resonance Imaging	Semiautomatic preprocessing (brain extraction)		y	T1, T1c, FSE, FLAIR	Fuzzy connectedness, Other	REG:ref; BFC:SPM2; BE:ref; denoising:ADF	2.99GHz 1BG RAM	<10 min	n	n	MS	RRMS	22	3	n	Manual, 1		DSC; Bland-Altman; other
J	2012	S. Damangir et al.	Multispectral MRI segmentation of age related white matter changes using a cascade of support vector machines	Journal of Neurological Sciences			y	T1, FLAIR	Support Vector Machine	REG:FLIRT; BE:BET; BFC:N3; IN:linear transformation + 0-1 range	n/a	3 min	n	n	WML	AD, LBD	70 + 32 healthy	3 (he Dementia Study in Western Norway)	y	Manual		ROC; Sen; Spe; Acc; VC
C	2007	H. Cai et al.	Probabilistic segmentation of brain tumours based on multimodality magnetic resonance images	ISBI			y	T1, T1c, B0, DWI, FLAIR, DTI (FA, ADC)	Quadratic Discriminant Analysis, Support Vector Machine	BE:FSL; denoising:FSL; REG:FLIRT; IN:histogram matching	n/a	n/a	n	n	BT	-	22	3 (Hospital, University of Pennsylvania)	y	Expert segmentation		Acc
C	2012	C. P. Yu et al.	Statistical asymmetry-based brain tumor segmentation from 3D MR images	Biosignals	Segmentation failure		y	T1, T1c	Symmetry plane, Watershed	n/a	n/a	3 min	n	n	BT	-	17	3	n/a	Manual		Jcc
C	2016	P. Chang	Fully convolutional neural networks with hyperlocal features for brain tumor segmentation	MICCAI BRATS			y	T1, T1c, T2, FLAIR	Artificial neural networks	IN:histogram matching + 0-1 range	NVIDIA GTX TITAN X 12GB RAM	<1 sec	n	n	BT	HGG	144	1 (BraTS'16)	y	-		DSC; Hdff
J	2017	I. Cabria et al.	MRI segmentation fusion for brain tumor detection	Information Fusion			n	FLAIR	Potential Field Segmentation	n/a	n/a	n/a	n	n	BT	-	22	1 (BraTS'13)	n/a	-		other
C	2013	P. Buendia et al.	A grouping artificial immune network for segmentation of tumor images	MICCAI BRATS			y	T1, T1c, T2, FLAIR	Grouping Artificial Immune Network	denoising; BFC; IN:histogram matching	n/a	21 sec	n	n	BT	HGG	20	1 (BraTS'12)	y	-		DSC; Jcc; Sen; Spe; Kappa
J	2000	A. Boudra et al.	Automated segmentation of multiple sclerosis lesions in multispectral MR imaging using fuzzy clustering	Computers in Biology and Medicine			y	PD, T2	Fuzzy C-Means	enhancement:histogram equalization, BE:ref	n/a	n/a	n	n	MS	-	10	3	n	Manual, 2		other
C	2016	A. Birenbaum et al.	Longitudinal multiple sclerosis lesion segmentation using multi-view convolutional neural networks	Deep Learning and Data Labeling for Medical Applications			y	T1, T2, PD, FLAIR	Artificial neural networks	REG:rigid; BFC; IN:histogram matching	n/a	n/a	n	n	MS	-	19x4-6	1 (MSC'15)		-		DSC
C	2011	A. Bijar et al.	Segmentation of MS lesions using active contour model, adaptive mixtures method and mrf model	International Symposium on Image and Signal Processing and Analysis (ISPA)			y	T1, T1c, T2, FLAIR	Adaptive mixture model, Markov Random Field, Bayesian Classification	n/a	n/a	n/a	n	n	MS	-	20	3 (Khayati et al. 2008)	n/a	Manual, 2		DSC; Overlap fraction; Extra fraction
J	2011	A. Bijar et al.	Segmentation of MS lesions using entropy-based EM algorithm and Markov random fields	Journal of Biomedical Science and Engineering			y	n/a	GMM, Markov Random Field, Bayesian Classification, Expectation-Maximization	n/a	n/a	n/a	n	n	MS	-	20	3 (Khayati et al. 2008)	n/a	Manual, 2		DSC; Overlap fraction; Extra fraction
J	2008	K. B. Prakash	Automatic processing of diffusion-weighted ischemic stroke images based on divergence measures: Slice and hemisphere identification, and stroke region segmentation	International Journal of Computer Assisted Radiology and Surgery			n	DWI	intensity pdf divergence of 2 hemispheres	n/a	n/a	n/a	n	n	S	-	57	3	y	Manual, 1		ROC; DSC; Sen; Spe
preprint	2017	A. Beers et al.	Sequential 3d u-nets for biologically-informed brain tumor segmentation	arXiv preprint arXiv:1709.02967			y	T1, T1c, T2, FLAIR	Artificial neural networks	IN:0u1v	NVIDIA Tesla P100 GPU	n/a	n	n	BT	-	331	1 (BraTS'17)	y	-		DSC
C	2012	S. Bauer et al.	Segmentation of brain tumor images based on integrated hierarchical classification and regularization	MICCAI BRATS	method integrated in neuroradiology workflow		y	T1, T1c, T2, FLAIR	Random Forest, Conditional RandomFfield	n/a	n/a	4-12 min	n	n	BT	-	30?	1 (BraTS'12)	y	-		DSC; Jcc; Sen; Spe; AD; Hdff; Kappa
J	2013	A. J. Asman et al.	Of-Atlas likelihood estimation using multi-atlas segmentation	Med Phys			n	T1c	Intensity probability density function divergence of atlas	REG:FLIRT; IN: 0u1v + intensity mapping	n/a	<2 hrs	n	n	BT	-	30	3	n/a	Manual, 1		DSC; FNR; FPR
J	2008	P. Anbeek et al.	Automated MS-lesion segmentation by K-nearest neighbour classification	MIDAS Journal			y	T1, FLAIR	K-Nearest Neighbours	BE:BET	n/a	n/a	n	n	MS	-	44	3 (Children's Hospital Boston); 3 (University of North Carolina)	y	Manual, 2		VD; AD; TP; FP
J	2017	V. Alex et al.	Semisupervised learning using denoising autoencoders for brain lesion detection and segmentation	Journal of Medical Imaging			Y	T1, T1c, T2, FLAIR, DWI	Artificial neural networks	IN: histogram matching + 0u1v	n/a	n/a	n	n	BT	LGG; HGG; S	257; 28	1 (BraTS'15); 1 (ISLES'15)	y	-		DSC
C	2017	V. Alex et al.	Generative adversarial networks for brain lesion detection	SPIE Medical Imaging			y	T1, T1c, T2, FLAIR, DWI	Artificial neural networks	IN: histogram matching + 0u1v	n/a	n/a	n	n	BT	LGG; HGG; S	22	1 (BraTS); 1 (ISLES'15)	y	-		DSC; Acc
C	2008	G. Agam et al.	Probabilistic brain lesion segmentation in DT-MRI	International Conference on Image Processing			y	T1, T2, DTI	Mixture model, Expectation-Maximization	n/a	n/a	n/a	n	n	S	#VALUE!	21 (+19healthy)	3	n	Manual		PPV; Sen; DSC

References:

van Norden, Anouk GW, et al. "Diffusion tensor imaging and cognition in cerebral small vessel disease: the RUN DMC study." Biochimica et Biophysica Acta (BBA)-Molecular Basis of Disease 1822.3 (2012): 401-407.

Aalten, Pauline, et al. "The Dutch Parelsnoer Institute-Neurodegenerative diseases; methods, design and baseline results." BMC neurology 14.1 (2014): 1-8.

Bakas, Spyridon, et al. "Advancing the cancer genome atlas glioma MRI collections with expert segmentation labels and radiomic features." Scientific data 4 (2017): 170117.

Bowden, Donald W., et al. "Review of the Diabetes Heart Study (DHS) family of studies: a comprehensively examined sample for genetic and epidemiological studies of type 2 diabetes and its complications." The review of diabetic studies: RDS 7.3 (2010): 188.

Reid, Christopher M., et al. "Aspirin for the prevention of cognitive decline in the elderly: rationale and design of a neuro-vascular imaging study (ENVIS-ion)." BMC neurology 12.1 (2012): 1-9.

Chabriat, Hugues, et al. "Predictors of clinical worsening in cerebral autosomal dominant arteriopathy with subcortical infarcts and leukoencephalopathy: prospective cohort study." Stroke 47.1 (2016): 4-11.

Clark, Kenneth, et al. "The Cancer Imaging Archive (TCIA): maintaining and operating a public information repository." Journal of digital imaging 26.6 (2013): 1045-1057.

De Leeuw, F. E., et al. "Prevalence of cerebral white matter lesions in elderly people: a population based magnetic resonance imaging study. The Rotterdam Scan Study." Journal of Neurology, Neurosurgery & Psychiatry 70.1 (2001): 9-14.

Firbank, Michael J., et al. "High resolution imaging of the medial temporal lobe in Alzheimer's disease and dementia with Lewy bodies." Journal of Alzheimer's disease 21.4 (2010): 1129-1140.

Hinton, Ladson, et al. "Recruitment of a community-based cohort for research on diversity and risk of dementia." Alzheimer disease and associated disorders 24.3 (2010): 234.

Hougaard, K. D., et al. "Remote ischemic perconditioning in thrombolysed stroke patients: randomized study of activating endogenous neuroprotection–design and MRI measurements." International Journal of Stroke 8.2 (2013): 141-146.

Hsieh FI, Lien LM, Chen ST, Bai CH, Sun MC, et al. (2010) Get with the Guidelines-Stroke performance indicators: Surveillance of stroke care in the Taiwan Stroke Registry: Get with the Guidelines-Stroke in Taiwan. Circulation 122: 1116–1123.

I-KNOW. "Integrating Information from Molecule to Man: Knowledge Discovery Accelerates Drug Development and Personalized Treatment in Acute Stroke." (2006).

Tremblay-Mercier, Jennifer, et al. "P4-070: A PROGRAM OF PRE-SYMPTOMATIC EVALUATION OF EXPERIMENTAL OR NOVEL TREATMENTS FOR ALZHEIMER'S DISEASE (PREVENT-AD): DESIGN, METHODS, AND PERSPECTIVES." Alzheimer's & Dementia 10 (2014): P808-P808.

Ellis, Kathryn A., et al. "The Australian Imaging, Biomarkers and Lifestyle (AIBL) study of aging: methodology and baseline characteristics of 1112 individuals recruited for a longitudinal study of Alzheimer's disease." International psychogeriatrics 21.4 (2009): 672-687.

Khayati R, Vafadust M, Towhidkhah F, Nabavi SM (2008) Fully automatic segmentation of multiple sclerosis lesions in brain MR FLAIR images using adaptive mixtures method and Markov random field model. Comput Biol Med 38 (3) 379–390.

Lee, J. H., et al. "Identification of pure subcortical vascular dementia using 11C-Pittsburgh compound B." Neurology 77.1 (2011): 18-25.

Prastawa, Marcel, Elizabeth Bullitt, and Guido Gerig. "Simulation of brain tumors in MR images for evaluation of segmentation efficacy." Medical image analysis 13.2 (2009): 297-311.

Machner, Björn, et al. "Impact of dynamic bottom-up features and top-down control on the visual exploration of moving real-world scenes in hemispatial neglect." Neuropsychologia 50.10 (2012): 2415-2425.
Maier, Oskar, et al. "Extra tree forests for sub-acute ischemic stroke lesion segmentation in MR sequences." Journal of neuroscience methods 240 (2015): 89-100.
Reijmer, Yael D., et al. "Microstructural white matter abnormalities and cognitive functioning in type 2 diabetes: a diffusion tensor imaging study." Diabetes care 36.1 (2013): 137-144.
Ropele, Stefan, et al. "Determinants of iron accumulation in deep grey matter of multiple sclerosis patients." Multiple Sclerosis Journal 20.13 (2014): 1692-1698.
Rothwell P.M., Coull A.J., Giles M.F., Howard S.C., Silver L.E., Bull L.M., Gutnikov S.A., Edwards P., Mant D., Sackley C.M., Farmer A., Sandercock P.A., Dennis M.S., Warlow C.P., Bamford J.M., Anslow P., Oxford Vascular S. Change in stroke incidence, mortality, case-fatality, severity, and risk factors in Oxfordshire, UK from 1981 to 2004 (Oxford Vascular Study) Lancet. 2004;363:1925–1933.
Sachdev, Perminder S., et al. "The contribution of twins to the study of cognitive ageing and dementia: the Older Australian Twins Study." International Review of Psychiatry 25.6 (2013): 738-747.
Sachdev, Perminder S., et al. "The Sydney Memory and Ageing Study (MAS): methodology and baseline medical and neuropsychiatric characteristics of an elderly epidemiological non-demented cohort of Australians aged 70-90 years." International psychogeriatrics 22.8 (2010): 1248.
Selnes, P., et al. (2013). Diffusion tensor imaging surpasses cerebrospinal fluid as predictor of cognitive decline and medial temporal lobe atrophy in subjective cognitive impairment and mild cognitive impairment. Journal of Alzheimer's Disease, 33(3), 723–736.
Walker, William C., et al. "The Chronic Effects of Neurotrauma Consortium (CENC) multi-centre observational study: description of study and characteristics of early participants." Brain injury 30.12 (2016): 1469-1480.
Zamboni G., Wilcock G.K., Douaud G., Drazich E., McCulloch E., Filippini N., Tracey I., Brooks J.C., Smith S.M., Jenkinson M., Mackay C.E. Resting functional connectivity reveals residual functional activity in Alzheimer's disease. Biol. Psychiatry. 2013;74:375–383.
Pantoni, Leonardo, et al. "Impact of age-related cerebral white matter changes on the transition to disability–the LADIS study: rationale, design and methodology." Neuroepidemiology 24.1-2 (2005): 51-62.
Shepherd, James, et al. "The design of a prospective study of pravastatin in the elderly at risk (PROSPER)." The American journal of cardiology 84.10 (1999): 1192-1197.
Weiner, MD, Howard L. "Oral tolerance for the treatment of autoimmune diseases." Annual review of medicine 48.1 (1997): 341-351.
"European project on brain morphometry,", BIOMORPH, EU-BIOMED2 project nr. BMH4-CT96-0845, 1996–1998.
Price, Catherine C., et al. "MRI-leukoaraiosis thresholds and the phenotypic expression of dementia." Neurology 79.8 (2012): 734-740.
Zacharaki, E.I., Wang, S., Chawla, S., Yoo, D.S., Wolf, R., Melhem, E.R., Davatzikos, C.: Classification of brain tumor type and grade using MRI texture and shape in a machine learning scheme. Magnetic Resonance in Medicine 62, 1609–1618 (2009)
Marias, K., Dionysiou, D., Sakkalis, V., Graf, N., Bohle, R.M., Coveney, P.V., Wan, S., Folarin, A., Buechler, P., Reyes, M., Clapworthy, G., Liu, E., Sabczynski, J., Bily, T., Roniotis, A., Tsiknakis, M., Giatili, S., Veith, C., Messe, E., Stenzhorn, H., Kim, Y.j., Zasada, S., Haidar, A.N., Bauer, S., Wang, T., Zhao, Y., Karasek, M., Grewer, R., Franz, A., Stamatakos, G.: Clinically driven design of multi-scale cancer models: the ContraCancrum project paradigm. J. Roy. Soc. Interface Fo- cus 1(3), 450–461 (2011)
Wardak, Zabi, et al. "Pre-treatment factors associated with detecting additional brain metastases at stereotactic radiosurgery." Journal of neuro-oncology 128.2 (2016): 251-257.
Victor, Ronald G., et al. "The Dallas Heart Study: a population-based probability sample for the multidisciplinary study of ethnic differences in cardiovascular health." The American journal of cardiology 93.12 (2004): 1473-1480.
Rutten-Jacobs, Loes CA, et al. "Risk factors and prognosis of young stroke. The FUTURE study: a prospective cohort study. Study rationale and protocol." BMC neurology 11.1 (2011): 109.

Links to access the referenced databases

MSC'15	https://smart-stats-tools.org/lesion-challenge
WMH Segmentation Challenge '17	https://wmh.isi.uu.nl/
MS Lesion Segmentation Challenge 2016	https://portal.fli-iam.irisa.fr/msseg-challenge/overview
NACC	https://www.alz.washington.edu/
IBSR	https://www.nitrc.org/projects/ibsr
SPL	https://spl.harvard.edu/software-and-data-sets
BrainWeb	http://www.bic.mni.mcgill.ca/brainweb/
Maier et al. 2015	https://figshare.com/articles/dataset/Sub_acute_Ischemic_Stroke_MRI_scans_T1_T2_DWI_ADC_ground_truth_and_segmentation_results/1585018
Hermes	https://clinicaltrials.gov/ct2/show/NCT00875654
