# Supplementary material for: Automatic brain lesion segmentation on standard magnetic resonance images: a scoping review
Source: BMJ Open. 2021 Jan 28;11(1):e042660. doi: 10.1136/bmjopen-2020-042660 (PMC7849889; doi:10.1136/bmjopen-2020-042660)
Supplement: Author's manuscript [file bmjopen-2020-042660.draft_revisions.pdf]

BMJ Open

BMJ Open is committed to open peer review. As part of this commitment we make the peer review history of every article we publish publicly available.

When an article is published we post the peer reviewers' comments and the authors' responses online. We also post the versions of the paper that were used during peer review. These are the versions that the peer review comments apply to.

The versions of the paper that follow are the versions that were submitted during the peer review process. They are not the versions of record or the final published versions. They should not be cited or distributed as the published version of this manuscript.

BMJ Open is an open access journal and the full, final, typeset and author-corrected version of record of the manuscript is available on our site with no access controls, subscription charges or pay-per-view fees (http://bmjopen.bmj.com).

If you have any questions on BMJ Open's open peer review process please email info.bmjopen@bmj.com

BMJ Open

Automatic brain lesion segmentation on standard magnetic resonance images of the human head: a scoping review

Journal:	*BMJ Open*
Manuscript ID	bmjopen-2020-042660
Article Type:	Original research
Date Submitted by the Author:	11-Jul-2020
Complete List of Authors:	Gryska, Emilia; Goteborgs universitet Institutionen for kliniska vetenskaper, Radiation Physics<br>Schneiderman, Justin; Goteborgs universitet Institutionen for neurovetenskap och fysiologi, Sektionen för klinisk neurovetenskap<br>Björkman-Burtscher, Isabella; Göteborgs universitet Institutionen för kliniska vetenskaper, Radiology<br>Heckemann, Rolf; Goteborgs universitet Institutionen for kliniska vetenskaper, Radiation Physics
Keywords:	RADIOLOGY & IMAGING, Magnetic resonance imaging < RADIOLOGY & IMAGING, Diagnostic radiology < RADIOLOGY & IMAGING, Neuroradiology < RADIOLOGY & IMAGING

SCHOLARONE™
Manuscripts

I, the Submitting Author has the right to grant and does grant on behalf of all authors of the Work (as defined in the below author licence), an exclusive licence and/or a non-exclusive licence for contributions from authors who are: i) UK Crown employees; ii) where BMJ has agreed a CC-BY licence shall apply, and/or iii) in accordance with the terms applicable for US Federal Government officers or employees acting as part of their official duties; on a worldwide, perpetual, irrevocable, royalty-free basis to BMJ Publishing Group Ltd ("BMJ") its licensees and where the relevant Journal is co-owned by BMJ to the co-owners of the Journal, to publish the Work in this journal and any other BMJ products and to exploit all rights, as set out in our licence.

The Submitting Author accepts and understands that any supply made under these terms is made by BMJ to the Submitting Author unless you are acting as an employee on behalf of your employer or a postgraduate student of an affiliated institution which is paying any applicable article publishing charge ("APC") for Open Access articles. Where the Submitting Author wishes to make the Work available on an Open Access basis (and intends to pay the relevant APC), the terms of reuse of such Open Access shall be governed by a Creative Commons licence – details of these licences and which Creative Commons licence will apply to this Work are set out in our licence referred to above.

Other than as permitted in any relevant BMJ Author's Self Archiving Policies, I confirm this Work has not been accepted for publication elsewhere, is not being considered for publication elsewhere and does not duplicate material already published. I confirm all authors consent to publication of this Work and authorise the granting of this licence.

For peer review only

Automatic brain lesion segmentation on standard magnetic resonance images of the human head: a scoping review

Emilia Gryska[1,2*], Justin F. Schneiderman[1,3], Isabella Björkman-Burtscher[4], Rolf A. Heckemann[1,2]

1 MedTech West at Sahlgrenska University Hospital, University of Gothenburg, Sweden

2 Department of Radiation Physics, Institute of Clinical Sciences, University of Gothenburg, Sweden

3 Department of Clinical Neurophysiology, Institute of Neuroscience and Physiology, Sahlgrenska Academy, University of Gothenburg, Sweden

4 Department of Radiology, Institute of Clinical Sciences, Sahlgrenska Academy, University of Gothenburg and Sahlgrenska University Hospital, Gothenburg, Sweden

* Corresponding author: Emilia Gryska

Postal address: MedTech West, Röda stråket 10B, Sahlgrenska University Hospital, 413 45 Göteborg

E-mail address: emilia.gryska@medtechwest.se

Telephone number: +46 720304106

Keywords: Scoping review, Brain lesions, Segmentation, Magnetic resonance imaging

Word count: 6 828

Author contributions: EG conducted the study and led writing of the article. RAH was the main supervisor and consultant of the study progress and design choices. JFS provided input on the study plan and methodology at all stages. IBB provided guidance on the interpretation of the findings from a clinical perspective. All co-authors collaborated on manuscript composition and editing.

Funding: JFS is supported by the Swedish Childhood Cancer Foundation (MT2018-0020) and the ATTRACT project funded by the EC under Grant agreement 777222.

Competing interests: None declared

Supplementary data: The data extracted for the study can be found in FigShare repository, DOI: 10.6084/m9.figshare.12639842

For peer review only

ABSTRACT

Objectives Medical image analysis practices face challenges that can potentially be addressed with algorithm-based segmentation tools. The standard practices of algorithm development and validation, however, have raised doubts. In the proposed study, we map the field of automatic brain lesion segmentation and examine clinical relevance of the published research.

Design Scoping review.

Setting Three databases (PubMed, IEEE Xplore, and Scopus) were searched with tailored queries. Studies were included based on predefined criteria and emerging themes during consecutive title, abstract, methods, and whole-text screening. The full-text analysis focused on materials, preprocessing, and performance evaluation and comparison.

Results Out of 2990 unique articles identified through the search, 441 articles met the eligibility criteria, with an estimated growth rate of 10% per year. We present a general overview and trends in the field with regard to publication sources, segmentation principles used, and types of lesions. Algorithms are predominantly evaluated by measuring the agreement of segmentation results with a trusted reference. Few articles describe measures of clinical validity.

Conclusions The observed reporting practices leave room for improvement with a view to studying replication, method comparison, and clinical applicability. To promote this improvement, we propose a list of recommendations for future studies in the field.

ARTICLE SUMMARY

Strengths and limitations of this study:

- We map the extensive landscape of the field following a rigorous published protocol.
- We scrutinize the field for clinical applicability of the proposed brain lesion segmentation methods and validation procedures.
- We propose a checklist of items to report for future studies to facilitate study replication, method comparison, and reduce the divide between the number of developed methods and scarce clinical implementation
- The broad and inclusive nature of a scoping review combined with variable design and reporting of the reviewed studies imposes unavoidable bias at the study inclusion stage.
- We acknowledge some epistemic drift that occurred while conducting the study, however, we account for all the changes made to the published protocol and justify our choices by the value our study delivers to the field.

INTRODUCTION

Magnetic resonance imaging (MRI) has become an integral part of diagnostics to rule out or differentiate brain lesions. Properties that determine this success are safety, high tissue contrast, and sensitivity to abnormality. Conditions that can lead to brain lesions include traumatic brain injury, vascular disease (including ischemic and hemorrhagic stroke), neoplasms, autoimmune disorders, infection, degenerative diseases, congenital conditions, and systemic diseases with secondary effects on the central nervous system. In some of these conditions, accurate estimation of lesion size and its progression may be essential to treatment planning, disease monitoring, and/or treatment evaluation.

Visual image interpretation is still the most common and accepted way to analyse clinical images. In some cases, the visual examination must be extended to include lesion delineation. For example, the boundaries of a tumour and structures at risk need to be localized accurately for radiation therapy planning. Such delineation depends on a skilled rater, is time-consuming, and is characterized by high variability between raters and high variability between repeated delineations on the same images by the same rater (i.e., low objectivity and reliability, respectively). In

For peer review only

other clinical examinations, surrogate metrics, which can be based on the largest perpendicular diameters [1,2] or visual scoring [3], are used for estimating the size of a lesion rapidly. Such visual or area-based lesion volume estimates may not be accurate enough [4].

Developments in magnetic resonance (MR) image acquisition procedures resulted in high-quality and complex images that capture a variety of structural and functional phenomena. On the one hand, the images carry more information about the lesions, thereby enabling more accurate diagnosis. On the other hand, each image requires more analysis time. This reduces the rate at which radiologists can analyse images. The increasing accessibility to MR scanners furthermore results in more images being acquired, further straining image processing capacity and potentially diminishing the quality of interpretation [5,6].

To summarize, three major issues with current clinical practices for image analysis are: subjective and time-consuming manual segmentation, limited accuracy of the surrogate volume estimates, and increasing information content and number of images to process.

Automatic lesion segmentation algorithms promise to alleviate these issues through fast and consistent lesion delineation that scales with demand. Quantitative image analysis using automatic lesion segmentation further has the potential to increase diagnostic and prognostic accuracy of lesion examination by providing radiologists with prompt and explicit information about a lesion.

Several reviews on automated brain lesion segmentation methods have previously been published. Most of these outline and analyse commonly used segmentation algorithms or general segmentation principles [7–16]. The perspective of the clinical applicability of automated segmentation algorithms has been explored by Garcia-Lorenzo et al. [10] and Bauer et al. [7]. In both of these reviews, the authors recommend improvements concerning study designs and formulation of research questions. The authors stress the importance of validating the robustness of the segmentation algorithms under variation stemming from three causes: differences between scanners and acquisition protocols, natural variability in normal anatomy and lesion appearance, and artifacts. Moreover, Bauer et al. expressed the need for better communication between researchers developing segmentation methods and clinical radiologists who are the intended users of these methods.

A comprehensive review of automatic segmentation methods without restriction to lesion type is still lacking today, perhaps because of the challenge associated with the large and growing literature. We therefore present a scoping review of such methods. Given the rapidly growing extent of the literature, this work provides a timely account of the field that may not be feasible in the future. To ensure rigour, reproducibility, and comprehensiveness, we adopted the scoping review method proposed by Arksey and O'Malley [17] and further developed by Levac et al. and Colquhoun et al. [18,19]. We also incorporate relevant parts of the PRISMA [20] and PRISMA-ScR [21] protocol for this type of review.

Furthermore, there is a disconnection between developers and users of automatic brain lesion segmentation methods [7]. We therefore focus on examining clinical relevance of the research conducted in the field. We seek to understand how the most prevalent methods (segmentation algorithms) and study designs (how they are validated) reflect the clinical applicability of methods described in the reviewed articles. Moreover, we aim to identify issues, limitations, and grand challenges of the field, and suggest actions to bridge the gap between research and clinical practice.

METHODS AND DESIGN

This study has been conducted according to a previously published protocol [22]. Here we provide a summary of the protocol as well as an account of changes made, along with their respective rationale.

Stage 1: identifying research questions

The following research questions (RQs) were posed in the protocol [22]:

1. Which common image processing steps are necessary for automatic brain lesion segmentation on MR images?

For peer review only

2. Which mathematical and computational theories are most commonly applied in which types of brain lesions?
3. What is the efficacy of existing implementations?
4. What are the limitations of those methods and issues that should be addressed in future studies to develop a tool that is suitable for clinical use?
5. What are the most commonly used MR data sets that provide reference lesion segmentation and/or diagnostic classification?

Upon getting familiar with the sample we questioned the relevance of RQ 1 and 2. We recognised the need for consultation at this early stage of the project as opposed to consultation on the findings as originally proposed by Arksey and O'Malley [17]. The RQs do not reflect fully the potential of our study to address issues critical to advancing the field and bringing a benefit to the community. The consultation was conducted as semi-structured interviews with five clinicians who have experience in brain image analysis. We interviewed two neurosurgeons, an oncologist, a radiation oncologist, and a neuroradiologist. The interviews were structured to elicit understanding of their daily workflow, of how they use and analyse images, and of their opinions on automating the process of brain lesion segmentation.

Through the consultation and screening of a subsample of abstracts (Stage 2, cf. Section 2.2), new aspects related to clinical applicability of the proposed methods emerged. Next to identifying the most common image processing steps and respective principles or theories applied for brain lesion segmentation, we sought to examine the clinical relevance of the validation procedures and the resultant performance of the methods. Moreover, following the aims of a scoping review, we want to identify overarching characteristics and grand challenges of the field.

Stage 2: identifying relevant articles

For the purpose of this study, we define an article as an individual published paper that was found according to the presented search strategy and that meets the inclusion criteria of this review.

Eligibility criteria were unchanged from those defined in the protocol. We thus looked for articles that evaluate an automatic brain lesion segmentation method applied to MR images. The images must be acquired from human patients examined for a condition causing brain lesions. The performance of the method must be evaluated by comparison with a reference segmentation.

The initial search strategy remained mostly unchanged. We used the articles we identified according to the procedure described in the protocol. Three databases (PubMed, IEEE Xplore, and Scopus) were queried with search phrases to identify articles dealing with automatic brain lesion segmentation methods on MRI. Initially, a broad search phrase was used to generate a first sample for each database. Controlled vocabulary tags were extracted from the articles and arranged in descending order of frequency. From this list, tags were selected to refine and customize the search phrase for each database. These refined and customized search phrases were used to retrieve the sample for our study. The search was conducted on 4th of November 2018.

The relevant articles were identified through hierarchical screening at four levels: 1) title; 2) abstract; 3) methods; 4) full paper screening. The aim of the title screening phase was to identify and exclude clearly ineligible articles. At the abstract screening phase, we refined the inclusion and exclusion criteria based on the literature content with the following algorithm. We read randomly selected abstracts out of a sample of 1359 until we identified 100 that were considered eligible for the study. Themes that would guide the study selection process (Stage 3) were gleaned from this sub-sample. We identified two core concepts: algorithm validation and applicability of an automatic segmentation method for clinical parameter prediction. All studies that dealt with the latter concept were included unless the abstract indicated that manual or semi-automatic segmentation was used. We excluded articles that fell under the 'algorithm validation' category if information about the source of the tested images or patients was missing, if only synthetic images were used, if the algorithm validation was conducted on fewer than 10 unique scans/images, or if the results of the algorithm's performance were not provided. A detailed description of the full paper screening procedure is presented in Stage 3: study selection section. The same hierarchical screening procedure was applied to references of the eligible sample to identify additional articles that were not returned in the original search. At each stage, we screened for duplicates and compared papers of the same authors to exclude

For peer review only

methods presented in conference proceedings that were later published in journal articles if the patient cohort was similar in both articles.

Stage 3: study selection

In the full-text screening, we applied the above criteria again. In addition, we looked for the information about how a gold standard or a reference segmentation was obtained, and about the number of unique 3D scans that were used for the validation of a method if this information was missing from the abstract. We excluded studies conducted on fewer than 10 unique scans of the same acquisition sequence, as well as studies where individual image sections were manually selected from volume images. For increased efficiency, we read full texts in non-standard order (method section first) and discontinued the screening upon finding an exclusion criterion. To summarise, we excluded papers that:

- did not perform segmentation or did not provide segmentation evaluation outcome measures (wrong outcome)
- proposed methods that require user interaction
- did not provide information about the gold standard (GS) or a reference measurement
- did not provide information about the origin of the images or used only synthetic images
- did not provide the number of images used for evaluation
- evaluated the method on fewer than 10 unique scans.

Stage 4: data charting

The data extracted from the sample were charted in a spreadsheet with one row per article and one column per variable. The extracted variables fell into five categories: bibliographic, segmentation method, validation, study cohort, and interesting findings. The bibliographic category included first authors' name, article title, journal or conference name, and year of publication. For the segmentation category, we extracted data regarding the preprocessing procedures, segmentation principles, required or allowed input modalities, processing time of the method, and availability of the software. In the validation category, we looked for information about the process of obtaining reference segmentations, the number of raters or observers, and evaluation metrics used. The study cohort category comprised patients' diagnoses, lesion type, sources, and the number of images used. The final category was a catch-all for information that did not fit any of the other categories, but was still considered relevant in view of the aims of our scoping review. The spreadsheet is available as supplementary information.

Stage 5: collating, summarizing, and reporting the data

The collected data is disseminated through numerical analysis of bibliographic information overview, trends in the field, and study design characteristics. We provide the account of both prevalent study design choices and less common ones that are relevant to the clinical applicability assessment. Study design features are grouped into materials, preprocessing, performance evaluation, and performance comparison.

Patient and Public Involvement

No patients nor members of the public were involved in this research project.

RESULTS

Overview

Among 2500 articles returned by the searches, and additional 490 titles identified through reference screening, we included 441 articles in the review. The number of papers included at each stage of the review is shown in Figure 1. Among the eligible papers, 254/441 were published in journals and 185/441 in conference proceedings or conference workshops, and 2/441 articles were uploaded as preprints. The following journals or conferences

For peer review only

occurred most frequently as the publication source: International Conference on Medical Image Computing and Computer-Assisted Intervention (66), NeuroImage (20), IEEE Transactions on Medical Imaging (20), SPIE Medical Imaging Conference (18), NeuroImage: Clinical (15), IEEE International Symposium on Biomedical Imaging (13), and PloS One (12).

Figure 2 shows the distribution of papers that propose a method to segment a particular brain lesion type. The dominance of methods segmenting brain tumours is evident from the data. Among the most commonly used algorithms or models in the segmentation methods (Figure 3), were artificial neural networks, decision trees, random fields, and thresholding. The analysis accounted for the same methods published in separate articles that deal with either different lesion types or similar lesion type with different image sources.

Trends

Substantial growth of the field was evident from the number of studies published annually (Figure 4). Three distinct periods with an increase in the number of published studies compared to preceding years were noticeable. During these years, an increase is also evident in the number of articles addressing segmentation of lesion types corresponding to the challenges taking place. The first wave corresponds to the *3D Segmentation in the Clinic: A Grand Challenge II: MS Lesion Segmentation* [23]. The second wave started in 2012 coinciding with the advent of *The Multimodal Brain Tumor Image Segmentation Benchmark (BraTS)* [24]. The third wave started in 2015 when three brain lesion segmentation challenges took place: BraTS, the *Longitudinal MS Lesion Segmentation Challenge* [25], and the *Ischemic Stroke Lesion Segmentation challenge (ISLES)* [26].

Study design

Materials

The majority of the proposed methods operated on multi-sequence scans as inputs (307/441) while 6 accepted both single- and multi-sequence input. 79 methods were built to evaluate single-sequence input and 22 did not specify the input modality. Articles that compared or evaluated a previously proposed method were not included in the analysis (27/441). Among image sources for methods validation, independently collected, non-public data sets were used in 217 articles, out of which 45 also used a publicly available data set (such as provided in a segmentation challenge) next to the independently acquired one. The *The Multimodal Brain Tumor Image Segmentation Benchmark (BraTS)* [24] database was the second most common source of images (157 articles), followed by *3D Segmentation in the Clinic: A Grand Challenge II: MS Lesion Segmentation* [23] (29). The remainder of articles used images coming from other various sources, or the sources are not clearly indicated.

Among the articles that perform validation on a non-public data set only, 72 do not mention the specific cause of the examined lesion. The distribution of the cohort sizes used to validate the proposed methods (Figure 5) shows that more than half of the studies described in included papers test methods on 50 or fewer individuals' brain images (260/441). Among the eligible papers, 280 describe a method validated on images obtained from more than one scanner.

Preprocessing

We identified the following most commonly applied preprocessing procedures and tools (disregarding publicly available databases for this purpose): bias field correction (192, N3 and N4 [27,28]), brain extraction (190, BET [29]), intensity normalization (224, histogram matching and or intensity rescaling), image (co-)registration (179, rigid, affine, SPM, FSL [30,31]), denoising (60, anisotropic diffusion filtering). 67 articles did not provide information about preprocessing procedures. Although authors commonly document the individual steps and corresponding algorithms, information on whether these algorithms are integrated in the segmentation tool is rarely available [32,33]. 15 papers mention visual supervision, semiautomatic preprocessing, manual correction, or preprocessing failure (usually at the brain extraction step) [34–48].

Five methods proposed in the reviewed articles use minimal [49,50] or no preprocessing [51–53], and in each case this is presented as an advantage of the proposed algorithm.

For peer review only

Performance evaluation

The reference segmentations of the lesions in independent data sets were mostly obtained manually by one rater (84) or two raters (67). More than two raters contributed to constructing the reference segmentations in 33 articles, and 28 articles used semiautomatic procedures for constructing the reference. When multiple raters delineated the region of interest, the reference segmentation was obtained through consensus of the raters or majority voting. 22 articles reported obtaining the reference segmentation through delineation of the lesion by one rater supervised by another with possible corrections. Although we did not include the number of years of raters' experience in our data charting categories, in retrospect we found only a few articles that provide such information (e.g. [54]). 45 articles have also included inter- and intra-rater variability evaluation on data sets used in their studies.

Comparison with the reference by overlap measures was by far the most commonly used criterion to evaluate the accuracy of the automatic segmentation. The most frequently applied measures were the Dice coefficient (330), sensitivity, also expressed as true positive ratio TPR, or overlap fraction (227), positive predictive value (102), Jaccard coefficient (58), and specificity (82). Volumetric measures, such as volume difference, error or correlation between the automatic and reference segmentation were used in 133 articles, and distance-based measures (such as Hausdorff distance, mean absolute distance, surface distance) in 41.

In addition to segmentation accuracy, reliability of a method was estimated in some of the articles. Reliability was directly evaluated through a test-retest procedure, where participants were scanned twice at a short interval, with repositioning between the scans in five articles [55–59]. In other articles, indirect approaches were described where consistency of longitudinal data with clinical findings of stable or progressive disease was used as a proxy for reliability [60–63].

Performance comparison

Maldjian et al. (2013) [46], Lesjak et al. (2016) [64], and de Sitter et al. (2017) [65] examined previously proposed methods and compared the results they obtained in their studies to the performance reported in the original papers. All three articles reported poorer independent testing results. The authors pointed out a lower lesion load of the studied population than in the original papers that accounted for reduced scores in the replication attempts. In their paper, de Sitter et al. (2017) [65] strongly called for improvements to automatic lesion segmentation before their introduction to routine clinical use.

Of 441 articles, 233 included information regarding both the processing time and computational system requirements used for segmentation. In 56 articles we found information about only one of the two parameters, however such information is incomplete and cannot be used to estimate how well a method will perform on different hardware.

Only 24 of the included articles reported on methods that were made publicly available to download [32,33,59,66–87]. One was available upon request [88]. Two articles promised future availability of the methods proposed [89,90] but they were not available as of 4th of May 2020.

DISCUSSION

In this paper, we present a scoping study of automatic brain lesion segmentation on magnetic resonance images based on rigorous literature review methodology [17]. This is the first review that takes all methods into account, independent of specialization towards lesions of a particular etiology. The key findings from our analysis of 441 articles are a) a rapid increase of interest in the field, b) a plethora of proposed methods contrasted with a dearth of evidence of successful clinical implementation and clinical usability, and c) high prevalence of problematic reporting practices that restrict the ability of independent researchers to replicate reported results and conduct method comparisons.

Variable design of automatic segmentation methods

Our first research question concerned imaging processing steps necessary for brain lesion segmentation. Image preprocessing emerged as a pervasive step in the processing chain for lesion segmentation. In five articles, however, authors present no or minimal need for preprocessing as an advantage of their respective methods. From the

For peer review only

collected data we identified procedures that are commonly agreed to constitute preprocessing: skull stripping, image co-registration, bias field correction, and intensity normalization. Some algorithms rely on additional preprocessing, such as tissue classification. The procedures vary considerably between methods, and a lack of a universal distinction between segmentation steps and preprocessing steps became apparent during the analysis.

For a clinically suitable method, such a distinction may not be necessary since the final segmentation will rely on a whole processing chain (including the preprocessing) applied to a raw image. Validation of the whole chain, and assessing the impact of each step on the outcome, however, are desirable in method evaluation studies. Unfortunately, authors rarely state whether preprocessing is integrated with the segmentation algorithm and to what extent it relies on user input. Without such information, namely a list of all the steps that are performed on a raw image, the study cannot be replicated. This implies that the findings are of no help in assessing the potential clinical validity of the proposed method.

In the second research question, we ask which mathematical and computational theories are most commonly applied for brain lesion segmentation. The most prevalent were artificial neural networks. The finding is not surprising given the popularity and remarkable performance of deep learning algorithms particularly in image processing applications. The research question and the findings, however, turned out not to have a high significance for this review. Due to a lack of meaningful way to compare performance of methods that rely on various theories (section *Validation process and efficacy of automatic segmentation methods*) it is difficult to establish if one model or algorithm exhibits superior performance for the lesion segmentation task and meets the clinical applicability requirements.

Validation process and efficacy of automatic segmentation methods

In the third research question we asked about efficacy of the implementations of automatic segmentation algorithms. We found that in view of current practices, the question cannot be answered: authors present assessments that are predicated on their own needs and biases, and there is no established standard that enables fair method comparison. Public challenges have been set up in an effort to address this problem, but they have only been partially successful, as illustrated in the following section. The lack of ground truth and the variability of both the reference segmentation and the image features due to the qualitative nature of MRI additionally impose a limit to which efficacy can evaluated.

To answer the question we posed regarding the efficacy, we recorded the values of the comparison measures from each article. The information, however, cannot be meaningfully synthesised or compared and thus was not presented in the results section. Our investigation did not result in a direct answer to the question, but revealed a paucity of standard comparison procedures.

Typically, the stated aim of the sampled articles is to propose a lesion segmentation method and to present validation results. The standard validation procedure is to apply the method to a set of cross-sectional images. The automatic segmentation is compared with a reference segmentation that is most commonly produced by one (84/441) or two (67/441) experts carrying out manual delineation of the region of interest. Comparison measures that are typically employed are spatial overlap, volume correlation or volume error, and surface distance. Automatic-to-manual comparison results are then compared to the inter-rater agreement or commonly reported thresholds proposed in previous studies [24,91].

Inability to answer the posed question points to further issues on which we reflect from the perspective of clinical suitability of the proposed methods. The medical image analysis procedures commonly used in clinical diagnostics today are strongly operator-dependent. They also scale poorly to the growing workload that results from the increasing number of modalities [92], the increasing accessibility of imaging scanners [93], and the increasing amount of image information due to technical advances that achieve enhanced spatial and contrast resolution [94]. Automatic image analysis methods in general, and among them automatic lesion segmentation methods promise to alleviate some of this pressure. Other requisite characteristics that need to be assessed to evaluate the clinical applicability of a tool are reliability, robustness, and generalizability of its findings.

Reliability of an automatic segmentation algorithm seems to be assumed since the decision whether a given voxel is a lesion or not is made based on well-defined rules. However, the characteristic is rarely tested in method validation studies. Only five articles in our sample included a test-retest evaluation with patient repositioning between the

For peer review only

scans. Such testing yields crucial reliability data against which longitudinal variations of lesion measures need to be compared to distinguish actual lesion change from other sources of variability.

Generalizability of a method is tested by processing images acquired from different scanners on a sufficient number of images from populations that are at least as heterogeneous as the population expected to be examined clinically. While more than half of the articles (280/441) used images coming from more than one scanner, validation on fewer than 50 images were equally prevalent (260/441). Such small sample sizes are insufficient for clinical validation, even if the images originate from multiple scanners. Using images acquired from multiple sources and validating a method on a large number of images reduces a method's bias towards the data set or sets on which a given method was originally developed.

To us, one of the most surprising findings was how few methods are publicly available. Authors who share their software enable independent testing in unanticipated conditions and with various cohorts. Finally, to evaluate clinical relevance of the results of automatic segmentation, a method should be tested in genuine clinical scenarios for a well-defined task, such as monitoring tumour progression, treatment response evaluation, or radiotherapy planning. Each of these tasks may require a different level of error margin and validation standard and the relevance of the results must be evaluated by clinicians. Many papers claim usefulness of their algorithm for particular clinical tasks, but do not test the acceptability of the results for the intended purpose.

Limitations and issues of the proposed methods, and grand challenges of the field

As in many validation tasks around *in vivo* image analysis, the inherent lack of ground truth poses a problem [24,95–100]. Currently, the gold standard is a delineation made by an expert rater or a fusion of multiple such delineations. Information about the training or experience was not commonly provided. The validation process is limited by unavailability of ground truth and variability of the reference segmentations. More effort should thus be devoted to increasing the informativeness of the validation step. This can be achieved by painstakingly reporting the number of annotators, their experience, how the delineations were fused, and all other details that could conceivably have an impact. According to Gibson et. al [101], such information can be used to calculate the statistical power of segmentation studies with respect to the number of reference images and how inexperienced annotators impact the sample size effect.

Due to the inherent reference, image, and lesion variability, a meaningful comparison of algorithms evaluated independently on separate data sets to establish which method performs better is challenging. One solution to the problem has been proposed in the form of segmentation challenges. The organizers publicly provide images while keeping expertly prepared associated reference segmentations secret. Participants enter the challenge by submitting segmentations. Organizers evaluate the submissions by comparison with the secret reference and return the comparison results. The images are preprocessed to enable comparison between segmentation algorithms and to remove information that could identify the imaged participant. The extent of preprocessing steps and their description varies between challenges. The steps that are always performed are co-registration of sequences, skull-stripping or defacing, and resampling. The images from *BraTS* [24] were rigidly co-registered and skull-stripped with tools available in the *Insight Toolkit* [102], and resampled to an isotropic voxel size of 1mm using linear interpolation. Images for the WMH segmentation challenge [103] were co-registered using the *elastix toolbox* [104,105], manually de-faced, and resampled to 3mm thickness in the transverse direction. No information about the interpolation method is supplied. In the *MS segmentation Grand Challenge II* [23], rigid registration was used to transform the T1-weighted images to the *MNI* reference [106,107] and remaining structural sequences to the T1-weighted images. Nonrigid registration was used, however, to register DTI images to T2. Resampling of the images to an isotropic voxel size of 0.5 mm was done with cubic spline interpolation. The images were anonymized using an undocumented procedure. The images for ISLES challenge [26] were co-registered with the *elastix toolbox* [104,105], skull-stripped using BET2 [108] with manual corrections when necessary, and resampled to two isotropic voxel sizes for the two challenge tasks with B-spline interpolation. The challenges have become a quasi-standard for comparing brain lesion segmentation algorithms. The setup, however, comes with certain shortcomings with respect to evaluating clinical applicability of the evaluated algorithms. As mentioned, evaluating the whole chain of processing steps is especially important for validation of a clinical tool. In a challenge setup, the algorithms are tested on partially preprocessed images. Even when the individual steps are described in detail, the impact of the preprocessing on the segmentation algorithms outcome is unknown. Validation of the processing chain becomes questionable if a step is changed, either based on the users' subjective judgement or due to implementation of a different procedure for a particular step. According to *ISLES* [26] and several articles in our sample, the skull-

For peer review only - http://bmjopen.bmj.com/site/about/guidelines.xhtml

stripping step may need to be supervised and manually corrected. The choice of interpolation method is yet another issue that can influence the outcome of an automatic segmentation algorithm, especially in tasks requiring high accuracy. Another aspect of the challenges is the prestige and publicity for the authors and their method after proposing a winning segmentation algorithm. The final rank is calculated by the organizers who evaluate the methods on hidden data sets. Organizers effectively take the role of independent arbiters. Participation in challenges and post-contest use of the challenge data as reference material indicate that organizers are generally trusted in this role. Maier-Hein et al. suggest, however, that the results of such competitions should be considered carefully [109]. The authors point to several issues that have a significant impact on the final ranking of evaluated methods. The first problem they report is lack of thorough reporting of relevant information that is essential for result interpretation. Moreover, it turns out that changes in metrics and aggregation methods for the scores of individual test cases alter the ranking of evaluated methods. A similar effect on the ranking was observed when the reference segmentations were exchanged against those of another rater. The final rank in such a competition depends also on the test data and on how missing data is handled.

These concerns cast doubt on the validity of testing clinical applicability of automatic brain lesion segmentation methods in a challenge setup. Even if the challenge databases contain images from multiple scanners, it cannot be regarded as generalizable if it has not been tested on raw images (reconstructed, but not otherwise processed) as produced by the scanning equipment. In our sample, only 10% of the articles explicitly report on such evaluation. While obtaining independent data sets is expensive, it is a crucial step on the way of creating a clinically applicable automatic lesion segmentation tool.

Ultimately, it is the target users who need to decide whether a given validation standard and resultant performance confidence are sufficient to apply a tool to answer a given clinical question. With guidance from support system developers, as well as transparent and thorough reporting of the processing steps and validation procedures, the users may consider a given tool trustworthy. Trustworthiness has been reported as a crucial factor in developing a clinically usable support tool [110]. The present scoping review does not show what properties and features clinicians need to develop trust in a tool. Still, this is an important question that we will seek to address in future work.

We conducted an exploratory study to begin to address the issue [111]. The aim was to learn how considering radiologists' competent input can improve the design and validation procedures of automatic brain lesion segmentation methods with a view to increasing trust and trustworthiness. Our findings corroborate previous findings [110]. We found that two crucial characteristics that influence clinicians' trust in a tool are the provision of an error margin with any quantitative measure, and consideration of the varying need for accuracy, depending on the diagnostic task.

Relevance for research and clinical practice

Noteworthy trends also emerged from the data regarding the prevalence of methods developed for segmentation of a particular lesion type, as well as the advent of segmentation challenges focussing on corresponding lesion types. We observed that the majority of the methods described in our sample have been developed for the purpose of brain tumour segmentation. We also note consistent growth of the number of images in the database underlying the *BraTS* challenge, which has been organised annually since 2012. Stroke lesion segmentation methods have not proliferated to the same extent, despite similar competitions having been organized (yearly *ISLES* challenges from 2015 to 2018). A distinct increase in the number of publications proposing white-matter lesion segmentation algorithms was observed when segmentation challenges of this type of lesion were organized.

Some compelling questions follow this observation, e.g. whether some tasks are more difficult to solve algorithmically, or whether these trends reflect on clinical usefulness of developing segmentation algorithms for a particular lesion type. Even though the questions cannot be directly answered by our findings, they point to important issues that need to be explored to better understand the relevance of different lesion segmentation algorithms and implications of the trends for clinical practice.

Another key factor in developing a clinical tool is obtaining regulatory approval for diagnostic use. None of the articles in our sample mentioned this requirement, possibly because authors do not consider it relevant at the stage of development when publication occurs.

For peer review only

It appears that despite claims of clinical relevance made by many authors, development of brain lesion segmentation methods happens predominantly in an academic space, where technological challenges matter most and implementation hurdles are not explicitly considered. To increase the relevance and societal benefit of method development in the field, it will be necessary for developers to widen their perspective to include the critical path towards clinical implementation, on which users' demands and regulatory requirements have to be met.

Challenges and limitations of the study

The biggest challenges we had to address in this study were designing data charting categories, extracting the relevant information regarding both inclusion and exclusion criteria, and the actual charting of the data. Arksey and O'Malley [17] recommend deciding the inclusion criteria after becoming familiar with the identified relevant studies. For us, this was impossible due to the large size of the raw sample of original search results, along with the variability of both the type and the level of detail of reported information.

Our inclusion and exclusion criteria were defined based on a pilot analysis of articles selected randomly from the raw sample. These criteria were then applied to the full raw sample in hierarchical fashion. A major impediment at this stage was the inconsistent way the information is organized and presented in the articles. In the case of conference papers, the authors typically have to adhere to page limits, so certain compromises are inevitable. When it comes to journal publications, the methodology of a study should be thoroughly reported for the sake of reproducibility, but also to enable data extraction for systematic reviews.

Another challenge we faced and a limitation of the study comes from the mentioned incoherent way to report studies and a challenge to derive strict definitions of the inclusion and exclusion criteria. Often, the criteria we sought in the articles are not presented clearly or the information is scarce. Keeping in mind the broad nature of a scoping review and the aim of mapping the field, the author conducting the scoping study (EG) chose to err on the side of inclusion if the information in an article did not allow a decisive application of the criteria. While this strategy, combined with a single-rater approach, introduces a certain amount of selection bias, this bias was at least consistent between articles. We believe the approach provides a reasonable tradeoff between transparency and reproducibility of the study, and fulfilling the objectives of conducting a scoping review.

We acknowledge self-critically that, due to the extensive nature of the task, the time taken, and the lessons learned during the work, some epistemic drift occurred, taking our focus away from the first two research questions and towards the question of clinical applicability that appears to be underserved by the current literature. Thanks to our early decision to hold ourselves to account by writing a detailed protocol [22], this drift stayed within reasonable boundaries and is well-documented, as we accounted for protocol modifications in the present work.

Recommendations for future work

To address the shortcomings revealed by our analysis, we propose a set of recommendations for future studies as well as avenues researchers might follow that promise, in our estimation, to advance the field.

The most important recommendation arises from the variability encountered in many aspects of the field. We propose a checklist (Table 1) of items that should be reported in investigations of automatic brain lesion segmentation methods. This checklist will facilitate replication as well as comparisons between methods and studies, both informal and in meta-analyses. If authors were to follow the checklist in future studies, this would be a step towards standardization of reporting in the interest of advancing knowledge and promoting implementation as clinical tools.

Table 1: Proposed reporting items for automatic brain lesion segmentation studies.

**Method**	List of processing steps necessary to apply to a raw image
	Computational system parameters
	Computation time
	Availability of the method
	Compatibility with picture archiving, communication, and storage (PACS) systems

For peer review only - http://bmjopen.bmj.com/site/about/guidelines.xhtml

**Reference segmentation**	Number of raters
	Raters' training/experience
	Method of segmentation
	Method of consolidation (if multiple raters)
**Validation**	List of validation metrics used
	Number of images (split into training/validation/testing if applicable)
	Input sequences
	Number of scanners used to acquire images
	Acquisition parameters
	Number of time points and intervals for longitudinal data
**Results**	Mean, median, standard deviation for each validation metric
	Number of failed cases (if applicable)
**Patient information**	Diagnosis and level of verification e.g. clinical follow-up, tissue sampling, autopsy, etc.
	Clinical presentation
	Administered treatments (if applicable)

With similar priority, we ask researchers who have developed or are working on automatic brain lesion segmentation algorithms to publish software implementations. The benefits and challenges with fully automated versus interactive preprocessing should be assessed in terms of segmentation accuracy and reproducibility for a given clinically relevant task.

We found relatively few articles that evaluate their algorithm on an independent data set on top of the challenge one. We encourage authors to endeavour to obtain independent images to test their method. We also encourage collaboration between the authors and independent researchers who may have access to annotated test images. A preprocessing and segmentation method that has an acceptable and consistent performance on images acquired from various sources should finally be tested in clinical conditions. Therefore we strongly advocate close collaboration between researchers and authors of well-performing methods with clinicians. In such a scenario, the acceptability of the method's performance can be validated against the relevance for a given task. Moreover, an open dialogue between researchers and clinicians will help build an ABS system that meets the requirements for a clinically useful and usable tool.

Finally, efforts to define steps on the path towards designing and validating a clinically applicable ABS system should be made. We recommend consultation with stakeholders as a key element to verify the actual clinical needs and how to assess to what extent these needs are met by available research.

CONCLUSIONS

This scoping study of automatic brain lesion segmentation on magnetic resonance images shows a field growing at a rapid pace, an imbalance between proposed methods of which there are many and methods implemented for clinical application of which there are few, and room for improvement of reporting practice with a view to replication, method comparison and implementation. To promote this improvement, we propose a list of recommendations for future studies in the field. We identify knowledge gaps and potentially fruitful avenues for future research.

For peer review only

REFERENCES

1 Therasse P, Arbuck SG, Eisenhauer EA *et al.* New guidelines to evaluate the response to treatment in solid tumors. European Organization for Research and Treatment of Cancer, National Cancer Institute of the United States, National Cancer Institute of Canada. *J Natl Cancer Inst* 2000;**92**:205–16. doi:10.1093/jnci/92.3.205

2 Miller AB, Hoogstraten B, Staquet M *et al.* Reporting results of cancer treatment. *Cancer* 1981;**47**:207–14. doi:10.1002/1097-0142(19810101)47:1<207::aid-cncr2820470134>3.0.co;2-6

3 Fazekas F, Chawluk JB, Alavi A *et al.* MR signal abnormalities at 1.5 T in Alzheimer's dementia and normal aging. *AJR Am J Roentgenol* 1987;**149**:351–6. doi:10.2214/ajr.149.2.351

4 Ellingson BM, Wen PY, Cloughesy TF. Modified Criteria for Radiographic Response Assessment in Glioblastoma Clinical Trials. *Neurotherapeutics* 2017;**14**:307–20. doi:10.1007/s13311-016-0507-6

5 Rosenkrantz AB, Hanna TN, Babb JS *et al.* Changes in Emergency Department Imaging: Perspectives From National Patient Surveys Over Two Decades. *Journal of the American College of Radiology* 2017;**14**:1282–90. doi:10.1016/j.jacr.2017.03.004

6 Smith-Bindman R, Miglioretti DL, Larson EB. Rising Use Of Diagnostic Medical Imaging In A Large Integrated Health System. *Health Aff (Millwood)* 2008;**27**:1491–502. doi:10.1377/hlthaff.27.6.1491

7 Bauer S, Wiest R, Nolte L-P *et al.* A survey of MRI-based medical image analysis for brain tumor studies. *Phys Med Biol* 2013;**58**:R97–R129. doi:10.1088/0031-9155/58/13/R97

8 Debette S, Markus HS. The clinical importance of white matter hyperintensities on brain magnetic resonance imaging: Systematic review and meta-analysis. *BMJ* 2010;**341**:c3666. doi:10.1136/bmj.c3666

9 Deepa, Singh A. Review of brain tumor detection from MRI images. In: *2016 3rd International Conference on Computing for Sustainable Global Development (INDIACom)*. 2016. 3997–4000.

10 García-Lorenzo D, Francis S, Narayanan S *et al.* Review of automatic segmentation methods of multiple sclerosis white matter lesions on conventional magnetic resonance imaging. *Medical Image Analysis* 2013;**17**:1–18. doi:10.1016/j.media.2012.09.004

11 Işın A, Direkoğlu C, Şah M. Review of MRI-based Brain Tumor Image Segmentation Using Deep Learning Methods. *Procedia Computer Science* 2016;**102**:317–24. doi:10.1016/j.procs.2016.09.407

12 Ito KL, Kim H, Liew S-L. A comparison of automated lesion segmentation approaches for chronic stroke T1-weighted MRI data. *bioRxiv* Published Online First: November 2018. doi:10.1101/441451

13 Kumari N, Saxena S. Review of Brain Tumor Segmentation and Classification. In: *2018 International Conference on Current Trends towards Converging Technologies (ICCTCT)*. 2018. 1–6. doi:10.1109/ICCTCT.2018.8551004

14 Mortazavi D, Kouzani AZ, Soltanian-Zadeh H. Segmentation of multiple sclerosis lesions in MR images: A review. *Neuroradiology* 2012;**54**:299–320. doi:10.1007/s00234-011-0886-7

15 Roy S, Nag S, Maitra IK *et al.* A Review on Automated Brain Tumor Detection and Segmentation from MRI of Brain.;41.

16 Saritha S, Prabha NA. A comprehensive review: Segmentation of MRI images—brain tumor. *International Journal of Imaging Systems and Technology* 2016;**26**:295–304. doi:10.1002/ima.22201

17 Arksey H, O'Malley L. Scoping studies: Towards a methodological framework. *International Journal of Social Research Methodology* 2005;**8**:19–32. doi:10.1080/1364557032000119616

For peer review only

18 Levac D, Colquhoun H, O'Brien KK. Scoping studies: Advancing the methodology. *Implement Sci* 2010;**5**:69. doi:10.1186/1748-5908-5-69

19 Colquhoun HL, Levac D, O'Brien KK *et al.* Scoping reviews: Time for clarity in definition, methods, and reporting. *J Clin Epidemiol* 2014;**67**:1291–4. doi:10.1016/j.jclinepi.2014.03.013

20 Moher D, Liberati A, Tetzlaff J *et al.* Preferred Reporting Items for Systematic Reviews and Meta-Analyses: The PRISMA Statement. *PLOS Medicine* 2009;**6**:e1000097. doi:10.1371/journal.pmed.1000097

21 Tricco AC, Lillie E, Zarin W *et al.* PRISMA Extension for Scoping Reviews (PRISMA-ScR): Checklist and Explanation. *Ann Intern Med* 2018;**169**:467–73. doi:10.7326/M18-0850

22 Gryska EA, Schneiderman J, Heckemann RA. Automatic brain lesion segmentation on standard MRIs of the human head: A scoping review protocol. *BMJ Open* 2019;**9**:e024824. doi:10.1136/bmjopen-2018-024824

23 Styner M, Lee J, Chin B *et al.* 3D Segmentation in the Clinic: A Grand Challenge II: MS lesion segmentation. *The MIDAS Journal* 2008;638

24 Menze BH, Jakab A, Bauer S *et al.* The Multimodal Brain Tumor Image Segmentation Benchmark (BRATS). *IEEE Transactions on Medical Imaging* 2015;**34**:1993–2024. doi:10.1109/TMI.2014.2377694

25 Carass A, Roy S, Jog A *et al.* Longitudinal multiple sclerosis lesion segmentation: Resource and challenge. *Neuroimage* 2017;**148**:77–102. doi:10.1016/j.neuroimage.2016.12.064

26 Maier O, Menze BH, Gablentz J von der *et al.* ISLES 2015 - A public evaluation benchmark for ischemic stroke lesion segmentation from multispectral MRI. *Medical Image Analysis* 2017;**35**:250–69. doi:10.1016/j.media.2016.07.009

27 Sled JG, Zijdenbos AP, Evans AC. A nonparametric method for automatic correction of intensity nonuniformity in MRI data. *IEEE Trans Med Imaging* 1998;**17**:87–97. doi:10.1109/42.668698

28 Tustison NJ, Avants BB, Cook PA *et al.* N4ITK: Improved N3 Bias Correction. *IEEE Trans Med Imaging* 2010;**29**:1310–20. doi:10.1109/TMI.2010.2046908

29 Smith SM. Fast robust automated brain extraction. *Hum Brain Mapp* 2002;**17**:143–55. doi:10.1002/hbm.10062

30 Statistical Parametric Mapping: The Analysis of Functional Brain Images - 1st Edition. Available from: https://www.elsevier.com/books/statistical-parametric-mapping-the-analysis-of-functional-brain-images/penny/978-0-12-372560-8 (accessed 7 Nov 2019).

31 Smith SM, Jenkinson M, Woolrich MW *et al.* Advances in functional and structural MR image analysis and implementation as FSL. *NeuroImage* 2004;**23**:S208–19. doi:10.1016/j.neuroimage.2004.07.051

32 Roura E, Sarbu N, Oliver A *et al.* Automated Detection of Lupus White Matter Lesions in MRI. *Front Neuroinform* 2016;**10**. doi:10.3389/fninf.2016.00033

33 Griffis JC, Allendorfer JB, Szaflarski JP. Voxel-based Gaussian naïve Bayes classification of ischemic stroke lesions in individual T1-weighted MRI scans. *Journal of Neuroscience Methods* 2016;**257**:97–108. doi:10.1016/j.jneumeth.2015.09.019

34 Galimzianova A, Pernuš F, Likar B *et al.* Stratified mixture modeling for segmentation of white-matter lesions in brain MR images. *NeuroImage* 2016;**124**:1031–43. doi:10.1016/j.neuroimage.2015.09.047

35 Klöppel S, Abdulkadir A, Hadjidemetriou S *et al.* A comparison of different automated methods for the detection of white matter lesions in MRI data. *NeuroImage* 2011;**57**:416–22. doi:10.1016/j.neuroimage.2011.04.053

36 Herskovits E, Bryan R, Yang F. Automated Bayesian Segmentation of Microvascular White-Matter Lesions in the ACCORD-MIND Study. 2008;**53**. doi:10.2478/v10039-008-0039-3

For peer review only

37 Rexilius J, Hahn HK, Klein J *et al.* Multispectral brain tumor segmentation based on histogram model adaptation. In: *Medical Imaging 2007: Computer-Aided Diagnosis*. International Society for Optics; Photonics 2007. 65140V. doi:10.1117/12.709410

38 Datta S, Sajja BR, He R *et al.* Segmentation of gadolinium-enhanced lesions on MRI in multiple sclerosis. *Journal of Magnetic Resonance Imaging* 2007;**25**:932–7. doi:10.1002/jmri.20896

39 Le Folgoc L, Nori AV, Ancha S *et al.* Lifted Auto-Context Forests for Brain Tumour Segmentation. In: Crimi A, Menze B, Maier O, eds. *et al. Brainlesion: Glioma, Multiple Sclerosis, Stroke and Traumatic Brain Injuries*. Cham:: Springer International Publishing 2016. 171–83. doi:10.1007/978-3-319-55524-9_17

40 Maier O, Schröder C, Forkert ND *et al.* Classifiers for Ischemic Stroke Lesion Segmentation: A Comparison Study. *PLOS ONE* 2015;**10**:e0145118. doi:10.1371/journal.pone.0145118

41 Kellner-Weldon F, Stippich C, Wiest R *et al.* Comparison of perioperative automated versus manual two-dimensional tumor analysis in glioblastoma patients. *European Journal of Radiology* 2017;**95**:75–81. doi:10.1016/j.ejrad.2017.07.028

42 Maier O, Wilms M, Gablentz J von der *et al.* Extra Tree forests for sub-acute ischemic stroke lesion segmentation in MR sequences. *Journal of Neuroscience Methods* 2015;**240**:89–100. doi:10.1016/j.jneumeth.2014.11.011

43 Rincón M, Díaz-López E, Selnes P *et al.* Improved Automatic Segmentation of White Matter Hyperintensities in MRI Based on Multilevel Lesion Features. *Neuroinform* 2017;**15**:231–45. doi:10.1007/s12021-017-9328-y

44 Nie J, Xue Z, Liu T *et al.* Automated brain tumor segmentation using spatial accuracy-weighted hidden Markov Random Field. *Computerized Medical Imaging and Graphics* 2009;**33**:431–41. doi:10.1016/j.compmedimag.2009.04.006

45 Adler S, Wagstyl K, Gunny R *et al.* Novel surface features for automated detection of focal cortical dysplasias in paediatric epilepsy. *Neuroimage Clin* 2016;**14**:18–27. doi:10.1016/j.nicl.2016.12.030

46 Maldjian JA, Whitlow CT, Saha BN *et al.* Automated White Matter Total Lesion Volume Segmentation in Diabetes. *American Journal of Neuroradiology* 2013;**34**:2265–70. doi:10.3174/ajnr.A3590

47 Alegro M de C, Amaro Junior E, Lopes R de D. Computerized brain tumor segmentation in magnetic resonance imaging. *Einstein (São Paulo)* 2012;**10**:158–63. doi:10.1590/S1679-45082012000200008

48 Kamber M, Shinghal R, Collins DL *et al.* Model-based 3-D segmentation of multiple sclerosis lesions in magnetic resonance brain images. *IEEE Transactions on Medical Imaging* 1995;**14**:442–53. doi:10.1109/42.414608

49 AlBadawy EA, Saha A, Mazurowski MA. Deep learning for segmentation of brain tumors: Impact of cross-institutional training and testing. *Medical Physics* 2018;**45**:1150–8. doi:10.1002/mp.12752

50 Puonti O, Van Leemput K. Simultaneous Whole-Brain Segmentation and White Matter Lesion Detection Using Contrast-Adaptive Probabilistic Models. In: Crimi A, Menze B, Maier O, eds. *et al. Brainlesion: Glioma, Multiple Sclerosis, Stroke and Traumatic Brain Injuries*. Cham:: Springer International Publishing 2016. 9–20. doi:10.1007/978-3-319-30858-6_2

51 Kaur T, Saini BS, Gupta S. A novel fully automatic multilevel thresholding technique based on optimized intuitionistic fuzzy sets and tsallis entropy for MR brain tumor image segmentation. *Australas Phys Eng Sci Med* 2018;**41**:41–58. doi:10.1007/s13246-017-0609-4

52 Dvořák P, Bartušek K, Smékal Z. Unsupervised Pathological Area Extraction using 3D T2 and FLAIR MR Images. *Measurement Science Review* 2014;**14**:357–64. doi:10.2478/msr-2014-0049

For peer review only

53 Kaur T, Saini BS, Gupta S. A joint intensity and edge magnitude-based multilevel thresholding algorithm for the automatic segmentation of pathological MR brain images. *Neural Comput & Applic* 2018;**30**:1317–40. doi:10.1007/s00521-016-2751-4

54 Sweeney EM, Vogelstein JT, Cuzzocreo JL *et al.* A Comparison of Supervised Machine Learning Algorithms and Feature Vectors for MS Lesion Segmentation Using Multimodal Structural MRI. *PLoS ONE* 2014;**9**:e95753. doi:10.1371/journal.pone.0095753

55 Admiraal-Behloul F, Heuvel DMJ van den, Olofsen H *et al.* Fully automatic segmentation of white matter hyperintensities in MR images of the elderly. *NeuroImage* 2005;**28**:607–17. doi:10.1016/j.neuroimage.2005.06.061

56 Jain S, Ribbens A, Sima DM *et al.* Unsupervised Framework for Consistent Longitudinal MS Lesion Segmentation. In: Müller H, Kelm BM, Arbel T, eds. *et al. Medical Computer Vision and Bayesian and Graphical Models for Biomedical Imaging*. Springer International Publishing 2017. 208–19.

57 Jain S, Sima DM, Ribbens A *et al.* Automatic segmentation and volumetry of multiple sclerosis brain lesions from MR images. *NeuroImage: Clinical* 2015;**8**:367–75. doi:10.1016/j.nicl.2015.05.003

58 Elliott C, Francis SJ, Arnold DL *et al.* Bayesian Classification of Multiple Sclerosis Lesions in Longitudinal MRI Using Subtraction Images. In: Jiang T, Navab N, Pluim JPW, eds. *et al. Medical Image Computing and Computer-Assisted Intervention – MICCAI 2010*. Springer Berlin Heidelberg 2010. 290–7.

59 Griffanti L, Zamboni G, Khan A *et al.* BIANCA (brain intensity abnormality classification algorithm): A new tool for automated segmentation of white matter hyperintensities. *NeuroImage* 2016;**141**:191–205. doi:10.1016/j.neuroimage.2016.07.018

60 Akselrod-Ballin A, Galun M, Gomori JM *et al.* Automatic Segmentation and Classification of Multiple Sclerosis in Multichannel MRI. *IEEE Transactions on Biomedical Engineering* 2009;**56**:2461–9. doi:10.1109/TBME.2008.926671

61 Weizman L, Ben Sira L, Joskowicz L *et al.* Automatic segmentation, internal classification, and follow-up of optic pathway gliomas in MRI. *Medical Image Analysis* 2012;**16**:177–88. doi:10.1016/j.media.2011.07.001

62 Zacharaki EI, Kanterakis S, Bryan RN *et al.* Measuring Brain Lesion Progression with a Supervised Tissue Classification System. In: Metaxas D, Axel L, Fichtinger G, eds. *et al. Medical Image Computing and Computer-Assisted Intervention – MICCAI 2008*. Springer Berlin Heidelberg 2008. 620–7.

63 Rachmadi MF, Valdés-Hernández M del C, Agan MLF *et al.* Segmentation of white matter hyperintensities using convolutional neural networks with global spatial information in routine clinical brain MRI with none or mild vascular pathology. *Computerized Medical Imaging and Graphics* 2018;**66**:28–43. doi:10.1016/j.compmedimag.2018.02.002

64 Lesjak Ž, Pernuš F, Likar B *et al.* Validation of White-Matter Lesion Change Detection Methods on a Novel Publicly Available MRI Image Database. *Neuroinform* 2016;**14**:403–20. doi:10.1007/s12021-016-9301-1

65 Sitter A de, Steenwijk MD, Ruet A *et al.* Performance of five research-domain automated WM lesion segmentation methods in a multi-center MS study. *NeuroImage* 2017;**163**:106–14. doi:10.1016/j.neuroimage.2017.09.011

66 Shiee N, Bazin P-L, Ozturk A *et al.* A topology-preserving approach to the segmentation of brain images with multiple sclerosis lesions. *NeuroImage* 2010;**49**:1524–35. doi:10.1016/j.neuroimage.2009.09.005

67 Ling Y, Jouvent E, Cousyn L *et al.* Validation and Optimization of BIANCA for the Segmentation of Extensive White Matter Hyperintensities. *Neuroinformatics* 2018;**16**:269–81. doi:10.1007/s12021-018-9372-2

68 Wang R, Li C, Wang J *et al.* Automatic Segmentation and Quantitative Analysis of White Matter Hyperintensities on FLAIR Images Using Trimmed-Likelihood Estimator. *Academic Radiology* 2014;**21**:1512–23. doi:10.1016/j.acra.2014.07.001

For peer review only

69 Mah Y-H, Jager R, Kennard C *et al.* A new method for automated high-dimensional lesion segmentation evaluated in vascular injury and applied to the human occipital lobe. *Cortex* 2014;**56**:51–63. doi:10.1016/j.cortex.2012.12.008

70 Maji P, Roy S. SoBT-RFW: Rough-Fuzzy Computing and Wavelet Analysis Based Automatic Brain Tumor Detection Method from MR Images. *Fundamenta Informaticae* 2015;**142**:237–67. doi:10.3233/FI-2015-1293

71 Roura E, Oliver A, Cabezas M *et al.* A toolbox for multiple sclerosis lesion segmentation. *Neuroradiology* 2015;**57**:1031–43. doi:10.1007/s00234-015-1552-2

72 Rios Velazquez E, Meier R, Dunn Jr WD *et al.* Fully automatic GBM segmentation in the TCGA-GBM dataset: Prognosis and correlation with VASARI features. *Scientific Reports* 2015;**5**:16822. doi:10.1038/srep16822

73 Stone JR, Wilde EA, Taylor BA *et al.* Supervised learning technique for the automated identification of white matter hyperintensities in traumatic brain injury. *Brain Injury* 2016;**30**:1458–68. doi:10.1080/02699052.2016.1222080

74 Meier R, Knecht U, Loosli T *et al.* Clinical Evaluation of a Fully-automatic Segmentation Method for Longitudinal Brain Tumor Volumetry. *Scientific Reports* 2016;**6**:23376. doi:10.1038/srep23376

75 Hansen MB, Nagenthiraja K, Ribe LR *et al.* Automated estimation of salvageable tissue: Comparison with expert readers. *Journal of Magnetic Resonance Imaging* 2016;**43**:220–8. doi:10.1002/jmri.24963

76 Goetz M, Weber C, Binczyk F *et al.* DALSA: Domain Adaptation for Supervised Learning From Sparsely Annotated MR Images. *IEEE Transactions on Medical Imaging* 2016;**35**:184–96. doi:10.1109/TMI.2015.2463078

77 Valverde S, Cabezas M, Roura E *et al.* Improving automated multiple sclerosis lesion segmentation with a cascaded 3D convolutional neural network approach. *NeuroImage* 2017;**155**:159–68. doi:10.1016/j.neuroimage.2017.04.034

78 Kamnitsas K, Ledig C, Newcombe VFJ *et al.* Efficient multi-scale 3D CNN with fully connected CRF for accurate brain lesion segmentation. *Medical Image Analysis* 2017;**36**:61–78. doi:10.1016/j.media.2016.10.004

79 Damangir S, Westman E, Simmons A *et al.* Reproducible segmentation of white matter hyperintensities using a new statistical definition. *Magn Reson Mater Phy* 2017;**30**:227–37. doi:10.1007/s10334-016-0599-3

80 Dadar M, Maranzano J, Misquitta K *et al.* Performance comparison of 10 different classification techniques in segmenting white matter hyperintensities in aging. *NeuroImage* 2017;**157**:233–49. doi:10.1016/j.neuroimage.2017.06.009

81 Knight J, Taylor GW, Khademi A. Voxel-Wise Logistic Regression and Leave-One-Source-Out Cross Validation for white matter hyperintensity segmentation. *Magnetic Resonance Imaging* 2018;**54**:119–36. doi:10.1016/j.mri.2018.06.009

82 Salem M, Cabezas M, Valverde S *et al.* A supervised framework with intensity subtraction and deformation field features for the detection of new T2-w lesions in multiple sclerosis. *NeuroImage: Clinical* 2018;**17**:607–15. doi:10.1016/j.nicl.2017.11.015

83 Li H, Jiang G, Zhang J *et al.* Fully convolutional network ensembles for white matter hyperintensities segmentation in MR images. *NeuroImage* 2018;**183**:650–65. doi:10.1016/j.neuroimage.2018.07.005

84 Jiang J, Liu T, Zhu W *et al.* UBO Detector – A cluster-based, fully automated pipeline for extracting white matter hyperintensities. *NeuroImage* 2018;**174**:539–49. doi:10.1016/j.neuroimage.2018.03.050

85 Tustison NJ, Shrinidhi KL, Wintermark M *et al.* Optimal Symmetric Multimodal Templates and Concatenated Random Forests for Supervised Brain Tumor Segmentation (Simplified) with ANTsR. *Neuroinform* 2015;**13**:209–25. doi:10.1007/s12021-014-9245-2

For peer review only

86 Souplet J-C, Lebrun C, Ayache N *et al.* An Automatic Segmentation of T2-FLAIR Multiple Sclerosis Lesions.;12.

87 Ithapu V, Singh V, Lindner C *et al.* Extracting and summarizing white matter hyperintensities using supervised segmentation methods in Alzheimer's disease risk and aging studies. *Human Brain Mapping* 2014;**35**:4219–35. doi:10.1002/hbm.22472

88 Ozenne B, Subtil F, Østergaard L *et al.* Spatially regularized mixture model for lesion segmentation with application to stroke patients. *Biostatistics* 2015;**16**:580–95. doi:10.1093/biostatistics/kxv004

89 Wang R, Li C, Wang J *et al.* Automatic segmentation and volumetric quantification of white matter hyperintensities on fluid-attenuated inversion recovery images using the extreme value distribution. *Neuroradiology* 2015;**57**:307–20. doi:10.1007/s00234-014-1466-4

90 Guizard N, Coupé P, Fonov VS *et al.* Rotation-invariant multi-contrast non-local means for MS lesion segmentation. *NeuroImage: Clinical* 2015;**8**:376–89. doi:10.1016/j.nicl.2015.05.001

91 Bartko JJ. Measurement and Reliability: Statistical Thinking Considerations. *Schizophr Bull* 1991;**17**:483–9. doi:10.1093/schbul/17.3.483

92 Recent Advances in MRI Technology. Imaging Technology News. 2016. Available from: https://www.itnonline.com/article/recent-advances-mri-technology (accessed 5 May 2020).

93 OECD Statistics, health care utilisation : Diagnostic exams. Available from: https://stats.oecd.org/Index.aspx?ThemeTreeId=9 (accessed 5 May 2020).

94 FDA Clears First 7T MRI System, Magnetom Terra. Imaging Technology News. 2017. Available from: https://www.itnonline.com/content/fda-clears-first-7t-mri-system-magnetom-terra (accessed 5 May 2020).

95 Visser M, Müller D, Duijn R van *et al.* Inter-rater agreement in glioma segmentations on longitudinal MRI. *NeuroImage: Clinical* 2019;**22**:101727. doi:10.1016/j.nicl.2019.101727

96 Bø HK, Solheim O, Jakola AS *et al.* Intra-rater variability in low-grade glioma segmentation. *J Neurooncol* 2017;**131**:393–402. doi:10.1007/s11060-016-2312-9

97 Zijdenbos AP, Dawant BM, Margolin RA *et al.* Morphometric analysis of white matter lesions in MR images: Method and validation. *IEEE Transactions on Medical Imaging* 1994;**13**:716–24. doi:10.1109/42.363096

98 Ashton EA, Takahashi C, Berg MJ *et al.* Accuracy and reproducibility of manual and semiautomated quantification of MS lesions by MRI. *Journal of Magnetic Resonance Imaging* 2003;**17**:300–8. doi:10.1002/jmri.10258

99 Zijdenbos AP, Forghani R, Evans AC. Automatic "pipeline" analysis of 3-D MRI data for clinical trials: Application to multiple sclerosis. *IEEE Transactions on Medical Imaging* 2002;**21**:1280–91. doi:10.1109/TMI.2002.806283

100 Grimaud J, Lai M, Thorpe J *et al.* Quantification of MRI lesion load in multiple sclerosis: A comparison of three computer-assisted techniques. *Magnetic Resonance Imaging* 1996;**14**:495–505. doi:10.1016/0730-725X(96)00018-5

101 Gibson E, Hu Y, Huisman HJ *et al.* Designing image segmentation studies: Statistical power, sample size and reference standard quality. *Med Image Anal* 2017;**42**:44–59. doi:10.1016/j.media.2017.07.004

102 Yoo TS, Ackerman MJ, Lorensen WE *et al.* Engineering and algorithm design for an image processing api: A technical report on itk-the insight toolkit. *Studies in health technology and informatics* 2002;586–92.

For peer review only

103 Kuijf HJ, Biesbroek JM, Bresser J de *et al.* Standardized Assessment of Automatic Segmentation of White Matter Hyperintensities; Results of the WMH Segmentation Challenge. *IEEE Transactions on Medical Imaging* 2019;1–1. doi:10.1109/TMI.2019.2905770

104 Klein S, Staring M, Murphy K *et al.* Elastix: A toolbox for intensity-based medical image registration. *IEEE transactions on medical imaging* 2009;**29**:196–205.

105 Shamonin DP, Bron EE, Lelieveldt BP *et al.* Fast parallel image registration on cpu and gpu for diagnostic classification of alzheimer's disease. *Frontiers in neuroinformatics* 2014;**7**:50.

106 Fonov V, Evans AC, Botteron K *et al.* Unbiased average age-appropriate atlases for pediatric studies. *Neuroimage* 2011;**54**:313–27.

107 Fonov VS, Evans AC, McKinstry RC *et al.* Unbiased nonlinear average age-appropriate brain templates from birth to adulthood. *NeuroImage* 2009;S102.

108 Jenkinson M, Pechaud M, Smith S *et al.* BET2: MR-based estimation of brain, skull and scalp surfaces. In: *Eleventh annual meeting of the organization for human brain mapping*. Toronto. 2005. 167.

109 Maier-Hein L, Eisenmann M, Reinke A *et al.* Why rankings of biomedical image analysis competitions should be interpreted with care. *Nat Commun* 2018;**9**:1–13. doi:10.1038/s41467-018-07619-7

110 Jorritsma W, Cnossen F, Ooijen PMA van. Improving the radiologist–CAD interaction: Designing for appropriate trust. *Clinical Radiology* 2015;**70**:115–22. doi:10.1016/j.crad.2014.09.017

111 Gryska E, Cerna K, Heckemann RA. Increasing trust through the design of algorithm-based lesion segmentation support systems. iConference 2020 Proceedings. 2020 Mar 23.

FIGURE LIST AND CAPTIONS

Figure 1: *Flowchart of the article selection process from the result of querying three databases. For each stage, the number of articles selected is shown.*

Figure 2: *Distribution of papers describing the segmentation method of a particular lesion type (MS - multiple sclerosis, WML - white matter lesions).*

Figure 3: *Distribution of the most common algorithms and models implemented in the proposed lesion segmentation methods (ANN - artificial neural networks, DT - decision trees, RF - random fields, THR - thresholding, FC - fuzzy clustering, MM - mixture models, SVM - support vector machines, EM - expectation maximization).*

Figure 4: *The* number *of publications per year in the sample of eligible papers with lesion type stratification. We fit a linear function to the number of papers published each year from 2012 to estimate the development of the field in the upcoming years.*

Figure 5: *The* number *of patients (n) used to validate the methods.*

For peer review only

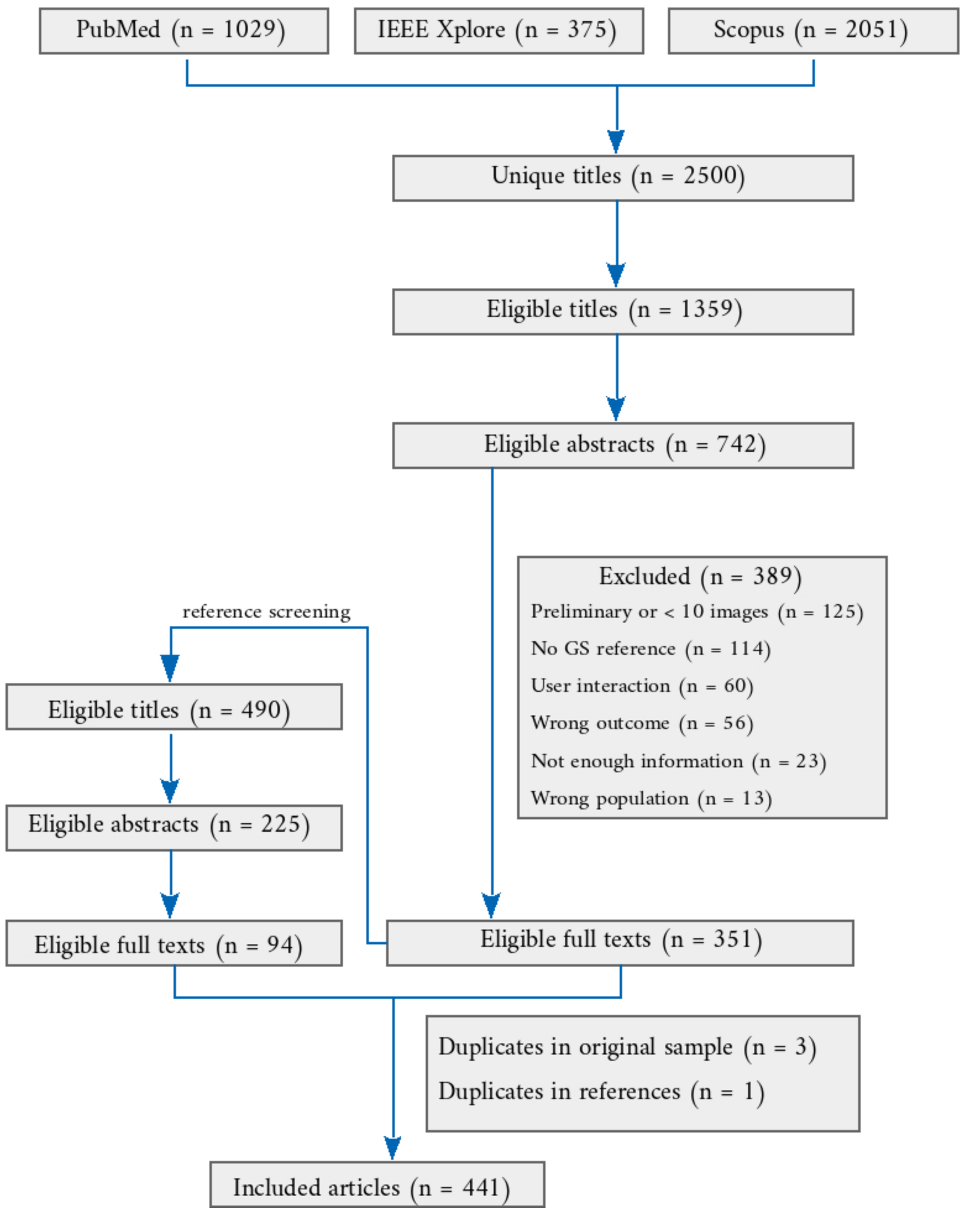

Flowchart of the article selection process from the result of querying three databases. For each stage, the number of articles selected is shown.

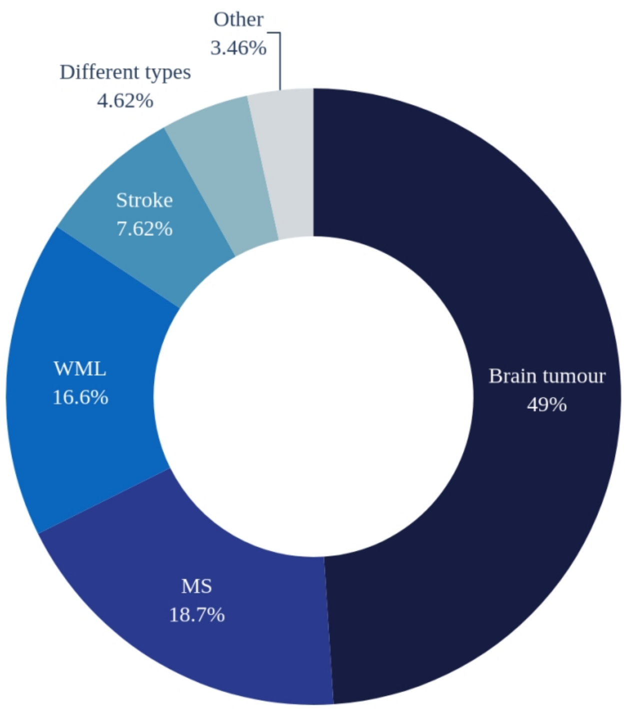

Distribution of papers describing the segmentation method of a particular lesion type (MS - multiple sclerosis, WML - white matter lesions).

481x378mm (72 x 72 DPI)

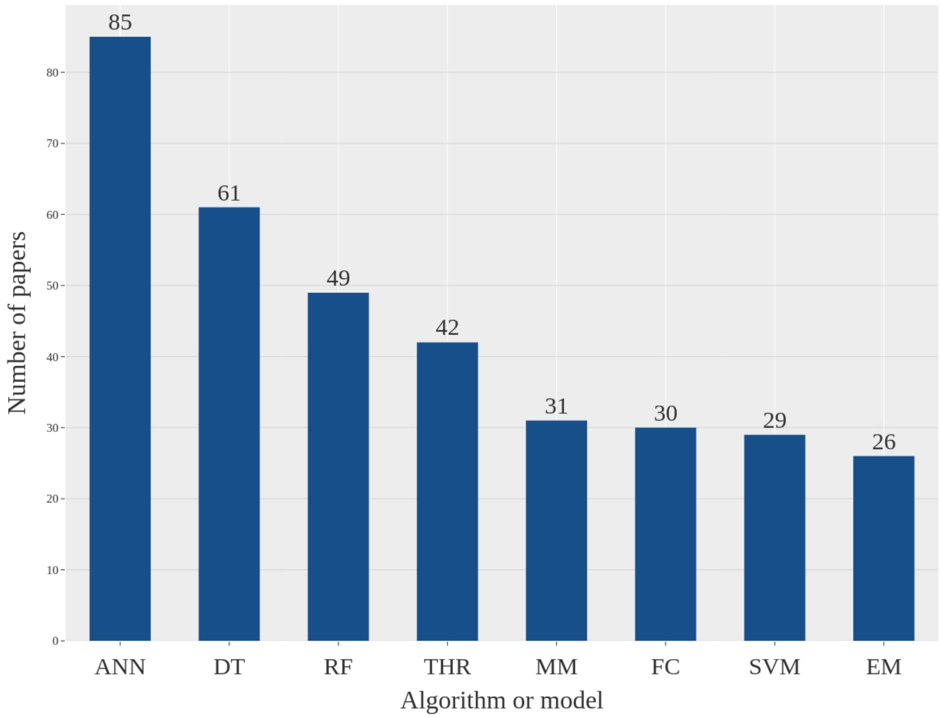

Distribution of the most common algorithms and models implemented in the proposed lesion segmentation methods (ANN - artificial neural networks, DT - decision trees, RF - random fields, THR - thresholding, FC - fuzzy clustering, MM - mixture models, SVM - support vector machines, EM - expectation maximization).

443x349mm (72 x 72 DPI)

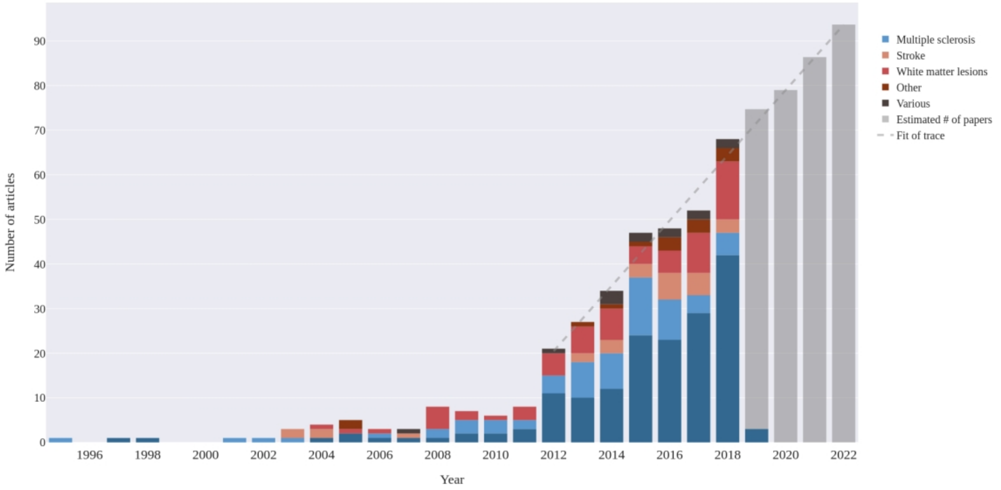

The number of publications per year in the sample of eligible papers with lesion type stratification. We fit a linear function to the number of papers published each year from 2012 to estimate the development of the field in the upcoming years.

640x345mm (72 x 72 DPI)

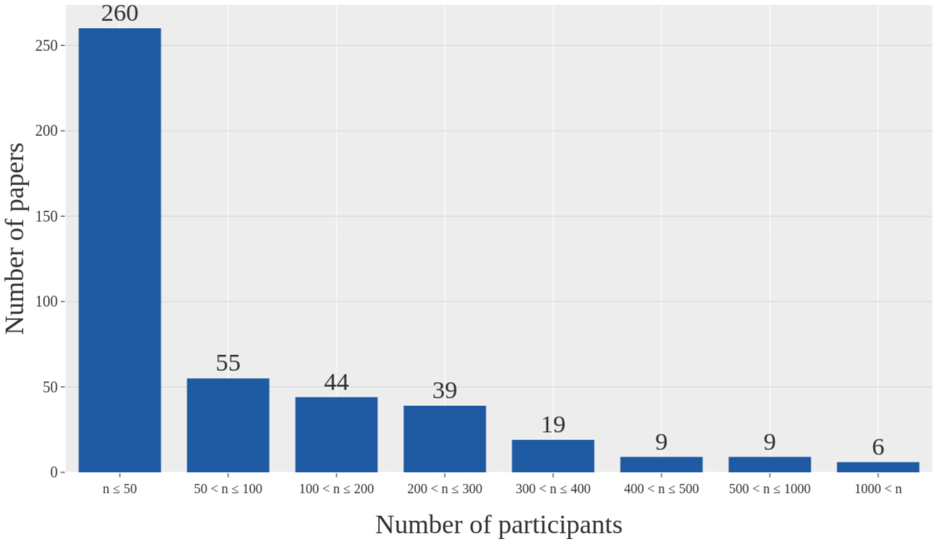

The number of patients (n) used to validate the methods.

423x282mm (72 x 72 DPI)

BMJ Open

Automatic brain lesion segmentation on standard magnetic resonance images: a scoping review

Journal:	*BMJ Open*
Manuscript ID	bmjopen-2020-042660.R1
Article Type:	Original research
Date Submitted by the Author:	24-Nov-2020
Complete List of Authors:	Gryska, Emilia; Goteborgs universitet Institutionen for kliniska vetenskaper, Radiation Physics<br>Schneiderman, Justin; Goteborgs universitet Institutionen for neurovetenskap och fysiologi, Sektionen för klinisk neurovetenskap<br>Björkman-Burtscher, Isabella; Göteborgs universitet Institutionen för kliniska vetenskaper, Radiology<br>Heckemann, Rolf; Goteborgs universitet Institutionen for kliniska vetenskaper, Radiation Physics
**Primary Subject Heading**:	Radiology and imaging
Secondary Subject Heading:	Research methods
Keywords:	RADIOLOGY & IMAGING, Magnetic resonance imaging < RADIOLOGY & IMAGING, Diagnostic radiology < RADIOLOGY & IMAGING, Neuroradiology < RADIOLOGY & IMAGING

SCHOLARONE™
Manuscripts

BMJ

I, the Submitting Author has the right to grant and does grant on behalf of all authors of the Work (as defined in the below author licence), an exclusive licence and/or a non-exclusive licence for contributions from authors who are: i) UK Crown employees; ii) where BMJ has agreed a CC-BY licence shall apply, and/or iii) in accordance with the terms applicable for US Federal Government officers or employees acting as part of their official duties; on a worldwide, perpetual, irrevocable, royalty-free basis to BMJ Publishing Group Ltd ("BMJ") its licensees and where the relevant Journal is co-owned by BMJ to the co-owners of the Journal, to publish the Work in this journal and any other BMJ products and to exploit all rights, as set out in our licence.

The Submitting Author accepts and understands that any supply made under these terms is made by BMJ to the Submitting Author unless you are acting as an employee on behalf of your employer or a postgraduate student of an affiliated institution which is paying any applicable article publishing charge ("APC") for Open Access articles. Where the Submitting Author wishes to make the Work available on an Open Access basis (and intends to pay the relevant APC), the terms of reuse of such Open Access shall be governed by a Creative Commons licence – details of these licences and which Creative Commons licence will apply to this Work are set out in our licence referred to above.

Other than as permitted in any relevant BMJ Author's Self Archiving Policies, I confirm this Work has not been accepted for publication elsewhere, is not being considered for publication elsewhere and does not duplicate material already published. I confirm all authors consent to publication of this Work and authorise the granting of this licence.

For peer review only

Automatic brain lesion segmentation on standard magnetic resonance images: a scoping review

Emilia Gryska[1,2*], Justin F. Schneiderman[1,3], Isabella Björkman-Burtscher[4], Rolf A. Heckemann[1,2]

1 MedTech West at Sahlgrenska University Hospital, University of Gothenburg, Sweden

2 Department of Radiation Physics, Institute of Clinical Sciences, University of Gothenburg, Sweden

3 Department of Clinical Neurophysiology, Institute of Neuroscience and Physiology, Sahlgrenska Academy, University of Gothenburg, Sweden

4 Department of Radiology, Institute of Clinical Sciences, Sahlgrenska Academy, University of Gothenburg and Sahlgrenska University Hospital, Gothenburg, Sweden

* Corresponding author: Emilia Gryska

Postal address: MedTech West, Röda stråket 10B, Sahlgrenska University Hospital, 413 45 Göteborg

E-mail address: emilia.gryska@medtechwest.se

Telephone number: +46 720304106

Keywords: Algorithms, Research design, Reproducibility of Results, Image Processing, Computer-Assisted

Word count: 6 302

Author contributions: EG conducted the study and led writing of the article. RAH was the main supervisor and consultant of the study progress and design choices. JFS provided input on the study plan and methodology at all stages. IBB provided guidance on the interpretation of the findings from a clinical perspective. All co-authors collaborated on manuscript composition and editing.

Funding: JFS is supported by the Swedish Childhood Cancer Foundation (MT2018-0020) and the ATTRACT project funded by the EC under Grant agreement 777222.

Competing interests: None declared

Supplementary data: The data extracted for the study can be found in FigShare repository, DOI: 10.6084/m9.figshare.12639842

ABSTRACT

Objectives Medical image analysis practices face challenges that can potentially be addressed with algorithm-based segmentation tools. In this study, we map the field of automatic MR brain lesion segmentation to understand the clinical applicability of prevalent methods and study designs, as well as challenges and limitations in the field.

Design Scoping review.

Setting Three databases (PubMed, IEEE Xplore, and Scopus) were searched with tailored queries. Studies were included based on predefined criteria. Emerging themes during consecutive title, abstract, methods, and whole-text screening were identified. The full-text analysis focused on materials, preprocessing, performance evaluation and comparison.

Results Out of 2990 unique articles identified through the search, 441 articles met the eligibility criteria, with an estimated growth rate of 10% per year. We present a general overview and trends in the field with regard to publication sources, segmentation principles used, and types of lesions. Algorithms are predominantly evaluated by measuring the agreement of segmentation results with a trusted reference. Few articles describe measures of clinical validity.

Conclusions The observed reporting practices leave room for improvement with a view to studying replication, method comparison, and clinical applicability. To promote this improvement, we propose a list of recommendations for future studies in the field.

ARTICLE SUMMARY

Strengths and limitations of this study

- This is the first overarching review of MR-based automatic brain lesion segmentation methods without restriction to a particular lesion type.
- The study was conducted following a previously published protocol.
- We consulted practitioners to ensure relevance of our work for clinical practice.
- The rigorous study design restricted our ability to expand on emerging themes, but did not entirely prevent epistemic drift.
- The restriction to a one-pass full-text analysis entailed a superficial level of interpretation – in hindsight a deeper reading would have been desirable.

INTRODUCTION

Magnetic resonance imaging (MRI) has become an integral part of diagnostics to detect, differentiate, and characterize brain lesions. Properties that determine this success are safety, high tissue contrast, and sensitivity to abnormality. Conditions that can lead to brain lesions include traumatic brain injury, vascular disease (including ischemic and hemorrhagic stroke), neoplasms, autoimmune disorders, infection, degenerative diseases, congenital conditions, and systemic diseases with secondary effects on the central nervous system. In some of these conditions, accurate estimation of lesion size and its progression may be essential to treatment planning, disease monitoring, and/or treatment evaluation.

Visual image interpretation is still the most common and accepted way to analyse clinical images. In some cases, the visual examination must be extended to include lesion delineation. For example, the boundaries of a tumour and structures at risk need to be localized accurately for radiation therapy planning. Such delineation depends on a skilled rater, is time-consuming, and is characterized by high variability between raters and high variability between repeated delineations on the same images by the same rater (i.e., low objectivity and reliability, respectively). In other clinical examinations, surrogate metrics, which can be based on the largest perpendicular diameters [1,2] or

For peer review only

visual scoring [3], are used for estimating the size of a lesion rapidly. Such visual or area-based lesion volume estimates may not be accurate enough [4].

Developments in magnetic resonance (MR) image acquisition procedures resulted in high-quality and complex images that capture a variety of structural and functional phenomena. On the one hand, the images carry more information about the lesions, thereby enabling more accurate diagnosis. On the other hand, each image requires more analysis time. This reduces the rate at which radiologists can analyse images. The increasing accessibility to MR scanners furthermore results in more images being acquired, further straining image processing capacity and potentially diminishing the quality of interpretation [5,6].

To summarize, three major issues with current clinical practices for image analysis are: subjective and time-consuming manual segmentation, limited accuracy of the surrogate volume estimates, and increasing information content and number of images to process.

Automatic lesion segmentation algorithms promise to alleviate these issues through fast and consistent lesion delineation that scales with demand. Quantitative image analysis using automatic lesion segmentation further has the potential to increase diagnostic and prognostic accuracy of lesion examination by providing radiologists with prompt and explicit information about a lesion.

Several reviews on automated brain lesion segmentation methods have previously been published. Most of these outline and analyse commonly used segmentation algorithms or general segmentation principles [7–16]. The perspective of the clinical applicability of automated segmentation algorithms has been explored by Garcia-Lorenzo et al. [10] and Bauer et al. [7]. In both reviews, the authors recommend improvements concerning study designs and formulation of research questions. The authors stress the importance of validating the robustness of the segmentation algorithms under variation stemming from three causes: differences between scanners and acquisition protocols, natural variability in normal anatomy and lesion appearance, and artifacts. Moreover, Bauer et al. [7] expressed the need for better communication between researchers developing segmentation methods and clinical radiologists who are the intended users of these methods.

A comprehensive review of automatic segmentation methods without restriction to lesion type is still lacking today, perhaps because of the challenge associated with the large and growing literature. We therefore present a scoping review of such methods and, given the rapidly growing extent of the literature, provide a timely account of the field that may not be feasible in the future. To ensure rigour, reproducibility, and comprehensiveness, we adopted scoping review methods proposed earlier [17–21].

Furthermore, there is a disconnection between developers and users of automatic brain lesion segmentation methods [7]. We therefore aim to examine clinical relevance of the research conducted in the field and seek to understand how the most prevalent methods (segmentation algorithms) and study designs (how they are validated) reflect the clinical applicability of methods described in the reviewed articles. Moreover, we aim to identify issues, limitations, and grand challenges of the field, and suggest actions to bridge the gap between research and clinical practice.

METHODS AND DESIGN

This study has been conducted according to a previously published protocol [22] and based on scoping review methods proposed by Arksey and O'Malley [17] and further developed by Levac et al. and Colquhoun et al. [18,19]. We also incorporate relevant parts of the PRISMA [20] and PRISMA-ScR [21] protocol for this type of review. Here we provide a summary of the protocol as well as an account of changes made, along with their respective rationale.

Stage 1: identifying research questions

The following research questions (RQs) were posed in the protocol [22]:

1 Which common image processing steps are necessary for automatic brain lesion segmentation on MR images?

2 Which mathematical and computational theories are most commonly applied in which types of brain lesions?

3 What is the efficacy of existing implementations?

4 What are the limitations of those methods and issues that should be addressed in future studies to develop a tool that is suitable for clinical use?

5 What are the most commonly used MR data sets that provide reference lesion segmentation and/or diagnostic classification?

While getting familiar with the abstracts of the sample we questioned the relevance of RQ 1 and 2. We recognised the need for consultation at this early stage of the project as opposed to consultation on the findings as originally proposed by Arksey and O'Malley [17]. The RQs do not reflect fully the potential of our study to address issues critical to advancing the field and bringing a benefit to the community. The consultation was conducted as semi-structured interviews with five clinicians who have experience in brain image analysis. We interviewed two neurosurgeons, an oncologist, a radiation oncologist, and a neuroradiologist. The interviews were structured to elicit understanding of their daily workflow, of how they use and analyse images, and of their opinions on automating the process of brain lesion segmentation.The initial screening of the sample abstracts and, in parallel, the consultations directed our analysis towards examining the clinical relevance of the prevalent methods and study designs.

Stage 2: identifying relevant articles

For the purpose of this study, we define an article as an individual published item that was found according to the presented search strategy and that meets the inclusion criteria of this review.

Eligibility criteria were unchanged from those defined in the protocol: articles evaluating an automatic brain lesion segmentation method applied to MR images acquired for human brain lesion inspection, and articles evaluating the performance of the method by comparison with a reference segmentation were suitable for the study.

The initial search strategy remained mostly unchanged. Three databases (PubMed, IEEE Xplore, and Scopus) were queried. Initially, a broad search phrase on automatic brain lesion segmentation methods on MRI was used to generate a first sample for each database. Controlled vocabulary tags were extracted from the articles and arranged in descending order of frequency. From this list, tags were selected to refine and customize the search phrase for each database. These refined and customized search phrases were used to retrieve the sample for our study. The search was conducted on 4th of November 2018.

The relevant articles were identified through hierarchical screening at four levels with given aims and objectives:

1) Title level: exclusion of clearly ineligible articles.

2) Abstract level: refinement of inclusion and exclusion criteria based on literature content; identification of 100 eligible articles by reading of randomly selected abstracts out of a sample of 1359; identification of themes to guide the study selection process (Stage 3);

3) Methods level: identification of two core concepts: algorithm validation and applicability of an automatic segmentation method for clinical parameter prediction; exclusion of studies using manual or semi-automatic segmentation, not stating the source of the tested images/patients, only using synthetic images, conducing the algorithm validation on fewer than 10 unique scans/images, or not providing results of the algorithm's performance.

4) full paper screening level (see Stage 3).

The same hierarchical screening procedure was applied to references of the eligible sample also screening for duplicates and excluding conference proceedings that were later published in journal articles if the patient cohort was similar in both articles. Screening was discontinued upon finding an exclusion criterion.

Stage 3: study selection

In the full-text screening, we applied the above criteria again and excluded papers that:

- did not perform segmentation or did not provide segmentation evaluation outcome measures (wrong outcome)

- proposed methods that require user interaction
- did not provide information about the gold standard (GS) or a reference measurement
- did not provide information about the origin of the images or used only synthetic images
- did not provide the number of images used for evaluation
- evaluated the method on fewer than 10 unique scans.

Stage 4: data charting

The data extracted were charted in a spreadsheet (supplementary material) with five categories of variables:

1) bibliographic category (first authors' name, article title, journal or conference name, and year of publication),

2) segmentation category (preprocessing procedures, algorithms and computational theories, if lesion classification used, required or allowed input modalities, processing time of the method, and availability of the software),

3) validation category (process of obtaining reference segmentations, number of raters or observers, and evaluation metrics),

4) study cohort category comprised patients' diagnoses, lesion type, sources, and number of images),

5) general comments category (information that did not fit in other categories, but was considered relevant) Stage 5: collating, summarizing, and reporting the data

Stage 5: collating, summarizing, and reporting the data

The collected data is disseminated through numerical analysis of bibliographic information overview, trends in the field, and study design characteristics. We provide the account of both prevalent study design choices and less common ones that are relevant to the clinical applicability assessment. Study design features are grouped into materials, preprocessing, performance evaluation, and performance comparison.

Patient and Public Involvement

No patients nor members of the public were involved in this research project.

RESULTS

The results of the scoping review include answers to research questions 2, 4, and 5. The answers to questions 1 and 5 are directly presented in the results. The answer to question 4 is formulated in the discussion based on the presented findings. In the discussion, we also justify why questions 1 ans 3 could not be explicitly answered.

Overview

The number of papers included at each stage of the review is shown in Figure 1. Among 2500 articles returned by the searches and 490 titles identified through reference screening, we included 441 articles in the review. Among the eligible papers, 255/441 were published in journals [23–277] and 184/441 in conference proceedings or conference workshops [278–460], and 2/441 articles were uploaded as preprints [461,462]. The following journals or conferences occurred most frequently as the publication source: International Conference on Medical Image Computing and Computer-Assisted Intervention (66 articles), NeuroImage (20 articles), IEEE Transactions on Medical Imaging (20 articles), SPIE Medical Imaging Conference (18 articles), NeuroImage: Clinical (15 articles), IEEE International Symposium on Biomedical Imaging (13 articles), and PloS One (12 articles).

Figure 2 shows the distribution of articles that propose a method to segment a particular brain lesion type. The dominance of methods segmenting brain tumours is evident from the data.

For peer review only - http://bmjopen.bmj.com/site/about/guidelines.xhtml

Trends

Substantial growth of the field was evident from the number of articles published annually (Figure 3). Three distinct periods with an increase in the number of published articles compared to preceding years were noticeable. During these years, an increase is also evident in the number of articles addressing segmentation of lesion types corresponding to the challenges taking place. The first wave corresponds to the *3D Segmentation in the Clinic: A Grand Challenge II: MS Lesion Segmentation* [463]. The second wave started in 2012 coinciding with the advent of *The Multimodal Brain Tumor Image Segmentation Benchmark (BraTS)* [464]. The third wave started in 2015 when three brain lesion segmentation challenges took place: BraTS, the *Longitudinal MS Lesion Segmentation Challenge* [465], and the *Ischemic Stroke Lesion Segmentation challenge (ISLES)* [466].

Image sources and databases

The distribution of the image sources is presented in Table 1. Most commonly, the image data were collected from non-public sources (254 articles). Publicly available data with reference segmentations were used in 217 articles. The most popular database was *BraTS* [464] (157 articles) followed by the *3D Segmentation in the Clinic: A Grand Challenge II: MS Lesion Segmentation* [463] and *ISLES* (17 articles) [466]. 10 articles rely on publicly available sources without reference segmentations. 41 articles validate the method on both publicly available datasets with reference segmentations and non-public data.

Table 1: Distribution of image sources used for algorithm validation.

	Publicly available data with reference segmentations				
Non-public data sources	BraTS [464]	Grand Challenge II: MS Lesion Segmentation [463]	ISLES [466]	Other	Publicly available data without reference segmentations
254	157	31	17	12	10

The distribution of the cohort sizes used to validate the proposed methods (Figure 4) shows that more than half of the studies described in included articles test methods on 50 or fewer individuals' brain images (260/441). Among the eligible articles, 280/441 describe a method validated on images obtained from more than one scanner.

Sequences

The majority of the proposed methods operated on multi-sequence scans as inputs (307/441) while 6 accepted both single- and multi-sequence input. 79 methods were built to evaluate single-sequence input and 22 did not specify the input modality. Articles that compared or evaluated a previously proposed method were not included in the analysis (27/441).

Image processing steps, algorithms and computational theories

Preprocessing procedures

The most commonly applied preprocessing procedures and tools (disregarding publicly available databases for this purpose) are shown in Table 2. Although authors commonly document individual steps and corresponding algorithms, information on whether these algorithms are integrated in the segmentation tool is rarely available [90,195]. Fifteen articles mention visual supervision [93,114,133], semiautomatic preprocessing [186,226,267,328,405], manual correction [155,171,209,273] , error or failure of preprocessing (usually at the brain extraction step) [135,136,160].

Five methods proposed in the reviewed articles use minimal [268,400] or no preprocessing [163,164,220], and in each case this is presented as an advantage of the proposed algorithm.

Table 2: Prevalent image preprocessing steps in 374/414 articles (not specified in 67 articles).

For peer review only

Procedure	Common tool/approach	N (of 374)
Intensity normalization	Histogram matching, intensity scaling	224
Bias field (field inhomogeneity) correction	N3, N4 [467,468]	192
Brain extraction	FSL BET [469]	190
Image (co-)registration	rigid/affine; SPM, FSL [470,471]	179
Denoising	anisotropic diffusion filtering	60
None or minimal		5

Algorithms and computational theories

Among the most commonly used algorithms or models in the segmentation methods were machine learning (decision trees, mixture models, fuzzy clustering, support vector machines, expectation maximization) and in particular deep-learning algorithms (artificial neural networks) (Figure 5). The analysis accounted for the same methods published in separate articles that deal with either different lesion types or similar lesion type with different image sources.

Performance evaluation

Comparison with the reference by overlap measures was by far the most commonly used criterion to evaluate the accuracy of the automatic segmentation. Among articles that specify how the reference segmentations had been generated (265) in publicly non-available data sets, most specified manual segmentation by one rater (85) or two raters (61). More than two raters contributed in 38 articles, and 33 articles used semiautomatic procedures for constructing the reference. When multiple raters delineated the region of interest, the reference segmentation was obtained through consensus of the raters or majority voting. Forty-five articles have also included inter- and intra-rater variability evaluation on data sets used in their studies. The most frequently applied measures were the Dice coefficient (330), sensitivity, also expressed as true positive ratio TPR, or overlap fraction (227), positive predictive value (102), Jaccard coefficient (58), and specificity (82). Volumetric measures, such as volume difference, error or correlation between the automatic and reference segmentation were used in 133 articles, and distance-based measures (such as Hausdorff distance, mean absolute distance, surface distance) in 41.

In addition to segmentation accuracy, reliability of a method was estimated in some of the articles. Reliability was directly evaluated through a test-retest procedure, where participants were scanned twice at a short interval, with repositioning between the scans in five articles [127,177,196,321,347]. In other articles, indirect approaches were described where consistency of longitudinal data with clinical findings of stable or progressive disease was used as a proxy for reliability [38,270,454].

Performance comparison

Maldjian et al. (2013) [133], Lesjak et al. (2016) [150], and de Sitter et al. (2017) [224] examined previously proposed methods and compared the results they obtained in their studies to the performance reported in the original papers. All three articles reported poorer independent testing results. The authors pointed out a lower lesion load of the studied population than in the original papers that accounted for reduced scores in the replication attempts. In their paper, de Sitter et al. (2017) [224] strongly called for improvements to automatic lesion segmentation before their introduction to routine clinical use.

For peer review only - http://bmjopen.bmj.com/site/about/guidelines.xhtml

Of 441 articles, 233 included information regarding both the processing time (from less than 1 second to 7 hours) and computational system requirements used for segmentation. In 56 articles we found information about only one of the two parameters, however such information is incomplete and cannot be used to estimate how well a method will perform on different hardware.

Only 24 of the included articles reported on methods that were made publicly available to download [43,50,53,67,76,84,90–92,126,134,138,144,149,154,170,173,178,190,195,196,199,228,230,426]. One was available upon request [111]. Two articles promised future availability of the methods proposed [41,193] but they were not available as of 4th of May 2020.

Reporting recommendations

Based on our findings, we developed a checklist with reporting recommendations (Table 3).

DISCUSSION

In this paper, we present a scoping study of automatic brain lesion segmentation on magnetic resonance images based on rigorous literature review methodology [17]. This is the first review that takes all methods into account, independent of specialization towards lesions of a particular etiology. The key findings from our analysis of 441 articles are a) a rapid increase of interest in the field, b) a plethora of proposed methods contrasted with a dearth of open documentation and available software, and c) high prevalence of problematic reporting practices that restrict the ability of independent researchers to replicate reported results and conduct method comparisons.

Variable design of automatic segmentation methods

RQ1: Which common image processing steps are necessary for automatic brain lesion segmentation on MR images?

Image preprocessing emerged as a pervasive step in the processing chain for lesion segmentation. From the collected data we identified procedures that are commonly agreed to constitute preprocessing: skull stripping, image co-registration, bias field correction, and intensity normalization. Some algorithms rely on additional preprocessing, such as tissue classification. The procedures vary considerably between methods, and a lack of a universal distinction between segmentation steps and preprocessing steps became apparent during the analysis.

For a clinically suitable method, a distinction between preprocessing and segmentation steps may not be necessary since the final segmentation will rely on a whole processing chain (including the preprocessing) applied to a raw image. Validation of the whole chain, and assessing the impact of each step on the outcome, however, are desirable in method evaluation studies. Unfortunately, authors rarely state whether preprocessing is integrated with the segmentation algorithm and to what extent it relies on user input. Without such information, namely a list of all the steps that are performed on a raw image, the study cannot be replicated. This implies that the findings are of no help in assessing the potential clinical validity of the proposed method implementation.

Another implementation issue arises from the necessity of visual supervision or manual corrections of the preprocessing that was indicated in 15 articles. Any requirement of user interaction at any point in the processing chain entails disadvantages: it impedes implementation by increasing the complexity of the technical integration, and, once the tool is implemented, permanently burdens staff with an additional task. Any potential benefit of user interaction (e.g. increased robustness) has to be weighed carefully against these costs.

RQ2: Which mathematical and computational theories are most commonly applied in which types of brain lesions?

The most prevalent methods utilized were artificial neural networks. The finding is not surprising given the popularity and remarkable performance of deep learning algorithms particularly in image processing applications. In the absence of widely used and agreed-upon criteria for performance evaluation, we have abstained from attempting to rank methods or make specific recommendations.

Validation process and efficacy of automatic segmentation methods

RQ2: *What is the efficacy of existing implementations?*

We found that in view of current practices, the question cannot be answered: authors present assessments that are predicated on their own needs and biases, and there is no established standard that enables fair method comparison. Public challenges have been set up in an effort to address this problem, but they have only been partially successful, as illustrated in the following section. Three obstacles to fair comparative method assessments are paramount: the principal lack of ground truth in *in vivo* imaging, the enormous parameter space of acquisition settings that leads to variable feature presentations, and the lack of objectivity (as shown by inter-rater and intra-rater variability [472,473]) of reference segmentations. More effort should thus be devoted to increasing the informativeness of the validation step. This can be achieved by painstakingly reporting the number of annotators, their experience, how the delineations were fused, and acquisition details that could conceivably have an impact. According to Gibson et al. [474], such information can be used to calculate the statistical power of segmentation studies with respect to the number of reference images.

To answer the question we posed regarding the efficacy, we recorded the values of the comparison measures from each article. The information, however, cannot be meaningfully synthesised or compared and was not presented in the results section for this reason. Our investigation did not result in a direct answer to the question, but revealed a paucity of standard comparison procedures.

Inability to answer the posed question points to further issues on which we reflect from the perspective of clinical suitability of the proposed methods. The medical image analysis procedures commonly used in clinical diagnostics today are strongly operator-dependent. They also scale poorly to the growing workload that results from the increasing number of modalities [475], the increasing accessibility of imaging scanners [476], and the increasing amount of image information due to technical advances that achieve enhanced spatial and contrast resolution [477]. Automatic image analysis methods in general, and among them automatic lesion segmentation methods promise to alleviate some of this pressure. Other requisite characteristics that need to be assessed to evaluate the clinical applicability of a tool are reliability, robustness, and generalizability of its findings.

Reliability of an automatic segmentation algorithm seems to be assumed since the decision whether a given voxel is a lesion or not is made based on well-defined rules. However, the characteristic is rarely tested in method validation studies. Only five articles in our sample included a test-retest evaluation with patient repositioning between the scans. Such testing yields crucial reliability data against which longitudinal variations of lesion measures need to be compared to distinguish actual lesion change from other sources of variability.

Generalizability of a method is tested by processing images acquired from different scanners on a sufficient number of images from populations that are at least as heterogeneous as the population expected to be examined clinically. While more than half of the articles (280/441) used images coming from more than one scanner, validation on fewer than 50 images were equally prevalent (260/441). Such small sample sizes are insufficient for clinical validation, even if the images originate from multiple scanners. Using images acquired from multiple sources and validating a method on a large number of images reduces a method's bias towards the data set or sets on which a given method was originally developed.

To us, one of the most surprising findings was how few methods are publicly available. Authors who share their software enable independent testing in unanticipated conditions and with various cohorts. Finally, to evaluate clinical relevance of the results of automatic segmentation, a method should be tested in genuine clinical scenarios for a well-defined task, such as monitoring disease progression, treatment response evaluation, or radiotherapy planning. Each of these tasks may require a different level of error margin and validation standard and the relevance of the results must be evaluated by clinicians. Various metrics may be used to either optimize the method or evaluate its accuracy for a particular clinical question, lesion type, and size.

Many papers claim usefulness of their algorithm for particular clinical tasks, but do not test the acceptability of the results for the intended purpose.

Limitations and issues of the proposed methods, and grand challenges of the field

RQ4 a) What are the limitations of those methods (...)?

Due to the inherent reference, image, and lesion variability, a meaningful comparison of algorithms evaluated independently on separate data sets to establish which method performs better is challenging. One solution to the problem has been proposed in the form of segmentation challenges. The challenges have become a quasi-standard for comparing brain lesion segmentation algorithms. The setup, however, comes with certain shortcomings with respect to evaluating clinical applicability of the evaluated algorithms. As mentioned, evaluating the whole chain of processing steps is especially important for validation of a clinical tool. In a challenge setup, the algorithms are tested on partially preprocessed images. Even when the individual steps are described in detail, the impact of the preprocessing on the segmentation algorithms outcome is unknown. Validation of the processing chain becomes questionable if a step is changed, either based on the users' subjective judgement or due to implementation of a different procedure for a particular step. According to *ISLES* [466] and several articles in our sample, the skull-stripping step may need to be supervised and manually corrected. The choice of interpolation method is yet another issue that can influence the outcome of an automatic segmentation algorithm, especially in tasks requiring high accuracy.

Another aspect of the challenges is the prestige and publicity for the authors and their method after proposing a winning segmentation algorithm. The final rank is calculated by the organizers who evaluate the methods on hidden data sets. Organizers effectively take the role of independent arbiters. Participation in challenges and post-contest use of the challenge data as reference material indicate that organizers are generally trusted in this role. Maier-Hein et al. suggest, however, that the results of such competitions should be considered carefully [478]. The authors point to several issues that have a significant impact on the final ranking of evaluated methods. The first problem they report is lack of thorough reporting of relevant information that is essential for result interpretation. Moreover, it turns out that changes in metrics and aggregation methods for the scores of individual test cases alter the ranking of evaluated methods. A similar effect on the ranking was observed when the reference segmentations were exchanged against those of another rater. The final rank in such a competition depends also on the test data and on how missing data is handled.

These concerns cast doubt on the validity of testing clinical applicability of automatic brain lesion segmentation methods in a challenge setup. Even if the challenge databases contain images from multiple scanners, it cannot be regarded as generalizable if it has not been tested on raw images (reconstructed, but not otherwise processed) as produced by the scanning equipment. In our sample, only 10% of the articles explicitly report on such evaluation. While obtaining independent data sets is expensive, it is a crucial step on the way of creating a clinically applicable automatic lesion segmentation tool.

Ultimately, it is the target users who need to decide whether a given validation standard and resultant performance confidence are sufficient to apply a tool to answer a given clinical question. With guidance from support system developers, as well as transparent and thorough reporting of the processing steps and validation procedures, the users may consider a given tool trustworthy. Trustworthiness has been reported as a crucial factor in developing a clinically usable support tool [479]. The present scoping review does not show what properties and features clinicians need to develop trust in a tool. Still, this is an important question that we will seek to address in future work.

We conducted an exploratory study to begin to address the issue [480]. The aim was to learn how considering radiologists' competent input can improve the design and validation procedures of automatic brain lesion segmentation methods with a view to increasing trust and trustworthiness. Our findings corroborate previous findings [479]. We found that two crucial characteristics that influence clinicians' trust in a tool are the provision of an error margin with any quantitative measure, and consideration of the varying need for accuracy, depending on the diagnostic task.

Relevance for research and clinical practice

Noteworthy trends also emerged from the data regarding the prevalence of methods developed for segmentation of a particular lesion type, as well as the advent of segmentation challenges focussing on corresponding lesion types. We observed that the majority of the methods described in our sample have been developed for the purpose of brain tumour segmentation. We also note consistent growth of the number of images in the database underlying the *BraTS* challenge, which has been organised annually since 2012. Stroke lesion segmentation methods have not proliferated to the same extent, despite similar competitions having been organized (yearly *ISLES* challenges from 2015 to

2018). A distinct increase in the number of publications proposing white-matter lesion segmentation algorithms was observed when segmentation challenges of this type of lesion were organized.

Some compelling questions follow this observation, e.g. whether some tasks are more difficult to solve algorithmically, or whether these trends reflect on clinical usefulness of developing segmentation algorithms for a particular lesion type. Even though the questions cannot be directly answered by our findings, they point to important issues that need to be explored to better understand the relevance of different lesion segmentation algorithms and implications of the trends for clinical practice.

Another finding that potentially contributes to the gap between research and clinical practice is the widespread use of deep-learning algorithms for the lesion segmentation task in the research setting. The practical efficiency of the algorithms depends on the availability of graphical processing units (GPUs). Implementing a deep-learning based algorithm in a clinical setting requires dedicating resources for purchasing suitable hardware and integrating it with radiological workflow, information, and storage systems.

A key factor in developing a clinical tool is obtaining regulatory approval for diagnostic use. None of the articles in our sample mentioned this requirement, possibly because authors do not consider it relevant at the stage of development when publication occurs.

It appears that despite claims of clinical relevance made by many authors, development of brain lesion segmentation methods happens predominantly in an academic space, where technological challenges matter most and implementation hurdles are not explicitly considered. To increase the relevance and societal benefit of method development in the field, it will be necessary for developers to widen their perspective to include the critical path towards clinical implementation, on which users' demands and regulatory requirements have to be met.

Challenges and limitations of the study

The biggest challenges we had to address in this study were designing data charting categories, extracting the relevant information regarding both inclusion and exclusion criteria, and the actual charting of the data. Arksey and O'Malley [17] recommend deciding the inclusion criteria after becoming familiar with the identified relevant studies. For us, this was impossible due to the large size of the raw sample of original search results, along with the variability of both the type and the level of detail of reported information.

Our inclusion and exclusion criteria were defined based on a pilot analysis of articles selected randomly from the raw sample. These criteria were then applied to the full raw sample in hierarchical fashion. A major impediment at this stage was the inconsistent way the information is organized and presented in the articles. In the case of conference papers, the authors typically have to adhere to page limits, so certain compromises are inevitable. When it comes to journal publications, the methodology of a study should be thoroughly reported for the sake of reproducibility, but also to enable data extraction for systematic reviews.

Another challenge we faced and a limitation of the study comes from the mentioned incoherent way of reporting studies and a challenge to derive strict definitions of the inclusion and exclusion criteria. Often, the criteria we sought in the articles are not presented clearly, or the information is scarce. Keeping in mind the broad nature of a scoping review and the aim of mapping the field, the author conducting the scoping study (EG) chose to err on the side of inclusion if the information in an article did not allow a decisive application of the criteria. While this strategy, combined with a single-rater approach, introduces a certain amount of selection bias, this bias was at least consistent between articles. We believe the approach provides a reasonable tradeoff between transparency and reproducibility of the study, and fulfilling the objectives of conducting a scoping review.

Other sources of bias were excluded by design. In particular, we conducted the consultation interviews after the literature sampling step, eliminating potential selection bias arising from the interview results. While the strong dominance of articles treating tumour segmentation (cf. Figure 2) may seem surprising, we believe that it is an accurate reflection of the research community's interest.

This review does not consider modalities such as projection radiography, computed tomography, nuclear imaging, or ultrasonography. It focuses instead on MR, which is used to address a larger variety of brain lesions, provides more detailed information, and stands out among radiological techniques regarding the number of related publications.

We acknowledge self-critically that, due to the extensive nature of the task, the time taken, and the lessons learned during the work, some epistemic drift occurred, taking our focus away from the first two research questions and towards the question of clinical applicability that appears to be underserved by the current literature. Thanks to our early decision to hold ourselves to account by writing a detailed protocol [22], this drift stayed within reasonable boundaries and is well-documented, as we accounted for protocol modifications in the present work.

Recommendations for future work

RQ4 b) What issues should be addressed in future studies to develop a tool that is suitable for clinical use?

To address the shortcomings revealed by our analysis, we propose a set of recommendations for future studies as well as avenues researchers might follow that promise, in our estimation, to advance the field in the direction of enabling clinical decision support.

The most important recommendation arises from the variability encountered in many aspects of the field. We propose a checklist (Table 3) of items that should be reported in investigations of automatic brain lesion segmentation methods. We split the items into three levels of validation that we see as a potential path towards developing a clinical tool. Technical validation studies focus on developing the algorithm and evaluating its performance according to common criteria in the field. The pre-clinical validation focuses on the performance of an algorithm in a setting resembling clinical environment and a realistic use case. The final level, clinical validation, requires the tool to be compatible with hospital and radiology information systems and to be ready for the process of obtaining regulatory approval for clinical use if such approval has not been yet obtained. This checklist, especially the technical validation level, will facilitate replication as well as comparisons between methods and studies, both informal and in meta-analyses. If authors were to follow the checklist in future studies, this would be a step towards standardization of reporting in the interest of advancing knowledge and promoting implementation as clinical tools.

Table 3: Proposed reporting items for automatic brain lesion segmentation studies.

Technical validation	**Method**	List of processing steps necessary to apply to a raw image
		Computational system parameters
		Computation time
		Open documentation of the algorithm
	**Reference segmentation**	Number of raters
		Raters' training/experience
		Method of segmentation
		Method of consolidation (if multiple raters)
	**Validation**	List of validation metrics used
		Number of images (split into training/validation/testing if applicable)
		Input sequences
		Number of scanners used to acquire images
		Acquisition parameters
		Number of time points and intervals for longitudinal data
	**Results**	Mean, median, standard deviation for each validation metric
		Number of failed cases (if applicable)
Pre-clinical validation	**Patient information**	Diagnosis and level of verification e.g. clinical follow-up, tissue sampling, autopsy, etc.
		Clinical presentation
		Administered treatments (if applicable)

For peer review only - http://bmjopen.bmj.com/site/about/guidelines.xhtml

Clinical validation	**Clinical task**	Explicit definition of the clinical task for which the algorithm is applied (e.g. lesion growth estimation, treatment evaluation, radiotherapy planning).
	**User validation**	List of optimization metrics used for the clinical task (if applicable).<br>User's method of evaluating the outcome.
	**Information and storage system compatibility**	Compatibility with picture archiving, communication, and storage (PACS) systems
	**Regulatory approval**	Compliance with the regulatory approvals for software as a medical device.

With similar priority, we ask researchers who have developed or are working on automatic brain lesion segmentation algorithms to publish software implementations. The benefits and challenges with fully automated versus interactive preprocessing should be assessed in terms of segmentation accuracy and reproducibility for a given, clinically relevant task.

Few articles in our sample evaluated their algorithm on an independent data set on top of the challenge one. We encourage authors to endeavour to test their method on images from other sites and sources. We also encourage collaboration between the authors and independent researchers who may have access to annotated test images. A preprocessing and segmentation method that has an acceptable and consistent performance on images acquired from various sources should finally be tested in clinical conditions. Therefore we strongly advocate close collaboration between researchers and authors of well-performing methods with clinicians. In such a scenario, the acceptability of the method's performance to clinicians can be assessed in conjunction with its relevance for a given task. Moreover, an open dialogue between researchers and clinicians will help build an ABS system that meets the requirements for a clinically useful and usable tool.

Finally, efforts to define steps on the path towards designing and validating a clinically applicable ABS system should be made. We recommend consultation with stakeholders as a key element to verify the actual clinical needs and how to assess to what extent these needs are met by available research.

CONCLUSIONS

This scoping study of automatic brain lesion segmentation on magnetic resonance images shows a field growing at a rapid pace, an imbalance between proposed methods of which there are many and methods implemented for clinical application of which there are few, and a room for improvement of reporting practice with a view to enabling replication, method comparison and implementation. To promote this improvement, we propose a list of recommendations for future studies in the field. We identify knowledge gaps and potentially fruitful avenues for future research.

REFERENCES

1 Therasse P, Arbuck SG, Eisenhauer EA, *et al.* New Guidelines to Evaluate the Response to Treatment in Solid Tumors. *J Natl Cancer Inst* 2000;**92**:205–16. doi:10.1093/jnci/92.3.205

2 Miller AB, Hoogstraten B, Staquet M, *et al.* Reporting results of cancer treatment. *Cancer* 1981;**47**:207–14. doi:https://doi.org/10.1002/1097-0142(19810101)47:1<207::AID-CNCR2820470134>3.0.CO;2-6

3 Fazekas F, Chawluk JB, Alavi A, *et al.* MR signal abnormalities at 1.5 T in Alzheimer's dementia and normal aging. *AJR Am J Roentgenol* 1987;**149**:351–6. doi:10.2214/ajr.149.2.351

4 Ellingson BM, Wen PY, Cloughesy TF. Modified Criteria for Radiographic Response Assessment in Glioblastoma Clinical Trials. *Neurotherapeutics* 2017;**14**:307–20. doi:10.1007/s13311-016-0507-6

5 Rosenkrantz AB, Hanna TN, Babb JS, *et al.* Changes in Emergency Department Imaging: Perspectives From National Patient Surveys Over Two Decades. *J Am Coll Radiol* 2017;**14**:1282–90. doi:10.1016/j.jacr.2017.03.004

6 Smith-Bindman R, Miglioretti DL, Larson EB. Rising use of diagnostic medical imaging in a large integrated health system. *Health Aff (Millwood)* 2008;**27**:1491–502. doi:10.1377/hlthaff.27.6.1491

7 Bauer S, Wiest R, Nolte L-P, *et al.* A survey of MRI-based medical image analysis for brain tumor studies. *Phys Med Biol* 2013;**58**:R97-129. doi:10.1088/0031-9155/58/13/R97

8 Debette S, Markus HS. The clinical importance of white matter hyperintensities on brain magnetic resonance imaging: systematic review and meta-analysis. *BMJ* 2010;**341**. doi:10.1136/bmj.c3666

9 Deepa, Singh A. Review of brain tumor detection from MRI images. In: *2016 3rd International Conference on Computing for Sustainable Global Development (INDIACom)*. 2016.

10 García-Lorenzo D, Francis S, Narayanan S, *et al.* Review of automatic segmentation methods of multiple sclerosis white matter lesions on conventional magnetic resonance imaging. *Med Image Anal* 2013;**17**:1–18. doi:10.1016/j.media.2012.09.004

11 Ali Işın, Cem Direkoğlu, Melike Şah. Review of MRI-based Brain Tumor Image Segmentation Using Deep Learning Methods. *Procedia Computer Science* 2016;**102**:317–24. doi:10.1016/j.procs.2016.09.407

12 Ito KL, Kim H, Liew S-L. A comparison of automated lesion segmentation approaches for chronic stroke T1-weighted MRI data. *Hum Brain Mapp* 2019;**40**:4669–85. doi:10.1002/hbm.24729

13 Kumari N, Saxena S. Review of Brain Tumor Segmentation and Classification. In: *2018 International Conference on Current Trends towards Converging Technologies (ICCTCT)*. 2018. 1–6. doi:10.1109/ICCTCT.2018.8551004

14 Mortazavi D, Kouzani AZ, Soltanian-Zadeh H. Segmentation of multiple sclerosis lesions in MR images: a review. *Neuroradiology* 2012;**54**:299–320. doi:10.1007/s00234-011-0886-7

15 Roy S, Nag S, Maitra IK, *et al.* A Review on Automated Brain Tumor Detection and Segmentation from MRI of Brain. *arXiv:13126150 [cs]* Published Online First: 16 December 2013.http://arxiv.org/abs/1312.6150 (accessed 23 Nov 2020).

16 Saritha S, Prabha NA. A comprehensive review: Segmentation of MRI images—brain tumor. *International Journal of Imaging Systems and Technology* 2016;**26**:295–304. doi:https://doi.org/10.1002/ima.22201

17 Arksey H, O'Malley L. Scoping studies: towards a methodological framework. *International Journal of Social Research Methodology* 2005;**8**:19–32. doi:10.1080/1364557032000119616

18 Levac D, Colquhoun H, O'Brien KK. Scoping studies: advancing the methodology. *Implementation Science* 2010;**5**:69. doi:10.1186/1748-5908-5-69

19 Colquhoun HL, Levac D, O'Brien KK, *et al.* Scoping reviews: time for clarity in definition, methods, and reporting. *J Clin Epidemiol* 2014;**67**:1291–4. doi:10.1016/j.jclinepi.2014.03.013

20 Moher D, Liberati A, Tetzlaff J, *et al.* Preferred reporting items for systematic reviews and meta-analyses: the PRISMA statement. *PLoS Med* 2009;**6**:e1000097. doi:10.1371/journal.pmed.1000097

21 Tricco AC, Lillie E, Zarin W, *et al.* PRISMA Extension for Scoping Reviews (PRISMA-ScR): Checklist and Explanation. *Ann Intern Med* 2018;**169**:467–73. doi:10.7326/M18-0850

For peer review only

22 Gryska EA, Schneiderman J, Heckemann RA. Automatic brain lesion segmentation on standard MRIs of the human head: a scoping review protocol. *BMJ Open* 2019;**9**:e024824. doi:10.1136/bmjopen-2018-024824

23 Zijdenbos AP, Forghani R, Evans AC. Automatic "pipeline" analysis of 3-D MRI data for clinical trials: application to multiple sclerosis. *IEEE Trans Med Imaging* 2002;**21**:1280–91. doi:10.1109/TMI.2002.806283

24 Zhuge Y, Krauze AV, Ning H, *et al.* Brain Tumor Segmentation using Holistically-Nested Neural Networks in MRI Images. *Med Phys* 2017;**44**:5234–43. doi:10.1002/mp.12481

25 Zhong Y, Utriainen D, Wang Y, *et al.* Automated White Matter Hyperintensity Detection in Multiple Sclerosis Using 3D T2 FLAIR. *International Journal of Biomedical Imaging* 2014;**2014**:e239123. doi:https://doi.org/10.1155/2014/239123

26 Zhao Z, Yang G, Lin Y, *et al.* Automated glioma detection and segmentation using graphical models. *PLOS ONE* 2018;**13**:e0200745. doi:10.1371/journal.pone.0200745

27 Zhao X, Wu Y, Song G, *et al.* A deep learning model integrating FCNNs and CRFs for brain tumor segmentation. *Med Image Anal* 2018;**43**:98–111. doi:10.1016/j.media.2017.10.002

28 Zhao L, Jia K. Multiscale CNNs for Brain Tumor Segmentation and Diagnosis. *Computational and Mathematical Methods in Medicine* 2016;**2016**:e8356294. doi:https://doi.org/10.1155/2016/8356294

29 Zhang R, Zhao L, Lou W, *et al.* Automatic Segmentation of Acute Ischemic Stroke From DWI Using 3-D Fully Convolutional DenseNets. *IEEE Trans Med Imaging* 2018;**37**:2149–60. doi:10.1109/TMI.2018.2821244

30 Zhang N, Ruan S, Lebonvallet S, *et al.* Kernel feature selection to fuse multi-spectral MRI images for brain tumor segmentation. *Computer Vision and Image Understanding* 2011;**115**:256–69. doi:10.1016/j.cviu.2010.09.007

31 Zhang J, Barboriak DP, Hobbs H, *et al.* A fully automatic extraction of magnetic resonance image features in glioblastoma patients. *Med Phys* 2014;**41**:042301. doi:10.1118/1.4866218

32 Zhan T, Chen Y, Hong X, *et al.* Brain Tumor Segmentation Using Deep Belief Networks and Pathological Knowledge. *CNS Neurol Disord Drug Targets* 2017;**16**:129–36. doi:10.2174/1871527316666170113101559

33 Zhan T, Shen F, Hong X, *et al.* A Glioma Segmentation Method Using CoTraining and Superpixel-Based Spatial and Clinical Constraints. *IEEE Access* 2018;**6**:57113–22. doi:10.1109/ACCESS.2018.2873674

34 Zacharaki EI, Bezerianos A. Abnormality Segmentation in Brain Images Via Distributed Estimation. *IEEE Transactions on Information Technology in Biomedicine* 2012;**16**:330–8. doi:10.1109/TITB.2011.2178422

35 Yu R, Xiao L, Wei Z. Coarse Classification to Region-Scalable Refining for White Matter Lesions Segmentation in Multi-Channel MRI. *CNS Neurol Disord Drug Targets* 2017;**16**:150–9. doi:10.2174/1871527315666161220145247

36 Yoo BI, Lee JJ, Han JW, *et al.* Application of variable threshold intensity to segmentation for white matter hyperintensities in fluid attenuated inversion recovery magnetic resonance images. *Neuroradiology* 2014;**56**:265–81. doi:10.1007/s00234-014-1322-6

37 Wu W, Chen AYC, Zhao L, *et al.* Brain tumor detection and segmentation in a CRF (conditional random fields) framework with pixel-pairwise affinity and superpixel-level features. *Int J Comput Assist Radiol Surg* 2014;**9**:241–53. doi:10.1007/s11548-013-0922-7

38 Weizman L, Ben Sira L, Joskowicz L, *et al.* Automatic segmentation, internal classification, and follow-up of optic pathway gliomas in MRI. *Med Image Anal* 2012;**16**:177–88. doi:10.1016/j.media.2011.07.001

39 Wang Y, Zhou Y, Wang H, *et al.* Voxel-based automated detection of focal cortical dysplasia lesions using diffusion tensor imaging and T2-weighted MRI data. *Epilepsy & Behavior* 2018;**84**:127–34. doi:10.1016/j.yebeh.2018.04.005

40 Wang Y, Catindig JA, Hilal S, *et al.* Multi-stage segmentation of white matter hyperintensity, cortical and lacunar infarcts. *Neuroimage* 2012;**60**:2379–88. doi:10.1016/j.neuroimage.2012.02.034

41 Wang R, Li C, Wang J, *et al.* Automatic segmentation and volumetric quantification of white matter hyperintensities on fluid-attenuated inversion recovery images using the extreme value distribution. *Neuroradiology* 2015;**57**:307–20. doi:10.1007/s00234-014-1466-4

42 Wang R, Li C, Wang J, *et al.* Automatic segmentation of white matter lesions on magnetic resonance images of the brain by using an outlier detection strategy. *Magnetic Resonance Imaging* 2014;**32**:1321–9. doi:10.1016/j.mri.2014.08.010

43 Wang R, Li C, Wang J, *et al.* Automatic Segmentation and Quantitative Analysis of White Matter Hyperintensities on FLAIR Images Using Trimmed-Likelihood Estimator. *Academic Radiology* 2014;**21**:1512–23. doi:10.1016/j.acra.2014.07.001

44 Wang H, Yushkevich PA. Multi-atlas segmentation without registration: a supervoxel-based approach. *Med Image Comput Comput Assist Interv* 2013;**16**:535–42. doi:10.1007/978-3-642-40760-4_67

45 Vishnuvarthanan A, Rajasekaran MP, Govindaraj V, *et al.* Development of a combinational framework to concurrently perform tissue segmentation and tumor identification in T1 - W, T2 - W, FLAIR and MPR type magnetic resonance brain images. *Expert Systems with Applications* 2018;**95**:280–311. doi:10.1016/j.eswa.2017.11.040

46 Vijayakumar C, Damayanti G, Pant R, *et al.* Segmentation and grading of brain tumors on apparent diffusion coefficient images using self-organizing maps. *Comput Med Imaging Graph* 2007;**31**:473–84. doi:10.1016/j.compmedimag.2007.04.004

47 Vidyaratne L, Alam M, Shboul Z, *et al.* Deep Learning and Texture-Based Semantic Label Fusion for Brain Tumor Segmentation. *Proc SPIE Int Soc Opt Eng* 2018;**2018**. doi:10.1117/12.2292930

48 Verma R, Zacharaki EI, Ou Y, *et al.* Multiparametric tissue characterization of brain neoplasms and their recurrence using pattern classification of MR images. *Acad Radiol* 2008;**15**:966–77. doi:10.1016/j.acra.2008.01.029

49 Van Leemput K, Maes F, Vandermeulen D, *et al.* Automated segmentation of multiple sclerosis lesions by model outlier detection. *IEEE Trans Med Imaging* 2001;**20**:677–88. doi:10.1109/42.938237

50 Valverde S, Cabezas M, Roura E, *et al.* Improving automated multiple sclerosis lesion segmentation with a cascaded 3D convolutional neural network approach. *NeuroImage* Published Online First: 2017. doi:10.1016/j.neuroimage.2017.04.034

51 Valcarcel AM, Linn KA, Vandekar SN, *et al.* MIMoSA: An Automated Method for Inter-Modal Segmentation Analysis of Multiple Sclerosis Brain Lesions. *J Neuroimaging* 2018;**28**:389–98. doi:10.1111/jon.12506

52 Usman K, Rajpoot K. Brain tumor classification from multi-modality MRI using wavelets and machine learning. *Pattern Anal Applic* 2017;**20**:871–81. doi:10.1007/s10044-017-0597-8

For peer review only - http://bmjopen.bmj.com/site/about/guidelines.xhtml

53 Tustison NJ, Shrinidhi KL, Wintermark M, *et al.* Optimal Symmetric Multimodal Templates and Concatenated Random Forests for Supervised Brain Tumor Segmentation (Simplified) with ANTsR. *Neuroinformatics* 2015;**13**:209–25. doi:10.1007/s12021-014-9245-2

54 Tsai J-Z, Peng S-J, Chen Y-W, *et al.* Automated Segmentation and Quantification of White Matter Hyperintensities in Acute Ischemic Stroke Patients with Cerebral Infarction. *PLoS One* 2014;**9**. doi:10.1371/journal.pone.0104011

55 Tsai J-Z, Peng S-J, Chen Y-W, *et al.* Automatic Detection and Quantification of Acute Cerebral Infarct by Fuzzy Clustering and Histographic Characterization on Diffusion Weighted MR Imaging and Apparent Diffusion Coefficient Map. *BioMed Research International* 2014;**2014**:e963032. doi:https://doi.org/10.1155/2014/963032

56 Tong J, Zhao Y, Zhang P, *et al.* MRI brain tumor segmentation based on texture features and kernel sparse coding. *Biomedical Signal Processing and Control* 2019;**47**:387–92. doi:10.1016/j.bspc.2018.06.001

57 Tong J, Zhang P, Weng Y, *et al.* Kernel sparse representation for MRI image analysis in automatic brain tumor segmentation. *Frontiers Inf Technol Electronic Eng* 2018;**19**:471–80. doi:10.1631/FITEE.1620342

58 Tomas-Fernandez X, Warfield SK. A Model of Population and Subject (MOPS) Intensities with Application to Multiple Sclerosis Lesion Segmentation. *IEEE Trans Med Imaging* 2015;**34**:1349–61. doi:10.1109/TMI.2015.2393853

59 Thomas T, S AR. A Novel Automatic Method for Extraction of Glioma Tumor, White matter and Grey matter from Brain Magnetic Resonant Images. *Biomedical imaging intervention Journal* 2013;**9**. doi:10.2349/biij.9.2.e21

60 Thiruvenkadam K, Perumal N. Fully automatic method for segmentation of brain tumor from multimodal magnetic resonance images using wavelet transformation and clustering technique. *International Journal of Imaging Systems and Technology* 2016;**26**:305–14. doi:https://doi.org/10.1002/ima.22202

61 Szwarc P, Kawa J, Rudzki M, *et al.* Automatic brain tumour detection and neovasculature assessment with multiseries MRI analysis. *Computerized Medical Imaging and Graphics* 2015;**46**:178–90. doi:10.1016/j.compmedimag.2015.06.002

62 Sweeney EM, Vogelstein JT, Cuzzocreo JL, *et al.* A Comparison of Supervised Machine Learning Algorithms and Feature Vectors for MS Lesion Segmentation Using Multimodal Structural MRI. *PLoS One* 2014;**9**. doi:10.1371/journal.pone.0095753

63 Sweeney EM, Shinohara RT, Shiee N, *et al.* OASIS is Automated Statistical Inference for Segmentation, with applications to multiple sclerosis lesion segmentation in MRI. *NeuroImage: Clinical* 2013;**2**:402–13. doi:10.1016/j.nicl.2013.03.002

64 Sweeney EM, Shinohara RT, Shea CD, *et al.* Automatic Lesion Incidence Estimation and Detection in Multiple Sclerosis Using Multisequence Longitudinal MRI. *AJNR Am J Neuroradiol* 2013;**34**:68–73. doi:10.3174/ajnr.A3172

65 Sudre CH, Cardoso MJ, Bouvy WH, *et al.* Bayesian Model Selection for Pathological Neuroimaging Data Applied to White Matter Lesion Segmentation. *IEEE Transactions on Medical Imaging* 2015;**34**:2079–102. doi:10.1109/TMI.2015.2419072

66 Strumia M, Schmidt FR, Anastasopoulos C, *et al.* White Matter MS-Lesion Segmentation Using a Geometric Brain Model. *IEEE Transactions on Medical Imaging* 2016;**35**:1636–46. doi:10.1109/TMI.2016.2522178

67 Stone JR, Wilde EA, Taylor BA, *et al.* Supervised learning technique for the automated identification of white matter hyperintensities in traumatic brain injury. *Brain Injury* 2016;**30**:1458–68. doi:10.1080/02699052.2016.1222080

68 Steenwijk MD, Pouwels PJW, Daams M, *et al.* Accurate white matter lesion segmentation by k nearest neighbor classification with tissue type priors (kNN-TTPs). *NeuroImage: Clinical* 2013;**3**:462–9. doi:10.1016/j.nicl.2013.10.003

69 Steed TC, Treiber JM, Patel KS, *et al.* Iterative probabilistic voxel labeling: automated segmentation for analysis of The Cancer Imaging Archive glioblastoma images. *AJNR Am J Neuroradiol* 2015;**36**:678–85. doi:10.3174/ajnr.A4171

70 Soltaninejad M, Yang G, Lambrou T, *et al.* Supervised learning based multimodal MRI brain tumour segmentation using texture features from supervoxels. *Computer Methods and Programs in Biomedicine* 2018;**157**:69–84. doi:10.1016/j.cmpb.2018.01.003

71 Soltaninejad M, Yang G, Lambrou T, *et al.* Automated brain tumour detection and segmentation using superpixel-based extremely randomized trees in FLAIR MRI. *Int J CARS* 2017;**12**:183–203. doi:10.1007/s11548-016-1483-3

72 Smart SD, Firbank MJ, O'Brien JT. Validation of Automated White Matter Hyperintensity Segmentation. *Journal of Aging Research* 2011;**2011**:e391783. doi:https://doi.org/10.4061/2011/391783

73 Sivakumar P, Ganeshkumar P. An efficient automated methodology for detecting and segmenting the ischemic stroke in brain MRI images. *International Journal of Imaging Systems and Technology* 2017;**27**:265–72. doi:https://doi.org/10.1002/ima.22231

74 Simões R, Mönninghoff C, Dlugaj M, *et al.* Automatic segmentation of cerebral white matter hyperintensities using only 3D FLAIR images. *Magn Reson Imaging* 2013;**31**:1182–9. doi:10.1016/j.mri.2012.12.004

75 Shimol EB, Joskowicz L, Eliahou R, *et al.* Computer-based radiological longitudinal evaluation of meningiomas following stereotactic radiosurgery. *Int J CARS* 2018;**13**:215–28. doi:10.1007/s11548-017-1673-7

76 Shiee N, Bazin P-L, Ozturk A, *et al.* A topology-preserving approach to the segmentation of brain images with multiple sclerosis lesions. *Neuroimage* 2010;**49**:1524–35. doi:10.1016/j.neuroimage.2009.09.005

77 Shi L, Wang D, Liu S, *et al.* Automated quantification of white matter lesion in magnetic resonance imaging of patients with acute infarction. *J Neurosci Methods* 2013;**213**:138–46. doi:10.1016/j.jneumeth.2012.12.014

78 Selvathi D, Selvaraj H, Selvi ST. Hybrid approach for brain tumor segmentation in magnetic resonance images using cellular neural networks and optimization techniques. *Int J Comp Intel Appl* 2010;**09**:17–31. doi:10.1142/S1469026810002781

79 Scully M, Anderson B, Lane T, *et al.* An Automated Method for Segmenting White Matter Lesions through Multi-Level Morphometric Feature Classification with Application to Lupus. *Front Hum Neurosci* 2010;**4**. doi:10.3389/fnhum.2010.00027

80 Schmidt P, Gaser C, Arsic M, *et al.* An automated tool for detection of FLAIR-hyperintense white-matter lesions in Multiple Sclerosis. *Neuroimage* 2012;**59**:3774–83. doi:10.1016/j.neuroimage.2011.11.032

81 Sasikanth S, Kumar SS. Glioma tumor detection in brain MRI image using ANFIS-based normalized graph cut approach. *International Journal of Imaging Systems and Technology* 2018;**28**:64–71. doi:https://doi.org/10.1002/ima.22257

82 Sanjuán A, Price CJ, Mancini L, *et al.* Automated identification of brain tumors from single MR images based on segmentation with refined patient-specific priors. *Front Neurosci* 2013;**7**. doi:10.3389/fnins.2013.00241

83 Samaille T, Fillon L, Cuingnet R, *et al.* Contrast-based fully automatic segmentation of white matter hyperintensities: method and validation. *PLoS One* 2012;**7**:e48953. doi:10.1371/journal.pone.0048953

84 Salem M, Cabezas M, Valverde S, *et al.* A supervised framework with intensity subtraction and deformation field features for the detection of new T2-w lesions in multiple sclerosis. *NeuroImage: Clinical* 2018;**17**:607–15. doi:10.1016/j.nicl.2017.11.015

85 Saad M Norhashimah. Fully Automated Region Growing Segmentation of Brain Lesion in Diffusion-weighted MRI. *IAENG International Journal of Computer Science* 2012;**39**:10.

86 Rundo L, Militello C, Tangherloni A, *et al.* NeXt for neuro-radiosurgery: A fully automatic approach for necrosis extraction in brain tumor MRI using an unsupervised machine learning technique. *International Journal of Imaging Systems and Technology* 2018;**28**:21–37. doi:https://doi.org/10.1002/ima.22253

87 Roy S, He Q, Sweeney E, *et al.* Subject Specific Sparse Dictionary Learning for Atlas Based Brain MRI Segmentation. *IEEE J Biomed Health Inform* 2015;**19**:1598–609. doi:10.1109/JBHI.2015.2439242

88 Roy S, He Q, Carass A, *et al.* Example Based Lesion Segmentation. *Proc SPIE Int Soc Opt Eng* 2014;**9034**. doi:10.1117/12.2043917

89 Roy PK, Bhuiyan A, Janke A, *et al.* Automatic white matter lesion segmentation using contrast enhanced FLAIR intensity and Markov Random Field. *Computerized Medical Imaging and Graphics* 2015;**45**:102–11. doi:10.1016/j.compmedimag.2015.08.005

90 Roura E, Sarbu N, Oliver A, *et al.* Automated Detection of Lupus White Matter Lesions in MRI. *Front Neuroinform* 2016;**10**. doi:10.3389/fninf.2016.00033

91 Roura E, Oliver A, Cabezas M, *et al.* A toolbox for multiple sclerosis lesion segmentation. *Neuroradiology* 2015;**57**:1031–43. doi:10.1007/s00234-015-1552-2

92 Rios Velazquez E, Meier R, Dunn Jr WD, *et al.* Fully automatic GBM segmentation in the TCGA-GBM dataset: Prognosis and correlation with VASARI features. *Sci Rep* 2015;**5**. doi:10.1038/srep16822

93 Rincón M, Díaz-López E, Selnes P, *et al.* Improved Automatic Segmentation of White Matter Hyperintensities in MRI Based on Multilevel Lesion Features. *Neuroinform* 2017;**15**:231–45. doi:10.1007/s12021-017-9328-y

94 Reza S, Iftekharuddin K. Multi-class Abnormal Brain Tissue Segmentation Using Texture Features. *Proc NCI MICCAI-BRATS* 2013;**2013**:38–42.

95 Razzak MI, Imran M, Xu G. Efficient Brain Tumor Segmentation With Multiscale Two-Pathway-Group Conventional Neural Networks. *IEEE Journal of Biomedical and Health Informatics* 2019;**23**:1911–9. doi:10.1109/JBHI.2018.2874033

96 Raju AR, Suresh P, Rao RR. Bayesian HCS-based multi-SVNN: A classification approach for brain tumor segmentation and classification using Bayesian fuzzy clustering. *Biocybernetics and Biomedical Engineering* 2018;**38**:646–60. doi:10.1016/j.bbe.2018.05.001

97 Rajinikanth V, Satapathy SC, Fernandes SL, *et al.* Entropy based segmentation of tumor from brain MR images – a study with teaching learning based optimization. *Pattern Recognition Letters* 2017;**94**:87–95. doi:10.1016/j.patrec.2017.05.028

For peer review only

98 Rachmadi MF, Valdés-Hernández M del C, Agan MLF, *et al.* Segmentation of white matter hyperintensities using convolutional neural networks with global spatial information in routine clinical brain MRI with none or mild vascular pathology. *Computerized Medical Imaging and Graphics* 2018;**66**:28–43. doi:10.1016/j.compmedimag.2018.02.002

99 Qu X, Yang J, Ma S, *et al.* Positive Unanimous Voting Algorithm for Focal Cortical Dysplasia Detection on Magnetic Resonance Image. *Front Comput Neurosci* 2016;**10**. doi:10.3389/fncom.2016.00025

100 Qin C, Guerrero R, Bowles C, *et al.* A large margin algorithm for automated segmentation of white matter hyperintensity. *Pattern Recognition* 2018;**77**:150–9. doi:10.1016/j.patcog.2017.12.016

101 Pustina D, Coslett HB, Turkeltaub PE, *et al.* Automated segmentation of chronic stroke lesions using LINDA: Lesion identification with neighborhood data analysis. *Hum Brain Mapp* 2016;**37**:1405–21. doi:10.1002/hbm.23110

102 Praveen GB, Agrawal A, Sundaram P, *et al.* Ischemic stroke lesion segmentation using stacked sparse autoencoder. *Computers in Biology and Medicine* 2018;**99**:38–52. doi:10.1016/j.compbiomed.2018.05.027

103 Prakash K N B, Gupta V, Bilello M, *et al.* Identification, segmentation, and image property study of acute infarcts in diffusion-weighted images by using a probabilistic neural network and adaptive Gaussian mixture model. *Acad Radiol* 2006;**13**:1474–84. doi:10.1016/j.acra.2006.09.045

104 Porz N, Habegger S, Meier R, *et al.* Fully Automated Enhanced Tumor Compartmentalization: Man vs. Machine Reloaded. *PLoS One* 2016;**11**. doi:10.1371/journal.pone.0165302

105 Porz N, Bauer S, Pica A, *et al.* Multi-Modal Glioblastoma Segmentation: Man versus Machine. *PLOS ONE* 2014;**9**:e96873. doi:10.1371/journal.pone.0096873

106 Popuri K, Cobzas D, Murtha A, *et al.* 3D variational brain tumor segmentation using Dirichlet priors on a clustered feature set. *Int J Comput Assist Radiol Surg* 2012;**7**:493–506. doi:10.1007/s11548-011-0649-2

107 Pinto A, Pereira S, Rasteiro D, *et al.* Hierarchical brain tumour segmentation using extremely randomized trees. *Pattern Recognition* 2018;**82**:105–17. doi:10.1016/j.patcog.2018.05.006

108 Pereira S, Pinto A, Alves V, *et al.* Brain Tumor Segmentation Using Convolutional Neural Networks in MRI Images. *IEEE Transactions on Medical Imaging* 2016;**35**:1240–51. doi:10.1109/TMI.2016.2538465

109 Parisot S, Wells W, Chemouny S, *et al.* Concurrent tumor segmentation and registration with uncertainty-based sparse non-uniform graphs. *Medical Image Analysis* 2014;**18**:647–59. doi:10.1016/j.media.2014.02.006

110 Pagnozzi AM, Dowson N, Doecke J, *et al.* Automated, quantitative measures of grey and white matter lesion burden correlates with motor and cognitive function in children with unilateral cerebral palsy. *Neuroimage Clin* 2016;**11**:751–9. doi:10.1016/j.nicl.2016.05.018

111 Ozenne B, Subtil F, Østergaard L, *et al.* Spatially regularized mixture model for lesion segmentation with application to stroke patients. *Biostatistics* 2015;**16**:580–95. doi:10.1093/biostatistics/kxv004

112 Ong KH, Ramachandram D, Mandava R, *et al.* Automatic white matter lesion segmentation using an adaptive outlier detection method. *Magn Reson Imaging* 2012;**30**:807–23. doi:10.1016/j.mri.2012.01.007

113 Njeh I, Sallemi L, Ayed IB, *et al.* 3D multimodal MRI brain glioma tumor and edema segmentation: A graph cut distribution matching approach. *Computerized Medical Imaging and Graphics* 2015;**40**:108–19. doi:10.1016/j.compmedimag.2014.10.009

114 Nie J, Xue Z, Liu T, *et al.* Automated brain tumor segmentation using spatial accuracy-weighted hidden Markov Random Field. *Computerized Medical Imaging and Graphics* 2009;**33**:431–41. doi:10.1016/j.compmedimag.2009.04.006

115 Nagenthiraja K, Walcott BP, Hansen MB, *et al.* Automated Decision-Support System for Prediction of Treatment Responders in Acute Ischemic Stroke. *Front Neurol* 2013;**4**. doi:10.3389/fneur.2013.00140

116 naceur MB, Saouli R, Akil M, *et al.* Fully Automatic Brain Tumor Segmentation using End-To-End Incremental Deep Neural Networks in MRI images. *Computer Methods and Programs in Biomedicine* 2018;**166**:39–49. doi:10.1016/j.cmpb.2018.09.007

117 Nabizadeh N, Kubat M. Brain tumors detection and segmentation in MR images: Gabor wavelet vs. statistical features. *Computers & Electrical Engineering* 2015;**45**:286–301. doi:10.1016/j.compeleceng.2015.02.007

118 Murphy K, van der Aa NE, Negro S, *et al.* Automatic quantification of ischemic injury on diffusion-weighted MRI of neonatal hypoxic ischemic encephalopathy. *Neuroimage Clin* 2017;**14**:222–32. doi:10.1016/j.nicl.2017.01.005

119 Muda AF, Mohd Saad N, Abu Bakar SAR, *et al.* Brain lesion segmentation using fuzzy C-means on diffusion-weighted imaging. *ARPN Journal of Engineering and Applied Sciences* 2015;**10**:1138–44.

120 Mouridsen K, Nagenthiraja K, Jónsdóttir KÝ, *et al.* Acute stroke: automatic perfusion lesion outlining using level sets. *Radiology* 2013;**269**:404–12. doi:10.1148/radiol.13121622

121 Moeskops P, de Bresser J, Kuijf HJ, *et al.* Evaluation of a deep learning approach for the segmentation of brain tissues and white matter hyperintensities of presumed vascular origin in MRI. *NeuroImage: Clinical* 2018;**17**:251–62. doi:10.1016/j.nicl.2017.10.007

122 Mitra S, Banerjee S, Hayashi Y. Volumetric brain tumour detection from MRI using visual saliency. *PLOS ONE* 2017;**12**:e0187209. doi:10.1371/journal.pone.0187209

123 Mitra J, Bourgeat P, Fripp J, *et al.* Lesion segmentation from multimodal MRI using random forest following ischemic stroke. *NeuroImage* 2014;**98**:324–35. doi:10.1016/j.neuroimage.2014.04.056

124 Menze BH, Van Leemput K, Lashkari D, *et al.* A generative probabilistic model and discriminative extensions for brain lesion segmentation – with application to tumor and stroke. *IEEE Trans Med Imaging* 2016;**35**:933–46. doi:10.1109/TMI.2015.2502596

125 Meier R, Porz N, Knecht U, *et al.* Automatic estimation of extent of resection and residual tumor volume of patients with glioblastoma. *J Neurosurg* 2017;**127**:798–806. doi:10.3171/2016.9.JNS16146

126 Meier R, Knecht U, Loosli T, *et al.* Clinical Evaluation of a Fully-automatic Segmentation Method for Longitudinal Brain Tumor Volumetry. *Sci Rep* 2016;**6**:23376. doi:10.1038/srep23376

127 Meier DS, Guttmann CRG, Tummala S, *et al.* Dual-Sensitivity Multiple Sclerosis Lesion and CSF Segmentation for Multichannel 3T Brain MRI. *J Neuroimaging* 2018;**28**:36–47. doi:10.1111/jon.12491

128 Mehta S, Grabowski TJ, Trivedi Y, *et al.* Evaluation of voxel-based morphometry for focal lesion detection in individuals. *NeuroImage* 2003;**20**:1438–54. doi:10.1016/S1053-8119(03)00377-X

129 Mechrez R, Goldberger J, Greenspan H. Patch-Based Segmentation with Spatial Consistency: Application to MS Lesions in Brain MRI. *Int J Biomed Imaging* 2016;**2016**. doi:10.1155/2016/7952541

130 McKinley R, Häni L, Gralla J, *et al.* Fully automated stroke tissue estimation using random forest classifiers (FASTER). *J Cereb Blood Flow Metab* 2017;**37**:2728–41. doi:10.1177/0271678X16674221

131 Mazzara GP, Velthuizen RP, Pearlman JL, *et al.* Brain tumor target volume determination for radiation treatment planning through automated MRI segmentation. *International Journal of Radiation Oncology*Biology*Physics* 2004;**59**:300–12. doi:10.1016/j.ijrobp.2004.01.026

132 Manjón JV, Coupé P, Raniga P, *et al.* MRI white matter lesion segmentation using an ensemble of neural networks and overcomplete patch-based voting. *Computerized Medical Imaging and Graphics* 2018;**69**:43–51. doi:10.1016/j.compmedimag.2018.05.001

133 Maldjian JA, Whitlow CT, Saha BN, *et al.* Automated White Matter Total Lesion Volume Segmentation in Diabetes. *AJNR Am J Neuroradiol* 2013;**34**:2265–70. doi:10.3174/ajnr.A3590

134 Maji P, Roy S. SoBT-RFW: Rough-Fuzzy Computing and Wavelet Analysis Based Automatic Brain Tumor Detection Method from MR Images. *Fundam Informaticae* Published Online First: 2015. doi:10.3233/FI-2015-1293

135 Maier O, Wilms M, von der Gablentz J, *et al.* Extra Tree forests for sub-acute ischemic stroke lesion segmentation in MR sequences. *Journal of Neuroscience Methods* 2015;**240**:89–100. doi:10.1016/j.jneumeth.2014.11.011

136 Maier O, Schröder C, Forkert ND, *et al.* Classifiers for Ischemic Stroke Lesion Segmentation: A Comparison Study. *PLOS ONE* 2015;**10**:e0145118. doi:10.1371/journal.pone.0145118

137 Maier O. MS lesion segmentation in MRI with random forests. *Proc 2015 Longitudinal Multiple Sclerosis Lesion Segmentation Challenge* 2015;:1–2.

138 Mah Y-H, Jager R, Kennard C, *et al.* A new method for automated high-dimensional lesion segmentation evaluated in vascular injury and applied to the human occipital lobe. *Cortex* 2014;**56**:51–63. doi:10.1016/j.cortex.2012.12.008

139 Ma C, Luo G, Wang K. Concatenated and Connected Random Forests With Multiscale Patch Driven Active Contour Model for Automated Brain Tumor Segmentation of MR Images. *IEEE Transactions on Medical Imaging* 2018;**37**:1943–54. doi:10.1109/TMI.2018.2805821

140 Lu Y, Jiang J, Yang W, *et al.* Multimodal Brain-Tumor Segmentation Based on Dirichlet Process Mixture Model with Anisotropic Diffusion and Markov Random Field Prior. *Computational and Mathematical Methods in Medicine* 2014;**2014**:e717206. doi:https://doi.org/10.1155/2014/717206

141 Liu Y, Stojadinovic S, Hrycushko B, *et al.* A deep convolutional neural network-based automatic delineation strategy for multiple brain metastases stereotactic radiosurgery. *PLoS One* 2017;**12**:e0185844. doi:10.1371/journal.pone.0185844

142 Liu Y, Stojadinovic S, Hrycushko B, *et al.* Automatic metastatic brain tumor segmentation for stereotactic radiosurgery applications. *Phys Med Biol* 2016;**61**:8440–8461. doi:10.1088/0031-9155/61/24/8440

143 Liu J, Chen F, Pan C, *et al.* A Cascaded Deep Convolutional Neural Network for Joint Segmentation and Genotype Prediction of Brainstem Gliomas. *IEEE Transactions on Biomedical Engineering* 2018;**65**:1943–52. doi:10.1109/TBME.2018.2845706

144 Ling Y, Jouvent E, Cousyn L, *et al.* Validation and Optimization of BIANCA for the Segmentation of Extensive White Matter Hyperintensities. *Neuroinform* 2018;**16**:269–81. doi:10.1007/s12021-018-9372-2

For peer review only

145 Liberman G, Louzoun Y, Aizenstein O, *et al.* Automatic multi-modal MR tissue classification for the assessment of response to bevacizumab in patients with glioblastoma. *European Journal of Radiology* 2013;**82**:e87–94. doi:10.1016/j.ejrad.2012.09.001

146 Li Z, Wang Y, Yu J, *et al.* Low-Grade Glioma Segmentation Based on CNN with Fully Connected CRF. *Journal of Healthcare Engineering* 2017;**2017**:e9283480. doi:https://doi.org/10.1155/2017/9283480

147 Li Y, Jia F, Qin J. Brain tumor segmentation from multimodal magnetic resonance images via sparse representation. *Artificial Intelligence in Medicine* 2016;**73**:1–13. doi:10.1016/j.artmed.2016.08.004

148 Li W, Tian J, Li E, *et al.* Robust unsupervised segmentation of infarct lesion from diffusion tensor MR images using multiscale statistical classification and partial volume voxel reclassification. *Neuroimage* 2004;**23**:1507–18. doi:10.1016/j.neuroimage.2004.08.009

149 Li H, Jiang G, Zhang J, *et al.* Fully convolutional network ensembles for white matter hyperintensities segmentation in MR images. *NeuroImage* 2018;**183**:650–65. doi:10.1016/j.neuroimage.2018.07.005

150 Lesjak Ž, Pernuš F, Likar B, *et al.* Validation of White-Matter Lesion Change Detection Methods on a Novel Publicly Available MRI Image Database. *Neuroinform* 2016;**14**:403–20. doi:10.1007/s12021-016-9301-1

151 Laukamp KR, Thiele F, Shakirin G, *et al.* Fully automated detection and segmentation of meningiomas using deep learning on routine multiparametric MRI. *Eur Radiol* 2019;**29**:124–32. doi:10.1007/s00330-018-5595-8

152 Lao Z, Shen D, Liu D, *et al.* Computer-assisted segmentation of white matter lesions in 3D MR images using support vector machine. *Acad Radiol* 2008;**15**:300–13. doi:10.1016/j.acra.2007.10.012

153 Korfiatis P, Kline TL, Erickson BJ. Automated Segmentation of Hyperintense Regions in FLAIR MRI Using Deep Learning. *Tomography* 2016;**2**:334–40. doi:10.18383/j.tom.2016.00166

154 Knight J, Taylor GW, Khademi A. Voxel-Wise Logistic Regression and Leave-One-Source-Out Cross Validation for white matter hyperintensity segmentation. *Magnetic Resonance Imaging* 2018;**54**:119–36. doi:10.1016/j.mri.2018.06.009

155 Klöppel S, Abdulkadir A, Hadjidemetriou S, *et al.* A comparison of different automated methods for the detection of white matter lesions in MRI data. *NeuroImage* 2011;**57**:416–22. doi:10.1016/j.neuroimage.2011.04.053

156 Khotanlou H, Afrasiabi M. Segmentation of Multiple Sclerosis Lesions in Brain MR Images Using Spatially Constrained Possibilistic Fuzzy C-Means Classification. *J Med Signals Sens* 2011;**1**:149–55.

157 Khayati R, Vafadust M, Towhidkhah F, *et al.* Fully automatic segmentation of multiple sclerosis lesions in brain MR FLAIR images using adaptive mixtures method and Markov random field model. *Comput Biol Med* 2008;**38**:379–90. doi:10.1016/j.compbiomed.2007.12.005

158 Khayati R, Vafadust M, Towhidkhah F, *et al.* A novel method for automatic determination of different stages of multiple sclerosis lesions in brain MR FLAIR images. *Comput Med Imaging Graph* 2008;**32**:124–33. doi:10.1016/j.compmedimag.2007.10.003

159 Khademi A, Venetsanopoulos A, Moody AR. Robust White Matter Lesion Segmentation in FLAIR MRI. *IEEE Transactions on Biomedical Engineering* 2012;**59**:860–71. doi:10.1109/TBME.2011.2181167

160 Kellner-Weldon F, Stippich C, Wiest R, *et al.* Comparison of perioperative automated versus manual two-dimensional tumor analysis in glioblastoma patients. *European Journal of Radiology* 2017;**95**:75–81. doi:10.1016/j.ejrad.2017.07.028

For peer review only

161 Kellner E, Reisert M, Kiselev VG, *et al.* Automated Infarct Core Volumetry Within the Hypoperfused Tissue: Technical Implementation and Evaluation. *J Comput Assist Tomogr* 2017;**41**:515–20. doi:10.1097/RCT.0000000000000570

162 Keçeli AS, Can AB, Kaya A. A GPU-Based Approach for Automatic Segmentation of White Matter Lesions. *IETE Journal of Research* 2017;**63**:461–72. doi:10.1080/03772063.2017.1284619

163 Kaur T, Saini BS, Gupta S. A joint intensity and edge magnitude-based multilevel thresholding algorithm for the automatic segmentation of pathological MR brain images. *Neural Comput & Applic* 2018;**30**:1317–40. doi:10.1007/s00521-016-2751-4

164 Kaur T, Saini BS, Gupta S. A novel fully automatic multilevel thresholding technique based on optimized intuitionistic fuzzy sets and tsallis entropy for MR brain tumor image segmentation. *Australas Phys Eng Sci Med* 2018;**41**:41–58. doi:10.1007/s13246-017-0609-4

165 Karimian A, Jafari S. A New Method to Segment the Multiple Sclerosis Lesions on Brain Magnetic Resonance Images. *J Med Signals Sens* 2015;**5**:238–44.

166 Karimaghaloo Z, Arnold DL, Arbel T. Adaptive multi-level conditional random fields for detection and segmentation of small enhanced pathology in medical images. *Medical Image Analysis* 2016;**27**:17–30. doi:10.1016/j.media.2015.06.004

167 Karimaghaloo Z, Shah M, Francis SJ, *et al.* Automatic Detection of Gadolinium-Enhancing Multiple Sclerosis Lesions in Brain MRI Using Conditional Random Fields. *IEEE Transactions on Medical Imaging* 2012;**31**:1181–94. doi:10.1109/TMI.2012.2186639

168 Karimaghaloo Z, Rivaz H, Arnold DL, *et al.* Temporal Hierarchical Adaptive Texture CRF for Automatic Detection of Gadolinium-Enhancing Multiple Sclerosis Lesions in Brain MRI. *IEEE Transactions on Medical Imaging* 2015;**34**:1227–41. doi:10.1109/TMI.2014.2382561

169 Kanas VG, Zacharaki EI, Davatzikos C, *et al.* A low cost approach for brain tumor segmentation based on intensity modeling and 3D Random Walker. *Biomedical Signal Processing and Control* 2015;**22**:19–30. doi:10.1016/j.bspc.2015.06.004

170 Kamnitsas K, Ledig C, Newcombe VFJ, *et al.* Efficient multi-scale 3D CNN with fully connected CRF for accurate brain lesion segmentation. *Medical Image Analysis* 2017;**36**:61–78. doi:10.1016/j.media.2016.10.004

171 Kamber M, Shinghal R, Collins DL, *et al.* Model-based 3-D segmentation of multiple sclerosis lesions in magnetic resonance brain images. *IEEE Transactions on Medical Imaging* 1995;**14**:442–53. doi:10.1109/42.414608

172 Juan-Albarracín J, Fuster-Garcia E, Manjón JV, *et al.* Automated Glioblastoma Segmentation Based on a Multiparametric Structured Unsupervised Classification. *PLOS ONE* 2015;**10**:e0125143. doi:10.1371/journal.pone.0125143

173 Jiang J, Liu T, Zhu W, *et al.* UBO Detector – A cluster-based, fully automated pipeline for extracting white matter hyperintensities. *NeuroImage* 2018;**174**:539–49. doi:10.1016/j.neuroimage.2018.03.050

174 Ji S, Ye C, Li F, *et al.* Automatic segmentation of white matter hyperintensities by an extended FitzHugh & Nagumo reaction diffusion model. *J Magn Reson Imaging* 2013;**37**:343–50. doi:10.1002/jmri.23836

175 Jeon S, Yoon U, Park J-S, *et al.* Fully automated pipeline for quantification and localization of white matter hyperintensity in brain magnetic resonance image. *International Journal of Imaging Systems and Technology* 2011;**21**:193–200. doi:https://doi.org/10.1002/ima.20277

176 Jansen JFA, Vlooswijk MCG, Majoie HM, *et al.* White matter lesions in patients with localization-related epilepsy. *Invest Radiol* 2008;**43**:552–8. doi:10.1097/RLI.0b013e31817e90d2

177 Jain S, Sima DM, Ribbens A, *et al.* Automatic segmentation and volumetry of multiple sclerosis brain lesions from MR images. *Neuroimage Clin* 2015;**8**:367–75. doi:10.1016/j.nicl.2015.05.003

178 Ithapu V, Singh V, Lindner C, *et al.* Extracting and summarizing white matter hyperintensities using supervised segmentation methods in Alzheimer's disease risk and aging studies. *Hum Brain Mapp* 2014;**35**:4219–35. doi:10.1002/hbm.22472

179 Islam A, Reza SMS, Iftekharuddin KM. Multifractal Texture Estimation for Detection and Segmentation of Brain Tumors. *IEEE Transactions on Biomedical Engineering* 2013;**60**:3204–15. doi:10.1109/TBME.2013.2271383

180 Iqbal S, Ghani MU, Saba T, *et al.* Brain tumor segmentation in multi-spectral MRI using convolutional neural networks (CNN). *Microsc Res Tech* 2018;**81**:419–27. doi:10.1002/jemt.22994

181 Ilunga–Mbuyamba E, Avina–Cervantes JG, Cepeda–Negrete J, *et al.* Automatic selection of localized region-based active contour models using image content analysis applied to brain tumor segmentation. *Computers in Biology and Medicine* 2017;**91**:69–79. doi:10.1016/j.compbiomed.2017.10.003

182 Ilunga-Mbuyamba E, Cruz-Duarte JM, Avina-Cervantes JG, *et al.* Active contours driven by Cuckoo Search strategy for brain tumour images segmentation. *Expert Systems with Applications* 2016;**56**:59–68. doi:10.1016/j.eswa.2016.02.048

183 Hussain S, Anwar SM, Majid M. Segmentation of glioma tumors in brain using deep convolutional neural network. *Neurocomputing* 2018;**282**:248–61. doi:10.1016/j.neucom.2017.12.032

184 Hulsey KM, Gupta M, King KS, *et al.* Automated quantification of white matter disease extent at 3 T: Comparison with volumetric readings. *Journal of Magnetic Resonance Imaging* 2012;**36**:305–11. doi:https://doi.org/10.1002/jmri.23659

185 Huang M, Yang W, Wu Y, *et al.* Brain Tumor Segmentation Based on Local Independent Projection-Based Classification. *IEEE Transactions on Biomedical Engineering* 2014;**61**:2633–45. doi:10.1109/TBME.2014.2325410

186 Herskovits E, Bryan R, Yang F. Automated Bayesian Segmentation of Microvascular White-Matter Lesions in the ACCORD-MIND Study. *Advances in Medical Sciences* 2008;**53**. doi:10.2478/v10039-008-0039-3

187 Havaei M, Davy A, Warde-Farley D, *et al.* Brain tumor segmentation with Deep Neural Networks. *Medical Image Analysis* 2017;**35**:18–31. doi:10.1016/j.media.2016.05.004

188 Harmouche R, Subbanna NK, Collins DL, *et al.* Probabilistic Multiple Sclerosis Lesion Classification Based on Modeling Regional Intensity Variability and Local Neighborhood Information. *IEEE Transactions on Biomedical Engineering* 2015;**62**:1281–92. doi:10.1109/TBME.2014.2385635

189 Harati V, Khayati R, Farzan A. Fully automated tumor segmentation based on improved fuzzy connectedness algorithm in brain MR images. *Computers in Biology and Medicine* 2011;**41**:483–92. doi:10.1016/j.compbiomed.2011.04.010

190 Hansen MB, Nagenthiraja K, Ribe LR, *et al.* Automated estimation of salvageable tissue: Comparison with expert readers. *Journal of Magnetic Resonance Imaging* 2016;**43**:220–8. doi:https://doi.org/10.1002/jmri.24963

191 Gupta N, Bhatele P, Khanna P. Identification of Gliomas from brain MRI through adaptive segmentation and run length of centralized patterns. *Journal of Computational Science* 2018;**25**:213–20. doi:10.1016/j.jocs.2017.02.009

192 Guo D, Fridriksson J, Fillmore P, *et al.* Automated lesion detection on MRI scans using combined unsupervised and supervised methods. *BMC Medical Imaging* 2015;**15**:50. doi:10.1186/s12880-015-0092-x

193 Guizard N, Coupé P, Fonov VS, *et al.* Rotation-invariant multi-contrast non-local means for MS lesion segmentation. *Neuroimage-Clinical* 2015;**8**:376–389. doi:10.1016/j.nicl.2015.05.001

194 Guerrero R, Qin C, Oktay O, *et al.* White matter hyperintensity and stroke lesion segmentation and differentiation using convolutional neural networks. *NeuroImage: Clinical* 2018;**17**:918–34. doi:10.1016/j.nicl.2017.12.022

195 Griffis JC, Allendorfer JB, Szaflarski JP. Voxel-based Gaussian naïve Bayes classification of ischemic stroke lesions in individual T1-weighted MRI scans. *J Neurosci Methods* 2016;**257**:97–108. doi:10.1016/j.jneumeth.2015.09.019

196 Griffanti L, Zamboni G, Khan A, *et al.* BIANCA (Brain Intensity AbNormality Classification Algorithm): A new tool for automated segmentation of white matter hyperintensities. *Neuroimage* 2016;**141**:191–205. doi:10.1016/j.neuroimage.2016.07.018

197 Gooya A, Pohl KM, Bilello M, *et al.* GLISTR: glioma image segmentation and registration. *IEEE Trans Med Imaging* 2012;**31**:1941–54. doi:10.1109/TMI.2012.2210558

198 Gonçalves N, Nikkilä J, Vigário R. Self-supervised MRI tissue segmentation by discriminative clustering. *Int J Neural Syst* 2014;**24**:1450004. doi:10.1142/S012906571450004X

199 Goetz M, Weber C, Binczyk F, *et al.* DALSA: Domain Adaptation for Supervised Learning From Sparsely Annotated MR Images. *IEEE Transactions on Medical Imaging* 2016;**35**:184–96. doi:10.1109/TMI.2015.2463078

200 Ghribi O, Sellami L, Slima MB, *et al.* Multiple sclerosis exploration based on automatic MRI modalities segmentation approach with advanced volumetric evaluations for essential feature extraction. *Biomedical Signal Processing and Control* 2018;**40**:473–87. doi:10.1016/j.bspc.2017.07.008

201 Ghribi O, Sellami L, Slima MB, *et al.* An Advanced MRI Multi-Modalities Segmentation Methodology Dedicated to Multiple Sclerosis Lesions Exploration and Differentiation. *IEEE Transactions on NanoBioscience* 2017;**16**:656–65. doi:10.1109/TNB.2017.2763246

202 Ghafoorian M, Karssemeijer N, van Uden IWM, *et al.* Automated detection of white matter hyperintensities of all sizes in cerebral small vessel disease. *Med Phys* 2016;**43**:6246. doi:10.1118/1.4966029

203 Ghafoorian M, Karssemeijer N, Heskes T, *et al.* Location Sensitive Deep Convolutional Neural Networks for Segmentation of White Matter Hyperintensities. *Scientific Reports* 2017;**7**:5110. doi:10.1038/s41598-017-05300-5

204 Ghafoorian M, Karssemeijer N, Heskes T, *et al.* Deep multi-scale location-aware 3D convolutional neural networks for automated detection of lacunes of presumed vascular origin. *NeuroImage: Clinical* 2017;**14**:391–9. doi:10.1016/j.nicl.2017.01.033

205 Geremia E, Clatz O, Menze BH, *et al.* Spatial decision forests for MS lesion segmentation in multi-channel magnetic resonance images. *NeuroImage* 2011;**57**:378–90. doi:10.1016/j.neuroimage.2011.03.080

206 García-Lorenzo D, Prima S, Arnold DL, *et al.* Trimmed-likelihood estimation for focal lesions and tissue segmentation in multisequence MRI for multiple sclerosis. *IEEE Trans Med Imaging* 2011;**30**:1455–67. doi:10.1109/TMI.2011.2114671

207 Gao J, Li C, Feng C, *et al.* Non-locally regularized segmentation of multiple sclerosis lesion from multi-channel MRI data. *Magn Reson Imaging* 2014;**32**:1058–66. doi:10.1016/j.mri.2014.03.006

208 Ganiler O, Oliver A, Diez Y, *et al.* A subtraction pipeline for automatic detection of new appearing multiple sclerosis lesions in longitudinal studies. *Neuroradiology* 2014;**56**:363–74. doi:10.1007/s00234-014-1343-1

209 Galimzianova A, Pernuš F, Likar B, *et al.* Stratified mixture modeling for segmentation of white-matter lesions in brain MR images. *NeuroImage* 2016;**124**:1031–43. doi:10.1016/j.neuroimage.2015.09.047

210 Galimzianova A, Lesjak Ž, Rubin DL, *et al.* Locally adaptive magnetic resonance intensity models for unsupervised segmentation of multiple sclerosis lesions. *J Med Imaging (Bellingham)* 2018;**5**. doi:10.1117/1.JMI.5.1.011007

211 Freire PGL, Ferrari RJ. Automatic iterative segmentation of multiple sclerosis lesions using Student's t mixture models and probabilistic anatomical atlases in FLAIR images. *Computers in Biology and Medicine* 2016;**73**:10–23. doi:10.1016/j.compbiomed.2016.03.025

212 Fiot J-B, Cohen LD, Raniga P, *et al.* Efficient brain lesion segmentation using multi-modality tissue-based feature selection and support vector machines. *Int J Numer Method Biomed Eng* 2013;**29**:905–15. doi:10.1002/cnm.2537

213 Fartaria MJ, Todea A, Kober T, *et al.* Partial volume-aware assessment of multiple sclerosis lesions. *Neuroimage Clin* 2018;**18**:245–53. doi:10.1016/j.nicl.2018.01.011

214 Fartaria MJ, Bonnier G, Roche A, *et al.* Automated detection of white matter and cortical lesions in early stages of multiple sclerosis. *Journal of Magnetic Resonance Imaging* 2016;**43**:1445–54. doi:https://doi.org/10.1002/jmri.25095

215 Essadike A, Ouabida E, Bouzid A. Brain tumor segmentation with Vander Lugt correlator based active contour. *Computer Methods and Programs in Biomedicine* 2018;**160**:103–17. doi:10.1016/j.cmpb.2018.04.004

216 Erus G, Zacharaki EI, Davatzikos C. Individualized statistical learning from medical image databases: application to identification of brain lesions. *Med Image Anal* 2014;**18**:542–54. doi:10.1016/j.media.2014.02.003

217 Emblem KE, Nedregaard B, Hald JK, *et al.* Automatic glioma characterization from dynamic susceptibility contrast imaging: brain tumor segmentation using knowledge-based fuzzy clustering. *J Magn Reson Imaging* 2009;**30**:1–10. doi:10.1002/jmri.21815

218 Elliott C, Arnold DL, Collins DL, *et al.* Temporally consistent probabilistic detection of new multiple sclerosis lesions in brain MRI. *IEEE Trans Med Imaging* 2013;**32**:1490–503. doi:10.1109/TMI.2013.2258403

219 Dyrby TB, Rostrup E, Baaré WFC, *et al.* Segmentation of age-related white matter changes in a clinical multi-center study. *NeuroImage* 2008;**41**:335–45. doi:10.1016/j.neuroimage.2008.02.024

220 Dvořák P, Bartusek K, Smékal Z. Unsupervised Pathological Area Extraction using 3D T2 and FLAIR MR Images. Published Online First: 2014. doi:10.2478/msr-2014-0049

For peer review only

221 Ding Y, Dong R, Lan T, *et al.* Multi-modal brain tumor image segmentation based on SDAE. *International Journal of Imaging Systems and Technology* 2018;**28**:38–47. doi:https://doi.org/10.1002/ima.22254

222 Dickson S, Thomas BT, Goddard P. Using neural networks to automatically detect brain tumours in MR images. *Int J Neural Syst* 1997;**8**:91–9. doi:10.1142/s0129065797000124

223 Demirhan A, Törü M, Güler İ. Segmentation of Tumor and Edema Along With Healthy Tissues of Brain Using Wavelets and Neural Networks. *IEEE Journal of Biomedical and Health Informatics* 2015;**19**:1451–8. doi:10.1109/JBHI.2014.2360515

224 de Sitter A, Steenwijk MD, Ruet A, *et al.* Performance of five research-domain automated WM lesion segmentation methods in a multi-center MS study. *Neuroimage* 2017;**163**:106–14. doi:10.1016/j.neuroimage.2017.09.011

225 de Boer R, Vrooman HA, van der Lijn F, *et al.* White matter lesion extension to automatic brain tissue segmentation on MRI. *NeuroImage* 2009;**45**:1151–61. doi:10.1016/j.neuroimage.2009.01.011

226 Datta S, Sajja BR, He R, *et al.* Segmentation of gadolinium-enhanced lesions on MRI in multiple sclerosis. *J Magn Reson Imaging* 2007;**25**:932–7. doi:10.1002/jmri.20896

227 Datta S, Narayana PA. A comprehensive approach to the segmentation of multichannel three-dimensional MR brain images in multiple sclerosis. *Neuroimage Clin* 2013;**2**:184–96. doi:10.1016/j.nicl.2012.12.007

228 Damangir S, Westman E, Simmons A, *et al.* Reproducible segmentation of white matter hyperintensities using a new statistical definition. *Magn Reson Mater Phy* 2017;**30**:227–37. doi:10.1007/s10334-016-0599-3

229 Damangir S, Manzouri A, Oppedal K, *et al.* Multispectral MRI segmentation of age related white matter changes using a cascade of support vector machines. *J Neurol Sci* 2012;**322**:211–6. doi:10.1016/j.jns.2012.07.064

230 Dadar M, Maranzano J, Misquitta K, *et al.* Performance comparison of 10 different classification techniques in segmenting white matter hyperintensities in aging. *Neuroimage* 2017;**157**:233–49. doi:10.1016/j.neuroimage.2017.06.009

231 Dadar M, Maranzano J, Ducharme S, *et al.* Validation of T1w-based segmentations of white matter hyperintensity volumes in large-scale datasets of aging. *Hum Brain Mapp* 2017;**39**:1093–107. doi:10.1002/hbm.23894

232 Dadar M, Pascoal TA, Manitsirikul S, *et al.* Validation of a Regression Technique for Segmentation of White Matter Hyperintensities in Alzheimer's Disease. *IEEE Transactions on Medical Imaging* 2017;**36**:1758–68. doi:10.1109/TMI.2017.2693978

233 Cui S, Mao L, Xiong S. Brain Tumor Automatic Segmentation Using Fully Convolutional Networks. *Journal of Medical Imaging and Health Informatics* 2017;**7**:1641–7. doi:10.1166/jmihi.2017.2179

234 Cui S, Mao L, Jiang J, *et al.* Automatic Semantic Segmentation of Brain Gliomas from MRI Images Using a Deep Cascaded Neural Network. *J Healthc Eng* 2018;**2018**. doi:10.1155/2018/4940593

235 Corso JJ, Sharon E, Dube S, *et al.* Efficient multilevel brain tumor segmentation with integrated bayesian model classification. *IEEE Trans Med Imaging* 2008;**27**:629–40. doi:10.1109/TMI.2007.912817

236 Cordier N, Delingette H, Ayache N. A Patch-Based Approach for the Segmentation of Pathologies: Application to Glioma Labelling. *IEEE Trans Med Imaging* 2016;**35**:1066–76. doi:10.1109/TMI.2015.2508150

For peer review only

237 Commowick O, Maarouf A, Ferré J-C, *et al.* Diffusion MRI abnormalities detection with orientation distribution functions: A multiple sclerosis longitudinal study. *Medical Image Analysis* 2015;**22**:114–23. doi:10.1016/j.media.2015.02.005

238 Colliot O, Mansi T, Bernasconi N, *et al.* Segmentation of focal cortical dysplasia lesions on MRI using level set evolution. *Neuroimage* 2006;**32**:1621–30. doi:10.1016/j.neuroimage.2006.04.225

239 Clarke LP, Velthuizen RP, Clark M, *et al.* MRI Measurement of Brain Tumor Response: Comparison of Visual Metric and Automatic Segmentation. *Magnetic Resonance Imaging* 1998;**16**:271–9. doi:10.1016/S0730-725X(97)00302-0

240 Cheriyan MM, Michael PA, Kumar A. Blind source separation with mixture models – A hybrid approach to MR brain classification. *Magnetic Resonance Imaging* 2018;**54**:137–47. doi:10.1016/j.mri.2018.08.023

241 Chen W, Liu B, Peng S, *et al.* Computer-Aided Grading of Gliomas Combining Automatic Segmentation and Radiomics. *International Journal of Biomedical Imaging* 2018;**2018**:e2512037. doi:https://doi.org/10.1155/2018/2512037

242 Chen L, Bentley P, Rueckert D. Fully automatic acute ischemic lesion segmentation in DWI using convolutional neural networks. *Neuroimage Clin* 2017;**15**:633–43. doi:10.1016/j.nicl.2017.06.016

243 Charron O, Lallement A, Jarnet D, *et al.* Automatic detection and segmentation of brain metastases on multimodal MR images with a deep convolutional neural network. *Computers in Biology and Medicine* 2018;**95**:43–54. doi:10.1016/j.compbiomed.2018.02.004

244 Cabria I, Gondra I. MRI segmentation fusion for brain tumor detection. *Information Fusion* 2017;**36**:1–9. doi:10.1016/j.inffus.2016.10.003

245 Cabezas M, Oliver A, Valverde S, *et al.* BOOST: A supervised approach for multiple sclerosis lesion segmentation. *Journal of Neuroscience Methods* 2014;**237**:108–17. doi:10.1016/j.jneumeth.2014.08.024

246 Cabezas M, Oliver A, Roura E, *et al.* Automatic multiple sclerosis lesion detection in brain MRI by FLAIR thresholding. *Computer Methods and Programs in Biomedicine* 2014;**115**:147–61. doi:10.1016/j.cmpb.2014.04.006

247 Cabezas M, Corral JF, Oliver A, *et al.* Improved Automatic Detection of New T2 Lesions in Multiple Sclerosis Using Deformation Fields. *American Journal of Neuroradiology* 2016;**37**:1816–23. doi:10.3174/ajnr.A4829

248 Brosch T, Tang LYW, Yoo Y, *et al.* Deep 3D Convolutional Encoder Networks With Shortcuts for Multiscale Feature Integration Applied to Multiple Sclerosis Lesion Segmentation. *IEEE Transactions on Medical Imaging* 2016;**35**:1229–39. doi:10.1109/TMI.2016.2528821

249 Bowles C, Qin C, Guerrero R, *et al.* Brain lesion segmentation through image synthesis and outlier detection. *NeuroImage: Clinical* 2017;**16**:643–58. doi:10.1016/j.nicl.2017.09.003

250 Boudraa AO, Dehak SM, Zhu YM, *et al.* Automated segmentation of multiple sclerosis lesions in multispectral MR imaging using fuzzy clustering. *Comput Biol Med* 2000;**30**:23–40. doi:10.1016/s0010-4825(99)00019-0

251 Bonte S, Goethals I, Van Holen R. Machine learning based brain tumour segmentation on limited data using local texture and abnormality. *Computers in Biology and Medicine* 2018;**98**:39–47. doi:10.1016/j.compbiomed.2018.05.005

For peer review only

252 Boldsen JK, Engedal TS, Pedraza S, *et al.* Better Diffusion Segmentation in Acute Ischemic Stroke Through Automatic Tree Learning Anomaly Segmentation. *Front Neuroinform* 2018;**12**. doi:10.3389/fninf.2018.00021

253 Binczyk F, Stjelties B, Weber C, *et al.* MiMSeg - an algorithm for automated detection of tumor tissue on NMR apparent diffusion coefficient maps. *Information Sciences* 2017;**384**:235–48. doi:10.1016/j.ins.2016.07.052

254 Binaghi E, Pedoia V, Balbi S. Meningioma and peritumoral edema segmentation of preoperative MRI brain scans. *Computer Methods in Biomechanics and Biomedical Engineering: Imaging & Visualization* 2018;**6**:362–70. doi:10.1080/21681163.2016.1250108

255 Bijar A, Khayati R, Benavent AP. Increasing the Contrast of the Brain MR FLAIR Images Using Fuzzy Membership Functions and Structural Similarity Indices in Order to Segment MS Lesions. *PLOS ONE* 2013;**8**:e65469. doi:10.1371/journal.pone.0065469

256 Bijar A, Khanloo MM, Benavent AP, *et al.* Segmentation of MS lesions using entropy-based EM algorithm and Markov random fields. *Journal of Biomedical Science and Engineering* 2011;**4**:552–61. doi:10.4236/jbise.2011.48071

257 Bhanu Prakash KN, Gupta V, Jianbo H, *et al.* Automatic processing of diffusion-weighted ischemic stroke images based on divergence measures: slice and hemisphere identification, and stroke region segmentation. *Int J CARS* 2008;**3**:559–70. doi:10.1007/s11548-008-0260-3

258 Beare R, Srikanth V, Chen J, *et al.* Development and validation of morphological segmentation of age-related cerebral white matter hyperintensities. *Neuroimage* 2009;**47**:199–203. doi:10.1016/j.neuroimage.2009.03.055

259 Banerjee S, Mitra S, Uma Shankar B. Automated 3D segmentation of brain tumor using visual saliency. *Information Sciences* 2018;**424**:337–53. doi:10.1016/j.ins.2017.10.011

260 Banerjee S, Mitra S, Shankar BU, *et al.* A Novel GBM Saliency Detection Model Using Multi-Channel MRI. *PLOS ONE* 2016;**11**:e0146388. doi:10.1371/journal.pone.0146388

261 Asman AJ, Chambless LB, Thompson RC, *et al.* Out-of-atlas likelihood estimation using multi-atlas segmentation. *Med Phys* 2013;**40**. doi:10.1118/1.4794478

262 Arakeri MeghaP, Reddy GRM. Computer-aided diagnosis system for tissue characterization of brain tumor on magnetic resonance images. *SIViP* 2015;**9**:409–25. doi:10.1007/s11760-013-0456-z

263 Anbeek P, Vincken KL, Viergever MA. Automated MS-Lesion Segmentation by K-Nearest Neighbor Classification. *The MIDAS Journal* 2008;:610.

264 Anbeek P, Vincken KL, van Osch MJP, *et al.* Automatic segmentation of different-sized white matter lesions by voxel probability estimation. *Medical Image Analysis* 2004;**8**:205–15. doi:10.1016/j.media.2004.06.019

265 Amiri S, Ali Mahjoub M, Rekik I. Tree-based Ensemble Classifier Learning for Automatic Brain Glioma Segmentation. *Neurocomputing* 2018;**313**:135–42. doi:10.1016/j.neucom.2018.05.112

266 Alex V, Vaidhya K, Thirunavukkarasu S, *et al.* Semisupervised learning using denoising autoencoders for brain lesion detection and segmentation. *J Med Imaging (Bellingham)* 2017;**4**:041311. doi:10.1117/1.JMI.4.4.041311

267 Alegro M de C, Amaro Junior E, Lopes R de D. Computerized brain tumor segmentation in magnetic resonance imaging. *Einstein (São Paulo)* 2012;**10**:158–63. doi:10.1590/S1679-45082012000200008

268 AlBadawy EA, Saha A, Mazurowski MA. Deep learning for segmentation of brain tumors: Impact of cross-institutional training and testing. *Medical Physics* 2018;**45**:1150–8. doi:https://doi.org/10.1002/mp.12752

269 Al-Shaikhli SDS, Yang MY, Rosenhahn B. Brain tumor classification and segmentation using sparse coding and dictionary learning. *Biomedical Engineering / Biomedizinische Technik* 2016;**61**:413–29. doi:10.1515/bmt-2015-0071

270 Akselrod-Ballin A, Galun M, Gomori JM, *et al.* Automatic segmentation and classification of multiple sclerosis in multichannel MRI. *IEEE Trans Biomed Eng* 2009;**56**:2461–9. doi:10.1109/TBME.2008.926671

271 Ahmed B, Brodley CE, Blackmon KE, *et al.* Cortical feature analysis and machine learning improves detection of "MRI-negative" focal cortical dysplasia. *Epilepsy Behav* 2015;**48**:21–8. doi:10.1016/j.yebeh.2015.04.055

272 Admiraal-Behloul F, van den Heuvel DMJ, Olofsen H, *et al.* Fully automatic segmentation of white matter hyperintensities in MR images of the elderly. *Neuroimage* 2005;**28**:607–17. doi:10.1016/j.neuroimage.2005.06.061

273 Adler S, Wagstyl K, Gunny R, *et al.* Novel surface features for automated detection of focal cortical dysplasias in paediatric epilepsy. *Neuroimage Clin* 2016;**14**:18–27. doi:10.1016/j.nicl.2016.12.030

274 Abdulraqeb AR, Al-Haidri WA, Sushkova LT, *et al.* An Automated Method for Segmenting Brain Tumors on MRI Images. *Biomed Eng* 2017;**51**:97–101. doi:10.1007/s10527-017-9692-9

275 Abdullah BA, Younis AA, John NM. Multi-Sectional Views Textural Based SVM for MS Lesion Segmentation in Multi-Channels MRIs. *Open Biomed Eng J* 2012;**6**:56–72. doi:10.2174/1874230001206010056

276 Abd-Ellah MK, Awad AI, Khalaf AAM, *et al.* Two-phase multi-model automatic brain tumour diagnosis system from magnetic resonance images using convolutional neural networks. *EURASIP Journal on Image and Video Processing* 2018;**2018**:97. doi:10.1186/s13640-018-0332-4

277 Abbasi S, Tajeripour F. Detection of brain tumor in 3D MRI images using local binary patterns and histogram orientation gradient. *Neurocomputing* 2017;**219**:526–35. doi:10.1016/j.neucom.2016.09.051

278 Agam G, Weiss D, Soman M, *et al.* Probabilistic Brain Lesion Segmentation in DT-MRI. In: *2006 International Conference on Image Processing*. 2006. 89–92. doi:10.1109/ICIP.2006.312369

279 Agn M, Law I, Munck Af Rosenschöld P, *et al.* A generative model for segmentation of tumor and organs-at-risk for radiation therapy planning of glioblastoma patients. In: *Progress in Biomedical Optics and Imaging - Proceedings of SPIE*. SPIE 2016. doi:10.1117/12.2216814

280 Agn M, Puonti O, Rosenschöld PM af, *et al.* Brain Tumor Segmentation Using a Generative Model with an RBM Prior on Tumor Shape. In: Crimi A, Menze B, Maier O, *et al.*, eds. *Brainlesion: Glioma, Multiple Sclerosis, Stroke and Traumatic Brain Injuries*. Cham: : Springer International Publishing 2016. 168–80. doi:10.1007/978-3-319-30858-6_15

281 Akter MK, Khan SM, Azad S, *et al.* Automated brain tumor segmentation from mri data based on exploration of histogram characteristics of the cancerous hemisphere. In: *2017 IEEE Region 10 Humanitarian Technology Conference (R10-HTC)*. 2017. 815–8. doi:10.1109/R10-HTC.2017.8289080

282 Al-Shaikhli SDS, Yang MY, Rosenhahn B. Coupled Dictionary Learning for Automatic Multi-Label Brain Tumor Segmentation in Flair MRI images. In: Bebis G, Boyle R, Parvin B, *et al.*, eds. *Advances in Visual Computing*. Cham: : Springer International Publishing 2014. 489–500. doi:10.1007/978-3-319-14249-4_46

283 Alex V, Safwan KPM, Chennamsetty SS, *et al.* Generative adversarial networks for brain lesion detection. In: *Medical Imaging 2017: Image Processing*. 2017. 101330G. doi:10.1117/12.2254487

284 Amiri S, Rekik I, Mahjoub MA. Deep random forest-based learning transfer to SVM for brain tumor segmentation. In: *2016 2nd International Conference on Advanced Technologies for Signal and Image Processing (ATSIP)*. 2016. 297–302. doi:10.1109/ATSIP.2016.7523095

285 Amiri S, Mahjoub M, Rekik I. Bayesian Network and Structured Random Forest Cooperative Deep Learning For Automatic Multi-label Brain Tumor Segmentation. In: *ICAART*. 2018. 183–90.

286 Ananda RS, Thomas T. Automatic segmentation framework for primary tumors from brain MRIs using morphological filtering techniques. In: *2012 5th International Conference on BioMedical Engineering and Informatics*. 2012. 238–42. doi:10.1109/BMEI.2012.6512995

287 Anwar SM, Yousaf S, Majid M. Brain tumor segmentation on Multimodal MRI scans using EMAP Algorithm. In: *2018 40th Annual International Conference of the IEEE Engineering in Medicine and Biology Society (EMBC)*. 2018. 550–3. doi:10.1109/EMBC.2018.8512304

288 Assaf Hoogi, Andrew Lee, Vivek Bharadwa, *et al.* Multimodal Brain Tumor Segmentation (BRATS) using Sparse Coding and 2-layer Neural Network. In: *Proceedings of the Multimodal Brain Tumor Image Segmentation Challenge*. 2015. 34.

289 Baid U, Talbar S, Talbar SN. Novel approach for brain tumor segmentation with non negative matrix factorization. In: *2017 International Conference on Innovations in Electronics, Signal Processing and Communication (IESC)*. 2017. 101–5. doi:10.1109/IESPC.2017.8071873

290 Bauer S, Gratz PP, Gralla J, *et al.* Towards automatic MRI volumetry for treatment selection in acute ischemic stroke patients. In: *Annual International Conference of the IEEE Engineering in Medicine and Biology Society.* 2014. 1521–4. doi:10.1109/EMBC.2014.6943891

291 Bauer S, Nolte L-P, Reyes M. Fully Automatic Segmentation of Brain Tumor Images Using Support Vector Machine Classification in Combination with Hierarchical Conditional Random Field Regularization. In: Fichtinger G, Martel A, Peters T, eds. *Medical Image Computing and Computer-Assisted Intervention – MICCAI 2011*. Berlin, Heidelberg: : Springer 2011. 354–61. doi:10.1007/978-3-642-23626-6_44

292 Behzadfar N, Soltanian-Zadeh H. Automatic segmentation of brain tumors in magnetic resonance images. In: *Proceedings of 2012 IEEE-EMBS International Conference on Biomedical and Health Informatics*. 2012. 329–32. doi:10.1109/BHI.2012.6211580

293 Ben Salah M, Diaz I, Greiner R, *et al.* Fully Automated Brain Tumor Segmentation Using Two MRI Modalities. In: Bebis G, Boyle R, Parvin B, *et al.*, eds. *Advances in Visual Computing*. Berlin, Heidelberg: : Springer 2013. 30–9. doi:10.1007/978-3-642-41914-0_4

294 Benson CC, Deepa V, Lajish VL, *et al.* Brain tumor segmentation from MR brain images using improved fuzzy c-means clustering and watershed algorithm. In: *2016 International Conference on Advances in Computing, Communications and Informatics (ICACCI)*. 2016. 187–92. doi:10.1109/ICACCI.2016.7732045

295 Bento M, de Souza R, Lotufo R, *et al.* WMH Segmentation Challenge: A Texture-Based Classification Approach. In: Crimi A, Bakas S, Kuijf H, *et al.*, eds. *Brainlesion: Glioma, Multiple Sclerosis, Stroke and Traumatic Brain Injuries*. Cham: : Springer International Publishing 2018. 489–500. doi:10.1007/978-3-319-75238-9_41

For peer review only

296 Bento M, Sym Y, Frayne R, *et al.* Probabilistic Segmentation of Brain White Matter Lesions Using Texture-Based Classification. In: Karray F, Campilho A, Cheriet F, eds. *Image Analysis and Recognition*. Cham: : Springer International Publishing 2017. 71–8. doi:10.1007/978-3-319-59876-5_9

297 Bharath HN, Colleman S, Sima DM, *et al.* Tumor Segmentation from Multimodal MRI Using Random Forest with Superpixel and Tensor Based Feature Extraction. In: Crimi A, Bakas S, Kuijf H, *et al.*, eds. *Brainlesion: Glioma, Multiple Sclerosis, Stroke and Traumatic Brain Injuries*. Cham: : Springer International Publishing 2018. 463–73. doi:10.1007/978-3-319-75238-9_39

298 Bhattacharya D, Sinha N. An improved approach of high graded glioma segmentation using sparse autoencoder and fuzzy c-means clustering from multi-modal MR images. In: *Medical Imaging 2018: Biomedical Applications in Molecular, Structural, and Functional Imaging*. International Society for Optics and Photonics 2018. 1057813. doi:10.1117/12.2293044

299 Bianchi A, Miller JV, Tan ET, *et al.* Brain tumor segmentation with symmetric texture and symmetric intensity-based decision forests. In: *Proceedings / IEEE International Symposium on Biomedical Imaging: from nano to macro.* 2013. 748–51. doi:10.1109/ISBI.2013.6556583

300 Bijar A, Khayati R. Segmentation of MS lesions using Active Contour Model, Adaptive Mixtures Method and MRF model. In: *2011 7th International Symposium on Image and Signal Processing and Analysis (ISPA)*. 2011. 159–64.

301 Bilotta E, Cerasa A, Pantano P, *et al.* A CNN Based Algorithm for the Automated Segmentation of Multiple Sclerosis Lesions. In: Di Chio C, Cagnoni S, Cotta C, *et al.*, eds. *Applications of Evolutionary Computation*. Berlin, Heidelberg: : Springer 2010. 211–20. doi:10.1007/978-3-642-12239-2_22

302 Birenbaum A, Greenspan H. Longitudinal Multiple Sclerosis Lesion Segmentation Using Multi-view Convolutional Neural Networks. In: Carneiro G, Mateus D, Peter L, *et al.*, eds. *Deep Learning and Data Labeling for Medical Applications*. Cham: : Springer International Publishing 2016. 58–67. doi:10.1007/978-3-319-46976-8_7

303 Bougacha A, Boughariou J, Slima MB, *et al.* Comparative study of supervised and unsupervised classification methods: Application to automatic MRI glioma brain tumors segmentation. In: *2018 4th International Conference on Advanced Technologies for Signal and Image Processing (ATSIP)*. 2018. 1–5. doi:10.1109/ATSIP.2018.8364463

304 Boussaid H, Kokkinos I, Paragios N. Rapid Mode Estimation for 3D Brain MRI Tumor Segmentation. In: Heyden A, Kahl F, Olsson C, *et al.*, eds. *Energy Minimization Methods in Computer Vision and Pattern Recognition*. Berlin, Heidelberg: : Springer 2013. 1–11. doi:10.1007/978-3-642-40395-8_1

305 Cabezas M, Oliver A, Freixenet J, *et al.* A Supervised Approach for Multiple Sclerosis Lesion Segmentation Using Context Features and an Outlier Map. In: Sanches JM, Micó L, Cardoso JS, eds. *Pattern Recognition and Image Analysis*. Berlin, Heidelberg: : Springer 2013. 782–9. doi:10.1007/978-3-642-38628-2_93

306 Cai H, Verma R, Ou Y, *et al.* Probabilistic segmentation of brain tumours based on multimodality magnetic resonance images. In: *2007 4th IEEE International Symposium on Biomedical Imaging: From Nano to Macro*. 2007. 600–3. doi:10.1109/ISBI.2007.356923

307 Chang P. Fully convolutional neural networks with hyperlocal features for brain tumor segmentation. 2016. 118. doi:10.1007/978-3-319-55524-9_11

308 Chen L, Wu Y, DSouza AM, *et al.* MRI tumor segmentation with densely connected 3D CNN. In: *Medical Imaging 2018: Image Processing*. International Society for Optics and Photonics 2018. 105741F. doi:10.1117/12.2293394

309 Chen W, Qiao X, Liu B, *et al.* Automatic brain tumor segmentation based on features of separated local square. In: *2017 Chinese Automation Congress (CAC)*. 2017. 6489–93. doi:10.1109/CAC.2017.8243946

310 Corso JJ, Sharon E, Yuille A. Multilevel Segmentation and Integrated Bayesian Model Classification with an Application to Brain Tumor Segmentation. In: Larsen R, Nielsen M, Sporring J, eds. *Medical Image Computing and Computer-Assisted Intervention – MICCAI 2006*. Berlin, Heidelberg: : Springer 2006. 790–8. doi:10.1007/11866763_97

311 Corso JJ, Yuille A, Sicotte NL, *et al.* Detection and segmentation of pathological structures by the extended graph-shifts algorithm. In: *International Conference on Medical Image Computing and Computer-Assisted Intervention*. 2007. 985–93. doi:10.1007/978-3-540-75757-3_119

312 Dalca AV, Sridharan R, Cloonan L, *et al.* Segmentation of Cerebrovascular Pathologies in Stroke Patients with Spatial and Shape Priors. In: *International Conference on Medical Image Computing and Computer-Assisted Intervention*. 2014. 773–80.https://www.ncbi.nlm.nih.gov/pmc/articles/PMC4260817/ (accessed 15 Nov 2020).

313 Derntl A, Plant C, Gruber P, *et al.* Stroke Lesion Segmentation Using a Probabilistic Atlas of Cerebral Vascular Territories. In: Crimi A, Menze B, Maier O, *et al.*, eds. *Brainlesion: Glioma, Multiple Sclerosis, Stroke and Traumatic Brain Injuries*. Cham: : Springer International Publishing 2016. 21–32. doi:10.1007/978-3-319-30858-6_3

314 Deshpande H, Maurel P, Barillot C. Adaptive dictionary learning for competitive classification of multiple sclerosis lesions. In: *2015 IEEE 12th International Symposium on Biomedical Imaging (ISBI)*. 2015. 136–9. doi:10.1109/ISBI.2015.7163834

315 Diaz I, Boulanger P, Greiner R, *et al.* An automatic brain tumor segmentation tool. In: *Annual International Conference of the IEEE Engineering in Medicine and Biology Society.* 2013. 3339–42. doi:10.1109/EMBC.2013.6610256

316 Dong H, Yang G, Liu F, *et al.* Automatic Brain Tumor Detection and Segmentation Using U-Net Based Fully Convolutional Networks. In: Valdés Hernández M, González-Castro V, eds. *Medical Image Understanding and Analysis*. Cham: : Springer International Publishing 2017. 506–17. doi:10.1007/978-3-319-60964-5_44

317 Doyle S, Forbes F, Jaillard A, *et al.* Sub-acute and Chronic Ischemic Stroke Lesion MRI Segmentation. In: Crimi A, Bakas S, Kuijf H, *et al.*, eds. *Brainlesion: Glioma, Multiple Sclerosis, Stroke and Traumatic Brain Injuries*. Cham: : Springer International Publishing 2018. 111–22. doi:10.1007/978-3-319-75238-9_10

318 Dvorak P, Bartusek K, Gescheidtova E. Automatic extraction of pathological area in 2D MR brain scan. In: *PIERS Proceedings*. 2014. 1885–9.

319 Dvořák P, Menze B. Local Structure Prediction with Convolutional Neural Networks for Multimodal Brain Tumor Segmentation. In: Menze B, Langs G, Montillo A, *et al.*, eds. *Medical Computer Vision: Algorithms for Big Data*. Cham: : Springer International Publishing 2016. 59–71. doi:10.1007/978-3-319-42016-5_6

320 El-Khamy SE, Sadek RA, El-Khoreby MA. An efficient brain mass detection with adaptive clustered based fuzzy C-mean and thresholding. In: *2015 IEEE International Conference on Signal and Image Processing Applications (ICSIPA)*. 2015. 429–33. doi:10.1109/ICSIPA.2015.7412229

321 Elliott C, Francis SJ, Arnold DL, *et al.* Bayesian Classification of Multiple Sclerosis Lesions in Longitudinal MRI Using Subtraction Images. In: Jiang T, Navab N, Pluim JPW, *et al.*, eds. *Medical Image Computing and Computer-Assisted Intervention – MICCAI 2010*. Berlin, Heidelberg: : Springer 2010. 290–7. doi:10.1007/978-3-642-15745-5_36

322 Ellwaa, A., Hussein, A., AlNaggar, E., *et al.* Brain Tumor Segmantation Using Random Forest Trained on Iteratively Selected Patients. 2016. 129–37.https://www.springerprofessional.de/en/brain-tumor-segmantation-using-random-forest-trained-on-iterativ/12216552 (accessed 16 Nov 2020).

323 Feng C, Zhao D, Huang M. Segmentation of Ischemic Stroke Lesions in Multi-spectral MR Images Using Weighting Suppressed FCM and Three Phase Level Set. In: Crimi A, Menze B, Maier O, *et al.*, eds. *Brainlesion: Glioma, Multiple Sclerosis, Stroke and Traumatic Brain Injuries*. Cham: : Springer International Publishing 2016. 233–45. doi:10.1007/978-3-319-30858-6_20

324 Ferrari RJ, M.d XW, M.d YZ, *et al.* Segmentation of multiple sclerosis lesions using support vector machines. In: *Medical Imaging 2003: Image Processing*. International Society for Optics and Photonics 2003. 16–26. doi:10.1117/12.481377

325 Festa J, Pereira S, Mariz J, *et al.* Automatic brain tumor segmentation of multi-sequence mr images using random decision forests. In: *Proceedings of the MICCAI grand challenge on MR brain image segmentation (MRBrainS'13)*. 2013.

326 Fiçici CÖ, Eroğul O, Telatar Z. Fully Automated Brain Tumor Segmentation and Volume Estimation Based on Symmetry Analysis in MR Images. In: Badnjevic A, ed. *CMBEBIH 2017*. Singapore: : Springer 2017. 53–60. doi:10.1007/978-981-10-4166-2_9

327 Folgoc LL, Nori AV, Alvarez-Valle J, *et al.* Segmentation of brain tumors via cascades of lifted decision forests. In: *Proceedings MICCAI-BRATS Workshop*. 2016.

328 Folgoc L, Nori A, Ancha S, *et al.* Lifted Auto-Context Forests for Brain Tumour Segmentation. 2016. 171–83. doi:10.1007/978-3-319-55524-9_17

329 García-Lorenzo D, Lecoeur J, Arnold DL, *et al.* Multiple Sclerosis Lesion Segmentation Using an Automatic Multimodal Graph Cuts. In: Yang G-Z, Hawkes D, Rueckert D, *et al.*, eds. *Medical Image Computing and Computer-Assisted Intervention – MICCAI 2009*. Berlin, Heidelberg: : Springer 2009. 584–91. doi:10.1007/978-3-642-04271-3_71

330 García-Lorenzo D, Prima S, Morrissey S, *et al.* A robust Expectation-Maximization algorithm for Multiple Sclerosis lesion segmentation. In: *Proceeding of MICCAI Workshop*. 2008.

331 Geremia E, Menze BH, Ayache N. Spatial Decision Forests for Glioma Segmentation in Multi-Channel MR Images. 2012. https://hal.inria.fr/hal-00813827 (accessed 15 Nov 2020).

332 Goel S, Sehgal A, Mangipudi P, *et al.* Brain tumor segmentation in multispectral MR images. In: *2017 4th International Conference on Signal Processing and Integrated Networks (SPIN)*. 2017. 1–4. doi:10.1109/SPIN.2017.8049904

333 Gondra I, Cabria I. Automated segmentation of brain tumors in MRI using potential field clustering. In: *IEEE EUROCON 2015 - International Conference on Computer as a Tool (EUROCON)*. 2015. 1–6. doi:10.1109/EUROCON.2015.7313670

334 Götz M, Weber C, Blöcher J, *et al.* Extremely randomized trees based brain tumor segmentation. In: *Proceeding of BRATS challenge-MICCAI*. 2014. 006–11.

335 Hadjiprocopis A, Tofts P. An automatic lesion segmentation method for fast spin echo magnetic resonance images using an ensemble of neural networks. In: *2003 IEEE XIII Workshop on Neural Networks for Signal Processing (IEEE Cat. No.03TH8718)*. 2003. 709–18. doi:10.1109/NNSP.2003.1318070

336 Haeck T, Maes F, Suetens P. Automated Model-Based Segmentation of Brain Tumors in MR Images. In: *Proceedings BraTS Challenge 2015*. 2015. 25–8.

337 Haeck T, Maes F, Suetens P. ISLES Challenge 2015: Automated Model-Based Segmentation of Ischemic Stroke in MR Images. In: Crimi A, Menze B, Maier O, *et al.*, eds. *Brainlesion: Glioma, Multiple Sclerosis, Stroke and Traumatic Brain Injuries*. Cham: : Springer International Publishing 2016. 246–53. doi:10.1007/978-3-319-30858-6_21

338 Harmouche R, Collins L, Arnold D, *et al.* Bayesian MS Lesion Classification Modeling Regional and Local Spatial Information. In: *18th International Conference on Pattern Recognition (ICPR'06)*. 2006. 984–7. doi:10.1109/ICPR.2006.318

339 Havaei M, Dutil F, Pal C, *et al.* A Convolutional Neural Network Approach to Brain Tumor Segmentation. In: Crimi A, Menze B, Maier O, *et al.*, eds. *Brainlesion: Glioma, Multiple Sclerosis, Stroke and Traumatic Brain Injuries*. Cham: : Springer International Publishing 2016. 195–208. doi:10.1007/978-3-319-30858-6_17

340 Hevia-Montiel N, Jiménez-Alaniz JR, Medina-Bañuelos V, *et al.* Robust nonparametric segmentation of infarct lesion from diffusion-weighted MR images. In: *Annual International Conference of the IEEE Engineering in Medicine and Biology Society.* 2007. 2102–5. doi:10.1109/IEMBS.2007.4352736

341 Hooda H, Verma OP, Singhal T. Brain tumor segmentation: A performance analysis using K-Means, Fuzzy C-Means and Region growing algorithm. In: *2014 IEEE International Conference on Advanced Communications, Control and Computing Technologies*. 2014. 1621–6. doi:10.1109/ICACCCT.2014.7019383

342 Hu Y, Xia Y. 3D Deep Neural Network-Based Brain Tumor Segmentation Using Multimodality Magnetic Resonance Sequences. In: Crimi A, Bakas S, Kuijf H, *et al.*, eds. *Brainlesion: Glioma, Multiple Sclerosis, Stroke and Traumatic Brain Injuries*. Cham: : Springer International Publishing 2018. 423–34. doi:10.1007/978-3-319-75238-9_36

343 Huang J, Yang F, Chen W. Brain Tumor Segmentation Based on Texture, Intensity, and Edge. In: Long M, ed. *World Congress on Medical Physics and Biomedical Engineering May 26-31, 2012, Beijing, China*. Berlin, Heidelberg: : Springer 2013. 991–4. doi:10.1007/978-3-642-29305-4_260

344 Hussain S, Anwar SM, Majid M. Brain tumor segmentation using cascaded deep convolutional neural network. In: *2017 39th Annual International Conference of the IEEE Engineering in Medicine and Biology Society (EMBC)*. 2017. 1998–2001. doi:10.1109/EMBC.2017.8037243

345 Iftekharuddin KM, Islam MA, Shaik J, *et al.* Automatic brain tumor detection in MRI: methodology and statistical validation. In: *Medical Imaging 2005: Image Processing*. International Society for Optics and Photonics 2005. 2012–22. doi:10.1117/12.595931

346 Ilunga-Mbuyamba E, Avina-Cervantes JG, Lindner D, *et al.* Automatic brain tumor tissue detection based on hierarchical centroid shape descriptor in Tl-weighted MR images. In: *2016 International Conference on Electronics, Communications and Computers (CONIELECOMP)*. 2016. 62–7. doi:10.1109/CONIELECOMP.2016.7438553

347 Jain S, Ribbens A, Sima DM, *et al.* Unsupervised Framework for Consistent Longitudinal MS Lesion Segmentation. In: Müller H, Kelm BM, Arbel T, *et al.*, eds. *Medical Computer Vision and Bayesian and Graphical Models for Biomedical Imaging*. Cham: : Springer International Publishing 2017. 208–19. doi:10.1007/978-3-319-61188-4_19

348 Jerman T, Galimzianova A, Pernuš F, *et al.* Combining Unsupervised and Supervised Methods for Lesion Segmentation. In: Crimi A, Menze B, Maier O, *et al.*, eds. *Brainlesion: Glioma, Multiple Sclerosis, Stroke*

and Traumatic Brain Injuries. Cham: : Springer International Publishing 2016. 45–56. doi:10.1007/978-3-319-30858-6_5

349 Jesson A, Arbel T. Hierarchical MRF and random forest segmentation of MS lesions and healthy tissue in brain MRI. In: *Proceedings of the 2015 longitudinal multiple sclerosis lesion segmentation challenge*. 2015. 3.

350 Jiang Y, Hou J, Xiao X, *et al.* A Brain Tumor Segmentation New Method Based on Statistical Thresholding and Multiscale CNN. In: Huang D-S, Gromiha MM, Han K, *et al.*, eds. *Intelligent Computing Methodologies*. Cham: : Springer International Publishing 2018. 235–45. doi:10.1007/978-3-319-95957-3_26

351 Jin D, Xu Z, Harrison AP, *et al.* White matter hyperintensity segmentation from T1 and FLAIR images using fully convolutional neural networks enhanced with residual connections. In: *2018 IEEE 15th International Symposium on Biomedical Imaging (ISBI 2018)*. 2018. 1060–4. doi:10.1109/ISBI.2018.8363754

352 Jog A, Carass A, Pham DL, *et al.* Multi-Output Decision Trees for Lesion Segmentation in Multiple Sclerosis. In: *Proceedings of SPIE the International Society for Optical Engineering*. 2015. doi:10.1117/12.2082157

353 Kanas VG, Zacharaki EI, Dermatas E, *et al.* Combining Outlier Detection with Random Walker for Automatic Brain Tumor Segmentation. In: Iliadis L, Maglogiannis I, Papadopoulos H, *et al.*, eds. *Artificial Intelligence Applications and Innovations*. Berlin, Heidelberg: : Springer 2012. 26–35. doi:10.1007/978-3-642-33412-2_3

354 Kapás Z, Lefkovits L, Iclănzan D, *et al.* Automatic Brain Tumor Segmentation in Multispectral MRI Volumes Using a Random Forest Approach. In: Paul M, Hitoshi C, Huang Q, eds. *Image and Video Technology*. Cham: : Springer International Publishing 2018. 137–49. doi:10.1007/978-3-319-75786-5_12

355 Karimaghaloo Z, Arnold DL, Collins DL, *et al.* Hierarchical Conditional Random Fields for Detection of Gad-Enhancing Lesions in Multiple Sclerosis. In: Ayache N, Delingette H, Golland P, *et al.*, eds. *Medical Image Computing and Computer-Assisted Intervention – MICCAI 2012*. Berlin, Heidelberg: : Springer 2012. 379–86. doi:10.1007/978-3-642-33418-4_47

356 Karimaghaloo Z, Rivaz H, Arnold DL, *et al.* Adaptive Voxel, Texture and Temporal Conditional Random Fields for Detection of Gad-Enhancing Multiple Sclerosis Lesions in Brain MRI. In: Mori K, Sakuma I, Sato Y, *et al.*, eds. *Medical Image Computing and Computer-Assisted Intervention – MICCAI 2013*. Berlin, Heidelberg: : Springer 2013. 543–50. doi:10.1007/978-3-642-40760-4_68

357 Karpate Y, Commowick O, Barillot C. Probabilistic one class learning for automatic detection of multiple sclerosis lesions. In: *2015 IEEE 12th International Symposium on Biomedical Imaging (ISBI)*. 2015. 486–9. doi:10.1109/ISBI.2015.7163917

358 Khademi A, Moody AR. Multiscale partial volume estimation for segmentation of white matter lesions using flair MRI. In: *2015 IEEE 12th International Symposium on Biomedical Imaging (ISBI)*. 2015. 568–71. doi:10.1109/ISBI.2015.7163937

359 Khotanlou H, Colliot O, Bloch I. Automatic Brain Tumor Segmentation Using Symmetry Analysis and Deformable Models. In: *Advances in Pattern Recognition*. WORLD SCIENTIFIC 2006. 198–202. doi:10.1142/9789812772381_0032

360 Knight J, Khademi A. MS Lesion Segmentation Using FLAIR MRI Only. In: *Proceedings of the 1st MICCAI Challenge on Multiple Sclerosis Lesions Segmentation Challenge Using a Data Management and Processing Infrastructure-MICCAI-MSSEG*. 2016. 21–8.

361 Kroon D-J, van Oort E, Slump K. Multiple Sclerosis Detection in Multispectral Magnetic Resonance Images with Principal Components Analysis. In: *Grand Challenge Work.: Mult. Scler. Lesion Segm. Challenge*. 2008. 1–14.http://hdl.handle.net/10380/1441 (accessed 15 Nov 2020).

362 Kuijf HJ, Tax CMW, Zaanen LK, *et al.* The Added Value of Diffusion Tensor Imaging for Automated White Matter Hyperintensity Segmentation. In: O'Donnell L, Nedjati-Gilani G, Rathi Y, *et al.*, eds. *Computational Diffusion MRI*. Cham: : Springer International Publishing 2014. 45–53. doi:10.1007/978-3-319-11182-7_5

363 Kumar SVA, Harish BS, Aradhya VNM. A picture fuzzy clustering approach for brain tumor segmentation. In: *2016 Second International Conference on Cognitive Computing and Information Processing (CCIP)*. 2016. 1–6. doi:10.1109/CCIP.2016.7802852

364 Kwon D, Akbari H, Da X, *et al.* Multimodal Brain Tumor Image Segmentation Using GLISTR. In: *MICCAI brain tumor segmentation (BraTS) challenge manuscripts*. 2014.

365 Latif G, Butt MM, Khan AH, *et al.* Automatic Multimodal Brain Image Classification Using MLP and 3D Glioma Tumor Reconstruction. In: *2017 9th IEEE-GCC Conference and Exhibition (GCCCE)*. 2017. 1–9. doi:10.1109/IEEEGCC.2017.8448135

366 Lefkovits L, Lefkovits S, Szilágyi L. Brain Tumor Segmentation with Optimized Random Forest. In: Crimi A, Menze B, Maier O, *et al.*, eds. *Brainlesion: Glioma, Multiple Sclerosis, Stroke and Traumatic Brain Injuries*. Cham: : Springer International Publishing 2016. 88–99. doi:10.1007/978-3-319-55524-9_9

367 Li W, Tian J. Automatic segmentation of brain infarction in diffusion-weighted MR images. In: *Medical Imaging 2003: Image Processing*. International Society for Optics and Photonics 2003. 1531–42. doi:10.1117/12.481350

368 Li W, Tian J, Dai J. Automatic segmentation of cerebral ischemic lesions from diffusion tensor MR images. In: *Medical Imaging 2004: Image Processing*. International Society for Optics and Photonics 2004. 1640–9. doi:10.1117/12.536007

369 Li Y, Dou Q, Yu J, *et al.* Automatic Brain Tumor Segmentation from MR Images via a Multimodal Sparse Coding Based Probabilistic Model. In: *2015 International Workshop on Pattern Recognition in NeuroImaging*. 2015. 41–4. doi:10.1109/PRNI.2015.18

370 Liu J, Smith CD, Chebrolu H. Automatic Multiple Sclerosis detection based on integrated square estimation. In: *2009 IEEE Computer Society Conference on Computer Vision and Pattern Recognition Workshops*. 2009. 31–8. doi:10.1109/CVPRW.2009.5204351

371 Liu R, Cheng J, Zhu X, *et al.* Multi-modal Brain Tumor Segmentation Based on Self-organizing Active Contour Model. In: Tan T, Li X, Chen X, *et al.*, eds. *Pattern Recognition*. Singapore: : Springer 2016. 486–98. doi:10.1007/978-981-10-3005-5_40

372 López-Zorrilla A, de Velasco-Vázquez M, Serradilla-Casado O, *et al.* Brain White Matter Lesion Segmentation with 2D/3D CNN. In: Ferrández Vicente JM, Álvarez-Sánchez JR, de la Paz López F, *et al.*, eds. *Natural and Artificial Computation for Biomedicine and Neuroscience*. Cham: : Springer International Publishing 2017. 394–403. doi:10.1007/978-3-319-59740-9_39

373 Lyksborg M, Puonti O, Agn M, *et al.* An Ensemble of 2D Convolutional Neural Networks for Tumor Segmentation. In: Paulsen RR, Pedersen KS, eds. *Image Analysis*. Cham: : Springer International Publishing 2015. 201–11. doi:10.1007/978-3-319-19665-7_17

374 Mahmood Q, Basit A. Automatic Ischemic Stroke Lesion Segmentation in Multi-spectral MRI Images Using Random Forests Classifier. In: Crimi A, Menze B, Maier O, *et al.*, eds. *Brainlesion: Glioma, Multiple*

Sclerosis, Stroke and Traumatic Brain Injuries. Cham: : Springer International Publishing 2016. 266–74. doi:10.1007/978-3-319-30858-6_23

375 Mahmood Q, Basit A. Prediction of Ischemic Stroke Lesion and Clinical Outcome in Multi-modal MRI Images Using Random Forests. In: Crimi A, Menze B, Maier O, *et al.*, eds. *Brainlesion: Glioma, Multiple Sclerosis, Stroke and Traumatic Brain Injuries*. Cham: : Springer International Publishing 2016. 244–55. doi:10.1007/978-3-319-55524-9_23

376 Maier O, Wilms M, Handels H. Image Features for Brain Lesion Segmentation Using Random Forests. In: Crimi A, Menze B, Maier O, *et al.*, eds. *Proc. 2015 Longitudinal Multiple Sclerosis Lesion Segmentation Challenge*. Cham: : Springer International Publishing 2015. 119–30. doi:10.1007/978-3-319-30858-6_11

377 McKinley R, Häni L, Wiest R, *et al.* Segmenting the Ischemic Penumbra: A Decision Forest Approach with Automatic Threshold Finding. In: Crimi A, Menze B, Maier O, *et al.*, eds. *Brainlesion: Glioma, Multiple Sclerosis, Stroke and Traumatic Brain Injuries*. Cham: : Springer International Publishing 2015. 275–83. doi:10.1007/978-3-319-30858-6_24

378 Mechrez R, Goldberger J, Greenspan H. MS lesion segmentation using a multi-channel patch-based approach with spatial consistency. In: *Medical Imaging 2015: Image Processing*. International Society for Optics and Photonics 2015. 94130O. doi:10.1117/12.2082558

379 Mehmood I, Baik R, Baik SW. Automatic Segmentation of Region of Interests in MR Images Using Saliency Information and Active Contours. In: Kim KJ, Chung K-Y, eds. *IT Convergence and Security 2012*. Dordrecht: : Springer Netherlands 2012. 537–44. doi:10.1007/978-94-007-5860-5_64

380 Meier R, Karamitsou V, Habegger S, *et al.* Parameter Learning for CRF-Based Tissue Segmentation of Brain Tumors. In: Crimi A, Menze B, Maier O, *et al.*, eds. *Brainlesion: Glioma, Multiple Sclerosis, Stroke and Traumatic Brain Injuries*. Cham: : Springer International Publishing 2016. 156–67. doi:10.1007/978-3-319-30858-6_14

381 Meier R, Knecht U, Wiest R, *et al.* CRF-Based Brain Tumor Segmentation: Alleviating the Shrinking Bias. In: Crimi A, Menze B, Maier O, *et al.*, eds. *Brainlesion: Glioma, Multiple Sclerosis, Stroke and Traumatic Brain Injuries*. Cham: : Springer International Publishing 2016. 100–7. doi:10.1007/978-3-319-55524-9_10

382 Mengqiao W, Jie Y, Yilei C, *et al.* The multimodal brain tumor image segmentation based on convolutional neural networks. In: *2017 2nd IEEE International Conference on Computational Intelligence and Applications (ICCIA)*. 2017. 336–9. doi:10.1109/CIAPP.2017.8167234

383 Mitra J, Bourgeat P, Fripp J, *et al.* Classification Forests and Markov Random Field to Segment Chronic Ischemic Infarcts from Multimodal MRI. In: Shen L, Liu T, Yap P-T, *et al.*, eds. *Multimodal Brain Image Analysis*. Cham: : Springer International Publishing 2013. 107–18. doi:10.1007/978-3-319-02126-3_11

384 Morra J, Tu Z, Toga A, *et al.* Automatic Segmentation of MS Lesions Using a Contextual Model for the MICCAI Grand Challenge. In: *Grand Challenge Work.: Mult. Scler. Lesion Segm. Challenge*. 2008. 1–7.

385 Mote SR, Baid UR, Talbar SN. Non-negative matrix factorization and self-organizing map for brain tumor segmentation. In: *2017 International Conference on Wireless Communications, Signal Processing and Networking (WiSPNET)*. 2017. 1133–7. doi:10.1109/WiSPNET.2017.8299940

386 Muda AF, Saad NM, Waeleh N, *et al.* Integration of Fuzzy C-Means with Correlation Template and Active Contour for Brain Lesion Segmentation in Diffusion-Weighted MRI. In: *2015 3rd International Conference on Artificial Intelligence, Modelling and Simulation (AIMS)*. 2015. 268–73. doi:10.1109/AIMS.2015.88

387 Müller S, Weickert J, Graf N. Automatic brain tumor segmentation with a fast Mumford-Shah algorithm. In: *Medical Imaging 2016: Image Processing*. International Society for Optics and Photonics 2016. 97842S. doi:10.1117/12.2214552

388 Nabizadeh N, Dorodchi M, Kubat M. Automatic tumor lesion detection and segmentation using modified winnow algorithm. In: *2015 IEEE 12th International Symposium on Biomedical Imaging (ISBI)*. 2015. 71–4. doi:10.1109/ISBI.2015.7163819

389 Oliveira GC, Varoto R, Jr AC. Brain Tumor Segmentation in Magnetic Resonance Images using Genetic Algorithm Clustering and AdaBoost Classifier. In: *BIOIMAGING*. 2020. 77–82.https://www.scitepress.org/PublicationsDetail.aspx?ID=y+ZZXSvPyrc=&t=1 (accessed 15 Nov 2020).

390 Osman AFI. Automated Brain Tumor Segmentation on Magnetic Resonance Images and Patient's Overall Survival Prediction Using Support Vector Machines. In: Crimi A, Bakas S, Kuijf H, *et al.*, eds. *Brainlesion: Glioma, Multiple Sclerosis, Stroke and Traumatic Brain Injuries*. Cham: : Springer International Publishing 2018. 435–49. doi:10.1007/978-3-319-75238-9_37

391 Pandian B, Boyle J, Orringer DA. Multimodal tumor segmentation with 3D convolutional neural networks. In: *Proceedings of the MICCAI Challenge on Multimodal Brain Tumor Image Segmentation (BRATS) 2016*. 2016.

392 Parisot S, Duffau H, Chemouny S, *et al.* Graph-based detection, segmentation characterization of brain tumors. In: *2012 IEEE Conference on Computer Vision and Pattern Recognition*. 2012. 988–95. doi:10.1109/CVPR.2012.6247775

393 Parisot S, Duffau H, Chemouny S, *et al.* Joint Tumor Segmentation and Dense Deformable Registration of Brain MR Images. In: Ayache N, Delingette H, Golland P, *et al.*, eds. *Medical Image Computing and Computer-Assisted Intervention – MICCAI 2012*. Berlin, Heidelberg: : Springer 2012. 651–8. doi:10.1007/978-3-642-33418-4_80

394 Patricia Buendia, Thomas Taylor, Michael Ryan, *et al.* A grouping artificial immune network for segmentation of tumor images. In: *Proceedings of the MICCAI Challenge on Multimodal Brain Tumor Image Segmentation (BRATS) 2013*. 2013.

395 Pedoia V, Balbi S, Binaghi E. Fully Automatic Brain Tumor Segmentation by Using Competitive EM and Graph Cut. In: Murino V, Puppo E, eds. *Image Analysis and Processing — ICIAP 2015*. Cham: : Springer International Publishing 2015. 568–78. doi:10.1007/978-3-319-23231-7_51

396 Pereira S, Oliveira A, Alves V, *et al.* On hierarchical brain tumor segmentation in MRI using fully convolutional neural networks: A preliminary study. In: *2017 IEEE 5th Portuguese Meeting on Bioengineering (ENBENG)*. 2017. 1–4. doi:10.1109/ENBENG.2017.7889452

397 Pinto A, Pereira S, Correia H, *et al.* Brain Tumour Segmentation based on Extremely Randomized Forest with high-level features. In: *2015 37th Annual International Conference of the IEEE Engineering in Medicine and Biology Society (EMBC)*. 2015. 3037–40. doi:10.1109/EMBC.2015.7319032

398 Pinto A, Pereira S, Dinis H, *et al.* Random decision forests for automatic brain tumor segmentation on multi-modal MRI images. In: *2015 IEEE 4th Portuguese Meeting on Bioengineering (ENBENG)*. 2015. 1–5. doi:10.1109/ENBENG.2015.7088842

399 Pourreza R, Zhuge Y, Ning H, *et al.* Brain Tumor Segmentation in MRI Scans Using Deeply-Supervised Neural Networks. In: Crimi A, Bakas S, Kuijf H, *et al.*, eds. *Brainlesion: Glioma, Multiple Sclerosis, Stroke and Traumatic Brain Injuries*. Cham: : Springer International Publishing 2018. 320–31. doi:10.1007/978-3-319-75238-9_28

400 Puonti O, Van Leemput K. Simultaneous Whole-Brain Segmentation and White Matter Lesion Detection Using Contrast-Adaptive Probabilistic Models. In: Crimi A, Menze B, Maier O, *et al.*, eds. *Brainlesion: Glioma, Multiple Sclerosis, Stroke and Traumatic Brain Injuries*. Cham: : Springer International Publishing 2016. 9–20. doi:10.1007/978-3-319-30858-6_2

401 Rachmadi MF, Valdés-Hernández M del C, Komura T. Automatic irregular texture detection in brain MRI without human supervision. In: *Medical Image Computing and Computer Assisted Intervention – MICCAI 2018*. Springer. 2018. doi:10.1007/978-3-030-00931-1_58

402 Randhawa RS, Modi A, Jain P, *et al.* Improving Boundary Classification for Brain Tumor Segmentation and Longitudinal Disease Progression. In: Crimi A, Menze B, Maier O, *et al.*, eds. *Brainlesion: Glioma, Multiple Sclerosis, Stroke and Traumatic Brain Injuries*. Cham: : Springer International Publishing 2016. 65–74. doi:10.1007/978-3-319-55524-9_7

403 Raniga P, Schmitt P, Bourgeat P, *et al.* Local intensity model: An outlier detection framework with applications to white matter hyperintensity segmentation. In: *2011 IEEE International Symposium on Biomedical Imaging: From Nano to Macro*. 2011. 2057–60. doi:10.1109/ISBI.2011.5872817

404 Rao A, Ledig C, Newcombe V, *et al.* Contusion segmentation from subjects with Traumatic Brain Injury: A random forest framework. In: *2014 IEEE 11th International Symposium on Biomedical Imaging (ISBI)*. 2014. 333–6. doi:10.1109/ISBI.2014.6867876

405 Rexilius J, Hahn HK, Klein J, *et al.* Multispectral brain tumor segmentation based on histogram model adaptation. In: *Medical Imaging 2007: Computer-Aided Diagnosis*. International Society for Optics and Photonics 2007. 65140V. doi:10.1117/12.709410

406 Reza S, Linmin P, Iftekharuddin KM. Ischemic stroke lesion segmentation using local gradient and texture features. In: *Ischemic Stroke Lesion Segmentation*. 2015.

407 Rezaei M, Yang H, Meinel C. Deep Neural Network with l2-Norm Unit for Brain Lesions Detection. In: Liu D, Xie S, Li Y, *et al.*, eds. *Neural Information Processing*. Cham: : Springer International Publishing 2017. 798–807. doi:10.1007/978-3-319-70093-9_85

408 Riad MM, Platel B, de Leeuw F-E, *et al.* Detection of white matter lesions in cerebral small vessel disease. 2013. 867014. doi:10.1117/12.2007940

409 Rios Piedra EA, Ellingson BM, Taira RK, *et al.* Brain Tumor Segmentation by Variability Characterization of Tumor Boundaries. In: Crimi A, Menze B, Maier O, *et al.*, eds. *Brainlesion: Glioma, Multiple Sclerosis, Stroke and Traumatic Brain Injuries*. Cham: : Springer International Publishing 2016. 206–16. doi:10.1007/978-3-319-55524-9_20

410 Rodrigo F, Graffigna JP, Isoardi R, *et al.* Segmentation of Hyperintense Regions Applied to Multiple Sclerosis Lesions. In: Braidot A, Hadad A, eds. *VI Latin American Congress on Biomedical Engineering CLAIB 2014, Paraná, Argentina 29, 30 & 31 October 2014*. Cham: : Springer International Publishing 2015. 425–8. doi:10.1007/978-3-319-13117-7_109

411 Roy PK, Bhuiyan A, Janke A, *et al.* Automated Segmentation of White Matter Lesions Using Global Neighbourhood Given Contrast Feature-Based Random Forest and Markov Random Field. In: *2014 IEEE International Conference on Healthcare Informatics*. 2014. 1–6. doi:10.1109/ICHI.2014.75

412 Roy S, Maji P. A New Post-processing Method to Detect Brain Tumor Using Rough-Fuzzy Clustering. In: Kryszkiewicz M, Bandyopadhyay S, Rybinski H, *et al.*, eds. *Pattern Recognition and Machine Intelligence*. Cham: : Springer International Publishing 2015. 407–17. doi:10.1007/978-3-319-19941-2_39

For peer review only

413 S. Doyle, M. Reyes, M. Dojat, *et al.* Fully Automatic Brain Tumor Segmentation from Multiple MR Sequences using Hidden Markov Fields and Variational. In: *NCI-MICCAI BraTS*. 2013. /paper/Fully-Automatic-Brain-Tumor-Segmentation-from-MR-Menze-Reyes/c3912d865a263e42d51ae28850cd8332cd1612ee (accessed 16 Nov 2020).

414 S. Reza, K.M. Iftekharuddin. Improved brain tumor tissue segmentation using texture features. In: *Proceedings MICCAI BraTS (brain tumor segmentation challenge)*. 2014. 27–30.

415 Saha R, Phophalia A, Mitra SK. Brain Tumor Segmentation from Multimodal MR Images Using Rough Sets. In: Mukherjee S, Mukherjee S, Mukherjee DP, *et al.*, eds. *Computer Vision, Graphics, and Image Processing*. Cham: : Springer International Publishing 2017. 133–44. doi:10.1007/978-3-319-68124-5_12

416 Sankari A, Vigneshwari S. Automatic tumor segmentation using convolutional neural networks. In: *2017 Third International Conference on Science Technology Engineering Management (ICONSTEM)*. 2017. 268–72. doi:10.1109/ICONSTEM.2017.8261291

417 Schmidt M, Levner I, Greiner R, *et al.* Segmenting brain tumors using alignment-based features. In: *Fourth International Conference on Machine Learning and Applications (ICMLA'05)*. 2005. 6 pp.-. doi:10.1109/ICMLA.2005.56

418 Sehgal A, Goel S, Mangipudi P, *et al.* Automatic brain tumor segmentation and extraction in MR images. In: *2016 Conference on Advances in Signal Processing (CASP)*. 2016. 104–7. doi:10.1109/CASP.2016.7746146

419 Shah N, Ziauddin S, Shahid AR. Brain tumor segmentation and classification using cascaded random decision forests. In: *2017 14th International Conference on Electrical Engineering/Electronics, Computer, Telecommunications and Information Technology (ECTI-CON)*. 2017. 718–21. doi:10.1109/ECTICon.2017.8096339

420 Shaikh M, Anand G, Acharya G, *et al.* Brain Tumor Segmentation Using Dense Fully Convolutional Neural Network. In: Crimi A, Bakas S, Kuijf H, *et al.*, eds. *Brainlesion: Glioma, Multiple Sclerosis, Stroke and Traumatic Brain Injuries*. Cham: : Springer International Publishing 2018. 309–19. doi:10.1007/978-3-319-75238-9_27

421 Shen H, Wang R, Zhang J, *et al.* Multi-task Fully Convolutional Network for Brain Tumour Segmentation. In: Valdés Hernández M, González-Castro V, eds. *Medical Image Understanding and Analysis*. Cham: : Springer International Publishing 2017. 239–48. doi:10.1007/978-3-319-60964-5_21

422 Shivhare SN, Sharma S, Singh N. An Efficient Brain Tumor Detection and Segmentation in MRI Using Parameter-Free Clustering. In: Tanveer M, Pachori RB, eds. *Machine Intelligence and Signal Analysis*. Singapore: : Springer 2019. 485–95. doi:10.1007/978-981-13-0923-6_42

423 Shreyas V, Pankajakshan V. A deep learning architecture for brain tumor segmentation in MRI images. In: *2017 IEEE 19th International Workshop on Multimedia Signal Processing (MMSP)*. 2017. 1–6. doi:10.1109/MMSP.2017.8122291

424 Soltaninejad M, Zhang L, Lambrou T, *et al.* MRI Brain Tumor Segmentation and Patient Survival Prediction Using Random Forests and Fully Convolutional Networks. In: Crimi A, Bakas S, Kuijf H, *et al.*, eds. *Brainlesion: Glioma, Multiple Sclerosis, Stroke and Traumatic Brain Injuries*. Cham: : Springer International Publishing 2018. 204–15. doi:10.1007/978-3-319-75238-9_18

425 Song B, Chou C-R, Chen X, *et al.* Anatomy-Guided Brain Tumor Segmentation and Classification. In: Crimi A, Menze B, Maier O, *et al.*, eds. *Brainlesion: Glioma, Multiple Sclerosis, Stroke and Traumatic Brain Injuries*. Cham: : Springer International Publishing 2016. 162–70. doi:10.1007/978-3-319-55524-9_16

426 Souplet J-C, Lebrun-Frenay C, Ayache N, *et al.* An Automatic Segmentation of T2-FLAIR Multiple Sclerosis Lesions. In: *MICCAI-Multiple Sclerosis Lesion Segmentation Challenge Workshop*. 2008.

427 Srivastava S, Maes F, Vandermeulen D, *et al.* Feature-based statistical analysis of structural MR data for automatic detection of focal cortical dysplastic (FCD) lesions. In: *2004 2nd IEEE International Symposium on Biomedical Imaging: Nano to Macro (IEEE Cat No. 04EX821)*. 2004. 1127-1130 Vol. 2. doi:10.1109/ISBI.2004.1398741

428 Stefan Bauer, Thomas Fejes, Johannes Slotboom, *et al.* Segmentation of brain tumor images based on integrated hierarchical classification and regularization. 2012.

429 Subbanna N, Precup D, Arbel T. Iterative Multilevel MRF Leveraging Context and Voxel Information for Brain Tumour Segmentation in MRI. In: *2014 IEEE Conference on Computer Vision and Pattern Recognition*. 2014. 400–5. doi:10.1109/CVPR.2014.58

430 Subbanna NK, Precup D, Collins DL, *et al.* Hierarchical Probabilistic Gabor and MRF Segmentation of Brain Tumours in MRI Volumes. In: Mori K, Sakuma I, Sato Y, *et al.*, eds. *Medical Image Computing and Computer-Assisted Intervention – MICCAI 2013*. Berlin, Heidelberg: : Springer 2013. 751–8. doi:10.1007/978-3-642-40811-3_94

431 Subbanna N, Precup D, Arnold D, *et al.* IMaGe: Iterative Multilevel Probabilistic Graphical Model for Detection and Segmentation of Multiple Sclerosis Lesions in Brain MRI. In: *International Conference on Information Processing in Medical Imaging*. Cham: : Springer 2015. 514–26. doi:10.1007/978-3-319-19992-4_40

432 Subbanna N, Shah M, Francis S, *et al.* MS Lesion Segmentation using Markov Random Fields. In: *Proceedings of International Conference on Medical Image Computing and Computer Assisted Intervention, London, UK*. 2009.

433 Szilágyi L, Lefkovits L, Iantovics B, *et al.* Automatic Brain Tumor Segmentation in Multispectral MRI Volumetric Records. In: Arik S, Huang T, Lai WK, *et al.*, eds. *Neural Information Processing*. Cham: : Springer International Publishing 2015. 174–81. doi:10.1007/978-3-319-26561-2_21

434 T. K. T. K. Lun, W. Hsu. Brain tumor segmentation using deep convolutional neural network. In: *Proceedings of BRATS-MICCAI*. 2016.

435 Tang H, Lu H, Liu W, *et al.* Tumor segmentation from single contrast MR images of human brain. In: *2015 IEEE 12th International Symposium on Biomedical Imaging (ISBI)*. 2015. 46–9. doi:10.1109/ISBI.2015.7163813

436 Thomas Taylor, Nigel John, Patricia Buendia, *et al.* Map-reduce enabled hidden Markov models for high throughput multimodal brain tumor segmentation. In: *Proceedings of the MICCAI Challenge on Multimodal Brain Tumor Image Segmentation (BRATS) 2013*. 2013.

437 Uchiyama Y, Kunieda T, Hara T, *et al.* Automatic segmentation of different-sized leukoaraiosis regions in brain MR images. In: *Medical Imaging 2008: Computer-Aided Diagnosis*. International Society for Optics and Photonics 2008. 69151S. doi:10.1117/12.770045

438 Urban G, Bendszus M, Hamprecht F, *et al.* Multi-modal Brain Tumor Segmentation using Deep Convolutional Neural Networks. In: *MICCAI BraTS (brain tumor segmentation) challenge*. 2014. /paper/Multi-modal-Brain-Tumor-Segmentation-using-Deep-Urban-Bendszus/97efa3acd4a45b28f3c9e25c961a9379abdc811b (accessed 15 Nov 2020).

For peer review only - http://bmjopen.bmj.com/site/about/guidelines.xhtml

439 Vaidhya K, Thirunavukkarasu S, Alex V, *et al.* Multi-modal Brain Tumor Segmentation Using Stacked Denoising Autoencoders. In: Crimi A, Menze B, Maier O, *et al.*, eds. *Brainlesion: Glioma, Multiple Sclerosis, Stroke and Traumatic Brain Injuries*. Cham: : Springer International Publishing 2016. 181–94. doi:10.1007/978-3-319-30858-6_16

440 Vaidya S, Chunduru A, Muthuganapathy R, *et al.* Longitudinal multiple sclerosis lesion segmentation using 3D convolutional neural networks. In: *Proceedings of the 2015 Longitudinal Multiple Sclerosis Lesion Segmentation Challenge*. 2015. 1–2.

441 Vaishnavee KB, Amshakala K. An automated MRI brain image segmentation and tumor detection using SOM-clustering and Proximal Support Vector Machine classifier. In: *2015 IEEE International Conference on Engineering and Technology (ICETECH)*. 2015. 1–6. doi:10.1109/ICETECH.2015.7275030

442 Wang G, Li W, Ourselin S, *et al.* Automatic Brain Tumor Segmentation Using Cascaded Anisotropic Convolutional Neural Networks. In: Crimi A, Bakas S, Kuijf H, *et al.*, eds. *Brainlesion: Glioma, Multiple Sclerosis, Stroke and Traumatic Brain Injuries*. Cham: : Springer International Publishing 2018. 178–90. doi:10.1007/978-3-319-75238-9_16

443 Wang T, Cheng I, Basu A. Fully automatic brain tumor segmentation using a normalized Gaussian Bayesian Classifier and 3D Fluid Vector Flow. In: *2010 IEEE International Conference on Image Processing*. 2010. 2553–6. doi:10.1109/ICIP.2010.5652559

444 Wang Y, Katsaggelos AK, Wang X, *et al.* A deep symmetry convnet for stroke lesion segmentation. In: *2016 IEEE International Conference on Image Processing (ICIP)*. 2016. 111–5. doi:10.1109/ICIP.2016.7532329

445 Weiss N, Rueckert D, Rao A. Multiple Sclerosis Lesion Segmentation Using Dictionary Learning and Sparse Coding. In: Mori K, Sakuma I, Sato Y, *et al.*, eds. *Medical Image Computing and Computer-Assisted Intervention – MICCAI 2013*. Berlin, Heidelberg: : Springer 2013. 735–42. doi:10.1007/978-3-642-40811-3_92

446 Xu Y, Géraud T, Puybareau É, *et al.* White Matter Hyperintensities Segmentation in a Few Seconds Using Fully Convolutional Network and Transfer Learning. In: Crimi A, Bakas S, Kuijf H, *et al.*, eds. *Brainlesion: Glioma, Multiple Sclerosis, Stroke and Traumatic Brain Injuries*. Cham: : Springer International Publishing 2018. 501–14. doi:10.1007/978-3-319-75238-9_42

447 Xuan Chen, Nguyen BP, Chee-Kong Chui, *et al.* Automated brain tumor segmentation using kernel dictionary learning and superpixel-level features. In: *2016 IEEE International Conference on Systems, Man, and Cybernetics (SMC)*. 2016. 002547–52. doi:10.1109/SMC.2016.7844622

448 Xuan X, Liao Q. Statistical Structure Analysis in MRI Brain Tumor Segmentation. In: *Fourth International Conference on Image and Graphics (ICIG 2007)*. 2007. 421–6. doi:10.1109/ICIG.2007.181

449 Yoo Y, Tang LW, Brosch T, *et al.* Deep Learning of Brain Lesion Patterns for Predicting Future Disease Activity in Patients with Early Symptoms of Multiple Sclerosis. In: Carneiro G, Mateus D, Peter L, *et al.*, eds. *Deep Learning and Data Labeling for Medical Applications*. Cham: : Springer International Publishing 2016. 86–94. doi:10.1007/978-3-319-46976-8_10

450 Yu C-P, Ruppert G, Nguyen D, *et al.* Statistical Asymmetry-based Brain Tumor Segmentation from 3D MR Images. 2012.

451 Yu R, Xiao L, Wei Z, *et al.* Automatic Segmentation of White Matter Lesions Using SVM and RSF Model in Multi-channel MRI. In: Zhang Y-J, ed. *Image and Graphics*. Cham: : Springer International Publishing 2015. 654–63. doi:10.1007/978-3-319-21978-3_57

452 Zabir I, Paul S, Rayhan MA, *et al.* Automatic brain tumor detection and segmentation from multi-modal MRI images based on region growing and level set evolution. In: *2015 IEEE International WIE Conference on Electrical and Computer Engineering (WIECON-ECE)*. 2015. 503–6. doi:10.1109/WIECON-ECE.2015.7443979

453 Zacharaki EI, Erus G, Bezerianos A, *et al.* Fuzzy Multi-channel Clustering with Individualized Spatial Priors for Segmenting Brain Lesions and Infarcts. In: Iliadis L, Maglogiannis I, Papadopoulos H, *et al.*, eds. *Artificial Intelligence Applications and Innovations*. Berlin, Heidelberg: : Springer 2012. 76–85. doi:10.1007/978-3-642-33412-2_8

454 Zacharaki EI, Kanterakis S, Bryan RN, *et al.* Measuring Brain Lesion Progression with a Supervised Tissue Classification System. In: Metaxas D, Axel L, Fichtinger G, *et al.*, eds. *Medical Image Computing and Computer Assisted Intervention – MICCAI 2008*. Berlin, Heidelberg: : Springer 2008. 620–7. doi:10.1007/978-3-540-85988-8_74

455 Zhan T, Gu S, Jiang L, *et al.* A Novel Brain Tumor Segmentation Method for Multi-Modality Human Brain MRIs. In: *MUE 2015*. 2015. doi:10.14257/ijmue.2015.10.11.11

456 Zhao L, Duygu Sarikaya, Corso JJ. Automatic brain tumor segmentation with MRF on supervoxels. In: *Multimodal Brain Tumor Segmentation*. 2013. 51.

457 Zhe Xiao, Ruohan Huang, Yi Ding, *et al.* A deep learning-based segmentation method for brain tumor in MR images. In: *2016 IEEE 6th International Conference on Computational Advances in Bio and Medical Sciences (ICCABS)*. 2016. 1–6. doi:10.1109/ICCABS.2016.7802771

458 Zhiqiang Lao, Dinggang Shen, Jawad A, *et al.* Automated segmentation of white matter lesions in 3D brain MR images, using multivariate pattern classification. In: *3rd IEEE International Symposium on Biomedical Imaging: Nano to Macro, 2006.* 2006. 307–10. doi:10.1109/ISBI.2006.1624914

459 Zikic D, Glocker B, Konukoglu E, *et al.* Context-sensitive Classification Forests for Segmentation of Brain Tumor Tissues. 2012.

460 Zikic D, Glocker B, Konukoglu E, *et al.* Decision Forests for Tissue-Specific Segmentation of High-Grade Gliomas in Multi-channel MR. In: Ayache N, Delingette H, Golland P, *et al.*, eds. *Medical Image Computing and Computer-Assisted Intervention – MICCAI 2012*. Berlin, Heidelberg: : Springer 2012. 369–76. doi:10.1007/978-3-642-33454-2_46

461 Yi D, Zhou M, Chen Z, *et al.* 3-D Convolutional Neural Networks for Glioblastoma Segmentation. *arXiv:161104534 [cs]* Published Online First: 14 November 2016.http://arxiv.org/abs/1611.04534 (accessed 15 Nov 2020).

462 Beers A, Chang K, Brown J, *et al.* Sequential 3D U-Nets for Biologically-Informed Brain Tumor Segmentation. *arXiv:170902967 [cs]* Published Online First: 9 September 2017.http://arxiv.org/abs/1709.02967 (accessed 16 Nov 2020).

463 Styner M, Lee J, Chin B, *et al.* 3D Segmentation in the Clinic: A Grand Challenge II: MS lesion segmentation. *The MIDAS Journal* 2008;:638.

464 Menze BH, Jakab A, Bauer S, *et al.* The Multimodal Brain Tumor Image Segmentation Benchmark (BRATS). *IEEE Trans Med Imaging* 2015;**34**:1993–2024. doi:10.1109/TMI.2014.2377694

465 Carass A, Roy S, Jog A, *et al.* Longitudinal multiple sclerosis lesion segmentation: Resource and challenge. *NeuroImage* 2017;**148**:77–102. doi:10.1016/j.neuroimage.2016.12.064

466 Maier O, Menze BH, von der Gablentz J, *et al.* ISLES 2015 - A public evaluation benchmark for ischemic stroke lesion segmentation from multispectral MRI. *Med Image Anal* 2017;**35**:250–69. doi:10.1016/j.media.2016.07.009

467 Sled JG, Zijdenbos AP, Evans AC. A nonparametric method for automatic correction of intensity nonuniformity in MRI data. *IEEE Trans Med Imaging* 1998;**17**:87–97. doi:10.1109/42.668698

468 Tustison NJ, Avants BB, Cook PA, *et al.* N4ITK: improved N3 bias correction. *IEEE Trans Med Imaging* 2010;**29**:1310–20. doi:10.1109/TMI.2010.2046908

469 Smith SM. Fast robust automated brain extraction. *Hum Brain Mapp* 2002;**17**:143–55. doi:10.1002/hbm.10062

470 William Penny, Karl Friston, John Ashburner, *et al.*, editors. *Statistical Parametric Mapping: The Analysis of Functional Brain Images - 1st Edition*. 1st ed. Elsevier 2006. https://www.elsevier.com/books/statistical-parametric-mapping-the-analysis-of-functional-brain-images/penny/978-0-12-372560-8 (accessed 23 Nov 2020).

471 Smith SM, Jenkinson M, Woolrich MW, *et al.* Advances in functional and structural MR image analysis and implementation as FSL. *Neuroimage* 2004;**23 Suppl 1**:S208-219. doi:10.1016/j.neuroimage.2004.07.051

472 Bø HK, Solheim O, Jakola AS, *et al.* Intra-rater variability in low-grade glioma segmentation. *J Neurooncol* 2017;**131**:393–402. doi:10.1007/s11060-016-2312-9

473 Zijdenbos AP, Dawant BM, Margolin RA, *et al.* Morphometric analysis of white matter lesions in MR images: method and validation. *IEEE Trans Med Imaging* 1994;**13**:716–24. doi:10.1109/42.363096

474 Gibson E, Hu Y, Huisman HJ, *et al.* Designing image segmentation studies: Statistical power, sample size and reference standard quality. *Med Image Anal* 2017;**42**:44–59. doi:10.1016/j.media.2017.07.004

475 Recent Advances in MRI Technology. Imaging Technology News. 2016.https://www.itnonline.com/article/recent-advances-mri-technology (accessed 23 Nov 2020).

476 OECD Statistics. https://stats.oecd.org/Index.aspx?ThemeTreeId=9 (accessed 23 Nov 2020).

477 FDA Clears First 7T MRI System, Magnetom Terra. Imaging Technology News. 2017.https://www.itnonline.com/content/fda-clears-first-7t-mri-system-magnetom-terra (accessed 23 Nov 2020).

478 Maier-Hein L, Eisenmann M, Reinke A, *et al.* Why rankings of biomedical image analysis competitions should be interpreted with care. *Nat Commun* 2018;**9**:5217. doi:10.1038/s41467-018-07619-7

479 Jorritsma W, Cnossen F, van Ooijen PMA. Improving the radiologist-CAD interaction: designing for appropriate trust. *Clin Radiol* 2015;**70**:115–22. doi:10.1016/j.crad.2014.09.017

480 Gryska E, Cerna K, Heckemann RA. Increasing trust through the design of algorithm-based lesion segmentation support systems. In: *iConference 2020 Proceedings*. iSchools 2020. https://www.ideals.illinois.edu/handle/2142/106560 (accessed 23 Nov 2020).

FIGURE LIST AND CAPTIONS

500 Figure 1: *Flowchart of the article selection process from the result of querying three databases. For each stage, the number of articles selected is shown and numbers of excluded articles and reasons are given.*

For peer review only

Figure 2: *The number of algorithms described in the included articles developed for and validated on particular lesion type. The distinction is made between multiple sclerosis (MS) lesions and other white matter lesions (WML) following the similar distinction present in the reviewed articles and lesion segmentation challenges. "Multiple lesion types" refers to algorithms that were evaluated on more than one type of lesion. "Other lesion types" types of lesions included focal cortical dysplasia, metastasis, traumatic brain injury, cerebral palsy, abscess, and necrosis.*

Figure 3: *The number of articles published per year in the eligible sample with an indication of the number of articles that presented a segmentation method for a particular lesion type. We fit a linear function to the number of papers published each year from 2012 to estimate the development of the field in the upcoming years.*

Figure 4: The number of patients (n) whose images were used to validate the methods in the eligible articles.

Figure 5: *Distribution of the most common algorithms and models implemented in the lesion segmentation methods proposed by the articles eligible for the review (ANN – artificial neural networks, DT – decision trees, EM – expectation maximization, FC – fuzzy clustering, MM – mixture models, RF – random fields, SVM – support vector machines, THR – thresholding).*

For peer review only

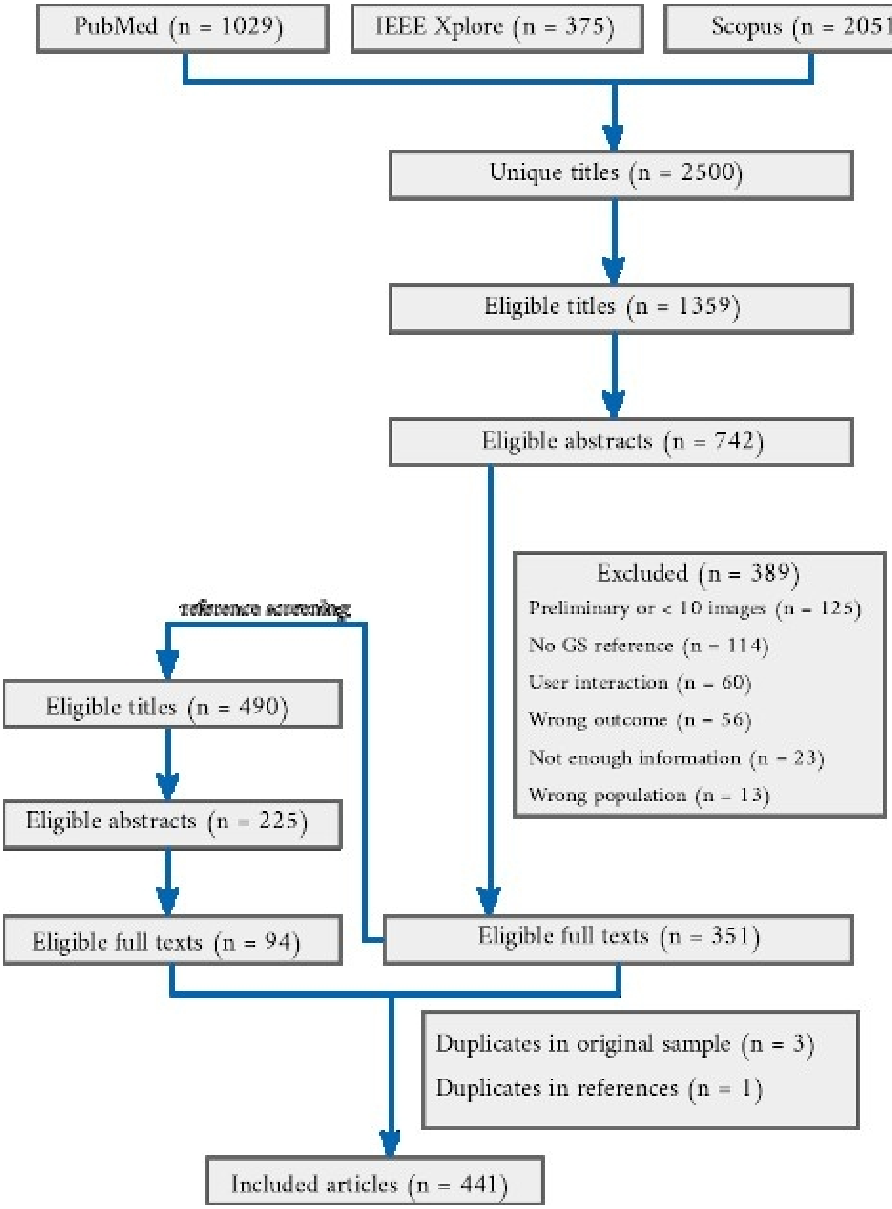

Flowchart of the article selection process from the result of querying three databases. For each stage, the number of articles selected is shown and numbers of excluded articles and reasons are given.

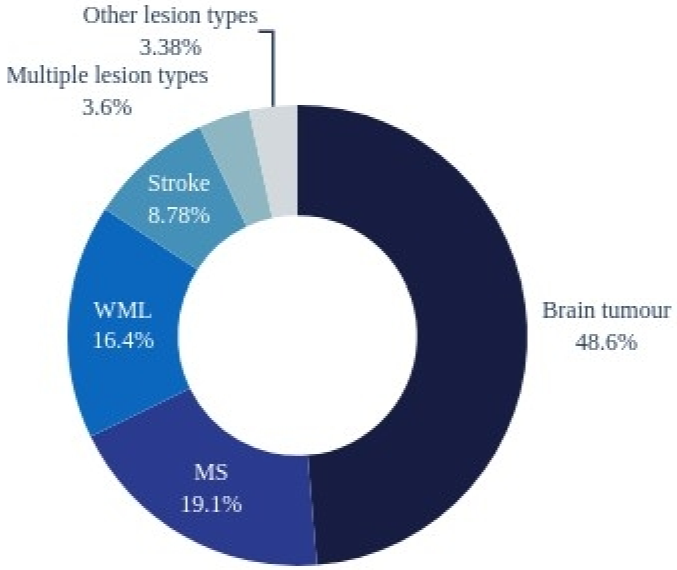

The number of algorithms described in the included articles developed for and validated on particular lesion type. The distinction is made between multiple sclerosis (MS) lesions and other white matter lesions (WML) following the similar distinction present in the reviewed articles and lesion segmentation challenges. “Multiple lesion types” refers to algorithms that were evaluated on more than one type of lesion. “Other lesion types” types of lesions included focal cortical dysplasia, metastasis, traumatic brain injury, cerebral palsy, abscess, and necrosis.

89x89mm (96 x 96 DPI)

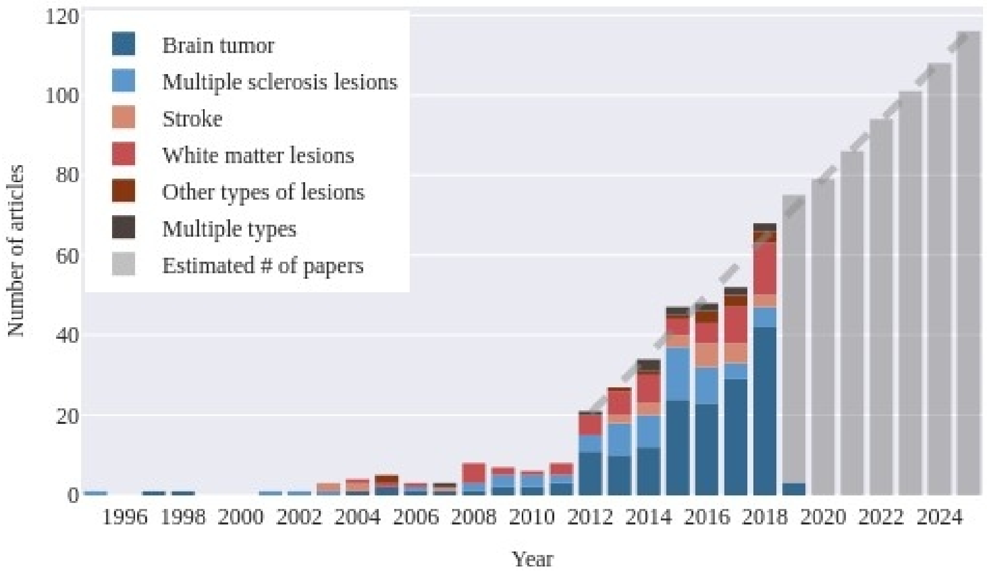

The number of articles published per year in the eligible sample with an indication of the number of articles that presented a segmentation method for a particular lesion type. We fit a linear function to the number of papers published each year from 2012 to estimate the development of the field in the upcoming years.

137x80mm (96 x 96 DPI)

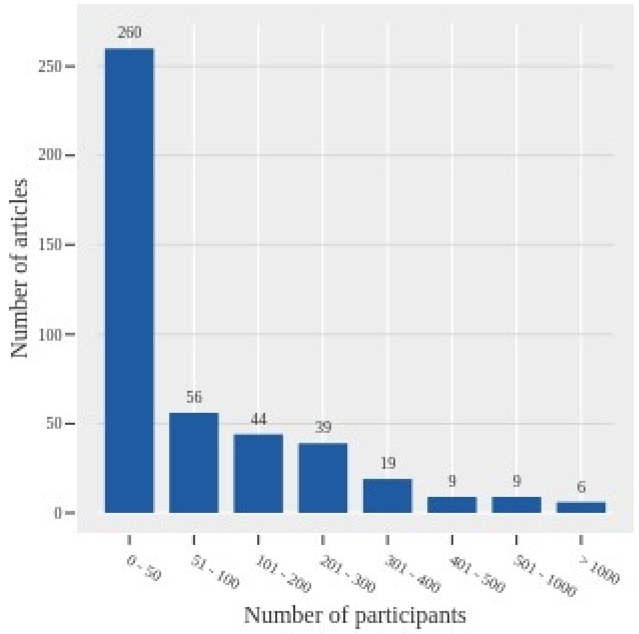

The number of patients (n) whose images were used to validate the methods in the eligible articles.

89x89mm (96 x 96 DPI)

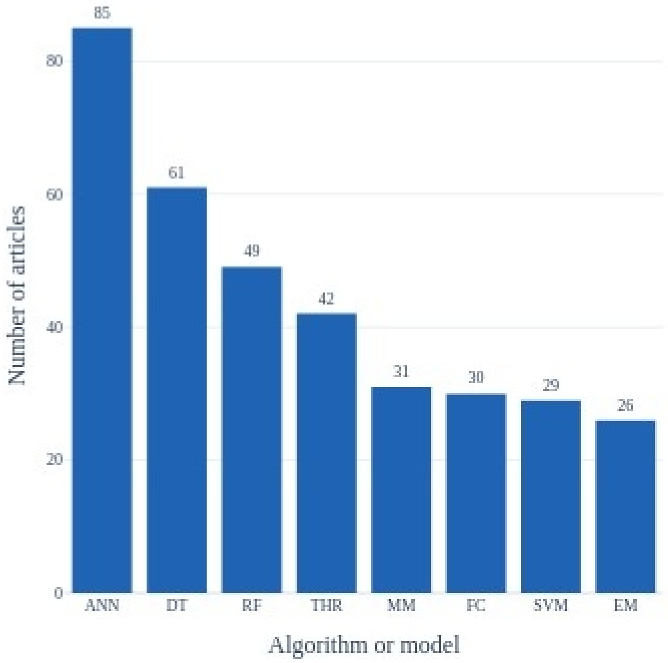

Distribution of the most common algorithms and models implemented in the lesion segmentation methods proposed by the articles eligible for the review (ANN – artificial neural networks, DT – decision trees, EM – expectation maximization, FC – fuzzy clustering, MM – mixture models, RF – random fields, SVM – support vector machines, THR – thresholding).

89x89mm (96 x 96 DPI)

Bibliographic data					General comments	Segmentation								Study cohort					Validation		
Item type	Publication year	Author(s)	Title	Publication Title		Multi-sequence input	Input sequence(s):	Algorithms and computational theories	Preprocessing procedures	Hardware	Segmentation time	Lesion classification used	Software made available by authors	Lesion type	Diagnosed conditions	Sample size	Sample source corrected	Multiscanner images	Reference segmentation	Decision (if multiple raters and specified)	Evaluation measures
C - conference paper<br>J - journal article						**y - yes<br>n - no**	CBF – Cerebral blood flow<br>DIR – Double inversion recovery<br>DSC – Dynamic susceptibility contrast<br>DTI – Diffusion tensor imaging<br>DWI – Diffusion weighted imaging<br>FA – Fractional anisotropy<br>FLAIR – Fluid-attenuated inversion recovery<br>FSE – Fast spin echo<br>MPR/MPRAGE – Magnetization Prepared Rapid Acquisition Gradient Echo<br>PD – Proton density<br>PWI – Perfusion weighted imaging<br>(R)CBV – (Relative) Cerebral blood volume<br>SD – Spin density weighted<br>T1c – T1 weighted with contrast<br>T1 – T1 weighted<br>T2 – T2 weighted<br>Tmax – Time to maximum<br>TTP – Time to peak		0u1v – 0 mean unit variance<br>ADF – Anidotropic diffusion filtering<br>BE – Brain extraction<br>BFC – Bias field(field inhomogeneity) correction<br>EM – Expectation maximization<br>IN – Intensity normalization<br>M(N)I – Mutual (normalized) information<br>N3 – Sled et al. 1998<br>N4 - Tustison et al. 2010<br>n/s – Not specified<br>REG – Image (co-) registration<br>WM – White matter			**y - yes<br>n - no**	**y - yes<br>n - no**	AD – Alzheimer's disease BT – Brain tumour CADASIL - Cerebral autosomal dominant arteriopathy with subcortical infarcts and leukoencephalopathy CI – Cognitive impairment CIS – Clinically isolatd syndrome CP – Cerebral palsy D - Dementia DM(2) – Diabetes mellitus (type II) EP – Epilepsy FCD – Focal cortical displasia GB(M) – Glioblastoma (multiforme) G – glioma HEM – Hemorrhage or bleeding HGG - high grade glioma HT – hypertension IFL – ischemic focal lesions LBD – Lewy body dementia LGG - low grade glioma MCI - minimal cognitive impairement MET – Metastasis MS – Multiple sclerosis lesions NEC – Necrosis (RR)MS - (Relapsing-remitting) multiple sclerosis SLE - systemic lupus erythematosus S – Stroke SVD – Small vessel disease TBI – Traumatic brain Injury T - Tumours VaD – Vascular disease WML – White matter lesions (other than MS lesions)			**1 – Publicably available data with reference segmentation (source)**<br><br>**2 – Publicably available data without reference segmentation (source)**<br><br>**3 – Non-public data sources (source)**	**y - yes<br>n - no**	**Procedure, number of raters**		Acc - Accuracy<br>AD - Average distance<br>avg - Average<br>DE - Detection error<br>DER - Detection error rate<br>DSC - Dice similarity coefficient<br>F1 - F1 score<br>FN - False negative<br>FNR - False negative ratio<br>FP - False positive<br>FPR - False positive rate<br>(F)ROC - (Free-response Receiver) operator curve<br>Hdff - Hausdorff distance<br>ICC - Inter-class correlation<br>Jcc - Jaccard coefficient<br>Kappa - Kappa coefficient<br>NPV - Negative predictive value<br>OER - Outline error rate<br>PPV - Positive predictive value<br>sd - Standard deviation<br>SD - Surface distance ??<br>Sen, TPR, Overlap fraction - Sensitivity<br>SI - Similarity index<br>Spe - Specificity<br>SSD - Symmetric SD<br>TI - Tanimoto index<br>TP - True positive<br>VC - Volume correlation<br>VD - Volume difference<br>VE - Volume error<br>VO - Volume overlap<br>VR - Regression analysis of volume
J	2014	M. Cabezas et al.	BOOST: A supervised approach for multiple sclerosis lesion segmentation	Journal of Neuroscience Methods		y	T1, T2, PD, FLAIR	Boosting	BE:BET; BFC:N4; denoising:ADF; IN:histogram matching	n/a	n/a	n	n	MS	-	45	3	y	Semiautomatic		DSC; avgSD
J	2014	S. Parisot et al.	Concurrent tumor segmentation and registration with uncertainty-based sparse non-uniform graphs	Medical Image Analysis	Inter-rater agreement evaluated	y	T2, FLAIR	Markov Random Field	BE; REG:rigid; IN:matching median and interquartile range to reference	n/a	~5 min	n	n	BT	LGG	110 (27 segmented); 10	1 (BraTS'12); 3	y	Manual, 2		DSC; FPR; TPR; MAD
J	2013	M. D. Steenwijk et al.	Accurate white matter lesion segmentation by k nearest neighbor classification with tissue type priors (kNN-TTPs)	NeuroImage: Clinical	Inter-rater agreement evaluated	y	T1, FLAIR	K-Nearest Neighbours	BE:BET; BFC:N3	n/a	n/a	n	n	WML	MS; HT	20; 20	3	y	Manual, 3	consensus	DSC; ICC; Sen; OER; DER
J	2018	D. S. Meier et al.	Dual-Sensitivity Multiple Sclerosis Lesion and CSF Segmentation for Multichannel 3T Brain MRI	Journal of Neuroimaging	Scan-rescan reproducibility	y	T1, T2, FLAIR	Other	BFC:N4; REG:rigid, affine + Bspline(BRAINS/ITK); BE:BET; tissue maps:Freesurfer; IN:histogram matching	n/a	n/a	n	n	MS	MS	29; 13x2	3	n	Manual, 2	supervision	DSC; Jcc; Hdff; Spe; PPV; Sen; ICC; Bland-Altman
J	2013	A. Sanjuán et al.	Automated identification of brain tumors from single MR images based on segmentation with refined patient-specific priors	Front. Neurosci.	Inter-rater agreement evaluated	n	T1	Previously proposed	n/a	n/a	n/a	n	n	BT	T	18 + 64 healthy	3	n	Manual, 2		DSC; ROC
C	2014	S. Roy et al.	Example based lesion segmentation	Proceedings of SPIE - The International Society for Optical Engineering		y	T1, FLAIR	Previously proposed	BE; IN:mean WM intensity=1	n/a	n/a	n	n	WML	MS	47	3 (described in Shee et al. 2009)	n	Manual		DSC;VC; Sen; Spe
J	2016	A. Galimzianova et al.	Stratified mixture modeling for segmentation of white-matter lesions in brain MR images	NeuroImage	Manual corrections of preprocessing (brain extraction)	y	T1, T2, FLAIR	Comparison	BE:Iglesias; REG:rigid; BFC:N4; downsampling	n/a	n/a	n	n	MS	MS	30	3	n	Manual, 2	consensus	DSC
C	2015	S. Roy and P. Maji	A New Post-processing Method to Detect Brain Tumor Using Rough-Fuzzy Clustering	Pattern Recognition and Machine Intelligence		n/a		Fuzzy C-Means, Region Growing	n/a	n/a	n/a	n	n	BT	-	10	1 (BraTS'12)	y	-		DSC; other
J	2011	S. Klöppel et al.	A comparison of different automated methods for the detection of white matter lesions in MRI data	NeuroImage	Manual corrections of preprocessing	y	T1, FLAIR	Comparison	REG:MI; tissue maps, spatial normalization, BFC:SPM8; IN:0 median + 1 interquartile interval; resampling	n/a	few sec to 5 hrs	n	n	WML	MCI, D	20	3	n	Manual, 1&2		PPV; Sen; ROC; DSC
C	2018	A. Bougacha et al.	Comparative study of supervised and unsupervised classification methods: Application to automatic MRI glioma brain tumors segmentation	[illegible] International Conference on Advanced Technologies for Signal and Image [illegible]		y	T1, T1c, T2, FLAIR	Comparison	n/a	n/a	n/a	n	n	BT	LGG; HGG	273	1 (BraTS'15)	y	-		DSC; Jcc
J	2005	S. Srivastava et al.	Feature-based statistical analysis of structural MR data for automatic detection of focal cortical dysplastic lesions	NeuroImage		n	T1	Previously proposed	REG:affine; tissue maps w/ non-rigid registration; IN	n/a	n/a	n	n	FCD	FCD	17 + 64 controls	3	y	Manual		VO
J	2013	S. Datta and P. A. Narayana	A comprehensive approach to the segmentation of multichannel three-dimensional MR brain images in multiple sclerosis	NeuroImage: Clinical		y	T1, T2, FLAIR	Other	REG:rigid (SPM2); BE:thresholding; BFC:SPM2 + ADF; IN:histogram matching	n/a	n/a	n	n	MS	-	60; 50	1 (MS GC MICCAI'08); 3	y	Segmentation correction		VD; avgSD; TPR; FPR; Bland-Altman; VC
J	2016	E. Roura et al.	Automated Detection of Lupus White Matter Lesions in MRI	Front. Neuroinform	Integrated preprocessing	y	T1, FLAIR	Thresholding	REG:SPM; BE:SPM; tissue masks:SPM; denoising:ADF; BFC:EM-based	n/a	n/a	n	y	WML	SLE	20	3	n	Manual, 1		DSC; PPV; TPR; VC; Bland-Altman; ROC
J	2018	W. Chen et al.	Computer-Aided Grading of Gliomas Combining Automatic Segmentation and Radiomics	International Journal of Biomedical Imaging		y	T1, T1c, T2, FLAIR	Previously proposed	BFC:N4; IN:histogram matching + 0u1v	GeForce GTX 1080 Ti GPU	n/a	LGG, HGG	n	BT	LGG; HGG	274	1 (BraTS'15)	y	-		DSC; Sen; Spe

For peer review only

J	2018	P. Moeskops et al.	Evaluation of a deep learning approach for the segmentation of brain tissues and white matter hyperintensities of presumed vascular origin in MRI	NeuroImage: Clinical	Inter-rater variability evaluated (visual scores)	y	T1, T1 inversion recovery, FLAIR	Artificial neural networks	n/a	n/a	n/a	n	n	WML	DM2	20; 96 relatively healthy; 110	3 (UDES2, Reijmer et al., 2013);<br>3 (the Dutch Parelsnoer Study, Aalten et al., 2014)	n	Manual		DSC; avgSD; VC; Sen; DER; OER
J	2018	Y. Ling et al.	Validation and Optimization of BIANCA for the Segmentation of Extensive White Matter Hyperintensities	Neuroinformatics	Inter-rater agreement evaluated	works with single/multi	T1, FLAIR	Previously proposed	BE:BET; REG:FLIRT; IN:variance scaling	I5 3.2GHz	4-22 min	n	y	WML	CADASIL	90; 66	3 (Chabriat et al. 2016)	y	Semiautomatic		DSC; FPR; FNR; ICC; Bland-Altman
J	2018	M. Dadar et al.	Validation of T1w-based segmentations of white matter hyperintensity volumes in large-scale datasets of aging	Human Brain Mapping		n	T1	Previously proposed	denoising:ref; BFC:ref; IN:ref; REG:non-linear	n/a	n/a	n	n	WML	MCI; AD	70; 669; 481	2 (ADNI);<br>2 (ADNI2/GO);<br>3 (Davis Alzheimer's Disease Center, Hinton et al. 2010)	y	Manual, 1		VC; VR
J	2018	O. Charron et al.	Automatic detection and segmentation of brain metastases on multimodal MR images with a deep convolutional neural network	Computers in Biology and Medicine		n/a	T1, T1c, FLAIR	Artificial neural networks	REF:rigid (FLIRT) + resampling; BE:BET; BFC:N4; IN:0u1v + thresholding	n/a	n/a	n	n	MET	-	182 + 3 of other lesion causes	3 (Paul Strauss Center, Strasbourg, France)	n	Manual, multiple raters		DSC; Sen; PPV
J	2017	R. Meier et al.	Automatic estimation of extent of resection and residual tumor volume of patients with glioblastoma	Journal of Neurosurgery	Intended clinical application	y	T1, T1c, T2, FLAIR	Previously proposed	BE:ITK; REG	n/a	n/a	n	n	BT	GB	19	3	y	Manual, 5	supervision	other
J	2017	F. Kellner-Weldon et al.	Comparison of perioperative automated versus manual two-dimensional tumor analysis in glioblastoma patients	European Journal of Radiology	Intended clinical application, Preprocessing failure, Segmentation failure	y	T1, T1c, T2, FLAIR	Previously proposed	n/a	n/a	n/a	n	n	BT	GB	92x2	3	y	n/a		other
J	2017	A. de Sitter et al.	Performance of five research-domain automated WM lesion segmentation methods in a multi-center MS study	NeuroImage	Inter-rater agreement evaluated	y*	T1, FLAIR	Comparison	n/a	n/a	n/a	n	n	MS	MS	70	3 (MAGNIMS, Ropele et al., 2014)	y	Manual, 2	supervision	VD; ICC; DSC; DER; OER
J	2017	R. McKinley et al.	Fully automated stroke tissue estimation using random forest classifiers (FASTER)	J Cereb Blood Flow Metab	Inter-rater agreement evaluated	y	T1c, T2, DWI, DSC, PWI	Decision Tree	denoising:Gaussian iltering; REG:rigid; BE:ref; resampling:linear interpolation; IN:0-225 range	n/a	n/a	n	n	S	S	80x2 + 20	3 (Bernese stroke registry)	y	Semiautomatic		ROC; Sen; Spe; PPV
C	2015	R. McKinley et al.	Segmenting the ischemic penumbra: a decision forest approach with automatic threshold finding	Brainlesion: Glioma, Multiple Sclerosis, Stroke and Traumatic Brain Injuries		y	T1c, T2, DWI, CBF, CBV, TTP, Tmax	Decision Tree	filtering:ITK; IN: 0225r; REG	n/a	n/a	n	n	S	-	50	1 (ISLES'15)	n	-		DSC
J	2017	Y. Renping et al.	Coarse Classification to Region-Scalable Refining for White Matter Lesions Segmentation in Multi-Channel MRI	CNS & Neurological Disorders-Drug Targets		y	T1, T2, PD, FLAIR	Support Vector Machine, Level Set	REG:rigid; BE:ref; BFC: N3; IN: paper	n/a	n/a	n	n	WML	-	45	3 (ACCORD-MIND)	y	Manual, 1		TPR; TNR; PPV; DSC
J	2016	Ž. Lesjak, F. Pernuš, B. Likar, Ž. Špiclin	Validation of White-Matter Lesion Change Detection Methods on a Novel Publicly Available MRI Image Database	Neuroinformatics		y	T1, T2, FLAIR	Comparison	REG:affine; tissue maps – WM; BFC:N4; BE:BET 2; resampling:ANTs; IN:M u & v	n/a	n/a	n	n	WML	MS	20x2	3 (University Medical Centre Ljubljana)	y	Manual, 2	consensus	ROC; DSC; other
J	2016	M. B. Hansen et al.	Automated estimation of salvageable tissue: Comparison with expert readers.	Journal of Magnetic Resonance Imaging		y	PWI, DWI	Previously proposed	n/a	I7 2.6GHz	28 sec	n	y	S	-	167	3 (I-KNOW)	y	Manual, 4	consensus	DSC; VC; Bland-Altman
J	2016	R. Meier et al.	Clinical Evaluation of a Fully-automatic Segmentation Method for Longitudinal Brain Tumor Volumetry	Scientific Reports	Inter-rater agreement evaluated, Treatment response evaluation, Survival prediction	y	T1, T1c, T2, FLAIR	Previously proposed	BE; REG:rigid	n/a	n/a	n	y	BT	GBM	64 (14xN)	3	n	Manual, 2		VD; DSC
J	2016	N. Porz et al.	Fully Automated Enhanced Tumor Compartmentalization: Man vs. Machine Reloaded	PloS one		y	T1, T1c, T2, FLAIR	Previously proposed	n/a	n/a	n/a	n	n	BT	-	19	3	y	Manual, 2	supervision	DSC; PPV; Sen; VD; other
J	2015	E. Rios Velazquez et al.	Fully automatic GBM segmentation in the TCGA-GBM dataset: Prognosis and correlation with VASARI features	Scientific Reports		y	T1, T1c, T2, FLAIR	Previously proposed	BE; REG:rigid	n/a	5 min	Overall survival; 1 year survival	y	BT	GB	109	2 (TCIA, Clark et al. 2013)	y	Manual		VC; VR
J	2015	O. Maier et al.	Classifiers for Ischemic Stroke Lesion Segmentation: A Comparison Study	PloS one	Inter-rater agreement evaluated, Errors in preprocessing, Segmentation failure	both	T1 and/or DWI, FLAIR	Comparison	downsampling:ref; BE:ref; BFC:ref; IN:ref	n/a	n/a	n	n	S	S	37	3 (Meier et al. 2015)	n	Manual, 2		DSC; avgSSD; Hdff; Sen; PPV; ROC
J	2015	S. Roy et al.	Subject-Specific Sparse Dictionary Learning for Atlas-Based Brain MRI Segmentation	IEEE Journal of Biomedical and Health Informatics		y	T1, T2, PD, FLAIR	Sparse representation, Dictionary Learning	REG:rigid (FLIRT); BFC:N4; BE:Spectre	2.7GHz 12-core AMD	15 min	n	n	MS	MS	18; 122	3	n	Manual, 1		DSC; VD; TPR; FPR; avgSSD

For peer review only - http://bmjopen.bmj.com/site/about/guidelines.xhtml

J	2014	N. Porz et al.	Multi-Modal Glioblastoma Segmentation: Man versus Machine	PloS one		y	T1, T1c, T2, FLAIR	Previously proposed	n/a	n/a	n/a	n	n	BT	GBM	25	1 (BraTS'12); 3	y	Manual, 3	supervision	DSC; PPV; Sen; VE
J	2014	J. Gao et al.	Non-locally regularized segmentation of multiple sclerosis lesion from multi-channel MRI data	Magnetic Resonance Imaging		y	T1, T2, FLAIR	Other	REG:rigid	n/a	n/a	n	n	MS	MS	20	1 (MS GC MICCAI'08)	y	-		DSC; Spe; FNR; VD
J	2013	J. A. Maldjian et al.	Automated White Matter Total Lesion Volume Segmentation in Diabetes	American Journal of Neuroradiology	Visual validation of preprocessing	y	T1, FLAIR	Other	REG:toMNI; tissue maps:DARTEL + SPM8	n/a	n/a	n	n	WML	DM2	50 (+50 healthy)	3 (Diabetes Heart Study-Mind, Bowden et al. 2010)	n	Manual, 4	supervision, consensus	VC; Bland-Altman
J	2013	K. Nagenthiraja et al.	Automated Decision-Support System for Prediction of Treatment Responders in Acute Ischemic Stroke	Front. Neurol.	Intended clinical application	y	PWI, DWI	Previously proposed	REG:linear; reslicing:SPM8	I7 2.6GHz	31 sec	n	n	S	-	288	3 (I-KNOW); 3 (Remote Ischemic Perconditioning Study, Hougaard et al. 2013)	y	Manual, 4	majority	VC; VD
J	2011	S. D. Smart et al.	Validation of Automated White Matter Hyperintensity Segmentation	Journal of Aging Research	Inter-rater agreement evaluated	y	T1, FLAIR	Previously proposed	REG:SPM	n/a	n/a	n	n	WML	-	30	3 (Firbank et al. 2010)	n	Manual, 2		Jcc
J	2004	G. P. Mazzara et al.	Brain tumor target volume determination for radiation treatment planning through automated MRI segmentation	International Journal of Radiation Oncology*Biology*Physics	Inter-rater agreement evaluated; Segmentation failure	y	T1c, T2, PD	Previously proposed	REG	n/a	n/a	n	n	BT	G	11x2	3	y	Manual, 3		Acc
J	2003	S. Mehta et al.	Evaluation of voxel-based morphometry for focal lesion detection in individuals	NeuroImage		n	T1	Previously proposed	n/a	n/a	n/a	n	n	S	IFL	10	3 (Cognitive Neuroscience Patient Registry)	n	Manual, 1		VC; avgSSD; sdSSD; other
C	2017	S. Jain et al.	Unsupervised Framework for Consistent Longitudinal MS Lesion Segmentation	Medical Computer Vision and Bayesian and Graphical Models for Biomedical Imaging	Scan-rescan reproducibility	y	T1, FLAIR	Previously proposed	REG; BFC; IN:histogram matching	n/a	n/a	n	n	WML	MS	10; 12	3	y	Expert segmentation		TPR; FPR; DSC
C	2016	C. C. Benson et al.	Brain tumor segmentation from MR brain images using improved fuzzy c-means clustering and watershed algorithm	2016 International Conference on Advances in Computing, Communications and Informatics (ICACCI)		n	T1	Fuzzy C-Means, Watershed	denoising: median filter; BE: ref; contrast enhancement:ref	n/a	n/a	n	n	BT	GBM	90	3	n	Manual		DSC; TI
C	2013	N. Weiss et al.	Multiple Sclerosis Lesion Segmentation Using Dictionary Learning and Sparse Coding	Medical Image Computing and Computer-Assisted Intervention – MICCAI 2013		n	FLAIR	Sparse representation, Dictionary Learning	BE:BET; BFC:N4; IN: sqrt of largest L1 norm of all patches	2.4GHz 4GB RAM	5 min	n	n	MS	MS	20	1 (MS GC MICCAI'08)	y	-		DSC; TPR; PPV
C	2013	H. Wang and P.A. Yushkevich	Multi-atlas Segmentation without Registration: A Supervoxel-Based Approach	MICCAI 2013		y	T1, T1c, T2, FLAIR	Other	IN:histogram equalization + 0-1 range	2 GHz	few sec	n	n	BT	LGG; HGG	20 (25 synthetic)	1 (BraTS'12)	y	-		DSC
C	2009	D. García-Lorenzo et al.	Multiple Sclerosis Lesion Segmentation Using an Automatic Multimodal Graph Cuts	MICCAI 2009	Inter-rater agreement evaluated	y	T1, T2, PD	Expectation-Maximization Gausian mixture model, Graph Cut	BFC:ref; REG:ref; BE:ref	n/a	n/a	n	n	MS	MS	10	3	n/a	Manual, 5	majority	DSC
C	2006	J. Corso et al.	Multilevel Segmentation and Integrated Bayesian Model Classification with an Application to Brain Tumor Segmentation	MICCAI 2006		y	T1, T1c, FLAIR	Bayesian classification	REG:FSL; denoising:FSL; BE:FSL; IN:FSL	n/a	n/a	n	n	BT	GBM	30	3	n/a	Manual		Jcc
J	2019	J. Tong et al.	MRI brain tumor segmentation based on texture features and kernel sparse coding	Biomedical Signal Processing and Control		n	FLAIR	Sparse representation, Dictionary Learning	IN:0-225 range; denoising:Gaussian filtering; brain mask	4GHz 32GB RAM	38 sec	n	n	BT	LGG; HGG	50	1 (BraTS'12)	y	-		Jcc; DSC; Spe; Sen
J	2018	J. Tong et al.	Kernel sparse representation for MRI image analysis in automatic brain tumor segmentation	Frontiers of Information Technology & Electronic Engineering		y	T1, T1c, T2, FLAIR	K-Means, Sparse representation	IN:0-225 range; denoising:Gaussian filtering; brain mask	4GHz 32 GB RAM	40s	n	n	BT	LGG; HGG	384	1 (BraTS'15)	y	-		DSC; PPV; Sen; Kappa

For peer review only

J	2018	O. Ghribi et al.	Multiple sclerosis exploration based on automatic MRI modalities segmentation approach with advanced volumetric evaluations for essential feature extraction	Biomedical Signal Processing and Control		y	T1, T1c; T1, T2, FLAIR; T1, T2, PD, FLAIR	Support Vector Machine, Genetic Algorithm	BFC; REG:BET; BE: FSL; denoising: ADF; IN	n/a	n/a	n	n	MS	MS	70; 45; 10x4, 3x5, 1x6	1 (MS GC MICCAI'08); 1 (MSC'15); 3 (Habib-Bourguiba University Hospital)	y	Manual, 1&2		DSC; TPR; PPV; Jcc; TPR; FPR; FP; VD
J	2018	A. Vishnuvarthanan et al.	Development of a combinational framework to concurrently perform tissue segmentation and tumor identification in T1 - W, T2 - W, FLAIR and MPR type magnetic resonance brain images	Expert Systems with Applications		y	T1, T2, FLAIR, MPR	Fuzzy C-Means, Bacteria Foraging Optimization	IN: histogram equalization	I7 2.6GHz 8GB RAM	unclear	n	n	BT	T	4; 25	1 (BraTS'13); 2 (The Whole Brain Atlas);	y	Manual		DSC; Sen; Spe
J	2018	J. Jiang et al.	UBO Detector – A cluster-based, fully automated pipeline for extracting white matter hyperintensities	NeuroImage		y	T1, FLAIR	K-Nearest Neighbours	REG:SPM12; tissue maps:SPM12; REG:DARTEL; BE	2.2GHz 256GB RAM	15 min	n	y	WML	-	397; 1037 (subsample longitudinal)	3 (OATS, Sachdev et al. 2013); 3 (Sydney Memory and Ageing Study, Sachdev et al. 2010)	y	Manual, 2		DSC; TPR; FPR; Spe; Acc; ICC
J	2018	H. Li et al.	Fully convolutional network ensembles for white matter hyperintensities segmentation in MR images	NeuroImage		y	T1, FLAIR	Artificial neural networks	cropping; IN:Gaussian normalization	32 GB RAM	CPU: 60 sec GPU: 8 sec	n	y	WML	MS	170	1 (WMH Segmentation Challenge '17)	y	-		DSC; Hdff; avgVD; Sen; F1
J	2018	T. Kaur et al.	A joint intensity and edge magnitude-based multilevel thresholding algorithm for the automatic segmentation of pathological MR brain images	Neural Comput & Applic	No preprocessing	n	FLAIR	Thresholding, Particle Swarm Optimization	IN:0-255 range; BE:Otsu's thresholding	3.1GHz i5 4GB RAM	5 sec	n	n	BT	LGG	10; 20; 10	1 (BraTS'12); 1 (BraTS'15); 3 (Advance Diagnostic Centre, Ludhiana, Punjab, India)	y	Manual,1		DSC; Jcc; other
J	2018	L. Rundo et al.	NeXt for neuro-radiosurgery: A fully automatic approach for necrosis extraction in brain tumor MRI using an unsupervised machine learning technique	International Journal of Imaging Systems and Technology		n	T1c	Fuzzy C-Means	morphological operations on GTV	2.8GHz i7 16BG RAM	< 1 sec	n	n	NEC	-	32	3	n	Manual,1		DSC; Jcc; Sen; Spe; MAD; Hdff; other
J	2018	M. Soltaninejad et al.	Supervised learning based multimodal MRI brain tumour segmentation using texture features from supervoxels	Computer Methods and Programs in Biomedicine		y	T1, T1c, T2, FLAIR, DTI	Random Forest	Eddy curent correction:FSL; BE:BET; REG:SPM12; IN:histogram matching + dynamic range normalization	n/a	n/a	n	n	BT	LGG; HGG	11; 30	1 (BraTS'13); 3	y	Manual,1		PPV; Sen; DSC; other
J	2018	E. A. AlBadawy et al.	Deep learning for segmentation of brain tumors: Impact of cross-institutional training and testing	Medical Physics	Minimal preprocessing	y	T1, T1c, FLAIR	Artificial neural networks	REG:FLIRT	n/a	n/a	n	n	BT	GB	44	2 (TCIA, Clark et al. 2013)	y	Manual, 3	supervision	DSC; avgHdff; avgVD; Sen; PPV
J	2018	T. Kaur et al.	A novel fully automatic multilevel thresholding technique based on optimized intuitionistic fuzzy sets and tsallis entropy for MR brain tumor image segmentation	Australas Phys Eng Sci Med	No preprocessing	n	FLAIR	Thresholding, Particle Swarm Optimization	none	3.10GHz i5 4GB RAM	< 5 sec	n	n	BT	LGG; HGG	8; 8	1 (BraTS'12); 3	y	Manual		DSC; Jcc; other
J	2018	M. Salem et al.	A supervised framework with intensity subtraction and deformation field features for the detection of new T2-w lesions in multiple sclerosis	NeuroImage: Clinical		y	T1, PD, T2, FLAIR	Logistic Regression	BE:ROBEX; BFC:N4; IN:histogram matching; REG:ITKv4Demons	n/a	n/a	n	y	MS	MS	60x2	3 (Vall d'Hebron Hospital)	n	Semiautomatic		DSC; VC
J	2018	E. B. Shimol et al.	Computer-based radiological longitudinal evaluation of meningiomas following stereotactic radiosurgery	Int J CARS		n	T1c	Active Contour	REG:SPM; IN:histogram normalization	n/a	n/a	n	n	BT	MEN	28	3	n	Manual, 1		DSC; Sen; maxSD; avgSD
J	2018	Z. Zhao et al.	Automated glioma detection and segmentation using graphical models	PloS one		y	T1, T1c, T2, FLAIR	Conditional Random Field	n/a	n/a	n/a	n	n	BT	LGG; HGG	30; 274; 161	1 (BraTS'13); 1 (BraTS'15); 3 (Henan Provincial People's Hospital)	y	Manual, 1		DSC; Hdff; VC; Spe; Sen
J	2018	M. M. Cheriyan et al.	Blind source separation with mixture models – A hybrid approach to MR brain classification	Magnetic Resonance Imaging		y	T1, T2, FLAIR	Gaussian Mixture Model, Particle Swarm Optimization	n/a	n/a	n/a	n	n	BT, MS, S	BT, MS, HEM	38 + 22 healthy; 152	3 (Medall Diagnostics, Kochi)	n	Manual, 3		Jcc; Sen; Spe; Acc
J	2018	M. B. Naceur et al.	Fully Automatic Brain Tumor Segmentation using End-To-End Incremental Deep Neural Networks in MRI images	Computer Methods and Programs in Biomedicine		y	T1, T1c, T2, FLAIR	Artificial neural networks	IN:1-99% clipping + 0u1v	GPU	22 sec	n	n	BT	-	285	1 (BraTS'17)	y	-		DSC; Sen; Spe; Hdff
J	2018	M. K. Abd-Ellah et al.	Two-phase multi-model automatic brain tumour diagnosis system from magnetic resonance images using convolutional neural networks	EURASIP Journal on Image and Video Processing	Detection followed by segmentation	n	T2	Artificial neural networks	resizing and RGB conversion	n/a	n/a	n	n	BT	LGG; HGG	19; 30	1 (BraTS'13)	y	-		Sen; Spe; Acc; PPV; NPV; DSC
J	2018	J. K. Boldsen et al.	Better Diffusion Segmentation in Acute Ischemic Stroke Through Automatic Tree Learning Anomaly Segmentation	Front. Neuroinform.		n	DWI	Decision Tree	IN: division by mean of contralateral hemisphere; smoothing:Gaussian kernel; morphological operations	n/a	n/a	n	n	S	S	108	3 (I-KNOW)	y	Manual, 1		DSC

For peer review only

J	2018	R. Guerrero et al.	White matter hyperintensity and stroke lesion segmentation and differentiation using convolutional neural networks	NeuroImage: Clinical		y	T1, FLAIR	Artificial neural networks	REG:FLIRT; reslicing; tissue maps; IN:0u1v + clipping (3stdev)	n/a	n/a	n	n	WML, S	S	167	3 (Brain Research Imaging Centre of Edinburgh)	n* various protocols	Semiautomatic		DSC; VD; other
J	2018	T. Zhan et al.	A Glioma Segmentation Method Using CoTraining and Superpixel-Based Spatial and Clinical Constraints	IEEE Access		y	T1, T1c, T2, FLAIR	Support Vector Machine, Sparse representation	BFC:N4; IN: 0-255 range	n/a	n/a	n	n	BT	LGG; HGG	30	1 (BraTS'12);<br>1 (BraTS'13)	y	-		DSC; PPV; Sen
J	2018	S. Cui et al.	Automatic Semantic Segmentation of Brain Gliomas from MRI Images Using a Deep Cascaded Neural Network	Journal of Healthcare Engineering		y	T1, T1c, T2, FLAIR	Fuzzy C-Means, Artificial neural networks	BFC:N4; IN:1-99% clipping + 0u1v	Intel E5 GTX 1080	2 sec	n	n	BT	LGG; HGG	274	1 (BraTS'15)	y	-		DSC; PPV; Sen
J	2018	S. Bonte et al.	Machine learning based brain tumour segmentation on limited data using local texture and abnormality	Computers in Biology and Medicine		y	T1c, FLAIR	Random Forest	REG:SPM12; tissue maps: SPM; BFC:SPM12	n/a	n/a	n	n	BT	LGG; HGG	30; 285; 257	1 (BraTS'17);<br>1 (BraTS'17);<br>3 (Ghent University Hospital)	y	n/a		DSC
J	2018	M. J. Fartaria et al.	Partial volume-aware assessment of multiple sclerosis lesions	NeuroImage: Clinical		y	T1, FLAIR	Other	REG:rigid (ELASTIX); BFC:N4; BE:in house; tissue maps:in house	n/a	n/a	n	n	MS	early MS	39	3	n	Manual, 2	consensus	DSC; FPR; Detection Rate
J	2018	C. Ma et al.	Concatenated and Connected Random Forests With Multiscale Patch Driven Active Contour Model for Automated Brain Tumor Segmentation of MR Images	IEEE Transactions on Medical Imaging		y	T1, T1c, T2, FLAIR	Random Forest, Active Contour	n/a	n/a	5 min	n	n	BT	-	n/a	1 (BraTS'15);<br>1 (TCIA, Bakas et al. 2017)	y	-		DSC; Sen; Spe
J	2018	I. Razzak et al.	Efficient Brain Tumor Segmentation with Multiscale Two-Pathway-Group Conventional Neural Networks	IEEE Journal of Biomedical and Health Informatics		y	T1, T1c, T2, FLAIR	Artificial neural networks	IN:1-99% clipping + 0u1v + 0-1 range; BFC:N4	n/a	3 min	n	n	BT	-	285 + 110;	1 (BraTS'13);<br>1 (BraTS'15)	y	-		DSC; Sen; Spe
J	2018	S. Hussain et al.	Segmentation of glioma tumors in brain using deep convolutional neural network	Neurocomputing		y	T1, T1c, T2, FLAIR	Artificial neural networks	BFC:N4; IN: 1-99% clipping + 0u1v	i7 16GB RAM	5 min	n	n	BT	LGG; HGG	30; 274	1 (BraTS'13);<br>1 (BraTS'15)		-		DSC; Sen; Spe
J	2018	S. Banerjee et al.	Automated 3D segmentation of brain tumor using visual saliency	Information Sciences	Inter-rater agreement evaluated	y	T1c, T2, FLAIR	Other, Grow Cut	BRATS only	I7 3.4GHz 16GB RAM	2 min	n	n	BT	LGG; HGG	30	1 (BraTS'13)	y	-		DSC; Hdff; other
J	2018	E. Binaghi et al.	Meningioma and peritumoral edema segmentation of preoperative MRI brain scans	Computer Methods in Biomechanics and Biomedical Engineering: Imaging & Visualization		y	T1c, FLAIR	Sparse representation, Graph Cut	REG:Iterative Closest Point; contrast enhancement:log transform	2.26GHz	145 sec	n	n	BT	T	15	3	n	Manual, 3	majority	DSC; Jcc; VE
J	2018	C. Qin et al.	A large margin algorithm for automated segmentation of white matter hyperintensity	Pattern Recognition		y	T1, FLAIR	Other	REG:FLIRT; tissue maps:ref; IN:ref	3.6GHz 16GB RAM	16 sec	n	n	WML	-	88	3 (Brain Research Imaging Centre of Edinburgh)	n	Semiautomatic		DSC; Acc; PPV; Sen; Bland-Altman; VC; other
J	2018	Y. Ding et al.	Multi-modal brain tumor image segmentation based on SDAE	International Journal of Imaging Systems and Technology		y	T1, T1c, T2, FLAIR	Artificial neural networks	contrast enhancement:top-hat transform	12 GB RAM	n/a	n	n	BT	-	95 ?	1 (BraTS'15)	y	-		DSC; Jcc
J	2018	A. M. Valcarcel et al.	MIMoSA: An Automated Method for Intermodal Segmentation Analysis of Multiple Sclerosis Brain Lesions	Journal of Neuroimaging		y	T1, T2, FLAIR, PD	Logistic Regression	REG:rigid; BFC:N3; brain tissue extraction:BE:SPECTRE + thresholding; IN:0u1v or WhiteStripe	n/a	22 min	n	n	MS	MS	94; 19xn	1 (MSC'15);<br>3 (Johns Hopkins Hospital)	y	Manual, 1		DSC; ROC; VC
J	2018	A. R. Raju et al.	Bayesian HCS-based multi-SVNN: A classification approach for brain tumor segmentation and classification using Bayesian fuzzy clustering	Biocybernetics and Biomedical Engineering		y	T1, T1c, T2, FLAIR	Fuzzy Clustering	n/a	2GB RAM	n/a	benign, malignant	n	BT	LGG; HGG	30	1 (BraTS'12)	y	-		Sen
J	2018	A. Essadike et al.	Brain tumor segmentation with Vander Lugt correlator based active contour	Computer Methods and Programs in Biomedicine		n/a	n/a	Active Contour, Other	n/a	n/a	<1 sec	n	n	BT	LGG; HGG	45 + 65 synthetic; 35	1 (BraTS'12);<br>1 (BraTS'13)	y	-		DSC; Hdff; Spe; Sen
J	2018	Y. Wang et al.	Voxel-based automated detection of focal cortical dysplasia lesions using diffusion tensor imaging and T2-weighted MRI data	Epilepsy & Behavior		y	T2, DTI	Other	BE:FSL; IN; REG; tissue maps; BFC; resampling	n/a	n/a	n	n	FCD		12	3 (Sanbo Brain Hospital Capital Medical University)	n	Manual, 1		ROC; FPR
J	2018	A. Pinto et al.	Hierarchical brain tumour segmentation using extremely randomized trees	Pattern Recognition		y	T1, T1c, T2, FLAIR	Decision Tree	BFC:N4; IN: histogram matching	n/a	n/a	n	n	BT	LGG; HGG	65	1 (BraTS'13)	y	-		DSC; PPV; Sen
J	2018	G. B. Praveen et al.	Ischemic stroke lesion segmentation using stacked sparse autoencoder	Computers in Biology and Medicine		y	T1, T2, DWI, FLAIR	Artificial neural networks	BFC:N4; IN:0u1v + whitening; resizing	3.5GHz 32 GB RAM	10 min	n	n	S	S	28	1 (ISLES'15)	y	-		DSC; PPV; Sen; Spe; Acc; ROC

For peer review only

J	2018	J. Knight et al.	Voxel-Wise Logistic Regression and Leave-One-Source-Out Cross Validation for white matter hyperintensity segmentation	Magnetic Resonance Imaging		n	FLAIR	Logistic Regression	BFC:SPM12; REG:affine + non-linear; resmpling:trilinear; BE:brain tissue probability maps; IN:histogram matching + 0-1 range	n/a	n/a	n	y	WML	-	96; 110	1 (WMH Segmentation Challenge '17); 1 (MSC'15); 1 (MS Lesion Segmentation Challenge '16)	y	-		DSC; PPV; Sen
J	2018	S. Amiri et al.	Tree-based Ensemble Classifier Learning for Automatic Brain Glioma Segmentation	Neurocomputing		y	T1c, T2, FLAIR	Decision Tree, Bayesian classification	BFC:N4; IN:histogram linear transformation	n/a	n/a	n	n	BT	HGG	50	1 (BraTS'15)	y	-		DSC
J	2018	J. Liu et al.	A Cascaded Deep Convolutional Neural Network for Joint Segmentation and Genotype Prediction of Brainstem Gliomas	IEEE Transactions on Biomedical Engineering		n	T1	Artificial neural networks	resizing; cropping	n/a	n/a	prediction of H3 K27M mutation	n	BT	T; LGG, HGG	55; 280	1 (BraTS'17); 3	y	Manual, 2		DSC; SD
J	2018	S. Sasikanth and S. Suresh Kumar	Glioma tumor detection in brain MRI image using ANFIS-based normalized graph cut approach	International Journal of Imaging Systems and Technology		n/a	n/a	Graph Cut	n/a	2.4GHz 2GB RAM	n/a	normal, abnormal	n	BT	LGG; HGG	100	1 (BraTS'15)	y	-		DSC; Acc; Sen; Spe
J	2018	J. V. Manjón et al.	MRI white matter lesion segmentation using an ensemble of neural networks and overcomplete patch-based voting	Computerized Medical Imaging and Graphics		n	FLAIR	Thresholding, Artificial neural networks	denoising:non-local means; REG:affine; BFC:SPM12; BE:SPM12; IN:division by median and squared	i7 16GB RAM	3 min	n	n	WML	-	128;20	1 (MS GC MICCAI'08); 3 (AIBL, Ellis et al., 2009)	y	Manual, 1		DSC; Spe; Sen; VD; VC
J	2018	M. F. Rachmadi et al.	Segmentation of white matter hyperintensities using convolutional neural networks with global spatial information in routine clinical brain MRI with none or mild vascular pathology	Computerized Medical Imaging and Graphics	Inter-rater agreement evaluated	y	T1, FLAIR	Artificial neural networks	REG:rigid(FLIRT); BE:opti-BET + morphological operations; tissue maps:FAST; IN:histogram matching + 0u1v	3.4GHz GPU 8GB RAM	9 sec	n	n	WML	-	20x3; 268 (with only Fazekas scores)	2 (ADNI)	y	Semiautomatic		DSC; PPV; Sen; VC; VD
J	2017	A. R. Abdulraqeb et al.	An Automated Method for Segmenting Brain Tumors on MRI Images	Biomed Eng		n	T1c	Thresholding	n/a	n/a	n/a	n	n	BT	-	12; 44	3	y	Expert segmentation		DSC; Jcc; Sen; Spe
J	2017	S. Cui et al.	Brain tumor Segmentation using fully convolutional networks	Journal of Medical Imaging and Health Informatics		y	T1, T1c, T2, FLAIR	Artificial neural networks	BFC:N4; IN:mode & deviation scaling + 0-255 range	GeForce GTX 1080; 2 Intel e5 2603 CPUs	<30 sec	n	n	BT	HGG	220	1 (BraTS'15)	y	-		DSC; PPV; Sen
J	2017	Y. Zhuge et al.	Brain tumor segmentation using holistically nested neural networks in MRI images	Medical Physics		y	T1, T1c, T2, FLAIR	Artificial neural networks	BFC:N4; IN:histogram matching	2.66GHz 32 GB RAM	30 sec	n	n	BT	HGG	20; 10	1 (BraTS'13); 3	y	Manual, 1		DSC; Sen
J	2017	L. Chen et al.	Fully automatic acute ischemic lesion segmentation in DWI using convolutional neural networks	NeuroImage: Clinical		n	DWI	Artificial neural networks	resampling:homogeneous linear; IN:0u1v	i7 16GB RAM	<1 sec	n	n	S	S	741	3	y	Expert segmentation		DSC; Detection Rate; other
J	2017	M. Dadar et al.	Performance comparison of 10 different classification techniques in segmenting white matter hyperintensities in aging	NeuroImage	Inter-rater agreement evaluated	y	T1, T2, PD, FLAIR	Comparison	denoising:MINCnlm; BFC:MINCnu_estimate; IN:histogram matching & 0-100 range; REG; BE	i7 2.6GHz 20GB RAM	0.11 – 3021 sec	n	y	WML	CI	70; 32; 53; 46	2 (ADNI); 2 (ADNI2/GO); 2 (NACC) 3 (Davis Alzheimer's Disease Center, Hinton et al. 2010)	y	Manual, 2		DSC; ICC; Sen; PPV; OER; DER
J	2017	K. Murphy et al.	Automatic quantification of ischemic injury on diffusion-weighted MRI of neonatal hypoxic ischemic encephalopathy	NeuroImage: Clinical	Inter-rater agreement evaluated	y	ADC, DWI	Random Forest	BE:BET2; BFC:N4; IN:histogram matching	n/a	n/a	n	n	S	S	74	3 (Wilhelmina Children's Hospital, University Medical Center Utrecht)	y	Manual, 2		ROC: Sen; Spe; F1; VC
J	2017	S. Damangir et al.	Reproducible segmentation of white matter hyperintensities using a new statistical definition	Magnetic Resonance Materials in Physics, Biology and Medicine		y	T1, T2, PD, FLAIR	Other	REG:FLIRT; BE:BET; BFC:N3; tissue maps:FSL-FAST	n/a	n/a	n	y	WML	CI	119	3 (Kings Health Partners-Dementia Case Register)	n	Manual, 1		FNR; VC; DSC
J	2017	M. Soltaninejad et al.	Automated brain tumour detection and segmentation using superpixel-based extremely randomized trees in FLAIR MRI	International Journal of Computer Assisted Radiology and Surgery		n	FLAIR	Decision Tree	n/a	n/a	n/a	n	n	BT	-	19; 30	1 (BraTS'12); 3	y	Manual, 1		DSC; Sen; PPV; other
J	2017	M. Dadar et al.	Validation of a Regression Technique for Segmentation of White Matter Hyperintensities in Alzheimer's Disease	IEEE Transactions on Medical Imaging		y	T1, T2, PD, FLAIR	Linear regression, Thresholding	denoising:non-local means; BFC:N3; IN:histogram matching	i3 3.3GHz	1.6 sec	n	n	WML	-	80; 40; 10	2 (ADNI2/GO); 3 (Davis Alzheimer's Disease Center, Hinton et al. 2010) 3 (PREVENT-AD, Tremblay-Mercier et al. 2014)	y	Semiautomatic; Manual, 2	union; supervision	ICC; DSC; FPR; Sen
J	2017	Z. Li et al.	Low-Grade Glioma Segmentation Based on CNN with Fully Connected CRF	Journal of Healthcare Engineering		n	FLAIR	Artificial neural networks, Conditional Random Field	BE:BrainSuite; BFC:BrainSuite	Intel Xeon 2.4GHz Nvidia Quadro	2-10 min	n	n	BT	LGG	160	3 (Shanghai Huashan Hospital)	n	Manual, 2		DSC; PPV; Sen
J	2017	A. Galimzianova et al.	Locally adaptive magnetic resonance intensity models for unsupervised segmentation of multiple sclerosis lesions	Journal of Medical Imaging	Inter-rater agreement evaluated	y	T1, T2, FLAIR	Gaussian Mixture Model, Markov Random Field	REG:ref; BE:ref; BFC:ref; resampling	n/a	n/a	n	n	MS	MS	30	3	n	Semiautomatic		DSC; VC
J	2017	C. Bowles et al.	Brain lesion segmentation through image synthesis and outlier detection	NeuroImage: Clinical		y	T1, FLAIR	Gaussian Mixture Model, Support Vector Machine	REG:rigid+free form (MIRTK); BFC:N4; BE:pincram; tissue maps:MALPEM; IN:ref	n/a	n/a	n	n	WML	SVD	127	3 (Brain Research Imaging Centre of Edinburgh)	n – different protocols	Semiautomatic		DSC; avgSD; Hdff; PPV; Sen; ICC; Bland-Altman

For peer review only

J	2017	O. Ghribi et al.	An Advanced MRI Multi-Modalities Segmentation Methodology Dedicated to Multiple Sclerosis Lesions Exploration and Differentiation	IEEE Transactions on NanoBioscience	Inter-rater agreement evaluated	y	T1c, FLAIR	Gaussian Mixture Model, Thresholding	REG:rigid; resampling:spline interpolation; BFC; BE:FSL; denoising: ADF; IN	i5 1.8GHz	2 min	n	n	MS	-	70 + 50 healthy; 45; 21x4 (or more)	1 (MS GC MICCAI'08); 1 (MSC'15); 3 (Habib-Bourguiba University Hospital)	y	Manual, 1		DSC; Jcc; PPV; TPR; FPR; SD; VD; DE; OE
J	2017	K. Kamnitsas et al.	Efficient multi-scale 3D CNN with fully connected CRF for accurate brain lesion segmentation	Medical Image Analysis		y	n/a	Artificial neural networks	BE:ROBEX; resampling; REG:affine; IN:0u1v	NVIDIA GTX Titan X GPU 3GB RAM	3 min	n	y	TBI; BT; S	-	61; 384; 64	1 (ISLES'15); 1 (BraTS'15); 3 (Neurosciences Critical Care Unit at Addenbrooke's Hospital, Cambridge, UK)	y	n/a		DSC; Sen; PPV; avgSD; Hdff
J	2017	V. Rajinikanth et al.	Entropy based segmentation of tumor from brain MR images – a study with teaching learning based optimization	Pattern Recognition Letters		y	T1c, T2, FLAIR	Level Set	BE:thresholding; Multilevel thresholding + teaching-learning based optimization – Shannon entropy optimization	n/a	n/a	n	n	BT	-	15?	1 (BraTS'12)	y	-		DSC; Jcc; FPR; FNR; PPV; F-score; Sen; Spe; Acc; other
J	2017	S. Abbasi and F. Tajeripour	Detection of brain tumor in 3D MRI images using local binary patterns and histogram orientation gradient	Neurocomputing		y	T1, T1c, T2, FLAIR	Thresholding, Random Forest	BFC:N4; IN:histogram matching	4GHz 32GB RAM	1900 sec	n	n	BT	LGG; HGG	30 (+50 simulated)	1 (BraTS'13)	y	-		DSC; Jcc
J	2017	F. Binczyk et al.	MIMSeg - an algorithm for automated detection of tumor tissue on NMR apparent diffusion coefficient maps	Information Sciences		y	DWI (ADC), FLAIR	Expectation-Maximization Gausian mixture model, K-Means	BE:BET; tissue maps:Otsu's thresholding	n/a	n/a	n	n	BT	GBM	17x7	3 (Maria Sklodowska-Curie Memorial Cancer Center and Institute of Oncology Gliwice Branch, Gliwice, Poland)	n	Manual, 1		DSC; Sen; Spe; PPV; NPV
J	2017	M. Rincón et al.	Improved Automatic Segmentation of White Matter Hyperintensities in MRI Based on Multilevel Lesion Features	Neuroinformatics	Inter-rater agreement evaluated; Visual inspection of preprocessing	y	T1, FLAIR	Thresholding, Support Vector Machine	REG:rigid; BFC: SPM8; tissue maps:Freesurfer; IN:Gaussian fit	n/a	n/a	n	n	WML	S, MCI	28	3 (Selnes et al. 2015)	n	Manual, 3	majority	DSC; other
J	2017	M. Havaei et al.	Brain tumor segmentation with Deep Neural Networks	Medical Image Analysis		y	T1, T1c, T2, FLAIR	Artificial neural networks	BFC:N4; IN:1-99% clipping + 0u1v	GPU	~3 min	n	n	BT	LGG; HGG	65	1 (BraTS'13)	y	-		DSC; Sen; Spe
J	2017	T. Zhan et al.	Brain Tumor Segmentation Using Deep Belief Networks and Pathological Knowledge	CNS & Neurological Disorders-Drug Targets		y	T1, T1c, T2, FLAIR	Logistic Regression, Graph Cut, Artificial neural networks, Markov Random Field	n/a	n/a	n/a	n	n	BT	LGG; HGG	10 hgg + N LGG (+20 synthetic)	1 (BraTS'12); 1 (BraTS'13)	y	-		DSC; Sen; PPV; F-score
J	2017	E. Kellner et al.	Automated Infarct Core Volumetry Within the Hypoperfused Tissue: Technical Implementation and Evaluation	Journal of Computer Assisted Tomography		y	PWI, DWI	Other	BE; hemisphere extraction	n/a	1 min	n	n	S	S	30	1 (ISLES'15)	y	-		TPR; FPR; FDR; DSC
J	2017	S. Adler et al.	Novel surface features for automated detection of focal cortical dysplasias in paediatric epilepsy	Neuroimage: Clinical	Manual corrections of preprocessing	y	T1, FLAIR	Artificial neural networks	REG:Freesurfer: resampling; IN; BFC; BE:ref	n/a	n/a	n	n	FCD	EP	22 + 28 healthy	3 (Great Ormond Street Hospital)	n	Expert segmentation		ROC; Sen
J	2017	P. Sivakumar and P. Ganeshkumar	An efficient automated methodology for detecting and segmenting the ischemic stroke in brain MRI images	International Journal of Imaging Systems and Technology		n/a	n/a	Graph Cut	enhancement: histogram equalization; Gabor transform	2.4GHz Core-2-Duo 4GB RAM	n/a	normal, abnormal	n	S	S	20 +25 healthy	1 (ISLES)	y	-		Sen; Spe; Acc; PPV; NPV; VC
J	2017	E. Ilunga–Mbuyamba et al.	Automatic selection of localized region-based active contour models using image content analysis applied to brain tumor segmentation	Computers in Biology and Medicine		n/a	n/a	K-Nearest Neighbours, Random Forest, Active Contour,	n/a	n/a	n/a	n	n	BT	LGG, HGG	21	1 (BraTS'12); 3 (University Hospital of Leipzig, Germany)	y	Manual, 1		DSC; Hdff
J	2017	S. Valverde et al.	Improving automated multiple sclerosis lesion segmentation with a cascaded 3D convolutional neural network approach	NeuroImage		y	T1, T2, PD, FLAIR	Artificial neural networks	BE:BET; BFC:N3; reg:SPM	32 GB RAM	n/a	n	y	MS	MS	45; 60	1 (MS GC MICCAI'08); 3 (Hospital Vall d'Hebron, Barcelona, Spain)	y	Semiautomatic		VD; TPR; FPR; DSC; PPV
J	2017	A. S. Keçeli et al.	A GPU-Based Approach for Automatic Segmentation of White Matter Lesions	IETE Journal of Research		n	FLAIR	Fuzzy C-Means, Region Growing	BE:morphological/watershed/ACM; enhancement: histogram equalization	GPU	<1 sec; ~5-10 sec	n	n	WML	-	10	3 (Hacettepe University Medical School Hospital)	n	Expert segmentation		SI; TPR; FPR
J	2016	B. H. Menze et al	A generative probabilistic model and discriminative extensions for brain lesion segmentation – with application to tumor and stroke	IEEE Trans Med Imaging		y	T1, T1c, T2, FLAIR	Markov Random Field, Watershed , Expectation-Maximization Gausian mixture model,	BFC:polynomial spline model;IN:linear template matching	n/a	n/a	n	n	BT; S	LGG; HGG	45; 18	1 (BraTS'12); 1 (BraTS'13)	y	-		DSC
J	2016	M. Strumia et al.	White Matter MS-Lesion Segmentation Using a Geometric Brain Model	IEEE Transactions on Medical Imaging		y	T1, FLAIR	Expectation-Maximization Gausian mixture model	REG:ITK; BE:BET; BFC:ref	n/a	3-7 hrs	n	n	MS	MS	42; 20	1 (MS GC MICCAI'08); 3	y	Manual, 1&2		VD; avgSD; TPR; FPR; DSC
J	2016	L. Griffanti et al.	BIANCA (Brain Intensity AbNormality Classification Algorithm): A new tool for automated segmentation of white matter hyperintensities	NeuroImage	Scan-rescan reproducibility	y: flexible to the number of input sequences	T1, FLAIR	K-Nearest Neighbours	BE: BET; REG:FLIRTIN: variance scaling	3GHz Intel Xeon	12-13 min	n	y	WML	neurodegenerative and vascular disorders	85; 474 + 20 longitudinal	3 (OPTIMA, Zamboni et al., 2013); 3 (OXVASC, Rothwell et al., 2004)	y	Manual, 3		DSC; FPR; FNR; DER; OER; ICC; Bland-Altman
J	2016	M. Cabezas et al.	Improved Automatic Detection of New T2 Lesions in Multiple Sclerosis Using Deformation Fields	American Journal of Neuroradiology		n	T1, PD; FLAIR	Thresholding	BE:BET2; BFC:N4; IN:histogram matching	n/a	n/a	n	n	MS	CIS; early MS	36x2	3	n	Semiautomatic		avgSD; DSC; VO; other
J	2016	Z. Karimaghaloo et al.	Adaptive multi-level conditional random fields for detection and segmentation of small enhanced pathology in medical images	Mecidal image Analysis		y	T1, T1c, T2, PD, FLAIR	Conditional Random Field	BE:BET; BFC:N3; IN: histogram matching	n/a	n/a	n	n	MS	-	1760; 2770	3	y	Manual, 2	consensus	Sen; FDR
J	2016	M. Ghafoorian et al.	Automated detection of white matter hyperintensities of all sizes in cerebral small vessel disease: Automated detection of white matter hyperintensities of all sizes	Medical Physics		y	T1, FLAIR	Random Forest, Boosting	REG: FLIRT; BE:BET; BFC:FSL-FAST; IN:histogram matching	n/a	n/a	n	n	WML	SVD	503	3 (RUNDMC, van Norden et al. 2011)	n	Manual, 1-3	supervision	FROC
J	2016	Y. Liu et al.	Automatic metastatic brain tumor segmentation for stereotactic radiosurgery applications	Physics in Medicine and Biology		n	T1c	Thresholding, Active Contour	BE:ROBEX	E3-1505M CPU 32GB RAM	<7 min	n	n	MET	-	15?	3 (University of Texas Southwestern Medical Center)	y	Manual, 1		DSC; SI; Hdff; avgSSD; sdSSD; other

For peer review only

J	2016	T. Brosch et al.	Deep 3D Convolutional Encoder Networks With Shortcuts for Multiscale Feature Integration Applied to Multiple Sclerosis Lesion Segmentation	IEEE Transactions on Medical Imaging		y	T1, T2, PD, FLAIR	Artificial neural networks	BE:BET; IN:0-1 range; REG:rigid; cropping	GeForce GTX 780	<1 sec	n	n	MS	-	43; 21; 195x2+n (377)	1 (MS GC MICCAI'08); 1 (ISBI'15); 3	y	Semiautomatic		DSC; VD; TPR; FPR
J	2016	D. Pustina et al.	Automated segmentation of chronic stroke lesions using LINDA: Lesion identification with neighborhood data analysis	Human Brain Mapping	Segmentation failure (low DSC)	n	T1	Random Forest	denoising:edge-preserving anisotropic algorithm; BFC:N4; BE:antsBrainExtraction	Xeon E4-2450, 2.1GHz 25GB RAM	3 hrs	n	n	S	S	60; 45	3	y	Manual, 1		DSC; Hdff; Sen; PPV; VC; other
J	2016	M. Goetz et al.	DALSA: Domain Adaptation for Supervised Learning From Sparsely Annotated MR Images	IEEE Transactions on Medical Imaging		y	T1, T1c, FLAIR, DTI	Random Forest	REG:rigid; BE; IN	GeCONiI	70 sec	n	y	BT	HGG	19; 30	1 (BraTS'13); 3	y	Manual, 1		DSC; Sen; Spe
J	2016	K. Thiruvenkadam and N. Perumal	Fully automatic method for segmentation of brain tumor from multimodal magnetic resonance images using wavelet transformation and clustering technique	International Journal of Imaging Systems and Technology		y	T1c, T2, FLAIR	Fuzzy C-Means, Discrete Wavelet Transform	sharpening:Haar WT	Intel Pentium Core Duo 1.6 GHz 512 MB RAM	2-3 min	n	n	BT	LGG; HGG	30	1 (BraTS'12)	y	-		DSC; Sen; PPV; Kappa
J	2016	R. Mechrez et al.	Patch-based Segmentation with Spatial Consistency: Application to MS Lesions in Brain MRI	Journal of Biomedical Imaging		y	T1, T2, FLAIR	K-Nearest Neighbours, Thresholding	subsampling; IN: histogram matching; BFC:N4; BE:BrainSuite13; tissue maps	i7 3.4GHz 8GB RAM	4 min	n	n	MS	-	65	1 (MS GC MICCAI'08)	y	-		TPR; PPV; DSC
J	2016	P. Korfiatis et al.	Automated Segmentation of Hyperintense Regions in FLAIR MRI Using Deep Learning	Tomography	Inter-rater agreement evaluated	n	FLAIR	Artificial neural networks	BE:atlas-based; BFC:N4; IN:histogram matching	NVIDIA K2	1 hr	n	n	BT	LGG; HGG	186; 135	1 (BraTS'15); 3		Manual, 3		DSC; Jcc; FPR; TPR
J	2016	A. M. Pagnozzi et al.	Automated, quantitative measures of grey and white matter lesion burden correlates with motor and cognitive function in children with unilateral cerebral palsy	NeuroImage: Clinical		y	T1, T2	Expectation-Maximization, Markov Random Field, Thresholding	BFC:N4; REG:affine; denoising:ADF; BE:in-house; tissue probability maps reg:free-form	n/a	n/a	n	n	CP	-	125	3 (University of Queensland Cerebral Palsy and Research Rehabilitation Centre)	y	Manual		Sen; Spe; Acc; FPR; FNR
J	2016	X. Qu et al.	Positive Unanimous Voting Algorithm for Focal Cortical Dysplasia Detection on Magnetic Resonance Image	Front. Comput. Neurosci		n	T1	Bayesian classification, Other	IN:histogram matching + 0-225 range; BE:BET; BFC:EM; resampling; tri-linear interpolation; REG:rigid; masking	n/a	n/a	n	n	FCD	-	10 + 31 controls	3 (Ghent University Hospital)	n	Manual, 1		TPR; FPR; F-score
J	2016	S. Banerjee et al.	A Novel GBM Saliency Detection Model Using Multi-Channel MRI	PloS one		y	T1c T2, FLAIR	Other	n/a	I7 3.4GHz 16GB RAM	n/a	n	n	BT	LGG; HGG	30 +50 simulated	1 (BraTS'12)	y	-		ROC
J	2016	L. Zhao and K. Jia	Multiscale CNNs for Brain Tumor Segmentation and Diagnosis	Computational and Mathematical Methods in Medicine		y	T1, T1c, T2, FLAIR	Artificial neural networks	n/a	n/a	n/a	n	n	BT	LGG; HGG	30 +50 simulated	1 (BraTS'13)	y	-		DSC
J	2016	P. G. L. Freire and R. J. Ferrari	Automatic iterative segmentation of multiple sclerosis lesions using Student's t mixture models and probabilistic anatomical atlases in FLAIR images	Computers in Biology and Medicine	Inter-rater agreement evaluated	n	FLAIR	Other	denoising:non-local means; BFC:N4; REG:non-rigid	i7 16GB RAM	~20 min	n	n	MS	-	5x4/5	1 (MSC'15)	y	-		DSC; Sen; FPR; VD; VC
J	2016	S. Pereira et al.	Brain Tumor Segmentation Using Convolutional Neural Networks in MRI Images	IEEE Transactions on Medical Imaging		y	T1, T1c, T2, FLAIR	Artificial neural networks	BFC:N4; IN:histogram matching	n/a	n/a	n	n	BT	LGG; HGG	65; 327	1 (BraTS'13); 1 (BraTS'15)	y	-		DSC; PPV; Sen
J	2016	N. Cordier et al.	A Patch-Based Approach for the Segmentation of Pathologies: Application to Glioma Labelling	IEEE Transactions on Medical Imaging		y	T1, T1c, T2, FLAIR	Other	REG:affine; resampling; IN:1-99% clipping + u & v scaling; tissue maps:FSL FAST	2.66GHz 6GB RAM	up to 16 min	n	n	BT	LGG; HGG	304?	1 (BraTS'13); 1 (BraTS'14)	y	-		DSC; Hdff
J	2016	M. J. Fartaria et al.	Automated detection of white matter and cortical lesions in early stages of multiple sclerosis	Journal of Magnetic Resonance Imaging		y	T1, FLAIR, MP2RAGE, DIR	K-Nearest Neighbours, Region Growing	REG:rigid; BE:MorphoBox; BFC:N4; IN:histogram mtaching; tissue maps	n/a	n/a	n	n	MS	MS	39	3	n	Manual, 2	consensus	DSC; Sen; Spe; Acc; Bland-Altman; other
J	2016	J. C. Griffis et al.	Voxel-based Gaussian naïve Bayes classification of ischemic stroke lesions in individual T1-weighted MRI scans	Journal of Neuroscience Methods		n	T1	Bayesian classification	tissue map:SPM12; BFC:SPM12; REG:SPM12	n/a	n/a	n	y	S	S	30	3 (Cincinnati Children's Hospital Medical Center)	y	Semiautomatic		DSC; VD; VC
J	2016	Y. Li et al.	Brain tumor segmentation from multimodal magnetic resonance images via sparse representation	Artificial Intelligence in Medicine		y	T1, T1c, T2, FLAIR	Sparse representation, Markov Random Field, Graph Cut,	BFC:N3; IN:histogram matching + 0-1 range	i7 1.8GHz 64 GB RAM	2-5 min	n	n	BT	LGG; HGG	64	1 (BraTS'13)	y	-		DSC; PPV; Sen; Kappa
J	2016	J. R. Stone et al.	Supervised learning technique for the automated identification of white matter hyperintensities in traumatic brain injury	Brain Injury		y	T1, T2, FLAIR	Random Forest, Markov Random Field	convert to nii; REG:rigid; denoising:ref; BFC:N4; IN:0-1 range; BE:ANTs	n/a	n/a		y	WML	TBI	24	3 (Chronic Effects of Neurotrauma Consortium, Walker et al. 2016)	n	Manual, 1		Sen; PPV; F1; VD
J	2015	V. G. Kanas et al.	A low cost approach for brain tumor segmentation based on intensity modeling and 3D Random Walker	Biomedical Signal Processing and Control	Segmentation failure	y	T1, T1c, T2, FLAIR	Random Walker	denoising; BFC; REG:FSL; BE:BET	3GHz 8GB RAM	<1 min	n	n	BT	LGG; HGG	34; 23	1 (BraTS'13); 3 (Prastawa et al. 2009); 3	y	Manual, 1		FPR; TRP; DSC; Hdff
J	2015	O. Commowick et al.	Diffusion MRI abnormalities detection with orientation distribution functions: A multiple sclerosis longitudinal study	Medical Image Analysis		n	DTI	Other	Movement correction; distortion correction; tensor and ODF estimation	n/a	n/a	n	n	MS	CIS	15x2	3	n	Manual, 1		other
J	2015	D. Guo et al.	Automated lesion detection on MRI scans using combined unsupervised and supervised methods	BMC Medical Imaging		n	T1	Support Vector Machine, Other	enantiomorphic normalization	I7 2.2GHz 16GB RAM	388 min	n	n	S; BT	S; G	60; 30	1 (BraTS'12); 3	y	Manual, 3	consensus	Acc; PPV; Sen; DSC
J	2015	P. K. Roy et al.	Automatic white matter lesion segmentation using contrast enhanced FLAIR intensity and Markov Random Field	Computerized Medical Imaging and Graphics		y	T1, FLAIR	Random Forest, Markov Random Field	REG:rigid(SPM8); BE:BET; BFC:N3	n/a	n/a	n	n	WML	HT	24; 20	1 (MS GC MICCAI'08); 3 (ENVISion, Reid et al. 2012)	y	Manual, 2		FROC; DSC; TPR; PPV
J	2015	E. Roura et al.	A toolbox for multiple sclerosis lesion segmentation.	Neuroradiology		y	T1, FLAIR	Thresholding	BE:BET; denoising:ADF;BFC:N3;REG:affine(SPM8/12)	n/a	n/a	n	y	MS	CIS	70; 20 (+23 test online); 14	1 (MS GC MICCAI'08); 3 (Hospital Vall d'Hebron, Spain); 3 (Clínica Girona, Spain)	y	Manual, multiple		DSC; TPR; PPV

For peer review only

J	2015	N. Guizard et al.	Rotation-invariant multi-contrast non-local means for MS lesion segmentation	NeuroImage: Clinical		y	T2, FLAIR	Non-local Means	denoising:NLM; BFC:N3; IN: histogram matching; REG:rigid; BE:ref	i7 3.06 GHz	40 min	n	planned	MS	-	108; 20 (+23 test online)	1 (MS GC MICCAI'08); 3	y	Semiautomatic		DSC; Sen; PPV; VDf; FPR; SSD; TPR; PPV; VR
J	2015	S. Jain et al.	Automatic segmentation and volumetry of multiple sclerosis brain lesions from MR images	NeuroImage: Clinical	Scan-rescan reproducibility	y	T1, FLAIR	Other	REG:rigid; BE:MNI brain mask reg(affine+non-rigid); tissue maps	n/a	n/a	n	n	MS	-	20; 10x2	3 (VU University Medical Center, Amsterdam, the Netherlands); 3 (UZ Brussels, Brussels, Belgium)	y	Manual, 3	consensus	DSC; ICC; absVD; Sen; PPV
J	2015	R. Harmouche et al.	Probabilistic Multiple Sclerosis Lesion Classification Based on Modeling Regional Intensity Variability and Local Neighborhood Information	IEEE Transactions on Biomedical Engineering		y	T1, T2, PD, FLAIR	Other	BFC:N3; REG:ref; BE:BET; IN:histogram matching	Optiplex 980 quad-core	2 min	n	n	MS	-	100	3	y	Manual, 1&5	majority	DSC; Sen; PPV
J	2015	Z. Karimaghaloo et al.	Temporal Hierarchical Adaptive Texture CRF for Automatic Detection of Gadolinium-Enhancing Multiple Sclerosis Lesions in Brain MRI	IEEE Transactions on Medical Imaging		y	T1, T1c, T2, PD, FLAIR	Conditional Random Field	REG:ref; BE:BET; BFC:N3; IN:histogram matching	2.66GHz	56 sec	treatment; placebo	n	MS	-	1190x2; 120x2; 69x4	3	y	Manual, 2	consensus	Sen; FPR
J	2015	T. Zhan et al.	A novel brain tumor segmentation method for multi-modality human brain MRIs	International Journal of Multimedia and Ubiquitous Engineering		y	T1, T1c, T2, FLAIR	Bayesian classification, Logistic Regression	REG:rigid; BFC:N3; BE	n/a	n/a	n	n	BT	-	10 +10 simulated	1 (BraTS'12)	y	-		Jcc
J	2015	M. P. Arakeri and G. R. M. Reddy	Computer-aided diagnosis system for tissue characterization of brain tumor on magnetic resonance images	SIViP	Inter-rater agreement evaluated	y	T1c, T2	Fuzzy C-Means	denoising:median filtering; contrast enhnacement:histogram equalization; REG:FLIRT; BE:Otsu's thresholding	3GHz 3GB RAM	1 sec	benign, malignant	n	BT	-	550	3 (Shirdi Sai Cancer Hospital, Manipal, India)	n	Manual, 2		Hdff; VO; avgSD
J	2015	A. Demirhan et al.	Segmentation of Tumor and Edema Along With Healthy Tissues of Brain Using Wavelets and Neural Networks	EEE journal of biomedical and health informatics		y	T1, T2, FLAIR	Self Organising Maps	BE: thresholding + morphological operations; IN:0-1 range; denoising:ADF; REG:affine	n/a	n/a	n	n	BT	G	20	1 (BraTS'12); 3 (Mayıs Hospital in Ankara, Turkey)	y	Manual		DSC; Sen; Spe
J	2015	C. H. Sudre et al.	Bayesian Model Selection for Pathological Neuroimaging Data Applied to White Matter Lesion Segmentation	IEEE Transactions on Medical Imaging		y	T1, FLAIR; T2, PD, FLAIR	Expectation-Maximization Gausian mixture model, Markov Random Field	BFC:in house; REG; IN:0-1 range; BE:ref	n/a	n/a	n	n	WML	MS; DM	20	1 (MS GC MICCAI'08)	y	-		DSC; TPR; FNR; VD; AD; DE; OER
J	2015	B. Ahmed et al.	Cortical feature analysis and machine learning improves detection of 'MRI-negative' focal cortical dysplasia	Epilepsy & Behavior		n	T1	Linear regression, Bagging	BFC; BE:ref; FreeSurfer	n/a	n/a	n	n	FCD	FCD	31 (24 MRI negative) + 62 controls	3 (New York University School of Medicine Comprehensive Epilepsy Center)	n	Manual, 2	supervision	TPR; FPR; DSC; avgSD
J	2015	S. D. S. Al-Shaikhli et al.	Brain tumor classification and segmentation using sparse coding and dictionary learning	Biomedical Engineering / Biomedizinische Technik		n	FLAIR	Graph Cut, Dictionary Learning	BE:BET; BFC:MIPAV	n/a	n/a	normal, abnormal	n	BT	GBM; G; MET	30; 30; 30; 30	1 (BraTS'13); 1 (IBSR); 1 (SPL)	y	-		DSC; PPV; Sen; Jcc; Kappa
J	2015	P. Szwarc et al.	Automatic brain tumour detection and neovasculature assessment with multiseries MRI analysis	Computerized Medical Imaging and Graphics	Inter-rater agreement evaluated	y	T1, T1c, FLAIR, RCBV	Fuzzy C-Means	REG; interpolation: nearest neighbour; BE:Otsu's thresholding; symmetry plane detection	n/a	n/a	n	n	BT	-	10; 10	3	y	Manual, 1&2		DSC; Sen; Spe
J	2015	J. Juan-Albarracín et al.	Automated Glioblastoma Segmentation Based on a Multiparametric Structured Unsupervised Classification	PloS one		y	T1, T1c, T2, FLAIR	Markov Random Field	denoising:ref; BE:Brain Suite Software; BFC:N4; resampling:ref	Intel Xeon E5-2620 64GB RAM	~39 min	n	n	BT	-	31	1 (BraTS'13)	y	-		DSC; PPV; Sen; Kappa
J	2015	B. Ozenne et al.	Spatially regularized mixture model for lesion segmentation with application to stroke patients	Biostatistics		y	T1, FLAIR	Markov Mixture Models	REG; tissue maps	n/a	15 min	n	upon request	S	S	12	3 (I-KNOW)	n	Manual, 3	majority	other
J	2015	X. Tomas-Fernandez and S. K. Warfield	A Model of Population and Subject (MOPS) Intensities With Application to Multiple Sclerosis Lesion Segmentation	IEEE Transactions on Medical Imaging		y	T1, T2, FLAIR	Expectation-Maximization Gausian mixture model, Graph Cut	BFC:ref; denoising:ref	n/a	n/a	n	n	MS	MS	3 (synthetic); 51	1 (MS GC MICCAI'08)	y	-		TPR; FPR; Extra Fraction; Miss Fraction; PPV
J	2015	I. Njeh et al.	3D multimodal MRI brain glioma tumor and edema segmentation: A graph cut distribution matching approach	Computerized Medical Imaging and Graphics		y	T1, T1c, T2, FLAIR	Other, Graph Cut	BRATS only	n/a	<0.5 sec	n	n	BT	LGG; HGG	45 +65 simulated	1 (BraTS'12)	y	-		DSC
J	2015	O. Maier et al.	Extra Tree forests for sub-acute ischemic stroke lesion segmentation in MR sequences	Journal of Neuroscience Methods	Errors in preprocessing, Segmentation failure	y – adaptable to single and multi	T1, T2, FLAIR, DWI	Random Forest	resampling; REG:rigid(elastix); BE:BET; BFC:Bies Field Correction Tool; IN:histogram matching	n/a	n/a	n	n	S	S	37	3 (Machner et al. 2012)	n	Manual, 1		DSC; avgSSD; Hdff; PPV; Sen
C	2015	O. Maier et al.	MS lesion segmentation in MRI with random forests	2015 Longitudinal Multiple Sclerosis Lesion Segmentation Challenge	Inter-rater agreement evaluated	n/a	n/a	Random Forest	IN:MedPy	n/a	n/a	n	n	MS	-	5x4	1 (MSC'15)	n	-		DSC; avgSSD; TPR. FPR
J	2015	T. C. Steed et al.	Iterative Probabilistic Voxel Labeling: Automated Segmentation for Analysis of The Cancer Imaging Archive Glioblastoma Images	American Journal of Neuroradiology	Inter-rater agreement evaluated	y	T1, T1c, T2, FLAIR	K-Nearest Neighbours, Thresholding	BFC:FAST; REG:nonlinear + affine; BE:BET+ROBEX; tissue maps	n/a	11 min	n	n	BT	-	40	2 (TCIA, Clark et al. 2013)	y	Manual, 3	supervision	DSC
J	2015	R. Wang et al.	Automatic segmentation and volumetric quantification of white matter hyperintensities on fluid-attenuated inversion recovery images using the extreme value distribution	Neuroradiology		n	FLAIR	Other	BFC:N3; IN:0-255 range; BE:BET	n/a	n/a	n	planned	WML	MS	60, 10	1 (MS GC MICCAI'08); 3	y	Manual, 2	consensus	DSC; FPR; FNR; OER; DER; Bland-altman; ICC; VC; SI
J	2015	P. Maji and S. Roy	SoBT-RFW: Rough-Fuzzy Computing and Wavelet Analysis Based Automatic Brain Tumor Detection Method from MR Images	Fundamenta Informaticae		n/a	n/a	Fuzzy C-Means, Region Growing	BE:S3	3.4GHz 16GB RAM	n/a	n	y	BT	-	25	1 (BraTS'12)	y	-		DSC; Sen; Spe
J	2015	N. Nabizadeh and M. Kubat	Brain tumors detection and segmentation in MR images: Gabor wavelet vs. statistical features	Computers & Electrical Engineering	Detection followed by segmentation	n	T1/FLAIR	Comparison	IN; histogram normalization	3GHz 64GB RAM	~5 min	n	n	BT	HGG	25 (unclear how many real and how many simulated)	1 (BraTS'13)	y	-		Sen; Spe; Acc

For peer review only

J	2014	N. Gonçalves et al.	Self-supervised MRI tissue segmentation by discriminative clustering	Int. J. Neur. Syst		y	T1, T2, PD; T1 T2, FLAIR	Self Organising Maps	REG:SPM5; BE:BET2	n/a	n/a	n	n	MS	MS	2; 54	1 (MS GC MICCAI'08)	y	-		DSC; other
J	2014	Y. Zhong et al.	Automated White Matter Hyperintensity Detection in Multiple Sclerosis Using 3D T2 FLAIR	Journal of Biomedical Imaging		n	FLAIR	Other	BE:in-house; BFC:modified homodyne HP filter; tissue maps:thresholding	I7 2.8GHz 8GB RAM	5 min	n	n	MS	-	26	3 (Synergy Health Concepts, CA)	n	Manual		SI; VC
J	2014	Y.-H. Mah et al.	A new method for automated high-dimensional lesion segmentation evaluated in vascular injury and applied to the human occipital lobe	Cortex		n	DWI	Other	REG:SPM5	n/a	n/a	n	y	S	-	38 + 95 controls	3	n	Manual, 1		DSC; Sen; Spe
J	2014	B. I. Yoo et al.	Application of variable threshold intensity to segmentation for white matter hyperintensities in fluid attenuated inversion recovery magnetic resonance images	Neuroradiology		n	FLAIR	Thresholding	BFC:SPM8; BE:SPM8	n/a	n/a	n	n	WML	-	48	3	y	Manual, 2		DSC; Sen; Spe; Jcc; ICC
J	2014	M. Cabezas et al.	Automatic multiple sclerosis lesion detection in brain MRI by FLAIR thresholding	Computer Methods and Programs in Biomedicine		n	FLAIR	Expectation-Maximization, Thresholding	BE:BET; denoising:ADF; BFC:N4; REG:affine	QuadCore 2.3 GHz, 16 Gb RAM	several min	n	n	MS	-	45	3 (Hospital Vall d'Hebron); 3 (Hospital Josep Trueta); 3 (Clínica Girona)	y	Semiautomatic		TPR; FPR; DSC; avgSD
J	2014	J.-Z. Tsai et al.	Automated Segmentation and Quantification of White Matter Hyperintensities in Acute Ischemic Stroke Patients with Cerebral Infarction	PloS one		y	T1, DWI, FLAIR	Thresholding, Other	REG:rigid(MNI); tisue maps:SPM8; IN:1-100 range; BE:BET	i5 2.67GHz 4GB RAM	~15 min	n	n	WML	S	30	3 (Taiwan Stroke Registry, Hsieh, et al. 2010)	n	Semiautomatic		SI; ICC; Bland-Altman; Sen; Spe
J	2014	P. Dvořák et al.	Unsupervised Pathological Area Extraction using 3D T2 and FLAIR MR Images	Measurement Science Review	No preprocessing	y	T2, FLAIR	Other	Mid-sagittal plane extraction	n/a	<1 min	n	n	BT	LGG; HGG	30 +50 simulated	1 (BraTS'12)	y	-		DSC; Acc
J	2014	Y. Lu et al.	Multimodal Brain-Tumor Segmentation Based on Dirichlet Process Mixture Model with Anisotropic Diffusion and Markov Random Field Prior	Computational and Mathematical Methods in Medicine		y	T1c, FLAIR	Markov Random Field, Other	denoising: ADF	n/a	n/a	n	n	BT	LGG; HGG	55 + 65 synthetic	1 (BraTS'12)	y	-		DSC; Jcc; Sen; Spe
J	2014	R. Wang et al.	Automatic segmentation of white matter lesions on magnetic resonance images of the brain by using an outlier detection strategy	Magnetic Resonance Imaging		y – adaptable to single and multi	T1, T2, FLAIR	Expectation-Maximization Gausian mixture model, Other	BFC:N3; BE:BET; REG:demons registration	n/a	2.2 sec per slice	n	n	WML	-	86 + synthetic	3	n	Manual, 2		DSC; Overlap Fraction; Extra Fraction; VC; Band-Altman
J	2014	M. Huang et al.	Brain Tumor Segmentation Based on Local Independent Projection-Based Classification	IEEE Transactions on Biomedical Engineering		y	T1, T1c, T2, FLAIR	Other	BFC:N3; IN:1-99% clipping + 0-100 range	I5 3.1GHz	26 min	n	n	BT	LGG; HGG	55 +65 synthetic	1 (BraTS'12); 1 (BraTS'13)	y	-		DSC; Jcc; FPR; FNR
J	2014	J. Mitra et al.	Lesion segmentation from multimodal MRI using random forest following ischemic stroke	NeuroImage		y	T1, T2, FLAIR, DWI (ADC)	Markov Random Field, Random Forest	REG:rigid; tissue masks:ref; BE: masking	3.2GHz 23.5GB RAM	27 min	n	n	S, WML	S	36	1 (MS GC MICCAI'08); 3	y	Manual, 1		DSC; PPV; TPR; avgSSD; VD
J	2014	J.-Z. Tsai et al.	Automatic Detection and Quantification of Acute Cerebral Infarct by Fuzzy Clustering and Histographic Characterization on Diffusion Weighted MR Imaging and Apparent Diffusion Coefficient Map	BioMed Research International		n	DWI (ADC)	Fuzzy C-Means, Thresholding, edge detection,	reg:rigid + trilinear interpolation NMI; IN:0-1 range; BE:BET; histogram smoothing:3rd order moving avg filter	i5 2.67GHz 4GB RAM	5 min	n	n	S	S	22	3 (Taiwan Stroke Registry, Hsieh, et al. 2010)	n	Semiautomatic		DSC; Kappa; ICC; Sen; Spe; PPV
J	2014	J. Zhang et al.	A fully automatic extraction of magnetic resonance image features in glioblastoma patients	Med Phys		y	T1, T1c, FLAIR	Random Forest	REG:rigid (FLIRT); denoising:median filtering	n/a	n/a	n	n	BT	GBM	73	2 (TCIA, Clark et al. 2013)	n	Manual, 1		DSC
J	2014	O. Ganiler et al.	A subtraction pipeline for automatic detection of new appearing multiple sclerosis lesions in longitudinal studies	Neuroradiology		y	T1, T2, PD	Thresholding, Other	BE:BET; REG:affine + rigid; BFC:N4; IN:histogram matching; tissue map	n/a	n/a	n	n	MS	MS	20x2	3	n	Manual, 2	supervision	Sen; FPR; DSC
J	2014	G. Erus et al.	Individualized statistical learning from medical image databases: Application to identification of brain lesions	Medical Image Analysis	Inter-rater agreement evaluated	n/a	n/a	Other	BE:BET; BFC: N3; IN:histogram matching; REG:non-lin(HAMMER)	n/a	n/a	n	n	WML, S	DM	80	3	n/a	Manual, 1&2		DSC; Sen; Spe
J	2014	E. M. Sweeney et al.	A Comparison of Supervised Machine Learning Algorithms and Feature Vectors for MS Lesion Segmentation Using Multimodal Structural MRI	PloS one	Raters' years of experience given	y	T1, T2, FLAIR	Comparison	REG:rigd; BFC:N3; BE:SPECTRE; CSF removal:15th percentile	1 core	<1 min	n	n	MS	MS	98	3	y	Manual, 2	supervision	ROC; DSC
J	2013	G. Liberman et al.	Automatic multi-modal MR tissue classification for the assessment of response to bevacizumab in patients with glioblastoma	European Journal of Radiology	Treatment response evaluation	y	T1, T1c, T2, T2*, T2*c, FLAIR	Expectation-Maximization Gausian mixture model, K-Nearest Neighbours	BFC:N3; REG:SPM; IN:multiplicative model; BE:BET	n/a	n/a	n	n	BT	GB	59	3	n	Manual		Sen; Spe; VC; other
J	2013	E. M. Sweeney et al.	Automatic Lesion Incidence Estimation and Detection in Multiple Sclerosis Using Multisequence Longitudinal MRI	American Journal of Neuroradiology		y	T1, T2, PD, FLAIR	Other, Logistic Regression	resampling; REG: rigid; BE:ref; tissue maps	n/a	n/a	n	n	MS	-	10x11	3	n	Manual, 1		ROC; Volume Change
J	2013	S. Ji et al.	Automatic segmentation of white matter hyperintensities by an extended FitzHugh & nagumo reaction diffusion model	Journal of Magnetic Resonance Imaging		n	FLAIR	Other	BE:BET; denoising:ADF	n/a	n/a	n	n	WML	-	127	3	y	Manual, 3	consensus	DSC
J	2013	C. Elliott et al.	Temporally Consistent Probabilistic Detection of New Multiple Sclerosis Lesions in Brain MRI	IEEE Transactions on Medical Imaging		y	T1, T2, PD, FLAIR	Bayesian classification, Markov Random Field	BE:BET; BFC:N3; REG:rigid; IN:histogram matchingl + least trimmed squares	n/a	n/a	n	n	MS	-	684 (155 participants)	3	y	Semiautomatic		Sen; FD
J	2013	E. M. Sweeney et al.	OASIS is Automated Statistical Inference for Segmentation, with applications to multiple sclerosis lesion segmentation in MRI	NeuroImage: Clinical	Inter-rater agreement evaluated	y	T1, T2, PD, FLAIR	Logistic Regression	REG:rigid; BFC:N3; BE:SPECTRE; IN:0u1v; smoothing	n/a	n/a	n	n	MS	-	98 + 33 healthy; 20x2, 128 + 1 healthy	3	y	Manual, 1		ROC; Sen; DSC
J	2013	K. Mouridsen et al.	Acute Stroke: Automatic Perfusion Lesion Outlining Using Level Sets	Radiology	Inter-rater agreement evaluated	y	PWI, DWI	Level Set	REG:linear	n/a	n/a	n	n	S	S	14	3	n	Manual, 4	consensus	Bland – Altman; DSC
J	2013	A. Resmi et al.	A novel automatic method for extraction of glioma tumour, white matter and grey matter from brain magnetic resonance images	Biomedical Imaging and Intervention Journal	Inter-rater agreement evaluated	y	T1, FLAIR, T2	Thresholding, Other	IN:0-1 range; BE:morphological operations	n/a	1 sec	n	n	BT	LGG, HGG	20 +50 synthetic	3 (Sree Chitra Institute of Medical Sciences and Technology); 3 (Regional Cancer Centre, Thiruvananthapuram, Kerala, India)	y	Manual, 1		PPV; Jcc; other
J	2013	A. Bijar et al.	Increasing the Contrast of the Brain MR FLAIR Images Using Fuzzy Membership Functions and Structural Similarity Indices in Order to Segment MS Lesions	PloS one		n	FLAIR	Thresholding, Genetic Algorithm	n/a	2.5 GHz 512 MB RAM	<30 sec	n	n	MS	MS	20	3 (Khayati et al. 2008)	n	Manual, 2	consensus	DSC; Sen; Extra Fraction; ICC
J	2013	R. Simões et al.	Automatic segmentation of cerebral white matter hyperintensities using only 3D FLAIR images	Magnetic Resonance Imaging		n	FLAIR	Expectation-Maximization Gausian mixture model	BE:BET; BFC:FAST	n/a	n/a	n	n	WML	MCI; progressive MCI	25 + 15 healthy; 23	1 (MS GC MICCAI'08); 3 (University Hospital of Essen, Germany)	y	Manual, 1		DSC; Overlap Fraction; Extra Fraction; VC
J	2013	L. Shi et al.	Automated quantification of white matter lesion in magnetic resonance imaging of patients with acute infarction	Journal of Neuroscience Methods		y	T1, FLAIR, DWI	Other	REG:affine; IN:in house; BE:BET; tissue maps:REF	2.8GHz 12GB RAM	25 sec	n	n	WML	S	91(30 segmented)	3	y	Semiautomatic		DSC; Sen; PPV; ICC; Bland-Altman
J	2012	L. Weizman et al.	Automatic segmentation, internal classification, and follow-up of optic pathway gliomas in MRI	Medical Image Analysis	Indirect reproducibility evaluation	y	T1, T2, FLAIR	Other	REG:SPM; IN:dynamic histogram warping	n/a	n/a	n	n	BT	optic pathway G	28	3	n	Manual, 2		VD; avgSSD; DSC; ROC
J	2012	Y. Wang et al.	Multi-stage segmentation of white matter hyperintensity, cortical and lacunar infarcts	NeuroImage		y	T1, T2, FLAIR	Expectation-Maximization Gausian mixture model, Region Growing	BFC:REF; BE:BET; REG: FLIRT; tissue map:FreeSurfer	n/a	n/a	WMH; CI; LI	n	WML	-	272	3 (Memory Aging & Cognition Center at the National University of Singapore)	n	Manual, 1-3	supervision	DSC; VC; Bland-Altman

For peer review only

J	2012	K. H. Ong et al.	Automatic white matter lesion segmentation using an adaptive outlier detection method	Magnetic Resonance Imaging		y	T1, FLAIR; T1, T2, FLAIR	Thresholding	BE:model-based level set; BFC:N3	n/a	n/a	n	n	WML	-	38; 23	1 (MS GC MICCAI'08); 3 (Universiti Sains Malaysia's hospital)	y	Manual, 1		VR; Jcc; DSC; Sen; FPR; VD
J	2012	T. Samaille et al.	Contrast-Based Fully Automatic Segmentation of White Matter Hyperintensities: Method and Validation	PloS one		y	T1, FLAIR	Thresholding	BE:SPM8; REG:rigid; BFC:SPM8	n/a	30 min	n	n	WML	MCI; CADASIL	24; 43	3 (Salpêtrière Hospital); 3 (Lariboisière Hospital)	y	Semiautomatic		ICC; Bland-Altman; VR; DSC
J	2012	B. A. Abdullah et al.	Multi-Sectional Views Textural Based SVM for MS Lesion Segmentation in Multi-Channels MRIs	Open Biomed Eng J		y	T1, T2, FLAIR	Support Vector Machine	Resampling; REG:AIR; IN:ref	n/a	n/a	n	n	MS	-	51 (+synthetic); 10	1 (MS GC MICCAI'08); 3 (University of Miami)	y	n/a		DSC; TPR; PPV; other
J	2012	P. Schmidt et al.	An automated tool for detection of FLAIR-hyperintense white-matter lesions in Multiple Sclerosis	NeuroImage		y	T1, FLAIR	Region Growing, Partial Volume Estimation	BFC:VBM8; tissue maps; REG:linear + non-linear	3.2 GHz 8GB RAM	13 min	n	planned	MS	-	52 + 10 (unclear)+ 18 control	3	n	Manual, 2	consensus	VC; VR; Sen; Spe; Acc; DSC
J	2012	A. Gooya et al.	GLISTR: Glioma Image Segmentation and Registration	IEEE Transactions on Medical Imaging		y	T1, T1c, T2, FLAIR	Expectation-Maximization, tumor growth, atlas registration,	BFC; BE; cerebelum removal; REG:affine	n/a	3-6 hs	n	n	BT	-	122 (10 segmented)	3 (University of Pennsylvania)	n	Manual, 1		DSC; SD
J	2014	D. Kwon et al.	Multimodal brain tumor image segmentation using glistr	MICCAI BRATS		y	T1, T1c, T2, FLAIR	Expectation-Maximization, tumor growth, atlas registration,	BFC; IN:0-255 range	I7 3.4 GHz	85 min	n	n	BT	LGG, HGG	35	1 (BraTS'13)	y	-		DSC; PPV; Sen
J	2012	K. Popuri et al.	3D variational brain tumor segmentation using Dirichlet priors on a clustered feature set	Int J CARS		y	T1, T1c, T2	Active Contour, Other	denoising:edge-preserving non-linear filter; IN:weighted least squares; BFC:N3; symmetrical alignment:rigid	n/a	n/a	n	n	BT	G	15	3	n	Manual, 1		Jcc; PPV; Sen; Hdff
J	2012	N. M. Saad et al.	Fully Automated Region Growing Segmentation of Brain Lesion in Diffusion-weighted MRI	IAENG International Journal of Computer Science		n	DWI	Thresholding, Region Growing, Other,	IN:0-1 range + gamma-law transformation; BE:thresholding + morphological operations	n/a	n/a	n	n	S, BT, abscess	-	23 (slices used)	3 (General Hospital of Kuala Lumpur)	n	Manual		Jcc; FPR; FNR; other
J	2012	M. de C. Alegro et al.	Computerized brain tumor segmentation in magnetic resonance imaging	Einstein (São Paulo)	Semiautomatic preprocessing	y	T1, T1c, FLAIR	Support Vector Machine	BFC:N3; denoising:ADF; REG:semiautomatic; BE:ref; IN:histogram matching	n/a	n/a	n	n	BT	T	11	3 (Universidade de São Paulo)	n	Manual 2		other
J	2012	A. Khademi et al.	Robust White Matter Lesion Segmentation in FLAIR MRI	IEEE Transactions on Biomedical Engineering		n	FLAIR	Thresholding, Partial Volume Estimation	BE:ref; bilateral filtering	n/a	n/a	n	n	WML	-	24	3	n	Manual, 1		DSC; Sen; Spe
J	2015	A. Khademi et al.	Multiscale Partial Volume Estimation for Segmentation of White Matter Lesions Using Flair MRI	2015 IEEE 12th International Symposium on Biomedical Imaging (ISBI)		n	FLAIR	edge content, Partial Volume Estimation	n/a	n/a	n/a	n	n	WML	-	30	3 (Khademi et al. 2012)	n/a	Manual, 1		DSC; Extra Fraction; Bland-Altman
J	2012	Zacharaki, E. and Bezerianos A.	Abnormality Segmentation in Brain Images Via Distributed Estimation	IEEE Transactions on Information Technology in Biomedicine		n/a		Other	BE:ref; BFC:ref; IN:histogram matching, 0-1 range + 0u1v; REG:deformable	Intel Core2 Duo CPU 2GHz	Min – hrs	n	n	WML	DM	33 +73 healthy	3 (ACCORD-MIND)	n/a	Manual, 1		ROC
J	2011	D. Garcia-Lorenzo et al.	Trimmed-Likelihood Estimation for Focal Lesions and Tissue Segmentation in Multisequence MRI for Multiple Sclerosis	IEEE Transactions on Medical Imaging	Inter-rater agreement evaluated	y	T1, T2, PD	Other	BFC:ref; reg:rigid; BE:BET	2.4GHz 4GB RAM	2 min	n	n	MS	MS	10; simulated	1 (BrainWeb); 3	n	Manual, 5	majority	DSC; ICC
J	2008	D. Garcia-Lorenzo et al.	A Robust Expectation-Maximization Algorithm for Multiple Sclerosis Lesion Segmentation	MICCAI Workshop: 3D Segmentation in the Clinic: A Grand Challenge II, MS Lesion Segmentation		y	T1, T2, FLAIR	Expectation-Maximization, outlier detection	BFC:ref; BE:BET	n/a	n/a	n	n	MS	MS	44	1 (MS GC MICCAI'08)	y	-		VD; AD; TP; FP
J	2011	E. Geremia et al.	Spatial decision forests for MS lesion segmentation in multi-channel magnetic resonance images	NeuroImage		y	T1, T2, FLAIR	Decision Tree	resampling; cropping; BFC:REF; IN:REF; REF:ref; tissue maps:INRIA	IBM e325 dual-Opterons246 2 GHz	n/a	n	n	MS	MS	45	1 (MS GC MICCAI'08)	y	-		TPR; PPV; VD; SD
J	2012	E. Geremia et al.	Spatial decision forests for glioma segmentation in multi-channel MR images	MICCAI Challenge on Multimodal Brain Tumor Segmentation		y	T1, T1c, T2, FLAIR	Decision Tree	IN:histogram matching	n/a	n/a	n	n	BT	LGG, HGG	30 (+25 synthetic)	1 (BrainWeb); 3	y	n/a		DSC; TPR; PPV
J	2011	N. Zhang et al.	Kernel feature selection to fuse multi-spectral MRI images for brain tumor segmentation	Computer Vision and Image Understanding		y	T2, PD, FLAIR	Support Vector Machine, Region Growing	REG:SPM	n/a	n/a	n	n	BT	-	8+3x5(?)	3	y	Manual, multiple		TP;FP; FN; other
J	2011	S. Jeon et al.	Fully automated pipeline for quantification and localization of white matter hyperintensity in brain magnetic resonance image	International Journal of Imaging Systems and Technology		y	T1, FLAIR	Other	BFC:N3; BE:BET; REG:affine; tissue masks:INSECT	n/a	n/a	n	n	WML	SVD	10 annotated (+35 not)	3 (Lee et al. 2011)	n	Manual, 1		DSC; Jcc; Bland-Altman; VR
J	2011	V. Harati et al.	Fully automated tumor segmentation based on improved fuzzy connectedness algorithm in brain MR images	Computers in Biology and Medicine		n	T1c	Other	denoising:ADF; head mask:thresholding; IN:0-255 range	3.2GHz, 1GB RAM	2.5 min	n	n	BT	T	10	3	n/a	Manual, 1		DSC; Spe; Extra fraction
J	2010	D. Selvathi et al.	Hybrid approach for brain tumor segmentation in magnetic resonance images using cellular neural networks and optimization techniques	International Journal of Computational Intelligence and Applications		y	T2, FLAIR	Artificial neural networks, Genetic Algorithm	IN:-1-1 range	3GHz 512MB RAM	n/a	n	n	BT	astrocytoma; MET; G; meningioma	30	3	n	Manual		Acc
J	2010	M. Scully et al.	An automated method for segmenting white matter lesions through multi-level morphometric feature classification with application to lupus	Frontiers in Human Neuroscience		y	T1, T2, FLAIR	Support Vector Machine	resampling:trilinear interpolation; REG:MI; BFC:ref; IN:histogram matching	n/a	n/a	n	n	WML	lupus	27	3 (The Mind Research Network, University of New Mexico Hospital)	n	Manual, multiple		Sen; Spe
J	2010	N. Shiee et al.	A topology-preserving approach to the segmentation of brain images with multiple sclerosis lesions	NeuroImage		y- adaptable	T1, FLAIR	Fuzzy Clustering, Other	REG:rigid	modern Linux workstation	~45 min	n	y	MS	MS	10	1 (BrainWeb); 3	n	Semiautomatic		DSC; TP; VC
J	2009	K. E. Emblem et al.	Automatic glioma characterization from dynamic susceptibility contrast imaging: Brain tumor segmentation using knowledge-based fuzzy clustering	Journal of Magnetic Resonance Imaging	Inter-rater agreement evaluated	y	T1c, T2, FLAIR	Fuzzy C-Means, Thresholding	REG:NMI (SPM5); IN:adaptive histogram equalization; BE:SPM5	Pentium 4 3.19GHz 3GB RAM	4 min	Survival: ow-risk; high-risk	n	BT	G	50	3	n	Manual, 4	majority	Sen; PPV; VC; ROC
J	2009	R. Beare et al.	Development and validation of morphological segmentation of age-related cerebral white matter hyperintensities	NeuroImage		y	T1, T2, FLAIR	boosting, Watershed	REG:SPM5; BFC:ref; IN:median and interquartile range scaling; tissue map	n/a	n/a	n	n	WML	-	247	3 (Tasmanian Study of Cognition and Gait)	n	Manual, 1; Semiautomatic		ICC; SI
J	2009	R. de Boer et al.	White matter lesion extension to automatic brain tissue segmentation on MRI	NeuroImage	Inter-rater agreement evaluated	y	T1, PD, FLAIR	K-Nearest Neighbours, Thresholding	REG:rigid; resampling; BE:ELASTIX; BFC:N3; IN:4-96% clipping + 0-1 range	n/a	n/a	n	n	WML	-	195 + 20	3 (Rotterdam Scan Study, de Leeuw et al. 2001)	n	Manual, 1&2		DSC; Sen; Extra Fraction; VD; other
J	2009	J. Nie et al.	Automated brain tumor segmentation using spatial accuracy-weighted hidden Markov Random Field	Computerized Medical Imaging and Graphics	Visual validation of preprocessing	y	T1, T2, FLAIR	Expectation-Maximization-Markov Random Field	REG; BE:BET	3GHz 2GB RAM	20-25 min	n	n	BT	G	15	3	n	Semiautomatic		Jcc; VO
J	2009	A. Akselrod-Ballin et al.	Automatic Segmentation and Classification of Multiple Sclerosis in Multichannel MRI	IEEE Transactions on Biomedical Engineering	Indirect reproducibility evaluation	y – adaptable to single and multi	T1, T2, PD and/or FLAIR	Decision Tree, Other	REG:during acquisition	Xeon PC 1.7GHz	<10 min	n	n	MS	MS	25 + 4x4	3 (cientific Institute Ospedale San Raffaele)	n	Manual, 2	consensus	DSC; Sen; Spe; Acc; VC
J	2008	R. Khayati et al.	Fully automatic segmentation of multiple sclerosis lesions in brain MR FLAIR images using adaptive mixtures method and markov random field model	Computers in Biology and Medicine		n	FLAIR	Markov Random Field, Mixture Model	IN:0-255 range; BE:in house	2.66GHz 512 MB RAM	21 sec	n	n	MS	-	20	3	n	Manual, 2		F1; Sen; Extra fraction; VC
J	2008	R. Khayati et al.	A novel method for automatic determination of different stages of multiple sclerosis lesions in brain MR FLAIR images	Computerized Medical Imaging and Graphics		n	FLAIR	Markov Random Field, Mixture Model	BE:ref	Pentium 4 2.66GHz 512 MB RAM	n/a	acute (recent/early); chronic	n	MS	MS	20	3	n	Manual, 2		DSC; Sen; Extra Fraction
J	2008	T. B. Dyrby et al.	Segmentation of age-related white matter changes in a clinical multi-center study	NeuroImage		y – adaptable to single and multi	T1, T2, FLAIR	Artificial neural networks	IN:polynomial fitting & z-score; REG:rigid(SPM2); resampling:b-spline interpolation; BE:SPM2; BFC:N3	n/a	n/a	n	n	WML	-	362	3 (LADIS, Pantoni et al. 2005)	y	Semiautomatic		SI; Sen

For peer review only

J	2008	J. F. A. Jansen et al.	White Matter Lesions in Patients With Localization-Related Epilepsy	Investigative Radiology		y	T1, T2, FLAIR	Other	REG; IN; BE:ref; tissue maps	n/a	n/a	n	n	WML	EP	33 + 16 healthy	3	n	Expert segmentation		ICC; VC
J	2008	E. Herskovits et al.	Automated Bayesian Segmentation of Microvascular White-Matter Lesions in the ACCORD-MIND Study	Advances in medical sciences	Semiautomatic preprocessing (brain extraction)	y	T1, T2, FLAIR, SD	Bayesian classification, Other	REG: rigid; BE:ref; tissue maps; IN:histogram matching; smoothing:Gaussian kernel	Silicon Graphics (Mountain View, CA) Origin 300 workstation	4 min	n	n	WML	-	42	3 (ACCORD-MIND)	y	Manual, 1		TPR; FPR; DSC; ROC
J	2008	J. J. Corso et al.	Efficient Multilevel Brain Tumor Segmentation With Integrated Bayesian Model Classification	IEEE Transactions on Medical Imaging		y	T1, T1c, T2, FLAIR	Other	REG:FSL; denoising:FSL; BE:FSL; IN:FSL	1.67 Gz 1.5 GB	<2 min	n	n	BT	GBM	20	3	n/a	Expert segmentation		Jcc
J	2005	F. Admiraal-Behloul et al.	Fully automatic segmentation of white matter hyperintensities in MR images of the elderly	NeuroImage	Inter-rater agreement evaluated, Scan-rescan reproducibility	y	PD; T2, FLAIR	Fuzzy C-Means	REG:rigid(AIR); tissue maps: affine transformation from MNI; BE:FCM	Pentium 4 512MB RAM	2 min	n	n	WML	-	100; 44 (different slice thickness)	3 (PROSPER, Shepherd et al. 1999)	n	Manual, 2		ICC; Bland-Altman; DSC
J	2005	O. Colliot et al.	Segmentation of focal cortical dysplasia lesions on MRI using level set evolution	NeuroImage	Inter-rater agreement evaluated, Segmentation failure	n	T1	Level Set	IN; BFC; REG; BE: BET; tissue maps:thresholding	1.6GHz	14 min	n	n	FCD	FCD	24	3	n	Manual, 2	consensus	SI; FP; other
J	2004	W. Li et al.	Robust unsupervised segmentation of infarct lesion from diffusion tensor MR images using multiscale statistical classification and partial volume voxel reclassification	Computational Diffusion MRI	Inter-rater agreement evaluated	n	DTI	Other	BFC & denoising:ADF	n/a	n/a	n	n	S	-	20	3	y	Manual		DSC
J	2004	P. Anbeek et al.	Automatic segmentation of different-sized white matter lesions by voxel probability estimation	Medical Image Analysis		y	T1, IR, T2, PD, FLAIR	K-Nearest Neighbours, Thresholding	IN:histogram matching; REG:rigid; BE:Mbrase	n/a	n/a	n	n	WML	Arterial VaD	19	3	n	Manual, 2	supervision	TPR; FPR; DSC; overlap fraction; error fraction; other
J	2002	A. P. Zijdenbos et al.	Automatic 'pipeline' analysis of 3-D MRI data for clinical trials: application to multiple sclerosis	IEEE Transactions on Medical Imaging		y	T1, T2, PD	Artificial neural networks	IN:adaptive classification; denoising:ADF; BFC:N3; REG; resampling	n/a	n/a	n	n	MS	-	10; 29	3 (Weiner et al. 1997)	y	Manual, 7; Semiautomatic		other
J	2001	K. Van Leemput et al.	Automated segmentation of multiple sclerosis lesions by model outlier detection	IEEE Transactions on Medical Imaging	Inter-rater agreement evaluated	y	PD, T2	Other	REG:affine; BFC:EM	n/a	n/a	n	n	MS	-	10x2 + 3 (HiRes)	3 (BIOMORPH, 1996–1998)	n	Manual, 1&2		DSC; VC
J	1998	L. P. Clarke et al.	MRI Measurement of Brain Tumor Response: Comparison of Visual Metric and Automatic Segmentation	Magnetic Resonance Imaging		y	T1, T2, PD	Fuzzy C-Means	n/a	n/a	n/a	n	n	BT	-	6x(2-5)	3	n	Manual, multiple		VC
J	1997	S. Dickson et al.	Using Neural Networks to Automatically Detect Brain Tumours in MR Images	International Journal of Neural Systems		n	T1c	Artificial neural networks	n/a	n/a	n/a	n	n	BT	Acoustic neuroma	50	3	n	Manual		other
J	1995	M. Kamber et al.	Model-based 3-D segmentation of multiple sclerosis lesions in magnetic resonance brain images	IEEE Transactions on Medical Imaging	Inter-rater agreement evaluated, Segmentation failure, Manual corrections of preprocessing	y	PD, T2	Other	BE; BFC:homomorphic filtering	n/a	n/a	n	n	MS	MS	12; 44	3	n	Manual, 1		other
C	2019	S. N. Shivhare et al.	An Efficient Brain Tumor Detection and Segmentation in MRI Using Parameter-Free Clustering	Machine Intelligence and Signal Analysis		n	T1c	K-Means, Other	n/a	n/a	n/a	n	n	BT	HGG	273	1 (BraTS'15)	y	-		DSC
C	2018	S. M. Anwar et al.	Brain tumor segmentation on Multimodal MRI scans using EMAP Algorithm	IEEE EMBC		y	T2; FLAIR	K-Means, Expectation-Maximization	BFC:N4r; IN:0-1 range	i7 16BG RAM	comparable to previous techniques	n	n	BT	LGG; HGG	40	1 (BraTS'15)	y	-		DSC; Jcc; PPV; Sen; Kappa
C	2018	D. Jin et al.	White matter hyperintensity segmentation from T1 and FLAIR images using fully convolutional neural networks enhanced with residual connections	ISBI		y	T1, FLAIR	Artificial neural networks	IN:0-1 range; tissue map	n/a	n/a	n	n	WML	-	170	1 (WMH Segmentation Challenge '17)	y	-		DSC; Hdff; avgVD; Sen; F1
C	2018	A. F. I. Osman	Automated Brain Tumor Segmentation on Magnetic Resonance Images and Patient's Overall Survival Prediction Using Support Vector Machines	Brainlesion: Glioma, Multiple Sclerosis, Stroke and Traumatic Brain Injuries		y	T1, T1c, T2, FLAIR	Support Vector Machine	IN:max global intensity + thresholding; denoising:median filtering	n/a	n/a	Overall survival:short, medium, long	n	BT	LGG; HGG	477	1 (BraTS'17)	y	-		DSC
C	2018	Y. Jiang et al.	A Brain Tumor Segmentation New Method Based on Statistical Thresholding and Multiscale CNN	Intelligent Computing Methodologies		y	T1, T1c, T2, FLAIR	Artificial neural networks, Thresholding	denoising:median filtering	n/a	n/a	n	n	BT	LGG; HGG	274	1 (BraTS'15)	y	-		DSC; PPV; Sen
C	2018	L. Chen et al.	MRI tumor segmentation with densely connected 3D CNN	Medical Imaging 2018: Image Processing		y	T1, T1c, T2, FLAIR	Artificial neural networks	BFC:N4; IN:0u1v	n/a	n/a	n	n	BT	-	210	1 (BraTS'17)	y	-		DSC
C	2018	Y. Xu et al.	White Matter Hyperintensities Segmentation in a Few Seconds Using Fully Convolutional Network and Transfer Learning	Brainlesion: Glioma, Multiple Sclerosis, Stroke and Traumatic Brain Injuries		y	T1, FLAIR	Artificial neural networks	BFC; morphological operations	NVIDIA GeForce GTX 1080	10 sec	n	n	WML	-	60 + 110 (testing)	1 (WMH Segmentation Challenge '17)	y	-		DSC; Hdff; avgVD; Sen; F1

For peer review only

C	2018	M. Soltaninejad et al.	MRI Brain Tumor Segmentation and Patient Survival Prediction Using Random Forests and Fully Convolutional Networks	Brainlesion: Glioma, Multiple Sclerosis, Stroke and Traumatic Brain Injuries		y	T1, T2, FLAIR	Artificial neural networks, Random Forest	IN:1-99% clipping + 0u1v + histogram matching	i7 16BG RAM GTX980i	n/a	overall survival; long; medium; short	n	BT	-	497	1 (BraTS'17)	y	-		DSC; Sen; Spe; Hdff
C	2017	M. Bento et al.	Probabilistic Segmentation of Brain White Matter Lesions Using Texture-Based Classification	Image Analysis and Recognition		y	T1, T2, DWI, FLAIR	Thresholding, Support Vector Machine, K-Nearest Neighbours, Random Forest	resizing; tissue maps:semi-automatic region growing; IN:0-255 range	n/a	n/a	n	n	S	S	28	1 (ISLES'15)	y	-		DSC; Sen; Spe; Acc
C	2018	M. Bento et al.	WMH Segmentation Challenge: A Texture-Based Classification Approach	Brainlesion: Glioma, Multiple Sclerosis, Stroke and Traumatic Brain Injuries		y	T1, FLAIR	Random Forest	cropping; resizing; tissue map; IN:0-255 range	Intel Zeon 12-Core 2.7 GHz 64GB DDR3 RAM	90 sec	n	n	WML	-	60 + 110 (testing)	1 (MS GC MICCAI'08)	y	-		DSC; Hdff; avgVD; Sen; F1
C	2018	M. Shaikh et al.	Brain Tumor Segmentation Using Dense Fully Convolutional Neural Network	Brainlesion: Glioma, Multiple Sclerosis, Stroke and Traumatic Brain Injuries		y	T1, T1c, T2, FLAIR	Artificial neural networks	IN:z-score	Titan X GPU	<30 sec	n	n	BT	LGG; HGG	284; 29	1 (BraTS'17)	y	-		DSC
C	2018	Y. Hu and Y. Xia	3D Deep Neural Network-Based Brain Tumor Segmentation Using Multimodality Magnetic Resonance Sequences	Brainlesion: Glioma, Multiple Sclerosis, Stroke and Traumatic Brain Injuries		y	T1, FLAIR	Artificial neural networks	IN:0-255 range	Intel Xeon 2.10 GHz CPU, NVIDIA GTX 1080 Ti GPU	3 min	n	n	BT	LGG; HGG	477	1 (BraTS'17)	y	-		DSC; Hdff
C	2018	Z. Kapás et al.	Automatic Brain Tumor Segmentation in Multispectral MRI Volumes Using a Random Forest Approach	Image and Video Technology		y	T1, T1c, T2, FLAIR	Random Forest	IN:linear transformation	I7 3.4GHz	45 sec	n	n	BT	HGG	220	1 (BraTS'16)	y	-		DSC; Sen; Spe
C	2018	L. Vidyaratne et al.	Deep learning and texture-based semantic label fusion for brain tumor segmentation	Proceedings of SPIE--the International Society for Optical Engineering		y	T1, T1c, T2, FLAIR	Artificial neural networks, Random Forest	BFC:N4; IN:ref	n/a	n/a	n	n	BT	HGG	243	1 (BraTS'17)	y	-		DSC
C	2018	S. Doyle et al.	Sub-acute and Chronic Ischemic Stroke Lesion MRI Segmentation	Brainlesion: Glioma, Multiple Sclerosis, Stroke and Traumatic Brain Injuries		y	T1, FLAIR	Other	IN: to a reference image; BFC:N4; REG:ITK; tissue maps:ITK	n/a	n/a	n	n	S	-	37; 47	1 (Maier et al. 2015); 3 (HERMES)	y	Manual, 1		DSC; Hdff; avgSD; PPV; Sen; VD
C	2018	S. Amiri et al.	Bayesian Network and Structured Random Forest Cooperative Deep Learning for Automatic Multi-label Brain Tumor Segmentation	International Conference on Agents and Artificial Intelligence		y	T1, T1c, FLAIR	Artificial neural networks, Random Forest	BFC:N4; IN:histogram linear transformation	n/a	n/a	n	n	BT	HGG	220	1 (BraTS'15)	y	-		DSC
C	2018	G. C. Oliveira et al.	Brain Tumor Segmentation in Magnetic Resonance Images using Genetic Algorithm Clustering and AdaBoost Classifier	BIOIMAGING			n/a	boosting, Genetic Algorithm	n/a	I7 2.4GHz 8GB RAM	6.22 min	n	n	BT	LGG	42	1 (BraTS'15)	y	-		DSC
C	2018	G. Wang et al.	Automatic Brain Tumor Segmentation Using Cascaded Anisotropic Convolutional Neural Networks	Brainlesion: Glioma, Multiple Sclerosis, Stroke and Traumatic Brain Injuries		y	T1, T1c, T2, FLAIR	Artificial neural networks	n/a	NVIDIA TITAN X GPU	n/a	n	n	BT	LGG; HGG	285; 46; 146	1 (BraTS'17)	y	-		DSC; Hdff
C	2018	D. Bhattacharya and N. Sinha	An improved approach of high graded glioma segmentation using sparse autoencoder and fuzzy c-means clustering from multi-modal MR images	SPIE Medical Imaging		y	T1, T1c, T2, FLAIR	Artificial neural networks	none	n/a	n/a	n	n	BT	HGG	15	1 (BraTS'15)	y	-		DSC; Jcc
C	2018	H. N. Bharath et al.	Tumor Segmentation from Multimodal MRI Using Random Forest with Superpixel and Tensor Based Feature Extraction	Brainlesion: Glioma, Multiple Sclerosis, Stroke and Traumatic Brain Injuries		y	T1, T1c, T2, FLAIR	Random Forest	IN:0-1 range + histogram equalization; backgound removal:Otsu's thresholding	n/a	n/a	n	n	BT	HGG	285; 46; 146	1 (BraTS'17)	y	-		DSC; Sen; Hdff
C	2018	R. Pourreza et al.	Brain Tumor Segmentation in MRI Scans Using Deeply-Supervised Neural Networks	Brainlesion: Glioma, Multiple Sclerosis, Stroke and Traumatic Brain Injuries		y	T1c, T2, FLAIR	Artificial neural networks	BFC:N4; IN:histogram mathcing	NVIDIA Titan Xp	30 sec	n	n	BT	LGG; HGG	285	1 (BraTS'17)	y	-		DSC
C	2018	M. F. Rachmadi et al.	Automatic Irregular Texture Detection in Brain MRI Without Human Supervision	Medical Image Computing and Computer Assisted Intervention – MICCAI 2018		n	FLAIR	Other	BE:optiBET, tissue map:in-house	GPU	13 min	n	n	WML	-	20	2 (ADNI)	y	n/a		DSC
C	2017	C. Ö. Fiçici et al.	Fully Automated Brain Tumor Segmentation and Volume Estimation Based on Symmetry Analysis in MR Images	CMBEBIH		y	T1, T1c, FLAIR	Fuzzy C-Means, Other	denoising:median filtering; cropping; BE:thresholding	n/a	n/a	n	n	BT	-	10	3	n	Expert segmentation		DSC; Jcc; Sen; Spe;
C	2017	S. R. Mote et al.	Non-negative matrix factorization and self-organizing map for brain tumor segmentation	2017 International Conference on Wireless Communications, Signal Processing and Networking (WiSPNET)		y	T2, FLAIR	Self Organising Maps	BFC & denoising:ADF	n/a	n/a	n	n	BT	LGG; HGG	30 (+25 synthetic)	1 (BraTS'12)	y	-		DSC; Sen; Spe
C	2017	A. López-Zorrilla et al.	Brain White Matter Lesion Segmentation with 2D/3D CNN	Natural and Artificial Computation for Biomedicine and Neuroscience		y	T1, FLAIR, DTI	Artificial neural networks	REG:affine; IN:0-1 range	GTX 1070 16GB RAM	n/a	n	n	WML	AD, VaD	18	3 (Price et al. 2012)	n	Manual		TPR
C	2017	H. Shen et al.	Multi-task Fully Convolutional Network for Brain Tumour Segmentation	Medical Image Understanding and Analysis		y	T1, T1c, T2, FLAIR	Artificial neural networks	n/a	n/a	n/a	n	n	BT	HGG	30; 220	1 (BraTS'13); 1 (BraTS'15)	y	-		DSC; Sen; PPV; F-score

For peer review only

C	2017	S. Pereira et al.	On hierarchical brain tumor segmentation in MRI using fully convolutional neural networks: A preliminary study,	ENBENG		y	T1, T1c, T2, FLAIR	Artificial neural networks	IN:histogram normalization + 0u1v	I7 3.2GHz 48GB RAM GTX970 GPU	40 sec	n	n	BT	-	40	1 (BraTS'13)	y	-		DSC; PPV; Sen
C	2017	H. Dong et al.	Automatic Brain Tumor Detection and Segmentation Using U-Net Based Fully Convolutional Networks	Medical Image Understanding and Analysis		y	T1c, FLAIR	Artificial neural networks	IN:0u1v	Titan X GPU 12Gb RAM	3 sec	n	n	BT	LGG; HGG	274	1 (BraTS'15)	y	-		DSC; Sen
C	2017	M. Rezaei et al.	Deep Neural Network with l2-Norm Unit for Brain Lesions Detection	Neural Information Processing		y	T1,T2, DWI, FLAIR	Artificial neural networks	n/a	n/a	n/a	LGG; HGG, AD; S	n	BT, S	LGG; HGG; S	328; 30	1 (BraTS'16); 1 (ISLES'16)	y	-		DSC
C	2017	S. Hussain et al.	Brain tumor segmentation using cascaded deep convolutional neural network	Annual International Conference of the IEEE		y	T1, T1c, T2, FLAIR	Artificial neural networks	BFC:N4; IN:0u1v	n/a	n/a	n	n	BT	LGG; HGG	30 +25 synthetic	1 (BraTS'13)	y	-		DSC; Sen; Spe
C	2017	W. Chen et al.	Automatic brain tumor segmentation based on features of separated local square	Chinese Automation Congress		n	FLAIR	Support Vector Machine	BFC:N4; IN:histogram matching	n/a	n/a	n	n	BT	LGG; HGG	20 (randomly selected from 30)	1 (BraTS'13)	y	-		DSC; Sen; Spe
C	2017	G. Latif et al.	Automatic Multimodal Brain Image Classification Using MLP and 3D Glioma Tumor Reconstruction	IEEE-GCC Conference and Exhibition		y	T1, T1c, T2, FLAIR	Other, Active Contour	denoising: ADF; BE:thresholding + morphologica operations	n/a	n/a	normal, abnormal	n	BT	LGG; HGG	160	1 (BraTS'15)	y	-		DSC; Spe; Sen; Acc; other
C	2017	W. Mengqiao et al.	The multimodal brain tumor image segmentation based on convolutional neural networks	IEEE International Conference on Computational Intelligence and Applications		y	T1, T1c, T2, FLAIR	Artificial neural networks	BFC:N4; IN:0u1v	I7 2.8GHz GPU GeForce 1050	n/a	n	n	BT	LGG; HGG	120	1 (BraTS'15)	y	-		DSC; PPV; Sen
C	2017	V. Shreyas et al.	A deep learning architecture for brain tumor segmentation in MRI images	International Workshop on Multimedia Signal Processing		y	T1, T1c, T2, FLAIR	Artificial neural networks	BFC:N4; IN:histogram matching + z-score	i7 128GB RAM	13 sec	n	n	BT	HGG	220	1 (BraTS'15)	y	-		DSC; PPV; Sen
C	2017	N. Shah et al.	Brain tumor segmentation and classification using cascaded random decision forests	International Conference on Electrical Engineering/Electronics, Computer, Telecommunications and Information Technology		y	T1, T1c, T2, FLAIR	Random Forest	IN:histogram matching	n/a	n/a	n	n	BT	LGG; HGG	30 +50 simulated	1 (BraTS'13)	y	-		DSC; Jcc
C	2017	U. Baid et al.	Novel approach for brain tumor segmentation with non negative matrix factorization	International Conference on Innovations in Electronics, Signal Processing and Communication		y	T2, FLAIR	Fuzzy C-Means, Matrix Factorization	denoising:ADF; BFC:N3	n/a	n/a	n	n	BT	LGG; HGG	30 +50 simulated	1 (BraTS'12)	y	-		DSC; Sen; Spe; Hdff
C	2017	M. K. Akter et al.	Automated brain tumor segmentation from mri data based on exploration of histogram characteristics of the cancerous hemisphere	IEEE Region 10 Humanitarian Technology Conference		y	T1, T1c, T2, FLAIR	Thresholding, Other	n/a	n/a	n/a	n	n	BT	HGG	15	1 (BraTS'12)	y	-		DSC; Sen; Spe
C	2017	S. Goel et al.	Brain tumor segmentation in multispectral MR images	International Conference on Signal Processing and Integrated Networks		y	T1, T1c, T2, FLAIR	Fuzzy C-Means	BFC:N3; IN:thresholding + contrast enhancement, image fusion; smoothing	n/a	n/a	n	n	BT	HGG	15	1 (BraTS'13)	y	-		DSC; PPV; Sen
C	2017	A. Sankari and S. Vigneshwari	Automatic tumor segmentation using convolutional neural networks	International Conference on Science Technology Engineering Management		n/a	n/a	Artificial neural networks	denoising; IN; BFC; contrast enhancement:histogram equalization	n/a	n/a	n	n	BT	LGG; HGG	391	1 (BraTS'15); 1 (IBSR)	y	-		Sen; Spe; acc
C	2016	R. Liu et al.	Multi-modal Brain Tumor Segmentation Based on Self-organizing Active Contour Model	Chinese Conference on Pattern Recognition		y	T1, T1c, T2, FLAIR	Self Organising Maps, Active Contour	IN:histogram normalization; contrast adjustment:ITKSnap	n/a	n/a	n	n	BT	LGG; HGG	45	1 (BraTS'13); 1 (BraTS'15)	y	-		DSC; Sen; Spe
C	2016	Y. Wang et al.	A deep symmetry convnet for stroke lesion segmentation	IEEE ICIP		n	T1	Artificial neural networks	BFC:AFNI; BE:SymROBEX; IN:0u1v; contrast enhancement:histogram equalization	n/a	n/a	n	n	S	Aphasia due to S	18	3	n	Manual		DSC
C	2016	B. Song et al.	Anatomy-Guided Brain Tumor Segmentation and Classification	Brainlesion: Glioma, Multiple Sclerosis, Stroke and Traumatic Brain Injuries		y	T1, T1c, T2, FLAIR	Random Forest, Grow Cut	BFC:N4; IN:linear normalization of tissue histograms	2.67GHz 24 GB RAM	1 min	n	n	BT	LGG; HGG	274	1 (BratTS'15); 1 (BraTS'16)	y	-		DSC; Hdff
C	2016	E. Ilunga-Mbuyamba et al.	Automatic brain tumor tissue detection based on hierarchical centroid shape descriptor in Tl-weighted MR images	2016 International Conference on Electronics, Communications and Computers (CONIELECOMP)		n	T1	K-Means, Other	BE: thresholding + morphological operations	1.5GHz 2GB RAM	n/a	n	n	BT	-	254	3 (University Hospital, Department of Neurosurgery, University of Leipzig, Germany)	n	Expert segmentation		DSC; Jcc
C	2016	M. Agn et al.	Brain Tumor Segmentation Using a Generative Model with an RBM Prior on Tumor Shape	Brainlesion: Glioma, Multiple Sclerosis, Stroke and Traumatic Brain Injuries		y	T1, T1c, T2, FLAIR	Artificial neural networks	BFC:log transform	i7 CPU, GTX Titan Black GPU	30 min	n	n	BT	LGG; HGG	65; 297	1 (BraTS'13); 1 (BraTS'15)	y	-		DSC
C	2015	M. Havaei et al.	A Convolutional Neural Network Approach to Brain Tumor Segmentation	Brainlesion: Glioma, Multiple Sclerosis, Stroke and Traumatic Brain Injuries		y	T1, T1c, T2, FLAIR; T1, DWI, FLAIR	Artificial neural networks	n/a	Nvidia Titan black GPU	25 sec – 3 min	n	n	BT, S	LGG; HGG; S	40; 327; 114	1 (BraTS'13); 1 (BraTS'15); 1 (ISLES'15)	y	-		DSC; Sen; Spe; avgSD; Hdff
C	2016	X. Chen et al.	Automated brain tumor segmentation using kernel dictionary learning and superpixel-level features	IEEE SMC		y	T1, T1c, T2, FLAIR	Sparse representation, Graph Cut	BFC:N4; IN:histogram matching	I7 3.2GHz 32GB RAM	30 sec	n	n	BT	HGG	20; 10	1 (BraTS'13)	y	-		DSC; Jcc; Sen
C	2016	Q. Mahmood and A. Basit	Prediction of Ischemic Stroke Lesion and Clinical Outcome in Multi-modal MRI Images Using Random Forests	Brainlesion: Glioma, Multiple Sclerosis, Stroke and Traumatic Brain Injuries		y	DWI, PWI	Random Forest	n/a	15 2.5GHz	5 min	clinical outcome – medical ranking score	n	S	S	49	1 (ISLES'16)	y	-		avgSSD; Hdff; DSC
C	2015	Q. Mahmood and A. Basit	Automatic ischemic stroke lesion segmentation in multi-spectral mri images using random forests classifier	MICCAI BrainLes		y	T1, T2, DWI, FLAIR	Random Forest	BFC:N3; IN:0-1 range	I5 2.5 GHz 4GB RAM	~30 min	n	n	S	S	64	1 (ISLES'15)	y	-		avgSSD; Hdff; DSC; PPV; Sen
C	2016	K. Vaidhya et al.	Multi-modal Brain Tumor Segmentation Using Stacked Denoising Autoencoders	Brainlesion: Glioma, Multiple Sclerosis, Stroke and Traumatic Brain Injuries		n/a	n/a	Artificial neural networks	IN:histogram matching + mean division and outlier removal	n/a	30 min	n	n	BT	LGG; HGG	274	1 (BraTS'15)	y	-		DSC
C	2016	S. Amiri et al.	Deep random forest-based learning transfer to SVM for brain tumor segmentation	International Conference on Advanced Technologies for Signal and Image Processing		n	FLAIR	Random Forest, Support Vector Machine	IN; contrast enhancement	n/a	n/a	n	n	BT	HGG	20	1 (BraTS'13)	y	-		DSC
C	2016	S. Müller et al.	Automatic brain tumor segmentation with a fast Mumford-Shah algorithm	SPIE Medical Imaging		y	T1c, T2, FLAIR	Thresholding, Other	BFC:N4; IN histogram equalization + 0-225 range	I7 3.4GHz 16BG RAM GTX 970 4GB RAM	<1 min	n	n	BT	LGG; HGG	213	1 (BraTS'14)	y	-		DSC; Sen; PPV
C	2016	E. A. R. Piedra et al.	Brain Tumor Segmentation by Variability Characterization of Tumor Boundaries	Brainlesion: Glioma, Multiple Sclerosis, Stroke and Traumatic Brain Injuries		y	T1, T1c, T2, FLAIR	Other	tissue maps	n/a	n/a	n	n	BT	-	220	1 (BraTS'15)	y	-		DSC; Hdff
C	2016	Z. Xiao et al.	A deep learning-based segmentation method for brain tumor in MR images	International Conference on Computational Advances in Bio and Medical Sciences		n/a	n/a	Artificial neural networks	cropping; IN	n/a	n/a	n	n	BT	-	10	3 (West China Hospital)	n	Manual		percentage match
C	2016	A. Sehgal et al.	Automatic brain tumor segmentation and extraction in MR images	Conference on Advances in Signal Processing		n	T1	Fuzzy C-Means, Other	BFC:N3; denoising:ADF; enhancement:unsharp mask	n/a	n/a	n	n	BT	-	30	1 (BraTS'13)	y	-		DSC
C	2016	A. Derntl et al.	Stroke Lesion Segmentation Using a Probabilistic Atlas of Cerebral Vascular Territories	Brainlesion: Glioma, Multiple Sclerosis, Stroke and Traumatic Brain Injuries		y	not specific to a modality	Expectation-Maximization Gausian mixture model, Conditional Random Field, Other,	REG; BFC; BE	n/a	n/a	n	n	S	S	13	3	n/a	Manual		DSC

For peer review only

C	2015	Y. Li et al.	Automatic Brain Tumor Segmentation from MR Images via a Multimodal Sparse Coding Based Probabilistic Model	International Workshop on Pattern Recognition in NeuroImaging		y	T1, T1c, T2, FLAIR	Sparse representation, Markov Random Field	REG:rigid; BE; IN:0-1 range	n/a	n/a	n	n	BT	-	30	1 (BraTS'12)	y	-		DSC; Sen; Spe
C	2015	M. Lyksborg et al.	An Ensemble of 2D Convolutional Neural Networks for Tumor Segmentation	Scandinavian Conference on Image Analysis		y	T1, T1c, T2, FLAIR	Artificial neural networks	BFC:N4; IN:histogram scaling	n/a	n/a	n	n	BT	-	131; 187 (longitudinal)	1 (BraTS'14)	y	-		DSC; PPV; Sen
C	2015	R. Mechrez et al.	MS lesion segmentation using a multi-channel patch-based approach with spatial consistency	Medical Imaging 2015: Image Processing		y	T1, T2, FLAIR	Non-local Means, K-Nearest Neighbours, Other,	BE; BFC; sub-sampling; IN	n/a	n/a	n	n	MS	-	10	1 (MS GC MICCAI'08)	n	-		DSC; TPR; PPV
C	2015	I. Zabir et al.	Automatic brain tumor detection and segmentation from multi-modal MRI images based on region growing and level set evolution	2015 IEEE International WIE Conference on Electrical and Computer Engineering (WIECON-ECE)		y	T2, FLAIR	K-Nearest Neighbours, Thresholding, region growing, level set	n/a	n/a	n/a	n	n	BT	-	17	1 (BraTS'12)	y	-		DSC; Jcc; Sen; Spe
C	2015	A. Jog et al.	Multi-output decision trees for lesion segmentation in multiple sclerosis	Proceedings of SPIE--the International Society for Optical Engineering		y	T1, T2, FLAIR	Decision Tree	BFC:N4; BE:ref; REG:ref; IN	n/a	2 min	n	n	MS	-	20; 49	1 (MS GC MICCAI'08); 3	y	Manual		PPV; TPR
C	2015	H. Tang et al.	Tumor segmentation from single contrast MR images of human brain	2015 IEEE 12th International Symposium on Biomedical Imaging (ISBI)	Segmentation failure	n	T2	Random Forest	REG:rigid+affine; IN:linear scaling to MNI	n/a	n/a	n	n	BT	LGG; HGG	30	1 (BraTS'12)	y	-		DSC
C	2015	L. Szilágyi et al.	Automatic Brain Tumor Segmentation in Multispectral MRI Volumetric Records	Neural Information Processing		y	T1, T1c, T2, FLAIR	Fuzzy C-Means	n/a	n/a	n/a	n	n	BT	-	13	1 (BraTS'12)	y	-		Jcc; DSC
C	2015	Y. Karpate et al.	Probabilistic one class learning for automatic detection of multiple sclerosis lesions	2015 IEEE 12th International Symposium on Biomedical Imaging (ISBI)		y	T1, T2, FLAIR	Thresholding, probabilistic classification	n/a	n/a	n/a	n	n	MS	-	16 + 20 controls	3	n	Manual, 1		PPV; Sen
C	2015	H. Deshpande et al.	Adaptive dictionary learning for competitive classification of multiple sclerosis lesions	2015 IEEE 12th International Symposium on Biomedical Imaging (ISBI)		y	T1, T2, PD, FLAIR	Sparse representation, Dictionary Learning	BFC; denoising:non-local means; REG:BE; ref	n/a	n/a	n	n	MS	MS	13	3	n	Manual, 1		Sen; PPV
C	2015	I. Gondra and I. Cabria	Automated segmentation of brain tumors in MRI using potential field clustering	International Conference on Computer as a Tool		n	FLAIR	Region Growing, Other	n/a	n/a	n/a	n	n	BT	-	22	1 (BraTS'12)	y	-		other
C	2015	A. Pinto et al.	Brain Tumour Segmentation based on Extremely Randomized Forest with high-level features	Annual International Conference of the IEEE Engineering in Medicine and Biology Society (EMBC)		y	T1, T1c, T2, FLAIR	Decision Tree	BFC:ref; IN: histogram matching	n/a	n/a	n	n	BT	LGG; HGG	40	1 (BraTS'13)	y	-		DSC; PPV; Sen
C	2015	K. B. Vaishnavee and K. Amshakala	An automated MRI brain image segmentation and tumor detection using SOM-clustering and Proximal Support Vector Machine classifier	IEEE International Conference on Engineering and Technology		n	T1	Self Organising Maps, Support Vector Machine	BE:BET; enhancement:histogram equalization	n/a	n/a	n	n	BT	-	20	1 (IBSR); 3 (Medicine Service of the "Virgen de las Nieves" Hospital, Granada, Spain)	y	Manual		TI
C	2015	A. Pinto et al.	Random decision forests for automatic brain tumor segmentation on multi-modal MRI images	IEEE Portuguese Meeting on Bioengineering		y	T1, T1c, T2, FLAIR	Random Forest	BFC:N4; IN:histogram matching	I7 3.2GHz 47GB RAM	n/a	n	n	BT	LGG; HGG	30	1 (BraTS'13)	y	-		DSC; PPV; Sen
C	2015	Y. Renping et al.	Automatic Segmentation of White Matter Lesions Using SVM and RSF Model in Multi-channel MRI	Image and Graphics		y	T1, T2, PD, FLAIR	Support Vector Machine, Other	REG:rigid (MI); BE:ref; BFC:N3; IN:in paper	n/a	n/a	n	n	WML	-	45	3 (ACCORD-MIND)	n/a	Manual, 1		DSC; TPR; TNR; PPV
C	2015	N. Nabizadeh et al.	Automatic tumor lesion detection and segmentation using modified winnow algorithm	ISBI	Detection followed by segmentation	n	FLAIR	Active Contour, Level Set	IN:histogram normalization	n/a	n/a	n	n	BT	HGG	25 +25 simulated	1 (BraTS'13)	y	-		Acc
C	2015	S. E. El-Khamy et al.	An efficient brain mass detection with adaptive clustered based fuzzy C-mean and thresholding	International Conference on Signal and Image Processing Applications		n/a	n/a	Fuzzy C-Means	enhancement:intensity scaling	I5 2.3GHz 4GB RAM	~5 sec	n	n	BT	T	15	3	n/a	Expert segmentation		Acc, other
C	2015	A. F. Muda et al.	Integration of Fuzzy C-Means with Correlation Template and Active Contour for Brain Lesion Segmentation in Diffusion-Weighted MRI	International Conference on Artificial Intelligence, Modelling and Simulation		n	DWI	Fuzzy C-Means, Active Contour, Other,	Ref	n/a	n/a	n	n	BT, S	-	40	3	n/a	Manual		Jcc; DSC; FPR; FNR
C	2015	A. F. Muda et al.	Brain Lesion Segmentation Using Fuzzy C-Means On Diffusion-Weighted Imaging	Journal of Engineering and Applied Sciences		n	DWI	Fuzzy C-Means, Edge detection, Watershed	Ref	n/a	n/a	n	n	BT, S	T, S	20	3 (General Hospital of Kuala Lumpur)	y	Manual		FPR; FNR; DSC; other
C	2014	S. D. S. Al-Shaikhli et al.	Coupled Dictionary Learning for Automatic Multi-Label Brain Tumor Segmentation in Flair MRI images	Advances in Visual Computing		n	FLAIR; T1	Graph Cut, Dictionary Learning	n/a	2GHz CPU	n/a	n	n	BT	-	10; 30	1 (BraTS'13); 1 (SPL)	y	-		DSC; PPV; Sen; Jcc; Kappa
C	2014	H. J. Kuijf et al.	The Added Value of Diffusion Tensor Imaging for Automated White Matter Hyperintensity Segmentation	Computational Diffusion MRI		y	T1, FLAIR, DTI	K-Nearest Neighbours	IN:histogram matching; REG:rigid (elastix)	n/a	n/a	n	n	WML	-	20	3	n	Manual, 2	supervision	DSC; Sen
C	2014	P. K. Roy et al.	Automated Segmentation of White Matter Lesions Using Global Neighbourhood Given Contrast Feature-Based Random Forest and Markov Random Field	2014 IEEE International Conference on Healthcare Informatics		y	T1, FLAIR	Random Forest, Markov Random Field	REG:rigid(SPM8); BE:BET; BFC:N3	n/a	n/a	n	n	WML	HT	24	3 (ENVISion, Reid et al. 2012)	n	Manual, 2	supervision	DSC
C	2014	A. Rao et al.	Contusion segmentation from subjects with Traumatic Brain Injury: A random forest framework	2014 IEEE 11th International Symposium on Biomedical Imaging (ISBI)		y	T1, DTI, FLAIR	Random Forest	BFC:N4; IN:linear scaling; REG:affine; tissue maps	n/a	n/a	n	n	TBI	-	23	3	n	Manual, 1		DSC; TPR; PPV
C	2014	N. Subbanna et al.	Iterative Multilevel MRF Leveraging Context and Voxel Information for Brain Tumour Segmentation in MRI	IEEE Conference on Computer Vision and Pattern Recognition		y	T1, T1c, T2, FLAIR	Bayesian classification, Markov Random Field	BFC; IN	Dell Optiplex 980 I7	75 min	n	n	BT	LGG; HGG	30;25 (challenge sets)	1 (BraTS'12); 1 (BraTS'13)	y	-		DSC
C	2015	S. Reza et al.	Ischemic stroke lesion segmentation using local gradient and texture features	MICCAI ISLES		y	T1, T2, DWI, FLAIR	Random Forest	IN:histogram matching + CSF normalizaiton	n/a	n/a	n	n	S	S	28	1 (ISLES'15)	y	-		DSC
C	2014	S. Reza et al.	Improved brain tumor tissue segmentation using texture features	MICCAI 2014		y	T1, T1c, T2, FLAIR	Random Forest	BFC; IN	n/a	n/a	n	n	BT	LGG; HGG	246	1 (BraTS'13); 1 (BraTS'14)	y	-		DSC; Jcc; PPV; Sen; Kappa
C	2014	H. Hooda et al.	Brain tumor segmentation: A performance analysis using K-Means, Fuzzy C-Means and Region growing algorithm	IEEE International Conference on Advanced Communications, Control and Computing Technologies		n/a	n/a	Fuzzy C-Means	BC: median filtering; BE: Otsu's thresholding + morphological operations	2GHz 2GB RAM	n/a	n	n	BT	-	10	3	n	Manual		TP; other
C	2014	P. Dvorak et al.	Automatic Extraction of Pathological Area in 2D MR Brain Scan	Progress in Electromagnetics Research Symposium		y	T2, FLAIR	Other	symmetry axis detection	n/a	n/a	n	n	BT	-	22	1 (BraTS'12)	y	-		DSC; Acc
C	2014	S. Bauer et al.	Towards automatic MRI volumetry for treatment selection in acute ischemic stroke patients	Annual International Conference of the IEEE Engineering in Medicine and Biology Society		y	T1c, T2, DWI, DSC	Decision Tree, Conditional Random Field	REG:rigid; BE:ref;	n/a	<1 min	n	n	S	S	10	3	n	Manual, 1		DSC; VE
C	2014	A. V. Dalca et al.	Segmentation of Cerebrovascular Pathologies in Stroke Patients with Spatial and Shape Priors	International Conference on Medical Image Computing and Computer-Assisted Intervention		y	FLAIR, T2	Markov Random Field	n/s	n/a	n/a	n	n	WML, S	-	148	3	n/a	Manual		VC
C	2014	F. Rodrigo et al.	Segmentation of Hyperintense Regions Applied to Multiple Sclerosis Lesions	Latin American Congress on Biomedical Engineering	Segmentation failure	n	FLAIR	Thresholding	IN: histogram matching; BE:SPM; REG; enhancement: LP filter + saturation	n/a	n/a	n	n	MS	MS	10	3	n/a	Manual, 1		other

For peer review only

C	2013	Z. Karimaghaloo et al.	Adaptive Voxel, Texture and Temporal Conditional Random Fields for Detection of Gad-Enhancing Multiple Sclerosis Lesions in Brain MRI	International Conference on Medical Image Computing and Computer-Assisted Intervention		y	T1, T1c, T2, PD, FLAIR	Conditional Random Field	BFC; BE; REG; IN:histogram equalization	n/a	n/a	n	n	MS	-	940x2	3	y	Manual, 2	consensus	Sen; PPV; avgFP
C	2013	M. M. Riad et al.	Detection of white matter lesions in cerebral small vessel disease	Medical Imaging 2013: Computer-Aided Diagnosis	Inter-rater agreement evaluated	y	T1, T2, FLAIR	Boosting	REG:FLIRT; BE:BET; BFC:MRF+EM; IN:mean-based	n/a	n/a	n	n	WML	-	100	3 (RUNDMC, van Norden et al. 2011)	n	Manual, 1-3	supervision	ROC; FROC
C	2013	M. Cabezas et al.	A Supervised Approach for Multiple Sclerosis Lesion Segmentation Using Context Features and an Outlier Map	Pattern Recognition and Image Analysis		y	T1, T2, PD, FLAIR	Boosting	BE:BET; denoising:ADF; BFC:N4; REG:affine+non-linear	n/a	n/a	n	n	MS	-	30	3 (Hospital Vall d'Hebron): 3 (Hospital Josep Trueta)	y	Manual, 1		DSC; TPR
C	2013	A. Bianchi et al.	Brain tumor segmentation with symmetric texture and symmetric intensity-based decision forests	Proceedings / IEEE International Symposium on Biomedical Imaging: from nano to macro. IEEE International Symposium on Biomedical Imaging		y	T1, T1c, T2, FLAIR	Decision Tree	IN:mode matching (shifting)	2.4GHz 16GB RAM	1.5 sec	n	n	BT	HGG	20 +50 synthetic	1 (BraTS'12)	y	-		DSC
C	2013	J. Mitra et al.	Classification Forests and Markov Random Field to Segment Chronic Ischemic Infarcts from Multimodal MRI	Multimodal Brain Image Analysis		y	T1, T2, FLAIR, DWI (ADC)	Random Forest, Markov Random Field	resampling; REG; BFC and tissue maps:ref; BE:masking with tissue maps	3.2GHz 23.5GB	13.5 min	n	n	S	-	17	3	n	Manual, 1		DSC; PPV; NPV; Acc
C	2013	M. B. Salah et al.	Fully Automated Brain Tumor Segmentation Using Two MRI Modalities	Advances in Visual Computing	Segmentation failure	y	T1c, FLAIR	Thresholding	BE:in paper; resampling	n/a	n/a	n	n	BT	GBM	19	3 (Cross Cancer Institute, Alberta)	n	Manual, multiple		DSC
C	2013	N. K. Subbanna et al.	Hierarchical Probabilistic Gabor and MRF Segmentation of Brain Tumours in MRI Volumes	Medical Image Computing and Computer-Assisted Intervention – MICCAI 2013		y	T1, T1c, T2, FLAIR	Bayesian classification, Markov Random Field	REG: non-linear	Dell Optiplex 980 i7	~1 hr	n	n	BT	LGG; HGG	45 +65 simulated	1 (BraTS'12)	y	-		DSC
C	2013	I. Diaz et al.	An automatic brain tumor segmentation tool	Annual International Conference of the IEEE Engineering in Medicine and Biology Society		y	T1, T1c, T2, FLAIR	Thresholding, Other	BE:thresholding; IN:histogram matching	1.73 GHz, 6GB RAM	~50 sec	n	n	BT	GBM	16 annotated (+ 60 not)	3 (Cross Cancer Institute, Alberta)	n	Manual, multiple		DSC
C	2013	H. Boussaid et al.	Rapid Mode Estimation for 3D Brain MRI Tumor Segmentation	Energy Minimization Methods in Computer Vision and Pattern Recognition		n/a	n/a	Graph Cut, Other	REG:rigid(medInria)	2.67GHz single core	19 sec	n	n	BT	LGG	113	3	n/a	Manual		DSC
C	2012	V. G. Kanas et al.	Combining Outlier Detection with Random Walker for Automatic Brain Tumor Segmentation	Artificial Intelligence Applications and Innovations		y	T1, T1c, FLAIR	Random Walker	denoising; BFC; REG; BE; IN:histogram matching	n/a	n/a	n	n	BT	meningiona, G, GBM	26	3 (Zacharaki et al. 2009)	n	Expert segmentation		DSC
C	2012	R. S. Ananda and T. Thomas	Automatic segmentation framework for primary tumors from brain MRIs using morphological filtering techniques	International Conference on BioMedical Engineering and Informatics		y	T1-FLAIR, T2	Thresholding, Other	BE:morphological operations	n/a	n/a	n	n	BT	-	50	3 (Sree Chitra Institute of Medical Sciences and Technology (SCIMST)); 3 (Regional Cancer Centre, Thiruvananthapuram, Kerala, Indi)	y	Manual, 1		TI
C	2012	S. Parisot et al.	Joint Tumor Segmentation and Dense Deformable Registration of Brain MR Images	Medical Image Computing and Computer-Assisted Intervention – MICCAI 2012		n	FLAIR	boosting, Markov Random Field	BE; IN:matching median and interquartile range to reference; REG:rigid	n/a	~6 min	n	n	BT	-	93	3	n/a	Manual, 1		DSC; FP; other
C	2012	Z. Karimaghaloo et al.	Hierarchical Conditional Random Fields for Detection of Gad-Enhancing Lesions in Multiple Sclerosis	Medical Image Computing and Computer-Assisted Intervention – MICCAI 2012		n	T1c	Conditional Random Field	BFC; BE; REG; IN	n/a	n/a	n	n	MS	MS	122	3	y	Manual, 2	consensus	Sen; PPV; FP
C	2012	D. Zikic et al.	Decision Forests for Tissue-Specific Segmentation of High-Grade Gliomas in Multi-channel MR	Medical Image Computing and Computer-Assisted Intervention – MICCAI 2012	Fully automatic pre-processing	y	T1, T1c, T2, DTI, FLAIR	Decision Tree, GMM	BE:ref; REG:affine; IN:mean intensity matching	n/a	n/a	n	n	BT	HGG	40	3	n	Manual		DSC
C	2012	D. Zikic et al.	Context-sensitive classification forests for segmentation of brain tumor tissues	Medical Image Computing and Computer-Assisted Intervention – MICCAI 2012		y	T1, T1c, T2, FLAIR	GMM, Decision Tree	BFC: N3; IN: global multiplicative factor	single desktop PC	1-2 min	n	n	BT	LGG, HGG	30 (+25 synthetic)	1 (BraTS'12)	y	-		DSC; Error; Volume Error
C	2012	N. Behzadfar and H. Soltanian-Zadeh	Automatic segmentation of brain tumors in magnetic resonance images	IEEE-EMBS International Conference on Biomedical and Health Informatics		y	T1, T1c, T2, FLAIR	Thresholding, Region Growing, Other,	IN:scaling; BE: LPF + Rodler's method + moprhological operations	n/a	n/a	n	n	BT	GBM	12	3 (Henry Ford Health System, Detroit, MI, USA)	n	Manual, 1		VC; VR
C	2012	I. Mehmood et al.	Automatic Segmentation of Region of Interests in MR Images Using Saliency Information and Active Contours	IT Convergence and Security		n/a	n/a	Other, Active Contour	BE:McStrip	n/a	n/a	n	n	BT	T	n/a (synthetic?); 10	1 (SPL); 3	y	Manual		FROC; DSC
C	2011	S. Bauer et al.	Fully Automatic Segmentation of Brain Tumor Images Using Support Vector Machine Classification in Combination with Hierarchical Conditional Random Field Regularization	Medical Image Computing and Computer-Assisted Intervention – MICCAI 2011		y	T1, T1c, T2, FLAIR	Support Vector Machine, Conditional Random Field	REG:rigid; BE:ref; denoising:edge-preserving smoothing filter; BFC	single CPU 2.33GHz	<120 sec	n	n	BT	-	10	3 (ContraCancrum brain tumor database, Marias et al. 2011)	n	Manual		DSC
C	2010	C. Elliott et al.	Bayesian Classification of Multiple Sclerosis Lesions in Longitudinal MRI Using Subtraction Images	Medical Image Computing and Computer-Assisted Intervention – MICCAI 2010	Scan-rescan reproducibility	y	T1, T2, PD	Bayesian classification	BFC; BE; REG:rigid	n/a	n/a	n	n	MS	-	3x2; 89x(2-4)	3	y	Semiautomatic; Manual		Sen
C	2010	E. Bilotta et al.	A CNN Based Algorithm for the Automated Segmentation of Multiple Sclerosis Lesions	Applications of Evolutionary Computation		n	FLAIR	Artificial neural networks, Genetic Algorithm	tissue maps:SPM8; BE:using WM map	n/a	n/a	n	n	MS	-	11	3	n	Manual, 1		DSC
C	2010	T. Wang et al.	Fully automatic brain tumor segmentation using a normalized Gaussian Bayesian Classifier and 3D Fluid Vector Flow	IEEE International Conference on Image Processing		n/a	n/a	GMM, Other	BE:MIPAV; REG:MIPAV	n/a	~430 sec	n	n	BT	-	10	1 (SPL)	n/a	-		TI
C	2009	J. Liu et al.	Automatic Multiple Sclerosis detection based on integrated square estimation	IEEE Computer Society Conference on Computer Vision and Pattern Recognition Workshops		y	T2, FLAIR	Expectation-Maximization Gausian mixture model, Other	BE:BET; IN:SPM2	1.66GHz 2GB RAM	2 min	n	n	MS	MS	16	3 (University of Kentucky Hospital)	n	Manual, 1		VD; DSC; Sen; Spe
C	2008	E. I. Zacharaki et al.	Measuring Brain Lesion Progression with a Supervised Tissue Classification System	Medical Image Computing and Computer Assisted Intervention – MICCAI 2008	Indirect reproducibility evaluation	y	T1, T2, PD	Support Vector Machine, Boosting	REG:FSL(NMI); BE:BET; BFC:N3; IN:histogram transformation; denoising:Gaussian filter	n/a	n/a	n	n	WML	DM	19 + 23x2	3	n	Manual		other
C	2008	Y. Uchiyama et al.	Automatic segmentation of different-sized leukoaraiosis regions in brain MR images	SPIE Medical Imaging		y	T1, T2	K-Means, Other	BE:region growing	n/a	n/a	n	n	WML	-	73	3 (Gero Hot Springs Hospital, Japan)	n	Manual		ROC

For peer review only

C	2007	X. Xuan and Q. Liao	Statistical Structure Analysis in MRI Brain Tumor Segmentation	International Conference on Image and Graphics		y	T1, T2, FLAIR	boosting	BE; IN	n/a	n/a	n	n	BT	G	10	3	n	Manual		other
C	2007	J. J. Corso et al.	Detection and Segmentation of Pathological Structures by the Extended Graph-Shifts Algorithm	Medical Image Computing and Computer Assisted Intervention – MICCAI 2007		y	T1, T1c, T2, FLAIR	Other	REG;BE;IN:FSL	n/a	~1 min	n	n	BT, MS	GBM; MS	20; 12	3	n/a	Manual		Jcc; PPV; Sen; DR
C	2007	N. Hevia-Montiel et al.	Robust Nonparametric Segmentation of Infarct Lesion from Diffusion-Weighted MR Images	Annual International Conference of the IEEE Engineering in Medicine and Biology Society		n	DWI	Other	ADC thresholding	n/a	n/a	n	n	S	S	15	3 (La Salpêtrière hospital, Paris, France)	n/a	Manual, 1		VC; TI
C	2006	Z. Lao et al.	Automated segmentation of white matter lesions in 3D brain MR images, using multivariate pattern classification	3rd IEEE International Symposium on Biomedical Imaging: Nano to Macro, 2006.	Inter-rater agreement evaluated	y	n/a	Support Vector Machine, Boosting	REG:ITK(MI); BE:BET; BFC:histogram matching; smoothing:Gaussian kernel	n/a	n/a	n	n	WML	-	45	3 (ACCORD-MIND)	n/a	Manual, 2		VC; other
C	2006	R. Harmouche et al.	Bayesian MS Lesion Classification Modeling Regional and Local Spatial Information	18th International Conference on Pattern Recognition (ICPR'06)	Inter-rater agreement evaluated	y	T1, T2, PD	Bayesian classification, Markov Random Field	BFC:N3; REG:ref; BE:ref; IN:ref	n/a	n/a	n	n	MS	MS	10	3	n/a	Manual, 5	majority	DSC
C	2005	K. M. Iftekharuddin et al.	Automatic brain tumor detection in MRI: methodology and statistical validation	SPIE Medical Imaging		n	T1	Self Organising Maps, Artificial neural networks	n/a	n/a	n/a	n	n	BT	-	50	3	n	Manual		ROC; DSC; Jcc; other
C	2005	M. Schmidt et al.	Segmenting brain tumors using alignment-based features	International Conference on Machine Learning and Applications		n/a	n/a	Support Vector Machine	denoising:non-linear filtering; BFC:N3; IN:weighted regression; REG:SPM	n/a	n/a	n	n	BT	-	10	3	y	Manual, 1		Jcc
C	2004	W. Li et al.	Automatic segmentation of cerebral ischemic lesions from diffusion tensor MR images	Medical Imaging 2004: Image Processing	Inter-rater agreement evaluated	n	DTI	Other	denoising:non-linear filtering	n/a	n/a	n	n	S	S	20	3	y	Manual		SI
C	2003	A. Hadjiprocopis and P. Tofts	An automatic lesion segmentation method for fast spin echo magnetic resonance images using an ensemble of neural networks	IEEE XIII Workshop on Neural Networks for Signal Processing		y	T1, T2, PD	Artificial neural networks	n/a	n/a	n/a	n	n	MS	-	20	3	y	Manual		Sen; Spe
C	2003	W. Li and J. Tian	Automatic segmentation of brain infarction in diffusion-weighted MR images	Medical Imaging 2003: Image Processing	Inter-rater agreement evaluated	n	DWI	Other	denoising:ADF; REG:global-rigid + local-non-rigid	n/a	n/a	n	n	S	-	20	3	y	Manual		DSC
C	2013	J. Huang et al.	Brain Tumor Segmentation Based on Texture, Intensity, and Edge	World Congress on Medical Physics and Biomedical Engineering		y	T1c, T2	Other	n/a	n/a	n/a	n	n	BT	-	19	3	n/a	Manual, multiple		TP; FP; FN; other
J	2012	Z. Karimaghaloo et al.	Automatic Detection of Gadolinium-Enhancing Multiple Sclerosis Lesions in Brain MRI Using Conditional Random Fields	IEEE Transactions on Medical Imaging		y	T1, T1c, T2, PD, FLAIR	Conditional Random Field, Graph Cut	REG:ref; BFC:N3; IN:histogram matching; BE:ref	2.67GHz quad-core P4 desktop	~5 sec	n	n	MS	MS	80	3	n/a	Manual, 2	consensus	Sen; FP; PPV
J	2017	Y. Liu et al.	A deep convolutional neural network-based automatic delineation strategy for multiple brain metastases stereotactic radiosurgery	PloS one		intended monomodality	T1c, FLAIR	Artificial neural networks	BE:ROBEX	NVIDIA Quadro M2000M Intel Xeon E3-1505	<2 min	n	n	MET	-	265; 240	1 (BraTS); 3 (University of Texas Southwestern Medical Center, Wardak et al. 2016)	y	Maunal, 1		DSC; avgnSSD; sdSSD; Sen; Spe; ROC
J	2017	S. Mitra et al.	Volumetric brain tumour detection from MRI using visual saliency	PloS one		y	T1c, T2, FLAIR	Other	IN; 8bit range	n/a	n/a	n	n	BT	LGG; HGG	274	1 (BraTS'15)	y	-		ROC
C	2016	M. Agn et al.	A generative model for segmentation of tumor and organs-at-risk for radiation therapy planning of glioblastoma patients	SPIE Medical ImagingMedical Imaging 2016: Image Processing	Organs at Risk segmentation	y	T1c, T2, FLAIR	Artificial neural networks	REG	Core i5-2400 3.1GHz CPU	2 hrs	n	n	BT	LGG, HGG; GBM	30; 20	1 (BraTS'13); 3 (Rigshospitalet, Copenhagen University Hospital)	y	Manual		DSC; TPR; PPV
J	2019	K. R. Laukamp et al.	Fully automated detection and segmentation of meningiomas using deep learning on routine multiparametric MRI	European Radiology		y	T1, T1c, T2, FLAIR	Artificial neural networks	BFC; REG; BE; resampling; IN:0u1v	n/a	n/a	n	n	BT	meningioma	136	3 (University Hospital of Cologne)	y	Semiautomatic		DSC
J	2015	A. Karimian and S. Jafari	A New Method to Segment the Multiple Sclerosis Lesions on Brain Magnetic Resonance Images	J Med Signals Sens		y	T1, T2, FLAIR	GMM	BFC:LPF; BE:morphological operations	n/a	n/a	n	n	MS	-	25	3	y	Manual		DSC; Acc; Sen; Spe
J	2014	R. Wang et al.	Automatic Segmentation and Quantitative Analysis of White Matter Hyperintensities on FLAIR Images Using Trimmed-Likelihood Estimator	Academic Radiology		n	FLAIR	Other	BFC:N3; BE:BET	n/a	n/a	n	y	WML	-	82	3	n	Manual, 1		DSC; FPR; FNR; VR; VD
J	2018	X. Zhao et al.	A deep learning model integrating FCNNs and CRFs for brain tumor segmentation	Medical Image Analysis		y	T1, T1c, T2, FLAIR	Artificial neural networks, Conditional Random Field	IN:in paper; BFC:N4	Tesla K80 GPU, Inter E5-2620 CPU	2-4 min	n	n	BT	LGG, HGG	65+31; 274+110; 191	1 (BraTS'13); 1 (BraTS'15); 1 (BraTS'16)	y	-		DSC; PPV; Sen
C	2013	L. Zhao et al.	Automatic brain tumor segmentation with MRF on supervoxels	Multimodal Brain Tumor Segmentation		y	T1, T1c, T2, FLAIR	Supervoxel, Markov Random Field, Graph Cut	Denoising: SUSAN; IN: z-score	n/a	n/a	n	n	BT	LGG; HGG	65+31	1 (BraTS'13)	y	-		DSC; Spe; Sen
J	2018	R. Zhng et al.	Automatic segmentation of acute ischemic stroke from DWI using 3D Fully Convolutional DenseNets	IEEE Trans Med Imaging		y	DTI, ADC	Artificial neural networks	IN:0u1v, zero-padding, cropping, resampling	I7-4790 3.6GHz, 16GB RAM CPU, Nvidia Titan X GPU	~0.1 sec	n	n	S	S	242; 64	1 (ISLES'15); 3	y	Manual, 2	supervision	DSC; Sen; F1; PPV; Sen; VD
J	2017	T. Zhan et al.	Multimodal spatial-based segmentation framework for white matter lesions in multi-sequence magnetic resonance images	Biomedical Signal Processing and Control		y	T1, T2, FLAIR	Logistic Regression, Markov Random Field, Bayesian classification	BE: BET; BFC: N4; IN:linear contrast adjustment	n/a	n/a	n	n	WML	-	50; 54	1 (MS GC MICCAI'08); 3 (ACCORD-MIND)	y	Manual, 1		DSC; TPR; TNR; VD; AD; ROC
C	2012	E. Zacharaki et al.	Fuzzy Multichannel clustering with individualized spatial priors for segmenting brain lesions and infarcts	Artificial Intelligence Applications and Innovations		y	T1, FLAIR	K-Means, Fuzzy C-Means	BE; BFC; IN; REG:HAMMER	n/a	n/a	n	n	S, NEC	DM	47 (+72 healthy)	3	y	Manual, 1&2		DSC
C	2016	Y. Yoo et al.	Deep learning of brain lesion patterns for predicting future disease activity in patients with early symptoms of multiple sclerosis	Deep Learning and Data Labeling for Medical Applications		y	T2, PD	Artificial neural networks, Euclidean Distance Transform	BE; IN: linear; REF: affine	n/a	n/a	n	n	MS	early MS	140	3	n/a	Semiautomatic		Acc; Sen; Spe; ROC
preprint	2016	D. Yi, et al.	3-D convolutional neural networks for glioblastoma segmentation	arXiv preprint		y	T1, T1c, T2, FLAIR	Artificial neural networks	n/a	n/a	n/a	n	n	BT	LGG; HGG	274 + n	1 (BraTS'13); 1 (BraTS'15)	y	-		DSC
J	2014	W. Wu et al.	Brain tumor detection and segmentation in a conditional random fields framework with pixel-pairwise affinity and superpixel-level features	International Journal of Computer Assisted Radiology and Surgery		y	T1, T1c, T2, FLAIR	Superpixel, Gabor wavelets, SVM, Conditional Random Field	n/a	single desktop PC	30 min	n	n	BT	GBM	20; 30 (+25 synthetic)	1 (BraTS'12); 3 (Corso et al. 2008)	y	n/a		Acc; PPV; Sen; Jcc; DSC
J	2007	C. Vijayakumar et al.	Segmentation and grading of brain tumors on apparent diffusion coefficient images using self-organizing maps	Computerized Medical Imaging and Graphics		y	ADC, T2, FLAIR	Self Organising Maps	REG; IN; BE	n/a	n/a	n	n	BT	LGG; HGG	10	3	n	Manual, 2		ROC; Sen; Spe
J	2008	R. Verma et al.	Multi-parametric tissue characterization of brain neoplasms and their recurrence using pattern classification of MR images	Academic Radiology		y	B0, DWI, FLAIR, T1, T1c, FA, ADC	Bayesian classification, SVM	BE:FSL; REG:FLIRT; IN: histogram matching	n/a	n/a	n	n	BT	HGG	14	3	y	Manual, 1		Sen; Spe; other
C	2015	S. Vaidya et al	Longitudinal multiple sclerosis lesion segmentation using 3D convolutional neural networks	2015 Longitudinal Multiple Sclerosis Lesion Segmentation Challenge		y	T1, T2, PD, FLAIR	Artificial neural networks	n/a	Nvidia Tesla K20 GPU	2 min	n	n	MS	MS	5xn; 14xn	1 (MSC'15)	n	-		DSC; Jcc; PPV; Sen; TPR; FPR; VD; SD; VC
J	2017	K. Usman et al	Brain tumor classification from multi-modality MRI using wavelets and machine learning	Pattern Analysis and Applications		y	T1, T1c, T2, FLAIR	Decision Tree	IN: histogram matching; bounding box of tumor	I5, 2.5GHz, 8 GB RAM	3 min	n	n	BT	LGG, HGG	30	1 (BraTS'13)	y	-		DSC; Jcc; Spe
C	2014	G. Urban et al.	Multi-modal brain tumor segmentation using deep convolutional neural networks	MICCAI BraTS (brain tumor segmentation) challenge		y	T1, T1c, T2, FLAIR	Artificial neural networks	IN: mean CSF normalization	n/a	n/a	n	n	BT	LGG, HGG	30	1 (BraTS'13)	y	-		DSC
J	2015	N. J. Tustison et al.	Optimal symmetric multimodal templates and concatenated random forests for supervised brain tumor segmentation (simplified) with ANTsR	Neuroinform		y	T1, T1c, T2, FLAIR	Decision Tree, MAP, Markov Random Field	IN: 0-1range	Single threaded cluster	2 hrs	n	y	BT	LGG, HGG	55	1 (BraTS'13)	Y	-		DSC; PPV; Sen
C	2013	T. Taylor et al.	Map-reduce enabled hidden Markov models for high throughput multimodal brain tumor segmentation	Multimodal Brain Tumor Segmentation		y	T1, T1c, T2, FLAIR	Hidden Markov Models	movement artefact removal; BFC; IN	n/a	~500 sec	n	n	BT	HGG	20 (+25 synthetic)	1 (BraTS'13)	y	-		DSC
C	2009	N. Subbanna et al.	MS lesion segmentation using Markov Random Fields	MICCAI 2009		y	T1, T2, PD	Markov Random Field	BFC: N3; REG:ref; BE:ref; IN	n/a	n/a	n	n	MS	MS	24	3	n	Manual, 5	consensus	DSC; FPR; FNR
C	2015	N. Subbanna et al.	IMaGe: Iterative multilevel probabilistic graphical model for detection and segmentation of multiple sclerosis lesions in brain MRI	Information Processing in Medical Imaging		y	T1, T2, PD, FLAIR	Markov Random Field	BFC: N3; REG:ref; BE:FSL; IN:histogram matching	Dell Optiplex 980 i7	~40 min	n	n	MS	MS	1195	3	y	Semiautomatic		PPV; Sen; DSC
C	2008	J. C. Souplet	An automatic segmentation of T2-FLAIR multiple sclerosis lesions	MICCAI 2008		y	T1, T2, FLAIR	Expectation-Maximization, outlier detection	cropping; BE:ref; BFC:ref	n/a	96 min	n	y	MS	MS	25	1 (MS GC MICCAI'08)	y	-		VD; AD; TP; FP
C	2016	R. Saha et al.	Brain tumor segmentation from multimodal mr images using rough sets	Computer Vision, Graphics, and Image Processing		y	T1c, T2, FLAIR	Rough set, k-means, Quadtree partitioning	n/a	n/a	n/a	n	n	BT	LGG, HGG	304	1 (BraTS'13); 1 (BraTS'15)	y	-		PPV; Sen; DSC

For peer review only

C	2007	J. Rexilius et al.	Multispectral brain tumor segmentation based on histogram model adaptation	SPIE Medical Imaging	Semiautomatic preprocessing (brain extraction)	y	T2, FLAIR	tumor model, region growing	movement artefact removal; REG: affine; BE: watershed	2GHz AMD Athlon	10 min	n	n	BT	-	12	3	y	Manual		Jcc
C	2012	K. K. Reddy	Confidence guided enhancing brain tumor segmentation in multi-parametric MRI	ISBI		y	T1, T1c, T2, FLAIR	SVM, AdaBoost, Level Set, Region Growing	n/a	n/a	n/a	n	n	BT	-	11	3	n	Manual, 1		TPR; FPR; DSC
C	2011	P. Raniga et al.	Local intensity model: An outlier detection framework with applications to white matter hyperintensity segmentation	ISBI		y	T2, FLAIR	local intensity model, outlier detection	BFC:ref; REG:rigid; IN: CSF and WM normalization	n/a	n/a	n	n	WML	MCI, AD	14 (+44 healthy elderly)	3 (AIBL, Ellis et al., 2009)	n	Manual, 1		DSC; Overlap fraction; Extra fraction; missed fraction
C	2016	R. P. Randhawa	Improving segment boundary classification for brain tumor segmentation and longitudinal disease progression	Brainlesion: Glioma, Multiple Sclerosis, Stroke and Traumatic Brain Injuries		y	T1, T1c, T2, FLAIR	Artificial neural networks	BFC:N4; IN:0u1v	n/a	6 min	n	n	BT	LGG, HGG	274	1 (BraTS'15)	y	-		DSC
C	2015	O. Puonti	Simultaneous whole-brain segmentation and white matter lesion detection using contrast-adaptive probabilistic models	Brainlesion: Glioma, Multiple Sclerosis, Stroke and Traumatic Brain Injuries	Minimal preprocessing	y	T1, T2, FLAIR	Restricted Boltzmann machine, GMM	none	Quad-core Xeon 5472 3GHz 32 RM, GTX Titan 6 GB GPU	2 hrs	n	n	WML	MS	20	1 (MS GC MICCAI'08)	y	-		PPV; TPR
J	2006	K. B. Prakash	Identification, segmentation, and image property study of acute infarcts in diffusion-weighted images by using a probabilistic neural network and adaptive gaussian mixture model	Academic Radiology		n	DWI	Artificial neural networks, GMM	n/a	n/a	1 min	n	n	S	-	13	3	n/a	Semiautomatic		DSC; Sen; Spe
C	2015	V. Pedoia	Fully automatic brain tumor segmentation by using competitive EM and Graph Cut	Image Analysis and Processing – ICIAP		n/a	FLAIR	Competitive Expectation-Maximization, Graph cut	n/a	n/a	n/a	n	n	BT	LGG, HGG	20; 30 (+50 synthetic)	1 (BraTS'12); 3	y	Manual, 6		Jcc; DSC
C	2012	S. Parisot et al.	Graph-based Detection, Segmentation and Characterisation of Brain Tumours	IEEE Conference on Computer Vision and Pattern Recognition		n	FLAIR	Sparse graph	IN: mean and IQ based	n/a	n/a	n	n	BT	LGG	113	3	n/a	Manual		DSC
C	2016	B. Pandian et al.	Multimodal tumor segmentation with 3D convolutional neural networks	MICCAI BRATS		y	T1, T1c, T2, FLAIR	Artificial neural networks	BFC:N4; IN: 0u1v	4 core i7 32GB RAM, NVIDIA GTX 1080 GPU	n/a	n	n	BT	-	40	1 (BraTS'16)	y	-		DSC
C	2008	J. Morra et al.	Automatic segmentation of MS lesions using a contextual model for the MICCAI grand challenge	MICCAI 2008		y	T1, T2, FLAIR, DTI	Probabilistic boosting tree	downsampling, BFC: ref	n/a	n/a	n	n	MS	MS	25	1 (MS GC MICCAI'08)	y	-		VD; AD; Sen; Spe; PPV
C	2015	R. Meier, et al.	Parameter Learning for CRF-based Tissue Segmentation of Brain Tumors	Brainlesion: Glioma, Multiple Sclerosis, Stroke and Traumatic Brain Injuries		y	T1, T1c, T2, FLAIR	Conditional Random Field, Decision trees	n/a	n/a	n/a	n	n	BT	LGG, HGG	24x2; 20; 53	1 (BraTS'13); 1 (BraTS'15)	y	-		DSC; VE
C	2016	R. Meier, et al.	CRF-based brain tumor segmentation: alleviating the shrinking bias	Brainlesion: Glioma, Multiple Sclerosis, Stroke and Traumatic Brain Injuries		y	T1, T1c, T2, FLAIR	Dense Conditional Random Field, Decision trees	n/a	n/a	n/a	n	n	BT	LGG; HGG	51; 357; 25	1 (BraTS'13); 1 (BraTS'15)	y	-		DSC; VE
C	2016	O. Maier at al.	Image features for brain lesion segmentation using random forests	Brainlesion: Glioma, Multiple Sclerosis, Stroke and Traumatic Brain Injuries		y	T1, T1c, T2, FLAIR, DWI, CBF, CBV, TTP, Tmax	Random Forest	BFC:CMTK; IN:intensity harmonization	n/a	n/a	n	n	BT, S	S, LGG, HGG	104; 274	1 (BraTS'15); 1 (ISLES'15)	y	-		DSC; avgSSD; Hdff
C	2016	T .K. Lun et al.	Brain tumor segmentation using deep convolutional neural network	MICCAI BRATS		y	T1, T1c, T2, FLAIR	Artificial neural networks	n/a	n/a	n/a	n	n	BT	-	274	1 (BraTS'15)	y	-		DSC; VE
C	2016	L. Lefkovits et al.	Brain Tumor Segmentation with Optimized Random Forest	Brainlesion: Glioma, Multiple Sclerosis, Stroke and Traumatic Brain Injuries		y	T1, T1c, T2, FLAIR	Random Forest	denoising: ADF; BFC:N4; IN;histogram matching	n/a	n/a	n	n	BT	LGG; HGG	274	1 (BraTS'15)	y	-		DSC
J	2008	Z. Lao et al.	Computer-assisted segmentation of white matter lesions in 3D MR images using support vector machine	Academic Radiology	Inter-rater agreement evaluated	y	T1, T2, PD, FLAIR	SVM	REG:affine; BE:BET; BFC:N3; IN:histogram matching	n/a	n/a	n	n	WML	DM	45	3 (ACCORD-MIND)	y	Manual, 1		ROC; VC
C	2016	S. Kumar et al.	A picture fuzzy clustering approach for brain tumor segmentation	International Conference on Cognitive Computing and Information Processing (CCIP)		n/a	n/a	Fuzzy set	n/a	n/a	n/a	n	n	BT	LGG; HGG	65 (+65 synthetic)	1 (BraTS)	y	-		Jcc; DSC
C	2008	D. Kroon	Multiple sclerosis detection in multispectral magnetic resonance images with principal components analysis	Grand Challenge Work.: Mult. Scler. Lesion Segm. Challenge		y	T1, T2, FLAIR, MD, FA	PCA, log.likelihood ratio	BFC: various	n/a	n/a	n	n	MS	MS	44	1 (MS GC MICCAI'08)	y	-		DSC; VD; AD; Sen; Spe; PPV
C	2016	J. Knight et al.	MS lesion segmentation using FLAIR MRI only	MICCAI-MSSEG		n	FLAIR	edge detection, Fuzzy classification	BFC:SPM12; denoising:Gaussian LPF	n/a	n/a	n	n	MS	MS	15	1 (MS Lesion Segmentation Challenge '16)	y	-		DSC; PPV; TPR; Bland-Altman; ANCOVA
C	2007	H. Khotanlou et al.	Automatic brain tumor segmentation using symmetry analysis and deformable models	Advances in Pattern Recognition		n	T1	symmetry plane, histogram subtraction, deformable model	n/a	n/a	n/a	n	n	BT	-	10	3	n	Manual		Hdff; SD; other
C	2015	A. Jesson et al.	Hierarchical MRF and random forest segmentation of MS lesions and healthy tissue in brain MRI	Proceedings of the 2015 Longitudinal Multiple Sclerosis Lesion Segmentation Challenge		y	T1, T2, FLAIR	Random Forest, pathological atlases	IN:sigmoid; denoising:non-local means	n/a	n/a	n	n	MS	-	5x21	1 (MSC'15)	y	-		DSC; Jcc; TPR; FPR
C	2015	T. Jerman et al.	Combining unsupervised and supervised methods for lesion segmentation	Brainlesion: Glioma, Multiple Sclerosis, Stroke and Traumatic Brain Injuries		y	T1, T2, FLAIR	GMM, Decision trees	BE:reg; REG:affine; BFC:N4; IN:histogram matching; resampling:iso 1mm3 vox	n/a	n/a	n	n	MS	-	18	3 (University Medical Centre Ljubljana)	n	Manual, 2	consensus	DSC; TPR; PPV
J	2014	V. Ithapu et al.	Extracting and summarizing white matter hyperintensities using supervised segmentation methods in Alzheimer's disease risk and aging studies	Human Brain Mapping		n	FLAIR	SVM, Random Forest	REG; tissue seg:SPM12	n/a	n/a	n	y	WML	MCI, AD	38	3 (Wisconsin Alzheimer's Disease Research Center)	n	Semiautomatic		ROC; F score; other
J	2013	A. Islam et al.	Multifractal Texture Estimation for Detection and Segmentation of Brain Tumors	IEEE Transactions on Biomedical Engineering		y	T1, T2, FLAIR	boosting	REG:SPM8; BFC:SPM8; IN:histogram matching; BE:BET	n/a	n/a	n	n	BT	Astrocytoma, medulloblastoma	14 ;14	1 (BraTS'12); 3	y	Manual		ROC; DSC; Jcc; TI; other
J	2018	S. Iqbal et al.	Brain tumor segmentation in multi-spectral MRI using convolutional neural networks	Microscopy Research & Technique		y	T1, T1c, T2, FLAIR	Artificial neural networks	IN:0-255 range & mean subtraction; BFC:N4; cropping	Titan X 12 GB RAM GPU	<1 sec	n	n	BT	LGG, HGG	274	1 (BraTS'15)	y	-		DSC; Sen; Spe
J	2016	E. Ilunga-Mbuyamba et al.	Active contours driven by Cuckoo Search strategy for brain tumour images segmentation	Expert Systems with Applications		n	T1	Active Contour, Cuckoo Search Strategy	n/a	1.9GHz 4GB RAM	~180 sec	n	n	BT	GBM, MET	10	3 (University Hospital, Department of Neurosurgery, University of Leipzig, Germany)	n	Manual, 1		DSC; Jcc; Hdff
J	2012	K. M. Hulsey et al.	Automated quantification of white matter disease extent at 3T: Comparison with volumetric readings	Journal of Magnetic Resonance Imaging		y	MP-RAGE, FLAIR	thresholding	BE:FSL; tissue seg:Sienax; REG:affine	n/a	n/a	n	n	WML	-	28	3 (Dallas Heart Study, Ronald et al 2004)	n	Manual, 2		VC; VR; Bland-Altman
C	2015	A. Hoogi et al.	Multimodal Brain Tumor Segmentation (BRATS) using Sparse Coding and 2-layer Neural Network	MICCAI BRATS		y	T1, T1c, T2, FLAIR	Artificial neural networks, Sparse coding	IN: mean subtraction	n/a	n/a	n	n	BT	LGG, HGG	100	1 (BraTS'15)	y	-		DSC
J	2011	H. Khotanlou et al.	Segmentation of multiple sclerosis lesions in brain MR images using spatially constrained possibilistic fuzzy c-means classification	J Med Signals Sens		y	T1, T2	probabilistic fuzzy c-means	n/a	n/a	n/a	n	n	MS	-	10	3 (Imam Khomeini hospital)	n	Manual		DSC; Overlap fraction; Extra fraction
C	2015	T. Haeck et al.	Automated model-based segmentation of brain tumors in MR images	MICCAI BRATS		y	T1, T1c, T2, FLAIR	Expectation-Maximization, level set	BFC; Brain mask erosion	2 x 2.66 GHz Quad Core CPU	15 min	n	n	BT	LGG, HGG	274	1 (BraTS'15)	y	-		DSC
C	2015	T. Haeck et al.	Automated model-based segmentation of ischemic stroke in MR images	Brainlesion: Glioma, Multiple Sclerosis, Stroke and Traumatic Brain Injuries		y	T1, TTP, FLAIR	Expectation-Maximization, level set	none	2 x 2.66 GHz Quad Core CPU	15 min	n	n	S	-	64; 50	1 (ISLES'15)	y	-		DSC; avgSSD; Hdff; PPV; Sen
J	2018	N. Gupta et al.	Identification of gliomas from brain mri through adaptive segmentation and run length of centralized patterns	Journal of Computational Science		y	T1, T1c, T2, FLAIR	Image fusion, Thresholding	denoising: ADF	n/a	n/a	n	n	BT	LGG; HGG	80, 134	1 (BraTS'13); 3 (NSCB Medical College Jabalpur, India)	y	n/a		Jcc; DSC; Hdff; other
C	2014	M. Goetz et al.	Extremely randomized trees based brain tumor segmentation	MICCAI BRATS		y	T1, T1c, T2, FLAIR	Decision Tree	BFC:N4; IN:histogram matching + mode subtraction and 1v	n/a	n/a	n	n	BT	LGG; HGG	30	1 (BraTS'13)	y	-		DSC
J	2016	M. Ghafoorian et al.	Location sensitive deep convolutional neural networks for segmentation of white matter hyperintensities	Scientific Reports	Inter-rater agreement evaluated	y	T1, FLAIR	Artificial neural networks	REG:FLIRT; BE:BET; BFC:FAST; IN:0-1 range	n/a	n/a	n	n	WML	SVD	420	3 (RUNDMC, van Norden et al. 2011)	n	Manual		DSC; ROC
J	2017	M. Ghafoorian et al.	Deep multiscale location-aware 3d convolutional neural networks for automated detection of lacunes of presumed vascular origin	NeuroImage: Clinical	Inter-rater agreement evaluated	y	T1, FLAIR	Artificial neural networks	REG:FLIRT; BE:BET; BFC:FAST; IN:0-1 range	n/a	n/a	n	n	S	SVD; S	654; 421	3 (RUNDMC, van Norden et al. 2011); 3 (FUTURE, Rutten-Jacobs et al. 2011)	y	Manual, 1		FROC

For peer review only

C	2016	L. Le Folgoc et al.	Segmentation of brain tumors via cascades of lifted decision forests	MICCAI BRATS		y	T1, T1c, T2, FLAIR	Decision Tree, mixture model, Markov Random Field	n/a	Intel Xeon 3.6GHz 16GB RAM	~30 sec	n	n	BT	LGG; HGG	20; 200	1 (BraTS'13); 1 (BraTS'15)	y	-		DSC
C	2016	L. Le Folgoc et al.	Lifted auto-context forests for brain tumour segmentation	Brainlesion: Glioma, Multiple Sclerosis, Stroke and Traumatic Brain Injuries	semiautomatic preprocessing *brats 2016 contains some unpreprocessed or partially preprocessed images	y	T1, T1c, T2, FLAIR	Decision Tree, mixture model, Markov Random Field	IN:median and deviation; reg:rigid; BFC:ref; BE	Intel Xeon 3.6GHz 16GB RAM	~20 sec	n	n	BT	LGG; HGG	30;70; 274(?)	1 (BraTS'13); 1 (BraTS'15); 1 (BraTS'16)	y	-		DSC
J	2013	J. B. Fiot et l.	Efficient brain lesion segmentation using multi-modality tissue-based feature selection and support vector machines	International journal for numerical methods in biomedical engineering		y	T1, T2, PD, FLAIR	Support Vector Machine	REG:rigid; BE:ref; BFC:ref; denoising:ADF; tissue seg:EM	n/a	n/a	n	n	WML	-	125	3 (AIBL, Ellis et al., 2009)	n/a	Manual, 2	supervision	DSC; Sen; Spe
C	2013	J. Festa et al.	Automatic brain tumor segmentation of multi-sequence MR images using random decision forests	MICCAI BRATS		y	T1, T1c, T2, FLAIR	Random Forest	BFC:N4; IN:histogram matching	I7 3.2GHz 24 GB RAM	20-25 min	n	n	BT	LGG; HGG	30 +50 simulated	1 (BraTS'13)	y	-		DSC; Jcc; Sen; Spe; Kappa
C	2003	R. J. Ferrari et al.	Segmentation of multiple sclerosis lesions using support vector machines	SPIE Medical Imaging		y	T1, T2, FLAIR	Support Vector Machine	denoising: ADF; BFC:N3; IN:CSF linear normalization	2GHz 1GB RAM	~40 min	n	n	MS	-	18	3	n	Semiautomatic		Acc; FNR
C	2015	C. Feng et al.	Segmentation of ischemic stroke lesions in multi-spectral MR images using weighting suppressed FCM and three phase level set	Brainlesion: Glioma, Multiple Sclerosis, Stroke and Traumatic Brain Injuries		y	T1, T1c, T2, FLAIR, DWI, CBF, CBV, TTP, Tmax	Fuzzy C-Means, Image fusion, Level set	n/a	n/a	n/a	n	n	S	-	64; 50	1 (ISLES'15)	y	-		DSC; avgSSD; Hdff
C	2016	A. Ellawaa et al.	Brain tumor segmentation using random forest trained on iteratively selected patients	Brainlesion: Glioma, Multiple Sclerosis, Stroke and Traumatic Brain Injuries		y	T1, T1c, T2, FLAIR	Random Forest	BFC:N4; IN:histogram matching	n/a	n/a	n	n	BT	LGG; HGG	70?	1 (BraTS'16)	y	-		DSC; Sen; Spe
C	2015	P. Dvorak et al.	Local Structure Prediction with Convolutional Neural Networks for Multimodal BrainTumor Segmentation	Medical Computer Vision: Algorithms for Big Data		y	T1, T1c, T2, FLAIR	Artificial neural networks	n/a	4 core Intel Xeon E3 3.3GHz	13 sec	n	n	BT	LGG; HGG	309	1 (BraTS'14)	y	-		DSC
C	2013	S. Doyle et al.	Fully automatic brain tumor segmentation from multiple MR sequences using hidden Markov fields and variational EM	MICCAI BRATS		y	n/a	Markov Random Field, Expectation-Maximization	n/a	n/a	n/a	n	n	BT	LGG; HGG	30?	1 (BraTS'13)	y	-		DSC
J	2007	S. Datta et al.	Segmentation of gadolinium-enhanced lesions on MRI in multiple sclerosis	Journal of Magnetic Resonance Imaging	Semiautomatic preprocessing (brain extraction)	y	T1, T1c, FSE, FLAIR	Fuzzy connectedness, Other	REG:ref; BFC:SPM2; BE:ref; denoising:ADF	2.99GHz 1BG RAM	<10 min	n	n	MS	RRMS	22	3	n	Manual, 1		DSC; Bland-Altman; other
J	2012	S. Damangir et al.	Multispectral MRI segmentation of age related white matter changes using a cascade of support vector machines	Journal of Neurological Sciences		y	T1, FLAIR	Support Vector Machine	REG:FLIRT; BE:BET; BFC:N3; IN:linear transformation + 0-1 range	n/a	3 min	n	n	WML	AD, LBD	70 + 32 healthy	3 (he Dementia Study in Western Norway)	y	Manual		ROC; Sen; Spe; Acc; VC
C	2007	H. Cai et al.	Probabilistic segmentation of brain tumours based on multimodality magnetic resonance images	ISBI		y	T1, T1c, B0, DWI, FLAIR, DTI (FA, ADC)	Quadratic Discriminant Analysis, Support Vector Machine	BE:FSL; denoising:FSL; REG:FLIRT; IN:histogram matching	n/a	n/a	n	n	BT	-	22	3 (Hospital, University of Pennsylvania)	y	Expert segmentation		Acc
C	2012	C. P. Yu et al.	Statistical asymmetry-based brain tumor segmentation from 3D MR images	Biosignals	Segmentation failure	y	T1, T1c	Symmetry plane, Watershed	n/a	n/a	3 min	n	n	BT	-	17	3	n/a	Manual		Jcc
C	2016	P. Chang	Fully convolutional neural networks with hyperlocal features for brain tumor segmentation	MICCAI BRATS		y	T1, T1c, T2, FLAIR	Artificial neural networks	IN:histogram matching + 0-1 range	NVIDIA GTX TITAN X 12GB RAM	<1 sec	n	n	BT	HGG	144	1 (BraTS'16)	y	-		DSC; Hdff
J	2017	I. Cabria et al.	MRI segmentation fusion for brain tumor detection	Information Fusion		n	FLAIR	Potential Field Segmentation	n/a	n/a	n/a	n	n	BT	-	22	1 (BraTS'13)	n/a	-		other
C	2013	P. Buendia et al.	A grouping artificial immune network for segmentation of tumor images	MICCAI BRATS		y	T1, T1c, T2, FLAIR	Grouping Artificial Immune Network	denoising; BFC; IN:histogram matching	n/a	21 sec	n	n	BT	HGG	20	1 (BraTS'12)	y	-		DSC; Jcc; Sen; Spe; Kappa
J	2000	A. Boudra et al.	Automated segmentation of multiple sclerosis lesions in multispectral MR imaging using fuzzy clustering	Computers in Biology and Medicine		y	PD, T2	Fuzzy C-Means	enhancement:histogram equalization, BE:ref	n/a	n/a	n	n	MS	-	10	3	n	Manual, 2		other
C	2016	A. Birenbaum et al.	Longitudinal multiple sclerosis lesion segmentation using multi-view convolutional neural networks	Deep Learning and Data Labeling for Medical Applications		y	T1, T2, PD, FLAIR	Artificial neural networks	REG:rigid; BFC; IN:histogram matching	n/a	n/a	n	n	MS	-	19x4-6	1 (MSC'15)		-		DSC
C	2011	A. Bijar et al.	Segmentation of MS lesions using active contour model, adaptive mixtures method and mrf model	International Symposium on Image and Signal Processing and Analysis (ISPA)		y	T1, T1c, T2, FLAIR	Adaptive mixture model, Markov Random Field, Bayesian Classification	n/a	n/a	n/a	n	n	MS	-	20	3 (Khayati et al. 2008)	n/a	Manual, 2		DSC; Overlap fraction; Extra fraction
J	2011	A. Bijar et al.	Segmentation of MS lesions using entropy-based EM algorithm and Markov random fields	Journal of Biomedical Science and Engineering		y	n/a	GMM, Markov Random Field, Bayesian Classification, Expectation-Maximization	n/a	n/a	n/a	n	n	MS	-	20	3 (Khayati et al. 2008)	n/a	Manual, 2		DSC; Overlap fraction; Extra fraction
J	2008	K. B. Prakash	Automatic processing of diffusion-weighted ischemic stroke images based on divergence measures: Slice and hemisphere identification, and stroke region segmentation	International Journal of Computer Assisted Radiology and Surgery		n	DWI	intensity pdf divergence of 2 hemispheres	n/a	n/a	n/a	n	n	S	-	57	3	y	Manual, 1		ROC; DSC; Sen; Spe
preprin	2017	A. Beers et al.	Sequential 3d u-nets for biologically-informed brain tumor segmentation	arXiv preprint arXiv:1709.02967		y	T1, T1c, T2, FLAIR	Artificial neural networks	IN:0u1v	NVIDIA Tesla P100 GPU	n/a	n	n	BT	-	331	1 (BraTS'17)	y	-		DSC
C	2012	S. Bauer et al.	Segmentation of brain tumor images based on integrated hierarchical classification and regularization	MICCAI BRATS	method integrated in neuroradiology workflow	y	T1, T1c, T2, FLAIR	Random Forest, Conditional RandomFfield	n/a	n/a	4-12 min	n	n	BT	-	30?	1 (BraTS'12)	y	-		DSC; Jcc; Sen; Spe; AD; Hdff; Kappa
J	2013	A. J. Asman et al.	Of-Atlas likelihood estimation using multi-atlas segmentation	Med Phys		n	T1c	Intensity probability density function divergence of atlas	REG:FLIRT; IN: 0u1v + intensity mapping	n/a	<2 hrs	n	n	BT	-	30	3	n/a	Manual, 1		DSC; FNR; FPR
J	2008	P. Anbeek et al.	Automated MS-lesion segmentation by K-nearest neighbour classification	MIDAS Journal		y	T1, FLAIR	K-Nearest Neighbours	BE:BET	n/a	n/a	n	n	MS	-	44	3 (Children's Hospital Boston); 3 (University of North Carolina)	y	Manual, 2		VD; AD; TP; FP
J	2017	V. Alex et al.	Semisupervised learning using denoising autoencoders for brain lesion detection and segmentation	Journal of Medical Imaging		Y	T1, T1c, T2, FLAIR, DWI	Artificial neural networks	IN: histogram matching + 0u1v	n/a	n/a	n	n	BT	LGG; HGG; S	257; 28	1 (BraTS'15); 1 (ISLES'15)	y	-		DSC
C	2017	V. Alex et al.	Generative adversarial networks for brain lesion detection	SPIE Medical Imaging		y	T1, T1c, T2, FLAIR, DWI	Artificial neural networks	IN: histogram matching + 0u1v	n/a	n/a	n	n	BT	LGG; HGG; S	22	1 (BraTS); 1 (ISLES'15)	y	-		DSC; Acc
C	2008	G. Agam et al.	Probabilistic brain lesion segmentation in DT-MRI	International Conference on Image Processing		y	T1, T2, DTI	Mixture model, Expectation-Maximization	n/a	n/a	n/a	n	n	S	#VALUE!	21 (+19healthy)	3	n	Manual		PPV; Sen; DSC

References:

van Norden, Anouk GW, et al. "Diffusion tensor imaging and cognition in cerebral small vessel disease: the RUN DMC study." Biochimica et Biophysica Acta (BBA)-Molecular Basis of Disease 1822.3 (2012): 401-407.

Aalten, Pauline, et al. "The Dutch Parelsnoer Institute-Neurodegenerative diseases; methods, design and baseline results." BMC neurology 14.1 (2014): 1-8.

Bakas, Spyridon, et al. "Advancing the cancer genome atlas glioma MRI collections with expert segmentation labels and radiomic features." Scientific data 4 (2017): 170117.

Bowden, Donald W., et al. "Review of the Diabetes Heart Study (DHS) family of studies: a comprehensively examined sample for genetic and epidemiological studies of type 2 diabetes and its complications." The review of diabetic studies: RDS 7.3 (2010): 188.

Reid, Christopher M., et al. "Aspirin for the prevention of cognitive decline in the elderly: rationale and design of a neuro-vascular imaging study (ENVIS-ion)." BMC neurology 12.1 (2012): 1-9.

Chabriat, Hugues, et al. "Predictors of clinical worsening in cerebral autosomal dominant arteriopathy with subcortical infarcts and leukoencephalopathy: prospective cohort study." Stroke 47.1 (2016): 4-11.

Clark, Kenneth, et al. "The Cancer Imaging Archive (TCIA): maintaining and operating a public information repository." Journal of digital imaging 26.6 (2013): 1045-1057.

De Leeuw, F. E., et al. "Prevalence of cerebral white matter lesions in elderly people: a population based magnetic resonance imaging study. The Rotterdam Scan Study." Journal of Neurology, Neurosurgery & Psychiatry 70.1 (2001): 9-14.

Firbank, Michael J., et al. "High resolution imaging of the medial temporal lobe in Alzheimer's disease and dementia with Lewy bodies." Journal of Alzheimer's disease 21.4 (2010): 1129-1140.

Hinton, Ladson, et al. "Recruitment of a community-based cohort for research on diversity and risk of dementia." Alzheimer disease and associated disorders 24.3 (2010): 234.

Hougaard, K. D., et al. "Remote ischemic perconditioning in thrombolysed stroke patients: randomized study of activating endogenous neuroprotection–design and MRI measurements." International Journal of Stroke 8.2 (2013): 141-146.

Hsieh FI, Lien LM, Chen ST, Bai CH, Sun MC, et al. (2010) Get with the Guidelines-Stroke performance indicators: Surveillance of stroke care in the Taiwan Stroke Registry: Get with the Guidelines-Stroke in Taiwan. Circulation 122: 1116–1123.

I-KNOW. "Integrating Information from Molecule to Man: Knowledge Discovery Accelerates Drug Development and Personalized Treatment in Acute Stroke." (2006).

Tremblay-Mercier, Jennifer, et al. "P4-070: A PROGRAM OF PRE-SYMPTOMATIC EVALUATION OF EXPERIMENTAL OR NOVEL TREATMENTS FOR ALZHEIMER'S DISEASE (PREVENT-AD): DESIGN, METHODS, AND PERSPECTIVES." Alzheimer's & Dementia 10 (2014): P808-P808.

Ellis, Kathryn A., et al. "The Australian Imaging, Biomarkers and Lifestyle (AIBL) study of aging: methodology and baseline characteristics of 1112 individuals recruited for a longitudinal study of Alzheimer's disease." International psychogeriatrics 21.4 (2009): 672-687.

Khayati R, Vafadust M, Towhidkhah F, Nabavi SM (2008) Fully automatic segmentation of multiple sclerosis lesions in brain MR FLAIR images using adaptive mixtures method and Markov random field model. Comput Biol Med 38 (3) 379–390.

Lee, J. H., et al. "Identification of pure subcortical vascular dementia using 11C-Pittsburgh compound B." Neurology 77.1 (2011): 18-25.

Prastawa, Marcel, Elizabeth Bullitt, and Guido Gerig. "Simulation of brain tumors in MR images for evaluation of segmentation efficacy." Medical image analysis 13.2 (2009): 297-311.

For peer review only - http://bmjopen.bmj.com/site/about/guidelines.xhtml

Machner, Björn, et al. "Impact of dynamic bottom-up features and top-down control on the visual exploration of moving real-world scenes in hemispatial neglect." Neuropsychologia 50.10 (2012): 2415-2425.

Maier, Oskar, et al. "Extra tree forests for sub-acute ischemic stroke lesion segmentation in MR sequences." Journal of neuroscience methods 240 (2015): 89-100.

Reijmer, Yael D., et al. "Microstructural white matter abnormalities and cognitive functioning in type 2 diabetes: a diffusion tensor imaging study." Diabetes care 36.1 (2013): 137-144.

Ropele, Stefan, et al. "Determinants of iron accumulation in deep grey matter of multiple sclerosis patients." Multiple Sclerosis Journal 20.13 (2014): 1692-1698.

Rothwell P.M., Coull A.J., Giles M.F., Howard S.C., Silver L.E., Bull L.M., Gutnikov S.A., Edwards P., Mant D., Sackley C.M., Farmer A., Sandercock P.A., Dennis M.S., Warlow C.P., Bamford J.M., Anslow P., Oxford Vascular S. Change in stroke incidence, mortality, case-fatality, severity, and risk factors in Oxfordshire, UK from 1981 to 2004 (Oxford Vascular Study) Lancet. 2004;363:1925–1933.

Sachdev, Perminder S., et al. "The contribution of twins to the study of cognitive ageing and dementia: the Older Australian Twins Study." International Review of Psychiatry 25.6 (2013): 738-747.

Sachdev, Perminder S., et al. "The Sydney Memory and Ageing Study (MAS): methodology and baseline medical and neuropsychiatric characteristics of an elderly epidemiological non-demented cohort of Australians aged 70-90 years." International psychogeriatrics 22.8 (2010): 1248.

Selnes, P., et al. (2013). Diffusion tensor imaging surpasses cerebrospinal fluid as predictor of cognitive decline and medial temporal lobe atrophy in subjective cognitive impairment and mild cognitive impairment. Journal of Alzheimer's Disease, 33(3), 723–736.

Walker, William C., et al. "The Chronic Effects of Neurotrauma Consortium (CENC) multi-centre observational study: description of study and characteristics of early participants." Brain injury 30.12 (2016): 1469-1480.

Zamboni G., Wilcock G.K., Douaud G., Drazich E., McCulloch E., Filippini N., Tracey I., Brooks J.C., Smith S.M., Jenkinson M., Mackay C.E. Resting functional connectivity reveals residual functional activity in Alzheimer's disease. Biol. Psychiatry. 2013;74:375–383.

Pantoni, Leonardo, et al. "Impact of age-related cerebral white matter changes on the transition to disability–the LADIS study: rationale, design and methodology." Neuroepidemiology 24.1-2 (2005): 51-62.

Shepherd, James, et al. "The design of a prospective study of pravastatin in the elderly at risk (PROSPER)." The American journal of cardiology 84.10 (1999): 1192-1197.

Weiner, MD, Howard L. "Oral tolerance for the treatment of autoimmune diseases." Annual review of medicine 48.1 (1997): 341-351.

"European project on brain morphometry,", BIOMORPH, EU-BIOMED2 project nr. BMH4-CT96-0845, 1996–1998.

Price, Catherine C., et al. "MRI-leukoaraiosis thresholds and the phenotypic expression of dementia." Neurology 79.8 (2012): 734-740.

Zacharaki, E.I., Wang, S., Chawla, S., Yoo, D.S., Wolf, R., Melhem, E.R., Davatzikos, C.: Classification of brain tumor type and grade using MRI texture and shape in a machine learning scheme. Magnetic Resonance in Medicine 62, 1609–1618 (2009)

Marias, K., Dionysiou, D., Sakkalis, V., Graf, N., Bohle, R.M., Coveney, P.V., Wan, S., Folarin, A., Buechler, P., Reyes, M., Clapworthy, G., Liu, E., Sabczynski, J., Bily, T., Roniotis, A., Tsiknakis, M., Giatili, S., Veith, C., Messe, E., Stenzhorn, H., Kim, Y.j., Zasada, S., Haidar, A.N., Bauer, S., Wang, T., Zhao, Y., Karasek, M., Grewer, R., Franz, A., Stamatakos, G.: Clinically driven design of multi-scale cancer models: the ContraCancrum project paradigm. J. Roy. Soc. Interface Fo- cus 1(3), 450–461 (2011)

Wardak, Zabi, et al. "Pre-treatment factors associated with detecting additional brain metastases at stereotactic radiosurgery." Journal of neuro-oncology 128.2 (2016): 251-257.

Victor, Ronald G., et al. "The Dallas Heart Study: a population-based probability sample for the multidisciplinary study of ethnic differences in cardiovascular health." The American journal of cardiology 93.12 (2004): 1473-1480.

Rutten-Jacobs, Loes CA, et al. "Risk factors and prognosis of young stroke. The FUTURE study: a prospective cohort study. Study rationale and protocol." BMC neurology 11.1 (2011): 109.

Links to access the referenced databases

MSC'15	https://smart-stats-tools.org/lesion-challenge
WMH Segmentation Challenge '17	https://wmh.isi.uu.nl/
MS Lesion Segmentation Challenge 2016	https://portal.fli-iam.irisa.fr/msseg-challenge/overview
NACC	https://www.alz.washington.edu/
IBSR	https://www.nitrc.org/projects/ibsr
SPL	https://spl.harvard.edu/software-and-data-sets
BrainWeb	http://www.bic.mni.mcgill.ca/brainweb/
Maier et al. 2015	https://figshare.com/articles/dataset/Sub_acute_Ischemic_Stroke_MRI_scans_T1_T2_DWI_ADC_ground_truth_and_segmentation_results/1585018
Hermes	https://clinicaltrials.gov/ct2/show/NCT00875654

For peer review only

Preferred Reporting Items for Systematic reviews and Meta-Analyses extension for Scoping Reviews (PRISMA-ScR) Checklist

SECTION	ITEM	PRISMA-ScR CHECKLIST ITEM	REPORTED ON PAGE #
**TITLE**			
Title	1	Identify the report as a scoping review.	1
**ABSTRACT**			
Structured summary	2	Provide a structured summary that includes (as applicable): background, objectives, eligibility criteria, sources of evidence, charting methods, results, and conclusions that relate to the review questions and objectives.	2
**INTRODUCTION**			
Rationale	3	Describe the rationale for the review in the context of what is already known. Explain why the review questions/objectives lend themselves to a scoping review approach.	3
Objectives	4	Provide an explicit statement of the questions and objectives being addressed with reference to their key elements (e.g., population or participants, concepts, and context) or other relevant key elements used to conceptualize the review questions and/or objectives.	3
**METHODS**			
Protocol and registration	5	Indicate whether a review protocol exists; state if and where it can be accessed (e.g., a Web address); and if available, provide registration information, including the registration number.	3
Eligibility criteria	6	Specify characteristics of the sources of evidence used as eligibility criteria (e.g., years considered, language, and publication status), and provide a rationale.	4-5
Information sources*	7	Describe all information sources in the search (e.g., databases with dates of coverage and contact with authors to identify additional sources), as well as the date the most recent search was executed.	4
Search	8	Present the full electronic search strategy for at least 1 database, including any limits used, such that it could be repeated.	-
Selection of sources of evidence†	9	State the process for selecting sources of evidence (i.e., screening and eligibility) included in the scoping review.	4-5
Data charting process‡	10	Describe the methods of charting data from the included sources of evidence (e.g., calibrated forms or forms that have been tested by the team before their use, and whether data charting was done independently or in duplicate) and any processes for obtaining and confirming data from investigators.	5
Data items	11	List and define all variables for which data were sought and any assumptions and simplifications made.	5
Critical appraisal of individual sources of evidence§	12	If done, provide a rationale for conducting a critical appraisal of included sources of evidence; describe the methods used and how this information was used in any data synthesis (if appropriate).	-
Synthesis of results	13	Describe the methods of handling and summarizing the data that were charted.	5

For peer review only - http://bmjopen.bmj.com/site/about/guidelines.xhtml

SECTION	ITEM	PRISMA-ScR CHECKLIST ITEM	REPORTED ON PAGE #
**RESULTS**			
Selection of sources of evidence	14	Give numbers of sources of evidence screened, assessed for eligibility, and included in the review, with reasons for exclusions at each stage, ideally using a flow diagram.	6
Characteristics of sources of evidence	15	For each source of evidence, present characteristics for which data were charted and provide the citations.	-
Critical appraisal within sources of evidence	16	If done, present data on critical appraisal of included sources of evidence (see item 12).	-
Results of individual sources of evidence	17	For each included source of evidence, present the relevant data that were charted that relate to the review questions and objectives.	-
Synthesis of results	18	Summarize and/or present the charting results as they relate to the review questions and objectives.	5-8
**DISCUSSION**			
Summary of evidence	19	Summarize the main results (including an overview of concepts, themes, and types of evidence available), link to the review questions and objectives, and consider the relevance to key groups.	8
Limitations	20	Discuss the limitations of the scoping review process.	11
Conclusions	21	Provide a general interpretation of the results with respect to the review questions and objectives, as well as potential implications and/or next steps.	13
**FUNDING**			
Funding	22	Describe sources of funding for the included sources of evidence, as well as sources of funding for the scoping review. Describe the role of the funders of the scoping review.	-

JBI = Joanna Briggs Institute; PRISMA-ScR = Preferred Reporting Items for Systematic reviews and Meta-Analyses extension for Scoping Reviews.

* Where *sources of evidence* (see second footnote) are compiled from, such as bibliographic databases, social media platforms, and Web sites.

† A more inclusive/heterogeneous term used to account for the different types of evidence or data sources (e.g., quantitative and/or qualitative research, expert opinion, and policy documents) that may be eligible in a scoping review as opposed to only studies. This is not to be confused with *information sources* (see first footnote).

‡ The frameworks by Arksey and O'Malley (6) and Levac and colleagues (7) and the JBI guidance (4, 5) refer to the process of data extraction in a scoping review as data charting.

§ The process of systematically examining research evidence to assess its validity, results, and relevance before using it to inform a decision. This term is used for items 12 and 19 instead of "risk of bias" (which is more applicable to systematic reviews of interventions) to include and acknowledge the various sources of evidence that may be used in a scoping review (e.g., quantitative and/or qualitative research, expert opinion, and policy document).

From: Tricco AC, Lillie E, Zarin W, O'Brien KK, Colquhoun H, Levac D, et al. PRISMA Extension for Scoping Reviews (PRISMAScR): Checklist and Explanation. Ann Intern Med. 2018;169:467–473. doi: 10.7326/M18-0850.

For peer review only - http://bmjopen.bmj.com/site/about/guidelines.xhtml

BMJ Open

Automatic brain lesion segmentation on standard magnetic resonance images: a scoping review

Journal:	*BMJ Open*
Manuscript ID	bmjopen-2020-042660.R2
Article Type:	Original research
Date Submitted by the Author:	09-Jan-2021
Complete List of Authors:	Gryska, Emilia; Goteborgs universitet Institutionen for kliniska vetenskaper, Radiation Physics<br>Schneiderman, Justin; Goteborgs universitet Institutionen for neurovetenskap och fysiologi, Sektionen för klinisk neurovetenskap<br>Björkman-Burtscher, Isabella; Göteborgs universitet Institutionen för kliniska vetenskaper, Radiology<br>Heckemann, Rolf; Goteborgs universitet Institutionen for kliniska vetenskaper, Radiation Physics
**Primary Subject Heading**:	Radiology and imaging
Secondary Subject Heading:	Research methods
Keywords:	RADIOLOGY & IMAGING, Magnetic resonance imaging < RADIOLOGY & IMAGING, Diagnostic radiology < RADIOLOGY & IMAGING, Neuroradiology < RADIOLOGY & IMAGING

SCHOLARONE™
Manuscripts

I, the Submitting Author has the right to grant and does grant on behalf of all authors of the Work (as defined in the below author licence), an exclusive licence and/or a non-exclusive licence for contributions from authors who are: i) UK Crown employees; ii) where BMJ has agreed a CC-BY licence shall apply, and/or iii) in accordance with the terms applicable for US Federal Government officers or employees acting as part of their official duties; on a worldwide, perpetual, irrevocable, royalty-free basis to BMJ Publishing Group Ltd ("BMJ") its licensees and where the relevant Journal is co-owned by BMJ to the co-owners of the Journal, to publish the Work in this journal and any other BMJ products and to exploit all rights, as set out in our licence.

The Submitting Author accepts and understands that any supply made under these terms is made by BMJ to the Submitting Author unless you are acting as an employee on behalf of your employer or a postgraduate student of an affiliated institution which is paying any applicable article publishing charge ("APC") for Open Access articles. Where the Submitting Author wishes to make the Work available on an Open Access basis (and intends to pay the relevant APC), the terms of reuse of such Open Access shall be governed by a Creative Commons licence – details of these licences and which Creative Commons licence will apply to this Work are set out in our licence referred to above.

Other than as permitted in any relevant BMJ Author's Self Archiving Policies, I confirm this Work has not been accepted for publication elsewhere, is not being considered for publication elsewhere and does not duplicate material already published. I confirm all authors consent to publication of this Work and authorise the granting of this licence.

For peer review only

Automatic brain lesion segmentation on standard magnetic resonance images: a scoping review

Emilia Gryska[1,2*], Justin F. Schneiderman[1,3], Isabella Björkman-Burtscher[4], Rolf A. Heckemann[1,2]

1 MedTech West at Sahlgrenska University Hospital, University of Gothenburg, Sweden

2 Department of Radiation Physics, Institute of Clinical Sciences, University of Gothenburg, Sweden

3 Department of Clinical Neurophysiology, Institute of Neuroscience and Physiology, Sahlgrenska Academy, University of Gothenburg, Sweden

4 Department of Radiology, Institute of Clinical Sciences, Sahlgrenska Academy, University of Gothenburg and Sahlgrenska University Hospital, Gothenburg, Sweden

* Corresponding author: Emilia Gryska

Postal address: MedTech West, Röda stråket 10B, Sahlgrenska University Hospital, 413 45 Göteborg

E-mail address: emilia.gryska@medtechwest.se

Telephone number: +46 720304106

Keywords: Algorithms, Research design, Reproducibility of Results, Image Processing, Computer-Assisted

Word count: 6 302

Author contributions: EG conducted the study and led writing of the article. RAH was the main supervisor and consultant of the study progress and design choices. JFS provided input on the study plan and methodology at all stages. IBB provided guidance on the interpretation of the findings from a clinical perspective. All co-authors collaborated on manuscript composition and editing.

Funding: JFS is supported by the Swedish Childhood Cancer Foundation (MT2018-0020) and the ATTRACT project funded by the EC under Grant agreement 777222.

Competing interests: None declared

Supplementary data: The data extracted for the study can be found in FigShare repository, DOI: 10.6084/m9.figshare.12639842

For peer review only

ABSTRACT

Objectives Medical image analysis practices face challenges that can potentially be addressed with algorithm-based segmentation tools. In this study, we map the field of automatic MR brain lesion segmentation to understand the clinical applicability of prevalent methods and study designs, as well as challenges and limitations in the field.

Design Scoping review.

Setting Three databases (PubMed, IEEE Xplore, and Scopus) were searched with tailored queries. Studies were included based on predefined criteria. Emerging themes during consecutive title, abstract, methods, and whole-text screening were identified. The full-text analysis focused on materials, preprocessing, performance evaluation and comparison.

Results Out of 2990 unique articles identified through the search, 441 articles met the eligibility criteria, with an estimated growth rate of 10% per year. We present a general overview and trends in the field with regard to publication sources, segmentation principles used, and types of lesions. Algorithms are predominantly evaluated by measuring the agreement of segmentation results with a trusted reference. Few articles describe measures of clinical validity.

Conclusions The observed reporting practices leave room for improvement with a view to studying replication, method comparison, and clinical applicability. To promote this improvement, we propose a list of recommendations for future studies in the field.

ARTICLE SUMMARY

Strengths and limitations of this study

- This is the first overarching review of MR-based automatic brain lesion segmentation methods without restriction to a particular lesion type.
- The study was conducted following a previously published protocol.
- We consulted practitioners to ensure relevance of our work for clinical practice.
- The rigorous study design restricted our ability to expand on emerging themes, but did not entirely prevent epistemic drift.
- The restriction to a one-pass full-text analysis entailed a superficial level of interpretation – in hindsight a deeper reading would have been desirable.

INTRODUCTION

Magnetic resonance imaging (MRI) has become an integral part of diagnostics to detect, differentiate, and characterize brain lesions. Properties that determine this success are safety, high tissue contrast, and sensitivity to abnormality. Conditions that can lead to brain lesions include traumatic brain injury, vascular disease (including ischemic and hemorrhagic stroke), neoplasms, autoimmune disorders, infection, degenerative diseases, congenital conditions, and systemic diseases with secondary effects on the central nervous system. In some of these conditions, accurate estimation of lesion size and its progression may be essential to treatment planning, disease monitoring, and/or treatment evaluation.

Visual image interpretation is still the most common and accepted way to analyse clinical images. In some cases, the visual examination must be extended to include lesion delineation. For example, the boundaries of a tumour and structures at risk need to be localized accurately for radiation therapy planning. Such delineation depends on a skilled rater, is time-consuming, and is characterized by high variability between raters and high variability between repeated delineations on the same images by the same rater (i.e., low objectivity and reliability, respectively). In other clinical examinations, surrogate metrics, which can be based on the largest perpendicular diameters [1,2] or

For peer review only

visual scoring [3], are used for estimating the size of a lesion rapidly. Such visual or area-based lesion volume estimates may not be accurate enough [4].

Developments in magnetic resonance (MR) image acquisition procedures resulted in high-quality and complex images that capture a variety of structural and functional phenomena. On the one hand, the images carry more information about the lesions, thereby enabling more accurate diagnosis. On the other hand, each image requires more analysis time. This reduces the rate at which radiologists can analyse images. The increasing accessibility to MR scanners furthermore results in more images being acquired, further straining image processing capacity and potentially diminishing the quality of interpretation [5,6].

To summarize, three major issues with current clinical practices for image analysis are: subjective and time-consuming manual segmentation, limited accuracy of the surrogate volume estimates, and increasing information content and number of images to process.

Automatic lesion segmentation algorithms promise to alleviate these issues through fast and consistent lesion delineation that scales with demand. Quantitative image analysis using automatic lesion segmentation further has the potential to increase diagnostic and prognostic accuracy of lesion examination by providing radiologists with prompt and explicit information about a lesion.

Several reviews on automated brain lesion segmentation methods have previously been published. Most of these outline and analyse commonly used segmentation algorithms or general segmentation principles [7–16]. The perspective of the clinical applicability of automated segmentation algorithms has been explored by Garcia-Lorenzo et al. [10] and Bauer et al. [7]. In both reviews, the authors recommend improvements concerning study designs and formulation of research questions. The authors stress the importance of validating the robustness of the segmentation algorithms under variation stemming from three causes: differences between scanners and acquisition protocols, natural variability in normal anatomy and lesion appearance, and artifacts. Moreover, Bauer et al. [7] expressed the need for better communication between researchers developing segmentation methods and clinical radiologists who are the intended users of these methods.

A comprehensive review of automatic segmentation methods without restriction to lesion type is still lacking today, perhaps because of the challenge associated with the large and growing literature. We therefore present a scoping review of such methods and, given the rapidly growing extent of the literature, provide a timely account of the field that may not be feasible in the future. To ensure rigour, reproducibility, and comprehensiveness, we adopted scoping review methods proposed earlier [17–21].

Furthermore, there is a disconnection between developers and users of automatic brain lesion segmentation methods [7]. We therefore aim to examine clinical relevance of the research conducted in the field and seek to understand how the most prevalent methods (segmentation algorithms) and study designs (how they are validated) reflect the clinical applicability of methods described in the reviewed articles. Moreover, we aim to identify issues, limitations, and grand challenges of the field, and suggest actions to bridge the gap between research and clinical practice.

METHODS AND DESIGN

This study has been conducted according to a previously published protocol [22] and based on scoping review methods proposed by Arksey and O'Malley [17] and further developed by Levac et al. and Colquhoun et al. [18,19]. We also incorporate relevant parts of the PRISMA [20] and PRISMA-ScR [21] protocol for this type of review. Here we provide a summary of the protocol as well as an account of changes made, along with their respective rationale.

Stage 1: identifying research questions

The following research questions (RQs) were posed in the protocol [22]:

1 Which common image processing steps are necessary for automatic brain lesion segmentation on MR images?

2 Which mathematical and computational theories are most commonly applied in which types of brain lesions?

For peer review only - http://bmjopen.bmj.com/site/about/guidelines.xhtml

3 What is the efficacy of existing implementations?

4 What are the limitations of those methods and issues that should be addressed in future studies to develop a tool that is suitable for clinical use?

5 What are the most commonly used MR data sets that provide reference lesion segmentation and/or diagnostic classification?

While getting familiar with the abstracts of the sample we questioned the relevance of RQ 1 and 2. We recognised the need for consultation at this early stage of the project as opposed to consultation on the findings as originally proposed by Arksey and O'Malley [17]. The RQs do not reflect fully the potential of our study to address issues critical to advancing the field and bringing a benefit to the community. The consultation was conducted as semi-structured interviews with five clinicians who have experience in brain image analysis. We interviewed two neurosurgeons, an oncologist, a radiation oncologist, and a neuroradiologist. The interviews were structured to elicit understanding of their daily workflow, of how they use and analyse images, and of their opinions on automating the process of brain lesion segmentation.The initial screening of the sample abstracts and, in parallel, the consultations directed our analysis towards examining the clinical relevance of the prevalent methods and study designs.

Stage 2: identifying relevant articles

For the purpose of this study, we define an article as an individual published item that was found according to the presented search strategy and that meets the inclusion criteria of this review.

Eligibility criteria were unchanged from those defined in the protocol: articles evaluating an automatic brain lesion segmentation method applied to MR images acquired for human brain lesion inspection, and articles evaluating the performance of the method by comparison with a reference segmentation were suitable for the study.

The initial search strategy remained mostly unchanged. Three databases (PubMed, IEEE Xplore, and Scopus) were queried. Initially, a broad search phrase on automatic brain lesion segmentation methods on MRI was used to generate a first sample for each database. Controlled vocabulary tags were extracted from the articles and arranged in descending order of frequency. From this list, tags were selected to refine and customize the search phrase for each database. These refined and customized search phrases were used to retrieve the sample for our study. The search was conducted on 4th of November 2018.

The relevant articles were identified through hierarchical screening at four levels with given aims and objectives:

1) Title level: exclusion of clearly ineligible articles.

2) Abstract level: refinement of inclusion and exclusion criteria based on literature content; identification of 100 eligible articles by reading of randomly selected abstracts out of a sample of 1359; identification of themes to guide the study selection process (Stage 3);

3) Methods level: identification of two core concepts: algorithm validation and applicability of an automatic segmentation method for clinical parameter prediction; exclusion of studies using manual or semi-automatic segmentation, not stating the source of the tested images/patients, only using synthetic images, conducing the algorithm validation on fewer than 10 unique scans/images, or not providing results of the algorithm's performance.

4) full paper screening level (see Stage 3).

The same hierarchical screening procedure was applied to references of the eligible sample also screening for duplicates and excluding conference proceedings that were later published in journal articles if the patient cohort was similar in both articles. Screening was discontinued upon finding an exclusion criterion.

Stage 3: study selection

In the full-text screening, we applied the above criteria again and excluded papers that:

- did not perform segmentation or did not provide segmentation evaluation outcome measures (wrong outcome)

- proposed methods that require user interaction
- did not provide information about the gold standard (GS) or a reference measurement
- did not provide information about the origin of the images or used only synthetic images
- did not provide the number of images used for evaluation
- evaluated the method on fewer than 10 unique scans.

Stage 4: data charting

The data extracted were charted in a spreadsheet (supplementary material) with five categories of variables:

1) bibliographic category (first authors' name, article title, journal or conference name, and year of publication),

2) segmentation category (preprocessing procedures, algorithms and computational theories, if lesion classification used, required or allowed input modalities, processing time of the method, and availability of the software),

3) validation category (process of obtaining reference segmentations, number of raters or observers, and evaluation metrics),

4) study cohort category comprised patients' diagnoses, lesion type, sources, and number of images),

5) general comments category (information that did not fit in other categories, but was considered relevant) Stage 5: collating, summarizing, and reporting the data

Stage 5: collating, summarizing, and reporting the data

The collected data is disseminated through numerical analysis of bibliographic information overview, trends in the field, and study design characteristics. We provide the account of both prevalent study design choices and less common ones that are relevant to the clinical applicability assessment. Study design features are grouped into materials, preprocessing, performance evaluation, and performance comparison.

Patient and Public Involvement

No patients nor members of the public were involved in this research project.

RESULTS

The results of the scoping review include answers to research questions 2, 4, and 5. The answers to questions 1 and 5 are directly presented in the results. The answer to question 4 is formulated in the discussion based on the presented findings. In the discussion, we also justify why questions 1 ans 3 could not be explicitly answered.

Overview

The number of papers included at each stage of the review is shown in Figure 1. Among 2500 articles returned by the searches and 490 titles identified through reference screening, we included 441 articles in the review. Among the eligible papers, 255/441 were published in journals [23–277] and 184/441 in conference proceedings or conference workshops [278–460], and 2/441 articles were uploaded as preprints [461,462]. The following journals or conferences occurred most frequently as the publication source: International Conference on Medical Image Computing and Computer-Assisted Intervention (66 articles), NeuroImage (20 articles), IEEE Transactions on Medical Imaging (20 articles), SPIE Medical Imaging Conference (18 articles), NeuroImage: Clinical (15 articles), IEEE International Symposium on Biomedical Imaging (13 articles), and PloS One (12 articles).

Figure 2 shows the distribution of articles that propose a method to segment a particular brain lesion type. The dominance of methods segmenting brain tumours is evident from the data.

For peer review only

Trends

Substantial growth of the field was evident from the number of articles published annually (Figure 3). Three distinct periods with an increase in the number of published articles compared to preceding years were noticeable. During these years, an increase is also evident in the number of articles addressing segmentation of lesion types corresponding to the challenges taking place. The first wave corresponds to the *3D Segmentation in the Clinic: A Grand Challenge II: MS Lesion Segmentation* [463]. The second wave started in 2012 coinciding with the advent of *The Multimodal Brain Tumor Image Segmentation Benchmark (BraTS)* [464]. The third wave started in 2015 when three brain lesion segmentation challenges took place: BraTS, the *Longitudinal MS Lesion Segmentation Challenge* [465], and the *Ischemic Stroke Lesion Segmentation challenge (ISLES)* [466].

Image sources and databases

The distribution of the image sources is presented in Table 1. Most commonly, the image data were collected from non-public sources (254 articles). Publicly available data with reference segmentations were used in 217 articles. The most popular database was *BraTS* [464] (157 articles) followed by the *3D Segmentation in the Clinic: A Grand Challenge II: MS Lesion Segmentation* [463] and *ISLES* (17 articles) [466]. 10 articles rely on publicly available sources without reference segmentations. 41 articles validate the method on both publicly available datasets with reference segmentations and non-public data.

Table 1: Distribution of image sources used for algorithm validation.

Non-public data sources	Publicly available data with reference segmentations				Publicly available data without reference segmentations
	BraTS [464]	Grand Challenge II: MS Lesion Segmentation [463]	ISLES [466]	Other	
254	157	31	17	12	10

The distribution of the cohort sizes used to validate the proposed methods (Figure 4) shows that more than half of the studies described in included articles test methods on 50 or fewer individuals' brain images (260/441). Among the eligible articles, 280/441 describe a method validated on images obtained from more than one scanner.

Sequences

The majority of the proposed methods operated on multi-sequence scans as inputs (307/441) while 6 accepted both single- and multi-sequence input. 79 methods were built to evaluate single-sequence input and 22 did not specify the input modality. Articles that compared or evaluated a previously proposed method were not included in the analysis (27/441).

Image processing steps, algorithms and computational theories

Preprocessing procedures

The most commonly applied preprocessing procedures and tools (disregarding publicly available databases for this purpose) are shown in Table 2. Although authors commonly document individual steps and corresponding algorithms, information on whether these algorithms are integrated in the segmentation tool is rarely available [90,195]. Fifteen articles mention visual supervision [93,114,133], semiautomatic preprocessing [186,226,267,328,405], manual correction [155,171,209,273] , error or failure of preprocessing (usually at the brain extraction step) [135,136,160].

Five methods proposed in the reviewed articles use minimal [268,400] or no preprocessing [163,164,220], and in each case this is presented as an advantage of the proposed algorithm.

Table 2: Prevalent image preprocessing steps in 374/414 articles (not specified in 67 articles).

For peer review only

Procedure	Common tool/approach	N (of 374)
Intensity normalization	Histogram matching, intensity scaling	224
Bias field (field inhomogeneity) correction	N3, N4 [467,468]	192
Brain extraction	FSL BET [469]	190
Image (co-)registration	rigid/affine; SPM, FSL [470,471]	179
Denoising	anisotropic diffusion filtering	60
None or minimal		5

Algorithms and computational theories

Among the most commonly used algorithms or models in the segmentation methods were machine learning (decision trees, mixture models, fuzzy clustering, support vector machines, expectation maximization) and in particular deep-learning algorithms (artificial neural networks) (Figure 5). The analysis accounted for the same methods published in separate articles that deal with either different lesion types or similar lesion type with different image sources.

Performance evaluation

Comparison with the reference by overlap measures was by far the most commonly used criterion to evaluate the accuracy of the automatic segmentation. Among articles that specify how the reference segmentations had been generated (265) in publicly non-available data sets, most specified manual segmentation by one rater (85) or two raters (61). More than two raters contributed in 38 articles, and 33 articles used semiautomatic procedures for constructing the reference. When multiple raters delineated the region of interest, the reference segmentation was obtained through consensus of the raters or majority voting. Forty-five articles have also included inter- and intra-rater variability evaluation on data sets used in their studies. The most frequently applied measures were the Dice coefficient (330), sensitivity, also expressed as true positive ratio TPR, or overlap fraction (227), positive predictive value (102), Jaccard coefficient (58), and specificity (82). Volumetric measures, such as volume difference, error or correlation between the automatic and reference segmentation were used in 133 articles, and distance-based measures (such as Hausdorff distance, mean absolute distance, surface distance) in 41.

In addition to segmentation accuracy, reliability of a method was estimated in some of the articles. Reliability was directly evaluated through a test-retest procedure, where participants were scanned twice at a short interval, with repositioning between the scans in five articles [127,177,196,321,347]. In other articles, indirect approaches were described where consistency of longitudinal data with clinical findings of stable or progressive disease was used as a proxy for reliability [38,270,454].

Performance comparison

Maldjian et al. (2013) [133], Lesjak et al. (2016) [150], and de Sitter et al. (2017) [224] examined previously proposed methods and compared the results they obtained in their studies to the performance reported in the original papers. All three articles reported poorer independent testing results. The authors pointed out a lower lesion load of the studied population than in the original papers that accounted for reduced scores in the replication attempts. In their paper, de Sitter et al. (2017) [224] strongly called for improvements to automatic lesion segmentation before their introduction to routine clinical use.

For peer review only - http://bmjopen.bmj.com/site/about/guidelines.xhtml

Of 441 articles, 233 included information regarding both the processing time (from less than 1 second to 7 hours) and computational system requirements used for segmentation. In 56 articles we found information about only one of the two parameters, however such information is incomplete and cannot be used to estimate how well a method will perform on different hardware.

Only 24 of the included articles reported on methods that were made publicly available to download [43,50,53,67,76,84,90–92,126,134,138,144,149,154,170,173,178,190,195,196,199,228,230,426]. One was available upon request [111]. Two articles promised future availability of the methods proposed [41,193] but they were not available as of 4th of May 2020.

Reporting recommendations

Based on our findings, we developed a checklist with reporting recommendations (Table 3).

DISCUSSION

In this paper, we present a scoping study of automatic brain lesion segmentation on magnetic resonance images based on rigorous literature review methodology [17]. This is the first review that takes all methods into account, independent of specialization towards lesions of a particular etiology. The key findings from our analysis of 441 articles are a) a rapid increase of interest in the field, b) a plethora of proposed methods contrasted with a dearth of open documentation and available software, and c) high prevalence of problematic reporting practices that restrict the ability of independent researchers to replicate reported results and conduct method comparisons.

Variable design of automatic segmentation methods

RQ1: Which common image processing steps are necessary for automatic brain lesion segmentation on MR images?

Image preprocessing emerged as a pervasive step in the processing chain for lesion segmentation. From the collected data we identified procedures that are commonly agreed to constitute preprocessing: skull stripping, image co-registration, bias field correction, and intensity normalization. Some algorithms rely on additional preprocessing, such as tissue classification. The procedures vary considerably between methods, and a lack of a universal distinction between segmentation steps and preprocessing steps became apparent during the analysis.

For a clinically suitable method, a distinction between preprocessing and segmentation steps may not be necessary since the final segmentation will rely on a whole processing chain (including the preprocessing) applied to a raw image. Validation of the whole chain, and assessing the impact of each step on the outcome, however, are desirable in method evaluation studies. Unfortunately, authors rarely state whether preprocessing is integrated with the segmentation algorithm and to what extent it relies on user input. Without such information, namely a list of all the steps that are performed on a raw image, the study cannot be replicated. This implies that the findings are of no help in assessing the potential clinical validity of the proposed method implementation.

Another implementation issue arises from the necessity of visual supervision or manual corrections of the preprocessing that was indicated in 15 articles. Any requirement of user interaction at any point in the processing chain entails disadvantages: it impedes implementation by increasing the complexity of the technical integration, and, once the tool is implemented, permanently burdens staff with an additional task. Any potential benefit of user interaction (e.g. increased robustness) has to be weighed carefully against these costs.

RQ2: Which mathematical and computational theories are most commonly applied in which types of brain lesions?

The most prevalent methods utilized were artificial neural networks. The finding is not surprising given the popularity and remarkable performance of deep learning algorithms particularly in image processing applications. In the absence of widely used and agreed-upon criteria for performance evaluation, we have abstained from attempting to rank methods or make specific recommendations.

Validation process and efficacy of automatic segmentation methods

RQ2: *What is the efficacy of existing implementations?*

We found that in view of current practices, the question cannot be answered: authors present assessments that are predicated on their own needs and biases, and there is no established standard that enables fair method comparison. Public challenges have been set up in an effort to address this problem, but they have only been partially successful, as illustrated in the following section. Three obstacles to fair comparative method assessments are paramount: the principal lack of ground truth in *in vivo* imaging, the enormous parameter space of acquisition settings that leads to variable feature presentations, and the lack of objectivity (as shown by inter-rater and intra-rater variability [472,473]) of reference segmentations. More effort should thus be devoted to increasing the informativeness of the validation step. This can be achieved by painstakingly reporting the number of annotators, their experience, how the delineations were fused, and acquisition details that could conceivably have an impact. According to Gibson et al. [474], such information can be used to calculate the statistical power of segmentation studies with respect to the number of reference images.

To answer the question we posed regarding the efficacy, we recorded the values of the comparison measures from each article. The information, however, cannot be meaningfully synthesised or compared and was not presented in the results section for this reason. Our investigation did not result in a direct answer to the question, but revealed a paucity of standard comparison procedures.

Inability to answer the posed question points to further issues on which we reflect from the perspective of clinical suitability of the proposed methods. The medical image analysis procedures commonly used in clinical diagnostics today are strongly operator-dependent. They also scale poorly to the growing workload that results from the increasing number of modalities [475], the increasing accessibility of imaging scanners [476], and the increasing amount of image information due to technical advances that achieve enhanced spatial and contrast resolution [477]. Automatic image analysis methods in general, and among them automatic lesion segmentation methods promise to alleviate some of this pressure. Other requisite characteristics that need to be assessed to evaluate the clinical applicability of a tool are reliability, robustness, and generalizability of its findings.

Reliability of an automatic segmentation algorithm seems to be assumed since the decision whether a given voxel is a lesion or not is made based on well-defined rules. However, the characteristic is rarely tested in method validation studies. Only five articles in our sample included a test-retest evaluation with patient repositioning between the scans. Such testing yields crucial reliability data against which longitudinal variations of lesion measures need to be compared to distinguish actual lesion change from other sources of variability.

Generalizability of a method is tested by processing images acquired from different scanners on a sufficient number of images from populations that are at least as heterogeneous as the population expected to be examined clinically. While more than half of the articles (280/441) used images coming from more than one scanner, validation on fewer than 50 images were equally prevalent (260/441). Such small sample sizes are insufficient for clinical validation, even if the images originate from multiple scanners. Using images acquired from multiple sources and validating a method on a large number of images reduces a method’s bias towards the data set or sets on which a given method was originally developed.

To us, one of the most surprising findings was how few methods are publicly available. Authors who share their software enable independent testing in unanticipated conditions and with various cohorts. Finally, to evaluate clinical relevance of the results of automatic segmentation, a method should be tested in genuine clinical scenarios for a well-defined task, such as monitoring disease progression, treatment response evaluation, or radiotherapy planning. Each of these tasks may require a different level of error margin and validation standard and the relevance of the results must be evaluated by clinicians. Various metrics may be used to either optimize the method or evaluate its accuracy for a particular clinical question, lesion type, and size.

Many papers claim usefulness of their algorithm for particular clinical tasks, but do not test the acceptability of the results for the intended purpose.

Limitations and issues of the proposed methods, and grand challenges of the field

RQ4 a) What are the limitations of those methods (...)?

Due to the inherent reference, image, and lesion variability, a meaningful comparison of algorithms evaluated independently on separate data sets to establish which method performs better is challenging. One solution to the problem has been proposed in the form of segmentation challenges. The challenges have become a quasi-standard for comparing brain lesion segmentation algorithms. The setup, however, comes with certain shortcomings with respect to evaluating clinical applicability of the evaluated algorithms. As mentioned, evaluating the whole chain of processing steps is especially important for validation of a clinical tool. In a challenge setup, the algorithms are tested on partially preprocessed images. Even when the individual steps are described in detail, the impact of the preprocessing on the segmentation algorithms outcome is unknown. Validation of the processing chain becomes questionable if a step is changed, either based on the users' subjective judgement or due to implementation of a different procedure for a particular step. According to *ISLES* [466] and several articles in our sample, the skull-stripping step may need to be supervised and manually corrected. The choice of interpolation method is yet another issue that can influence the outcome of an automatic segmentation algorithm, especially in tasks requiring high accuracy.

Another aspect of the challenges is the prestige and publicity for the authors and their method after proposing a winning segmentation algorithm. The final rank is calculated by the organizers who evaluate the methods on hidden data sets. Organizers effectively take the role of independent arbiters. Participation in challenges and post-contest use of the challenge data as reference material indicate that organizers are generally trusted in this role. Maier-Hein et al. suggest, however, that the results of such competitions should be considered carefully [478]. The authors point to several issues that have a significant impact on the final ranking of evaluated methods. The first problem they report is lack of thorough reporting of relevant information that is essential for result interpretation. Moreover, it turns out that changes in metrics and aggregation methods for the scores of individual test cases alter the ranking of evaluated methods. A similar effect on the ranking was observed when the reference segmentations were exchanged against those of another rater. The final rank in such a competition depends also on the test data and on how missing data is handled.

These concerns cast doubt on the validity of testing clinical applicability of automatic brain lesion segmentation methods in a challenge setup. Even if the challenge databases contain images from multiple scanners, it cannot be regarded as generalizable if it has not been tested on raw images (reconstructed, but not otherwise processed) as produced by the scanning equipment. In our sample, only 10% of the articles explicitly report on such evaluation. While obtaining independent data sets is expensive, it is a crucial step on the way of creating a clinically applicable automatic lesion segmentation tool.

Ultimately, it is the target users who need to decide whether a given validation standard and resultant performance confidence are sufficient to apply a tool to answer a given clinical question. With guidance from support system developers, as well as transparent and thorough reporting of the processing steps and validation procedures, the users may consider a given tool trustworthy. Trustworthiness has been reported as a crucial factor in developing a clinically usable support tool [479]. The present scoping review does not show what properties and features clinicians need to develop trust in a tool. Still, this is an important question that we will seek to address in future work.

We conducted an exploratory study to begin to address the issue [480]. The aim was to learn how considering radiologists' competent input can improve the design and validation procedures of automatic brain lesion segmentation methods with a view to increasing trust and trustworthiness. Our findings corroborate previous findings [479]. We found that two crucial characteristics that influence clinicians' trust in a tool are the provision of an error margin with any quantitative measure, and consideration of the varying need for accuracy, depending on the diagnostic task.

Relevance for research and clinical practice

Noteworthy trends also emerged from the data regarding the prevalence of methods developed for segmentation of a particular lesion type, as well as the advent of segmentation challenges focussing on corresponding lesion types. We observed that the majority of the methods described in our sample have been developed for the purpose of brain tumour segmentation. We also note consistent growth of the number of images in the database underlying the *BraTS* challenge, which has been organised annually since 2012. Stroke lesion segmentation methods have not proliferated to the same extent, despite similar competitions having been organized (yearly *ISLES* challenges from 2015 to

2018). A distinct increase in the number of publications proposing white-matter lesion segmentation algorithms was observed when segmentation challenges of this type of lesion were organized.

Some compelling questions follow this observation, e.g. whether some tasks are more difficult to solve algorithmically, or whether these trends reflect on clinical usefulness of developing segmentation algorithms for a particular lesion type. Even though the questions cannot be directly answered by our findings, they point to important issues that need to be explored to better understand the relevance of different lesion segmentation algorithms and implications of the trends for clinical practice.

Another finding that potentially contributes to the gap between research and clinical practice is the widespread use of deep-learning algorithms for the lesion segmentation task in the research setting. The practical efficiency of the algorithms depends on the availability of graphical processing units (GPUs). Implementing a deep-learning based algorithm in a clinical setting requires dedicating resources for purchasing suitable hardware and integrating it with radiological workflow, information, and storage systems.

A key factor in developing a clinical tool is obtaining regulatory approval for diagnostic use. None of the articles in our sample mentioned this requirement, possibly because authors do not consider it relevant at the stage of development when publication occurs.

It appears that despite claims of clinical relevance made by many authors, development of brain lesion segmentation methods happens predominantly in an academic space, where technological challenges matter most and implementation hurdles are not explicitly considered. To increase the relevance and societal benefit of method development in the field, it will be necessary for developers to widen their perspective to include the critical path towards clinical implementation, on which users' demands and regulatory requirements have to be met.

Challenges and limitations of the study

The biggest challenges we had to address in this study were designing data charting categories, extracting the relevant information regarding both inclusion and exclusion criteria, and the actual charting of the data. Arksey and O'Malley [17] recommend deciding the inclusion criteria after becoming familiar with the identified relevant studies. For us, this was impossible due to the large size of the raw sample of original search results, along with the variability of both the type and the level of detail of reported information.

Our inclusion and exclusion criteria were defined based on a pilot analysis of articles selected randomly from the raw sample. These criteria were then applied to the full raw sample in hierarchical fashion. A major impediment at this stage was the inconsistent way the information is organized and presented in the articles. In the case of conference papers, the authors typically have to adhere to page limits, so certain compromises are inevitable. When it comes to journal publications, the methodology of a study should be thoroughly reported for the sake of reproducibility, but also to enable data extraction for systematic reviews.

Another challenge we faced and a limitation of the study comes from the mentioned incoherent way of reporting studies and a challenge to derive strict definitions of the inclusion and exclusion criteria. Often, the criteria we sought in the articles are not presented clearly, or the information is scarce. Keeping in mind the broad nature of a scoping review and the aim of mapping the field, the author conducting the scoping study (EG) chose to err on the side of inclusion if the information in an article did not allow a decisive application of the criteria. While this strategy, combined with a single-rater approach, introduces a certain amount of selection bias, this bias was at least consistent between articles. We believe the approach provides a reasonable tradeoff between transparency and reproducibility of the study, and fulfilling the objectives of conducting a scoping review.

Other sources of bias were excluded by design. In particular, we conducted the consultation interviews after the literature sampling step, eliminating potential selection bias arising from the interview results. While the strong dominance of articles treating tumour segmentation (cf. Figure 2) may seem surprising, we believe that it is an accurate reflection of the research community's interest.

This review does not consider modalities such as projection radiography, computed tomography, nuclear imaging, or ultrasonography. It focuses instead on MR, which is used to address a larger variety of brain lesions, provides more detailed information, and stands out among radiological techniques regarding the number of related publications.

We acknowledge self-critically that, due to the extensive nature of the task, the time taken, and the lessons learned during the work, some epistemic drift occurred, taking our focus away from the first two research questions and towards the question of clinical applicability that appears to be underserved by the current literature. Thanks to our early decision to hold ourselves to account by writing a detailed protocol [22], this drift stayed within reasonable boundaries and is well-documented, as we accounted for protocol modifications in the present work.

Recommendations for future work

RQ4 b) What issues should be addressed in future studies to develop a tool that is suitable for clinical use?

To address the shortcomings revealed by our analysis, we propose a set of recommendations for future studies as well as avenues researchers might follow that promise, in our estimation, to advance the field in the direction of enabling clinical decision support.

The most important recommendation arises from the variability encountered in many aspects of the field. We propose a checklist (Table 3) of items that should be reported in investigations of automatic brain lesion segmentation methods. We split the items into three levels of validation that we see as a potential path towards developing a clinical tool. Technical validation studies focus on developing the algorithm and evaluating its performance according to common criteria in the field. The pre-clinical validation focuses on the performance of an algorithm in a setting resembling clinical environment and a realistic use case. The final level, clinical validation, requires the tool to be compatible with hospital and radiology information systems and to be ready for the process of obtaining regulatory approval for clinical use if such approval has not been yet obtained. This checklist, especially the technical validation level, will facilitate replication as well as comparisons between methods and studies, both informal and in meta-analyses. If authors were to follow the checklist in future studies, this would be a step towards standardization of reporting in the interest of advancing knowledge and promoting implementation as clinical tools.

Table 3: Proposed reporting items for automatic brain lesion segmentation studies.

Technical validation	**Method**	List of processing steps necessary to apply to a raw image
		Computational system parameters
		Computation time
		Open documentation of the algorithm
	**Reference segmentation**	Number of raters
		Raters' training/experience
		Method of segmentation
		Method of consolidation (if multiple raters)
	**Validation**	List of validation metrics used
		Number of images (split into training/validation/testing if applicable)
		Input sequences
		Number of scanners used to acquire images
		Acquisition parameters
		Number of time points and intervals for longitudinal data
	**Results**	Mean, median, standard deviation for each validation metric
		Number of failed cases (if applicable)
Pre-clinical validation	**Patient information**	Diagnosis and level of verification e.g. clinical follow-up, tissue sampling, autopsy, etc.
		Clinical presentation
		Administered treatments (if applicable)

For peer review only - http://bmjopen.bmj.com/site/about/guidelines.xhtml

Clinical validation	**Clinical task**	Explicit definition of the clinical task for which the algorithm is applied (e.g. lesion growth estimation, treatment evaluation, radiotherapy planning).
	**User validation**	List of optimization metrics used for the clinical task (if applicable).<br>User's method of evaluating the outcome.
	**Information and storage system compatibility**	Compatibility with picture archiving, communication, and storage (PACS) systems
	**Regulatory approval**	Compliance with the regulatory approvals for software as a medical device.

With similar priority, we ask researchers who have developed or are working on automatic brain lesion segmentation algorithms to publish software implementations. The benefits and challenges with fully automated versus interactive preprocessing should be assessed in terms of segmentation accuracy and reproducibility for a given, clinically relevant task.

Few articles in our sample evaluated their algorithm on an independent data set on top of the challenge one. We encourage authors to endeavour to test their method on images from other sites and sources. We also encourage collaboration between the authors and independent researchers who may have access to annotated test images. A preprocessing and segmentation method that has an acceptable and consistent performance on images acquired from various sources should finally be tested in clinical conditions. Therefore we strongly advocate close collaboration between researchers and authors of well-performing methods with clinicians. In such a scenario, the acceptability of the method's performance to clinicians can be assessed in conjunction with its relevance for a given task. Moreover, an open dialogue between researchers and clinicians will help build an ABS system that meets the requirements for a clinically useful and usable tool.

Finally, efforts to define steps on the path towards designing and validating a clinically applicable ABS system should be made. We recommend consultation with stakeholders as a key element to verify the actual clinical needs and how to assess to what extent these needs are met by available research.

CONCLUSIONS

This scoping study of automatic brain lesion segmentation on magnetic resonance images shows a field growing at a rapid pace, an imbalance between proposed methods of which there are many and methods implemented for clinical application of which there are few, and a room for improvement of reporting practice with a view to enabling replication, method comparison and implementation. To promote this improvement, we propose a list of recommendations for future studies in the field. We identify knowledge gaps and potentially fruitful avenues for future research.

REFERENCES

1 Therasse P, Arbuck SG, Eisenhauer EA, *et al.* New Guidelines to Evaluate the Response to Treatment in Solid Tumors. *J Natl Cancer Inst* 2000;**92**:205–16. doi:10.1093/jnci/92.3.205

2 Miller AB, Hoogstraten B, Staquet M, *et al.* Reporting results of cancer treatment. *Cancer* 1981;**47**:207–14. doi:https://doi.org/10.1002/1097-0142(19810101)47:1<207::AID-CNCR2820470134>3.0.CO;2-6

3 Fazekas F, Chawluk JB, Alavi A, *et al.* MR signal abnormalities at 1.5 T in Alzheimer's dementia and normal aging. *AJR Am J Roentgenol* 1987;**149**:351–6. doi:10.2214/ajr.149.2.351

4 Ellingson BM, Wen PY, Cloughesy TF. Modified Criteria for Radiographic Response Assessment in Glioblastoma Clinical Trials. *Neurotherapeutics* 2017;**14**:307–20. doi:10.1007/s13311-016-0507-6

For peer review only - http://bmjopen.bmj.com/site/about/guidelines.xhtml

5 Rosenkrantz AB, Hanna TN, Babb JS, *et al.* Changes in Emergency Department Imaging: Perspectives From National Patient Surveys Over Two Decades. *J Am Coll Radiol* 2017;**14**:1282–90. doi:10.1016/j.jacr.2017.03.004

6 Smith-Bindman R, Miglioretti DL, Larson EB. Rising use of diagnostic medical imaging in a large integrated health system. *Health Aff (Millwood)* 2008;**27**:1491–502. doi:10.1377/hlthaff.27.6.1491

7 Bauer S, Wiest R, Nolte L-P, *et al.* A survey of MRI-based medical image analysis for brain tumor studies. *Phys Med Biol* 2013;**58**:R97-129. doi:10.1088/0031-9155/58/13/R97

8 Debette S, Markus HS. The clinical importance of white matter hyperintensities on brain magnetic resonance imaging: systematic review and meta-analysis. *BMJ* 2010;**341**. doi:10.1136/bmj.c3666

9 Deepa, Singh A. Review of brain tumor detection from MRI images. In: *2016 3rd International Conference on Computing for Sustainable Global Development (INDIACom)*. 2016.

10 García-Lorenzo D, Francis S, Narayanan S, *et al.* Review of automatic segmentation methods of multiple sclerosis white matter lesions on conventional magnetic resonance imaging. *Med Image Anal* 2013;**17**:1–18. doi:10.1016/j.media.2012.09.004

11 Ali Işın, Cem Direkoğlu, Melike Şah. Review of MRI-based Brain Tumor Image Segmentation Using Deep Learning Methods. *Procedia Computer Science* 2016;**102**:317–24. doi:10.1016/j.procs.2016.09.407

12 Ito KL, Kim H, Liew S-L. A comparison of automated lesion segmentation approaches for chronic stroke T1-weighted MRI data. *Hum Brain Mapp* 2019;**40**:4669–85. doi:10.1002/hbm.24729

13 Kumari N, Saxena S. Review of Brain Tumor Segmentation and Classification. In: *2018 International Conference on Current Trends towards Converging Technologies (ICCTCT)*. 2018. 1–6. doi:10.1109/ICCTCT.2018.8551004

14 Mortazavi D, Kouzani AZ, Soltanian-Zadeh H. Segmentation of multiple sclerosis lesions in MR images: a review. *Neuroradiology* 2012;**54**:299–320. doi:10.1007/s00234-011-0886-7

15 Roy S, Nag S, Maitra IK, *et al.* A Review on Automated Brain Tumor Detection and Segmentation from MRI of Brain. *arXiv:13126150 [cs]* Published Online First: 16 December 2013.http://arxiv.org/abs/1312.6150 (accessed 23 Nov 2020).

16 Saritha S, Prabha NA. A comprehensive review: Segmentation of MRI images—brain tumor. *International Journal of Imaging Systems and Technology* 2016;**26**:295–304. doi:https://doi.org/10.1002/ima.22201

17 Arksey H, O'Malley L. Scoping studies: towards a methodological framework. *International Journal of Social Research Methodology* 2005;**8**:19–32. doi:10.1080/1364557032000119616

18 Levac D, Colquhoun H, O'Brien KK. Scoping studies: advancing the methodology. *Implementation Science* 2010;**5**:69. doi:10.1186/1748-5908-5-69

19 Colquhoun HL, Levac D, O'Brien KK, *et al.* Scoping reviews: time for clarity in definition, methods, and reporting. *J Clin Epidemiol* 2014;**67**:1291–4. doi:10.1016/j.jclinepi.2014.03.013

20 Moher D, Liberati A, Tetzlaff J, *et al.* Preferred reporting items for systematic reviews and meta-analyses: the PRISMA statement. *PLoS Med* 2009;**6**:e1000097. doi:10.1371/journal.pmed.1000097

21 Tricco AC, Lillie E, Zarin W, *et al.* PRISMA Extension for Scoping Reviews (PRISMA-ScR): Checklist and Explanation. *Ann Intern Med* 2018;**169**:467–73. doi:10.7326/M18-0850

For peer review only

22 Gryska EA, Schneiderman J, Heckemann RA. Automatic brain lesion segmentation on standard MRIs of the human head: a scoping review protocol. *BMJ Open* 2019;**9**:e024824. doi:10.1136/bmjopen-2018-024824

23 Zijdenbos AP, Forghani R, Evans AC. Automatic "pipeline" analysis of 3-D MRI data for clinical trials: application to multiple sclerosis. *IEEE Trans Med Imaging* 2002;**21**:1280–91. doi:10.1109/TMI.2002.806283

24 Zhuge Y, Krauze AV, Ning H, *et al.* Brain Tumor Segmentation using Holistically-Nested Neural Networks in MRI Images. *Med Phys* 2017;**44**:5234–43. doi:10.1002/mp.12481

25 Zhong Y, Utriainen D, Wang Y, *et al.* Automated White Matter Hyperintensity Detection in Multiple Sclerosis Using 3D T2 FLAIR. *International Journal of Biomedical Imaging* 2014;**2014**:e239123. doi:https://doi.org/10.1155/2014/239123

26 Zhao Z, Yang G, Lin Y, *et al.* Automated glioma detection and segmentation using graphical models. *PLOS ONE* 2018;**13**:e0200745. doi:10.1371/journal.pone.0200745

27 Zhao X, Wu Y, Song G, *et al.* A deep learning model integrating FCNNs and CRFs for brain tumor segmentation. *Med Image Anal* 2018;**43**:98–111. doi:10.1016/j.media.2017.10.002

28 Zhao L, Jia K. Multiscale CNNs for Brain Tumor Segmentation and Diagnosis. *Computational and Mathematical Methods in Medicine* 2016;**2016**:e8356294. doi:https://doi.org/10.1155/2016/8356294

29 Zhang R, Zhao L, Lou W, *et al.* Automatic Segmentation of Acute Ischemic Stroke From DWI Using 3-D Fully Convolutional DenseNets. *IEEE Trans Med Imaging* 2018;**37**:2149–60. doi:10.1109/TMI.2018.2821244

30 Zhang N, Ruan S, Lebonvallet S, *et al.* Kernel feature selection to fuse multi-spectral MRI images for brain tumor segmentation. *Computer Vision and Image Understanding* 2011;**115**:256–69. doi:10.1016/j.cviu.2010.09.007

31 Zhang J, Barboriak DP, Hobbs H, *et al.* A fully automatic extraction of magnetic resonance image features in glioblastoma patients. *Med Phys* 2014;**41**:042301. doi:10.1118/1.4866218

32 Zhan T, Chen Y, Hong X, *et al.* Brain Tumor Segmentation Using Deep Belief Networks and Pathological Knowledge. *CNS Neurol Disord Drug Targets* 2017;**16**:129–36. doi:10.2174/1871527316666170113101559

33 Zhan T, Shen F, Hong X, *et al.* A Glioma Segmentation Method Using CoTraining and Superpixel-Based Spatial and Clinical Constraints. *IEEE Access* 2018;**6**:57113–22. doi:10.1109/ACCESS.2018.2873674

34 Zacharaki EI, Bezerianos A. Abnormality Segmentation in Brain Images Via Distributed Estimation. *IEEE Transactions on Information Technology in Biomedicine* 2012;**16**:330–8. doi:10.1109/TITB.2011.2178422

35 Yu R, Xiao L, Wei Z. Coarse Classification to Region-Scalable Refining for White Matter Lesions Segmentation in Multi-Channel MRI. *CNS Neurol Disord Drug Targets* 2017;**16**:150–9. doi:10.2174/1871527315666161220145247

36 Yoo BI, Lee JJ, Han JW, *et al.* Application of variable threshold intensity to segmentation for white matter hyperintensities in fluid attenuated inversion recovery magnetic resonance images. *Neuroradiology* 2014;**56**:265–81. doi:10.1007/s00234-014-1322-6

37 Wu W, Chen AYC, Zhao L, *et al.* Brain tumor detection and segmentation in a CRF (conditional random fields) framework with pixel-pairwise affinity and superpixel-level features. *Int J Comput Assist Radiol Surg* 2014;**9**:241–53. doi:10.1007/s11548-013-0922-7

38 Weizman L, Ben Sira L, Joskowicz L, *et al.* Automatic segmentation, internal classification, and follow-up of optic pathway gliomas in MRI. *Med Image Anal* 2012;**16**:177–88. doi:10.1016/j.media.2011.07.001

39 Wang Y, Zhou Y, Wang H, *et al.* Voxel-based automated detection of focal cortical dysplasia lesions using diffusion tensor imaging and T2-weighted MRI data. *Epilepsy & Behavior* 2018;**84**:127–34. doi:10.1016/j.yebeh.2018.04.005

40 Wang Y, Catindig JA, Hilal S, *et al.* Multi-stage segmentation of white matter hyperintensity, cortical and lacunar infarcts. *Neuroimage* 2012;**60**:2379–88. doi:10.1016/j.neuroimage.2012.02.034

41 Wang R, Li C, Wang J, *et al.* Automatic segmentation and volumetric quantification of white matter hyperintensities on fluid-attenuated inversion recovery images using the extreme value distribution. *Neuroradiology* 2015;**57**:307–20. doi:10.1007/s00234-014-1466-4

42 Wang R, Li C, Wang J, *et al.* Automatic segmentation of white matter lesions on magnetic resonance images of the brain by using an outlier detection strategy. *Magnetic Resonance Imaging* 2014;**32**:1321–9. doi:10.1016/j.mri.2014.08.010

43 Wang R, Li C, Wang J, *et al.* Automatic Segmentation and Quantitative Analysis of White Matter Hyperintensities on FLAIR Images Using Trimmed-Likelihood Estimator. *Academic Radiology* 2014;**21**:1512–23. doi:10.1016/j.acra.2014.07.001

44 Wang H, Yushkevich PA. Multi-atlas segmentation without registration: a supervoxel-based approach. *Med Image Comput Comput Assist Interv* 2013;**16**:535–42. doi:10.1007/978-3-642-40760-4_67

45 Vishnuvarthanan A, Rajasekaran MP, Govindaraj V, *et al.* Development of a combinational framework to concurrently perform tissue segmentation and tumor identification in T1 - W, T2 - W, FLAIR and MPR type magnetic resonance brain images. *Expert Systems with Applications* 2018;**95**:280–311. doi:10.1016/j.eswa.2017.11.040

46 Vijayakumar C, Damayanti G, Pant R, *et al.* Segmentation and grading of brain tumors on apparent diffusion coefficient images using self-organizing maps. *Comput Med Imaging Graph* 2007;**31**:473–84. doi:10.1016/j.compmedimag.2007.04.004

47 Vidyaratne L, Alam M, Shboul Z, *et al.* Deep Learning and Texture-Based Semantic Label Fusion for Brain Tumor Segmentation. *Proc SPIE Int Soc Opt Eng* 2018;**2018**. doi:10.1117/12.2292930

48 Verma R, Zacharaki EI, Ou Y, *et al.* Multiparametric tissue characterization of brain neoplasms and their recurrence using pattern classification of MR images. *Acad Radiol* 2008;**15**:966–77. doi:10.1016/j.acra.2008.01.029

49 Van Leemput K, Maes F, Vandermeulen D, *et al.* Automated segmentation of multiple sclerosis lesions by model outlier detection. *IEEE Trans Med Imaging* 2001;**20**:677–88. doi:10.1109/42.938237

50 Valverde S, Cabezas M, Roura E, *et al.* Improving automated multiple sclerosis lesion segmentation with a cascaded 3D convolutional neural network approach. *NeuroImage* Published Online First: 2017. doi:10.1016/j.neuroimage.2017.04.034

51 Valcarcel AM, Linn KA, Vandekar SN, *et al.* MIMoSA: An Automated Method for Inter-Modal Segmentation Analysis of Multiple Sclerosis Brain Lesions. *J Neuroimaging* 2018;**28**:389–98. doi:10.1111/jon.12506

52 Usman K, Rajpoot K. Brain tumor classification from multi-modality MRI using wavelets and machine learning. *Pattern Anal Applic* 2017;**20**:871–81. doi:10.1007/s10044-017-0597-8

For peer review only

53 Tustison NJ, Shrinidhi KL, Wintermark M, *et al.* Optimal Symmetric Multimodal Templates and Concatenated Random Forests for Supervised Brain Tumor Segmentation (Simplified) with ANTsR. *Neuroinformatics* 2015;**13**:209–25. doi:10.1007/s12021-014-9245-2

54 Tsai J-Z, Peng S-J, Chen Y-W, *et al.* Automated Segmentation and Quantification of White Matter Hyperintensities in Acute Ischemic Stroke Patients with Cerebral Infarction. *PLoS One* 2014;**9**. doi:10.1371/journal.pone.0104011

55 Tsai J-Z, Peng S-J, Chen Y-W, *et al.* Automatic Detection and Quantification of Acute Cerebral Infarct by Fuzzy Clustering and Histographic Characterization on Diffusion Weighted MR Imaging and Apparent Diffusion Coefficient Map. *BioMed Research International* 2014;**2014**:e963032. doi:https://doi.org/10.1155/2014/963032

56 Tong J, Zhao Y, Zhang P, *et al.* MRI brain tumor segmentation based on texture features and kernel sparse coding. *Biomedical Signal Processing and Control* 2019;**47**:387–92. doi:10.1016/j.bspc.2018.06.001

57 Tong J, Zhang P, Weng Y, *et al.* Kernel sparse representation for MRI image analysis in automatic brain tumor segmentation. *Frontiers Inf Technol Electronic Eng* 2018;**19**:471–80. doi:10.1631/FITEE.1620342

58 Tomas-Fernandez X, Warfield SK. A Model of Population and Subject (MOPS) Intensities with Application to Multiple Sclerosis Lesion Segmentation. *IEEE Trans Med Imaging* 2015;**34**:1349–61. doi:10.1109/TMI.2015.2393853

59 Thomas T, S AR. A Novel Automatic Method for Extraction of Glioma Tumor, White matter and Grey matter from Brain Magnetic Resonant Images. *Biomedical imaging intervention Journal* 2013;**9**. doi:10.2349/biij.9.2.e21

60 Thiruvenkadam K, Perumal N. Fully automatic method for segmentation of brain tumor from multimodal magnetic resonance images using wavelet transformation and clustering technique. *International Journal of Imaging Systems and Technology* 2016;**26**:305–14. doi:https://doi.org/10.1002/ima.22202

61 Szwarc P, Kawa J, Rudzki M, *et al.* Automatic brain tumour detection and neovasculature assessment with multiseries MRI analysis. *Computerized Medical Imaging and Graphics* 2015;**46**:178–90. doi:10.1016/j.compmedimag.2015.06.002

62 Sweeney EM, Vogelstein JT, Cuzzocreo JL, *et al.* A Comparison of Supervised Machine Learning Algorithms and Feature Vectors for MS Lesion Segmentation Using Multimodal Structural MRI. *PLoS One* 2014;**9**. doi:10.1371/journal.pone.0095753

63 Sweeney EM, Shinohara RT, Shiee N, *et al.* OASIS is Automated Statistical Inference for Segmentation, with applications to multiple sclerosis lesion segmentation in MRI. *NeuroImage: Clinical* 2013;**2**:402–13. doi:10.1016/j.nicl.2013.03.002

64 Sweeney EM, Shinohara RT, Shea CD, *et al.* Automatic Lesion Incidence Estimation and Detection in Multiple Sclerosis Using Multisequence Longitudinal MRI. *AJNR Am J Neuroradiol* 2013;**34**:68–73. doi:10.3174/ajnr.A3172

65 Sudre CH, Cardoso MJ, Bouvy WH, *et al.* Bayesian Model Selection for Pathological Neuroimaging Data Applied to White Matter Lesion Segmentation. *IEEE Transactions on Medical Imaging* 2015;**34**:2079–102. doi:10.1109/TMI.2015.2419072

66 Strumia M, Schmidt FR, Anastasopoulos C, *et al.* White Matter MS-Lesion Segmentation Using a Geometric Brain Model. *IEEE Transactions on Medical Imaging* 2016;**35**:1636–46. doi:10.1109/TMI.2016.2522178

67 Stone JR, Wilde EA, Taylor BA, *et al.* Supervised learning technique for the automated identification of white matter hyperintensities in traumatic brain injury. *Brain Injury* 2016;**30**:1458–68. doi:10.1080/02699052.2016.1222080

68 Steenwijk MD, Pouwels PJW, Daams M, *et al.* Accurate white matter lesion segmentation by k nearest neighbor classification with tissue type priors (kNN-TTPs). *NeuroImage: Clinical* 2013;**3**:462–9. doi:10.1016/j.nicl.2013.10.003

69 Steed TC, Treiber JM, Patel KS, *et al.* Iterative probabilistic voxel labeling: automated segmentation for analysis of The Cancer Imaging Archive glioblastoma images. *AJNR Am J Neuroradiol* 2015;**36**:678–85. doi:10.3174/ajnr.A4171

70 Soltaninejad M, Yang G, Lambrou T, *et al.* Supervised learning based multimodal MRI brain tumour segmentation using texture features from supervoxels. *Computer Methods and Programs in Biomedicine* 2018;**157**:69–84. doi:10.1016/j.cmpb.2018.01.003

71 Soltaninejad M, Yang G, Lambrou T, *et al.* Automated brain tumour detection and segmentation using superpixel-based extremely randomized trees in FLAIR MRI. *Int J CARS* 2017;**12**:183–203. doi:10.1007/s11548-016-1483-3

72 Smart SD, Firbank MJ, O'Brien JT. Validation of Automated White Matter Hyperintensity Segmentation. *Journal of Aging Research* 2011;**2011**:e391783. doi:https://doi.org/10.4061/2011/391783

73 Sivakumar P, Ganeshkumar P. An efficient automated methodology for detecting and segmenting the ischemic stroke in brain MRI images. *International Journal of Imaging Systems and Technology* 2017;**27**:265–72. doi:https://doi.org/10.1002/ima.22231

74 Simões R, Mönninghoff C, Dlugaj M, *et al.* Automatic segmentation of cerebral white matter hyperintensities using only 3D FLAIR images. *Magn Reson Imaging* 2013;**31**:1182–9. doi:10.1016/j.mri.2012.12.004

75 Shimol EB, Joskowicz L, Eliahou R, *et al.* Computer-based radiological longitudinal evaluation of meningiomas following stereotactic radiosurgery. *Int J CARS* 2018;**13**:215–28. doi:10.1007/s11548-017-1673-7

76 Shiee N, Bazin P-L, Ozturk A, *et al.* A topology-preserving approach to the segmentation of brain images with multiple sclerosis lesions. *Neuroimage* 2010;**49**:1524–35. doi:10.1016/j.neuroimage.2009.09.005

77 Shi L, Wang D, Liu S, *et al.* Automated quantification of white matter lesion in magnetic resonance imaging of patients with acute infarction. *J Neurosci Methods* 2013;**213**:138–46. doi:10.1016/j.jneumeth.2012.12.014

78 Selvathi D, Selvaraj H, Selvi ST. Hybrid approach for brain tumor segmentation in magnetic resonance images using cellular neural networks and optimization techniques. *Int J Comp Intel Appl* 2010;**09**:17–31. doi:10.1142/S1469026810002781

79 Scully M, Anderson B, Lane T, *et al.* An Automated Method for Segmenting White Matter Lesions through Multi-Level Morphometric Feature Classification with Application to Lupus. *Front Hum Neurosci* 2010;**4**. doi:10.3389/fnhum.2010.00027

80 Schmidt P, Gaser C, Arsic M, *et al.* An automated tool for detection of FLAIR-hyperintense white-matter lesions in Multiple Sclerosis. *Neuroimage* 2012;**59**:3774–83. doi:10.1016/j.neuroimage.2011.11.032

81 Sasikanth S, Kumar SS. Glioma tumor detection in brain MRI image using ANFIS-based normalized graph cut approach. *International Journal of Imaging Systems and Technology* 2018;**28**:64–71. doi:https://doi.org/10.1002/ima.22257

82 Sanjuán A, Price CJ, Mancini L, *et al.* Automated identification of brain tumors from single MR images based on segmentation with refined patient-specific priors. *Front Neurosci* 2013;**7**. doi:10.3389/fnins.2013.00241

83 Samaille T, Fillon L, Cuingnet R, *et al.* Contrast-based fully automatic segmentation of white matter hyperintensities: method and validation. *PLoS One* 2012;**7**:e48953. doi:10.1371/journal.pone.0048953

84 Salem M, Cabezas M, Valverde S, *et al.* A supervised framework with intensity subtraction and deformation field features for the detection of new T2-w lesions in multiple sclerosis. *NeuroImage: Clinical* 2018;**17**:607–15. doi:10.1016/j.nicl.2017.11.015

85 Saad M Norhashimah. Fully Automated Region Growing Segmentation of Brain Lesion in Diffusion-weighted MRI. *IAENG International Journal of Computer Science* 2012;**39**:10.

86 Rundo L, Militello C, Tangherloni A, *et al.* NeXt for neuro-radiosurgery: A fully automatic approach for necrosis extraction in brain tumor MRI using an unsupervised machine learning technique. *International Journal of Imaging Systems and Technology* 2018;**28**:21–37. doi:https://doi.org/10.1002/ima.22253

87 Roy S, He Q, Sweeney E, *et al.* Subject Specific Sparse Dictionary Learning for Atlas Based Brain MRI Segmentation. *IEEE J Biomed Health Inform* 2015;**19**:1598–609. doi:10.1109/JBHI.2015.2439242

88 Roy S, He Q, Carass A, *et al.* Example Based Lesion Segmentation. *Proc SPIE Int Soc Opt Eng* 2014;**9034**. doi:10.1117/12.2043917

89 Roy PK, Bhuiyan A, Janke A, *et al.* Automatic white matter lesion segmentation using contrast enhanced FLAIR intensity and Markov Random Field. *Computerized Medical Imaging and Graphics* 2015;**45**:102–11. doi:10.1016/j.compmedimag.2015.08.005

90 Roura E, Sarbu N, Oliver A, *et al.* Automated Detection of Lupus White Matter Lesions in MRI. *Front Neuroinform* 2016;**10**. doi:10.3389/fninf.2016.00033

91 Roura E, Oliver A, Cabezas M, *et al.* A toolbox for multiple sclerosis lesion segmentation. *Neuroradiology* 2015;**57**:1031–43. doi:10.1007/s00234-015-1552-2

92 Rios Velazquez E, Meier R, Dunn Jr WD, *et al.* Fully automatic GBM segmentation in the TCGA-GBM dataset: Prognosis and correlation with VASARI features. *Sci Rep* 2015;**5**. doi:10.1038/srep16822

93 Rincón M, Díaz-López E, Selnes P, *et al.* Improved Automatic Segmentation of White Matter Hyperintensities in MRI Based on Multilevel Lesion Features. *Neuroinform* 2017;**15**:231–45. doi:10.1007/s12021-017-9328-y

94 Reza S, Iftekharuddin K. Multi-class Abnormal Brain Tissue Segmentation Using Texture Features. *Proc NCI MICCAI-BRATS* 2013;**2013**:38–42.

95 Razzak MI, Imran M, Xu G. Efficient Brain Tumor Segmentation With Multiscale Two-Pathway-Group Conventional Neural Networks. *IEEE Journal of Biomedical and Health Informatics* 2019;**23**:1911–9. doi:10.1109/JBHI.2018.2874033

96 Raju AR, Suresh P, Rao RR. Bayesian HCS-based multi-SVNN: A classification approach for brain tumor segmentation and classification using Bayesian fuzzy clustering. *Biocybernetics and Biomedical Engineering* 2018;**38**:646–60. doi:10.1016/j.bbe.2018.05.001

97 Rajinikanth V, Satapathy SC, Fernandes SL, *et al.* Entropy based segmentation of tumor from brain MR images – a study with teaching learning based optimization. *Pattern Recognition Letters* 2017;**94**:87–95. doi:10.1016/j.patrec.2017.05.028

For peer review only

98 Rachmadi MF, Valdés-Hernández M del C, Agan MLF, *et al.* Segmentation of white matter hyperintensities using convolutional neural networks with global spatial information in routine clinical brain MRI with none or mild vascular pathology. *Computerized Medical Imaging and Graphics* 2018;**66**:28–43. doi:10.1016/j.compmedimag.2018.02.002

99 Qu X, Yang J, Ma S, *et al.* Positive Unanimous Voting Algorithm for Focal Cortical Dysplasia Detection on Magnetic Resonance Image. *Front Comput Neurosci* 2016;**10**. doi:10.3389/fncom.2016.00025

100 Qin C, Guerrero R, Bowles C, *et al.* A large margin algorithm for automated segmentation of white matter hyperintensity. *Pattern Recognition* 2018;**77**:150–9. doi:10.1016/j.patcog.2017.12.016

101 Pustina D, Coslett HB, Turkeltaub PE, *et al.* Automated segmentation of chronic stroke lesions using LINDA: Lesion identification with neighborhood data analysis. *Hum Brain Mapp* 2016;**37**:1405–21. doi:10.1002/hbm.23110

102 Praveen GB, Agrawal A, Sundaram P, *et al.* Ischemic stroke lesion segmentation using stacked sparse autoencoder. *Computers in Biology and Medicine* 2018;**99**:38–52. doi:10.1016/j.compbiomed.2018.05.027

103 Prakash K N B, Gupta V, Bilello M, *et al.* Identification, segmentation, and image property study of acute infarcts in diffusion-weighted images by using a probabilistic neural network and adaptive Gaussian mixture model. *Acad Radiol* 2006;**13**:1474–84. doi:10.1016/j.acra.2006.09.045

104 Porz N, Habegger S, Meier R, *et al.* Fully Automated Enhanced Tumor Compartmentalization: Man vs. Machine Reloaded. *PLoS One* 2016;**11**. doi:10.1371/journal.pone.0165302

105 Porz N, Bauer S, Pica A, *et al.* Multi-Modal Glioblastoma Segmentation: Man versus Machine. *PLOS ONE* 2014;**9**:e96873. doi:10.1371/journal.pone.0096873

106 Popuri K, Cobzas D, Murtha A, *et al.* 3D variational brain tumor segmentation using Dirichlet priors on a clustered feature set. *Int J Comput Assist Radiol Surg* 2012;**7**:493–506. doi:10.1007/s11548-011-0649-2

107 Pinto A, Pereira S, Rasteiro D, *et al.* Hierarchical brain tumour segmentation using extremely randomized trees. *Pattern Recognition* 2018;**82**:105–17. doi:10.1016/j.patcog.2018.05.006

108 Pereira S, Pinto A, Alves V, *et al.* Brain Tumor Segmentation Using Convolutional Neural Networks in MRI Images. *IEEE Transactions on Medical Imaging* 2016;**35**:1240–51. doi:10.1109/TMI.2016.2538465

109 Parisot S, Wells W, Chemouny S, *et al.* Concurrent tumor segmentation and registration with uncertainty-based sparse non-uniform graphs. *Medical Image Analysis* 2014;**18**:647–59. doi:10.1016/j.media.2014.02.006

110 Pagnozzi AM, Dowson N, Doecke J, *et al.* Automated, quantitative measures of grey and white matter lesion burden correlates with motor and cognitive function in children with unilateral cerebral palsy. *Neuroimage Clin* 2016;**11**:751–9. doi:10.1016/j.nicl.2016.05.018

111 Ozenne B, Subtil F, Østergaard L, *et al.* Spatially regularized mixture model for lesion segmentation with application to stroke patients. *Biostatistics* 2015;**16**:580–95. doi:10.1093/biostatistics/kxv004

112 Ong KH, Ramachandram D, Mandava R, *et al.* Automatic white matter lesion segmentation using an adaptive outlier detection method. *Magn Reson Imaging* 2012;**30**:807–23. doi:10.1016/j.mri.2012.01.007

113 Njeh I, Sallemi L, Ayed IB, *et al.* 3D multimodal MRI brain glioma tumor and edema segmentation: A graph cut distribution matching approach. *Computerized Medical Imaging and Graphics* 2015;**40**:108–19. doi:10.1016/j.compmedimag.2014.10.009

114 Nie J, Xue Z, Liu T, *et al.* Automated brain tumor segmentation using spatial accuracy-weighted hidden Markov Random Field. *Computerized Medical Imaging and Graphics* 2009;**33**:431–41. doi:10.1016/j.compmedimag.2009.04.006

115 Nagenthiraja K, Walcott BP, Hansen MB, *et al.* Automated Decision-Support System for Prediction of Treatment Responders in Acute Ischemic Stroke. *Front Neurol* 2013;**4**. doi:10.3389/fneur.2013.00140

116 naceur MB, Saouli R, Akil M, *et al.* Fully Automatic Brain Tumor Segmentation using End-To-End Incremental Deep Neural Networks in MRI images. *Computer Methods and Programs in Biomedicine* 2018;**166**:39–49. doi:10.1016/j.cmpb.2018.09.007

117 Nabizadeh N, Kubat M. Brain tumors detection and segmentation in MR images: Gabor wavelet vs. statistical features. *Computers & Electrical Engineering* 2015;**45**:286–301. doi:10.1016/j.compeleceng.2015.02.007

118 Murphy K, van der Aa NE, Negro S, *et al.* Automatic quantification of ischemic injury on diffusion-weighted MRI of neonatal hypoxic ischemic encephalopathy. *Neuroimage Clin* 2017;**14**:222–32. doi:10.1016/j.nicl.2017.01.005

119 Muda AF, Mohd Saad N, Abu Bakar SAR, *et al.* Brain lesion segmentation using fuzzy C-means on diffusion-weighted imaging. *ARPN Journal of Engineering and Applied Sciences* 2015;**10**:1138–44.

120 Mouridsen K, Nagenthiraja K, Jónsdóttir KÝ, *et al.* Acute stroke: automatic perfusion lesion outlining using level sets. *Radiology* 2013;**269**:404–12. doi:10.1148/radiol.13121622

121 Moeskops P, de Bresser J, Kuijf HJ, *et al.* Evaluation of a deep learning approach for the segmentation of brain tissues and white matter hyperintensities of presumed vascular origin in MRI. *NeuroImage: Clinical* 2018;**17**:251–62. doi:10.1016/j.nicl.2017.10.007

122 Mitra S, Banerjee S, Hayashi Y. Volumetric brain tumour detection from MRI using visual saliency. *PLOS ONE* 2017;**12**:e0187209. doi:10.1371/journal.pone.0187209

123 Mitra J, Bourgeat P, Fripp J, *et al.* Lesion segmentation from multimodal MRI using random forest following ischemic stroke. *NeuroImage* 2014;**98**:324–35. doi:10.1016/j.neuroimage.2014.04.056

124 Menze BH, Van Leemput K, Lashkari D, *et al.* A generative probabilistic model and discriminative extensions for brain lesion segmentation – with application to tumor and stroke. *IEEE Trans Med Imaging* 2016;**35**:933–46. doi:10.1109/TMI.2015.2502596

125 Meier R, Porz N, Knecht U, *et al.* Automatic estimation of extent of resection and residual tumor volume of patients with glioblastoma. *J Neurosurg* 2017;**127**:798–806. doi:10.3171/2016.9.JNS16146

126 Meier R, Knecht U, Loosli T, *et al.* Clinical Evaluation of a Fully-automatic Segmentation Method for Longitudinal Brain Tumor Volumetry. *Sci Rep* 2016;**6**:23376. doi:10.1038/srep23376

127 Meier DS, Guttmann CRG, Tummala S, *et al.* Dual-Sensitivity Multiple Sclerosis Lesion and CSF Segmentation for Multichannel 3T Brain MRI. *J Neuroimaging* 2018;**28**:36–47. doi:10.1111/jon.12491

128 Mehta S, Grabowski TJ, Trivedi Y, *et al.* Evaluation of voxel-based morphometry for focal lesion detection in individuals. *NeuroImage* 2003;**20**:1438–54. doi:10.1016/S1053-8119(03)00377-X

129 Mechrez R, Goldberger J, Greenspan H. Patch-Based Segmentation with Spatial Consistency: Application to MS Lesions in Brain MRI. *Int J Biomed Imaging* 2016;**2016**. doi:10.1155/2016/7952541

For peer review only - http://bmjopen.bmj.com/site/about/guidelines.xhtml

130 McKinley R, Häni L, Gralla J, *et al.* Fully automated stroke tissue estimation using random forest classifiers (FASTER). *J Cereb Blood Flow Metab* 2017;**37**:2728–41. doi:10.1177/0271678X16674221

131 Mazzara GP, Velthuizen RP, Pearlman JL, *et al.* Brain tumor target volume determination for radiation treatment planning through automated MRI segmentation. *International Journal of Radiation Oncology*Biology*Physics* 2004;**59**:300–12. doi:10.1016/j.ijrobp.2004.01.026

132 Manjón JV, Coupé P, Raniga P, *et al.* MRI white matter lesion segmentation using an ensemble of neural networks and overcomplete patch-based voting. *Computerized Medical Imaging and Graphics* 2018;**69**:43–51. doi:10.1016/j.compmedimag.2018.05.001

133 Maldjian JA, Whitlow CT, Saha BN, *et al.* Automated White Matter Total Lesion Volume Segmentation in Diabetes. *AJNR Am J Neuroradiol* 2013;**34**:2265–70. doi:10.3174/ajnr.A3590

134 Maji P, Roy S. SoBT-RFW: Rough-Fuzzy Computing and Wavelet Analysis Based Automatic Brain Tumor Detection Method from MR Images. *Fundam Informaticae* Published Online First: 2015. doi:10.3233/FI-2015-1293

135 Maier O, Wilms M, von der Gablentz J, *et al.* Extra Tree forests for sub-acute ischemic stroke lesion segmentation in MR sequences. *Journal of Neuroscience Methods* 2015;**240**:89–100. doi:10.1016/j.jneumeth.2014.11.011

136 Maier O, Schröder C, Forkert ND, *et al.* Classifiers for Ischemic Stroke Lesion Segmentation: A Comparison Study. *PLOS ONE* 2015;**10**:e0145118. doi:10.1371/journal.pone.0145118

137 Maier O. MS lesion segmentation in MRI with random forests. *Proc 2015 Longitudinal Multiple Sclerosis Lesion Segmentation Challenge* 2015;:1–2.

138 Mah Y-H, Jager R, Kennard C, *et al.* A new method for automated high-dimensional lesion segmentation evaluated in vascular injury and applied to the human occipital lobe. *Cortex* 2014;**56**:51–63. doi:10.1016/j.cortex.2012.12.008

139 Ma C, Luo G, Wang K. Concatenated and Connected Random Forests With Multiscale Patch Driven Active Contour Model for Automated Brain Tumor Segmentation of MR Images. *IEEE Transactions on Medical Imaging* 2018;**37**:1943–54. doi:10.1109/TMI.2018.2805821

140 Lu Y, Jiang J, Yang W, *et al.* Multimodal Brain-Tumor Segmentation Based on Dirichlet Process Mixture Model with Anisotropic Diffusion and Markov Random Field Prior. *Computational and Mathematical Methods in Medicine* 2014;**2014**:e717206. doi:https://doi.org/10.1155/2014/717206

141 Liu Y, Stojadinovic S, Hrycushko B, *et al.* A deep convolutional neural network-based automatic delineation strategy for multiple brain metastases stereotactic radiosurgery. *PLoS One* 2017;**12**:e0185844. doi:10.1371/journal.pone.0185844

142 Liu Y, Stojadinovic S, Hrycushko B, *et al.* Automatic metastatic brain tumor segmentation for stereotactic radiosurgery applications. *Phys Med Biol* 2016;**61**:8440–8461. doi:10.1088/0031-9155/61/24/8440

143 Liu J, Chen F, Pan C, *et al.* A Cascaded Deep Convolutional Neural Network for Joint Segmentation and Genotype Prediction of Brainstem Gliomas. *IEEE Transactions on Biomedical Engineering* 2018;**65**:1943–52. doi:10.1109/TBME.2018.2845706

144 Ling Y, Jouvent E, Cousyn L, *et al.* Validation and Optimization of BIANCA for the Segmentation of Extensive White Matter Hyperintensities. *Neuroinform* 2018;**16**:269–81. doi:10.1007/s12021-018-9372-2

For peer review only

145 Liberman G, Louzoun Y, Aizenstein O, *et al.* Automatic multi-modal MR tissue classification for the assessment of response to bevacizumab in patients with glioblastoma. *European Journal of Radiology* 2013;**82**:e87–94. doi:10.1016/j.ejrad.2012.09.001

146 Li Z, Wang Y, Yu J, *et al.* Low-Grade Glioma Segmentation Based on CNN with Fully Connected CRF. *Journal of Healthcare Engineering* 2017;**2017**:e9283480. doi:https://doi.org/10.1155/2017/9283480

147 Li Y, Jia F, Qin J. Brain tumor segmentation from multimodal magnetic resonance images via sparse representation. *Artificial Intelligence in Medicine* 2016;**73**:1–13. doi:10.1016/j.artmed.2016.08.004

148 Li W, Tian J, Li E, *et al.* Robust unsupervised segmentation of infarct lesion from diffusion tensor MR images using multiscale statistical classification and partial volume voxel reclassification. *Neuroimage* 2004;**23**:1507–18. doi:10.1016/j.neuroimage.2004.08.009

149 Li H, Jiang G, Zhang J, *et al.* Fully convolutional network ensembles for white matter hyperintensities segmentation in MR images. *NeuroImage* 2018;**183**:650–65. doi:10.1016/j.neuroimage.2018.07.005

150 Lesjak Ž, Pernuš F, Likar B, *et al.* Validation of White-Matter Lesion Change Detection Methods on a Novel Publicly Available MRI Image Database. *Neuroinform* 2016;**14**:403–20. doi:10.1007/s12021-016-9301-1

151 Laukamp KR, Thiele F, Shakirin G, *et al.* Fully automated detection and segmentation of meningiomas using deep learning on routine multiparametric MRI. *Eur Radiol* 2019;**29**:124–32. doi:10.1007/s00330-018-5595-8

152 Lao Z, Shen D, Liu D, *et al.* Computer-assisted segmentation of white matter lesions in 3D MR images using support vector machine. *Acad Radiol* 2008;**15**:300–13. doi:10.1016/j.acra.2007.10.012

153 Korfiatis P, Kline TL, Erickson BJ. Automated Segmentation of Hyperintense Regions in FLAIR MRI Using Deep Learning. *Tomography* 2016;**2**:334–40. doi:10.18383/j.tom.2016.00166

154 Knight J, Taylor GW, Khademi A. Voxel-Wise Logistic Regression and Leave-One-Source-Out Cross Validation for white matter hyperintensity segmentation. *Magnetic Resonance Imaging* 2018;**54**:119–36. doi:10.1016/j.mri.2018.06.009

155 Klöppel S, Abdulkadir A, Hadjidemetriou S, *et al.* A comparison of different automated methods for the detection of white matter lesions in MRI data. *NeuroImage* 2011;**57**:416–22. doi:10.1016/j.neuroimage.2011.04.053

156 Khotanlou H, Afrasiabi M. Segmentation of Multiple Sclerosis Lesions in Brain MR Images Using Spatially Constrained Possibilistic Fuzzy C-Means Classification. *J Med Signals Sens* 2011;**1**:149–55.

157 Khayati R, Vafadust M, Towhidkhah F, *et al.* Fully automatic segmentation of multiple sclerosis lesions in brain MR FLAIR images using adaptive mixtures method and Markov random field model. *Comput Biol Med* 2008;**38**:379–90. doi:10.1016/j.compbiomed.2007.12.005

158 Khayati R, Vafadust M, Towhidkhah F, *et al.* A novel method for automatic determination of different stages of multiple sclerosis lesions in brain MR FLAIR images. *Comput Med Imaging Graph* 2008;**32**:124–33. doi:10.1016/j.compmedimag.2007.10.003

159 Khademi A, Venetsanopoulos A, Moody AR. Robust White Matter Lesion Segmentation in FLAIR MRI. *IEEE Transactions on Biomedical Engineering* 2012;**59**:860–71. doi:10.1109/TBME.2011.2181167

160 Kellner-Weldon F, Stippich C, Wiest R, *et al.* Comparison of perioperative automated versus manual two-dimensional tumor analysis in glioblastoma patients. *European Journal of Radiology* 2017;**95**:75–81. doi:10.1016/j.ejrad.2017.07.028

For peer review only

161 Kellner E, Reisert M, Kiselev VG, *et al.* Automated Infarct Core Volumetry Within the Hypoperfused Tissue: Technical Implementation and Evaluation. *J Comput Assist Tomogr* 2017;**41**:515–20. doi:10.1097/RCT.0000000000000570

162 Keçeli AS, Can AB, Kaya A. A GPU-Based Approach for Automatic Segmentation of White Matter Lesions. *IETE Journal of Research* 2017;**63**:461–72. doi:10.1080/03772063.2017.1284619

163 Kaur T, Saini BS, Gupta S. A joint intensity and edge magnitude-based multilevel thresholding algorithm for the automatic segmentation of pathological MR brain images. *Neural Comput & Applic* 2018;**30**:1317–40. doi:10.1007/s00521-016-2751-4

164 Kaur T, Saini BS, Gupta S. A novel fully automatic multilevel thresholding technique based on optimized intuitionistic fuzzy sets and tsallis entropy for MR brain tumor image segmentation. *Australas Phys Eng Sci Med* 2018;**41**:41–58. doi:10.1007/s13246-017-0609-4

165 Karimian A, Jafari S. A New Method to Segment the Multiple Sclerosis Lesions on Brain Magnetic Resonance Images. *J Med Signals Sens* 2015;**5**:238–44.

166 Karimaghaloo Z, Arnold DL, Arbel T. Adaptive multi-level conditional random fields for detection and segmentation of small enhanced pathology in medical images. *Medical Image Analysis* 2016;**27**:17–30. doi:10.1016/j.media.2015.06.004

167 Karimaghaloo Z, Shah M, Francis SJ, *et al.* Automatic Detection of Gadolinium-Enhancing Multiple Sclerosis Lesions in Brain MRI Using Conditional Random Fields. *IEEE Transactions on Medical Imaging* 2012;**31**:1181–94. doi:10.1109/TMI.2012.2186639

168 Karimaghaloo Z, Rivaz H, Arnold DL, *et al.* Temporal Hierarchical Adaptive Texture CRF for Automatic Detection of Gadolinium-Enhancing Multiple Sclerosis Lesions in Brain MRI. *IEEE Transactions on Medical Imaging* 2015;**34**:1227–41. doi:10.1109/TMI.2014.2382561

169 Kanas VG, Zacharaki EI, Davatzikos C, *et al.* A low cost approach for brain tumor segmentation based on intensity modeling and 3D Random Walker. *Biomedical Signal Processing and Control* 2015;**22**:19–30. doi:10.1016/j.bspc.2015.06.004

170 Kamnitsas K, Ledig C, Newcombe VFJ, *et al.* Efficient multi-scale 3D CNN with fully connected CRF for accurate brain lesion segmentation. *Medical Image Analysis* 2017;**36**:61–78. doi:10.1016/j.media.2016.10.004

171 Kamber M, Shinghal R, Collins DL, *et al.* Model-based 3-D segmentation of multiple sclerosis lesions in magnetic resonance brain images. *IEEE Transactions on Medical Imaging* 1995;**14**:442–53. doi:10.1109/42.414608

172 Juan-Albarracín J, Fuster-Garcia E, Manjón JV, *et al.* Automated Glioblastoma Segmentation Based on a Multiparametric Structured Unsupervised Classification. *PLOS ONE* 2015;**10**:e0125143. doi:10.1371/journal.pone.0125143

173 Jiang J, Liu T, Zhu W, *et al.* UBO Detector – A cluster-based, fully automated pipeline for extracting white matter hyperintensities. *NeuroImage* 2018;**174**:539–49. doi:10.1016/j.neuroimage.2018.03.050

174 Ji S, Ye C, Li F, *et al.* Automatic segmentation of white matter hyperintensities by an extended FitzHugh & Nagumo reaction diffusion model. *J Magn Reson Imaging* 2013;**37**:343–50. doi:10.1002/jmri.23836

175 Jeon S, Yoon U, Park J-S, *et al.* Fully automated pipeline for quantification and localization of white matter hyperintensity in brain magnetic resonance image. *International Journal of Imaging Systems and Technology* 2011;**21**:193–200. doi:https://doi.org/10.1002/ima.20277

For peer review only - http://bmjopen.bmj.com/site/about/guidelines.xhtml

176 Jansen JFA, Vlooswijk MCG, Majoie HM, *et al.* White matter lesions in patients with localization-related epilepsy. *Invest Radiol* 2008;**43**:552–8. doi:10.1097/RLI.0b013e31817e90d2

177 Jain S, Sima DM, Ribbens A, *et al.* Automatic segmentation and volumetry of multiple sclerosis brain lesions from MR images. *Neuroimage Clin* 2015;**8**:367–75. doi:10.1016/j.nicl.2015.05.003

178 Ithapu V, Singh V, Lindner C, *et al.* Extracting and summarizing white matter hyperintensities using supervised segmentation methods in Alzheimer's disease risk and aging studies. *Hum Brain Mapp* 2014;**35**:4219–35. doi:10.1002/hbm.22472

179 Islam A, Reza SMS, Iftekharuddin KM. Multifractal Texture Estimation for Detection and Segmentation of Brain Tumors. *IEEE Transactions on Biomedical Engineering* 2013;**60**:3204–15. doi:10.1109/TBME.2013.2271383

180 Iqbal S, Ghani MU, Saba T, *et al.* Brain tumor segmentation in multi-spectral MRI using convolutional neural networks (CNN). *Microsc Res Tech* 2018;**81**:419–27. doi:10.1002/jemt.22994

181 Ilunga–Mbuyamba E, Avina–Cervantes JG, Cepeda–Negrete J, *et al.* Automatic selection of localized region-based active contour models using image content analysis applied to brain tumor segmentation. *Computers in Biology and Medicine* 2017;**91**:69–79. doi:10.1016/j.compbiomed.2017.10.003

182 Ilunga-Mbuyamba E, Cruz-Duarte JM, Avina-Cervantes JG, *et al.* Active contours driven by Cuckoo Search strategy for brain tumour images segmentation. *Expert Systems with Applications* 2016;**56**:59–68. doi:10.1016/j.eswa.2016.02.048

183 Hussain S, Anwar SM, Majid M. Segmentation of glioma tumors in brain using deep convolutional neural network. *Neurocomputing* 2018;**282**:248–61. doi:10.1016/j.neucom.2017.12.032

184 Hulsey KM, Gupta M, King KS, *et al.* Automated quantification of white matter disease extent at 3 T: Comparison with volumetric readings. *Journal of Magnetic Resonance Imaging* 2012;**36**:305–11. doi:https://doi.org/10.1002/jmri.23659

185 Huang M, Yang W, Wu Y, *et al.* Brain Tumor Segmentation Based on Local Independent Projection-Based Classification. *IEEE Transactions on Biomedical Engineering* 2014;**61**:2633–45. doi:10.1109/TBME.2014.2325410

186 Herskovits E, Bryan R, Yang F. Automated Bayesian Segmentation of Microvascular White-Matter Lesions in the ACCORD-MIND Study. *Advances in Medical Sciences* 2008;**53**. doi:10.2478/v10039-008-0039-3

187 Havaei M, Davy A, Warde-Farley D, *et al.* Brain tumor segmentation with Deep Neural Networks. *Medical Image Analysis* 2017;**35**:18–31. doi:10.1016/j.media.2016.05.004

188 Harmouche R, Subbanna NK, Collins DL, *et al.* Probabilistic Multiple Sclerosis Lesion Classification Based on Modeling Regional Intensity Variability and Local Neighborhood Information. *IEEE Transactions on Biomedical Engineering* 2015;**62**:1281–92. doi:10.1109/TBME.2014.2385635

189 Harati V, Khayati R, Farzan A. Fully automated tumor segmentation based on improved fuzzy connectedness algorithm in brain MR images. *Computers in Biology and Medicine* 2011;**41**:483–92. doi:10.1016/j.compbiomed.2011.04.010

190 Hansen MB, Nagenthiraja K, Ribe LR, *et al.* Automated estimation of salvageable tissue: Comparison with expert readers. *Journal of Magnetic Resonance Imaging* 2016;**43**:220–8. doi:https://doi.org/10.1002/jmri.24963

For peer review only

191 Gupta N, Bhatele P, Khanna P. Identification of Gliomas from brain MRI through adaptive segmentation and run length of centralized patterns. *Journal of Computational Science* 2018;**25**:213–20. doi:10.1016/j.jocs.2017.02.009

192 Guo D, Fridriksson J, Fillmore P, *et al.* Automated lesion detection on MRI scans using combined unsupervised and supervised methods. *BMC Medical Imaging* 2015;**15**:50. doi:10.1186/s12880-015-0092-x

193 Guizard N, Coupé P, Fonov VS, *et al.* Rotation-invariant multi-contrast non-local means for MS lesion segmentation. *Neuroimage-Clinical* 2015;**8**:376–389. doi:10.1016/j.nicl.2015.05.001

194 Guerrero R, Qin C, Oktay O, *et al.* White matter hyperintensity and stroke lesion segmentation and differentiation using convolutional neural networks. *NeuroImage: Clinical* 2018;**17**:918–34. doi:10.1016/j.nicl.2017.12.022

195 Griffis JC, Allendorfer JB, Szaflarski JP. Voxel-based Gaussian naïve Bayes classification of ischemic stroke lesions in individual T1-weighted MRI scans. *J Neurosci Methods* 2016;**257**:97–108. doi:10.1016/j.jneumeth.2015.09.019

196 Griffanti L, Zamboni G, Khan A, *et al.* BIANCA (Brain Intensity AbNormality Classification Algorithm): A new tool for automated segmentation of white matter hyperintensities. *Neuroimage* 2016;**141**:191–205. doi:10.1016/j.neuroimage.2016.07.018

197 Gooya A, Pohl KM, Bilello M, *et al.* GLISTR: glioma image segmentation and registration. *IEEE Trans Med Imaging* 2012;**31**:1941–54. doi:10.1109/TMI.2012.2210558

198 Gonçalves N, Nikkilä J, Vigário R. Self-supervised MRI tissue segmentation by discriminative clustering. *Int J Neural Syst* 2014;**24**:1450004. doi:10.1142/S012906571450004X

199 Goetz M, Weber C, Binczyk F, *et al.* DALSA: Domain Adaptation for Supervised Learning From Sparsely Annotated MR Images. *IEEE Transactions on Medical Imaging* 2016;**35**:184–96. doi:10.1109/TMI.2015.2463078

200 Ghribi O, Sellami L, Slima MB, *et al.* Multiple sclerosis exploration based on automatic MRI modalities segmentation approach with advanced volumetric evaluations for essential feature extraction. *Biomedical Signal Processing and Control* 2018;**40**:473–87. doi:10.1016/j.bspc.2017.07.008

201 Ghribi O, Sellami L, Slima MB, *et al.* An Advanced MRI Multi-Modalities Segmentation Methodology Dedicated to Multiple Sclerosis Lesions Exploration and Differentiation. *IEEE Transactions on NanoBioscience* 2017;**16**:656–65. doi:10.1109/TNB.2017.2763246

202 Ghafoorian M, Karssemeijer N, van Uden IWM, *et al.* Automated detection of white matter hyperintensities of all sizes in cerebral small vessel disease. *Med Phys* 2016;**43**:6246. doi:10.1118/1.4966029

203 Ghafoorian M, Karssemeijer N, Heskes T, *et al.* Location Sensitive Deep Convolutional Neural Networks for Segmentation of White Matter Hyperintensities. *Scientific Reports* 2017;**7**:5110. doi:10.1038/s41598-017-05300-5

204 Ghafoorian M, Karssemeijer N, Heskes T, *et al.* Deep multi-scale location-aware 3D convolutional neural networks for automated detection of lacunes of presumed vascular origin. *NeuroImage: Clinical* 2017;**14**:391–9. doi:10.1016/j.nicl.2017.01.033

205 Geremia E, Clatz O, Menze BH, *et al.* Spatial decision forests for MS lesion segmentation in multi-channel magnetic resonance images. *NeuroImage* 2011;**57**:378–90. doi:10.1016/j.neuroimage.2011.03.080

206 García-Lorenzo D, Prima S, Arnold DL, *et al.* Trimmed-likelihood estimation for focal lesions and tissue segmentation in multisequence MRI for multiple sclerosis. *IEEE Trans Med Imaging* 2011;**30**:1455–67. doi:10.1109/TMI.2011.2114671

207 Gao J, Li C, Feng C, *et al.* Non-locally regularized segmentation of multiple sclerosis lesion from multi-channel MRI data. *Magn Reson Imaging* 2014;**32**:1058–66. doi:10.1016/j.mri.2014.03.006

208 Ganiler O, Oliver A, Diez Y, *et al.* A subtraction pipeline for automatic detection of new appearing multiple sclerosis lesions in longitudinal studies. *Neuroradiology* 2014;**56**:363–74. doi:10.1007/s00234-014-1343-1

209 Galimzianova A, Pernuš F, Likar B, *et al.* Stratified mixture modeling for segmentation of white-matter lesions in brain MR images. *NeuroImage* 2016;**124**:1031–43. doi:10.1016/j.neuroimage.2015.09.047

210 Galimzianova A, Lesjak Ž, Rubin DL, *et al.* Locally adaptive magnetic resonance intensity models for unsupervised segmentation of multiple sclerosis lesions. *J Med Imaging (Bellingham)* 2018;**5**. doi:10.1117/1.JMI.5.1.011007

211 Freire PGL, Ferrari RJ. Automatic iterative segmentation of multiple sclerosis lesions using Student's t mixture models and probabilistic anatomical atlases in FLAIR images. *Computers in Biology and Medicine* 2016;**73**:10–23. doi:10.1016/j.compbiomed.2016.03.025

212 Fiot J-B, Cohen LD, Raniga P, *et al.* Efficient brain lesion segmentation using multi-modality tissue-based feature selection and support vector machines. *Int J Numer Method Biomed Eng* 2013;**29**:905–15. doi:10.1002/cnm.2537

213 Fartaria MJ, Todea A, Kober T, *et al.* Partial volume-aware assessment of multiple sclerosis lesions. *Neuroimage Clin* 2018;**18**:245–53. doi:10.1016/j.nicl.2018.01.011

214 Fartaria MJ, Bonnier G, Roche A, *et al.* Automated detection of white matter and cortical lesions in early stages of multiple sclerosis. *Journal of Magnetic Resonance Imaging* 2016;**43**:1445–54. doi:https://doi.org/10.1002/jmri.25095

215 Essadike A, Ouabida E, Bouzid A. Brain tumor segmentation with Vander Lugt correlator based active contour. *Computer Methods and Programs in Biomedicine* 2018;**160**:103–17. doi:10.1016/j.cmpb.2018.04.004

216 Erus G, Zacharaki EI, Davatzikos C. Individualized statistical learning from medical image databases: application to identification of brain lesions. *Med Image Anal* 2014;**18**:542–54. doi:10.1016/j.media.2014.02.003

217 Emblem KE, Nedregaard B, Hald JK, *et al.* Automatic glioma characterization from dynamic susceptibility contrast imaging: brain tumor segmentation using knowledge-based fuzzy clustering. *J Magn Reson Imaging* 2009;**30**:1–10. doi:10.1002/jmri.21815

218 Elliott C, Arnold DL, Collins DL, *et al.* Temporally consistent probabilistic detection of new multiple sclerosis lesions in brain MRI. *IEEE Trans Med Imaging* 2013;**32**:1490–503. doi:10.1109/TMI.2013.2258403

219 Dyrby TB, Rostrup E, Baaré WFC, *et al.* Segmentation of age-related white matter changes in a clinical multi-center study. *NeuroImage* 2008;**41**:335–45. doi:10.1016/j.neuroimage.2008.02.024

220 Dvořák P, Bartusek K, Smékal Z. Unsupervised Pathological Area Extraction using 3D T2 and FLAIR MR Images. Published Online First: 2014. doi:10.2478/msr-2014-0049

For peer review only

221 Ding Y, Dong R, Lan T, *et al.* Multi-modal brain tumor image segmentation based on SDAE. *International Journal of Imaging Systems and Technology* 2018;**28**:38–47. doi:https://doi.org/10.1002/ima.22254

222 Dickson S, Thomas BT, Goddard P. Using neural networks to automatically detect brain tumours in MR images. *Int J Neural Syst* 1997;**8**:91–9. doi:10.1142/s0129065797000124

223 Demirhan A, Törü M, Güler İ. Segmentation of Tumor and Edema Along With Healthy Tissues of Brain Using Wavelets and Neural Networks. *IEEE Journal of Biomedical and Health Informatics* 2015;**19**:1451–8. doi:10.1109/JBHI.2014.2360515

224 de Sitter A, Steenwijk MD, Ruet A, *et al.* Performance of five research-domain automated WM lesion segmentation methods in a multi-center MS study. *Neuroimage* 2017;**163**:106–14. doi:10.1016/j.neuroimage.2017.09.011

225 de Boer R, Vrooman HA, van der Lijn F, *et al.* White matter lesion extension to automatic brain tissue segmentation on MRI. *NeuroImage* 2009;**45**:1151–61. doi:10.1016/j.neuroimage.2009.01.011

226 Datta S, Sajja BR, He R, *et al.* Segmentation of gadolinium-enhanced lesions on MRI in multiple sclerosis. *J Magn Reson Imaging* 2007;**25**:932–7. doi:10.1002/jmri.20896

227 Datta S, Narayana PA. A comprehensive approach to the segmentation of multichannel three-dimensional MR brain images in multiple sclerosis. *Neuroimage Clin* 2013;**2**:184–96. doi:10.1016/j.nicl.2012.12.007

228 Damangir S, Westman E, Simmons A, *et al.* Reproducible segmentation of white matter hyperintensities using a new statistical definition. *Magn Reson Mater Phy* 2017;**30**:227–37. doi:10.1007/s10334-016-0599-3

229 Damangir S, Manzouri A, Oppedal K, *et al.* Multispectral MRI segmentation of age related white matter changes using a cascade of support vector machines. *J Neurol Sci* 2012;**322**:211–6. doi:10.1016/j.jns.2012.07.064

230 Dadar M, Maranzano J, Misquitta K, *et al.* Performance comparison of 10 different classification techniques in segmenting white matter hyperintensities in aging. *Neuroimage* 2017;**157**:233–49. doi:10.1016/j.neuroimage.2017.06.009

231 Dadar M, Maranzano J, Ducharme S, *et al.* Validation of T1w-based segmentations of white matter hyperintensity volumes in large-scale datasets of aging. *Hum Brain Mapp* 2017;**39**:1093–107. doi:10.1002/hbm.23894

232 Dadar M, Pascoal TA, Manitsirikul S, *et al.* Validation of a Regression Technique for Segmentation of White Matter Hyperintensities in Alzheimer's Disease. *IEEE Transactions on Medical Imaging* 2017;**36**:1758–68. doi:10.1109/TMI.2017.2693978

233 Cui S, Mao L, Xiong S. Brain Tumor Automatic Segmentation Using Fully Convolutional Networks. *Journal of Medical Imaging and Health Informatics* 2017;**7**:1641–7. doi:10.1166/jmihi.2017.2179

234 Cui S, Mao L, Jiang J, *et al.* Automatic Semantic Segmentation of Brain Gliomas from MRI Images Using a Deep Cascaded Neural Network. *J Healthc Eng* 2018;**2018**. doi:10.1155/2018/4940593

235 Corso JJ, Sharon E, Dube S, *et al.* Efficient multilevel brain tumor segmentation with integrated bayesian model classification. *IEEE Trans Med Imaging* 2008;**27**:629–40. doi:10.1109/TMI.2007.912817

236 Cordier N, Delingette H, Ayache N. A Patch-Based Approach for the Segmentation of Pathologies: Application to Glioma Labelling. *IEEE Trans Med Imaging* 2016;**35**:1066–76. doi:10.1109/TMI.2015.2508150

For peer review only - http://bmjopen.bmj.com/site/about/guidelines.xhtml

237 Commowick O, Maarouf A, Ferré J-C, *et al.* Diffusion MRI abnormalities detection with orientation distribution functions: A multiple sclerosis longitudinal study. *Medical Image Analysis* 2015;**22**:114–23. doi:10.1016/j.media.2015.02.005

238 Colliot O, Mansi T, Bernasconi N, *et al.* Segmentation of focal cortical dysplasia lesions on MRI using level set evolution. *Neuroimage* 2006;**32**:1621–30. doi:10.1016/j.neuroimage.2006.04.225

239 Clarke LP, Velthuizen RP, Clark M, *et al.* MRI Measurement of Brain Tumor Response: Comparison of Visual Metric and Automatic Segmentation. *Magnetic Resonance Imaging* 1998;**16**:271–9. doi:10.1016/S0730-725X(97)00302-0

240 Cheriyan MM, Michael PA, Kumar A. Blind source separation with mixture models – A hybrid approach to MR brain classification. *Magnetic Resonance Imaging* 2018;**54**:137–47. doi:10.1016/j.mri.2018.08.023

241 Chen W, Liu B, Peng S, *et al.* Computer-Aided Grading of Gliomas Combining Automatic Segmentation and Radiomics. *International Journal of Biomedical Imaging* 2018;**2018**:e2512037. doi:https://doi.org/10.1155/2018/2512037

242 Chen L, Bentley P, Rueckert D. Fully automatic acute ischemic lesion segmentation in DWI using convolutional neural networks. *Neuroimage Clin* 2017;**15**:633–43. doi:10.1016/j.nicl.2017.06.016

243 Charron O, Lallement A, Jarnet D, *et al.* Automatic detection and segmentation of brain metastases on multimodal MR images with a deep convolutional neural network. *Computers in Biology and Medicine* 2018;**95**:43–54. doi:10.1016/j.compbiomed.2018.02.004

244 Cabria I, Gondra I. MRI segmentation fusion for brain tumor detection. *Information Fusion* 2017;**36**:1–9. doi:10.1016/j.inffus.2016.10.003

245 Cabezas M, Oliver A, Valverde S, *et al.* BOOST: A supervised approach for multiple sclerosis lesion segmentation. *Journal of Neuroscience Methods* 2014;**237**:108–17. doi:10.1016/j.jneumeth.2014.08.024

246 Cabezas M, Oliver A, Roura E, *et al.* Automatic multiple sclerosis lesion detection in brain MRI by FLAIR thresholding. *Computer Methods and Programs in Biomedicine* 2014;**115**:147–61. doi:10.1016/j.cmpb.2014.04.006

247 Cabezas M, Corral JF, Oliver A, *et al.* Improved Automatic Detection of New T2 Lesions in Multiple Sclerosis Using Deformation Fields. *American Journal of Neuroradiology* 2016;**37**:1816–23. doi:10.3174/ajnr.A4829

248 Brosch T, Tang LYW, Yoo Y, *et al.* Deep 3D Convolutional Encoder Networks With Shortcuts for Multiscale Feature Integration Applied to Multiple Sclerosis Lesion Segmentation. *IEEE Transactions on Medical Imaging* 2016;**35**:1229–39. doi:10.1109/TMI.2016.2528821

249 Bowles C, Qin C, Guerrero R, *et al.* Brain lesion segmentation through image synthesis and outlier detection. *NeuroImage: Clinical* 2017;**16**:643–58. doi:10.1016/j.nicl.2017.09.003

250 Boudraa AO, Dehak SM, Zhu YM, *et al.* Automated segmentation of multiple sclerosis lesions in multispectral MR imaging using fuzzy clustering. *Comput Biol Med* 2000;**30**:23–40. doi:10.1016/s0010-4825(99)00019-0

251 Bonte S, Goethals I, Van Holen R. Machine learning based brain tumour segmentation on limited data using local texture and abnormality. *Computers in Biology and Medicine* 2018;**98**:39–47. doi:10.1016/j.compbiomed.2018.05.005

For peer review only

252 Boldsen JK, Engedal TS, Pedraza S, *et al.* Better Diffusion Segmentation in Acute Ischemic Stroke Through Automatic Tree Learning Anomaly Segmentation. *Front Neuroinform* 2018;**12**. doi:10.3389/fninf.2018.00021

253 Binczyk F, Stjelties B, Weber C, *et al.* MiMSeg - an algorithm for automated detection of tumor tissue on NMR apparent diffusion coefficient maps. *Information Sciences* 2017;**384**:235–48. doi:10.1016/j.ins.2016.07.052

254 Binaghi E, Pedoia V, Balbi S. Meningioma and peritumoral edema segmentation of preoperative MRI brain scans. *Computer Methods in Biomechanics and Biomedical Engineering: Imaging & Visualization* 2018;**6**:362–70. doi:10.1080/21681163.2016.1250108

255 Bijar A, Khayati R, Benavent AP. Increasing the Contrast of the Brain MR FLAIR Images Using Fuzzy Membership Functions and Structural Similarity Indices in Order to Segment MS Lesions. *PLOS ONE* 2013;**8**:e65469. doi:10.1371/journal.pone.0065469

256 Bijar A, Khanloo MM, Benavent AP, *et al.* Segmentation of MS lesions using entropy-based EM algorithm and Markov random fields. *Journal of Biomedical Science and Engineering* 2011;**4**:552–61. doi:10.4236/jbise.2011.48071

257 Bhanu Prakash KN, Gupta V, Jianbo H, *et al.* Automatic processing of diffusion-weighted ischemic stroke images based on divergence measures: slice and hemisphere identification, and stroke region segmentation. *Int J CARS* 2008;**3**:559–70. doi:10.1007/s11548-008-0260-3

258 Beare R, Srikanth V, Chen J, *et al.* Development and validation of morphological segmentation of age-related cerebral white matter hyperintensities. *Neuroimage* 2009;**47**:199–203. doi:10.1016/j.neuroimage.2009.03.055

259 Banerjee S, Mitra S, Uma Shankar B. Automated 3D segmentation of brain tumor using visual saliency. *Information Sciences* 2018;**424**:337–53. doi:10.1016/j.ins.2017.10.011

260 Banerjee S, Mitra S, Shankar BU, *et al.* A Novel GBM Saliency Detection Model Using Multi-Channel MRI. *PLOS ONE* 2016;**11**:e0146388. doi:10.1371/journal.pone.0146388

261 Asman AJ, Chambless LB, Thompson RC, *et al.* Out-of-atlas likelihood estimation using multi-atlas segmentation. *Med Phys* 2013;**40**. doi:10.1118/1.4794478

262 Arakeri MeghaP, Reddy GRM. Computer-aided diagnosis system for tissue characterization of brain tumor on magnetic resonance images. *SIViP* 2015;**9**:409–25. doi:10.1007/s11760-013-0456-z

263 Anbeek P, Vincken KL, Viergever MA. Automated MS-Lesion Segmentation by K-Nearest Neighbor Classification. *The MIDAS Journal* 2008;:610.

264 Anbeek P, Vincken KL, van Osch MJP, *et al.* Automatic segmentation of different-sized white matter lesions by voxel probability estimation. *Medical Image Analysis* 2004;**8**:205–15. doi:10.1016/j.media.2004.06.019

265 Amiri S, Ali Mahjoub M, Rekik I. Tree-based Ensemble Classifier Learning for Automatic Brain Glioma Segmentation. *Neurocomputing* 2018;**313**:135–42. doi:10.1016/j.neucom.2018.05.112

266 Alex V, Vaidhya K, Thirunavukkarasu S, *et al.* Semisupervised learning using denoising autoencoders for brain lesion detection and segmentation. *J Med Imaging (Bellingham)* 2017;**4**:041311. doi:10.1117/1.JMI.4.4.041311

267 Alegro M de C, Amaro Junior E, Lopes R de D. Computerized brain tumor segmentation in magnetic resonance imaging. *Einstein (São Paulo)* 2012;**10**:158–63. doi:10.1590/S1679-45082012000200008

268 AlBadawy EA, Saha A, Mazurowski MA. Deep learning for segmentation of brain tumors: Impact of cross-institutional training and testing. *Medical Physics* 2018;**45**:1150–8. doi:https://doi.org/10.1002/mp.12752

269 Al-Shaikhli SDS, Yang MY, Rosenhahn B. Brain tumor classification and segmentation using sparse coding and dictionary learning. *Biomedical Engineering / Biomedizinische Technik* 2016;**61**:413–29. doi:10.1515/bmt-2015-0071

270 Akselrod-Ballin A, Galun M, Gomori JM, *et al.* Automatic segmentation and classification of multiple sclerosis in multichannel MRI. *IEEE Trans Biomed Eng* 2009;**56**:2461–9. doi:10.1109/TBME.2008.926671

271 Ahmed B, Brodley CE, Blackmon KE, *et al.* Cortical feature analysis and machine learning improves detection of "MRI-negative" focal cortical dysplasia. *Epilepsy Behav* 2015;**48**:21–8. doi:10.1016/j.yebeh.2015.04.055

272 Admiraal-Behloul F, van den Heuvel DMJ, Olofsen H, *et al.* Fully automatic segmentation of white matter hyperintensities in MR images of the elderly. *Neuroimage* 2005;**28**:607–17. doi:10.1016/j.neuroimage.2005.06.061

273 Adler S, Wagstyl K, Gunny R, *et al.* Novel surface features for automated detection of focal cortical dysplasias in paediatric epilepsy. *Neuroimage Clin* 2016;**14**:18–27. doi:10.1016/j.nicl.2016.12.030

274 Abdulraqeb AR, Al-Haidri WA, Sushkova LT, *et al.* An Automated Method for Segmenting Brain Tumors on MRI Images. *Biomed Eng* 2017;**51**:97–101. doi:10.1007/s10527-017-9692-9

275 Abdullah BA, Younis AA, John NM. Multi-Sectional Views Textural Based SVM for MS Lesion Segmentation in Multi-Channels MRIs. *Open Biomed Eng J* 2012;**6**:56–72. doi:10.2174/1874230001206010056

276 Abd-Ellah MK, Awad AI, Khalaf AAM, *et al.* Two-phase multi-model automatic brain tumour diagnosis system from magnetic resonance images using convolutional neural networks. *EURASIP Journal on Image and Video Processing* 2018;**2018**:97. doi:10.1186/s13640-018-0332-4

277 Abbasi S, Tajeripour F. Detection of brain tumor in 3D MRI images using local binary patterns and histogram orientation gradient. *Neurocomputing* 2017;**219**:526–35. doi:10.1016/j.neucom.2016.09.051

278 Agam G, Weiss D, Soman M, *et al.* Probabilistic Brain Lesion Segmentation in DT-MRI. In: *2006 International Conference on Image Processing*. 2006. 89–92. doi:10.1109/ICIP.2006.312369

279 Agn M, Law I, Munck Af Rosenschöld P, *et al.* A generative model for segmentation of tumor and organs-at-risk for radiation therapy planning of glioblastoma patients. In: *Progress in Biomedical Optics and Imaging - Proceedings of SPIE*. SPIE 2016. doi:10.1117/12.2216814

280 Agn M, Puonti O, Rosenschöld PM af, *et al.* Brain Tumor Segmentation Using a Generative Model with an RBM Prior on Tumor Shape. In: Crimi A, Menze B, Maier O, *et al.*, eds. *Brainlesion: Glioma, Multiple Sclerosis, Stroke and Traumatic Brain Injuries*. Cham: : Springer International Publishing 2016. 168–80. doi:10.1007/978-3-319-30858-6_15

281 Akter MK, Khan SM, Azad S, *et al.* Automated brain tumor segmentation from mri data based on exploration of histogram characteristics of the cancerous hemisphere. In: *2017 IEEE Region 10 Humanitarian Technology Conference (R10-HTC)*. 2017. 815–8. doi:10.1109/R10-HTC.2017.8289080

282 Al-Shaikhli SDS, Yang MY, Rosenhahn B. Coupled Dictionary Learning for Automatic Multi-Label Brain Tumor Segmentation in Flair MRI images. In: Bebis G, Boyle R, Parvin B, *et al.*, eds. *Advances in Visual Computing*. Cham: : Springer International Publishing 2014. 489–500. doi:10.1007/978-3-319-14249-4_46

283 Alex V, Safwan KPM, Chennamsetty SS, *et al.* Generative adversarial networks for brain lesion detection. In: *Medical Imaging 2017: Image Processing*. 2017. 101330G. doi:10.1117/12.2254487

284 Amiri S, Rekik I, Mahjoub MA. Deep random forest-based learning transfer to SVM for brain tumor segmentation. In: *2016 2nd International Conference on Advanced Technologies for Signal and Image Processing (ATSIP)*. 2016. 297–302. doi:10.1109/ATSIP.2016.7523095

285 Amiri S, Mahjoub M, Rekik I. Bayesian Network and Structured Random Forest Cooperative Deep Learning For Automatic Multi-label Brain Tumor Segmentation. In: *ICAART*. 2018. 183–90.

286 Ananda RS, Thomas T. Automatic segmentation framework for primary tumors from brain MRIs using morphological filtering techniques. In: *2012 5th International Conference on BioMedical Engineering and Informatics*. 2012. 238–42. doi:10.1109/BMEI.2012.6512995

287 Anwar SM, Yousaf S, Majid M. Brain tumor segmentation on Multimodal MRI scans using EMAP Algorithm. In: *2018 40th Annual International Conference of the IEEE Engineering in Medicine and Biology Society (EMBC)*. 2018. 550–3. doi:10.1109/EMBC.2018.8512304

288 Assaf Hoogi, Andrew Lee, Vivek Bharadwa, *et al.* Multimodal Brain Tumor Segmentation (BRATS) using Sparse Coding and 2-layer Neural Network. In: *Proceedings of the Multimodal Brain Tumor Image Segmentation Challenge*. 2015. 34.

289 Baid U, Talbar S, Talbar SN. Novel approach for brain tumor segmentation with non negative matrix factorization. In: *2017 International Conference on Innovations in Electronics, Signal Processing and Communication (IESC)*. 2017. 101–5. doi:10.1109/IESPC.2017.8071873

290 Bauer S, Gratz PP, Gralla J, *et al.* Towards automatic MRI volumetry for treatment selection in acute ischemic stroke patients. In: *Annual International Conference of the IEEE Engineering in Medicine and Biology Society.* 2014. 1521–4. doi:10.1109/EMBC.2014.6943891

291 Bauer S, Nolte L-P, Reyes M. Fully Automatic Segmentation of Brain Tumor Images Using Support Vector Machine Classification in Combination with Hierarchical Conditional Random Field Regularization. In: Fichtinger G, Martel A, Peters T, eds. *Medical Image Computing and Computer-Assisted Intervention – MICCAI 2011*. Berlin, Heidelberg: : Springer 2011. 354–61. doi:10.1007/978-3-642-23626-6_44

292 Behzadfar N, Soltanian-Zadeh H. Automatic segmentation of brain tumors in magnetic resonance images. In: *Proceedings of 2012 IEEE-EMBS International Conference on Biomedical and Health Informatics*. 2012. 329–32. doi:10.1109/BHI.2012.6211580

293 Ben Salah M, Diaz I, Greiner R, *et al.* Fully Automated Brain Tumor Segmentation Using Two MRI Modalities. In: Bebis G, Boyle R, Parvin B, *et al.*, eds. *Advances in Visual Computing*. Berlin, Heidelberg: : Springer 2013. 30–9. doi:10.1007/978-3-642-41914-0_4

294 Benson CC, Deepa V, Lajish VL, *et al.* Brain tumor segmentation from MR brain images using improved fuzzy c-means clustering and watershed algorithm. In: *2016 International Conference on Advances in Computing, Communications and Informatics (ICACCI)*. 2016. 187–92. doi:10.1109/ICACCI.2016.7732045

295 Bento M, de Souza R, Lotufo R, *et al.* WMH Segmentation Challenge: A Texture-Based Classification Approach. In: Crimi A, Bakas S, Kuijf H, *et al.*, eds. *Brainlesion: Glioma, Multiple Sclerosis, Stroke and Traumatic Brain Injuries*. Cham: : Springer International Publishing 2018. 489–500. doi:10.1007/978-3-319-75238-9_41

296 Bento M, Sym Y, Frayne R, *et al.* Probabilistic Segmentation of Brain White Matter Lesions Using Texture-Based Classification. In: Karray F, Campilho A, Cheriet F, eds. *Image Analysis and Recognition*. Cham: : Springer International Publishing 2017. 71–8. doi:10.1007/978-3-319-59876-5_9

297 Bharath HN, Colleman S, Sima DM, *et al.* Tumor Segmentation from Multimodal MRI Using Random Forest with Superpixel and Tensor Based Feature Extraction. In: Crimi A, Bakas S, Kuijf H, *et al.*, eds. *Brainlesion: Glioma, Multiple Sclerosis, Stroke and Traumatic Brain Injuries*. Cham: : Springer International Publishing 2018. 463–73. doi:10.1007/978-3-319-75238-9_39

298 Bhattacharya D, Sinha N. An improved approach of high graded glioma segmentation using sparse autoencoder and fuzzy c-means clustering from multi-modal MR images. In: *Medical Imaging 2018: Biomedical Applications in Molecular, Structural, and Functional Imaging*. International Society for Optics and Photonics 2018. 1057813. doi:10.1117/12.2293044

299 Bianchi A, Miller JV, Tan ET, *et al.* Brain tumor segmentation with symmetric texture and symmetric intensity-based decision forests. In: *Proceedings / IEEE International Symposium on Biomedical Imaging: from nano to macro.* 2013. 748–51. doi:10.1109/ISBI.2013.6556583

300 Bijar A, Khayati R. Segmentation of MS lesions using Active Contour Model, Adaptive Mixtures Method and MRF model. In: *2011 7th International Symposium on Image and Signal Processing and Analysis (ISPA)*. 2011. 159–64.

301 Bilotta E, Cerasa A, Pantano P, *et al.* A CNN Based Algorithm for the Automated Segmentation of Multiple Sclerosis Lesions. In: Di Chio C, Cagnoni S, Cotta C, *et al.*, eds. *Applications of Evolutionary Computation*. Berlin, Heidelberg: : Springer 2010. 211–20. doi:10.1007/978-3-642-12239-2_22

302 Birenbaum A, Greenspan H. Longitudinal Multiple Sclerosis Lesion Segmentation Using Multi-view Convolutional Neural Networks. In: Carneiro G, Mateus D, Peter L, *et al.*, eds. *Deep Learning and Data Labeling for Medical Applications*. Cham: : Springer International Publishing 2016. 58–67. doi:10.1007/978-3-319-46976-8_7

303 Bougacha A, Boughariou J, Slima MB, *et al.* Comparative study of supervised and unsupervised classification methods: Application to automatic MRI glioma brain tumors segmentation. In: *2018 4th International Conference on Advanced Technologies for Signal and Image Processing (ATSIP)*. 2018. 1–5. doi:10.1109/ATSIP.2018.8364463

304 Boussaid H, Kokkinos I, Paragios N. Rapid Mode Estimation for 3D Brain MRI Tumor Segmentation. In: Heyden A, Kahl F, Olsson C, *et al.*, eds. *Energy Minimization Methods in Computer Vision and Pattern Recognition*. Berlin, Heidelberg: : Springer 2013. 1–11. doi:10.1007/978-3-642-40395-8_1

305 Cabezas M, Oliver A, Freixenet J, *et al.* A Supervised Approach for Multiple Sclerosis Lesion Segmentation Using Context Features and an Outlier Map. In: Sanches JM, Micó L, Cardoso JS, eds. *Pattern Recognition and Image Analysis*. Berlin, Heidelberg: : Springer 2013. 782–9. doi:10.1007/978-3-642-38628-2_93

306 Cai H, Verma R, Ou Y, *et al.* Probabilistic segmentation of brain tumours based on multimodality magnetic resonance images. In: *2007 4th IEEE International Symposium on Biomedical Imaging: From Nano to Macro*. 2007. 600–3. doi:10.1109/ISBI.2007.356923

307 Chang P. Fully convolutional neural networks with hyperlocal features for brain tumor segmentation. 2016. 118. doi:10.1007/978-3-319-55524-9_11

308 Chen L, Wu Y, DSouza AM, *et al.* MRI tumor segmentation with densely connected 3D CNN. In: *Medical Imaging 2018: Image Processing*. International Society for Optics and Photonics 2018. 105741F. doi:10.1117/12.2293394

309 Chen W, Qiao X, Liu B, *et al.* Automatic brain tumor segmentation based on features of separated local square. In: *2017 Chinese Automation Congress (CAC)*. 2017. 6489–93. doi:10.1109/CAC.2017.8243946

310 Corso JJ, Sharon E, Yuille A. Multilevel Segmentation and Integrated Bayesian Model Classification with an Application to Brain Tumor Segmentation. In: Larsen R, Nielsen M, Sporring J, eds. *Medical Image Computing and Computer-Assisted Intervention – MICCAI 2006*. Berlin, Heidelberg: : Springer 2006. 790–8. doi:10.1007/11866763_97

311 Corso JJ, Yuille A, Sicotte NL, *et al.* Detection and segmentation of pathological structures by the extended graph-shifts algorithm. In: *International Conference on Medical Image Computing and Computer-Assisted Intervention*. 2007. 985–93. doi:10.1007/978-3-540-75757-3_119

312 Dalca AV, Sridharan R, Cloonan L, *et al.* Segmentation of Cerebrovascular Pathologies in Stroke Patients with Spatial and Shape Priors. In: *International Conference on Medical Image Computing and Computer-Assisted Intervention*. 2014. 773–80.https://www.ncbi.nlm.nih.gov/pmc/articles/PMC4260817/ (accessed 15 Nov 2020).

313 Derntl A, Plant C, Gruber P, *et al.* Stroke Lesion Segmentation Using a Probabilistic Atlas of Cerebral Vascular Territories. In: Crimi A, Menze B, Maier O, *et al.*, eds. *Brainlesion: Glioma, Multiple Sclerosis, Stroke and Traumatic Brain Injuries*. Cham: : Springer International Publishing 2016. 21–32. doi:10.1007/978-3-319-30858-6_3

314 Deshpande H, Maurel P, Barillot C. Adaptive dictionary learning for competitive classification of multiple sclerosis lesions. In: *2015 IEEE 12th International Symposium on Biomedical Imaging (ISBI)*. 2015. 136–9. doi:10.1109/ISBI.2015.7163834

315 Diaz I, Boulanger P, Greiner R, *et al.* An automatic brain tumor segmentation tool. In: *Annual International Conference of the IEEE Engineering in Medicine and Biology Society.* 2013. 3339–42. doi:10.1109/EMBC.2013.6610256

316 Dong H, Yang G, Liu F, *et al.* Automatic Brain Tumor Detection and Segmentation Using U-Net Based Fully Convolutional Networks. In: Valdés Hernández M, González-Castro V, eds. *Medical Image Understanding and Analysis*. Cham: : Springer International Publishing 2017. 506–17. doi:10.1007/978-3-319-60964-5_44

317 Doyle S, Forbes F, Jaillard A, *et al.* Sub-acute and Chronic Ischemic Stroke Lesion MRI Segmentation. In: Crimi A, Bakas S, Kuijf H, *et al.*, eds. *Brainlesion: Glioma, Multiple Sclerosis, Stroke and Traumatic Brain Injuries*. Cham: : Springer International Publishing 2018. 111–22. doi:10.1007/978-3-319-75238-9_10

318 Dvorak P, Bartusek K, Gescheidtova E. Automatic extraction of pathological area in 2D MR brain scan. In: *PIERS Proceedings*. 2014. 1885–9.

319 Dvořák P, Menze B. Local Structure Prediction with Convolutional Neural Networks for Multimodal Brain Tumor Segmentation. In: Menze B, Langs G, Montillo A, *et al.*, eds. *Medical Computer Vision: Algorithms for Big Data*. Cham: : Springer International Publishing 2016. 59–71. doi:10.1007/978-3-319-42016-5_6

320 El-Khamy SE, Sadek RA, El-Khoreby MA. An efficient brain mass detection with adaptive clustered based fuzzy C-mean and thresholding. In: *2015 IEEE International Conference on Signal and Image Processing Applications (ICSIPA)*. 2015. 429–33. doi:10.1109/ICSIPA.2015.7412229

321 Elliott C, Francis SJ, Arnold DL, *et al.* Bayesian Classification of Multiple Sclerosis Lesions in Longitudinal MRI Using Subtraction Images. In: Jiang T, Navab N, Pluim JPW, *et al.*, eds. *Medical Image Computing and Computer-Assisted Intervention – MICCAI 2010*. Berlin, Heidelberg: : Springer 2010. 290–7. doi:10.1007/978-3-642-15745-5_36

For peer review only

322 Ellwaa, A., Hussein, A., AlNaggar, E., *et al.* Brain Tumor Segmantation Using Random Forest Trained on Iteratively Selected Patients. 2016. 129–37.https://www.springerprofessional.de/en/brain-tumor-segmantation-using-random-forest-trained-on-iterativ/12216552 (accessed 16 Nov 2020).

323 Feng C, Zhao D, Huang M. Segmentation of Ischemic Stroke Lesions in Multi-spectral MR Images Using Weighting Suppressed FCM and Three Phase Level Set. In: Crimi A, Menze B, Maier O, *et al.*, eds. *Brainlesion: Glioma, Multiple Sclerosis, Stroke and Traumatic Brain Injuries*. Cham: : Springer International Publishing 2016. 233–45. doi:10.1007/978-3-319-30858-6_20

324 Ferrari RJ, M.d XW, M.d YZ, *et al.* Segmentation of multiple sclerosis lesions using support vector machines. In: *Medical Imaging 2003: Image Processing*. International Society for Optics and Photonics 2003. 16–26. doi:10.1117/12.481377

325 Festa J, Pereira S, Mariz J, *et al.* Automatic brain tumor segmentation of multi-sequence mr images using random decision forests. In: *Proceedings of the MICCAI grand challenge on MR brain image segmentation (MRBrainS'13)*. 2013.

326 Fiçici CÖ, Eroğul O, Telatar Z. Fully Automated Brain Tumor Segmentation and Volume Estimation Based on Symmetry Analysis in MR Images. In: Badnjevic A, ed. *CMBEBIH 2017*. Singapore: : Springer 2017. 53–60. doi:10.1007/978-981-10-4166-2_9

327 Folgoc LL, Nori AV, Alvarez-Valle J, *et al.* Segmentation of brain tumors via cascades of lifted decision forests. In: *Proceedings MICCAI-BRATS Workshop*. 2016.

328 Folgoc L, Nori A, Ancha S, *et al.* Lifted Auto-Context Forests for Brain Tumour Segmentation. 2016. 171–83. doi:10.1007/978-3-319-55524-9_17

329 García-Lorenzo D, Lecoeur J, Arnold DL, *et al.* Multiple Sclerosis Lesion Segmentation Using an Automatic Multimodal Graph Cuts. In: Yang G-Z, Hawkes D, Rueckert D, *et al.*, eds. *Medical Image Computing and Computer-Assisted Intervention – MICCAI 2009*. Berlin, Heidelberg: : Springer 2009. 584–91. doi:10.1007/978-3-642-04271-3_71

330 García-Lorenzo D, Prima S, Morrissey S, *et al.* A robust Expectation-Maximization algorithm for Multiple Sclerosis lesion segmentation. In: *Proceeding of MICCAI Workshop*. 2008.

331 Geremia E, Menze BH, Ayache N. Spatial Decision Forests for Glioma Segmentation in Multi-Channel MR Images. 2012. https://hal.inria.fr/hal-00813827 (accessed 15 Nov 2020).

332 Goel S, Sehgal A, Mangipudi P, *et al.* Brain tumor segmentation in multispectral MR images. In: *2017 4th International Conference on Signal Processing and Integrated Networks (SPIN)*. 2017. 1–4. doi:10.1109/SPIN.2017.8049904

333 Gondra I, Cabria I. Automated segmentation of brain tumors in MRI using potential field clustering. In: *IEEE EUROCON 2015 - International Conference on Computer as a Tool (EUROCON)*. 2015. 1–6. doi:10.1109/EUROCON.2015.7313670

334 Götz M, Weber C, Blöcher J, *et al.* Extremely randomized trees based brain tumor segmentation. In: *Proceeding of BRATS challenge-MICCAI*. 2014. 006–11.

335 Hadjiprocopis A, Tofts P. An automatic lesion segmentation method for fast spin echo magnetic resonance images using an ensemble of neural networks. In: *2003 IEEE XIII Workshop on Neural Networks for Signal Processing (IEEE Cat. No.03TH8718)*. 2003. 709–18. doi:10.1109/NNSP.2003.1318070

For peer review only

336 Haeck T, Maes F, Suetens P. Automated Model-Based Segmentation of Brain Tumors in MR Images. In: *Proceedings BraTS Challenge 2015*. 2015. 25–8.

337 Haeck T, Maes F, Suetens P. ISLES Challenge 2015: Automated Model-Based Segmentation of Ischemic Stroke in MR Images. In: Crimi A, Menze B, Maier O, *et al.*, eds. *Brainlesion: Glioma, Multiple Sclerosis, Stroke and Traumatic Brain Injuries*. Cham: : Springer International Publishing 2016. 246–53. doi:10.1007/978-3-319-30858-6_21

338 Harmouche R, Collins L, Arnold D, *et al.* Bayesian MS Lesion Classification Modeling Regional and Local Spatial Information. In: *18th International Conference on Pattern Recognition (ICPR'06)*. 2006. 984–7. doi:10.1109/ICPR.2006.318

339 Havaei M, Dutil F, Pal C, *et al.* A Convolutional Neural Network Approach to Brain Tumor Segmentation. In: Crimi A, Menze B, Maier O, *et al.*, eds. *Brainlesion: Glioma, Multiple Sclerosis, Stroke and Traumatic Brain Injuries*. Cham: : Springer International Publishing 2016. 195–208. doi:10.1007/978-3-319-30858-6_17

340 Hevia-Montiel N, Jiménez-Alaniz JR, Medina-Bañuelos V, *et al.* Robust nonparametric segmentation of infarct lesion from diffusion-weighted MR images. In: *Annual International Conference of the IEEE Engineering in Medicine and Biology Society.* 2007. 2102–5. doi:10.1109/IEMBS.2007.4352736

341 Hooda H, Verma OP, Singhal T. Brain tumor segmentation: A performance analysis using K-Means, Fuzzy C-Means and Region growing algorithm. In: *2014 IEEE International Conference on Advanced Communications, Control and Computing Technologies*. 2014. 1621–6. doi:10.1109/ICACCCT.2014.7019383

342 Hu Y, Xia Y. 3D Deep Neural Network-Based Brain Tumor Segmentation Using Multimodality Magnetic Resonance Sequences. In: Crimi A, Bakas S, Kuijf H, *et al.*, eds. *Brainlesion: Glioma, Multiple Sclerosis, Stroke and Traumatic Brain Injuries*. Cham: : Springer International Publishing 2018. 423–34. doi:10.1007/978-3-319-75238-9_36

343 Huang J, Yang F, Chen W. Brain Tumor Segmentation Based on Texture, Intensity, and Edge. In: Long M, ed. *World Congress on Medical Physics and Biomedical Engineering May 26-31, 2012, Beijing, China*. Berlin, Heidelberg: : Springer 2013. 991–4. doi:10.1007/978-3-642-29305-4_260

344 Hussain S, Anwar SM, Majid M. Brain tumor segmentation using cascaded deep convolutional neural network. In: *2017 39th Annual International Conference of the IEEE Engineering in Medicine and Biology Society (EMBC)*. 2017. 1998–2001. doi:10.1109/EMBC.2017.8037243

345 Iftekharuddin KM, Islam MA, Shaik J, *et al.* Automatic brain tumor detection in MRI: methodology and statistical validation. In: *Medical Imaging 2005: Image Processing*. International Society for Optics and Photonics 2005. 2012–22. doi:10.1117/12.595931

346 Ilunga-Mbuyamba E, Avina-Cervantes JG, Lindner D, *et al.* Automatic brain tumor tissue detection based on hierarchical centroid shape descriptor in Tl-weighted MR images. In: *2016 International Conference on Electronics, Communications and Computers (CONIELECOMP)*. 2016. 62–7. doi:10.1109/CONIELECOMP.2016.7438553

347 Jain S, Ribbens A, Sima DM, *et al.* Unsupervised Framework for Consistent Longitudinal MS Lesion Segmentation. In: Müller H, Kelm BM, Arbel T, *et al.*, eds. *Medical Computer Vision and Bayesian and Graphical Models for Biomedical Imaging*. Cham: : Springer International Publishing 2017. 208–19. doi:10.1007/978-3-319-61188-4_19

348 Jerman T, Galimzianova A, Pernuš F, *et al.* Combining Unsupervised and Supervised Methods for Lesion Segmentation. In: Crimi A, Menze B, Maier O, *et al.*, eds. *Brainlesion: Glioma, Multiple Sclerosis, Stroke*

and Traumatic Brain Injuries. Cham: : Springer International Publishing 2016. 45–56. doi:10.1007/978-3-319-30858-6_5

349 Jesson A, Arbel T. Hierarchical MRF and random forest segmentation of MS lesions and healthy tissue in brain MRI. In: *Proceedings of the 2015 longitudinal multiple sclerosis lesion segmentation challenge*. 2015. 3.

350 Jiang Y, Hou J, Xiao X, *et al.* A Brain Tumor Segmentation New Method Based on Statistical Thresholding and Multiscale CNN. In: Huang D-S, Gromiha MM, Han K, *et al.*, eds. *Intelligent Computing Methodologies*. Cham: : Springer International Publishing 2018. 235–45. doi:10.1007/978-3-319-95957-3_26

351 Jin D, Xu Z, Harrison AP, *et al.* White matter hyperintensity segmentation from T1 and FLAIR images using fully convolutional neural networks enhanced with residual connections. In: *2018 IEEE 15th International Symposium on Biomedical Imaging (ISBI 2018)*. 2018. 1060–4. doi:10.1109/ISBI.2018.8363754

352 Jog A, Carass A, Pham DL, *et al.* Multi-Output Decision Trees for Lesion Segmentation in Multiple Sclerosis. In: *Proceedings of SPIE the International Society for Optical Engineering*. 2015. doi:10.1117/12.2082157

353 Kanas VG, Zacharaki EI, Dermatas E, *et al.* Combining Outlier Detection with Random Walker for Automatic Brain Tumor Segmentation. In: Iliadis L, Maglogiannis I, Papadopoulos H, *et al.*, eds. *Artificial Intelligence Applications and Innovations*. Berlin, Heidelberg: : Springer 2012. 26–35. doi:10.1007/978-3-642-33412-2_3

354 Kapás Z, Lefkovits L, Iclănzan D, *et al.* Automatic Brain Tumor Segmentation in Multispectral MRI Volumes Using a Random Forest Approach. In: Paul M, Hitoshi C, Huang Q, eds. *Image and Video Technology*. Cham: : Springer International Publishing 2018. 137–49. doi:10.1007/978-3-319-75786-5_12

355 Karimaghaloo Z, Arnold DL, Collins DL, *et al.* Hierarchical Conditional Random Fields for Detection of Gad-Enhancing Lesions in Multiple Sclerosis. In: Ayache N, Delingette H, Golland P, *et al.*, eds. *Medical Image Computing and Computer-Assisted Intervention – MICCAI 2012*. Berlin, Heidelberg: : Springer 2012. 379–86. doi:10.1007/978-3-642-33418-4_47

356 Karimaghaloo Z, Rivaz H, Arnold DL, *et al.* Adaptive Voxel, Texture and Temporal Conditional Random Fields for Detection of Gad-Enhancing Multiple Sclerosis Lesions in Brain MRI. In: Mori K, Sakuma I, Sato Y, *et al.*, eds. *Medical Image Computing and Computer-Assisted Intervention – MICCAI 2013*. Berlin, Heidelberg: : Springer 2013. 543–50. doi:10.1007/978-3-642-40760-4_68

357 Karpate Y, Commowick O, Barillot C. Probabilistic one class learning for automatic detection of multiple sclerosis lesions. In: *2015 IEEE 12th International Symposium on Biomedical Imaging (ISBI)*. 2015. 486–9. doi:10.1109/ISBI.2015.7163917

358 Khademi A, Moody AR. Multiscale partial volume estimation for segmentation of white matter lesions using flair MRI. In: *2015 IEEE 12th International Symposium on Biomedical Imaging (ISBI)*. 2015. 568–71. doi:10.1109/ISBI.2015.7163937

359 Khotanlou H, Colliot O, Bloch I. Automatic Brain Tumor Segmentation Using Symmetry Analysis and Deformable Models. In: *Advances in Pattern Recognition*. WORLD SCIENTIFIC 2006. 198–202. doi:10.1142/9789812772381_0032

360 Knight J, Khademi A. MS Lesion Segmentation Using FLAIR MRI Only. In: *Proceedings of the 1st MICCAI Challenge on Multiple Sclerosis Lesions Segmentation Challenge Using a Data Management and Processing Infrastructure-MICCAI-MSSEG*. 2016. 21–8.

361 Kroon D-J, van Oort E, Slump K. Multiple Sclerosis Detection in Multispectral Magnetic Resonance Images with Principal Components Analysis. In: *Grand Challenge Work.: Mult. Scler. Lesion Segm. Challenge*. 2008. 1–14.http://hdl.handle.net/10380/1441 (accessed 15 Nov 2020).

362 Kuijf HJ, Tax CMW, Zaanen LK, *et al.* The Added Value of Diffusion Tensor Imaging for Automated White Matter Hyperintensity Segmentation. In: O'Donnell L, Nedjati-Gilani G, Rathi Y, *et al.*, eds. *Computational Diffusion MRI*. Cham: : Springer International Publishing 2014. 45–53. doi:10.1007/978-3-319-11182-7_5

363 Kumar SVA, Harish BS, Aradhya VNM. A picture fuzzy clustering approach for brain tumor segmentation. In: *2016 Second International Conference on Cognitive Computing and Information Processing (CCIP)*. 2016. 1–6. doi:10.1109/CCIP.2016.7802852

364 Kwon D, Akbari H, Da X, *et al.* Multimodal Brain Tumor Image Segmentation Using GLISTR. In: *MICCAI brain tumor segmentation (BraTS) challenge manuscripts*. 2014.

365 Latif G, Butt MM, Khan AH, *et al.* Automatic Multimodal Brain Image Classification Using MLP and 3D Glioma Tumor Reconstruction. In: *2017 9th IEEE-GCC Conference and Exhibition (GCCCE)*. 2017. 1–9. doi:10.1109/IEEEGCC.2017.8448135

366 Lefkovits L, Lefkovits S, Szilágyi L. Brain Tumor Segmentation with Optimized Random Forest. In: Crimi A, Menze B, Maier O, *et al.*, eds. *Brainlesion: Glioma, Multiple Sclerosis, Stroke and Traumatic Brain Injuries*. Cham: : Springer International Publishing 2016. 88–99. doi:10.1007/978-3-319-55524-9_9

367 Li W, Tian J. Automatic segmentation of brain infarction in diffusion-weighted MR images. In: *Medical Imaging 2003: Image Processing*. International Society for Optics and Photonics 2003. 1531–42. doi:10.1117/12.481350

368 Li W, Tian J, Dai J. Automatic segmentation of cerebral ischemic lesions from diffusion tensor MR images. In: *Medical Imaging 2004: Image Processing*. International Society for Optics and Photonics 2004. 1640–9. doi:10.1117/12.536007

369 Li Y, Dou Q, Yu J, *et al.* Automatic Brain Tumor Segmentation from MR Images via a Multimodal Sparse Coding Based Probabilistic Model. In: *2015 International Workshop on Pattern Recognition in NeuroImaging*. 2015. 41–4. doi:10.1109/PRNI.2015.18

370 Liu J, Smith CD, Chebrolu H. Automatic Multiple Sclerosis detection based on integrated square estimation. In: *2009 IEEE Computer Society Conference on Computer Vision and Pattern Recognition Workshops*. 2009. 31–8. doi:10.1109/CVPRW.2009.5204351

371 Liu R, Cheng J, Zhu X, *et al.* Multi-modal Brain Tumor Segmentation Based on Self-organizing Active Contour Model. In: Tan T, Li X, Chen X, *et al.*, eds. *Pattern Recognition*. Singapore: : Springer 2016. 486–98. doi:10.1007/978-981-10-3005-5_40

372 López-Zorrilla A, de Velasco-Vázquez M, Serradilla-Casado O, *et al.* Brain White Matter Lesion Segmentation with 2D/3D CNN. In: Ferrández Vicente JM, Álvarez-Sánchez JR, de la Paz López F, *et al.*, eds. *Natural and Artificial Computation for Biomedicine and Neuroscience*. Cham: : Springer International Publishing 2017. 394–403. doi:10.1007/978-3-319-59740-9_39

373 Lyksborg M, Puonti O, Agn M, *et al.* An Ensemble of 2D Convolutional Neural Networks for Tumor Segmentation. In: Paulsen RR, Pedersen KS, eds. *Image Analysis*. Cham: : Springer International Publishing 2015. 201–11. doi:10.1007/978-3-319-19665-7_17

374 Mahmood Q, Basit A. Automatic Ischemic Stroke Lesion Segmentation in Multi-spectral MRI Images Using Random Forests Classifier. In: Crimi A, Menze B, Maier O, *et al.*, eds. *Brainlesion: Glioma, Multiple*

Sclerosis, Stroke and Traumatic Brain Injuries. Cham: : Springer International Publishing 2016. 266–74. doi:10.1007/978-3-319-30858-6_23

375 Mahmood Q, Basit A. Prediction of Ischemic Stroke Lesion and Clinical Outcome in Multi-modal MRI Images Using Random Forests. In: Crimi A, Menze B, Maier O, *et al.*, eds. *Brainlesion: Glioma, Multiple Sclerosis, Stroke and Traumatic Brain Injuries*. Cham: : Springer International Publishing 2016. 244–55. doi:10.1007/978-3-319-55524-9_23

376 Maier O, Wilms M, Handels H. Image Features for Brain Lesion Segmentation Using Random Forests. In: Crimi A, Menze B, Maier O, *et al.*, eds. *Proc. 2015 Longitudinal Multiple Sclerosis Lesion Segmentation Challenge*. Cham: : Springer International Publishing 2015. 119–30. doi:10.1007/978-3-319-30858-6_11

377 McKinley R, Häni L, Wiest R, *et al.* Segmenting the Ischemic Penumbra: A Decision Forest Approach with Automatic Threshold Finding. In: Crimi A, Menze B, Maier O, *et al.*, eds. *Brainlesion: Glioma, Multiple Sclerosis, Stroke and Traumatic Brain Injuries*. Cham: : Springer International Publishing 2015. 275–83. doi:10.1007/978-3-319-30858-6_24

378 Mechrez R, Goldberger J, Greenspan H. MS lesion segmentation using a multi-channel patch-based approach with spatial consistency. In: *Medical Imaging 2015: Image Processing*. International Society for Optics and Photonics 2015. 94130O. doi:10.1117/12.2082558

379 Mehmood I, Baik R, Baik SW. Automatic Segmentation of Region of Interests in MR Images Using Saliency Information and Active Contours. In: Kim KJ, Chung K-Y, eds. *IT Convergence and Security 2012*. Dordrecht: : Springer Netherlands 2012. 537–44. doi:10.1007/978-94-007-5860-5_64

380 Meier R, Karamitsou V, Habegger S, *et al.* Parameter Learning for CRF-Based Tissue Segmentation of Brain Tumors. In: Crimi A, Menze B, Maier O, *et al.*, eds. *Brainlesion: Glioma, Multiple Sclerosis, Stroke and Traumatic Brain Injuries*. Cham: : Springer International Publishing 2016. 156–67. doi:10.1007/978-3-319-30858-6_14

381 Meier R, Knecht U, Wiest R, *et al.* CRF-Based Brain Tumor Segmentation: Alleviating the Shrinking Bias. In: Crimi A, Menze B, Maier O, *et al.*, eds. *Brainlesion: Glioma, Multiple Sclerosis, Stroke and Traumatic Brain Injuries*. Cham: : Springer International Publishing 2016. 100–7. doi:10.1007/978-3-319-55524-9_10

382 Mengqiao W, Jie Y, Yilei C, *et al.* The multimodal brain tumor image segmentation based on convolutional neural networks. In: *2017 2nd IEEE International Conference on Computational Intelligence and Applications (ICCIA)*. 2017. 336–9. doi:10.1109/CIAPP.2017.8167234

383 Mitra J, Bourgeat P, Fripp J, *et al.* Classification Forests and Markov Random Field to Segment Chronic Ischemic Infarcts from Multimodal MRI. In: Shen L, Liu T, Yap P-T, *et al.*, eds. *Multimodal Brain Image Analysis*. Cham: : Springer International Publishing 2013. 107–18. doi:10.1007/978-3-319-02126-3_11

384 Morra J, Tu Z, Toga A, *et al.* Automatic Segmentation of MS Lesions Using a Contextual Model for the MICCAI Grand Challenge. In: *Grand Challenge Work.: Mult. Scler. Lesion Segm. Challenge*. 2008. 1–7.

385 Mote SR, Baid UR, Talbar SN. Non-negative matrix factorization and self-organizing map for brain tumor segmentation. In: *2017 International Conference on Wireless Communications, Signal Processing and Networking (WiSPNET)*. 2017. 1133–7. doi:10.1109/WiSPNET.2017.8299940

386 Muda AF, Saad NM, Waeleh N, *et al.* Integration of Fuzzy C-Means with Correlation Template and Active Contour for Brain Lesion Segmentation in Diffusion-Weighted MRI. In: *2015 3rd International Conference on Artificial Intelligence, Modelling and Simulation (AIMS)*. 2015. 268–73. doi:10.1109/AIMS.2015.88

387 Müller S, Weickert J, Graf N. Automatic brain tumor segmentation with a fast Mumford-Shah algorithm. In: *Medical Imaging 2016: Image Processing*. International Society for Optics and Photonics 2016. 97842S. doi:10.1117/12.2214552

388 Nabizadeh N, Dorodchi M, Kubat M. Automatic tumor lesion detection and segmentation using modified winnow algorithm. In: *2015 IEEE 12th International Symposium on Biomedical Imaging (ISBI)*. 2015. 71–4. doi:10.1109/ISBI.2015.7163819

389 Oliveira GC, Varoto R, Jr AC. Brain Tumor Segmentation in Magnetic Resonance Images using Genetic Algorithm Clustering and AdaBoost Classifier. In: *BIOIMAGING*. 2020. 77–82.https://www.scitepress.org/PublicationsDetail.aspx?ID=y+ZZXSvPyrc=&t=1 (accessed 15 Nov 2020).

390 Osman AFI. Automated Brain Tumor Segmentation on Magnetic Resonance Images and Patient's Overall Survival Prediction Using Support Vector Machines. In: Crimi A, Bakas S, Kuijf H, *et al.*, eds. *Brainlesion: Glioma, Multiple Sclerosis, Stroke and Traumatic Brain Injuries*. Cham: : Springer International Publishing 2018. 435–49. doi:10.1007/978-3-319-75238-9_37

391 Pandian B, Boyle J, Orringer DA. Multimodal tumor segmentation with 3D convolutional neural networks. In: *Proceedings of the MICCAI Challenge on Multimodal Brain Tumor Image Segmentation (BRATS) 2016*. 2016.

392 Parisot S, Duffau H, Chemouny S, *et al.* Graph-based detection, segmentation characterization of brain tumors. In: *2012 IEEE Conference on Computer Vision and Pattern Recognition*. 2012. 988–95. doi:10.1109/CVPR.2012.6247775

393 Parisot S, Duffau H, Chemouny S, *et al.* Joint Tumor Segmentation and Dense Deformable Registration of Brain MR Images. In: Ayache N, Delingette H, Golland P, *et al.*, eds. *Medical Image Computing and Computer-Assisted Intervention – MICCAI 2012*. Berlin, Heidelberg: : Springer 2012. 651–8. doi:10.1007/978-3-642-33418-4_80

394 Patricia Buendia, Thomas Taylor, Michael Ryan, *et al.* A grouping artificial immune network for segmentation of tumor images. In: *Proceedings of the MICCAI Challenge on Multimodal Brain Tumor Image Segmentation (BRATS) 2013*. 2013.

395 Pedoia V, Balbi S, Binaghi E. Fully Automatic Brain Tumor Segmentation by Using Competitive EM and Graph Cut. In: Murino V, Puppo E, eds. *Image Analysis and Processing — ICIAP 2015*. Cham: : Springer International Publishing 2015. 568–78. doi:10.1007/978-3-319-23231-7_51

396 Pereira S, Oliveira A, Alves V, *et al.* On hierarchical brain tumor segmentation in MRI using fully convolutional neural networks: A preliminary study. In: *2017 IEEE 5th Portuguese Meeting on Bioengineering (ENBENG)*. 2017. 1–4. doi:10.1109/ENBENG.2017.7889452

397 Pinto A, Pereira S, Correia H, *et al.* Brain Tumour Segmentation based on Extremely Randomized Forest with high-level features. In: *2015 37th Annual International Conference of the IEEE Engineering in Medicine and Biology Society (EMBC)*. 2015. 3037–40. doi:10.1109/EMBC.2015.7319032

398 Pinto A, Pereira S, Dinis H, *et al.* Random decision forests for automatic brain tumor segmentation on multi-modal MRI images. In: *2015 IEEE 4th Portuguese Meeting on Bioengineering (ENBENG)*. 2015. 1–5. doi:10.1109/ENBENG.2015.7088842

399 Pourreza R, Zhuge Y, Ning H, *et al.* Brain Tumor Segmentation in MRI Scans Using Deeply-Supervised Neural Networks. In: Crimi A, Bakas S, Kuijf H, *et al.*, eds. *Brainlesion: Glioma, Multiple Sclerosis, Stroke and Traumatic Brain Injuries*. Cham: : Springer International Publishing 2018. 320–31. doi:10.1007/978-3-319-75238-9_28

400 Puonti O, Van Leemput K. Simultaneous Whole-Brain Segmentation and White Matter Lesion Detection Using Contrast-Adaptive Probabilistic Models. In: Crimi A, Menze B, Maier O, *et al.*, eds. *Brainlesion: Glioma, Multiple Sclerosis, Stroke and Traumatic Brain Injuries*. Cham: : Springer International Publishing 2016. 9–20. doi:10.1007/978-3-319-30858-6_2

401 Rachmadi MF, Valdés-Hernández M del C, Komura T. Automatic irregular texture detection in brain MRI without human supervision. In: *Medical Image Computing and Computer Assisted Intervention – MICCAI 2018*. Springer. 2018. doi:10.1007/978-3-030-00931-1_58

402 Randhawa RS, Modi A, Jain P, *et al.* Improving Boundary Classification for Brain Tumor Segmentation and Longitudinal Disease Progression. In: Crimi A, Menze B, Maier O, *et al.*, eds. *Brainlesion: Glioma, Multiple Sclerosis, Stroke and Traumatic Brain Injuries*. Cham: : Springer International Publishing 2016. 65–74. doi:10.1007/978-3-319-55524-9_7

403 Raniga P, Schmitt P, Bourgeat P, *et al.* Local intensity model: An outlier detection framework with applications to white matter hyperintensity segmentation. In: *2011 IEEE International Symposium on Biomedical Imaging: From Nano to Macro*. 2011. 2057–60. doi:10.1109/ISBI.2011.5872817

404 Rao A, Ledig C, Newcombe V, *et al.* Contusion segmentation from subjects with Traumatic Brain Injury: A random forest framework. In: *2014 IEEE 11th International Symposium on Biomedical Imaging (ISBI)*. 2014. 333–6. doi:10.1109/ISBI.2014.6867876

405 Rexilius J, Hahn HK, Klein J, *et al.* Multispectral brain tumor segmentation based on histogram model adaptation. In: *Medical Imaging 2007: Computer-Aided Diagnosis*. International Society for Optics and Photonics 2007. 65140V. doi:10.1117/12.709410

406 Reza S, Linmin P, Iftekharuddin KM. Ischemic stroke lesion segmentation using local gradient and texture features. In: *Ischemic Stroke Lesion Segmentation*. 2015.

407 Rezaei M, Yang H, Meinel C. Deep Neural Network with l2-Norm Unit for Brain Lesions Detection. In: Liu D, Xie S, Li Y, *et al.*, eds. *Neural Information Processing*. Cham: : Springer International Publishing 2017. 798–807. doi:10.1007/978-3-319-70093-9_85

408 Riad MM, Platel B, de Leeuw F-E, *et al.* Detection of white matter lesions in cerebral small vessel disease. 2013. 867014. doi:10.1117/12.2007940

409 Rios Piedra EA, Ellingson BM, Taira RK, *et al.* Brain Tumor Segmentation by Variability Characterization of Tumor Boundaries. In: Crimi A, Menze B, Maier O, *et al.*, eds. *Brainlesion: Glioma, Multiple Sclerosis, Stroke and Traumatic Brain Injuries*. Cham: : Springer International Publishing 2016. 206–16. doi:10.1007/978-3-319-55524-9_20

410 Rodrigo F, Graffigna JP, Isoardi R, *et al.* Segmentation of Hyperintense Regions Applied to Multiple Sclerosis Lesions. In: Braidot A, Hadad A, eds. *VI Latin American Congress on Biomedical Engineering CLAIB 2014, Paraná, Argentina 29, 30 & 31 October 2014*. Cham: : Springer International Publishing 2015. 425–8. doi:10.1007/978-3-319-13117-7_109

411 Roy PK, Bhuiyan A, Janke A, *et al.* Automated Segmentation of White Matter Lesions Using Global Neighbourhood Given Contrast Feature-Based Random Forest and Markov Random Field. In: *2014 IEEE International Conference on Healthcare Informatics*. 2014. 1–6. doi:10.1109/ICHI.2014.75

412 Roy S, Maji P. A New Post-processing Method to Detect Brain Tumor Using Rough-Fuzzy Clustering. In: Kryszkiewicz M, Bandyopadhyay S, Rybinski H, *et al.*, eds. *Pattern Recognition and Machine Intelligence*. Cham: : Springer International Publishing 2015. 407–17. doi:10.1007/978-3-319-19941-2_39

For peer review only

413 S. Doyle, M. Reyes, M. Dojat, *et al.* Fully Automatic Brain Tumor Segmentation from Multiple MR Sequences using Hidden Markov Fields and Variational. In: *NCI-MICCAI BraTS*. 2013. /paper/Fully-Automatic-Brain-Tumor-Segmentation-from-MR-Menze-Reyes/c3912d865a263e42d51ae28850cd8332cd1612ee (accessed 16 Nov 2020).

414 S. Reza, K.M. Iftekharuddin. Improved brain tumor tissue segmentation using texture features. In: *Proceedings MICCAI BraTS (brain tumor segmentation challenge)*. 2014. 27–30.

415 Saha R, Phophalia A, Mitra SK. Brain Tumor Segmentation from Multimodal MR Images Using Rough Sets. In: Mukherjee S, Mukherjee S, Mukherjee DP, *et al.*, eds. *Computer Vision, Graphics, and Image Processing*. Cham: : Springer International Publishing 2017. 133–44. doi:10.1007/978-3-319-68124-5_12

416 Sankari A, Vigneshwari S. Automatic tumor segmentation using convolutional neural networks. In: *2017 Third International Conference on Science Technology Engineering Management (ICONSTEM)*. 2017. 268–72. doi:10.1109/ICONSTEM.2017.8261291

417 Schmidt M, Levner I, Greiner R, *et al.* Segmenting brain tumors using alignment-based features. In: *Fourth International Conference on Machine Learning and Applications (ICMLA'05)*. 2005. 6 pp.-. doi:10.1109/ICMLA.2005.56

418 Sehgal A, Goel S, Mangipudi P, *et al.* Automatic brain tumor segmentation and extraction in MR images. In: *2016 Conference on Advances in Signal Processing (CASP)*. 2016. 104–7. doi:10.1109/CASP.2016.7746146

419 Shah N, Ziauddin S, Shahid AR. Brain tumor segmentation and classification using cascaded random decision forests. In: *2017 14th International Conference on Electrical Engineering/Electronics, Computer, Telecommunications and Information Technology (ECTI-CON)*. 2017. 718–21. doi:10.1109/ECTICon.2017.8096339

420 Shaikh M, Anand G, Acharya G, *et al.* Brain Tumor Segmentation Using Dense Fully Convolutional Neural Network. In: Crimi A, Bakas S, Kuijf H, *et al.*, eds. *Brainlesion: Glioma, Multiple Sclerosis, Stroke and Traumatic Brain Injuries*. Cham: : Springer International Publishing 2018. 309–19. doi:10.1007/978-3-319-75238-9_27

421 Shen H, Wang R, Zhang J, *et al.* Multi-task Fully Convolutional Network for Brain Tumour Segmentation. In: Valdés Hernández M, González-Castro V, eds. *Medical Image Understanding and Analysis*. Cham: : Springer International Publishing 2017. 239–48. doi:10.1007/978-3-319-60964-5_21

422 Shivhare SN, Sharma S, Singh N. An Efficient Brain Tumor Detection and Segmentation in MRI Using Parameter-Free Clustering. In: Tanveer M, Pachori RB, eds. *Machine Intelligence and Signal Analysis*. Singapore: : Springer 2019. 485–95. doi:10.1007/978-981-13-0923-6_42

423 Shreyas V, Pankajakshan V. A deep learning architecture for brain tumor segmentation in MRI images. In: *2017 IEEE 19th International Workshop on Multimedia Signal Processing (MMSP)*. 2017. 1–6. doi:10.1109/MMSP.2017.8122291

424 Soltaninejad M, Zhang L, Lambrou T, *et al.* MRI Brain Tumor Segmentation and Patient Survival Prediction Using Random Forests and Fully Convolutional Networks. In: Crimi A, Bakas S, Kuijf H, *et al.*, eds. *Brainlesion: Glioma, Multiple Sclerosis, Stroke and Traumatic Brain Injuries*. Cham: : Springer International Publishing 2018. 204–15. doi:10.1007/978-3-319-75238-9_18

425 Song B, Chou C-R, Chen X, *et al.* Anatomy-Guided Brain Tumor Segmentation and Classification. In: Crimi A, Menze B, Maier O, *et al.*, eds. *Brainlesion: Glioma, Multiple Sclerosis, Stroke and Traumatic Brain Injuries*. Cham: : Springer International Publishing 2016. 162–70. doi:10.1007/978-3-319-55524-9_16

426 Souplet J-C, Lebrun-Frenay C, Ayache N, *et al.* An Automatic Segmentation of T2-FLAIR Multiple Sclerosis Lesions. In: *MICCAI-Multiple Sclerosis Lesion Segmentation Challenge Workshop*. 2008.

427 Srivastava S, Maes F, Vandermeulen D, *et al.* Feature-based statistical analysis of structural MR data for automatic detection of focal cortical dysplastic (FCD) lesions. In: *2004 2nd IEEE International Symposium on Biomedical Imaging: Nano to Macro (IEEE Cat No. 04EX821)*. 2004. 1127-1130 Vol. 2. doi:10.1109/ISBI.2004.1398741

428 Stefan Bauer, Thomas Fejes, Johannes Slotboom, *et al.* Segmentation of brain tumor images based on integrated hierarchical classification and regularization. 2012.

429 Subbanna N, Precup D, Arbel T. Iterative Multilevel MRF Leveraging Context and Voxel Information for Brain Tumour Segmentation in MRI. In: *2014 IEEE Conference on Computer Vision and Pattern Recognition*. 2014. 400–5. doi:10.1109/CVPR.2014.58

430 Subbanna NK, Precup D, Collins DL, *et al.* Hierarchical Probabilistic Gabor and MRF Segmentation of Brain Tumours in MRI Volumes. In: Mori K, Sakuma I, Sato Y, *et al.*, eds. *Medical Image Computing and Computer-Assisted Intervention – MICCAI 2013*. Berlin, Heidelberg: : Springer 2013. 751–8. doi:10.1007/978-3-642-40811-3_94

431 Subbanna N, Precup D, Arnold D, *et al.* IMaGe: Iterative Multilevel Probabilistic Graphical Model for Detection and Segmentation of Multiple Sclerosis Lesions in Brain MRI. In: *International Conference on Information Processing in Medical Imaging*. Cham: : Springer 2015. 514–26. doi:10.1007/978-3-319-19992-4_40

432 Subbanna N, Shah M, Francis S, *et al.* MS Lesion Segmentation using Markov Random Fields. In: *Proceedings of International Conference on Medical Image Computing and Computer Assisted Intervention, London, UK*. 2009.

433 Szilágyi L, Lefkovits L, Iantovics B, *et al.* Automatic Brain Tumor Segmentation in Multispectral MRI Volumetric Records. In: Arik S, Huang T, Lai WK, *et al.*, eds. *Neural Information Processing*. Cham: : Springer International Publishing 2015. 174–81. doi:10.1007/978-3-319-26561-2_21

434 T. K. T. K. Lun, W. Hsu. Brain tumor segmentation using deep convolutional neural network. In: *Proceedings of BRATS-MICCAI*. 2016.

435 Tang H, Lu H, Liu W, *et al.* Tumor segmentation from single contrast MR images of human brain. In: *2015 IEEE 12th International Symposium on Biomedical Imaging (ISBI)*. 2015. 46–9. doi:10.1109/ISBI.2015.7163813

436 Thomas Taylor, Nigel John, Patricia Buendia, *et al.* Map-reduce enabled hidden Markov models for high throughput multimodal brain tumor segmentation. In: *Proceedings of the MICCAI Challenge on Multimodal Brain Tumor Image Segmentation (BRATS) 2013*. 2013.

437 Uchiyama Y, Kunieda T, Hara T, *et al.* Automatic segmentation of different-sized leukoaraiosis regions in brain MR images. In: *Medical Imaging 2008: Computer-Aided Diagnosis*. International Society for Optics and Photonics 2008. 69151S. doi:10.1117/12.770045

438 Urban G, Bendszus M, Hamprecht F, *et al.* Multi-modal Brain Tumor Segmentation using Deep Convolutional Neural Networks. In: *MICCAI BraTS (brain tumor segmentation) challenge*. 2014. /paper/Multi-modal-Brain-Tumor-Segmentation-using-Deep-Urban-Bendszus/97efa3acd4a45b28f3c9e25c961a9379abdc811b (accessed 15 Nov 2020).

439 Vaidhya K, Thirunavukkarasu S, Alex V, *et al.* Multi-modal Brain Tumor Segmentation Using Stacked Denoising Autoencoders. In: Crimi A, Menze B, Maier O, *et al.*, eds. *Brainlesion: Glioma, Multiple Sclerosis, Stroke and Traumatic Brain Injuries*. Cham: : Springer International Publishing 2016. 181–94. doi:10.1007/978-3-319-30858-6_16

440 Vaidya S, Chunduru A, Muthuganapathy R, *et al.* Longitudinal multiple sclerosis lesion segmentation using 3D convolutional neural networks. In: *Proceedings of the 2015 Longitudinal Multiple Sclerosis Lesion Segmentation Challenge*. 2015. 1–2.

441 Vaishnavee KB, Amshakala K. An automated MRI brain image segmentation and tumor detection using SOM-clustering and Proximal Support Vector Machine classifier. In: *2015 IEEE International Conference on Engineering and Technology (ICETECH)*. 2015. 1–6. doi:10.1109/ICETECH.2015.7275030

442 Wang G, Li W, Ourselin S, *et al.* Automatic Brain Tumor Segmentation Using Cascaded Anisotropic Convolutional Neural Networks. In: Crimi A, Bakas S, Kuijf H, *et al.*, eds. *Brainlesion: Glioma, Multiple Sclerosis, Stroke and Traumatic Brain Injuries*. Cham: : Springer International Publishing 2018. 178–90. doi:10.1007/978-3-319-75238-9_16

443 Wang T, Cheng I, Basu A. Fully automatic brain tumor segmentation using a normalized Gaussian Bayesian Classifier and 3D Fluid Vector Flow. In: *2010 IEEE International Conference on Image Processing*. 2010. 2553–6. doi:10.1109/ICIP.2010.5652559

444 Wang Y, Katsaggelos AK, Wang X, *et al.* A deep symmetry convnet for stroke lesion segmentation. In: *2016 IEEE International Conference on Image Processing (ICIP)*. 2016. 111–5. doi:10.1109/ICIP.2016.7532329

445 Weiss N, Rueckert D, Rao A. Multiple Sclerosis Lesion Segmentation Using Dictionary Learning and Sparse Coding. In: Mori K, Sakuma I, Sato Y, *et al.*, eds. *Medical Image Computing and Computer-Assisted Intervention – MICCAI 2013*. Berlin, Heidelberg: : Springer 2013. 735–42. doi:10.1007/978-3-642-40811-3_92

446 Xu Y, Géraud T, Puybareau É, *et al.* White Matter Hyperintensities Segmentation in a Few Seconds Using Fully Convolutional Network and Transfer Learning. In: Crimi A, Bakas S, Kuijf H, *et al.*, eds. *Brainlesion: Glioma, Multiple Sclerosis, Stroke and Traumatic Brain Injuries*. Cham: : Springer International Publishing 2018. 501–14. doi:10.1007/978-3-319-75238-9_42

447 Xuan Chen, Nguyen BP, Chee-Kong Chui, *et al.* Automated brain tumor segmentation using kernel dictionary learning and superpixel-level features. In: *2016 IEEE International Conference on Systems, Man, and Cybernetics (SMC)*. 2016. 002547–52. doi:10.1109/SMC.2016.7844622

448 Xuan X, Liao Q. Statistical Structure Analysis in MRI Brain Tumor Segmentation. In: *Fourth International Conference on Image and Graphics (ICIG 2007)*. 2007. 421–6. doi:10.1109/ICIG.2007.181

449 Yoo Y, Tang LW, Brosch T, *et al.* Deep Learning of Brain Lesion Patterns for Predicting Future Disease Activity in Patients with Early Symptoms of Multiple Sclerosis. In: Carneiro G, Mateus D, Peter L, *et al.*, eds. *Deep Learning and Data Labeling for Medical Applications*. Cham: : Springer International Publishing 2016. 86–94. doi:10.1007/978-3-319-46976-8_10

450 Yu C-P, Ruppert G, Nguyen D, *et al.* Statistical Asymmetry-based Brain Tumor Segmentation from 3D MR Images. 2012.

451 Yu R, Xiao L, Wei Z, *et al.* Automatic Segmentation of White Matter Lesions Using SVM and RSF Model in Multi-channel MRI. In: Zhang Y-J, ed. *Image and Graphics*. Cham: : Springer International Publishing 2015. 654–63. doi:10.1007/978-3-319-21978-3_57

For peer review only

452 Zabir I, Paul S, Rayhan MA, *et al.* Automatic brain tumor detection and segmentation from multi-modal MRI images based on region growing and level set evolution. In: *2015 IEEE International WIE Conference on Electrical and Computer Engineering (WIECON-ECE)*. 2015. 503–6. doi:10.1109/WIECON-ECE.2015.7443979

453 Zacharaki EI, Erus G, Bezerianos A, *et al.* Fuzzy Multi-channel Clustering with Individualized Spatial Priors for Segmenting Brain Lesions and Infarcts. In: Iliadis L, Maglogiannis I, Papadopoulos H, *et al.*, eds. *Artificial Intelligence Applications and Innovations*. Berlin, Heidelberg: : Springer 2012. 76–85. doi:10.1007/978-3-642-33412-2_8

454 Zacharaki EI, Kanterakis S, Bryan RN, *et al.* Measuring Brain Lesion Progression with a Supervised Tissue Classification System. In: Metaxas D, Axel L, Fichtinger G, *et al.*, eds. *Medical Image Computing and Computer Assisted Intervention – MICCAI 2008*. Berlin, Heidelberg: : Springer 2008. 620–7. doi:10.1007/978-3-540-85988-8_74

455 Zhan T, Gu S, Jiang L, *et al.* A Novel Brain Tumor Segmentation Method for Multi-Modality Human Brain MRIs. In: *MUE 2015*. 2015. doi:10.14257/ijmue.2015.10.11.11

456 Zhao L, Duygu Sarikaya, Corso JJ. Automatic brain tumor segmentation with MRF on supervoxels. In: *Multimodal Brain Tumor Segmentation*. 2013. 51.

457 Zhe Xiao, Ruohan Huang, Yi Ding, *et al.* A deep learning-based segmentation method for brain tumor in MR images. In: *2016 IEEE 6th International Conference on Computational Advances in Bio and Medical Sciences (ICCABS)*. 2016. 1–6. doi:10.1109/ICCABS.2016.7802771

458 Zhiqiang Lao, Dinggang Shen, Jawad A, *et al.* Automated segmentation of white matter lesions in 3D brain MR images, using multivariate pattern classification. In: *3rd IEEE International Symposium on Biomedical Imaging: Nano to Macro, 2006.* 2006. 307–10. doi:10.1109/ISBI.2006.1624914

459 Zikic D, Glocker B, Konukoglu E, *et al.* Context-sensitive Classification Forests for Segmentation of Brain Tumor Tissues. 2012.

460 Zikic D, Glocker B, Konukoglu E, *et al.* Decision Forests for Tissue-Specific Segmentation of High-Grade Gliomas in Multi-channel MR. In: Ayache N, Delingette H, Golland P, *et al.*, eds. *Medical Image Computing and Computer-Assisted Intervention – MICCAI 2012*. Berlin, Heidelberg: : Springer 2012. 369–76. doi:10.1007/978-3-642-33454-2_46

461 Yi D, Zhou M, Chen Z, *et al.* 3-D Convolutional Neural Networks for Glioblastoma Segmentation. *arXiv:161104534 [cs]* Published Online First: 14 November 2016.http://arxiv.org/abs/1611.04534 (accessed 15 Nov 2020).

462 Beers A, Chang K, Brown J, *et al.* Sequential 3D U-Nets for Biologically-Informed Brain Tumor Segmentation. *arXiv:170902967 [cs]* Published Online First: 9 September 2017.http://arxiv.org/abs/1709.02967 (accessed 16 Nov 2020).

463 Styner M, Lee J, Chin B, *et al.* 3D Segmentation in the Clinic: A Grand Challenge II: MS lesion segmentation. *The MIDAS Journal* 2008;:638.

464 Menze BH, Jakab A, Bauer S, *et al.* The Multimodal Brain Tumor Image Segmentation Benchmark (BRATS). *IEEE Trans Med Imaging* 2015;**34**:1993–2024. doi:10.1109/TMI.2014.2377694

465 Carass A, Roy S, Jog A, *et al.* Longitudinal multiple sclerosis lesion segmentation: Resource and challenge. *NeuroImage* 2017;**148**:77–102. doi:10.1016/j.neuroimage.2016.12.064

For peer review only

466 Maier O, Menze BH, von der Gablentz J, *et al.* ISLES 2015 - A public evaluation benchmark for ischemic stroke lesion segmentation from multispectral MRI. *Med Image Anal* 2017;**35**:250–69. doi:10.1016/j.media.2016.07.009

467 Sled JG, Zijdenbos AP, Evans AC. A nonparametric method for automatic correction of intensity nonuniformity in MRI data. *IEEE Trans Med Imaging* 1998;**17**:87–97. doi:10.1109/42.668698

468 Tustison NJ, Avants BB, Cook PA, *et al.* N4ITK: improved N3 bias correction. *IEEE Trans Med Imaging* 2010;**29**:1310–20. doi:10.1109/TMI.2010.2046908

469 Smith SM. Fast robust automated brain extraction. *Hum Brain Mapp* 2002;**17**:143–55. doi:10.1002/hbm.10062

470 William Penny, Karl Friston, John Ashburner, *et al.*, editors. *Statistical Parametric Mapping: The Analysis of Functional Brain Images - 1st Edition*. 1st ed. Elsevier 2006. https://www.elsevier.com/books/statistical-parametric-mapping-the-analysis-of-functional-brain-images/penny/978-0-12-372560-8 (accessed 23 Nov 2020).

471 Smith SM, Jenkinson M, Woolrich MW, *et al.* Advances in functional and structural MR image analysis and implementation as FSL. *Neuroimage* 2004;**23 Suppl 1**:S208-219. doi:10.1016/j.neuroimage.2004.07.051

472 Bø HK, Solheim O, Jakola AS, *et al.* Intra-rater variability in low-grade glioma segmentation. *J Neurooncol* 2017;**131**:393–402. doi:10.1007/s11060-016-2312-9

473 Zijdenbos AP, Dawant BM, Margolin RA, *et al.* Morphometric analysis of white matter lesions in MR images: method and validation. *IEEE Trans Med Imaging* 1994;**13**:716–24. doi:10.1109/42.363096

474 Gibson E, Hu Y, Huisman HJ, *et al.* Designing image segmentation studies: Statistical power, sample size and reference standard quality. *Med Image Anal* 2017;**42**:44–59. doi:10.1016/j.media.2017.07.004

475 Recent Advances in MRI Technology. Imaging Technology News. 2016.https://www.itnonline.com/article/recent-advances-mri-technology (accessed 23 Nov 2020).

476 OECD Statistics. https://stats.oecd.org/Index.aspx?ThemeTreeId=9 (accessed 23 Nov 2020).

477 FDA Clears First 7T MRI System, Magnetom Terra. Imaging Technology News. 2017.https://www.itnonline.com/content/fda-clears-first-7t-mri-system-magnetom-terra (accessed 23 Nov 2020).

478 Maier-Hein L, Eisenmann M, Reinke A, *et al.* Why rankings of biomedical image analysis competitions should be interpreted with care. *Nat Commun* 2018;**9**:5217. doi:10.1038/s41467-018-07619-7

479 Jorritsma W, Cnossen F, van Ooijen PMA. Improving the radiologist-CAD interaction: designing for appropriate trust. *Clin Radiol* 2015;**70**:115–22. doi:10.1016/j.crad.2014.09.017

480 Gryska E, Cerna K, Heckemann RA. Increasing trust through the design of algorithm-based lesion segmentation support systems. In: *iConference 2020 Proceedings*. iSchools 2020. https://www.ideals.illinois.edu/handle/2142/106560 (accessed 23 Nov 2020).

FIGURE LIST AND CAPTIONS

500 Figure 1: *Flowchart of the article selection process from the result of querying three databases. For each stage, the number of articles selected is shown and numbers of excluded articles and reasons are given.*

For peer review only

Figure 2: *The number of algorithms described in the included articles developed for and validated on particular lesion type. The distinction is made between multiple sclerosis (MS) lesions and other white matter lesions (WML) following the similar distinction present in the reviewed articles and lesion segmentation challenges. "Multiple lesion types" refers to algorithms that were evaluated on more than one type of lesion. "Other lesion types" types of lesions included focal cortical dysplasia, metastasis, traumatic brain injury, cerebral palsy, abscess, and necrosis.*

Figure 3: *The number of articles published per year in the eligible sample with an indication of the number of articles that presented a segmentation method for a particular lesion type. We fit a linear function to the number of papers published each year from 2012 to estimate the development of the field in the upcoming years.*

Figure 4: The number of patients (n) whose images were used to validate the methods in the eligible articles.

Figure 5: *Distribution of the most common algorithms and models implemented in the lesion segmentation methods proposed by the articles eligible for the review (ANN – artificial neural networks, DT – decision trees, EM – expectation maximization, FC – fuzzy clustering, MM – mixture models, RF – random fields, SVM – support vector machines, THR – thresholding).*

For peer review only

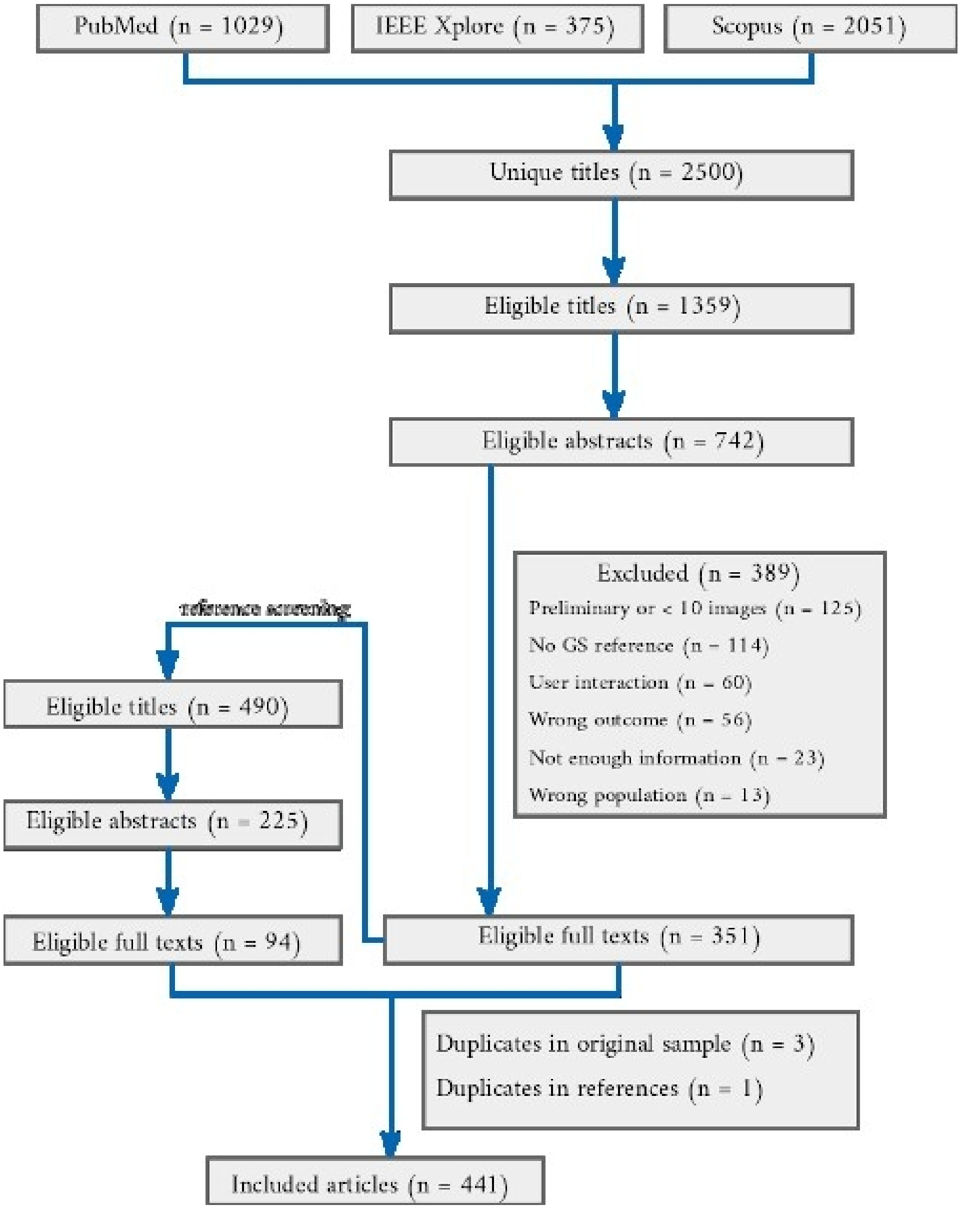

Flowchart of the article selection process from the result of querying three databases. For each stage, the number of articles selected is shown and numbers of excluded articles and reasons are given.

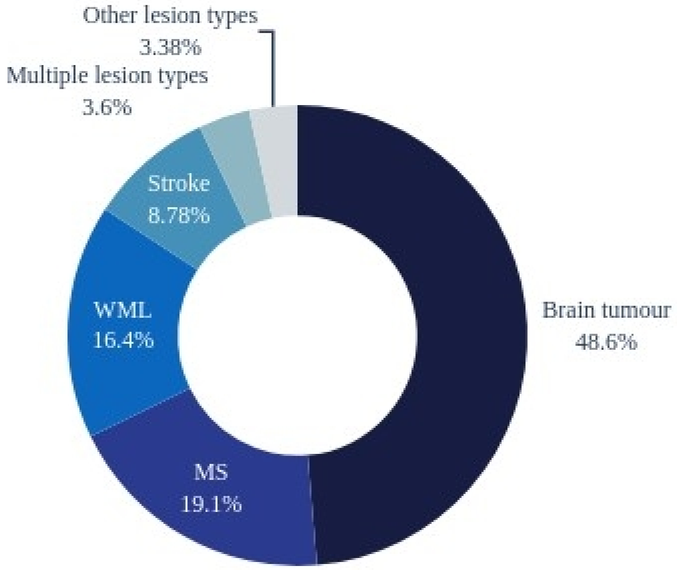

The number of algorithms described in the included articles developed for and validated on particular lesion type. The distinction is made between multiple sclerosis (MS) lesions and other white matter lesions (WML) following the similar distinction present in the reviewed articles and lesion segmentation challenges. “Multiple lesion types” refers to algorithms that were evaluated on more than one type of lesion. “Other lesion types” types of lesions included focal cortical dysplasia, metastasis, traumatic brain injury, cerebral palsy, abscess, and necrosis.

89x89mm (96 x 96 DPI)

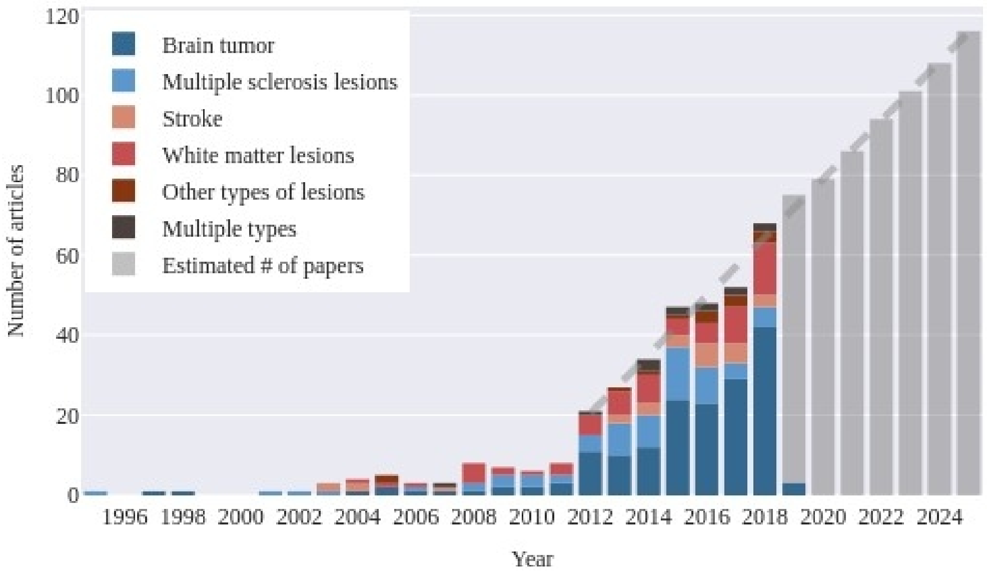

The number of articles published per year in the eligible sample with an indication of the number of articles that presented a segmentation method for a particular lesion type. We fit a linear function to the number of papers published each year from 2012 to estimate the development of the field in the upcoming years.

137x80mm (96 x 96 DPI)

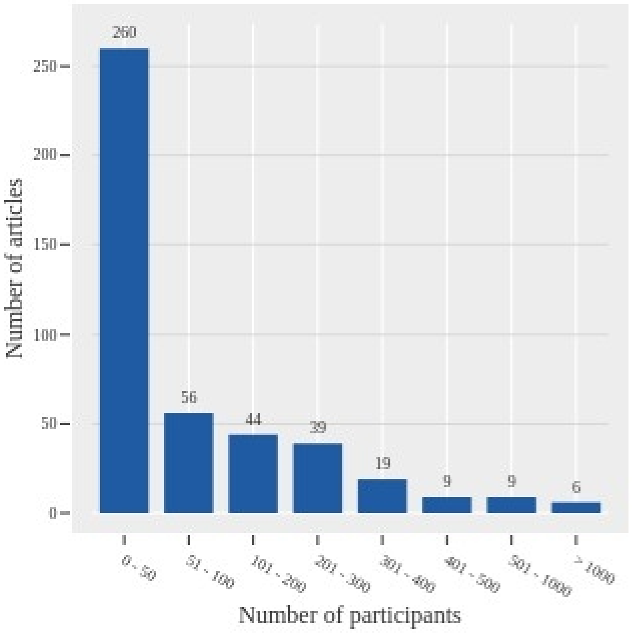

The number of patients (n) whose images were used to validate the methods in the eligible articles.

89x89mm (96 x 96 DPI)

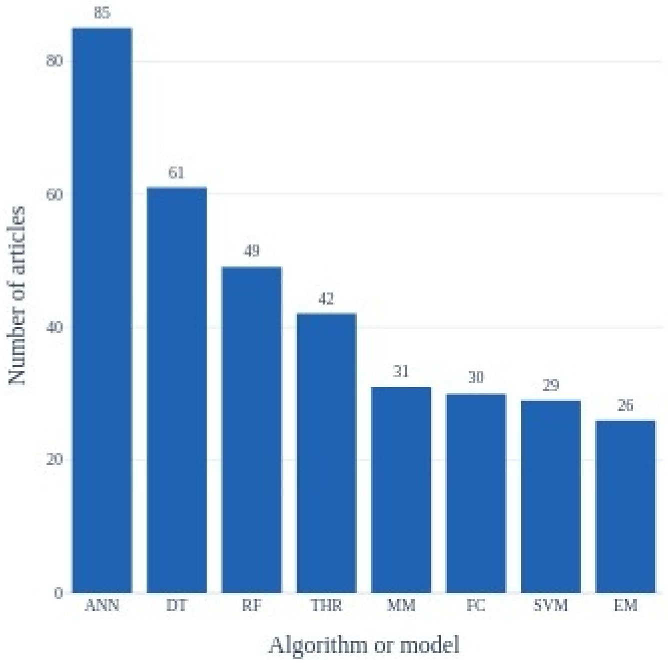

Distribution of the most common algorithms and models implemented in the lesion segmentation methods proposed by the articles eligible for the review (ANN – artificial neural networks, DT – decision trees, EM – expectation maximization, FC – fuzzy clustering, MM – mixture models, RF – random fields, SVM – support vector machines, THR – thresholding).

89x89mm (96 x 96 DPI)

Bibliographic data					General comments	Segmentation								Study cohort					Validation		
Item type	Publication year	Author(s)	Title	Publication Title		Multi-sequence input	Input sequence(s):	Algorithms and computational theories	Preprocessing procedures	Hardware	Segmentation time	Lesion classification used	Software made available by authors	Lesion type	Diagnosed conditions	Sample size	Sample source corrected	Multiscanner images	Reference segmentation	Decision (if multiple raters and specified)	Evaluation measures
C – conference paper<br>J – journal article						y - yes<br>n - no	CBF – Cerebral blood flow<br>DIR – Double inversion recovery<br>DSC – Dynamic susceptibility contrast<br>DTI – Diffusion tensor imaging<br>DWI – Diffusion weighted imaging<br>FA – Fractional anisotropy<br>FLAIR – Fluid-attenuated inversion recovery<br>FSE – Fast spin echo<br>MPR/MPRAGE – Magnetization Prepared Rapid Acquisition Gradient Echo<br>PD – Proton density<br>PWI – Perfusion weighted imaging<br>(R)CBV – (Relative) Cerebral blood volume<br>SD – Spin density weighted<br>T1c – T1 weighted with contrast<br>T1 – T1 weighted<br>T2 – T2 weighted<br>Tmax – Time to maximum<br>TTP – Time to peak		0u1v – 0 mean unit variance<br>ADF – Anidotropic diffusion filtering<br>BE – Brain extraction<br>BFC – Bias field(field inhomogeneity) correction<br>EM – Expectation maximization<br>IN – Intensity normalization<br>M(N)I – Mutual (normalized) information<br>N3 – Sled et al. 1998<br>N4 - Tustison et al. 2010<br>n/s – Not specified<br>REG – Image (co-) registration<br>WM – White matter			y - yes<br>n - no	y - yes<br>n - no	AD – Alzheimer's disease BT – Brain tumour CADASIL - Cerebral autosomal dominant arteriopathy with subcortical infarcts and leukoencephalopathy CI – Cognitive impairment CIS – Clinically isolatd syndrome CP – Cerebral palsy D - Dementia DM(2) – Diabetes mellitus (type II) EP – Epilepsy FCD – Focal cortical displasia GB(M) – Glioblastoma (multiforme) G – glioma HEM – Hemorrhage or bleeding HGG - high grade glioma HT – hypertension IFL – ischemic focal lesions LBD – Lewy body dementia LGG - low grade glioma MCI - minimal cognitive impairement MET – Metastasis MS – Multiple sclerosis lesions NEC – Necrosis (RR)MS - (Relapsing-remitting) multiple sclerosis SLE - systemic lupus erythematosus S – Stroke SVD – Small vessel disease TBI – Traumatic brain Injury T - Tumours VaD – Vascular disease WML – White matter lesions (other than MS lesions)			**1 – Publicably available data with reference segmentation (source)**<br>**2 – Publicably available data without reference segmentation (source)**<br>**3 – Non-public data sources (source)**	y - yes<br>n - no	**Procedure, number of raters**		Acc - Accuracy<br>AD - Average distance<br>avg - Average<br>DE - Detection error<br>DER - Detection error rate<br>DSC - Dice similarity coefficient<br>F1 - F1 score<br>FN - False negative<br>FNR - False negative ratio<br>FP - False positive<br>FPR - False positive rate<br>(F)ROC - (Free-response Receiver) operator curve<br>Hdff - Hausdorff distance<br>ICC - Inter-class correlation<br>Jcc - Jaccard coefficient<br>Kappa - Kappa coefficient<br>NPV - Negative predictive value<br>OER - Outline error rate<br>PPV - Positive predictive value<br>sd - Standard deviation<br>SD - Surface distance ??<br>Sen, TPR, Overlap fraction - Sensitivity<br>SI - Similarity index<br>Spe - Specificity<br>SSD - Symmetric SD<br>TI - Tanimoto index<br>TP - True positive<br>VC - Volume correlation<br>VD - Volume difference<br>VE - Volume error<br>VO - Volume overlap<br>VR - Regression analysis of volume
J	2014	M. Cabezas et al.	BOOST: A supervised approach for multiple sclerosis lesion segmentation	Journal of Neuroscience Methods		y	T1, T2, PD, FLAIR	Boosting	BE:BET; BFC:N4; denoising:ADF; IN:histogram matching	n/a	n/a	n	n	MS	-	45	3	y	Semiautomatic		DSC; avgSD
J	2014	S. Parisot et al.	Concurrent tumor segmentation and registration with uncertainty-based sparse non-uniform graphs	Medical Image Analysis	Inter-rater agreement evaluated	y	T2, FLAIR	Markov Random Field	BE; REG:rigid; IN:matching median and interquartile range to reference	n/a	~5 min	n	n	BT	LGG	110 (27 segmented); 10	1 (BraTS'12); 3	y	Manual, 2		DSC; FPR; TPR; MAD
J	2013	M. D. Steenwijk et al.	Accurate white matter lesion segmentation by k nearest neighbor classification with tissue type priors (kNN-TTPs)	NeuroImage: Clinical	Inter-rater agreement evaluated	y	T1, FLAIR	K-Nearest Neighbours	BE:BET; BFC:N3	n/a	n/a	n	n	WML	MS; HT	20; 20	3	y	Manual, 3	consensus	DSC; ICC; Sen; OER; DER
J	2018	D. S. Meier et al.	Dual-Sensitivity Multiple Sclerosis Lesion and CSF Segmentation for Multichannel 3T Brain MRI	Journal of Neuroimaging	Scan-rescan reproducibility	y	T1, T2, FLAIR	Other	BFC:N4; REG:rigid, affine + Bspline(BRAINS/ITK); BE:BET; tissue maps:Freesurfer; IN:histogram matching	n/a	n/a	n	n	MS	MS	29; 13x2	3	n	Manual, 2	supervision	DSC; Jcc; Hdff; Spe; PPV; Sen; ICC; Bland-Altman
J	2013	A. Sanjuán et al.	Automated identification of brain tumors from single MR images based on segmentation with refined patient-specific priors	Front. Neurosci.	Inter-rater agreement evaluated	n	T1	Previously proposed	n/a	n/a	n/a	n	n	BT	T	18 + 64 healthy	3	n	Manual, 2		DSC; ROC
C	2014	S. Roy et al.	Example based lesion segmentation	Proceedings of SPIE - The International Society for Optical Engineering		y	T1, FLAIR	Previously proposed	BE; IN:mean WM intensity=1	n/a	n/a	n	n	WML	MS	47	3 (described in Shee et al. 2009)	n	Manual		DSC;VC; Sen; Spe
J	2016	A. Galimzianova et al.	Stratified mixture modeling for segmentation of white-matter lesions in brain MR images	NeuroImage	Manual corrections of preprocessing (brain extraction)	y	T1, T2, FLAIR	Comparison	BE:Iglesias; REG:rigid; BFC:N4; downsampling	n/a	n/a	n	n	MS	MS	30	3	n	Manual, 2	consensus	DSC
C	2015	S. Roy and P. Maji	A New Post-processing Method to Detect Brain Tumor Using Rough-Fuzzy Clustering	Pattern Recognition and Machine Intelligence		n/a		Fuzzy C-Means, Region Growing	n/a	n/a	n/a	n	n	BT	-	10	1 (BraTS'12)	y	-		DSC; other
J	2011	S. Klöppel et al.	A comparison of different automated methods for the detection of white matter lesions in MRI data	NeuroImage	Manual corrections of preprocessing	y	T1, FLAIR	Comparison	REG:MI; tissue maps, spatial normalization, BFC:SPM8; IN:0 median + 1 interquartile interval; resampling	n/a	few sec to 5 hrs	n	n	WML	MCI, D	20	3	n	Manual, 1&2		PPV; Sen; ROC; DSC
C	2018	A. Bougacha et al.	Comparative study of supervised and unsupervised classification methods: Application to automatic MRI glioma brain tumors segmentation	[illegible] International Conference on Advanced Technologies for Signal and Image [illegible]		y	T1, T1c, T2, FLAIR	Comparison	n/a	n/a	n/a	n	n	BT	LGG; HGG	273	1 (BraTS'15)	y	-		DSC; Jcc
J	2005	S. Srivastava et al.	Feature-based statistical analysis of structural MR data for automatic detection of focal cortical dysplastic lesions	NeuroImage		n	T1	Previously proposed	REG:affine; tissue maps w/ non-rigid registration; IN	n/a	n/a	n	n	FCD	FCD	17 + 64 controls	3	y	Manual		VO
J	2013	S. Datta and P. A. Narayana	A comprehensive approach to the segmentation of multichannel three-dimensional MR brain images in multiple sclerosis	NeuroImage: Clinical		y	T1, T2, FLAIR	Other	REG:rigid (SPM2); BE:thresholding; BFC:SPM2 + ADF; IN:histogram matching	n/a	n/a	n	n	MS	-	60; 50	1 (MS GC MICCAI'08); 3	y	Segmentation correction		VD; avgSD; TPR; FPR; Bland-Altman; VC
J	2016	E. Roura et al.	Automated Detection of Lupus White Matter Lesions in MRI	Front. Neuroinform	Integrated preprocessing	y	T1, FLAIR	Thresholding	REG:SPM; BE:SPM; tissue masks:SPM; denoising:ADF; BFC:EM-based	n/a	n/a	n	y	WML	SLE	20	3	n	Manual, 1		DSC; PPV; TPR; VC; Bland-Altman; ROC
J	2018	W. Chen et al.	Computer-Aided Grading of Gliomas Combining Automatic Segmentation and Radiomics	International Journal of Biomedical Imaging		y	T1, T1c, T2, FLAIR	Previously proposed	BFC:N4; IN:histogram matching + 0u1v	GeForce GTX 1080 Ti GPU	n/a	LGG, HGG	n	BT	LGG; HGG	274	1 (BraTS'15)	y	-		DSC; Sen; Spe

For peer review only

J	2018	P. Moeskops et al.	Evaluation of a deep learning approach for the segmentation of brain tissues and white matter hyperintensities of presumed vascular origin in MRI	NeuroImage: Clinical	Inter-rater variability evaluated (visual scores)	y	T1, T1 inversion recovery, FLAIR	Artificial neural networks	n/a	n/a	n/a	n	n	WML	DM2	20; 96 relatively healthy; 110	3 (UDES2, Reijmer et al., 2013); 3 (the Dutch Parelsnoer Study, Aalten et al., 2014)	n	Manual		DSC; avgSD; VC; Sen; DER; OER
J	2018	Y. Ling et al.	Validation and Optimization of BIANCA for the Segmentation of Extensive White Matter Hyperintensities	Neuroinformatics	Inter-rater agreement evaluated	works with single/multi	T1, FLAIR	Previously proposed	BE:BET; REG:FLIRT; IN:variance scaling	I5 3.2GHz	4-22 min	n	y	WML	CADASIL	90; 66	3 (Chabriat et al. 2016)	y	Semiautomatic		DSC; FPR; FNR; ICC; Bland-Altman
J	2018	M. Dadar et al.	Validation of T1w-based segmentations of white matter hyperintensity volumes in large-scale datasets of aging	Human Brain Mapping		n	T1	Previously proposed	denoising:ref; BFC:ref; IN:ref; REG:non-linear	n/a	n/a	n	n	WML	MCI; AD	70; 669; 481	2 (ADNI); 2 (ADNI2/GO); 3 (Davis Alzheimer's Disease Center, Hinton et al. 2010)	y	Manual, 1		VC; VR
J	2018	O. Charron et al.	Automatic detection and segmentation of brain metastases on multimodal MR images with a deep convolutional neural network	Computers in Biology and Medicine		n/a	T1, T1c, FLAIR	Artificial neural networks	REF:rigid (FLIRT) + resampling; BE:BET; BFC:N4; IN:0u1v + thresholding	n/a	n/a	n	n	MET	-	182 + 3 of other lesion causes	3 (Paul Strauss Center, Strasbourg, France)	n	Manual, multiple raters		DSC; Sen; PPV
J	2017	R. Meier et al.	Automatic estimation of extent of resection and residual tumor volume of patients with glioblastoma	Journal of Neurosurgery	Intended clinical application	y	T1, T1c, T2, FLAIR	Previously proposed	BE:ITK; REG	n/a	n/a	n	n	BT	GB	19	3	y	Manual, 5	supervision	other
J	2017	F. Kellner-Weldon et al.	Comparison of perioperative automated versus manual two-dimensional tumor analysis in glioblastoma patients	European Journal of Radiology	Intended clinical application, Preprocessing failure, Segmentation failure	y	T1, T1c, T2, FLAIR	Previously proposed	n/a	n/a	n/a	n	n	BT	GB	92x2	3	y	n/a		other
J	2017	A. de Sitter et al.	Performance of five research-domain automated WM lesion segmentation methods in a multi-center MS study	NeuroImage	Inter-rater agreement evaluated	y*	T1, FLAIR	Comparison	n/a	n/a	n/a	n	n	MS	MS	70	3 (MAGNIMS, Ropele et al., 2014)	y	Manual, 2	supervision	VD; ICC; DSC; DER; OER
J	2017	R. McKinley et al.	Fully automated stroke tissue estimation using random forest classifiers (FASTER)	J Cereb Blood Flow Metab	Inter-rater agreement evaluated	y	T1c, T2, DWI, DSC, PWI	Decision Tree	denoising:Gaussian iltering; REG:rigid; BE:ref; resampling:linear interpolation; IN:0-225 range	n/a	n/a	n	n	S	S	80x2 + 20	3 (Bernese stroke registry)	y	Semiautomatic		ROC; Sen; Spe; PPV
J	2015	R. McKinley et al.	Segmenting the ischemic penumbra: a decision forest approach with automatic threshold finding	Brainlesion: Glioma, Multiple Sclerosis, Stroke and Traumatic Brain Injuries		y	T1c, T2, DWI, CBF, CBV, TTP, Tmax	Decision Tree	filtering:ITK; IN: 0225r; REG	n/a	n/a	n	n	S	-	50	1 (ISLES'15)	n	-		DSC
J	2017	Y. Renping et al.	Coarse Classification to Region-Scalable Refining for White Matter Lesions Segmentation in Multi-Channel MRI	CNS & Neurological Disorders-Drug Targets		y	T1, T2, PD, FLAIR	Support Vector Machine, Level Set	REG:rigid; BE:ref; BFC: N3; IN: paper	n/a	n/a	n	n	WML	-	45	3 (ACCORD-MIND)	y	Manual, 1		TPR; TNR; PPV; DSC
J	2016	Ž. Lesjak, F. Pernuš, B. Likar, Ž. Špiclin	Validation of White-Matter Lesion Change Detection Methods on a Novel Publicly Available MRI Image Database	Neuroinformatics		y	T1, T2, FLAIR	Comparison	REG:affine; tissue maps – WM; BFC:N4; BE:BET 2; resampling:ANTs; IN:M u & v	n/a	n/a	n	n	WML	MS	20x2	3 (University Medical Centre Ljubljana)	y	Manual, 2	consensus	ROC; DSC; other
J	2016	M. B. Hansen et al.	Automated estimation of salvageable tissue: Comparison with expert readers.	Journal of Magnetic Resonance Imaging		y	PWI, DWI	Previously proposed	n/a	I7 2.6GHz	28 sec	n	y	S	-	167	3 (I-KNOW)	y	Manual, 4	consensus	DSC; VC; Bland-Altman
J	2016	R. Meier et al.	Clinical Evaluation of a Fully-automatic Segmentation Method for Longitudinal Brain Tumor Volumetry	Scientific Reports	Inter-rater agreement evaluated, Treatment response evaluation, Survival prediction	y	T1, T1c, T2, FLAIR	Previously proposed	BE; REG:rigid	n/a	n/a	n	y	BT	GBM	64 (14xN)	3	n	Manual, 2		VD; DSC
J	2016	N. Porz et al.	Fully Automated Enhanced Tumor Compartmentalization: Man vs. Machine Reloaded	PloS one		y	T1, T1c, T2, FLAIR	Previously proposed	n/a	n/a	n/a	n	n	BT	-	19	3	y	Manual, 2	supervision	DSC; PPV; Sen; VD; other
J	2015	E. Rios Velazquez et al.	Fully automatic GBM segmentation in the TCGA-GBM dataset: Prognosis and correlation with VASARI features	Scientific Reports		y	T1, T1c, T2, FLAIR	Previously proposed	BE; REG:rigid	n/a	5 min	Overall survival; 1 year survival	y	BT	GB	109	2 (TCIA, Clark et al. 2013)	y	Manual		VC; VR
J	2015	O. Maier et al.	Classifiers for Ischemic Stroke Lesion Segmentation: A Comparison Study	PloS one	Inter-rater agreement evaluated, Errors in preprocessing, Segmentation failure	both	T1 and/or DWI, FLAIR	Comparison	downsampling:ref; BE:ref; BFC:ref; IN:ref	n/a	n/a	n	n	S	S	37	3 (Meier et al. 2015)	n	Manual, 2		DSC; avgSSD; Hdff; Sen; PPV; ROC
J	2015	S. Roy et al.	Subject-Specific Sparse Dictionary Learning for Atlas-Based Brain MRI Segmentation	IEEE Journal of Biomedical and Health Informatics		y	T1, T2, PD, FLAIR	Sparse representation, Dictionary Learning	REG:rigid (FLIRT); BFC:N4; BE:Spectre	2.7GHz 12-core AMD	15 min	n	n	MS	MS	18; 122	3	n	Manual, 1		DSC; VD; TPR; FPR; avgSSD

For peer review only

J	2014	N. Porz et al.	Multi-Modal Glioblastoma Segmentation: Man versus Machine	PloS one		y	T1, T1c, T2, FLAIR	Previously proposed	n/a	n/a	n/a	n	n	BT	GBM	25	1 (BraTS'12); 3	y	Manual, 3	supervision	DSC; PPV; Sen; VE
J	2014	J. Gao et al.	Non-locally regularized segmentation of multiple sclerosis lesion from multi-channel MRI data	Magnetic Resonance Imaging		y	T1, T2, FLAIR	Other	REG:rigid	n/a	n/a	n	n	MS	MS	20	1 (MS GC MICCAI'08)	y	-		DSC; Spe; FNR; VD
J	2013	J. A. Maldjian et al.	Automated White Matter Total Lesion Volume Segmentation in Diabetes	American Journal of Neuroradiology	Visual validation of preprocessing	y	T1, FLAIR	Other	REG:toMNI; tissue maps:DARTEL + SPM8	n/a	n/a	n	n	WML	DM2	50 (+50 healthy)	3 (Diabetes Heart Study-Mind, Bowden et al. 2010)	n	Manual, 4	supervision, consensus	VC; Bland-Altman
J	2013	K. Nagenthiraja et al.	Automated Decision-Support System for Prediction of Treatment Responders in Acute Ischemic Stroke	Front. Neurol.	Intended clinical application	y	PWI, DWI	Previously proposed	REG:linear; reslicing:SPM8	I7 2.6GHz	31 sec	n	n	S	-	288	3 (I-KNOW); 3 (Remote Ischemic Perconditioning Study, Hougaard et al. 2013)	y	Manual, 4	majority	VC; VD
J	2011	S. D. Smart et al.	Validation of Automated White Matter Hyperintensity Segmentation	Journal of Aging Research	Inter-rater agreement evaluated	y	T1, FLAIR	Previously proposed	REG:SPM	n/a	n/a	n	n	WML	-	30	3 (Firbank et al. 2010)	n	Manual, 2		Jcc
J	2004	G. P. Mazzara et al.	Brain tumor target volume determination for radiation treatment planning through automated MRI segmentation	International Journal of Radiation Oncology*Biology*Physics	Inter-rater agreement evaluated; Segmentation failure	y	T1c, T2, PD	Previously proposed	REG	n/a	n/a	n	n	BT	G	11x2	3	y	Manual, 3		Acc
J	2003	S. Mehta et al.	Evaluation of voxel-based morphometry for focal lesion detection in individuals	NeuroImage		n	T1	Previously proposed	n/a	n/a	n/a	n	n	S	IFL	10	3 (Cognitive Neuroscience Patient Registry)	n	Manual, 1		VC; avgSSD; sdSSD; other
C	2017	S. Jain et al.	Unsupervised Framework for Consistent Longitudinal MS Lesion Segmentation	Medical Computer Vision and Bayesian and Graphical Models for Biomedical Imaging	Scan-rescan reproducibility	y	T1, FLAIR	Previously proposed	REG; BFC; IN:histogram matching	n/a	n/a	n	n	WML	MS	10; 12	3	y	Expert segmentation		TPR; FPR; DSC
C	2016	C. C. Benson et al.	Brain tumor segmentation from MR brain images using improved fuzzy c-means clustering and watershed algorithm	2016 International Conference on Advances in Computing, Communications and Informatics (ICACCI)		n	T1	Fuzzy C-Means, Watershed	denoising: median filter; BE: ref; contrast enhancement:ref	n/a	n/a	n	n	BT	GBM	90	3	n	Manual		DSC; TI
C	2013	N. Weiss et al.	Multiple Sclerosis Lesion Segmentation Using Dictionary Learning and Sparse Coding	Medical Image Computing and Computer-Assisted Intervention – MICCAI 2013		n	FLAIR	Sparse representation, Dictionary Learning	BE:BET; BFC:N4; IN: sqrt of largest L1 norm of all patches	2.4GHz 4GB RAM	5 min	n	n	MS	MS	20	1 (MS GC MICCAI'08)	y	-		DSC; TPR; PPV
C	2013	H. Wang and P.A. Yushkevich	Multi-atlas Segmentation without Registration: A Supervoxel-Based Approach	MICCAI 2013		y	T1, T1c, T2, FLAIR	Other	IN:histogram equalization + 0-1 range	2 GHz	few sec	n	n	BT	LGG; HGG	20 (25 synthetic)	1 (BraTS'12)	y	-		DSC
C	2009	D. García-Lorenzo et al.	Multiple Sclerosis Lesion Segmentation Using an Automatic Multimodal Graph Cuts	MICCAI 2009	Inter-rater agreement evaluated	y	T1, T2, PD	Expectation-Maximization Gausian mixture model, Graph Cut	BFC:ref; REG:ref; BE:ref	n/a	n/a	n	n	MS	MS	10	3	n/a	Manual, 5	majority	DSC
C	2006	J. Corso et al.	Multilevel Segmentation and Integrated Bayesian Model Classification with an Application to Brain Tumor Segmentation	MICCAI 2006		y	T1, T1c, FLAIR	Bayesian classification	REG:FSL; denoising:FSL; BE:FSL; IN:FSL	n/a	n/a	n	n	BT	GBM	30	3	n/a	Manual		Jcc
J	2019	J. Tong et al.	MRI brain tumor segmentation based on texture features and kernel sparse coding	Biomedical Signal Processing and Control		n	FLAIR	Sparse representation, Dictionary Learning	IN:0-225 range; denoising:Gaussian filtering; brain mask	4GHz 32GB RAM	38 sec	n	n	BT	LGG; HGG	50	1 (BraTS'12)	y	-		Jcc; DSC; Spe; Sen
J	2018	J. Tong et al.	Kernel sparse representation for MRI image analysis in automatic brain tumor segmentation	Frontiers of Information Technology & Electronic Engineering		y	T1, T1c, T2, FLAIR	K-Means, Sparse representation	IN:0-225 range; denoising:Gaussian filtering; brain mask	4GHz 32 GB RAM	40s	n	n	BT	LGG; HGG	384	1 (BraTS'15)	y	-		DSC; PPV; Sen; Kappa

For peer review only

J	2018	O. Ghribi et al.	Multiple sclerosis exploration based on automatic MRI modalities segmentation approach with advanced volumetric evaluations for essential feature extraction	Biomedical Signal Processing and Control		y	T1, T1c; T1, T2, FLAIR; T1, T2, PD, FLAIR	Support Vector Machine, Genetic Algorithm	BFC; REG:BET; BE: FSL; denoising: ADF; IN	n/a	n/a	n	n	MS	MS	70; 45; 10x4, 3x5, 1x6	1 (MS GC MICCAI'08); 1 (MSC'15); 3 (Habib-Bourguiba University Hospital)	y	Manual, 1&2		DSC; TPR; PPV; Jcc; TPR; FPR; FP; VD
J	2018	A. Vishnuvarthanan et al.	Development of a combinational framework to concurrently perform tissue segmentation and tumor identification in T1 - W, T2 - W, FLAIR and MPR type magnetic resonance brain images	Expert Systems with Applications		y	T1, T2, FLAIR, MPR	Fuzzy C-Means, Bacteria Foraging Optimization	IN: histogram equalization	I7 2.6GHz 8GB RAM	unclear	n	n	BT	T	4; 25	1 (BraTS'13); 2 (The Whole Brain Atlas);	y	Manual		DSC; Sen; Spe
J	2018	J. Jiang et al.	UBO Detector – A cluster-based, fully automated pipeline for extracting white matter hyperintensities	NeuroImage		y	T1, FLAIR	K-Nearest Neighbours	REG:SPM12; tissue maps:SPM12; REG:DARTEL; BE	2.2GHz 256GB RAM	15 min	n	y	WML	-	397; 1037 (subsample longitudinal)	3 (OATS, Sachdev et al. 2013); 3 (Sydney Memory and Ageing Study, Sachdev et al. 2010)	y	Manual, 2		DSC; TPR; FPR; Spe; Acc; ICC
J	2018	H. Li et al.	Fully convolutional network ensembles for white matter hyperintensities segmentation in MR images	NeuroImage		y	T1, FLAIR	Artificial neural networks	cropping; IN:Gaussian normalization	32 GB RAM	CPU: 60 sec GPU: 8 sec	n	y	WML	MS	170	1 (WMH Segmentation Challenge '17)	y	-		DSC; Hdff; avgVD; Sen; F1
J	2018	T. Kaur et al.	A joint intensity and edge magnitude-based multilevel thresholding algorithm for the automatic segmentation of pathological MR brain images	Neural Comput & Applic	No preprocessing	n	FLAIR	Thresholding, Particle Swarm Optimization	IN:0-255 range; BE:Otsu's thresholding	3.1GHz i5 4GB RAM	5 sec	n	n	BT	LGG	10; 20; 10	1 (BraTS'12); 1 (BraTS'15); 3 (Advance Diagnostic Centre, Ludhiana, Punjab, India)	y	Manual,1		DSC; Jcc; other
J	2018	L. Rundo et al.	NeXt for neuro-radiosurgery: A fully automatic approach for necrosis extraction in brain tumor MRI using an unsupervised machine learning technique	International Journal of Imaging Systems and Technology		n	T1c	Fuzzy C-Means	morphological operations on GTV	2.8GHz i7 16BG RAM	< 1 sec	n	n	NEC	-	32	3	n	Manual,1		DSC; Jcc; Sen; Spe; MAD; Hdff; other
J	2018	M. Soltaninejad et al.	Supervised learning based multimodal MRI brain tumour segmentation using texture features from supervoxels	Computer Methods and Programs in Biomedicine		y	T1, T1c, T2, FLAIR, DTI	Random Forest	Eddy curent correction:FSL; BE:BET; REG:SPM12; IN:histogram matching + dynamic range normalization	n/a	n/a	n	n	BT	LGG; HGG	11; 30	1 (BraTS'13); 3	y	Manual,1		PPV; Sen; DSC; other
J	2018	E. A. AlBadawy et al.	Deep learning for segmentation of brain tumors: Impact of cross-institutional training and testing	Medical Physics	Minimal preprocessing	y	T1, T1c, FLAIR	Artificial neural networks	REG:FLIRT	n/a	n/a	n	n	BT	GB	44	2 (TCIA, Clark et al. 2013)	y	Manual, 3	supervision	DSC; avgHdff; avgVD; Sen; PPV
J	2018	T. Kaur et al.	A novel fully automatic multilevel thresholding technique based on optimized intuitionistic fuzzy sets and tsallis entropy for MR brain tumor image segmentation	Australas Phys Eng Sci Med	No preprocessing	n	FLAIR	Thresholding, Particle Swarm Optimization	none	3.10GHz i5 4GB RAM	< 5 sec	n	n	BT	LGG; HGG	8; 8	1 (BraTS'12); 3	y	Manual		DSC; Jcc; other
J	2018	M. Salem et al.	A supervised framework with intensity subtraction and deformation field features for the detection of new T2-w lesions in multiple sclerosis	NeuroImage: Clinical		y	T1, PD, T2, FLAIR	Logistic Regression	BE:ROBEX; BFC:N4; IN:histogram matching; REG:ITKv4Demons	n/a	n/a	n	y	MS	MS	60x2	3 (Vall d'Hebron Hospital)	n	Semiautomatic		DSC; VC
J	2018	E. B. Shimol et al.	Computer-based radiological longitudinal evaluation of meningiomas following stereotactic radiosurgery	Int J CARS		n	T1c	Active Contour	REG:SPM; IN:histogram normalization	n/a	n/a	n	n	BT	MEN	28	3	n	Manual, 1		DSC; Sen; maxSD; avgSD
J	2018	Z. Zhao et al.	Automated glioma detection and segmentation using graphical models	PloS one		y	T1, T1c, T2, FLAIR	Conditional Random Field	n/a	n/a	n/a	n	n	BT	LGG; HGG	30; 274; 161	1 (BraTS'13); 1 (BraTS'15); 3 (Henan Provincial People's Hospital)	y	Manual, 1		DSC; Hdff; VC; Spe; Sen
J	2018	M. M. Cheriyan et al.	Blind source separation with mixture models – A hybrid approach to MR brain classification	Magnetic Resonance Imaging		y	T1, T2, FLAIR	Gaussian Mixture Model, Particle Swarm Optimization	n/a	n/a	n/a	n	n	BT, MS, S	BT, MS, HEM	38 + 22 healthy; 152	3 (Medall Diagnostics, Kochi)	n	Manual, 3		Jcc; Sen; Spe; Acc
J	2018	M. B. Naceur et al.	Fully Automatic Brain Tumor Segmentation using End-To-End Incremental Deep Neural Networks in MRI images	Computer Methods and Programs in Biomedicine		y	T1, T1c, T2, FLAIR	Artificial neural networks	IN:1-99% clipping + 0u1v	GPU	22 sec	n	n	BT	-	285	1 (BraTS'17)	y	-		DSC; Sen; Spe; Hdff
J	2018	M. K. Abd-Ellah et al.	Two-phase multi-model automatic brain tumour diagnosis system from magnetic resonance images using convolutional neural networks	EURASIP Journal on Image and Video Processing	Detection followed by segmentation	n	T2	Artificial neural networks	resizing and RGB conversion	n/a	n/a	n	n	BT	LGG; HGG	19; 30	1 (BraTS'13)	y	-		Sen; Spe; Acc; PPV; NPV; DSC
J	2018	J. K. Boldsen et al.	Better Diffusion Segmentation in Acute Ischemic Stroke Through Automatic Tree Learning Anomaly Segmentation	Front. Neuroinform.		n	DWI	Decision Tree	IN: division by mean of contralateral hemisphere; smoothing:Gaussian kernel; morphological operations	n/a	n/a	n	n	S	S	108	3 (I-KNOW)	y	Manual, 1		DSC

For peer review only

J	2018	R. Guerrero et al.	White matter hyperintensity and stroke lesion segmentation and differentiation using convolutional neural networks	NeuroImage: Clinical		y	T1, FLAIR	Artificial neural networks	REG:FLIRT; reslicing; tissue maps; IN:0u1v + clipping (3stdev)	n/a	n/a	n	n	WML, S	S	167	3 (Brain Research Imaging Centre of Edinburgh)	n* various protocols	Semiautomatic		DSC; VD; other
J	2018	T. Zhan et al.	A Glioma Segmentation Method Using CoTraining and Superpixel-Based Spatial and Clinical Constraints	IEEE Access		y	T1, T1c, T2, FLAIR	Support Vector Machine, Sparse representation	BFC:N4; IN: 0-255 range	n/a	n/a	n	n	BT	LGG; HGG	30	1 (BraTS'12); 1 (BraTS'13)	y	-		DSC; PPV; Sen
J	2018	S. Cui et al.	Automatic Semantic Segmentation of Brain Gliomas from MRI Images Using a Deep Cascaded Neural Network	Journal of Healthcare Engineering		y	T1, T1c, T2, FLAIR	Fuzzy C-Means, Artificial neural networks	BFC:N4; IN:1-99% clipping + 0u1v	Intel E5 GTX 1080	2 sec	n	n	BT	LGG; HGG	274	1 (BraTS'15)	y	-		DSC; PPV; Sen
J	2018	S. Bonte et al.	Machine learning based brain tumour segmentation on limited data using local texture and abnormality	Computers in Biology and Medicine		y	T1c, FLAIR	Random Forest	REG:SPM12; tissue maps: SPM; BFC:SPM12	n/a	n/a	n	n	BT	LGG; HGG	30; 285; 257	1 (BraTS'17); 1 (BraTS'17); 3 (Ghent University Hospital)	y	n/a		DSC
J	2018	M. J. Fartaria et al.	Partial volume-aware assessment of multiple sclerosis lesions	NeuroImage: Clinical		y	T1, FLAIR	Other	REG:rigid (ELASTIX); BFC:N4; BE:in house; tissue maps:in house	n/a	n/a	n	n	MS	early MS	39	3	n	Manual, 2	consensus	DSC; FPR; Detection Rate
J	2018	C. Ma et al.	Concatenated and Connected Random Forests With Multiscale Patch Driven Active Contour Model for Automated Brain Tumor Segmentation of MR Images	IEEE Transactions on Medical Imaging		y	T1, T1c, T2, FLAIR	Random Forest, Active Contour	n/a	n/a	5 min	n	n	BT	-	n/a	1 (BraTS'15); 1 (TCIA, Bakas et al. 2017)	y	-		DSC; Sen; Spe
J	2018	I. Razzak et al.	Efficient Brain Tumor Segmentation with Multiscale Two-Pathway-Group Conventional Neural Networks	IEEE Journal of Biomedical and Health Informatics		y	T1, T1c, T2, FLAIR	Artificial neural networks	IN:1-99% clipping + 0u1v + 0-1 range; BFC:N4	n/a	3 min	n	n	BT	-	285 + 110;	1 (BraTS'13); 1 (BraTS'15)	y	-		DSC; Sen; Spe
J	2018	S. Hussain et al.	Segmentation of glioma tumors in brain using deep convolutional neural network	Neurocomputing		y	T1, T1c, T2, FLAIR	Artificial neural networks	BFC:N4; IN: 1-99% clipping + 0u1v	i7 16GB RAM	5 min	n	n	BT	LGG; HGG	30; 274	1 (BraTS'13); 1 (BraTS'15)		-		DSC; Sen; Spe
J	2018	S. Banerjee et al.	Automated 3D segmentation of brain tumor using visual saliency	Information Sciences	Inter-rater agreement evaluated	y	T1c, T2, FLAIR	Other, Grow Cut	BRATS only	I7 3.4GHz 16GB RAM	2 min	n	n	BT	LGG; HGG	30	1 (BraTS'13)	y	-		DSC; Hdff; other
J	2018	E. Binaghi et al.	Meningioma and peritumoral edema segmentation of preoperative MRI brain scans	Computer Methods in Biomechanics and Biomedical Engineering: Imaging & Visualization		y	T1c, FLAIR	Sparse representation, Graph Cut	REG:Iterative Closest Point; contrast enhancement:log transform	2.26GHz	145 sec	n	n	BT	T	15	3	n	Manual, 3	majority	DSC; Jcc; VE
J	2018	C. Qin et al.	A large margin algorithm for automated segmentation of white matter hyperintensity	Pattern Recognition		y	T1, FLAIR	Other	REG:FLIRT; tissue maps:ref; IN:ref	3.6GHz 16GB RAM	16 sec	n	n	WML	-	88	3 (Brain Research Imaging Centre of Edinburgh)	n	Semiautomatic		DSC; Acc; PPV; Sen; Bland-Altman; VC; other
J	2018	Y. Ding et al.	Multi-modal brain tumor image segmentation based on SDAE	International Journal of Imaging Systems and Technology		y	T1, T1c, T2, FLAIR	Artificial neural networks	contrast enhancement:top-hat transform	12 GB RAM	n/a	n	n	BT	-	95 ?	1 (BraTS'15)	y	-		DSC; Jcc
J	2018	A. M. Valcarcel et al.	MIMoSA: An Automated Method for Intermodal Segmentation Analysis of Multiple Sclerosis Brain Lesions	Journal of Neuroimaging		y	T1, T2, FLAIR, PD	Logistic Regression	REG:rigid; BFC:N3; brain tissue extraction:BE:SPECTRE + thresholding; IN:0u1v or WhiteStripe	n/a	22 min	n	n	MS	MS	94; 19xn	1 (MSC'15); 3 (Johns Hopkins Hospital)	y	Manual, 1		DSC; ROC; VC
J	2018	A. R. Raju et al.	Bayesian HCS-based multi-SVNN: A classification approach for brain tumor segmentation and classification using Bayesian fuzzy clustering	Biocybernetics and Biomedical Engineering		y	T1, T1c, T2, FLAIR	Fuzzy Clustering	n/a	2GB RAM	n/a	benign, malignant	n	BT	LGG; HGG	30	1 (BraTS'12)	y	-		Sen
J	2018	A. Essadike et al.	Brain tumor segmentation with Vander Lugt correlator based active contour	Computer Methods and Programs in Biomedicine		n/a	n/a	Active Contour, Other	n/a	n/a	<1 sec	n	n	BT	LGG; HGG	45 + 65 synthetic; 35	1 (BraTS'12); 1 (BraTS'13)	y	-		DSC; Hdff; Spe; Sen
J	2018	Y. Wang et al.	Voxel-based automated detection of focal cortical dysplasia lesions using diffusion tensor imaging and T2-weighted MRI data	Epilepsy & Behavior		y	T2, DTI	Other	BE:FSL; IN; REG; tissue maps; BFC; resampling	n/a	n/a	n	n	FCD		12	3 (Sanbo Brain Hospital Capital Medical University)	n	Manual, 1		ROC; FPR
J	2018	A. Pinto et al.	Hierarchical brain tumour segmentation using extremely randomized trees	Pattern Recognition		y	T1, T1c, T2, FLAIR	Decision Tree	BFC:N4; IN: histogram matching	n/a	n/a	n	n	BT	LGG; HGG	65	1 (BraTS'13)	y	-		DSC; PPV; Sen
J	2018	G. B. Praveen et al.	Ischemic stroke lesion segmentation using stacked sparse autoencoder	Computers in Biology and Medicine		y	T1, T2, DWI, FLAIR	Artificial neural networks	BFC:N4; IN:0u1v + whitening; resizing	3.5GHz 32 GB RAM	10 min	n	n	S	S	28	1 (ISLES'15)	y	-		DSC; PPV; Sen; Spe; Acc; ROC

For peer review only

J	2018	J. Knight et al.	Voxel-Wise Logistic Regression and Leave-One-Source-Out Cross Validation for white matter hyperintensity segmentation	Magnetic Resonance Imaging		n	FLAIR	Logistic Regression	BFC:SPM12; REG:affine + non-linear; resmpling:trilinear; BE:brain tissue probability maps; IN:histogram matching + 0-1 range	n/a	n/a	n	y	WML	-	96; 110	1 (WMH Segmentation Challenge '17); 1 (MSC'15); 1 (MS Lesion Segmentation Challenge '16)	y	-		DSC; PPV; Sen
J	2018	S. Amiri et al.	Tree-based Ensemble Classifier Learning for Automatic Brain Glioma Segmentation	Neurocomputing		y	T1c, T2, FLAIR	Decision Tree, Bayesian classification	BFC:N4; IN:histogram linear transformation	n/a	n/a	n	n	BT	HGG	50	1 (BraTS'15)	y	-		DSC
J	2018	J. Liu et al.	A Cascaded Deep Convolutional Neural Network for Joint Segmentation and Genotype Prediction of Brainstem Gliomas	IEEE Transactions on Biomedical Engineering		n	T1	Artificial neural networks	resizing; cropping	n/a	n/a	prediction of H3 K27M mutation	n	BT	T; LGG, HGG	55; 280	1 (BraTS'17); 3	y	Manual, 2		DSC; SD
J	2018	S. Sasikanth and S. Suresh Kumar	Glioma tumor detection in brain MRI image using ANFIS-based normalized graph cut approach	International Journal of Imaging Systems and Technology		n/a	n/a	Graph Cut	n/a	2.4GHz 2GB RAM	n/a	normal, abnormal	n	BT	LGG; HGG	100	1 (BraTS'15)	y	-		DSC; Acc; Sen; Spe
J	2018	J. V. Manjón et al.	MRI white matter lesion segmentation using an ensemble of neural networks and overcomplete patch-based voting	Computerized Medical Imaging and Graphics		n	FLAIR	Thresholding, Artificial neural networks	denoising:non-local means; REG:affine; BFC:SPM12; BE:SPM12; IN:division by median and squared	i7 16GB RAM	3 min	n	n	WML	-	128;20	1 (MS GC MICCAI'08); 3 (AIBL, Ellis et al., 2009)	y	Manual, 1		DSC; Spe; Sen; VD; VC
J	2018	M. F. Rachmadi et al.	Segmentation of white matter hyperintensities using convolutional neural networks with global spatial information in routine clinical brain MRI with none or mild vascular pathology	Computerized Medical Imaging and Graphics	Inter-rater agreement evaluated	y	T1, FLAIR	Artificial neural networks	REG:rigid(FLIRT); BE:opti-BET + morphological operations; tissue maps:FAST; IN:histogram matching + 0u1v	3.4GHz GPU 8GB RAM	9 sec	n	n	WML	-	20x3; 268 (with only Fazekas scores)	2 (ADNI)	y	Semiautomatic		DSC; PPV; Sen; VC; VD
J	2017	A. R. Abdulraqeb et al.	An Automated Method for Segmenting Brain Tumors on MRI Images	Biomed Eng		n	T1c	Thresholding	n/a	n/a	n/a	n	n	BT	-	12; 44	3	y	Expert segmentation		DSC; Jcc; Sen; Spe
J	2017	S. Cui et al.	Brain tumor Segmentation using fully convolutional networks	Journal of Medical Imaging and Health Informatics		y	T1, T1c, T2, FLAIR	Artificial neural networks	BFC:N4; IN:mode & deviation scaling + 0-255 range	GeForce GTX 1080; 2 Intel e5 2603 CPUs	<30 sec	n	n	BT	HGG	220	1 (BraTS'15)	y	-		DSC; PPV; Sen
J	2017	Y. Zhuge et al.	Brain tumor segmentation using holistically nested neural networks in MRI images	Medical Physics		y	T1, T1c, T2, FLAIR	Artificial neural networks	BFC:N4; IN:histogram matching	2.66GHz 32 GB RAM	30 sec	n	n	BT	HGG	20; 10	1 (BraTS'13); 3	y	Manual, 1		DSC; Sen
J	2017	L. Chen et al.	Fully automatic acute ischemic lesion segmentation in DWI using convolutional neural networks	NeuroImage: Clinical		n	DWI	Artificial neural networks	resampling:homogeneous linear; IN:0u1v	i7 16GB RAM	<1 sec	n	n	S	S	741	3	y	Expert segmentation		DSC; Detection Rate; other
J	2017	M. Dadar et al.	Performance comparison of 10 different classification techniques in segmenting white matter hyperintensities in aging	NeuroImage	Inter-rater agreement evaluated	y	T1, T2, PD, FLAIR	Comparison	denoising:MINCnlm; BFC:MINCnu_estimate; IN:histogram matching & 0-100 range; REG; BE	i7 2.6GHz 20GB RAM	0.11 – 3021 sec	n	y	WML	CI	70; 32; 53; 46	2 (ADNI); 2 (ADNI2/GO); 2 (NACC) 3 (Davis Alzheimer's Disease Center, Hinton et al. 2010)	y	Manual, 2		DSC; ICC; Sen; PPV; OER; DER
J	2017	K. Murphy et al.	Automatic quantification of ischemic injury on diffusion-weighted MRI of neonatal hypoxic ischemic encephalopathy	NeuroImage: Clinical	Inter-rater agreement evaluated	y	ADC, DWI	Random Forest	BE:BET2; BFC:N4; IN:histogram matching	n/a	n/a	n	n	S	S	74	3 (Wilhelmina Children's Hospital, University Medical Center Utrecht)	y	Manual, 2		ROC: Sen; Spe; F1; VC
J	2017	S. Damangir et al.	Reproducible segmentation of white matter hyperintensities using a new statistical definition	Magnetic Resonance Materials in Physics, Biology and Medicine		y	T1, T2, PD, FLAIR	Other	REG:FLIRT; BE:BET; BFC:N3; tissue maps:FSL-FAST	n/a	n/a	n	y	WML	CI	119	3 (Kings Health Partners-Dementia Case Register)	n	Manual, 1		FNR; VC; DSC
J	2017	M. Soltaninejad et al.	Automated brain tumour detection and segmentation using superpixel-based extremely randomized trees in FLAIR MRI	International Journal of Computer Assisted Radiology and Surgery		n	FLAIR	Decision Tree	n/a	n/a	n/a	n	n	BT	-	19; 30	1 (BraTS'12); 3	y	Manual, 1		DSC; Sen; PPV; other
J	2017	M. Dadar et al.	Validation of a Regression Technique for Segmentation of White Matter Hyperintensities in Alzheimer's Disease	IEEE Transactions on Medical Imaging		y	T1, T2, PD, FLAIR	Linear regression, Thresholding	denoising:non-local means; BFC:N3; IN:histogram matching	i3 3.3GHz	1.6 sec	n	n	WML	-	80; 40; 10	2 (ADNI2/GO); 3 (Davis Alzheimer's Disease Center, Hinton et al. 2010) 3 (PREVENT-AD, Tremblay-Mercier et al. 2014)	y	Semiautomatic; Manual, 2	union; supervision	ICC; DSC; FPR; Sen
J	2017	Z. Li et al.	Low-Grade Glioma Segmentation Based on CNN with Fully Connected CRF	Journal of Healthcare Engineering		n	FLAIR	Artificial neural networks, Conditional Random Field	BE:BrainSuite; BFC:BrainSuite	Intel Xeon 2.4GHz Nvidia Quadro	2-10 min	n	n	BT	LGG	160	3 (Shanghai Huashan Hospital)	n	Manual, 2		DSC; PPV; Sen
J	2017	A. Galimzianova et al.	Locally adaptive magnetic resonance intensity models for unsupervised segmentation of multiple sclerosis lesions	Journal of Medical Imaging	Inter-rater agreement evaluated	y	T1, T2, FLAIR	Gaussian Mixture Model, Markov Random Field	REG:ref; BE:ref; BFC:ref; resampling	n/a	n/a	n	n	MS	MS	30	3	n	Semiautomatic		DSC; VC
J	2017	C. Bowles et al.	Brain lesion segmentation through image synthesis and outlier detection	NeuroImage: Clinical		y	T1, FLAIR	Gaussian Mixture Model, Support Vector Machine	REG:rigid+free form (MIRTK); BFC:N4; BE:pincram; tissue maps:MALPEM; IN:ref	n/a	n/a	n	n	WML	SVD	127	3 (Brain Research Imaging Centre of Edinburgh)	n – different protocols	Semiautomatic		DSC; avgSD; Hdff; PPV; Sen; ICC; Bland-Altman

For peer review only

J	2017	O. Ghribi et al.	An Advanced MRI Multi-Modalities Segmentation Methodology Dedicated to Multiple Sclerosis Lesions Exploration and Differentiation	IEEE Transactions on NanoBioscience	Inter-rater agreement evaluated	y	T1c, FLAIR	Gaussian Mixture Model, Thresholding	REG:rigid; resampling:spline interpolation; BFC; BE:FSL; denoising: ADF; IN	i5 1.8GHz	2 min	n	n	MS	-	70 + 50 healthy; 45; 21x4 (or more)	1 (MS GC MICCAI'08); 1 (MSC'15); 3 (Habib-Bourguiba University Hospital)	y	Manual, 1		DSC; Jcc; PPV; TPR; FPR; SD; VD; DE; OE
J	2017	K. Kamnitsas et al.	Efficient multi-scale 3D CNN with fully connected CRF for accurate brain lesion segmentation	Medical Image Analysis		y	n/a	Artificial neural networks	BE:ROBEX; resampling; REG:affine; IN:0u1v	NVIDIA GTX Titan X GPU 3GB RAM	3 min	n	y	TBI; BT; S	-	61; 384; 64	1 (ISLES'15); 1 (BraTS'15); 3 (Neurosciences Critical Care Unit at Addenbrooke's Hospital, Cambridge, UK)	y	n/a		DSC; Sen; PPV; avgSD; Hdff
J	2017	V. Rajinikanth et al.	Entropy based segmentation of tumor from brain MR images – a study with teaching learning based optimization	Pattern Recognition Letters		y	T1c, T2, FLAIR	Level Set	BE:thresholding; Multilevel thresholding + teaching-learning based optimization – Shannon entropy optimization	n/a	n/a	n	n	BT	-	15?	1 (BraTS'12)	y	-		DSC; Jcc; FPR; FNR; PPV; F-score; Sen; Spe; Acc; other
J	2017	S. Abbasi and F. Tajeripour	Detection of brain tumor in 3D MRI images using local binary patterns and histogram orientation gradient	Neurocomputing		y	T1, T1c, T2, FLAIR	Thresholding, Random Forest	BFC:N4; IN:histogram matching	4GHz 32GB RAM	1900 sec	n	n	BT	LGG; HGG	30 (+50 simulated)	1 (BraTS'13)	y	-		DSC; Jcc
J	2017	F. Binczyk et al.	MiMSeg - an algorithm for automated detection of tumor tissue on NMR apparent diffusion coefficient maps	Information Sciences		y	DWI (ADC), FLAIR	Expectation-Maximization Gausian mixture model, K-Means	BE:BET; tissue maps:Otsu's thresholding	n/a	n/a	n	n	BT	GBM	17x7	3 (Maria Sklodowska-Curie Memorial Cancer Center and Institute of Oncology Gliwice Branch, Gliwice, Poland)	n	Manual, 1		DSC; Sen; Spe; PPV; NPV
J	2017	M. Rincón et al.	Improved Automatic Segmentation of White Matter Hyperintensities in MRI Based on Multilevel Lesion Features	Neuroinformatics	Inter-rater agreement evaluated; Visual inspection of preprocessing	y	T1, FLAIR	Thresholding, Support Vector Machine	REG:rigid; BFC: SPM8; tissue maps:Freesurfer; IN:Gaussian fit	n/a	n/a	n	n	WML	S, MCI	28	3 (Selnes et al. 2015)	n	Manual, 3	majority	DSC; other
J	2017	M. Havaei et al.	Brain tumor segmentation with Deep Neural Networks	Medical Image Analysis		y	T1, T1c, T2, FLAIR	Artificial neural networks	BFC:N4; IN:1-99% clipping + 0u1v	GPU	~3 min	n	n	BT	LGG; HGG	65	1 (BraTS'13)	y	-		DSC; Sen; Spe
J	2017	T. Zhan et al.	Brain Tumor Segmentation Using Deep Belief Networks and Pathological Knowledge	CNS & Neurological Disorders-Drug Targets		y	T1, T1c, T2, FLAIR	Logistic Regression, Graph Cut, Artificial neural networks, Markov Random Field	n/a	n/a	n/a	n	n	BT	LGG; HGG	10 hgg + N LGG (+20 synthetic)	1 (BraTS'12); 1 (BraTS'13)	y	-		DSC; Sen; PPV; F-score
J	2017	E. Kellner et al.	Automated Infarct Core Volumetry Within the Hypoperfused Tissue: Technical Implementation and Evaluation	Journal of Computer Assisted Tomography		y	PWI, DWI	Other	BE; hemisphere extraction	n/a	1 min	n	n	S	S	30	1 (ISLES'15)	y	-		TPR; FPR; FDR; DSC
J	2017	S. Adler et al.	Novel surface features for automated detection of focal cortical dysplasias in paediatric epilepsy	Neuroimage: Clinical	Manual corrections of preprocessing	y	T1, FLAIR	Artificial neural networks	REG:Freesurfer: resampling; IN; BFC; BE:ref	n/a	n/a	n	n	FCD	EP	22 + 28 healthy	3 (Great Ormond Street Hospital)	n	Expert segmentation		ROC; Sen
J	2017	P. Sivakumar and P. Ganeshkumar	An efficient automated methodology for detecting and segmenting the ischemic stroke in brain MRI images	International Journal of Imaging Systems and Technology		n/a	n/a	Graph Cut	enhancement: histogram equalization; Gabor transform	2.4GHz Core-2-Duo 4GB RAM	n/a	normal, abnormal	n	S	S	20 +25 healthy	1 (ISLES)	y	-		Sen; Spe; Acc; PPV; NPV; VC
J	2017	E. Ilunga–Mbuyamba et al.	Automatic selection of localized region-based active contour models using image content analysis applied to brain tumor segmentation	Computers in Biology and Medicine		n/a	n/a	K-Nearest Neighbours, Random Forest, Active Contour,	n/a	n/a	n/a	n	n	BT	LGG, HGG	21	1 (BraTS'12); 3 (University Hospital of Leipzig, Germany)	y	Manual, 1		DSC; Hdff
J	2017	S. Valverde et al.	Improving automated multiple sclerosis lesion segmentation with a cascaded 3D convolutional neural network approach	NeuroImage		y	T1, T2, PD, FLAIR	Artificial neural networks	BE:BET; BFC:N3; reg:SPM	32 GB RAM	n/a	n	y	MS	MS	45; 60	1 (MS GC MICCAI'08); 3 (Hospital Vall d'Hebron, Barcelona, Spain)	y	Semiautomatic		VD; TPR; FPR; DSC; PPV
J	2017	A. S. Keçeli et al.	A GPU-Based Approach for Automatic Segmentation of White Matter Lesions	IETE Journal of Research		n	FLAIR	Fuzzy C-Means, Region Growing	BE:morphological/watershed/ACM; enhancement: histogram equalization	GPU	<1 sec; ~5-10 sec	n	n	WML	-	10	3 (Hacettepe University Medical School Hospital)	n	Expert segmentation		SI; TPR; FPR
J	2016	B. H. Menze et al	A generative probabilistic model and discriminative extensions for brain lesion segmentation – with application to tumor and stroke	IEEE Trans Med Imaging		y	T1, T1c, T2, FLAIR	Markov Random Field, Watershed , Expectation-Maximization Gausian mixture model,	BFC:polynomial spline model;IN:linear template matching	n/a	n/a	n	n	BT; S	LGG; HGG	45; 18	1 (BraTS'12); 1 (BraTS'13)	y	-		DSC
J	2016	M. Strumia et al.	White Matter MS-Lesion Segmentation Using a Geometric Brain Model	IEEE Transactions on Medical Imaging		y	T1, FLAIR	Expectation-Maximization Gausian mixture model	REG:ITK; BE:BET; BFC:ref	n/a	3-7 hrs	n	n	MS	MS	42; 20	1 (MS GC MICCAI'08); 3	y	Manual, 1&2		VD; avgSD; TPR; FPR; DSC
J	2016	L. Griffanti et al.	BIANCA (Brain Intensity AbNormality Classification Algorithm): A new tool for automated segmentation of white matter hyperintensities	NeuroImage	Scan-rescan reproducibility	y: flexible to the number of input sequences	T1, FLAIR	K-Nearest Neighbours	BE: BET; REG:FLIRTIN: variance scaling	3GHz Intel Xeon	12-13 min	n	y	WML	neurodegenerative and vascular disorders	85; 474 + 20 longitudinal	3 (OPTIMA, Zamboni et al., 2013); 3 (OXVASC, Rothwell et al., 2004)	y	Manual, 3		DSC; FPR; FNR; DER; OER; ICC; Bland-Altman
J	2016	M. Cabezas et al.	Improved Automatic Detection of New T2 Lesions in Multiple Sclerosis Using Deformation Fields	American Journal of Neuroradiology		n	T1, PD; FLAIR	Thresholding	BE:BET2; BFC:N4; IN:histogram matching	n/a	n/a	n	n	MS	CIS; early MS	36x2	3	n	Semiautomatic		avgSD; DSC; VO; other
J	2016	Z. Karimaghaloo et al.	Adaptive multi-level conditional random fields for detection and segmentation of small enhanced pathology in medical images	Mecidal image Analysis		y	T1, T1c, T2, PD, FLAIR	Conditional Random Field	BE:BET; BFC:N3; IN: histogram matching	n/a	n/a	n	n	MS	-	1760; 2770	3	y	Manual, 2	consensus	Sen; FDR
J	2016	M. Ghafoorian et al.	Automated detection of white matter hyperintensities of all sizes in cerebral small vessel disease: Automated detection of white matter hyperintensities of all sizes	Medical Physics		y	T1, FLAIR	Random Forest, Boosting	REG: FLIRT; BE:BET; BFC:FSL-FAST; IN:histogram matching	n/a	n/a	n	n	WML	SVD	503	3 (RUNDMC, van Norden et al. 2011)	n	Manual, 1-3	supervision	FROC
J	2016	Y. Liu et al.	Automatic metastatic brain tumor segmentation for stereotactic radiosurgery applications	Physics in Medicine and Biology		n	T1c	Thresholding, Active Contour	BE:ROBEX	E3-1505M CPU 32GB RAM	<7 min	n	n	MET	-	15?	3 (University of Texas Southwestern Medical Center)	y	Manual, 1		DSC; SI; Hdff; avgSSD; sdSSD; other

For peer review only

J	2016	T. Brosch et al.	Deep 3D Convolutional Encoder Networks With Shortcuts for Multiscale Feature Integration Applied to Multiple Sclerosis Lesion Segmentation	IEEE Transactions on Medical Imaging		y	T1, T2, PD, FLAIR	Artificial neural networks	BE:BET; IN:0-1 range; REG:rigid; cropping	GeForce GTX 780	<1 sec	n	n	MS	-	43; 21; 195x2+n (377)	1 (MS GC MICCAI'08); 1 (ISBI'15); 3	y	Semiautomatic		DSC; VD; TPR; FPR
J	2016	D. Pustina et al.	Automated segmentation of chronic stroke lesions using LINDA: Lesion identification with neighborhood data analysis	Human Brain Mapping	Segmentation failure (low DSC)	n	T1	Random Forest	denoising:edge-preserving anisotropic algorithm; BFC:N4; BE:antsBrainExtraction	Xeon E4-2450, 2.1GHz 25GB RAM	3 hrs	n	n	S	S	60; 45	3	y	Manual, 1		DSC; Hdff; Sen; PPV; VC; other
J	2016	M. Goetz et al.	DALSA: Domain Adaptation for Supervised Learning From Sparsely Annotated MR Images	IEEE Transactions on Medical Imaging		y	T1, T1c, FLAIR, DTI	Random Forest	REG:rigid; BE; IN	GeCONiI	70 sec	n	y	BT	HGG	19; 30	1 (BraTS'13); 3	y	Manual, 1		DSC; Sen; Spe
J	2016	K. Thiruvenkadam and N. Perumal	Fully automatic method for segmentation of brain tumor from multimodal magnetic resonance images using wavelet transformation and clustering technique	International Journal of Imaging Systems and Technology		y	T1c, T2, FLAIR	Fuzzy C-Means, Discrete Wavelet Transform	sharpening:Haar WT	Intel Pentium Core Duo 1.6 GHz 512 MB RAM	2-3 min	n	n	BT	LGG; HGG	30	1 (BraTS'12)	y	-		DSC; Sen; PPV; Kappa
J	2016	R. Mechrez et al.	Patch-based Segmentation with Spatial Consistency: Application to MS Lesions in Brain MRI	Journal of Biomedical Imaging		y	T1, T2, FLAIR	K-Nearest Neighbours, Thresholding	subsampling; IN: histogram matching; BFC:N4; BE:BrainSuite13; tissue maps	i7 3.4GHz 8GB RAM	4 min	n	n	MS	-	65	1 (MS GC MICCAI'08)	y	-		TPR; PPV; DSC
J	2016	P. Korfiatis et al.	Automated Segmentation of Hyperintense Regions in FLAIR MRI Using Deep Learning	Tomography	Inter-rater agreement evaluated	n	FLAIR	Artificial neural networks	BE:atlas-based; BFC:N4; IN:histogram matching	NVIDIA K2	1 hr	n	n	BT	LGG; HGG	186; 135	1 (BraTS'15); 3		Manual, 3		DSC; Jcc; FPR; TPR
J	2016	A. M. Pagnozzi et al.	Automated, quantitative measures of grey and white matter lesion burden correlates with motor and cognitive function in children with unilateral cerebral palsy	NeuroImage: Clinical		y	T1, T2	Expectation-Maximization, Markov Random Field, Thresholding	BFC:N4; REG:affine; denoising:ADF; BE:in-house; tissue probability maps reg:free-form	n/a	n/a	n	n	CP	-	125	3 (University of Queensland Cerebral Palsy and Research Rehabilitation Centre)	y	Manual		Sen; Spe; Acc; FPR; FNR
J	2016	X. Qu et al.	Positive Unanimous Voting Algorithm for Focal Cortical Dysplasia Detection on Magnetic Resonance Image	Front. Comput. Neurosci		n	T1	Bayesian classification, Other	IN:histogram matching + 0-225 range; BE:BET; BFC:EM; resampling; tri-linear interpolation; REG:rigid; masking	n/a	n/a	n	n	FCD	-	10 + 31 controls	3 (Ghent University Hospital)	n	Manual, 1		TPR; FPR; F-score
J	2016	S. Banerjee et al.	A Novel GBM Saliency Detection Model Using Multi-Channel MRI	PloS one		y	T1c T2, FLAIR	Other	n/a	I7 3.4GHz 16GB RAM	n/a	n	n	BT	LGG; HGG	30 +50 simulated	1 (BraTS'12)	y	-		ROC
J	2016	L. Zhao and K. Jia	Multiscale CNNs for Brain Tumor Segmentation and Diagnosis	Computational and Mathematical Methods in Medicine		y	T1, T1c, T2, FLAIR	Artificial neural networks	n/a	n/a	n/a	n	n	BT	LGG; HGG	30 +50 simulated	1 (BraTS'13)	y	-		DSC
J	2016	P. G. L. Freire and R. J. Ferrari	Automatic iterative segmentation of multiple sclerosis lesions using Student's t mixture models and probabilistic anatomical atlases in FLAIR images	Computers in Biology and Medicine	Inter-rater agreement evaluated	n	FLAIR	Other	denoising:non-local means; BFC:N4; REG:non-rigid	i7 16GB RAM	~20 min	n	n	MS	-	5x4/5	1 (MSC'15)	y	-		DSC; Sen; FPR; VD; VC
J	2016	S. Pereira et al.	Brain Tumor Segmentation Using Convolutional Neural Networks in MRI Images	IEEE Transactions on Medical Imaging		y	T1, T1c, T2, FLAIR	Artificial neural networks	BFC:N4; IN:histogram matching	n/a	n/a	n	n	BT	LGG; HGG	65; 327	1 (BraTS'13); 1 (BraTS'15)	y	-		DSC; PPV; Sen
J	2016	N. Cordier et al.	A Patch-Based Approach for the Segmentation of Pathologies: Application to Glioma Labelling	IEEE Transactions on Medical Imaging		y	T1, T1c, T2, FLAIR	Other	REG:affine; resampling; IN:1-99% clipping + u & v scaling; tissue maps:FSL FAST	2.66GHz 6GB RAM	up to 16 min	n	n	BT	LGG; HGG	304?	1 (BraTS'13); 1 (BraTS'14)	y	-		DSC; Hdff
J	2016	M. J. Fartaria et al.	Automated detection of white matter and cortical lesions in early stages of multiple sclerosis	Journal of Magnetic Resonance Imaging		y	T1, FLAIR, MP2RAGE, DIR	K-Nearest Neighbours, Region Growing	REG:rigid; BE:MorphoBox; BFC:N4; IN:histogram mtaching; tissue maps	n/a	n/a	n	n	MS	MS	39	3	n	Manual, 2	consensus	DSC; Sen; Spe; Acc; Bland-Altman; other
J	2016	J. C. Griffis et al.	Voxel-based Gaussian naïve Bayes classification of ischemic stroke lesions in individual T1-weighted MRI scans	Journal of Neuroscience Methods		n	T1	Bayesian classification	tissue map:SPM12; BFC:SPM12; REG:SPM12	n/a	n/a	n	y	S	S	30	3 (Cincinnati Children's Hospital Medical Center)	y	Semiautomatic		DSC; VD; VC
J	2016	Y. Li et al.	Brain tumor segmentation from multimodal magnetic resonance images via sparse representation	Artificial Intelligence in Medicine		y	T1, T1c, T2, FLAIR	Sparse representation, Markov Random Field, Graph Cut,	BFC:N3; IN:histogram matching + 0-1 range	i7 1.8GHz 64 GB RAM	2-5 min	n	n	BT	LGG; HGG	64	1 (BraTS'13)	y	-		DSC; PPV; Sen; Kappa
J	2016	J. R. Stone et al.	Supervised learning technique for the automated identification of white matter hyperintensities in traumatic brain injury	Brain Injury		y	T1, T2, FLAIR	Random Forest, Markov Random Field	convert to nii; REG:rigid; denoising:ref; BFC:N4; IN:0-1 range; BE:ANTs	n/a	n/a		y	WML	TBI	24	3 (Chronic Effects of Neurotrauma Consortium, Walker et al. 2016)	n	Manual, 1		Sen; PPV; F1; VD
J	2015	V. G. Kanas et al.	A low cost approach for brain tumor segmentation based on intensity modeling and 3D Random Walker	Biomedical Signal Processing and Control	Segmentation failure	y	T1, T1c, T2, FLAIR	Random Walker	denoising; BFC; REG:FSL; BE:BET	3GHz 8GB RAM	<1 min	n	n	BT	LGG; HGG	34; 23	1 (BraTS'13); 3 (Prastawa et al. 2009); 3	y	Manual, 1		FPR; TRP; DSC; Hdff
J	2015	O. Commowick et al.	Diffusion MRI abnormalities detection with orientation distribution functions: A multiple sclerosis longitudinal study	Medical Image Analysis		n	DTI	Other	Movement correction; distortion correction; tensor and ODF estimation	n/a	n/a	n	n	MS	CIS	15x2	3	n	Manual, 1		other
J	2015	D. Guo et al.	Automated lesion detection on MRI scans using combined unsupervised and supervised methods	BMC Medical Imaging		n	T1	Support Vector Machine, Other	enantiomorphic normalization	I7 2.2GHz 16GB RAM	388 min	n	n	S; BT	S; G	60; 30	1 (BraTS'12); 3	y	Manual, 3	consensus	Acc; PPV; Sen; DSC
J	2015	P. K. Roy et al.	Automatic white matter lesion segmentation using contrast enhanced FLAIR intensity and Markov Random Field	Computerized Medical Imaging and Graphics		y	T1, FLAIR	Random Forest, Markov Random Field	REG:rigid(SPM8); BE:BET; BFC:N3	n/a	n/a	n	n	WML	HT	24; 20	1 (MS GC MICCAI'08); 3 (ENVISion, Reid et al. 2012)	y	Manual, 2		FROC; DSC; TPR; PPV
J	2015	E. Roura et al.	A toolbox for multiple sclerosis lesion segmentation.	Neuroradiology		y	T1, FLAIR	Thresholding	BE:BET; denoising:ADF;BFC:N3;REG:affine(SPM8/12)	n/a	n/a	n	y	MS	CIS	70; 20 (+23 test online); 14	1 (MS GC MICCAI'08); 3 (Hospital Vall d'Hebron, Spain); 3 (Clínica Girona, Spain)	y	Manual, multiple		DSC; TPR; PPV

For peer review only

J	2015	N. Guizard et al.	Rotation-invariant multi-contrast non-local means for MS lesion segmentation	NeuroImage: Clinical		y	T2, FLAIR	Non-local Means	denoising:NLM; BFC:N3; IN: histogram matching; REG:rigid; BE:ref	i7 3.06 GHz	40 min	n	planned	MS	-	108; 20 (+23 test online)	1 (MS GC MICCAI'08); 3	y	Semiautomatic		DSC; Sen; PPV; VDf; FPR; SSD; TPR; PPV; VR
J	2015	S. Jain et al.	Automatic segmentation and volumetry of multiple sclerosis brain lesions from MR images	NeuroImage: Clinical	Scan-rescan reproducibility	y	T1, FLAIR	Other	REG:rigid; BE:MNI brain mask reg(affine+non-rigid); tissue maps	n/a	n/a	n	n	MS	-	20; 10x2	3 (VU University Medical Center, Amsterdam, the Netherlands); 3 (UZ Brussels, Brussels, Belgium)	y	Manual, 3	consensus	DSC; ICC; absVD; Sen; PPV
J	2015	R. Harmouche et al.	Probabilistic Multiple Sclerosis Lesion Classification Based on Modeling Regional Intensity Variability and Local Neighborhood Information	IEEE Transactions on Biomedical Engineering		y	T1, T2, PD, FLAIR	Other	BFC:N3; REG:ref; BE:BET; IN:histogram matching	Optiplex 980 quad-core	2 min	n	n	MS	-	100	3	y	Manual, 1&5	majority	DSC; Sen; PPV
J	2015	Z. Karimaghaloo et al.	Temporal Hierarchical Adaptive Texture CRF for Automatic Detection of Gadolinium-Enhancing Multiple Sclerosis Lesions in Brain MRI	IEEE Transactions on Medical Imaging		y	T1, T1c, T2, PD, FLAIR	Conditional Random Field	REG:ref; BE:BET; BFC:N3; IN:histogram matching	2.66GHz	56 sec	treatment; placebo	n	MS	-	1190x2; 120x2; 69x4	3	y	Manual, 2	consensus	Sen; FPR
J	2015	T. Zhan et al.	A novel brain tumor segmentation method for multi-modality human brain MRIs	International Journal of Multimedia and Ubiquitous Engineering		y	T1, T1c, T2, FLAIR	Bayesian classification, Logistic Regression	REG:rigid; BFC:N3; BE	n/a	n/a	n	n	BT	-	10 +10 simulated	1 (BraTS'12)	y	-		Jcc
J	2015	M. P. Arakeri and G. R. M. Reddy	Computer-aided diagnosis system for tissue characterization of brain tumor on magnetic resonance images	SIViP	Inter-rater agreement evaluated	y	T1c, T2	Fuzzy C-Means	denoising:median filtering; contrast enhnacement:histogram equalization; REG:FLIRT; BE:Otsu's thresholding	3GHz 3GB RAM	1 sec	benign, malignant	n	BT	-	550	3 (Shirdi Sai Cancer Hospital, Manipal, India)	n	Manual, 2		Hdff; VO; avgSD
J	2015	A. Demirhan et al.	Segmentation of Tumor and Edema Along With Healthy Tissues of Brain Using Wavelets and Neural Networks	EEE journal of biomedical and health informatics		y	T1, T2, FLAIR	Self Organising Maps	BE: thresholding + morphological operations; IN:0-1 range; denoising:ADF; REG:affine	n/a	n/a	n	n	BT	G	20	1 (BraTS'12); 3 (Mayıs Hospital in Ankara, Turkey)	y	Manual		DSC; Sen; Spe
J	2015	C. H. Sudre et al.	Bayesian Model Selection for Pathological Neuroimaging Data Applied to White Matter Lesion Segmentation	IEEE Transactions on Medical Imaging		y	T1, FLAIR; T2, PD, FLAIR	Expectation-Maximization Gausian mixture model, Markov Random Field	BFC:in house; REG; IN:0-1 range; BE:ref	n/a	n/a	n	n	WML	MS; DM	20	1 (MS GC MICCAI'08)	y	-		DSC; TPR; FNR; VD; AD; DE; OER
J	2015	B. Ahmed et al.	Cortical feature analysis and machine learning improves detection of 'MRI-negative' focal cortical dysplasia	Epilepsy & Behavior		n	T1	Linear regression, Bagging	BFC; BE:ref; FreeSurfer	n/a	n/a	n	n	FCD	FCD	31 (24 MRI negative) + 62 controls	3 (New York University School of Medicine Comprehensive Epilepsy Center)	n	Manual, 2	supervision	TPR; FPR; DSC; avgSD
J	2015	S. D. S. Al-Shaikhli et al.	Brain tumor classification and segmentation using sparse coding and dictionary learning	Biomedical Engineering / Biomedizinische Technik		n	FLAIR	Graph Cut, Dictionary Learning	BE:BET; BFC:MIPAV	n/a	n/a	normal, abnormal	n	BT	GBM; G; MET	30; 30; 30; 30	1 (BraTS'13); 1 (IBSR); 1 (SPL)	y	-		DSC; PPV; Sen; Jcc; Kappa
J	2015	P. Szwarc et al.	Automatic brain tumour detection and neovasculature assessment with multiseries MRI analysis	Computerized Medical Imaging and Graphics	Inter-rater agreement evaluated	y	T1, T1c, FLAIR, RCBV	Fuzzy C-Means	REG; interpolation: nearest neighbour; BE:Otsu's thresholding; symmetry plane detection	n/a	n/a	n	n	BT	-	10; 10	3	y	Manual, 1&2		DSC; Sen; Spe
J	2015	J. Juan-Albarracín et al.	Automated Glioblastoma Segmentation Based on a Multiparametric Structured Unsupervised Classification	PloS one		y	T1, T1c, T2, FLAIR	Markov Random Field	denoising:ref; BE:Brain Suite Software; BFC:N4; resampling:ref	Intel Xeon E5-2620 64GB RAM	~39 min	n	n	BT	-	31	1 (BraTS'13)	y	-		DSC; PPV; Sen; Kappa
J	2015	B. Ozenne et al.	Spatially regularized mixture model for lesion segmentation with application to stroke patients	Biostatistics		y	T1, FLAIR	Markov Mixture Models	REG; tissue maps	n/a	15 min	n	upon request	S	S	12	3 (I-KNOW)	n	Manual, 3	majority	other
J	2015	X. Tomas-Fernandez and S. K. Warfield	A Model of Population and Subject (MOPS) Intensities With Application to Multiple Sclerosis Lesion Segmentation	IEEE Transactions on Medical Imaging		y	T1, T2, FLAIR	Expectation-Maximization Gausian mixture model, Graph Cut	BFC:ref; denoising:ref	n/a	n/a	n	n	MS	MS	3 (synthetic); 51	1 (MS GC MICCAI'08)	y	-		TPR; FPR; Extra Fraction; Miss Fraction; PPV
J	2015	I. Njeh et al.	3D multimodal MRI brain glioma tumor and edema segmentation: A graph cut distribution matching approach	Computerized Medical Imaging and Graphics		y	T1, T1c, T2, FLAIR	Other, Graph Cut	BRATS only	n/a	<0.5 sec	n	n	BT	LGG; HGG	45 +65 simulated	1 (BraTS'12)	y	-		DSC
J	2015	O. Maier et al.	Extra Tree forests for sub-acute ischemic stroke lesion segmentation in MR sequences	Journal of Neuroscience Methods	Errors in preprocessing, Segmentation failure	y – adaptable to single and multi	T1, T2, FLAIR, DWI	Random Forest	resampling; REG:rigid(elastix); BE:BET; BFC:Bies Field Correction Tool; IN:histogram matching	n/a	n/a	n	n	S	S	37	3 (Machner et al. 2012)	n	Manual, 1		DSC; avgSSD; Hdff; PPV; Sen
C	2015	O. Maier et al.	MS lesion segmentation in MRI with random forests	2015 Longitudinal Multiple Sclerosis Lesion Segmentation Challenge	Inter-rater agreement evaluated	n/a	n/a	Random Forest	IN:MedPy	n/a	n/a	n	n	MS	-	5x4	1 (MSC'15)	n	-		DSC; avgSSD; TPR. FPR
J	2015	T. C. Steed et al.	Iterative Probabilistic Voxel Labeling: Automated Segmentation for Analysis of The Cancer Imaging Archive Glioblastoma Images	American Journal of Neuroradiology	Inter-rater agreement evaluated	y	T1, T1c, T2, FLAIR	K-Nearest Neighbours, Thresholding	BFC:FAST; REG:nonlinear + affine; BE:BET+ROBEX; tissue maps	n/a	11 min	n	n	BT	-	40	2 (TCIA, Clark et al. 2013)	y	Manual, 3	supervision	DSC
J	2015	R. Wang et al.	Automatic segmentation and volumetric quantification of white matter hyperintensities on fluid-attenuated inversion recovery images using the extreme value distribution	Neuroradiology		n	FLAIR	Other	BFC:N3; IN:0-255 range; BE:BET	n/a	n/a	n	planned	WML	MS	60, 10	1 (MS GC MICCAI'08); 3	y	Manual, 2	consensus	DSC; FPR; FNR; OER; DER; Bland-altman; ICC; VC; SI
J	2015	P. Maji and S. Roy	SoBT-RFW: Rough-Fuzzy Computing and Wavelet Analysis Based Automatic Brain Tumor Detection Method from MR Images	Fundamenta Informaticae		n/a	n/a	Fuzzy C-Means, Region Growing	BE:S3	3.4GHz 16GB RAM	n/a	n	y	BT	-	25	1 (BraTS'12)	y	-		DSC; Sen; Spe
J	2015	N. Nabizadeh and M. Kubat	Brain tumors detection and segmentation in MR images: Gabor wavelet vs. statistical features	Computers & Electrical Engineering	Detection followed by segmentation	n	T1/FLAIR	Comparison	IN; histogram normalization	3GHz 64GB RAM	~5 min	n	n	BT	HGG	25 (unclear how many real and how many simulated)	1 (BraTS'13)	y	-		Sen; Spe; Acc

For peer review only

J	2014	N. Gonçalves et al.	Self-supervised MRI tissue segmentation by discriminative clustering	Int. J. Neur. Syst		y	T1, T2, PD; T1 T2, FLAIR	Self Organising Maps	REG:SPM5; BE:BET2	n/a	n/a	n	n	MS	MS	2; 54	1 (MS GC MICCAI'08)	y	-		DSC; other
J	2014	Y. Zhong et al.	Automated White Matter Hyperintensity Detection in Multiple Sclerosis Using 3D T2 FLAIR	Journal of Biomedical Imaging		n	FLAIR	Other	BE:in-house; BFC:modified homodyne HP filter; tissue maps:thresholding	I7 2.8GHz 8GB RAM	5 min	n	n	MS	-	26	3 (Synergy Health Concepts, CA)	n	Manual		SI; VC
J	2014	Y.-H. Mah et al.	A new method for automated high-dimensional lesion segmentation evaluated in vascular injury and applied to the human occipital lobe	Cortex		n	DWI	Other	REG:SPM5	n/a	n/a	n	y	S	-	38 + 95 controls	3	n	Manual, 1		DSC; Sen; Spe
J	2014	B. I. Yoo et al.	Application of variable threshold intensity to segmentation for white matter hyperintensities in fluid attenuated inversion recovery magnetic resonance images	Neuroradiology		n	FLAIR	Thresholding	BFC:SPM8; BE:SPM8	n/a	n/a	n	n	WML	-	48	3	y	Manual, 2		DSC; Sen; Spe; Jcc; ICC
J	2014	M. Cabezas et al.	Automatic multiple sclerosis lesion detection in brain MRI by FLAIR thresholding	Computer Methods and Programs in Biomedicine		n	FLAIR	Expectation-Maximization, Thresholding	BE:BET; denoising:ADF; BFC:N4; REG:affine	QuadCore 2.3 GHz, 16 Gb RAM	several min	n	n	MS	-	45	3 (Hospital Vall d'Hebron); 3 (Hospital Josep Trueta); 3 (Clínica Girona)	y	Semiautomatic		TPR; FPR; DSC; avgSD
J	2014	J.-Z. Tsai et al.	Automated Segmentation and Quantification of White Matter Hyperintensities in Acute Ischemic Stroke Patients with Cerebral Infarction	PloS one		y	T1, DWI, FLAIR	Thresholding, Other	REG:rigid(MNI); tisue maps:SPM8; IN:1-100 range; BE:BET	i5 2.67GHz 4GB RAM	~15 min	n	n	WML	S	30	3 (Taiwan Stroke Registry, Hsieh, et al. 2010)	n	Semiautomatic		SI; ICC; Bland-Altman; Sen; Spe
J	2014	P. Dvořák et al.	Unsupervised Pathological Area Extraction using 3D T2 and FLAIR MR Images	Measurement Science Review	No preprocessing	y	T2, FLAIR	Other	Mid-sagittal plane extraction	n/a	<1 min	n	n	BT	LGG; HGG	30 +50 simulated	1 (BraTS'12)	y	-		DSC; Acc
J	2014	Y. Lu et al.	Multimodal Brain-Tumor Segmentation Based on Dirichlet Process Mixture Model with Anisotropic Diffusion and Markov Random Field Prior	Computational and Mathematical Methods in Medicine		y	T1c, FLAIR	Markov Random Field, Other	denoising: ADF	n/a	n/a	n	n	BT	LGG; HGG	55 + 65 synthetic	1 (BraTS'12)	y	-		DSC; Jcc; Sen; Spe
J	2014	R. Wang et al.	Automatic segmentation of white matter lesions on magnetic resonance images of the brain by using an outlier detection strategy	Magnetic Resonance Imaging		y – adaptable to single and multi	T1, T2, FLAIR	Expectation-Maximization Gausian mixture model, Other	BFC:N3; BE:BET; REG:demons registration	n/a	2.2 sec per slice	n	n	WML	-	86 + synthetic	3	n	Manual, 2		DSC; Overlap Fraction; Extra Fraction; VC; Band-Altman
J	2014	M. Huang et al.	Brain Tumor Segmentation Based on Local Independent Projection-Based Classification	IEEE Transactions on Biomedical Engineering		y	T1, T1c, T2, FLAIR	Other	BFC:N3; IN:1-99% clipping + 0-100 range	I5 3.1GHz	26 min	n	n	BT	LGG; HGG	55 +65 synthetic	1 (BraTS'12); 1 (BraTS'13)	y	-		DSC; Jcc; FPR; FNR
J	2014	J. Mitra et al.	Lesion segmentation from multimodal MRI using random forest following ischemic stroke	NeuroImage		y	T1, T2, FLAIR, DWI (ADC)	Markov Random Field, Random Forest	REG:rigid; tissue masks:ref; BE: masking	3.2GHz 23.5GB RAM	27 min	n	n	S, WML	S	36	1 (MS GC MICCAI'08); 3	y	Manual, 1		DSC; PPV; TPR; avgSSD; VD
J	2014	J.-Z. Tsai et al.	Automatic Detection and Quantification of Acute Cerebral Infarct by Fuzzy Clustering and Histographic Characterization on Diffusion Weighted MR Imaging and Apparent Diffusion Coefficient Map	BioMed Research International		n	DWI (ADC)	Fuzzy C-Means, Thresholding, edge detection,	reg:rigid + trilinear interpolation NMI; IN:0-1 range; BE:BET; histogram smoothing:3rd order moving avg filter	i5 2.67GHz 4GB RAM	5 min	n	n	S	S	22	3 (Taiwan Stroke Registry, Hsieh, et al. 2010)	n	Semiautomatic		DSC; Kappa; ICC; Sen; Spe; PPV
J	2014	J. Zhang et al.	A fully automatic extraction of magnetic resonance image features in glioblastoma patients	Med Phys		y	T1, T1c, FLAIR	Random Forest	REG:rigid (FLIRT); denoising:median filtering	n/a	n/a	n	n	BT	GBM	73	2 (TCIA, Clark et al. 2013)	n	Manual, 1		DSC
J	2014	O. Ganiler et al.	A subtraction pipeline for automatic detection of new appearing multiple sclerosis lesions in longitudinal studies	Neuroradiology		y	T1, T2, PD	Thresholding, Other	BE:BET; REG:affine + rigid; BFC:N4; IN:histogram matching; tissue map	n/a	n/a	n	n	MS	MS	20x2	3	n	Manual, 2	supervision	Sen; FPR; DSC
J	2014	G. Erus et al.	Individualized statistical learning from medical image databases: Application to identification of brain lesions	Medical Image Analysis	Inter-rater agreement evaluated	n/a	n/a	Other	BE:BET; BFC: N3; IN:histogram matching; REG:non-lin(HAMMER)	n/a	n/a	n	n	WML, S	DM	80	3	n/a	Manual, 1&2		DSC; Sen; Spe
J	2014	E. M. Sweeney et al.	A Comparison of Supervised Machine Learning Algorithms and Feature Vectors for MS Lesion Segmentation Using Multimodal Structural MRI	PloS one	Raters' years of experience given	y	T1, T2, FLAIR	Comparison	REG:rigd; BFC:N3; BE:SPECTRE; CSF removal:15th percentile	1 core	<1 min	n	n	MS	MS	98	3	y	Manual, 2	supervision	ROC; DSC
J	2013	G. Liberman et al.	Automatic multi-modal MR tissue classification for the assessment of response to bevacizumab in patients with glioblastoma	European Journal of Radiology	Treatment response evaluation	y	T1, T1c, T2, T2*, T2*c, FLAIR	Expectation-Maximization Gausian mixture model, K-Nearest Neighbours	BFC:N3; REG:SPM; IN:multiplicative model; BE:BET	n/a	n/a	n	n	BT	GB	59	3	n	Manual		Sen; Spe; VC; other
J	2013	E. M. Sweeney et al.	Automatic Lesion Incidence Estimation and Detection in Multiple Sclerosis Using Multisequence Longitudinal MRI	American Journal of Neuroradiology		y	T1, T2, PD, FLAIR	Other, Logistic Regression	resampling; REG: rigid; BE:ref; tissue maps	n/a	n/a	n	n	MS	-	10x11	3	n	Manual, 1		ROC; Volume Change
J	2013	S. Ji et al.	Automatic segmentation of white matter hyperintensities by an extended FitzHugh & nagumo reaction diffusion model	Journal of Magnetic Resonance Imaging		n	FLAIR	Other	BE:BET; denoising:ADF	n/a	n/a	n	n	WML	-	127	3	y	Manual, 3	consensus	DSC
J	2013	C. Elliott et al.	Temporally Consistent Probabilistic Detection of New Multiple Sclerosis Lesions in Brain MRI	IEEE Transactions on Medical Imaging		y	T1, T2, PD, FLAIR	Bayesian classification, Markov Random Field	BE:BET; BFC:N3; REG:rigid; IN:histogram matchingl + least trimmed squares	n/a	n/a	n	n	MS	-	684 (155 participants)	3	y	Semiautomatic		Sen; FD
J	2013	E. M. Sweeney et al.	OASIS is Automated Statistical Inference for Segmentation, with applications to multiple sclerosis lesion segmentation in MRI	NeuroImage: Clinical	Inter-rater agreement evaluated	y	T1, T2, PD, FLAIR	Logistic Regression	REG:rigid; BFC:N3; BE:SPECTRE; IN:0u1v; smoothing	n/a	n/a	n	n	MS	-	98 + 33 healthy; 20x2, 128 + 1 healthy	3	y	Manual, 1		ROC; Sen; DSC
J	2013	K. Mouridsen et al.	Acute Stroke: Automatic Perfusion Lesion Outlining Using Level Sets	Radiology	Inter-rater agreement evaluated	y	PWI, DWI	Level Set	REG:linear	n/a	n/a	n	n	S	S	14	3	n	Manual, 4	consensus	Bland – Altman; DSC
J	2013	A. Resmi et al.	A novel automatic method for extraction of glioma tumour, white matter and grey matter from brain magnetic resonance images	Biomedical Imaging and Intervention Journal	Inter-rater agreement evaluated	y	T1, FLAIR, T2	Thresholding, Other	IN:0-1 range; BE:morphological operations	n/a	1 sec	n	n	BT	LGG, HGG	20 +50 synthetic	3 (Sree Chitra Institute of Medical Sciences and Technology); 3 (Regional Cancer Centre, Thiruvananthapuram, Kerala, India)	y	Manual, 1		PPV; Jcc; other
J	2013	A. Bijar et al.	Increasing the Contrast of the Brain MR FLAIR Images Using Fuzzy Membership Functions and Structural Similarity Indices in Order to Segment MS Lesions	PloS one		n	FLAIR	Thresholding, Genetic Algorithm	n/a	2.5 GHz 512 MB RAM	<30 sec	n	n	MS	MS	20	3 (Khayati et al. 2008)	n	Manual, 2	consensus	DSC; Sen; Extra Fraction; ICC
J	2013	R. Simões et al.	Automatic segmentation of cerebral white matter hyperintensities using only 3D FLAIR images	Magnetic Resonance Imaging		n	FLAIR	Expectation-Maximization Gausian mixture model	BE:BET; BFC:FAST	n/a	n/a	n	n	WML	MCI; progressive MCI	25 + 15 healthy; 23	1 (MS GC MICCAI'08); 3 (University Hospital of Essen, Germany)	y	Manual, 1		DSC; Overlap Fraction; Extra Fraction; VC
J	2013	L. Shi et al.	Automated quantification of white matter lesion in magnetic resonance imaging of patients with acute infarction	Journal of Neuroscience Methods		y	T1, FLAIR, DWI	Other	REG:affine; IN:in house; BE:BET; tissue maps:REF	2.8GHz 12GB RAM	25 sec	n	n	WML	S	91(30 segmented)	3	y	Semiautomatic		DSC; Sen; PPV; ICC; Bland-Altman
J	2012	L. Weizman et al.	Automatic segmentation, internal classification, and follow-up of optic pathway gliomas in MRI	Medical Image Analysis	Indirect reproducibility evaluation	y	T1, T2, FLAIR	Other	REG:SPM; IN:dynamic histogram warping	n/a	n/a	n	n	BT	optic pathway G	28	3	n	Manual, 2		VD; avgSSD; DSC; ROC
J	2012	Y. Wang et al.	Multi-stage segmentation of white matter hyperintensity, cortical and lacunar infarcts	NeuroImage		y	T1, T2, FLAIR	Expectation-Maximization Gausian mixture model, Region Growing	BFC:REF; BE:BET; REG: FLIRT; tissue map:FreeSurfer	n/a	n/a	WMH; CI; LI	n	WML	-	272	3 (Memory Aging & Cognition Center at the National University of Singapore)	n	Manual, 1-3	supervision	DSC; VC; Bland-Altman

For peer review only

J	2012	K. H. Ong et al.	Automatic white matter lesion segmentation using an adaptive outlier detection method	Magnetic Resonance Imaging		y	T1, FLAIR; T1, T2, FLAIR	Thresholding	BE:model-based level set; BFC:N3	n/a	n/a	n	n	WML	-	38; 23	1 (MS GC MICCAI'08); 3 (Universiti Sains Malaysia's hospital)	y	Manual, 1		VR; Jcc; DSC; Sen; FPR; VD
J	2012	T. Samaille et al.	Contrast-Based Fully Automatic Segmentation of White Matter Hyperintensities: Method and Validation	PloS one		y	T1, FLAIR	Thresholding	BE:SPM8; REG:rigid; BFC:SPM8	n/a	30 min	n	n	WML	MCI; CADASIL	24; 43	3 (Salpêtrière Hospital); 3 (Lariboisière Hospital)	y	Semiautomatic		ICC; Bland-Altman; VR; DSC
J	2012	B. A. Abdullah et al.	Multi-Sectional Views Textural Based SVM for MS Lesion Segmentation in Multi-Channels MRIs	Open Biomed Eng J		y	T1, T2, FLAIR	Support Vector Machine	Resampling; REG:AIR; IN:ref	n/a	n/a	n	n	MS	-	51 (+synthetic); 10	1 (MS GC MICCAI'08); 3 (University of Miami)	y	n/a		DSC; TPR; PPV; other
J	2012	P. Schmidt et al.	An automated tool for detection of FLAIR-hyperintense white-matter lesions in Multiple Sclerosis	NeuroImage		y	T1, FLAIR	Region Growing, Partial Volume Estimation	BFC:VBM8; tissue maps; REG:linear + non-linear	3.2 GHz 8GB RAM	13 min	n	planned	MS	-	52 + 10 (unclear)+ 18 control	3	n	Manual, 2	consensus	VC; VR; Sen; Spe; Acc; DSC
J	2012	A. Gooya et al.	GLISTR: Glioma Image Segmentation and Registration	IEEE Transactions on Medical Imaging		y	T1, T1c, T2, FLAIR	Expectation-Maximization, tumor growth, atlas registration,	BFC; BE; cerebelum removal; REG:affine	n/a	3-6 hs	n	n	BT	-	122 (10 segmented)	3 (University of Pennsylvania)	n	Manual, 1		DSC; SD
C	2014	D. Kwon et al.	Multimodal brain tumor image segmentation using glistr	MICCAI BRATS		y	T1, T1c, T2, FLAIR	Expectation-Maximization, tumor growth, atlas registration,	BFC; IN:0-255 range	I7 3.4 GHz	85 min	n	n	BT	LGG, HGG	35	1 (BraTS'13)	y	-		DSC; PPV; Sen
J	2012	K. Popuri et al.	3D variational brain tumor segmentation using Dirichlet priors on a clustered feature set	Int J CARS		y	T1, T1c, T2	Active Contour, Other	denoising:edge-preserving non-linear filter; IN:weighted least squares; BFC:N3; symmetrical alignment:rigid	n/a	n/a	n	n	BT	G	15	3	n	Manual, 1		Jcc; PPV; Sen; Hdff
J	2012	N. M. Saad et al.	Fully Automated Region Growing Segmentation of Brain Lesion in Diffusion-weighted MRI	IAENG International Journal of Computer Science		n	DWI	Thresholding, Region Growing, Other,	IN:0-1 range + gamma-law transformation; BE:thresholding + morphological operations	n/a	n/a	n	n	S, BT, abscess	-	23 (slices used)	3 (General Hospital of Kuala Lumpur)	n	Manual		Jcc; FPR; FNR; other
J	2012	M. de C. Alegro et al.	Computerized brain tumor segmentation in magnetic resonance imaging	Einstein (São Paulo)	Semiautomatic preprocessing	y	T1, T1c, FLAIR	Support Vector Machine	BFC:N3; denoising:ADF; REG:semiautomatic; BE:ref; IN:histogram matching	n/a	n/a	n	n	BT	T	11	3 (Universidade de São Paulo)	n	Manual 2		other
J	2012	A. Khademi et al.	Robust White Matter Lesion Segmentation in FLAIR MRI	IEEE Transactions on Biomedical Engineering		n	FLAIR	Thresholding, Partial Volume Estimation	BE:ref; bilateral filtering	n/a	n/a	n	n	WML	-	24	3	n	Manual, 1		DSC; Sen; Spe
C	2015	A. Khademi et al.	Multiscale Partial Volume Estimation for Segmentation of White Matter Lesions Using Flair MRI	2015 IEEE 12th International Symposium on Biomedical Imaging (ISBI)		n	FLAIR	edge content, Partial Volume Estimation	n/a	n/a	n/a	n	n	WML	-	30	3 (Khademi et al. 2012)	n/a	Manual, 1		DSC; Extra Fraction; Bland-Altman
J	2012	Zacharaki, E. and Bezerianos A.	Abnormality Segmentation in Brain Images Via Distributed Estimation	IEEE Transactions on Information Technology in Biomedicine		n/a		Other	BE:ref; BFC:ref; IN:histogram matching, 0-1 range + 0u1v; REG:deformable	Intel Core2 Duo CPU 2GHz	Min – hrs	n	n	WML	DM	33 +73 healthy	3 (ACCORD-MIND)	n/a	Manual, 1		ROC
J	2011	D. Garcia-Lorenzo et al.	Trimmed-Likelihood Estimation for Focal Lesions and Tissue Segmentation in Multisequence MRI for Multiple Sclerosis	IEEE Transactions on Medical Imaging	Inter-rater agreement evaluated	y	T1, T2, PD	Other	BFC:ref; reg:rigid; BE:BET	2.4GHz 4GB RAM	2 min	n	n	MS	MS	10; simulated	1 (BrainWeb); 3	n	Manual, 5	majority	DSC; ICC
C	2008	D. Garcia-Lorenzo et al.	A Robust Expectation-Maximization Algorithm for Multiple Sclerosis Lesion Segmentation	MICCAI Workshop: 3D Segmentation in the Clinic: A Grand Challenge II, MS Lesion Segmentation		y	T1, T2, FLAIR	Expectation-Maximization, outlier detection	BFC:ref; BE:BET	n/a	n/a	n	n	MS	MS	44	1 (MS GC MICCAI'08)	y	-		VD; AD; TP; FP
J	2011	E. Geremia et al.	Spatial decision forests for MS lesion segmentation in multi-channel magnetic resonance images	NeuroImage		y	T1, T2, FLAIR	Decision Tree	resampling; cropping; BFC:REF; IN:REF; REF:ref; tissue maps:INRIA	IBM e325 dual-Opterons246 2 GHz	n/a	n	n	MS	MS	45	1 (MS GC MICCAI'08)	y	-		TPR; PPV; VD; SD
C	2012	E. Geremia et al.	Spatial decision forests for glioma segmentation in multi-channel MR images	MICCAI Challenge on Multimodal Brain Tumor Segmentation		y	T1, T1c, T2, FLAIR	Decision Tree	IN:histogram matching	n/a	n/a	n	n	BT	LGG, HGG	30 (+25 synthetic)	1 (BrainWeb); 3	y	n/a		DSC; TPR; PPV
J	2011	N. Zhang et al.	Kernel feature selection to fuse multi-spectral MRI images for brain tumor segmentation	Computer Vision and Image Understanding		y	T2, PD, FLAIR	Support Vector Machine, Region Growing	REG:SPM	n/a	n/a	n	n	BT	-	8+3x5(?)	3	y	Manual, multiple		TP;FP; FN; other
J	2011	S. Jeon et al.	Fully automated pipeline for quantification and localization of white matter hyperintensity in brain magnetic resonance image	International Journal of Imaging Systems and Technology		y	T1, FLAIR	Other	BFC:N3; BE:BET; REG:affine; tissue masks:INSECT	n/a	n/a	n	n	WML	SVD	10 annotated (+35 not)	3 (Lee et al. 2011)	n	Manual, 1		DSC; Jcc; Bland-Altman; VR
J	2011	V. Harati et al.	Fully automated tumor segmentation based on improved fuzzy connectedness algorithm in brain MR images	Computers in Biology and Medicine		n	T1c	Other	denoising:ADF; head mask:thresholding; IN:0-255 range	3.2GHz, 1GB RAM	2.5 min	n	n	BT	T	10	3	n/a	Manual, 1		DSC; Spe; Extra fraction
J	2010	D. Selvathi et al.	Hybrid approach for brain tumor segmentation in magnetic resonance images using cellular neural networks and optimization techniques	International Journal of Computational Intelligence and Applications		y	T2, FLAIR	Artificial neural networks, Genetic Algorithm	IN:-1-1 range	3GHz 512MB RAM	n/a	n	n	BT	astrocytoma; MET; G; meningioma	30	3	n	Manual		Acc
J	2010	M. Scully et al.	An automated method for segmenting white matter lesions through multi-level morphometric feature classification with application to lupus	Frontiers in Human Neuroscience		y	T1, T2, FLAIR	Support Vector Machine	resampling:trilinear interpolation; REG:MI; BFC:ref; IN:histogram matching	n/a	n/a	n	n	WML	lupus	27	3 (The Mind Research Network, University of New Mexico Hospital)	n	Manual, multiple		Sen; Spe
J	2010	N. Shiee et al.	A topology-preserving approach to the segmentation of brain images with multiple sclerosis lesions	NeuroImage		y- adaptable	T1, FLAIR	Fuzzy Clustering, Other	REG:rigid	modern Linux workstation	~45 min	n	y	MS	MS	10	1 (BrainWeb); 3	n	Semiautomatic		DSC; TP; VC
J	2009	K. E. Emblem et al.	Automatic glioma characterization from dynamic susceptibility contrast imaging: Brain tumor segmentation using knowledge-based fuzzy clustering	Journal of Magnetic Resonance Imaging	Inter-rater agreement evaluated	y	T1c, T2, FLAIR	Fuzzy C-Means, Thresholding	REG:NMI (SPM5); IN:adaptive histogram equalization; BE:SPM5	Pentium 4 3.19GHz 3GB RAM	4 min	Survival: ow-risk; high-risk	n	BT	G	50	3	n	Manual, 4	majority	Sen; PPV; VC; ROC
J	2009	R. Beare et al.	Development and validation of morphological segmentation of age-related cerebral white matter hyperintensities	NeuroImage		y	T1, T2, FLAIR	boosting, Watershed	REG:SPM5; BFC:ref; IN:median and interquartile range scaling; tissue map	n/a	n/a	n	n	WML	-	247	3 (Tasmanian Study of Cognition and Gait)	n	Manual, 1; Semiautomatic		ICC; SI
J	2009	R. de Boer et al.	White matter lesion extension to automatic brain tissue segmentation on MRI	NeuroImage	Inter-rater agreement evaluated	y	T1, PD, FLAIR	K-Nearest Neighbours, Thresholding	REG:rigid; resampling; BE:ELASTIX; BFC:N3; IN:4-96% clipping + 0-1 range	n/a	n/a	n	n	WML	-	195 + 20	3 (Rotterdam Scan Study, de Leeuw et al. 2001)	n	Manual, 1&2		DSC; Sen; Extra Fraction; VD; other
J	2009	J. Nie et al.	Automated brain tumor segmentation using spatial accuracy-weighted hidden Markov Random Field	Computerized Medical Imaging and Graphics	Visual validation of preprocessing	y	T1, T2, FLAIR	Expectation-Maximization-Markov Random Field	REG; BE:BET	3GHz 2GB RAM	20-25 min	n	n	BT	G	15	3	n	Semiautomatic		Jcc; VO
J	2009	A. Akselrod-Ballin et al.	Automatic Segmentation and Classification of Multiple Sclerosis in Multichannel MRI	IEEE Transactions on Biomedical Engineering	Indirect reproducibility evaluation	y – adaptable to single and multi	T1, T2, PD and/or FLAIR	Decision Tree, Other	REG:during acquisition	Xeon PC 1.7GHz	<10 min	n	n	MS	MS	25 + 4x4	3 (cientific Institute Ospedale San Raffaele)	n	Manual, 2	consensus	DSC; Sen; Spe; Acc; VC
J	2008	R. Khayati et al.	Fully automatic segmentation of multiple sclerosis lesions in brain MR FLAIR images using adaptive mixtures method and markov random field model	Computers in Biology and Medicine		n	FLAIR	Markov Random Field, Mixture Model	IN:0-255 range; BE:in house	2.66GHz 512 MB RAM	21 sec	n	n	MS	-	20	3	n	Manual, 2		F1; Sen: Extra fraction; VC
J	2008	R. Khayati et al.	A novel method for automatic determination of different stages of multiple sclerosis lesions in brain MR FLAIR images	Computerized Medical Imaging and Graphics		n	FLAIR	Markov Random Field, Mixture Model	BE:ref	Pentium 4 2.66GHz 512 MB RAM	n/a	acute (recent/ early); chronic	n	MS	MS	20	3	n	Manual, 2		DSC; Sen; Extra Fraction
J	2008	T. B. Dyrby et al.	Segmentation of age-related white matter changes in a clinical multi-center study	NeuroImage		y – adaptable to single and multi	T1, T2, FLAIR	Artificial neural networks	IN:polynomial fitting & z-score; REG:rigid(SPM2); resampling:b-spline interpolation; BE:SPM2; BFC:N3	n/a	n/a	n	n	WML	-	362	3 (LADIS, Pantoni et al. 2005)	y	Semiautomatic		SI; Sen

For peer review only

J	2008	J. F. A. Jansen et al.	White Matter Lesions in Patients With Localization-Related Epilepsy	Investigative Radiology		y	T1, T2, FLAIR	Other	REG; IN; BE:ref; tissue maps	n/a	n/a	n	n	WML	EP	33 + 16 healthy	3	n	Expert segmentation		ICC; VC
J	2008	E. Herskovits et al.	Automated Bayesian Segmentation of Microvascular White-Matter Lesions in the ACCORD-MIND Study	Advances in medical sciences	Semiautomatic preprocessing (brain extraction)	y	T1, T2, FLAIR, SD	Bayesian classification, Other	REG: rigid; BE:ref; tissue maps; IN:histogram matching; smoothing:Gaussian kernel	Silicon Graphics (Mountain View, CA) Origin 300 workstation	4 min	n	n	WML	-	42	3 (ACCORD-MIND)	y	Manual, 1		TPR; FPR; DSC; ROC
J	2008	J. J. Corso et al.	Efficient Multilevel Brain Tumor Segmentation With Integrated Bayesian Model Classification	IEEE Transactions on Medical Imaging		y	T1, T1c, T2, FLAIR	Other	REG:FSL; denoising:FSL; BE:FSL; IN:FSL	1.67 Gz 1.5 GB	<2 min	n	n	BT	GBM	20	3	n/a	Expert segmentation		Jcc
J	2005	F. Admiraal-Behloul et al.	Fully automatic segmentation of white matter hyperintensities in MR images of the elderly	NeuroImage	Inter-rater agreement evaluated, Scan-rescan reproducibility	y	PD; T2, FLAIR	Fuzzy C-Means	REG:rigid(AIR); tissue maps: affine transformation from MNI; BE:FCM	Pentium 4 512MB RAM	2 min	n	n	WML	-	100; 44 (different slice thickness)	3 (PROSPER, Shepherd et al. 1999)	n	Manual, 2		ICC; Bland-Altman; DSC
J	2005	O. Colliot et al.	Segmentation of focal cortical dysplasia lesions on MRI using level set evolution	NeuroImage	Inter-rater agreement evaluated, Segmentation failure	n	T1	Level Set	IN; BFC; REG; BE: BET; tissue maps:thresholding	1.6GHz	14 min	n	n	FCD	FCD	24	3	n	Manual, 2	consensus	SI; FP; other
J	2004	W. Li et al.	Robust unsupervised segmentation of infarct lesion from diffusion tensor MR images using multiscale statistical classification and partial volume voxel reclassification	Computational Diffusion MRI	Inter-rater agreement evaluated	n	DTI	Other	BFC & denoising:ADF	n/a	n/a	n	n	S	-	20	3	y	Manual		DSC
J	2004	P. Anbeek et al.	Automatic segmentation of different-sized white matter lesions by voxel probability estimation	Medical Image Analysis		y	T1, IR, T2, PD, FLAIR	K-Nearest Neighbours, Thresholding	IN:histogram matching; REG:rigid; BE:Mbrase	n/a	n/a	n	n	WML	Arterial VaD	19	3	n	Manual, 2	supervision	TPR; FPR; DSC; overlap fraction; error fraction; other
J	2002	A. P. Zijdenbos et al.	Automatic 'pipeline' analysis of 3-D MRI data for clinical trials: application to multiple sclerosis	IEEE Transactions on Medical Imaging		y	T1, T2, PD	Artificial neural networks	IN:adaptive classification; denoising:ADF; BFC:N3; REG; resampling	n/a	n/a	n	n	MS	-	10; 29	3 (Weiner et al. 1997)	y	Manual, 7; Semiautomatic		other
J	2001	K. Van Leemput et al.	Automated segmentation of multiple sclerosis lesions by model outlier detection	IEEE Transactions on Medical Imaging	Inter-rater agreement evaluated	y	PD, T2	Other	REG:affine; BFC:EM	n/a	n/a	n	n	MS	-	10x2 + 3 (HiRes)	3 (BIOMORPH, 1996–1998)	n	Manual, 1&2		DSC; VC
J	1998	L. P. Clarke et al.	MRI Measurement of Brain Tumor Response: Comparison of Visual Metric and Automatic Segmentation	Magnetic Resonance Imaging		y	T1, T2, PD	Fuzzy C-Means	n/a	n/a	n/a	n	n	BT	-	6x(2-5)	3	n	Manual, multiple		VC
J	1997	S. Dickson et al.	Using Neural Networks to Automatically Detect Brain Tumours in MR Images	International Journal of Neural Systems		n	T1c	Artificial neural networks	n/a	n/a	n/a	n	n	BT	Acoustic neuroma	50	3	n	Manual		other
J	1995	M. Kamber et al.	Model-based 3-D segmentation of multiple sclerosis lesions in magnetic resonance brain images	IEEE Transactions on Medical Imaging	Inter-rater agreement evaluated, Segmentation failure, Manual corrections of preprocessing	y	PD, T2	Other	BE; BFC:homomorphic filtering	n/a	n/a	n	n	MS	MS	12; 44	3	n	Manual, 1		other
C	2019	S. N. Shivhare et al.	An Efficient Brain Tumor Detection and Segmentation in MRI Using Parameter-Free Clustering	Machine Intelligence and Signal Analysis		n	T1c	K-Means, Other	n/a	n/a	n/a	n	n	BT	HGG	273	1 (BraTS'15)	y	-		DSC
C	2018	S. M. Anwar et al.	Brain tumor segmentation on Multimodal MRI scans using EMAP Algorithm	IEEE EMBC		y	T2; FLAIR	K-Means, Expectation-Maximization	BFC:N4r; IN:0-1 range	i7 16BG RAM	comparable to previous techniques	n	n	BT	LGG; HGG	40	1 (BraTS'15)	y	-		DSC; Jcc; PPV; Sen; Kappa
C	2018	D. Jin et al.	White matter hyperintensity segmentation from T1 and FLAIR images using fully convolutional neural networks enhanced with residual connections	ISBI		y	T1, FLAIR	Artificial neural networks	IN:0-1 range; tissue map	n/a	n/a	n	n	WML	-	170	1 (WMH Segmentation Challenge '17)	y	-		DSC; Hdff; avgVD; Sen; F1
C	2018	A. F. I. Osman	Automated Brain Tumor Segmentation on Magnetic Resonance Images and Patient's Overall Survival Prediction Using Support Vector Machines	Brainlesion: Glioma, Multiple Sclerosis, Stroke and Traumatic Brain Injuries		y	T1, T1c, T2, FLAIR	Support Vector Machine	IN:max global intensity + thresholding; denoising:median filtering	n/a	n/a	Overall survival:short, medium, long	n	BT	LGG; HGG	477	1 (BraTS'17)	y	-		DSC
C	2018	Y. Jiang et al.	A Brain Tumor Segmentation New Method Based on Statistical Thresholding and Multiscale CNN	Intelligent Computing Methodologies		y	T1, T1c, T2, FLAIR	Artificial neural networks, Thresholding	denoising:median filtering	n/a	n/a	n	n	BT	LGG; HGG	274	1 (BraTS'15)	y	-		DSC; PPV; Sen
C	2018	L. Chen et al.	MRI tumor segmentation with densely connected 3D CNN	Medical Imaging 2018: Image Processing		y	T1, T1c, T2, FLAIR	Artificial neural networks	BFC:N4; IN:0u1v	n/a	n/a	n	n	BT	-	210	1 (BraTS'17)	y	-		DSC
C	2018	Y. Xu et al.	White Matter Hyperintensities Segmentation in a Few Seconds Using Fully Convolutional Network and Transfer Learning	Brainlesion: Glioma, Multiple Sclerosis, Stroke and Traumatic Brain Injuries		y	T1, FLAIR	Artificial neural networks	BFC; morphological operations	NVIDIA GeForce GTX 1080	10 sec	n	n	WML	-	60 + 110 (testing)	1 (WMH Segmentation Challenge '17)	y	-		DSC; Hdff; avgVD; Sen; F1

For peer review only

C	2018	M. Soltaninejad et al.	MRI Brain Tumor Segmentation and Patient Survival Prediction Using Random Forests and Fully Convolutional Networks	Brainlesion: Glioma, Multiple Sclerosis, Stroke and Traumatic Brain Injuries		y	T1, T2, FLAIR	Artificial neural networks, Random Forest	IN:1-99% clipping + 0u1v + histogram matching	i7 16BG RAM GTX980i	n/a	overall survival; long; medium; short	n	BT	-	497	1 (BraTS'17)	y	-		DSC; Sen; Spe; Hdff
C	2017	M. Bento et al.	Probabilistic Segmentation of Brain White Matter Lesions Using Texture-Based Classification	Image Analysis and Recognition		y	T1, T2, DWI, FLAIR	Thresholding, Support Vector Machine, K-Nearest Neighbours, Random Forest	resizing; tissue maps:semi-automatic region growing; IN:0-255 range	n/a	n/a	n	n	S	S	28	1 (ISLES'15)	y	-		DSC; Sen; Spe; Acc
C	2018	M. Bento et al.	WMH Segmentation Challenge: A Texture-Based Classification Approach	Brainlesion: Glioma, Multiple Sclerosis, Stroke and Traumatic Brain Injuries		y	T1, FLAIR	Random Forest	cropping; resizing; tissue map; IN:0-255 range	Intel Zeon 12-Core 2.7 GHz 64GB DDR3 RAM	90 sec	n	n	WML	-	60 + 110 (testing)	1 (MS GC MICCAI'08)	y	-		DSC; Hdff; avgVD; Sen; F1
C	2018	M. Shaikh et al.	Brain Tumor Segmentation Using Dense Fully Convolutional Neural Network	Brainlesion: Glioma, Multiple Sclerosis, Stroke and Traumatic Brain Injuries		y	T1, T1c, T2, FLAIR	Artificial neural networks	IN:z-score	Titan X GPU	<30 sec	n	n	BT	LGG; HGG	284; 29	1 (BraTS'17)	y	-		DSC
C	2018	Y. Hu and Y. Xia	3D Deep Neural Network-Based Brain Tumor Segmentation Using Multimodality Magnetic Resonance Sequences	Brainlesion: Glioma, Multiple Sclerosis, Stroke and Traumatic Brain Injuries		y	T1, FLAIR	Artificial neural networks	IN:0-255 range	Intel Xeon 2.10 GHz CPU, NVIDIA GTX 1080 Ti GPU	3 min	n	n	BT	LGG; HGG	477	1 (BraTS'17)	y	-		DSC; Hdff
C	2018	Z. Kapás et al.	Automatic Brain Tumor Segmentation in Multispectral MRI Volumes Using a Random Forest Approach	Image and Video Technology		y	T1, T1c, T2, FLAIR	Random Forest	IN:linear transformation	I7 3.4GHz	45 sec	n	n	BT	HGG	220	1 (BraTS'16)	y	-		DSC; Sen; Spe
C	2018	L. Vidyaratne et al.	Deep learning and texture-based semantic label fusion for brain tumor segmentation	Proceedings of SPIE--the International Society for Optical Engineering		y	T1, T1c, T2, FLAIR	Artificial neural networks, Random Forest	BFC:N4; IN:ref	n/a	n/a	n	n	BT	HGG	243	1 (BraTS'17)	y	-		DSC
C	2018	S. Doyle et al.	Sub-acute and Chronic Ischemic Stroke Lesion MRI Segmentation	Brainlesion: Glioma, Multiple Sclerosis, Stroke and Traumatic Brain Injuries		y	T1, FLAIR	Other	IN: to a reference image; BFC:N4; REG:ITK; tissue maps:ITK	n/a	n/a	n	n	S	-	37; 47	1 (Maier et al. 2015); 3 (HERMES)	y	Manual, 1		DSC; Hdff; avgSD; PPV; Sen; VD
C	2018	S. Amiri et al.	Bayesian Network and Structured Random Forest Cooperative Deep Learning for Automatic Multi-label Brain Tumor Segmentation	International Conference on Agents and Artificial Intelligence		y	T1, T1c, FLAIR	Artificial neural networks, Random Forest	BFC:N4; IN:histogram linear transformation	n/a	n/a	n	n	BT	HGG	220	1 (BraTS'15)	y	-		DSC
C	2018	G. C. Oliveira et al.	Brain Tumor Segmentation in Magnetic Resonance Images using Genetic Algorithm Clustering and AdaBoost Classifier	BIOIMAGING			n/a	boosting, Genetic Algorithm	n/a	I7 2.4GHz 8GB RAM	6.22 min	n	n	BT	LGG	42	1 (BraTS'15)	y	-		DSC
C	2018	G. Wang et al.	Automatic Brain Tumor Segmentation Using Cascaded Anisotropic Convolutional Neural Networks	Brainlesion: Glioma, Multiple Sclerosis, Stroke and Traumatic Brain Injuries		y	T1, T1c, T2, FLAIR	Artificial neural networks	n/a	NVIDIA TITAN X GPU	n/a	n	n	BT	LGG; HGG	285; 46; 146	1 (BraTS'17)	y	-		DSC; Hdff
C	2018	D. Bhattacharya and N. Sinha	An improved approach of high graded glioma segmentation using sparse autoencoder and fuzzy c-means clustering from multi-modal MR images	SPIE Medical Imaging		y	T1, T1c, T2, FLAIR	Artificial neural networks	none	n/a	n/a	n	n	BT	HGG	15	1 (BraTS'15)	y	-		DSC; Jcc
C	2018	H. N. Bharath et al.	Tumor Segmentation from Multimodal MRI Using Random Forest with Superpixel and Tensor Based Feature Extraction	Brainlesion: Glioma, Multiple Sclerosis, Stroke and Traumatic Brain Injuries		y	T1, T1c, T2, FLAIR	Random Forest	IN:0-1 range + histogram equalization; backgound removal:Otsu's thresholding	n/a	n/a	n	n	BT	HGG	285; 46; 146	1 (BraTS'17)	y	-		DSC; Sen; Hdff
C	2018	R. Pourreza et al.	Brain Tumor Segmentation in MRI Scans Using Deeply-Supervised Neural Networks	Brainlesion: Glioma, Multiple Sclerosis, Stroke and Traumatic Brain Injuries		y	T1c, T2, FLAIR	Artificial neural networks	BFC:N4; IN:histogram mathcing	NVIDIA Titan Xp	30 sec	n	n	BT	LGG; HGG	285	1 (BraTS'17)	y	-		DSC
C	2018	M. F. Rachmadi et al.	Automatic Irregular Texture Detection in Brain MRI Without Human Supervision	Medical Image Computing and Computer Assisted Intervention – MICCAI 2018		n	FLAIR	Other	BE:optiBET, tissue map:in-house	GPU	13 min	n	n	WML	-	20	2 (ADNI)	y	n/a		DSC
C	2017	C. Ö. Fiçici et al.	Fully Automated Brain Tumor Segmentation and Volume Estimation Based on Symmetry Analysis in MR Images	CMBEBIH		y	T1, T1c, FLAIR	Fuzzy C-Means, Other	denoising:median filtering; cropping; BE:thresholding	n/a	n/a	n	n	BT	-	10	3	n	Expert segmentation		DSC; Jcc; Sen; Spe;
C	2017	S. R. Mote et al.	Non-negative matrix factorization and self-organizing map for brain tumor segmentation	2017 International Conference on Wireless Communications, Signal Processing and Networking (WiSPNET)		y	T2, FLAIR	Self Organising Maps	BFC & denoising:ADF	n/a	n/a	n	n	BT	LGG; HGG	30 (+25 synthetic)	1 (BraTS'12)	y	-		DSC; Sen; Spe
C	2017	A. López-Zorrilla et al.	Brain White Matter Lesion Segmentation with 2D/3D CNN	Natural and Artificial Computation for Biomedicine and Neuroscience		y	T1, FLAIR, DTI	Artificial neural networks	REG:affine; IN:0-1 range	GTX 1070 16GB RAM	n/a	n	n	WML	AD, VaD	18	3 (Price et al. 2012)	n	Manual		TPR
C	2017	H. Shen et al.	Multi-task Fully Convolutional Network for Brain Tumour Segmentation	Medical Image Understanding and Analysis		y	T1, T1c, T2, FLAIR	Artificial neural networks	n/a	n/a	n/a	n	n	BT	HGG	30; 220	1 (BraTS'13); 1 (BraTS'15)	y	-		DSC; Sen; PPV; F-score

For peer review only

C	2017	S. Pereira et al.	On hierarchical brain tumor segmentation in MRI using fully convolutional neural networks: A preliminary study,	ENBENG	y	T1, T1c, T2, FLAIR	Artificial neural networks	IN:histogram normalization + 0u1v	I7 3.2GHz 48GB RAM GTX970 GPU	40 sec	n	n	BT	-	40	1 (BraTS'13)	y	-	DSC; PPV; Sen
C	2017	H. Dong et al.	Automatic Brain Tumor Detection and Segmentation Using U-Net Based Fully Convolutional Networks	Medical Image Understanding and Analysis	y	T1c, FLAIR	Artificial neural networks	IN:0u1v	Titan X GPU 12Gb RAM	3 sec	n	n	BT	LGG; HGG	274	1 (BraTS'15)	y	-	DSC; Sen
C	2017	M. Rezaei et al.	Deep Neural Network with l2-Norm Unit for Brain Lesions Detection	Neural Information Processing	y	T1,T2, DWI, FLAIR	Artificial neural networks	n/a	n/a	n/a	LGG; HGG, AD; S	n	BT, S	LGG; HGG; S	328; 30	1 (BraTS'16); 1 (ISLES'16)	y	-	DSC
C	2017	S. Hussain et al.	Brain tumor segmentation using cascaded deep convolutional neural network	Annual International Conference of the IEEE	y	T1, T1c, T2, FLAIR	Artificial neural networks	BFC:N4; IN:0u1v	n/a	n/a	n	n	BT	LGG; HGG	30 +25 synthetic	1 (BraTS'13)	y	-	DSC; Sen; Spe
C	2017	W. Chen et al.	Automatic brain tumor segmentation based on features of separated local square	Chinese Automation Congress	n	FLAIR	Support Vector Machine	BFC:N4; IN:histogram matching	n/a	n/a	n	n	BT	LGG; HGG	20 (randomly selected from 30)	1 (BraTS'13)	y	-	DSC; Sen; Spe
C	2017	G. Latif et al.	Automatic Multimodal Brain Image Classification Using MLP and 3D Glioma Tumor Reconstruction	IEEE-GCC Conference and Exhibition	y	T1, T1c, T2, FLAIR	Other, Active Contour	denoising: ADF; BE:thresholding + morphologica operations	n/a	n/a	normal, abnormal	n	BT	LGG; HGG	160	1 (BraTS'15)	y	-	DSC; Spe; Sen; Acc; other
C	2017	W. Mengqiao et al.	The multimodal brain tumor image segmentation based on convolutional neural networks	IEEE International Conference on Computational Intelligence and Applications	y	T1, T1c, T2, FLAIR	Artificial neural networks	BFC:N4; IN:0u1v	I7 2.8GHz GPU GeForce 1050	n/a	n	n	BT	LGG; HGG	120	1 (BraTS'15)	y	-	DSC; PPV; Sen
C	2017	V. Shreyas et al.	A deep learning architecture for brain tumor segmentation in MRI images	International Workshop on Multimedia Signal Processing	y	T1, T1c, T2, FLAIR	Artificial neural networks	BFC:N4; IN:histogram matching + z-score	i7 128GB RAM	13 sec	n	n	BT	HGG	220	1 (BraTS'15)	y	-	DSC; PPV; Sen
C	2017	N. Shah et al.	Brain tumor segmentation and classification using cascaded random decision forests	International Conference on Electrical Engineering/Electronics, Computer, Telecommunications and Information Technology	y	T1, T1c, T2, FLAIR	Random Forest	IN:histogram matching	n/a	n/a	n	n	BT	LGG; HGG	30 +50 simulated	1 (BraTS'13)	y	-	DSC; Jcc
C	2017	U. Baid et al.	Novel approach for brain tumor segmentation with non negative matrix factorization	International Conference on Innovations in Electronics, Signal Processing and Communication	y	T2, FLAIR	Fuzzy C-Means, Matrix Factorization	denoising:ADF; BFC:N3	n/a	n/a	n	n	BT	LGG; HGG	30 +50 simulated	1 (BraTS'12)	y	-	DSC; Sen; Spe; Hdff
C	2017	M. K. Akter et al.	Automated brain tumor segmentation from mri data based on exploration of histogram characteristics of the cancerous hemisphere	IEEE Region 10 Humanitarian Technology Conference	y	T1, T1c, T2, FLAIR	Thresholding, Other	n/a	n/a	n/a	n	n	BT	HGG	15	1 (BraTS'12)	y	-	DSC; Sen; Spe
C	2017	S. Goel et al.	Brain tumor segmentation in multispectral MR images	International Conference on Signal Processing and Integrated Networks	y	T1, T1c, T2, FLAIR	Fuzzy C-Means	BFC:N3; IN:thresholding + contrast enhancement, image fusion; smoothing	n/a	n/a	n	n	BT	HGG	15	1 (BraTS'13)	y	-	DSC; PPV; Sen
C	2017	A. Sankari and S. Vigneshwari	Automatic tumor segmentation using convolutional neural networks	International Conference on Science Technology Engineering Management	n/a	n/a	Artificial neural networks	denoising; IN; BFC; contrast enhancement:histogram equalization	n/a	n/a	n	n	BT	LGG; HGG	391	1 (BraTS'15); 1 (IBSR)	y	-	Sen; Spe; acc
C	2016	R. Liu et al.	Multi-modal Brain Tumor Segmentation Based on Self-organizing Active Contour Model	Chinese Conference on Pattern Recognition	y	T1, T1c, T2, FLAIR	Self Organising Maps, Active Contour	IN:histogram normalization; contrast adjustment:ITKSnap	n/a	n/a	n	n	BT	LGG; HGG	45	1 (BraTS'13); 1 (BraTS'15)	y	-	DSC; Sen; Spe
C	2016	Y. Wang et al.	A deep symmetry convnet for stroke lesion segmentation	IEEE ICIP	n	T1	Artificial neural networks	BFC:AFNI; BE:SymROBEX; IN:0u1v; contrast enhancement:histogram equalization	n/a	n/a	n	n	S	Aphasia due to S	18	3	n	Manual	DSC
C	2016	B. Song et al.	Anatomy-Guided Brain Tumor Segmentation and Classification	Brainlesion: Glioma, Multiple Sclerosis, Stroke and Traumatic Brain Injuries	y	T1, T1c, T2, FLAIR	Random Forest, Grow Cut	BFC:N4; IN:linear normalization of tissue histograms	2.67GHz 24 GB RAM	1 min	n	n	BT	LGG; HGG	274	1 (BratTS'15); 1 (BraTS'16)	y	-	DSC; Hdff
C	2016	E. Ilunga-Mbuyamba et al.	Automatic brain tumor tissue detection based on hierarchical centroid shape descriptor in Tl-weighted MR images	2016 International Conference on Electronics, Communications and Computers (CONIELECOMP)	n	T1	K-Means, Other	BE: thresholding + morphological operations	1.5GHz 2GB RAM	n/a	n	n	BT	-	254	3 (University Hospital, Department of Neurosurgery, University of Leipzig, Germany)	n	Expert segmentation	DSC; Jcc
C	2016	M. Agn et al.	Brain Tumor Segmentation Using a Generative Model with an RBM Prior on Tumor Shape	Brainlesion: Glioma, Multiple Sclerosis, Stroke and Traumatic Brain Injuries	y	T1, T1c, T2, FLAIR	Artificial neural networks	BFC:log transform	i7 CPU, GTX Titan Black GPU	30 min	n	n	BT	LGG; HGG	65; 297	1 (BraTS'13); 1 (BraTS'15)	y	-	DSC
C	2015	M. Havaei et al.	A Convolutional Neural Network Approach to Brain Tumor Segmentation	Brainlesion: Glioma, Multiple Sclerosis, Stroke and Traumatic Brain Injuries	y	T1, T1c, T2, FLAIR; T1, DWI, FLAIR	Artificial neural networks	n/a	Nvidia Titan black GPU	25 sec – 3 min	n	n	BT, S	LGG; HGG; S	40; 327; 114	1 (BraTS'13); 1 (BraTS'15); 1 (ISLES'15)	y	-	DSC; Sen; Spe; avgSD; Hdff
C	2016	X. Chen et al.	Automated brain tumor segmentation using kernel dictionary learning and superpixel-level features	IEEE SMC	y	T1, T1c, T2, FLAIR	Sparse representation, Graph Cut	BFC:N4; IN:histogram matching	I7 3.2GHz 32GB RAM	30 sec	n	n	BT	HGG	20; 10	1 (BraTS'13)	y	-	DSC; Jcc; Sen
C	2016	Q. Mahmood and A. Basit	Prediction of Ischemic Stroke Lesion and Clinical Outcome in Multi-modal MRI Images Using Random Forests	Brainlesion: Glioma, Multiple Sclerosis, Stroke and Traumatic Brain Injuries	y	DWI, PWI	Random Forest	n/a	15 2.5GHz	5 min	clinical outcome – medical ranking score	n	S	S	49	1 (ISLES'16)	y	-	avgSSD; Hdff; DSC
C	2015	Q. Mahmood and A. Basit	Automatic ischemic stroke lesion segmentation in multi-spectral mri images using random forests classifier	MICCAI BrainLes	y	T1, T2, DWI, FLAIR	Random Forest	BFC:N3; IN:0-1 range	I5 2.5 GHz 4GB RAM	~30 min	n	n	S	S	64	1 (ISLES'15)	y	-	avgSSD; Hdff; DSC; PPV; Sen
C	2016	K. Vaidhya et al.	Multi-modal Brain Tumor Segmentation Using Stacked Denoising Autoencoders	Brainlesion: Glioma, Multiple Sclerosis, Stroke and Traumatic Brain Injuries	n/a	n/a	Artificial neural networks	IN:histogram matching + mean division and outlier removal	n/a	30 min	n	n	BT	LGG; HGG	274	1 (BraTS'15)	y	-	DSC
C	2016	S. Amiri et al.	Deep random forest-based learning transfer to SVM for brain tumor segmentation	International Conference on Advanced Technologies for Signal and Image Processing	n	FLAIR	Random Forest, Support Vector Machine	IN; contrast enhancement	n/a	n/a	n	n	BT	HGG	20	1 (BraTS'13)	y	-	DSC
C	2016	S. Müller et al.	Automatic brain tumor segmentation with a fast Mumford-Shah algorithm	SPIE Medical Imaging	y	T1c, T2, FLAIR	Thresholding, Other	BFC:N4; IN histogram equalization + 0-225 range	I7 3.4GHz 16BG RAM GTX 970 4GB RAM	<1 min	n	n	BT	LGG; HGG	213	1 (BraTS'14)	y	-	DSC; Sen; PPV
C	2016	E. A. R. Piedra et al.	Brain Tumor Segmentation by Variability Characterization of Tumor Boundaries	Brainlesion: Glioma, Multiple Sclerosis, Stroke and Traumatic Brain Injuries	y	T1, T1c, T2, FLAIR	Other	tissue maps	n/a	n/a	n	n	BT	-	220	1 (BraTS'15)	y	-	DSC; Hdff
C	2016	Z. Xiao et al.	A deep learning-based segmentation method for brain tumor in MR images	International Conference on Computational Advances in Bio and Medical Sciences	n/a	n/a	Artificial neural networks	cropping; IN	n/a	n/a	n	n	BT	-	10	3 (West China Hospital)	n	Manual	percentage match
C	2016	A. Sehgal et al.	Automatic brain tumor segmentation and extraction in MR images	Conference on Advances in Signal Processing	n	T1	Fuzzy C-Means, Other	BFC:N3; denoising:ADF; enhancement:unsharp mask	n/a	n/a	n	n	BT	-	30	1 (BraTS'13)	y	-	DSC
C	2016	A. Derntl et al.	Stroke Lesion Segmentation Using a Probabilistic Atlas of Cerebral Vascular Territories	Brainlesion: Glioma, Multiple Sclerosis, Stroke and Traumatic Brain Injuries	y	not specific to a modality	Expectation-Maximization Gausian mixture model, Conditional Random Field, Other,	REG; BFC; BE	n/a	n/a	n	n	S	S	13	3	n/a	Manual	DSC

For peer review only

C	2015	Y. Li et al.	Automatic Brain Tumor Segmentation from MR Images via a Multimodal Sparse Coding Based Probabilistic Model	International Workshop on Pattern Recognition in NeuroImaging		y	T1, T1c, T2, FLAIR	Sparse representation, Markov Random Field	REG:rigid; BE; IN:0-1 range	n/a	n/a	n	n	BT	-	30	1 (BraTS'12)	y	-		DSC; Sen; Spe
C	2015	M. Lyksborg et al.	An Ensemble of 2D Convolutional Neural Networks for Tumor Segmentation	Scandinavian Conference on Image Analysis		y	T1, T1c, T2, FLAIR	Artificial neural networks	BFC:N4; IN:histogram scaling	n/a	n/a	n	n	BT	-	131; 187 (longitudinal)	1 (BraTS'14)	y	-		DSC; PPV; Sen
C	2015	R. Mechrez et al.	MS lesion segmentation using a multi-channel patch-based approach with spatial consistency	Medical Imaging 2015: Image Processing		y	T1, T2, FLAIR	Non-local Means, K-Nearest Neighbours, Other,	BE; BFC; sub-sampling; IN	n/a	n/a	n	n	MS	-	10	1 (MS GC MICCAI'08)	n	-		DSC; TPR; PPV
C	2015	I. Zabir et al.	Automatic brain tumor detection and segmentation from multi-modal MRI images based on region growing and level set evolution	2015 IEEE International WIE Conference on Electrical and Computer Engineering (WIECON-ECE)		y	T2, FLAIR	K-Nearest Neighbours, Thresholding, region growing, level set	n/a	n/a	n/a	n	n	BT	-	17	1 (BraTS'12)	y	-		DSC; Jcc; Sen; Spe
C	2015	A. Jog et al.	Multi-output decision trees for lesion segmentation in multiple sclerosis	Proceedings of SPIE--the International Society for Optical Engineering		y	T1, T2, FLAIR	Decision Tree	BFC:N4; BE:ref; REG:ref; IN	n/a	2 min	n	n	MS	-	20; 49	1 (MS GC MICCAI'08); 3	y	Manual		PPV; TPR
C	2015	H. Tang et al.	Tumor segmentation from single contrast MR images of human brain	2015 IEEE 12th International Symposium on Biomedical Imaging (ISBI)	Segmentation failure	n	T2	Random Forest	REG:rigid+affine; IN:linear scaling to MNI	n/a	n/a	n	n	BT	LGG; HGG	30	1 (BraTS'12)	y	-		DSC
C	2015	L. Szilágyi et al.	Automatic Brain Tumor Segmentation in Multispectral MRI Volumetric Records	Neural Information Processing		y	T1, T1c, T2, FLAIR	Fuzzy C-Means	n/a	n/a	n/a	n	n	BT	-	13	1 (BraTS'12)	y	-		Jcc; DSC
C	2015	Y. Karpate et al.	Probabilistic one class learning for automatic detection of multiple sclerosis lesions	2015 IEEE 12th International Symposium on Biomedical Imaging (ISBI)		y	T1, T2, FLAIR	Thresholding, probabilistic classification	n/a	n/a	n/a	n	n	MS	-	16 + 20 controls	3	n	Manual, 1		PPV; Sen
C	2015	H. Deshpande et al.	Adaptive dictionary learning for competitive classification of multiple sclerosis lesions	2015 IEEE 12th International Symposium on Biomedical Imaging (ISBI)		y	T1, T2, PD, FLAIR	Sparse representation, Dictionary Learning	BFC; denoising:non-local means; REG:BE; ref	n/a	n/a	n	n	MS	MS	13	3	n	Manual, 1		Sen; PPV
C	2015	I. Gondra and I. Cabria	Automated segmentation of brain tumors in MRI using potential field clustering	International Conference on Computer as a Tool		n	FLAIR	Region Growing, Other	n/a	n/a	n/a	n	n	BT	-	22	1 (BraTS'12)	y	-		other
C	2015	A. Pinto et al.	Brain Tumour Segmentation based on Extremely Randomized Forest with high-level features	Annual International Conference of the IEEE Engineering in Medicine and Biology Society (EMBC)		y	T1, T1c, T2, FLAIR	Decision Tree	BFC:ref; IN: histogram matching	n/a	n/a	n	n	BT	LGG; HGG	40	1 (BraTS'13)	y	-		DSC; PPV; Sen
C	2015	K. B. Vaishnavee and K. Amshakala	An automated MRI brain image segmentation and tumor detection using SOM-clustering and Proximal Support Vector Machine classifier	IEEE International Conference on Engineering and Technology		n	T1	Self Organising Maps, Support Vector Machine	BE:BET; enhancement:histogram equalization	n/a	n/a	n	n	BT	-	20	1 (IBSR); 3 (Medicine Service of the "Virgen de las Nieves" Hospital, Granada, Spain)	y	Manual		TI
C	2015	A. Pinto et al.	Random decision forests for automatic brain tumor segmentation on multi-modal MRI images	IEEE Portuguese Meeting on Bioengineering		y	T1, T1c, T2, FLAIR	Random Forest	BFC:N4; IN:histogram matching	I7 3.2GHz 47GB RAM	n/a	n	n	BT	LGG; HGG	30	1 (BraTS'13)	y	-		DSC; PPV; Sen
C	2015	Y. Renping et al.	Automatic Segmentation of White Matter Lesions Using SVM and RSF Model in Multi-channel MRI	Image and Graphics		y	T1, T2, PD, FLAIR	Support Vector Machine, Other	REG:rigid (MI); BE:ref; BFC:N3; IN:in paper	n/a	n/a	n	n	WML	-	45	3 (ACCORD-MIND)	n/a	Manual, 1		DSC; TPR; TNR; PPV
C	2015	N. Nabizadeh et al.	Automatic tumor lesion detection and segmentation using modified winnow algorithm	ISBI	Detection followed by segmentation	n	FLAIR	Active Contour, Level Set	IN:histogram normalization	n/a	n/a	n	n	BT	HGG	25 +25 simulated	1 (BraTS'13)	y	-		Acc
C	2015	S. E. El-Khamy et al.	An efficient brain mass detection with adaptive clustered based fuzzy C-mean and thresholding	International Conference on Signal and Image Processing Applications		n/a	n/a	Fuzzy C-Means	enhancement:intensity scaling	I5 2.3GHz 4GB RAM	~5 sec	n	n	BT	T	15	3	n/a	Expert segmentation		Acc, other
C	2015	A. F. Muda et al.	Integration of Fuzzy C-Means with Correlation Template and Active Contour for Brain Lesion Segmentation in Diffusion-Weighted MRI	International Conference on Artificial Intelligence, Modelling and Simulation		n	DWI	Fuzzy C-Means, Active Contour, Other,	Ref	n/a	n/a	n	n	BT, S	-	40	3	n/a	Manual		Jcc; DSC; FPR; FNR
C	2015	A. F. Muda et al.	Brain Lesion Segmentation Using Fuzzy C-Means On Diffusion-Weighted Imaging	Journal of Engineering and Applied Sciences		n	DWI	Fuzzy C-Means, Edge detection, Watershed	Ref	n/a	n/a	n	n	BT, S	T, S	20	3 (General Hospital of Kuala Lumpur)	y	Manual		FPR; FNR; DSC; other
C	2014	S. D. S. Al-Shaikhli et al.	Coupled Dictionary Learning for Automatic Multi-Label Brain Tumor Segmentation in Flair MRI images	Advances in Visual Computing		n	FLAIR; T1	Graph Cut, Dictionary Learning	n/a	2GHz CPU	n/a	n	n	BT	-	10; 30	1 (BraTS'13); 1 (SPL)	y	-		DSC; PPV; Sen; Jcc; Kappa
C	2014	H. J. Kuijf et al.	The Added Value of Diffusion Tensor Imaging for Automated White Matter Hyperintensity Segmentation	Computational Diffusion MRI		y	T1, FLAIR, DTI	K-Nearest Neighbours	IN:histogram matching; REG:rigid (elastix)	n/a	n/a	n	n	WML	-	20	3	n	Manual, 2	supervision	DSC; Sen
C	2014	P. K. Roy et al.	Automated Segmentation of White Matter Lesions Using Global Neighbourhood Given Contrast Feature-Based Random Forest and Markov Random Field	2014 IEEE International Conference on Healthcare Informatics		y	T1, FLAIR	Random Forest, Markov Random Field	REG:rigid(SPM8); BE:BET; BFC:N3	n/a	n/a	n	n	WML	HT	24	3 (ENVISion, Reid et al. 2012)	n	Manual, 2	supervision	DSC
C	2014	A. Rao et al.	Contusion segmentation from subjects with Traumatic Brain Injury: A random forest framework	2014 IEEE 11th International Symposium on Biomedical Imaging (ISBI)		y	T1, DTI, FLAIR	Random Forest	BFC:N4; IN:linear scaling; REG:affine; tissue maps	n/a	n/a	n	n	TBI	-	23	3	n	Manual, 1		DSC; TPR; PPV
C	2014	N. Subbanna et al.	Iterative Multilevel MRF Leveraging Context and Voxel Information for Brain Tumour Segmentation in MRI	IEEE Conference on Computer Vision and Pattern Recognition		y	T1, T1c, T2, FLAIR	Bayesian classification, Markov Random Field	BFC; IN	Dell Optiplex 980 I7	75 min	n	n	BT	LGG; HGG	30;25 (challenge sets)	1 (BraTS'12); 1 (BraTS'13)	y	-		DSC
C	2015	S. Reza et al.	Ischemic stroke lesion segmentation using local gradient and texture features	MICCAI ISLES		y	T1, T2, DWI, FLAIR	Random Forest	IN:histogram matching + CSF normalizaiton	n/a	n/a	n	n	S	S	28	1 (ISLES'15)	y	-		DSC
C	2014	S. Reza et al.	Improved brain tumor tissue segmentation using texture features	MICCAI 2014		y	T1, T1c, T2, FLAIR	Random Forest	BFC; IN	n/a	n/a	n	n	BT	LGG; HGG	246	1 (BraTS'13); 1 (BraTS'14)	y	-		DSC; Jcc; PPV; Sen; Kappa
C	2014	H. Hooda et al.	Brain tumor segmentation: A performance analysis using K-Means, Fuzzy C-Means and Region growing algorithm	IEEE International Conference on Advanced Communications, Control and Computing Technologies		n/a	n/a	Fuzzy C-Means	BC: median filtering; BE: Otsu's thresholding + morphological operations	2GHz 2GB RAM	n/a	n	n	BT	-	10	3	n	Manual		TP; other
C	2014	P. Dvorak et al.	Automatic Extraction of Pathological Area in 2D MR Brain Scan	Progress in Electromagnetics Research Symposium		y	T2, FLAIR	Other	symmetry axis detection	n/a	n/a	n	n	BT	-	22	1 (BraTS'12)	y	-		DSC; Acc
C	2014	S. Bauer et al.	Towards automatic MRI volumetry for treatment selection in acute ischemic stroke patients	Annual International Conference of the IEEE Engineering in Medicine and Biology Society		y	T1c, T2, DWI, DSC	Decision Tree, Conditional Random Field	REG:rigid; BE:ref;	n/a	<1 min	n	n	S	S	10	3	n	Manual, 1		DSC; VE
C	2014	A. V. Dalca et al.	Segmentation of Cerebrovascular Pathologies in Stroke Patients with Spatial and Shape Priors	International Conference on Medical Image Computing and Computer-Assisted Intervention		y	FLAIR, T2	Markov Random Field	n/s	n/a	n/a	n	n	WML, S	-	148	3	n/a	Manual		VC
C	2014	F. Rodrigo et al.	Segmentation of Hyperintense Regions Applied to Multiple Sclerosis Lesions	Latin American Congress on Biomedical Engineering	Segmentation failure	n	FLAIR	Thresholding	IN: histogram matching; BE:SPM; REG; enhancement: LP filter + saturation	n/a	n/a	n	n	MS	MS	10	3	n/a	Manual, 1		other

For peer review only

C	2013	Z. Karimaghaloo et al.	Adaptive Voxel, Texture and Temporal Conditional Random Fields for Detection of Gad-Enhancing Multiple Sclerosis Lesions in Brain MRI	International Conference on Medical Image Computing and Computer-Assisted Intervention		y	T1, T1c, T2, PD, FLAIR	Conditional Random Field	BFC; BE; REG; IN:histogram equalization	n/a	n/a	n	n	MS	-	940x2	3	y	Manual, 2	consensus	Sen; PPV; avgFP
C	2013	M. M. Riad et al.	Detection of white matter lesions in cerebral small vessel disease	Medical Imaging 2013: Computer-Aided Diagnosis	Inter-rater agreement evaluated	y	T1, T2, FLAIR	Boosting	REG:FLIRT; BE:BET; BFC:MRF+EM; IN:mean-based	n/a	n/a	n	n	WML	-	100	3 (RUNDMC, van Norden et al. 2011)	n	Manual, 1-3	supervision	ROC; FROC
C	2013	M. Cabezas et al.	A Supervised Approach for Multiple Sclerosis Lesion Segmentation Using Context Features and an Outlier Map	Pattern Recognition and Image Analysis		y	T1, T2, PD, FLAIR	Boosting	BE:BET; denoising:ADF; BFC:N4; REG:affine+non-linear	n/a	n/a	n	n	MS	-	30	3 (Hospital Vall d'Hebron): 3 (Hospital Josep Trueta)	y	Manual, 1		DSC; TPR
C	2013	A. Bianchi et al.	Brain tumor segmentation with symmetric texture and symmetric intensity-based decision forests	Proceedings / IEEE International Symposium on Biomedical Imaging: from nano to macro. IEEE International Symposium on Biomedical Imaging		y	T1, T1c, T2, FLAIR	Decision Tree	IN:mode matching (shifting)	2.4GHz 16GB RAM	1.5 sec	n	n	BT	HGG	20 +50 synthetic	1 (BraTS'12)	y	-		DSC
C	2013	J. Mitra et al.	Classification Forests and Markov Random Field to Segment Chronic Ischemic Infarcts from Multimodal MRI	Multimodal Brain Image Analysis		y	T1, T2, FLAIR, DWI (ADC)	Random Forest, Markov Random Field	resampling; REG; BFC and tissue maps:ref; BE:masking with tissue maps	3.2GHz 23.5GB	13.5 min	n	n	S	-	17	3	n	Manual, 1		DSC; PPV; NPV; Acc
C	2013	M. B. Salah et al.	Fully Automated Brain Tumor Segmentation Using Two MRI Modalities	Advances in Visual Computing	Segmentation failure	y	T1c, FLAIR	Thresholding	BE:in paper; resampling	n/a	n/a	n	n	BT	GBM	19	3 (Cross Cancer Institute, Alberta)	n	Manual, multiple		DSC
C	2013	N. K. Subbanna et al.	Hierarchical Probabilistic Gabor and MRF Segmentation of Brain Tumours in MRI Volumes	Medical Image Computing and Computer-Assisted Intervention – MICCAI 2013		y	T1, T1c, T2, FLAIR	Bayesian classification, Markov Random Field	REG: non-linear	Dell Optiplex 980 i7	~1 hr	n	n	BT	LGG; HGG	45 +65 simulated	1 (BraTS'12)	y	-		DSC
C	2013	I. Diaz et al.	An automatic brain tumor segmentation tool	Annual International Conference of the IEEE Engineering in Medicine and Biology Society		y	T1, T1c, T2, FLAIR	Thresholding, Other	BE:thresholding; IN:histogram matching	1.73 GHz, 6GB RAM	~50 sec	n	n	BT	GBM	16 annotated (+ 60 not)	3 (Cross Cancer Institute, Alberta)	n	Manual, multiple		DSC
C	2013	H. Boussaid et al.	Rapid Mode Estimation for 3D Brain MRI Tumor Segmentation	Energy Minimization Methods in Computer Vision and Pattern Recognition		n/a	n/a	Graph Cut, Other	REG:rigid(medInria)	2.67GHz single core	19 sec	n	n	BT	LGG	113	3	n/a	Manual		DSC
C	2012	V. G. Kanas et al.	Combining Outlier Detection with Random Walker for Automatic Brain Tumor Segmentation	Artificial Intelligence Applications and Innovations		y	T1, T1c, FLAIR	Random Walker	denoising; BFC; REG; BE; IN:histogram matching	n/a	n/a	n	n	BT	meningiona, G, GBM	26	3 (Zacharaki et al. 2009)	n	Expert segmentation		DSC
C	2012	R. S. Ananda and T. Thomas	Automatic segmentation framework for primary tumors from brain MRIs using morphological filtering techniques	International Conference on BioMedical Engineering and Informatics		y	T1-FLAIR, T2	Thresholding, Other	BE:morphological operations	n/a	n/a	n	n	BT	-	50	3 (Sree Chitra Institute of Medical Sciences and Technology (SCIMST)); 3 (Regional Cancer Centre, Thiruvananthapuram, Kerala, Indi)	y	Manual, 1		TI
C	2012	S. Parisot et al.	Joint Tumor Segmentation and Dense Deformable Registration of Brain MR Images	Medical Image Computing and Computer-Assisted Intervention – MICCAI 2012		n	FLAIR	boosting, Markov Random Field	BE; IN:matching median and interquartile range to reference; REG:rigid	n/a	~6 min	n	n	BT	-	93	3	n/a	Manual, 1		DSC; FP; other
C	2012	Z. Karimaghaloo et al.	Hierarchical Conditional Random Fields for Detection of Gad-Enhancing Lesions in Multiple Sclerosis	Medical Image Computing and Computer-Assisted Intervention – MICCAI 2012		n	T1c	Conditional Random Field	BFC; BE; REG; IN	n/a	n/a	n	n	MS	MS	122	3	y	Manual, 2	consensus	Sen; PPV; FP
C	2012	D. Zikic et al.	Decision Forests for Tissue-Specific Segmentation of High-Grade Gliomas in Multi-channel MR	Medical Image Computing and Computer-Assisted Intervention – MICCAI 2012	Fully automatic pre-processing	y	T1, T1c, T2, DTI, FLAIR	Decision Tree, GMM	BE:ref; REG:affine; IN:mean intensity matching	n/a	n/a	n	n	BT	HGG	40	3	n	Manual		DSC
C	2012	D. Zikic et al.	Context-sensitive classification forests for segmentation of brain tumor tissues	Medical Image Computing and Computer-Assisted Intervention – MICCAI 2012		y	T1, T1c, T2, FLAIR	GMM, Decision Tree	BFC: N3; IN: global multiplicative factor	single desktop PC	1-2 min	n	n	BT	LGG, HGG	30 (+25 synthetic)	1 (BraTS'12)	y	-		DSC; Error; Volume Error
C	2012	N. Behzadfar and H. Soltanian-Zadeh	Automatic segmentation of brain tumors in magnetic resonance images	IEEE-EMBS International Conference on Biomedical and Health Informatics		y	T1, T1c, T2, FLAIR	Thresholding, Region Growing, Other,	IN:scaling; BE: LPF + Rodler's method + moprhological operations	n/a	n/a	n	n	BT	GBM	12	3 (Henry Ford Health System, Detroit, MI, USA)	n	Manual, 1		VC; VR
C	2012	I. Mehmood et al.	Automatic Segmentation of Region of Interests in MR Images Using Saliency Information and Active Contours	IT Convergence and Security		n/a	n/a	Other, Active Contour	BE:McStrip	n/a	n/a	n	n	BT	T	n/a (synthetic?); 10	1 (SPL); 3	y	Manual		FROC; DSC
C	2011	S. Bauer et al.	Fully Automatic Segmentation of Brain Tumor Images Using Support Vector Machine Classification in Combination with Hierarchical Conditional Random Field Regularization	Medical Image Computing and Computer-Assisted Intervention – MICCAI 2011		y	T1, T1c, T2, FLAIR	Support Vector Machine, Conditional Random Field	REG:rigid; BE:ref; denoising:edge-preserving smoothing filter; BFC	single CPU 2.33GHz	<120 sec	n	n	BT	-	10	3 (ContraCancrum brain tumor database, Marias et al. 2011)	n	Manual		DSC
C	2010	C. Elliott et al.	Bayesian Classification of Multiple Sclerosis Lesions in Longitudinal MRI Using Subtraction Images	Medical Image Computing and Computer-Assisted Intervention – MICCAI 2010	Scan-rescan reproducibility	y	T1, T2, PD	Bayesian classification	BFC; BE; REG:rigid	n/a	n/a	n	n	MS	-	3x2; 89x(2-4)	3	y	Semiautomatic; Manual		Sen
C	2010	E. Bilotta et al.	A CNN Based Algorithm for the Automated Segmentation of Multiple Sclerosis Lesions	Applications of Evolutionary Computation		n	FLAIR	Artificial neural networks, Genetic Algorithm	tissue maps:SPM8; BE:using WM map	n/a	n/a	n	n	MS	-	11	3	n	Manual, 1		DSC
C	2010	T. Wang et al.	Fully automatic brain tumor segmentation using a normalized Gaussian Bayesian Classifier and 3D Fluid Vector Flow	IEEE International Conference on Image Processing		n/a	n/a	GMM, Other	BE:MIPAV; REG:MIPAV	n/a	~430 sec	n	n	BT	-	10	1 (SPL)	n/a	-		TI
C	2009	J. Liu et al.	Automatic Multiple Sclerosis detection based on integrated square estimation	IEEE Computer Society Conference on Computer Vision and Pattern Recognition Workshops		y	T2, FLAIR	Expectation-Maximization Gausian mixture model, Other	BE:BET; IN:SPM2	1.66GHz 2GB RAM	2 min	n	n	MS	MS	16	3 (University of Kentucky Hospital)	n	Manual, 1		VD; DSC; Sen; Spe
C	2008	E. I. Zacharaki et al.	Measuring Brain Lesion Progression with a Supervised Tissue Classification System	Medical Image Computing and Computer Assisted Intervention – MICCAI 2008	Indirect reproducibility evaluation	y	T1, T2, PD	Support Vector Machine, Boosting	REG:FSL(NMI); BE:BET; BFC:N3; IN:histogram transformation; denoising:Gaussian filter	n/a	n/a	n	n	WML	DM	19 + 23x2	3	n	Manual		other
C	2008	Y. Uchiyama et al.	Automatic segmentation of different-sized leukoaraiosis regions in brain MR images	SPIE Medical Imaging		y	T1, T2	K-Means, Other	BE:region growing	n/a	n/a	n	n	WML	-	73	3 (Gero Hot Springs Hospital, Japan)	n	Manual		ROC

For peer review only

C	2007	X. Xuan and Q. Liao	Statistical Structure Analysis in MRI Brain Tumor Segmentation	International Conference on Image and Graphics		y	T1, T2, FLAIR	boosting	BE; IN	n/a	n/a	n	n	BT	G	10	3	n	Manual		other
C	2007	J. J. Corso et al.	Detection and Segmentation of Pathological Structures by the Extended Graph-Shifts Algorithm	Medical Image Computing and Computer Assisted Intervention – MICCAI 2007		y	T1, T1c, T2, FLAIR	Other	REG;BE;IN:FSL	n/a	~1 min	n	n	BT, MS	GBM; MS	20; 12	3	n/a	Manual		Jcc; PPV; Sen; DR
C	2007	N. Hevia-Montiel et al.	Robust Nonparametric Segmentation of Infarct Lesion from Diffusion-Weighted MR Images	Annual International Conference of the IEEE Engineering in Medicine and Biology Society		n	DWI	Other	ADC thresholding	n/a	n/a	n	n	S	S	15	3 (La Salpêtrière hospital, Paris, France)	n/a	Manual, 1		VC; TI
C	2006	Z. Lao et al.	Automated segmentation of white matter lesions in 3D brain MR images, using multivariate pattern classification	3rd IEEE International Symposium on Biomedical Imaging: Nano to Macro, 2006.	Inter-rater agreement evaluated	y	n/a	Support Vector Machine, Boosting	REG:ITK(MI); BE:BET; BFC:histogram matching; smoothing:Gaussian kernel	n/a	n/a	n	n	WML	-	45	3 (ACCORD-MIND)	n/a	Manual, 2		VC; other
C	2006	R. Harmouche et al.	Bayesian MS Lesion Classification Modeling Regional and Local Spatial Information	18th International Conference on Pattern Recognition (ICPR'06)	Inter-rater agreement evaluated	y	T1, T2, PD	Bayesian classification, Markov Random Field	BFC:N3; REG:ref; BE:ref; IN:ref	n/a	n/a	n	n	MS	MS	10	3	n/a	Manual, 5	majority	DSC
C	2005	K. M. Iftekharuddin et al.	Automatic brain tumor detection in MRI: methodology and statistical validation	SPIE Medical Imaging		n	T1	Self Organising Maps, Artificial neural networks	n/a	n/a	n/a	n	n	BT	-	50	3	n	Manual		ROC; DSC; Jcc; other
C	2005	M. Schmidt et al.	Segmenting brain tumors using alignment-based features	International Conference on Machine Learning and Applications		n/a	n/a	Support Vector Machine	denoising:non-linear filtering; BFC:N3; IN:weighted regression; REG:SPM	n/a	n/a	n	n	BT	-	10	3	y	Manual, 1		Jcc
C	2004	W. Li et al.	Automatic segmentation of cerebral ischemic lesions from diffusion tensor MR images	Medical Imaging 2004: Image Processing	Inter-rater agreement evaluated	n	DTI	Other	denoising:non-linear filtering	n/a	n/a	n	n	S	S	20	3	y	Manual		SI
C	2003	A. Hadjiprocopis and P. Tofts	An automatic lesion segmentation method for fast spin echo magnetic resonance images using an ensemble of neural networks	IEEE XIII Workshop on Neural Networks for Signal Processing		y	T1, T2, PD	Artificial neural networks	n/a	n/a	n/a	n	n	MS	-	20	3	y	Manual		Sen; Spe
C	2003	W. Li and J. Tian	Automatic segmentation of brain infarction in diffusion-weighted MR images	Medical Imaging 2003: Image Processing	Inter-rater agreement evaluated	n	DWI	Other	denoising:ADF; REG:global-rigid + local-non-rigid	n/a	n/a	n	n	S	-	20	3	y	Manual		DSC
C	2013	J. Huang et al.	Brain Tumor Segmentation Based on Texture, Intensity, and Edge	World Congress on Medical Physics and Biomedical Engineering		y	T1c, T2	Other	n/a	n/a	n/a	n	n	BT	-	19	3	n/a	Manual, multiple		TP; FP; FN; other
J	2012	Z. Karimaghaloo et al.	Automatic Detection of Gadolinium-Enhancing Multiple Sclerosis Lesions in Brain MRI Using Conditional Random Fields	IEEE Transactions on Medical Imaging		y	T1, T1c, T2, PD, FLAIR	Conditional Random Field, Graph Cut	REG:ref; BFC:N3; IN:histogram matching; BE:ref	2.67GHz quad-core P4 desktop	~5 sec	n	n	MS	MS	80	3	n/a	Manual, 2	consensus	Sen; FP; PPV
J	2017	Y. Liu et al.	A deep convolutional neural network-based automatic delineation strategy for multiple brain metastases stereotactic radiosurgery	PloS one		intended monomodality	T1c, FLAIR	Artificial neural networks	BE:ROBEX	NVIDIA Quadro M2000M Intel Xeon E3-1505	<2 min	n	n	MET	-	265; 240	1 (BraTS); 3 (University of Texas Southwestern Medical Center, Wardak et al. 2016)	y	Manual, 1		DSC; avgnSSD; sdSSD; Sen; Spe; ROC
J	2017	S. Mitra et al.	Volumetric brain tumour detection from MRI using visual saliency	PloS one		y	T1c, T2, FLAIR	Other	IN; 8bit range	n/a	n/a	n	n	BT	LGG; HGG	274	1 (BraTS'15)	y	-		ROC
C	2016	M. Agn et al.	A generative model for segmentation of tumor and organs-at-risk for radiation therapy planning of glioblastoma patients	SPIE Medical ImagingMedical Imaging 2016: Image Processing	Organs at Risk segmentation	y	T1c, T2, FLAIR	Artificial neural networks	REG	Core i5-2400 3.1GHz CPU	2 hrs	n	n	BT	LGG, HGG; GBM	30; 20	1 (BraTS'13); 3 (Rigshospitalet, Copenhagen University Hospital)	y	Manual		DSC; TPR; PPV
J	2019	K. R. Laukamp et al.	Fully automated detection and segmentation of meningiomas using deep learning on routine multiparametric MRI	European Radiology		y	T1, T1c, T2, FLAIR	Artificial neural networks	BFC; REG; BE; resampling; IN:0u1v	n/a	n/a	n	n	BT	meningioma	136	3 (University Hospital of Cologne)	y	Semiautomatic		DSC
J	2015	A. Karimian and S. Jafari	A New Method to Segment the Multiple Sclerosis Lesions on Brain Magnetic Resonance Images	J Med Signals Sens		y	T1, T2, FLAIR	GMM	BFC:LPF; BE:morphological operations	n/a	n/a	n	n	MS	-	25	3	y	Manual		DSC; Acc; Sen; Spe
J	2014	R. Wang et al.	Automatic Segmentation and Quantitative Analysis of White Matter Hyperintensities on FLAIR Images Using Trimmed-Likelihood Estimator	Academic Radiology		n	FLAIR	Other	BFC:N3; BE:BET	n/a	n/a	n	y	WML	-	82	3	n	Manual, 1		DSC; FPR; FNR; VR; VD
J	2018	X. Zhao et al.	A deep learning model integrating FCNNs and CRFs for brain tumor segmentation	Medical Image Analysis		y	T1, T1c, T2, FLAIR	Artificial neural networks, Conditional Random Field	IN:in paper; BFC:N4	Tesla K80 GPU, Inter E5-2620 CPU	2-4 min	n	n	BT	LGG, HGG	65+31; 274+110; 191	1 (BraTS'13); 1 (BraTS'15); 1 (BraTS'16)	y	-		DSC; PPV; Sen
C	2013	L. Zhao et al.	Automatic brain tumor segmentation with MRF on supervoxels	Multimodal Brain Tumor Segmentation		y	T1, T1c, T2, FLAIR	Supervoxel, Markov Random Field, Graph Cut	Denoising: SUSAN; IN: z-score	n/a	n/a	n	n	BT	LGG; HGG	65+31	1 (BraTS'13)	y	-		DSC; Spe; Sen
J	2018	R. Zhng et al.	Automatic segmentation of acute ischemic stroke from DWI using 3D Fully Convolutional DenseNets	IEEE Trans Med Imaging		y	DTI, ADC	Artificial neural networks	IN:0u1v, zero-padding, cropping, resampling	I7-4790 3.6GHz, 16GB RAM CPU, Nvidia Titan X GPU	~0.1 sec	n	n	S	S	242; 64	1 (ISLES'15); 3	y	Manual, 2	supervision	DSC; Sen; F1; PPV; Sen; VD
J	2017	T. Zhan et al.	Multimodal spatial-based segmentation framework for white matter lesions in multi-sequence magnetic resonance images	Biomedical Signal Processing and Control		y	T1, T2, FLAIR	Logistic Regression, Markov Random Field, Bayesian classification	BE: BET; BFC: N4; IN:linear contrast adjustment	n/a	n/a	n	n	WML	-	50; 54	1 (MS GC MICCAI'08); 3 (ACCORD-MIND)	y	Manual, 1		DSC; TPR; TNR; VD; AD; ROC
C	2012	E. Zacharaki et al.	Fuzzy Multichannel clustering with individualized spatial priors for segmenting brain lesions and infarcts	Artificial Intelligence Applications and Innovations		y	T1, FLAIR	K-Means, Fuzzy C-Means	BE; BFC; IN; REG:HAMMER	n/a	n/a	n	n	S, NEC	DM	47 (+72 healthy)	3	y	Manual, 1&2		DSC
C	2016	Y. Yoo et al.	Deep learning of brain lesion patterns for predicting future disease activity in patients with early symptoms of multiple sclerosis	Deep Learning and Data Labeling for Medical Applications		y	T2, PD	Artificial neural networks, Euclidean Distance Transform	BE; IN: linear; REF: affine	n/a	n/a	n	n	MS	early MS	140	3	n/a	Semiautomatic		Acc; Sen; Spe; ROC
preprint	2016	D. Yi, et al.	3-D convolutional neural networks for glioblastoma segmentation	arXiv preprint		y	T1, T1c, T2, FLAIR	Artificial neural networks	n/a	n/a	n/a	n	n	BT	LGG; HGG	274 + n	1 (BraTS'13); 1 (BraTS'15)	y	-		DSC
J	2014	W. Wu et al.	Brain tumor detection and segmentation in a conditional random fields framework with pixel-pairwise affinity and superpixel-level features	International Journal of Computer Assisted Radiology and Surgery		y	T1, T1c, T2, FLAIR	Superpixel, Gabor wavelets, SVM, Conditional Random Field	n/a	single desktop PC	30 min	n	n	BT	GBM	20; 30 (+25 synthetic)	1 (BraTS'12); 3 (Corso et al. 2008)	y	n/a		Acc; PPV; Sen; Jcc; DSC
J	2007	C. Vijayakumar et al.	Segmentation and grading of brain tumors on apparent diffusion coefficient images using self-organizing maps	Computerized Medical Imaging and Graphics		y	ADC, T2, FLAIR	Self Organising Maps	REG; IN; BE	n/a	n/a	n	n	BT	LGG; HGG	10	3	n	Manual, 2		ROC; Sen; Spe
J	2008	R. Verma et al.	Multi-parametric tissue characterization of brain neoplasms and their recurrence using pattern classification of MR images	Academic Radiology		y	B0, DWI, FLAIR, T1, T1c, FA, ADC	Bayesian classification, SVM	BE:FSL; REG:FLIRT; IN: histogram matching	n/a	n/a	n	n	BT	HGG	14	3	y	Manual, 1		Sen; Spe; other
C	2015	S. Vaidya et al	Longitudinal multiple sclerosis lesion segmentation using 3D convolutional neural networks	2015 Longitudinal Multiple Sclerosis Lesion Segmentation Challenge		y	T1, T2, PD, FLAIR	Artificial neural networks	n/a	Nvidia Tesla K20 GPU	2 min	n	n	MS	MS	5xn; 14xn	1 (MSC'15)	n	-		DSC; Jcc; PPV; Sen; TPR; FPR; VD; SD; VC
J	2017	K. Usman et al	Brain tumor classification from multi-modality MRI using wavelets and machine learning	Pattern Analysis and Applications		y	T1, T1c, T2, FLAIR	Decision Tree	IN: histogram matching; bounding box of tumor	I5, 2.5GHz, 8 GB RAM	3 min	n	n	BT	LGG, HGG	30	1 (BraTS'13)	y	-		DSC; Jcc; Spe
C	2014	G. Urban et al.	Multi-modal brain tumor segmentation using deep convolutional neural networks	MICCAI BraTS (brain tumor segmentation) challenge		y	T1, T1c, T2, FLAIR	Artificial neural networks	IN: mean CSF normalization	n/a	n/a	n	n	BT	LGG, HGG	30	1 (BraTS'13)	y	-		DSC
J	2015	N. J. Tustison et al.	Optimal symmetric multimodal templates and concatenated random forests for supervised brain tumor segmentation (simplified) with ANTsR	Neuroinform		y	T1, T1c, T2, FLAIR	Decision Tree, MAP, Markov Random Field	IN: 0-1range	Single threaded cluster	2 hrs	n	y	BT	LGG, HGG	55	1 (BraTS'13)	Y	-		DSC; PPV; Sen
C	2013	T. Taylor et al.	Map-reduce enabled hidden Markov models for high throughput multimodal brain tumor segmentation	Multimodal Brain Tumor Segmentation		y	T1, T1c, T2, FLAIR	Hidden Markov Models	movement artefact removal; BFC; IN	n/a	~500 sec	n	n	BT	HGG	20 (+25 synthetic)	1 (BraTS'13)	y	-		DSC
C	2009	N. Subbanna et al.	MS lesion segmentation using Markov Random Fields	MICCAI 2009		y	T1, T2, PD	Markov Random Field	BFC: N3; REG:ref; BE:ref; IN	n/a	n/a	n	n	MS	MS	24	3	n	Manual, 5	consensus	DSC; FPR; FNR
C	2015	N. Subbanna et al.	IMaGe: Iterative multilevel probabilistic graphical model for detection and segmentation of multiple sclerosis lesions in brain MRI	Information Processing in Medical Imaging		y	T1, T2, PD, FLAIR	Markov Random Field	BFC: N3; REG:ref; BE:FSL; IN:histogram matching	Dell Optiplex 980 i7	~40 min	n	n	MS	MS	1195	3	y	Semiautomatic		PPV; Sen; DSC
C	2008	J. C. Souplet	An automatic segmentation of T2-FLAIR multiple sclerosis lesions	MICCAI 2008		y	T1, T2, FLAIR	Expectation-Maximization, outlier detection	cropping; BE:ref; BFC:ref	n/a	96 min	n	y	MS	MS	25	1 (MS GC MICCAI'08)	y	-		VD; AD; TP; FP
C	2016	R. Saha et al.	Brain tumor segmentation from multimodal mr images using rough sets	Computer Vision, Graphics, and Image Processing		y	T1c, T2, FLAIR	Rough set, k-means, Quadtree partitioning	n/a	n/a	n/a	n	n	BT	LGG, HGG	304	1 (BraTS'13); 1 (BraTS'15)	y	-		PPV; Sen; DSC

For peer review only

For peer review only - http://bmjopen.bmj.com/site/about/guidelines.xhtml

C	2007	J. Rexilius et al.	Multispectral brain tumor segmentation based on histogram model adaptation	SPIE Medical Imaging	Semiautomatic preprocessing (brain extraction)	y	T2, FLAIR	tumor model, region growing	movement artefact removal; REG: affine; BE: watershed	2GHz AMD Athlon	10 min	n	n	BT	-	12	3	y	Manual		Jcc
C	2012	K. K. Reddy	Confidence guided enhancing brain tumor segmentation in multi-parametric MRI	ISBI		y	T1, T1c, T2, FLAIR	SVM, AdaBoost, Level Set, Region Growing	n/a	n/a	n/a	n	n	BT	-	11	3	n	Manual, 1		TPR; FPR; DSC
C	2011	P. Raniga et al.	Local intensity model: An outlier detection framework with applications to white matter hyperintensity segmentation	ISBI		y	T2, FLAIR	local intensity model, outlier detection	BFC:ref; REG:rigid; IN: CSF and WM normalization	n/a	n/a	n	n	WML	MCI, AD	14 (+44 healthy elderly)	3 (AIBL, Ellis et al., 2009)	n	Manual, 1		DSC; Overlap fraction; Extra fraction; missed fraction
C	2016	R. P. Randhawa	Improving segment boundary classification for brain tumor segmentation and longitudinal disease progression	Brainlesion: Glioma, Multiple Sclerosis, Stroke and Traumatic Brain Injuries		y	T1, T1c, T2, FLAIR	Artificial neural networks	BFC:N4; IN:0u1v	n/a	6 min	n	n	BT	LGG, HGG	274	1 (BraTS'15)	y	-		DSC
C	2015	O. Puonti	Simultaneous whole-brain segmentation and white matter lesion detection using contrast-adaptive probabilistic models	Brainlesion: Glioma, Multiple Sclerosis, Stroke and Traumatic Brain Injuries	Minimal preprocessing	y	T1, T2, FLAIR	Restricted Boltzmann machine, GMM	none	Quad-core Xeon 5472 3GHz 32 RM, GTX Titan 6 GB GPU	2 hrs	n	n	WML	MS	20	1 (MS GC MICCAI'08)	y	-		PPV; TPR
J	2006	K. B. Prakash	Identification, segmentation, and image property study of acute infarcts in diffusion-weighted images by using a probabilistic neural network and adaptive gaussian mixture model	Academic Radiology		n	DWI	Artificial neural networks, GMM	n/a	n/a	1 min	n	n	S	-	13	3	n/a	Semiautomatic		DSC; Sen; Spe
C	2015	V. Pedoia	Fully automatic brain tumor segmentation by using competitive EM and Graph Cut	Image Analysis and Processing – ICIAP		n/a	FLAIR	Competitive Expectation-Maximization, Graph cut	n/a	n/a	n/a	n	n	BT	LGG, HGG	20; 30 (+50 synthetic)	1 (BraTS'12); 3	y	Manual, 6		Jcc; DSC
C	2012	S. Parisot et al.	Graph-based Detection, Segmentation and Characterisation of Brain Tumours	IEEE Conference on Computer Vision and Pattern Recognition		n	FLAIR	Sparse graph	IN: mean and IQ based	n/a	n/a	n	n	BT	LGG	113	3	n/a	Manual		DSC
C	2016	B. Pandian et al.	Multimodal tumor segmentation with 3D convolutional neural networks	MICCAI BRATS		y	T1, T1c, T2, FLAIR	Artificial neural networks	BFC:N4; IN: 0u1v	4 core i7 32GB RAM, NVIDIA GTX 1080 GPU	n/a	n	n	BT	-	40	1 (BraTS'16)	y	-		DSC
C	2008	J. Morra et al.	Automatic segmentation of MS lesions using a contextual model for the MICCAI grand challenge	MICCAI 2008		y	T1, T2, FLAIR, DTI	Probabilistic boosting tree	downsampling, BFC: ref	n/a	n/a	n	n	MS	MS	25	1 (MS GC MICCAI'08)	y	-		VD; AD; Sen; Spe; PPV
C	2015	R. Meier, et al.	Parameter Learning for CRF-based Tissue Segmentation of Brain Tumors	Brainlesion: Glioma, Multiple Sclerosis, Stroke and Traumatic Brain Injuries		y	T1, T1c, T2, FLAIR	Conditional Random Field, Decision trees	n/a	n/a	n/a	n	n	BT	LGG, HGG	24x2; 20; 53	1 (BraTS'13); 1 (BraTS'15)	y	-		DSC; VE
C	2016	R. Meier, et al.	CRF-based brain tumor segmentation: alleviating the shrinking bias	Brainlesion: Glioma, Multiple Sclerosis, Stroke and Traumatic Brain Injuries		y	T1, T1c, T2, FLAIR	Dense Conditional Random Field, Decision trees	n/a	n/a	n/a	n	n	BT	LGG; HGG	51; 357; 25	1 (BraTS'13); 1 (BraTS'15)	y	-		DSC; VE
C	2016	O. Maier at al.	Image features for brain lesion segmentation using random forests	Brainlesion: Glioma, Multiple Sclerosis, Stroke and Traumatic Brain Injuries		y	T1, T1c, T2, FLAIR, DWI, CBF, CBV, TTP, Tmax	Random Forest	BFC:CMTK; IN:intensity harmonization	n/a	n/a	n	n	BT, S	S, LGG, HGG	104; 274	1 (BraTS'15); 1 (ISLES'15)	y	-		DSC; avgSSD; Hdff
C	2016	T .K. Lun et al.	Brain tumor segmentation using deep convolutional neural network	MICCAI BRATS		y	T1, T1c, T2, FLAIR	Artificial neural networks	n/a	n/a	n/a	n	n	BT	-	274	1 (BraTS'15)	y	-		DSC; VE
C	2016	L. Lefkovits et al.	Brain Tumor Segmentation with Optimized Random Forest	Brainlesion: Glioma, Multiple Sclerosis, Stroke and Traumatic Brain Injuries		y	T1, T1c, T2, FLAIR	Random Forest	denoising: ADF; BFC:N4; IN;histogram matching	n/a	n/a	n	n	BT	LGG; HGG	274	1 (BraTS'15)	y	-		DSC
J	2008	Z. Lao et al.	Computer-assisted segmentation of white matter lesions in 3D MR images using support vector machine	Academic Radiology	Inter-rater agreement evaluated	y	T1, T2, PD, FLAIR	SVM	REG:affine; BE:BET; BFC:N3; IN:histogram matching	n/a	n/a	n	n	WML	DM	45	3 (ACCORD-MIND)	y	Manual, 1		ROC; VC
C	2016	S. Kumar et al.	A picture fuzzy clustering approach for brain tumor segmentation	International Conference on Cognitive Computing and Information Processing (CCIP)		n/a	n/a	Fuzzy set	n/a	n/a	n/a	n	n	BT	LGG; HGG	65 (+65 synthetic)	1 (BraTS)	y	-		Jcc; DSC
C	2008	D. Kroon	Multiple sclerosis detection in multispectral magnetic resonance images with principal components analysis	Grand Challenge Work.: Mult. Scler. Lesion Segm. Challenge		y	T1, T2, FLAIR, MD, FA	PCA, log.likelihood ratio	BFC: various	n/a	n/a	n	n	MS	MS	44	1 (MS GC MICCAI'08)	y	-		DSC; VD; AD; Sen; Spe; PPV
C	2016	J. Knight et al.	MS lesion segmentation using FLAIR MRI only	MICCAI-MSSEG		n	FLAIR	edge detection, Fuzzy classification	BFC:SPM12; denoising:Gaussian LPF	n/a	n/a	n	n	MS	MS	15	1 (MS Lesion Segmentation Challenge '16)	y	-		DSC; PPV; TPR; Bland-Altman; ANCOVA
C	2007	H. Khotanlou et al.	Automatic brain tumor segmentation using symmetry analysis and deformable models	Advances in Pattern Recognition		n	T1	symmetry plane, histogram subtraction, deformable model	n/a	n/a	n/a	n	n	BT	-	10	3	n	Manual		Hdff; SD; other
C	2015	A. Jesson et al.	Hierarchical MRF and random forest segmentation of MS lesions and healthy tissue in brain MRI	Proceedings of the 2015 Longitudinal Multiple Sclerosis Lesion Segmentation Challenge		y	T1, T2, FLAIR	Random Forest, pathological atlases	IN:sigmoid; denoising:non-local means	n/a	n/a	n	n	MS	-	5x21	1 (MSC'15)	y	-		DSC; Jcc; TPR; FPR
C	2015	T. Jerman et al.	Combining unsupervised and supervised methods for lesion segmentation	Brainlesion: Glioma, Multiple Sclerosis, Stroke and Traumatic Brain Injuries		y	T1, T2, FLAIR	GMM, Decision trees	BE:reg; REG:affine; BFC:N4; IN:histogram matching; resampling:iso 1mm3 vox	n/a	n/a	n	n	MS	-	18	3 (University Medical Centre Ljubljana)	n	Manual, 2	consensus	DSC; TPR; PPV
J	2014	V. Ithapu et al.	Extracting and summarizing white matter hyperintensities using supervised segmentation methods in Alzheimer's disease risk and aging studies	Human Brain Mapping		n	FLAIR	SVM, Random Forest	REG; tissue seg:SPM12	n/a	n/a	n	y	WML	MCI, AD	38	3 (Wisconsin Alzheimer's Disease Research Center)	n	Semiautomatic		ROC; F score; other
J	2013	A. Islam et al.	Multifractal Texture Estimation for Detection and Segmentation of Brain Tumors	IEEE Transactions on Biomedical Engineering		y	T1, T2, FLAIR	boosting	REG:SPM8; BFC:SPM8; IN:histogram matching; BE:BET	n/a	n/a	n	n	BT	Astrocytoma, medulloblastoma	14 ;14	1 (BraTS'12); 3	y	Manual		ROC; DSC; Jcc; TI; other
J	2018	S. Iqbal et al.	Brain tumor segmentation in multi-spectral MRI using convolutional neural networks	Microscopy Research & Technique		y	T1, T1c, T2, FLAIR	Artificial neural networks	IN:0-255 range & mean subtraction; BFC:N4; cropping	Titan X 12 GB RAM GPU	<1 sec	n	n	BT	LGG, HGG	274	1 (BraTS'15)	y	-		DSC; Sen; Spe
J	2016	E. Ilunga-Mbuyamba et al.	Active contours driven by Cuckoo Search strategy for brain tumour images segmentation	Expert Systems with Applications		n	T1	Active Contour, Cuckoo Search Strategy	n/a	1.9GHz 4GB RAM	~180 sec	n	n	BT	GBM, MET	10	3 (University Hospital, Department of Neurosurgery, University of Leipzig, Germany)	n	Manual, 1		DSC; Jcc; Hdff
J	2012	K. M. Hulsey et al.	Automated quantification of white matter disease extent at 3T: Comparison with volumetric readings	Journal of Magnetic Resonance Imaging		y	MP-RAGE, FLAIR	thresholding	BE:FSL; tissue seg:Sienax; REG:affine	n/a	n/a	n	n	WML	-	28	3 (Dallas Heart Study, Ronald et al 2004)	n	Manual, 2		VC; VR; Bland-Altman
C	2015	A. Hoogi et al.	Multimodal Brain Tumor Segmentation (BRATS) using Sparse Coding and 2-layer Neural Network	MICCAI BRATS		y	T1, T1c, T2, FLAIR	Artificial neural networks, Sparse coding	IN: mean subtraction	n/a	n/a	n	n	BT	LGG, HGG	100	1 (BraTS'15)	y	-		DSC
J	2011	H. Khotanlou et al.	Segmentation of multiple sclerosis lesions in brain MR images using spatially constrained possibilistic fuzzy c-means classification	J Med Signals Sens		y	T1, T2	probabilistic fuzzy c-means	n/a	n/a	n/a	n	n	MS	-	10	3 (Imam Khomeini hospital)	n	Manual		DSC; Overlap fraction; Extra fraction
C	2015	T. Haeck et al.	Automated model-based segmentation of brain tumors in MR images	MICCAI BRATS		y	T1, T1c, T2, FLAIR	Expectation-Maximization, level set	BFC; Brain mask erosion	2 x 2.66 GHz Quad Core CPU	15 min	n	n	BT	LGG, HGG	274	1 (BraTS'15)	y	-		DSC
C	2015	T. Haeck et al.	Automated model-based segmentation of ischemic stroke in MR images	Brainlesion: Glioma, Multiple Sclerosis, Stroke and Traumatic Brain Injuries		y	T1, TTP, FLAIR	Expectation-Maximization, level set	none	2 x 2.66 GHz Quad Core CPU	15 min	n	n	S	-	64; 50	1 (ISLES'15)	y	-		DSC; avgSSD; Hdff; PPV; Sen
J	2018	N. Gupta et al.	Identification of gliomas from brain mri through adaptive segmentation and run length of centralized patterns	Journal of Computational Science		y	T1, T1c, T2, FLAIR	Image fusion, Thresholding	denoising: ADF	n/a	n/a	n	n	BT	LGG; HGG	80, 134	1 (BraTS'13); 3 (NSCB Medical College Jabalpur, India)	y	n/a		Jcc; DSC; Hdff; other
C	2014	M. Goetz et al.	Extremely randomized trees based brain tumor segmentation	MICCAI BRATS		y	T1, T1c, T2, FLAIR	Decision Tree	BFC:N4; IN:histogram matching + mode subtraction and 1v	n/a	n/a	n	n	BT	LGG; HGG	30	1 (BraTS'13)	y	-		DSC
J	2016	M. Ghafoorian et al.	Location sensitive deep convolutional neural networks for segmentation of white matter hyperintensities	Scientific Reports	Inter-rater agreement evaluated	y	T1, FLAIR	Artificial neural networks	REG:FLIRT; BE:BET; BFC:FAST; IN:0-1 range	n/a	n/a	n	n	WML	SVD	420	3 (RUNDMC, van Norden et al. 2011)	n	Manual		DSC; ROC
J	2017	M. Ghafoorian et al.	Deep multiscale location-aware 3d convolutional neural networks for automated detection of lacunes of presumed vascular origin	NeuroImage: Clinical	Inter-rater agreement evaluated	y	T1, FLAIR	Artificial neural networks	REG:FLIRT; BE:BET; BFC:FAST; IN:0-1 range	n/a	n/a	n	n	S	SVD; S	654; 421	3 (RUNDMC, van Norden et al. 2011); 3 (FUTURE, Rutten-Jacobs et al. 2011)	y	Manual, 1		FROC

For peer review only

C	2016	L. Le Folgoc et al.	Segmentation of brain tumors via cascades of lifted decision forests	MICCAI BRATS			y	T1, T1c, T2, FLAIR	Decision Tree, mixture model, Markov Random Field	n/a	Intel Xeon 3.6GHz 16GB RAM	~30 sec	n	n	BT	LGG; HGG	20; 200	1 (BraTS'13); 1 (BraTS'15)	y	-		DSC
C	2016	L. Le Folgoc et al.	Lifted auto-context forests for brain tumour segmentation	Brainlesion: Glioma, Multiple Sclerosis, Stroke and Traumatic Brain Injuries	semiautomatic preprocessing *brats 2016 contains some unpreprocessed or partially preprocessed images		y	T1, T1c, T2, FLAIR	Decision Tree, mixture model, Markov Random Field	IN:median and deviation; reg:rigid; BFC:ref; BE	Intel Xeon 3.6GHz 16GB RAM	~20 sec	n	n	BT	LGG; HGG	30;70; 274(?)	1 (BraTS'13); 1 (BraTS'15); 1 (BraTS'16)	y	-		DSC
J	2013	J. B. Fiot et l.	Efficient brain lesion segmentation using multi-modality tissue-based feature selection and support vector machines	International journal for numerical methods in biomedical engineering			y	T1, T2, PD, FLAIR	Support Vector Machine	REG:rigid; BE:ref; BFC:ref; denoising:ADF; tissue seg:EM	n/a	n/a	n	n	WML	-	125	3 (AIBL, Ellis et al., 2009)	n/a	Manual, 2	supervision	DSC; Sen; Spe
C	2013	J. Festa et al.	Automatic brain tumor segmentation of multi-sequence MR images using random decision forests	MICCAI BRATS			y	T1, T1c, T2, FLAIR	Random Forest	BFC:N4; IN:histogram matching	I7 3.2GHz 24 GB RAM	20-25 min	n	n	BT	LGG; HGG	30 +50 simulated	1 (BraTS'13)	y	-		DSC; Jcc; Sen; Spe; Kappa
C	2003	R. J. Ferrari et al.	Segmentation of multiple sclerosis lesions using support vector machines	SPIE Medical Imaging			y	T1, T2, FLAIR	Support Vector Machine	denoising: ADF; BFC:N3; IN:CSF linear normalization	2GHz 1GB RAM	~40 min	n	n	MS	-	18	3	n	Semiautomatic		Acc; FNR
C	2015	C. Feng et al.	Segmentation of ischemic stroke lesions in multi-spectral MR images using weighting suppressed FCM and three phase level set	Brainlesion: Glioma, Multiple Sclerosis, Stroke and Traumatic Brain Injuries			y	T1, T1c, T2, FLAIR, DWI, CBF, CBV, TTP, Tmax	Fuzzy C-Means, Image fusion, Level set	n/a	n/a	n/a	n	n	S	-	64; 50	1 (ISLES'15)	y	-		DSC; avgSSD; Hdff
C	2016	A. Ellawaa et al.	Brain tumor segmentation using random forest trained on iteratively selected patients	Brainlesion: Glioma, Multiple Sclerosis, Stroke and Traumatic Brain Injuries			y	T1, T1c, T2, FLAIR	Random Forest	BFC:N4; IN:histogram matching	n/a	n/a	n	n	BT	LGG; HGG	70?	1 (BraTS'16)	y	-		DSC; Sen; Spe
C	2015	P. Dvorak et al.	Local Structure Prediction with Convolutional Neural Networks for Multimodal BrainTumor Segmentation	Medical Computer Vision: Algorithms for Big Data			y	T1, T1c, T2, FLAIR	Artificial neural networks	n/a	4 core Intel Xeon E3 3.3GHz	13 sec	n	n	BT	LGG; HGG	309	1 (BraTS'14)	y	-		DSC
C	2013	S. Doyle et al.	Fully automatic brain tumor segmentation from multiple MR sequences using hidden Markov fields and variational EM	MICCAI BRATS			y	n/a	Markov Random Field, Expectation-Maximization	n/a	n/a	n/a	n	n	BT	LGG; HGG	30?	1 (BraTS'13)	y	-		DSC
J	2007	S. Datta et al.	Segmentation of gadolinium-enhanced lesions on MRI in multiple sclerosis	Journal of Magnetic Resonance Imaging	Semiautomatic preprocessing (brain extraction)		y	T1, T1c, FSE, FLAIR	Fuzzy connectedness, Other	REG:ref; BFC:SPM2; BE:ref; denoising:ADF	2.99GHz 1BG RAM	<10 min	n	n	MS	RRMS	22	3	n	Manual, 1		DSC; Bland-Altman; other
J	2012	S. Damangir et al.	Multispectral MRI segmentation of age related white matter changes using a cascade of support vector machines	Journal of Neurological Sciences			y	T1, FLAIR	Support Vector Machine	REG:FLIRT; BE:BET; BFC:N3; IN:linear transformation + 0-1 range	n/a	3 min	n	n	WML	AD, LBD	70 + 32 healthy	3 (he Dementia Study in Western Norway)	y	Manual		ROC; Sen; Spe; Acc; VC
C	2007	H. Cai et al.	Probabilistic segmentation of brain tumours based on multimodality magnetic resonance images	ISBI			y	T1, T1c, B0, DWI, FLAIR, DTI (FA, ADC)	Quadratic Discriminant Analysis, Support Vector Machine	BE:FSL; denoising:FSL; REG:FLIRT; IN:histogram matching	n/a	n/a	n	n	BT	-	22	3 (Hospital, University of Pennsylvania)	y	Expert segmentation		Acc
C	2012	C. P. Yu et al.	Statistical asymmetry-based brain tumor segmentation from 3D MR images	Biosignals	Segmentation failure		y	T1, T1c	Symmetry plane, Watershed	n/a	n/a	3 min	n	n	BT	-	17	3	n/a	Manual		Jcc
C	2016	P. Chang	Fully convolutional neural networks with hyperlocal features for brain tumor segmentation	MICCAI BRATS			y	T1, T1c, T2, FLAIR	Artificial neural networks	IN:histogram matching + 0-1 range	NVIDIA GTX TITAN X 12GB RAM	<1 sec	n	n	BT	HGG	144	1 (BraTS'16)	y	-		DSC; Hdff
J	2017	I. Cabria et al.	MRI segmentation fusion for brain tumor detection	Information Fusion			n	FLAIR	Potential Field Segmentation	n/a	n/a	n/a	n	n	BT	-	22	1 (BraTS'13)	n/a	-		other
C	2013	P. Buendia et al.	A grouping artificial immune network for segmentation of tumor images	MICCAI BRATS			y	T1, T1c, T2, FLAIR	Grouping Artificial Immune Network	denoising; BFC; IN:histogram matching	n/a	21 sec	n	n	BT	HGG	20	1 (BraTS'12)	y	-		DSC; Jcc; Sen; Spe; Kappa
J	2000	A. Boudra et al.	Automated segmentation of multiple sclerosis lesions in multispectral MR imaging using fuzzy clustering	Computers in Biology and Medicine			y	PD, T2	Fuzzy C-Means	enhancement:histogram equalization, BE:ref	n/a	n/a	n	n	MS	-	10	3	n	Manual, 2		other
C	2016	A. Birenbaum et al.	Longitudinal multiple sclerosis lesion segmentation using multi-view convolutional neural networks	Deep Learning and Data Labeling for Medical Applications			y	T1, T2, PD, FLAIR	Artificial neural networks	REG:rigid; BFC; IN:histogram matching	n/a	n/a	n	n	MS	-	19x4-6	1 (MSC'15)		-		DSC
C	2011	A. Bijar et al.	Segmentation of MS lesions using active contour model, adaptive mixtures method and mrf model	International Symposium on Image and Signal Processing and Analysis (ISPA)			y	T1, T1c, T2, FLAIR	Adaptive mixture model, Markov Random Field, Bayesian Classification	n/a	n/a	n/a	n	n	MS	-	20	3 (Khayati et al. 2008)	n/a	Manual, 2		DSC; Overlap fraction; Extra fraction
J	2011	A. Bijar et al.	Segmentation of MS lesions using entropy-based EM algorithm and Markov random fields	Journal of Biomedical Science and Engineering			y	n/a	GMM, Markov Random Field, Bayesian Classification, Expectation-Maximization	n/a	n/a	n/a	n	n	MS	-	20	3 (Khayati et al. 2008)	n/a	Manual, 2		DSC; Overlap fraction; Extra fraction
J	2008	K. B. Prakash	Automatic processing of diffusion-weighted ischemic stroke images based on divergence measures: Slice and hemisphere identification, and stroke region segmentation	International Journal of Computer Assisted Radiology and Surgery			n	DWI	intensity pdf divergence of 2 hemispheres	n/a	n/a	n/a	n	n	S	-	57	3	y	Manual, 1		ROC; DSC; Sen; Spe
preprint	2017	A. Beers et al.	Sequential 3d u-nets for biologically-informed brain tumor segmentation	arXiv preprint arXiv:1709.02967			y	T1, T1c, T2, FLAIR	Artificial neural networks	IN:0u1v	NVIDIA Tesla P100 GPU	n/a	n	n	BT	-	331	1 (BraTS'17)	y	-		DSC
C	2012	S. Bauer et al.	Segmentation of brain tumor images based on integrated hierarchical classification and regularization	MICCAI BRATS	method integrated in neuroradiology workflow		y	T1, T1c, T2, FLAIR	Random Forest, Conditional RandomFfield	n/a	n/a	4-12 min	n	n	BT	-	30?	1 (BraTS'12)	y	-		DSC; Jcc; Sen; Spe; AD; Hdff; Kappa
J	2013	A. J. Asman et al.	Of-Atlas likelihood estimation using multi-atlas segmentation	Med Phys			n	T1c	Intensity probability density function divergence of atlas	REG:FLIRT; IN: 0u1v + intensity mapping	n/a	<2 hrs	n	n	BT	-	30	3	n/a	Manual, 1		DSC; FNR; FPR
J	2008	P. Anbeek et al.	Automated MS-lesion segmentation by K-nearest neighbour classification	MIDAS Journal			y	T1, FLAIR	K-Nearest Neighbours	BE:BET	n/a	n/a	n	n	MS	-	44	3 (Children's Hospital Boston); 3 (University of North Carolina)	y	Manual, 2		VD; AD; TP; FP
J	2017	V. Alex et al.	Semisupervised learning using denoising autoencoders for brain lesion detection and segmentation	Journal of Medical Imaging			Y	T1, T1c, T2, FLAIR, DWI	Artificial neural networks	IN: histogram matching + 0u1v	n/a	n/a	n	n	BT	LGG; HGG; S	257; 28	1 (BraTS'15); 1 (ISLES'15)	y	-		DSC
C	2017	V. Alex et al.	Generative adversarial networks for brain lesion detection	SPIE Medical Imaging			y	T1, T1c, T2, FLAIR, DWI	Artificial neural networks	IN: histogram matching + 0u1v	n/a	n/a	n	n	BT	LGG; HGG; S	22	1 (BraTS); 1 (ISLES'15)	y	-		DSC; Acc
C	2008	G. Agam et al.	Probabilistic brain lesion segmentation in DT-MRI	International Conference on Image Processing			y	T1, T2, DTI	Mixture model, Expectation-Maximization	n/a	n/a	n/a	n	n	S	#VALUE!	21 (+19healthy)	3	n	Manual		PPV; Sen; DSC

References:

van Norden, Anouk GW, et al. "Diffusion tensor imaging and cognition in cerebral small vessel disease: the RUN DMC study." Biochimica et Biophysica Acta (BBA)-Molecular Basis of Disease 1822.3 (2012): 401-407.

Aalten, Pauline, et al. "The Dutch Parelsnoer Institute-Neurodegenerative diseases; methods, design and baseline results." BMC neurology 14.1 (2014): 1-8.

Bakas, Spyridon, et al. "Advancing the cancer genome atlas glioma MRI collections with expert segmentation labels and radiomic features." Scientific data 4 (2017): 170117.

Bowden, Donald W., et al. "Review of the Diabetes Heart Study (DHS) family of studies: a comprehensively examined sample for genetic and epidemiological studies of type 2 diabetes and its complications." The review of diabetic studies: RDS 7.3 (2010): 188.

Reid, Christopher M., et al. "Aspirin for the prevention of cognitive decline in the elderly: rationale and design of a neuro-vascular imaging study (ENVIS-ion)." BMC neurology 12.1 (2012): 1-9.

Chabriat, Hugues, et al. "Predictors of clinical worsening in cerebral autosomal dominant arteriopathy with subcortical infarcts and leukoencephalopathy: prospective cohort study." Stroke 47.1 (2016): 4-11.

Clark, Kenneth, et al. "The Cancer Imaging Archive (TCIA): maintaining and operating a public information repository." Journal of digital imaging 26.6 (2013): 1045-1057.

De Leeuw, F. E., et al. "Prevalence of cerebral white matter lesions in elderly people: a population based magnetic resonance imaging study. The Rotterdam Scan Study." Journal of Neurology, Neurosurgery & Psychiatry 70.1 (2001): 9-14.

Firbank, Michael J., et al. "High resolution imaging of the medial temporal lobe in Alzheimer's disease and dementia with Lewy bodies." Journal of Alzheimer's disease 21.4 (2010): 1129-1140.

Hinton, Ladson, et al. "Recruitment of a community-based cohort for research on diversity and risk of dementia." Alzheimer disease and associated disorders 24.3 (2010): 234.

Hougaard, K. D., et al. "Remote ischemic perconditioning in thrombolysed stroke patients: randomized study of activating endogenous neuroprotection–design and MRI measurements." International Journal of Stroke 8.2 (2013): 141-146.

Hsieh FI, Lien LM, Chen ST, Bai CH, Sun MC, et al. (2010) Get with the Guidelines-Stroke performance indicators: Surveillance of stroke care in the Taiwan Stroke Registry: Get with the Guidelines-Stroke in Taiwan. Circulation 122: 1116–1123.

I-KNOW. "Integrating Information from Molecule to Man: Knowledge Discovery Accelerates Drug Development and Personalized Treatment in Acute Stroke." (2006).

Tremblay-Mercier, Jennifer, et al. "P4-070: A PROGRAM OF PRE-SYMPTOMATIC EVALUATION OF EXPERIMENTAL OR NOVEL TREATMENTS FOR ALZHEIMER'S DISEASE (PREVENT-AD): DESIGN, METHODS, AND PERSPECTIVES." Alzheimer's & Dementia 10 (2014): P808-P808.

Ellis, Kathryn A., et al. "The Australian Imaging, Biomarkers and Lifestyle (AIBL) study of aging: methodology and baseline characteristics of 1112 individuals recruited for a longitudinal study of Alzheimer's disease." International psychogeriatrics 21.4 (2009): 672-687.

Khayati R, Vafadust M, Towhidkhah F, Nabavi SM (2008) Fully automatic segmentation of multiple sclerosis lesions in brain MR FLAIR images using adaptive mixtures method and Markov random field model. Comput Biol Med 38 (3) 379–390.

Lee, J. H., et al. "Identification of pure subcortical vascular dementia using 11C-Pittsburgh compound B." Neurology 77.1 (2011): 18-25.

Prastawa, Marcel, Elizabeth Bullitt, and Guido Gerig. "Simulation of brain tumors in MR images for evaluation of segmentation efficacy." Medical image analysis 13.2 (2009): 297-311.

For peer review only

Machner, Björn, et al. "Impact of dynamic bottom-up features and top-down control on the visual exploration of moving real-world scenes in hemispatial neglect." Neuropsychologia 50.10 (2012): 2415-2425.

Meier, Oskar, et al. "Extra tree forests for sub-acute ischemic stroke lesion segmentation in MR sequences." Journal of neuroscience methods 240 (2015): 89-100.

Reijmer, Yael D., et al. "Microstructural white matter abnormalities and cognitive functioning in type 2 diabetes: a diffusion tensor imaging study." Diabetes care 36.1 (2013): 137-144.

Ropele, Stefan, et al. "Determinants of iron accumulation in deep grey matter of multiple sclerosis patients." Multiple Sclerosis Journal 20.13 (2014): 1692-1698.

Rothwell P.M., Coull A.J., Giles M.F., Howard S.C., Silver L.E., Bull L.M., Gutnikov S.A., Edwards P., Mant D., Sackley C.M., Farmer A., Sandercock P.A., Dennis M.S., Warlow C.P., Bamford J.M., Anslow P., Oxford Vascular S. Change in stroke incidence, mortality, case-fatality, severity, and risk factors in Oxfordshire, UK from 1981 to 2004 (Oxford Vascular Study) Lancet. 2004;363:1925–1933.

Sachdev, Perminder S., et al. "The contribution of twins to the study of cognitive ageing and dementia: the Older Australian Twins Study." International Review of Psychiatry 25.6 (2013): 738-747.

Sachdev, Perminder S., et al. "The Sydney Memory and Ageing Study (MAS): methodology and baseline medical and neuropsychiatric characteristics of an elderly epidemiological non-demented cohort of Australians aged 70-90 years." International psychogeriatrics 22.8 (2010): 1248.

Selnes, P., et al. (2013). Diffusion tensor imaging surpasses cerebrospinal fluid as predictor of cognitive decline and medial temporal lobe atrophy in subjective cognitive impairment and mild cognitive impairment. Journal of Alzheimer's Disease, 33(3), 723–736.

Walker, William C., et al. "The Chronic Effects of Neurotrauma Consortium (CENC) multi-centre observational study: description of study and characteristics of early participants." Brain injury 30.12 (2016): 1469-1480.

Zamboni G., Wilcock G.K., Douaud G., Drazich E., McCulloch E., Filippini N., Tracey I., Brooks J.C., Smith S.M., Jenkinson M., Mackay C.E. Resting functional connectivity reveals residual functional activity in Alzheimer's disease. Biol. Psychiatry. 2013;74:375–383.

Pantoni, Leonardo, et al. "Impact of age-related cerebral white matter changes on the transition to disability–the LADIS study: rationale, design and methodology." Neuroepidemiology 24.1-2 (2005): 51-62.

Shepherd, James, et al. "The design of a prospective study of pravastatin in the elderly at risk (PROSPER)." The American journal of cardiology 84.10 (1999): 1192-1197.

Weiner, MD, Howard L. "Oral tolerance for the treatment of autoimmune diseases." Annual review of medicine 48.1 (1997): 341-351.

"European project on brain morphometry,", BIOMORPH, EU-BIOMED2 project nr. BMH4-CT96-0845, 1996–1998.

Price, Catherine C., et al. "MRI-leukoaraiosis thresholds and the phenotypic expression of dementia." Neurology 79.8 (2012): 734-740.

Zacharaki, E.I., Wang, S., Chawla, S., Yoo, D.S., Wolf, R., Melhem, E.R., Davatzikos, C.: Classification of brain tumor type and grade using MRI texture and shape in a machine learning scheme. Magnetic Resonance in Medicine 62, 1609–1618 (2009)

Marias, K., Dionysiou, D., Sakkalis, V., Graf, N., Bohle, R.M., Coveney, P.V., Wan, S., Folarin, A., Buechler, P., Reyes, M., Clapworthy, G., Liu, E., Sabczynski, J., Bily, T., Roniotis, A., Tsiknakis, M., Giatili, S., Veith, C., Messe, E., Stenzhorn, H., Kim, Y.j., Zasada, S., Haidar, A.N., Bauer, S., Wang, T., Zhao, Y., Karasek, M., Grewer, R., Franz, A., Stamatakos, G.: Clinically driven design of multi-scale cancer models: the ContraCancrum project paradigm. J. Roy. Soc. Interface Fo- cus 1(3), 450–461 (2011)

Wardak, Zabi, et al. "Pre-treatment factors associated with detecting additional brain metastases at stereotactic radiosurgery." Journal of neuro-oncology 128.2 (2016): 251-257.

Victor, Ronald G., et al. "The Dallas Heart Study: a population-based probability sample for the multidisciplinary study of ethnic differences in cardiovascular health." The American journal of cardiology 93.12 (2004): 1473-1480.

Rutten-Jacobs, Loes CA, et al. "Risk factors and prognosis of young stroke. The FUTURE study: a prospective cohort study. Study rationale and protocol." BMC neurology 11.1 (2011): 109.

Links to access the referenced databases

MSC'15	https://smart-stats-tools.org/lesion-challenge
WMH Segmentation Challenge '17	https://wmh.isi.uu.nl/
MS Lesion Segmentation Challenge 2016	https://portal.fli-iam.irisa.fr/msseg-challenge/overview
NACC	https://www.alz.washington.edu/
IBSR	https://www.nitrc.org/projects/ibsr
SPL	https://spl.harvard.edu/software-and-data-sets
BrainWeb	http://www.bic.mni.mcgill.ca/brainweb/
Maier et al. 2015	https://figshare.com/articles/dataset/Sub_acute_Ischemic_Stroke_MRI_scans_T1_T2_DWI_ADC_ground_truth_and_segmentation_results/1585018
Hermes	https://clinicaltrials.gov/ct2/show/NCT00875654

For peer review only

Preferred Reporting Items for Systematic reviews and Meta-Analyses extension for Scoping Reviews (PRISMA-ScR) Checklist

SECTION	ITEM	PRISMA-ScR CHECKLIST ITEM	REPORTED ON PAGE #
**TITLE**			
Title	1	Identify the report as a scoping review.	1
**ABSTRACT**			
Structured summary	2	Provide a structured summary that includes (as applicable): background, objectives, eligibility criteria, sources of evidence, charting methods, results, and conclusions that relate to the review questions and objectives.	2
**INTRODUCTION**			
Rationale	3	Describe the rationale for the review in the context of what is already known. Explain why the review questions/objectives lend themselves to a scoping review approach.	3
Objectives	4	Provide an explicit statement of the questions and objectives being addressed with reference to their key elements (e.g., population or participants, concepts, and context) or other relevant key elements used to conceptualize the review questions and/or objectives.	3
**METHODS**			
Protocol and registration	5	Indicate whether a review protocol exists; state if and where it can be accessed (e.g., a Web address); and if available, provide registration information, including the registration number.	3
Eligibility criteria	6	Specify characteristics of the sources of evidence used as eligibility criteria (e.g., years considered, language, and publication status), and provide a rationale.	4-5
Information sources*	7	Describe all information sources in the search (e.g., databases with dates of coverage and contact with authors to identify additional sources), as well as the date the most recent search was executed.	4
Search	8	Present the full electronic search strategy for at least 1 database, including any limits used, such that it could be repeated.	-
Selection of sources of evidence†	9	State the process for selecting sources of evidence (i.e., screening and eligibility) included in the scoping review.	4-5
Data charting process‡	10	Describe the methods of charting data from the included sources of evidence (e.g., calibrated forms or forms that have been tested by the team before their use, and whether data charting was done independently or in duplicate) and any processes for obtaining and confirming data from investigators.	5
Data items	11	List and define all variables for which data were sought and any assumptions and simplifications made.	5
Critical appraisal of individual sources of evidence§	12	If done, provide a rationale for conducting a critical appraisal of included sources of evidence; describe the methods used and how this information was used in any data synthesis (if appropriate).	-
Synthesis of results	13	Describe the methods of handling and summarizing the data that were charted.	5

For peer review only - http://bmjopen.bmj.com/site/about/guidelines.xhtml

SECTION	ITEM	PRISMA-ScR CHECKLIST ITEM	REPORTED ON PAGE #
**RESULTS**			
Selection of sources of evidence	14	Give numbers of sources of evidence screened, assessed for eligibility, and included in the review, with reasons for exclusions at each stage, ideally using a flow diagram.	6
Characteristics of sources of evidence	15	For each source of evidence, present characteristics for which data were charted and provide the citations.	-
Critical appraisal within sources of evidence	16	If done, present data on critical appraisal of included sources of evidence (see item 12).	-
Results of individual sources of evidence	17	For each included source of evidence, present the relevant data that were charted that relate to the review questions and objectives.	-
Synthesis of results	18	Summarize and/or present the charting results as they relate to the review questions and objectives.	5-8
**DISCUSSION**			
Summary of evidence	19	Summarize the main results (including an overview of concepts, themes, and types of evidence available), link to the review questions and objectives, and consider the relevance to key groups.	8
Limitations	20	Discuss the limitations of the scoping review process.	11
Conclusions	21	Provide a general interpretation of the results with respect to the review questions and objectives, as well as potential implications and/or next steps.	13
**FUNDING**			
Funding	22	Describe sources of funding for the included sources of evidence, as well as sources of funding for the scoping review. Describe the role of the funders of the scoping review.	-

JBI = Joanna Briggs Institute; PRISMA-ScR = Preferred Reporting Items for Systematic reviews and Meta-Analyses extension for Scoping Reviews.
* Where *sources of evidence* (see second footnote) are compiled from, such as bibliographic databases, social media platforms, and Web sites.
† A more inclusive/heterogeneous term used to account for the different types of evidence or data sources (e.g., quantitative and/or qualitative research, expert opinion, and policy documents) that may be eligible in a scoping review as opposed to only studies. This is not to be confused with *information sources* (see first footnote).
‡ The frameworks by Arksey and O'Malley (6) and Levac and colleagues (7) and the JBI guidance (4, 5) refer to the process of data extraction in a scoping review as data charting.
§ The process of systematically examining research evidence to assess its validity, results, and relevance before using it to inform a decision. This term is used for items 12 and 19 instead of "risk of bias" (which is more applicable to systematic reviews of interventions) to include and acknowledge the various sources of evidence that may be used in a scoping review (e.g., quantitative and/or qualitative research, expert opinion, and policy document).

From: Tricco AC, Lillie E, Zarin W, O'Brien KK, Colquhoun H, Levac D, et al. PRISMA Extension for Scoping Reviews (PRISMAScR): Checklist and Explanation. Ann Intern Med. 2018;169:467–473. doi: 10.7326/M18-0850.

For peer review only - http://bmjopen.bmj.com/site/about/guidelines.xhtml
